# Precision installation of silyl synthetic handles within arenes by regiocontrolled ruthenium C(*sp*^2^)–H functionalization

**DOI:** 10.1038/s41929-025-01309-6

**Published:** 2025-04-02

**Authors:** Jamie H. Docherty, Mishra Deepak Hareram, Luke M. Nichols, Ignacio Pérez-Ortega, Iñigo J. Vitorica-Yrezabal, Igor Larrosa

**Affiliations:** 1 Department of Chemistry, University of Manchester, Manchester, UK; 2 Department of Chemistry, Lancaster University, Lancaster, UK

**Keywords:** Homogeneous catalysis, Catalytic mechanisms, Reaction mechanisms

## Abstract

The site-selective functionalization of C(*sp*^2^)–H bonds represents a powerful strategy for the synthesis of structurally diverse compounds with broad applicability. Here we report efficient regioselective catalytic methods for the formation of benzyltrimethylsilanes through ruthenium-catalysed C(*sp*^2^)–H silylmethylation. The developed protocols enable selective functionalization at both *ortho* and *meta* positions within arenes bearing N-based directing groups. The resulting silylmethyl compounds can undergo diverse transformations, including nucleophilic aromatic substitution, carbonyl addition, olefination and desilylation. Significantly, the regiodivergent installation of silylmethyl synthetic handles allows for the synthesis of the pharmaceutical losmapimod and could further be applied in direct late-stage functionalizations. Mechanistically, an essential role for biscyclometallated ruthenium(II) species has been found, with the formation of intermediate ruthenium(III) species indicated by paramagnetic NMR experiments. These synthetic inventions and mechanistic elucidations signify a transformative step within ruthenium-catalysed C(*sp*^2^)–H functionalization, enabling diverse syntheses and providing a framework for future development.

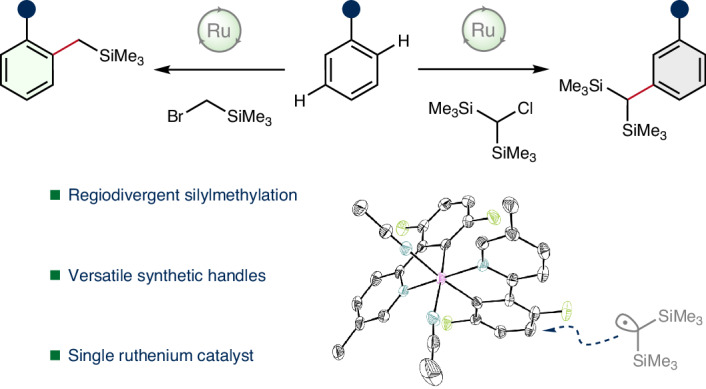

## Abstract (web summary)

The site-selective functionalization of C(*sp*^2^)–H bonds is a powerful strategy for the synthesis of structurally diverse compounds with broad applicability. Here the authors show ruthenium-catalysed regiodivergent methods for the installation of silyl synthetic handles at both *ortho* and *meta* sites within arenes.

## Main

**Fig. 1:**
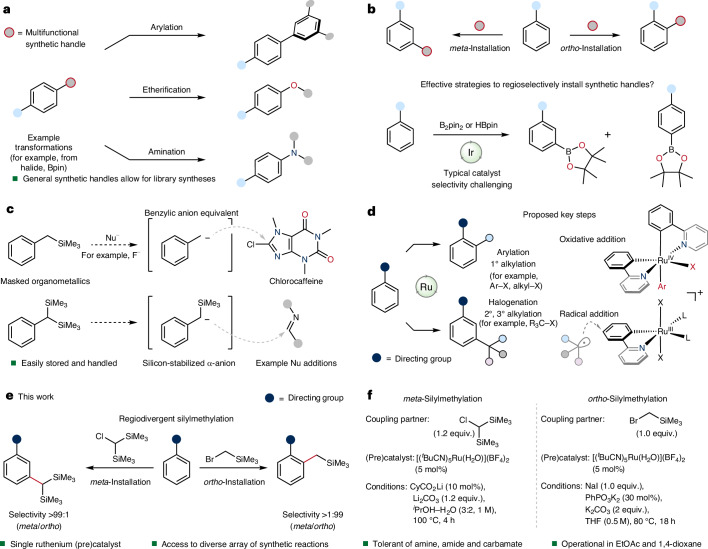
Concepts and strategies for the installation of synthetic handles within arenes and their utility. **a**, Generic utility of multifunctional synthetic handles within chemical transformations. **b**, Regiochemical challenges associated with C(*sp*^2^)–H functionalization reactions. Pinacolboronic esters have proven broad synthetic utility in a variety of transformations; however, installation by metal-catalysed C(*sp*^2^)–H borylation has required innovative strategies for regiocontrol. Bpin, 4,4,5,5-tetramethyl-1,3,2-dioxaborolane. **c**, Synthetic utility of benzylic trimethylsilanes as masked anion equivalents. Nu, nucleophile. **d**, Overview of mechanistic pathways for the proposed ruthenium-catalysed C(*sp*^2^)–H functionalization leading to regiodivergent outcomes. 1°, primary; 2°, secondary; 3°, tertiary. **e**, Regiodivergent silylmethylation enabled by a single ruthenium catalyst, [(^*t*^BuCN)_5_Ru(H_2_O)](BF_4_)_2_, providing site-selective access to both *ortho*- and *meta*-silylmethylated arenes. **f**, Reaction conditions for both *meta*- and *ortho*-silylmethylation.

Highly regioselective catalytic protocols have served as powerful tools for the transformation of both simple and complex molecules into structures of profound utility across diverse fields such as pharmaceuticals and materials science^1–6^. One critical tenet in synthetic chemistry has been the effective incorporation of multifunctional synthetic handles within organic molecules^7–14^. These handles can be used to introduce structural diversity by serving as general reactive coupling partners or reagents, substantially broadening the range of strategies available to synthetic practitioners (Fig. 1a). Therefore, the ability to selectively install versatile handles into densely functionalized substrates offers compelling advantages, such as allowing for general arrays of diverse late-stage modifications^15,16^. As such, these approaches represent powerful strategies for enabling library syntheses and facilitating accelerated exploration of chemical space.

Given their synthetic versatility, boronic esters and acids have emerged as exemplar multifunctional handles for a plethora of synthetic manipulations^17–19^. The C(*sp*^2^)–H borylation of arenes represents an efficient example of their synthetic preparation, which has been achieved using metal-catalysed^20–22^, metal-free^23^ and radical-mediated processes^24^ (Fig. 1b). However, maintaining control over regioselectivity within these reactions presents a primary challenge and has necessitated the development of innovative strategies^25^.

Alternatives have emerged to complement the utility of boron-based compounds, for example, silicon-based synthetic handles have attracted interest for their ability to serve as useful pro-nucleophiles and coupling partners^26–29^. The synthetic applicability of silyl groups is analogous to that of boronic esters with potentially wide synthetic suitability for a varied selection of synthetic transformations. Benzylic silanes in particular hold considerable promise due to their ability to serve as masked benzylic anion equivalents (Fig. 1c)^30–34^. This reactivity allows the silyl group to be readily unmasked using fluoride or alkoxide reagents, unveiling species capable of acting as general nucleophiles towards a diverse set of electrophiles. Moreover, the utility of benzylic silanes extends beyond anion reactivity, with this class of compounds additionally serving as robust precursors for benzylic radicals^35,36^. However, classical synthetic strategies for their preparation have required the use of highly reactive halosilanes, such as chlorotrimethylsilane, and organometallic reagents^37–41^.

Considering the growing synthetic utility exhibited by silyl synthetic handles, we questioned whether ruthenium-catalysed C(*sp*^2^)–H functionalization might serve as an effective method for installing silylmethyl groups. We reasoned that, with a ruthenium catalyst, we could achieve site-selective installation of this class of synthetic handle at both *ortho* and *meta* sites within arenes bearing N-based directing groups^42,43^. This approach relied on the emerging evidence supporting the ability to obtain high levels of site selectivity in ruthenium-catalysed C(*sp*^2^)–H functionalization reactions. For instance, prevailing studies suggest that aryl halide and primary alkyl halide electrophiles preferentially react to give *ortho*-C(*sp*^2^)–H functionalization, while secondary and tertiary alkyl halides favour *meta* addition (Fig. 1d)^44–48^. The regiocontrol in these transformations is therefore commonly predicated on the structure of the electrophile, thus providing a framework for precision functionalization. We posited that if these observations hold universally, we could strategically use primary and secondary halo(silyl)methane reagents for a regiodivergent set of C(*sp*^2^)–H silylmethylation reactions. While differentiating between *ortho* and *meta* positions was feasible based on substrate design, we recognized the challenge of obtaining good reactivity with sterically encumbered electrophiles (for example, SiMe_3_, *A*-value = 2.5) as well as limiting the formation of over-addition products.

In this report, we present ruthenium-catalysed procedures for the site-selective installation of these useful silyl synthetic handles at *ortho* and *meta* sites within arenes bearing N-based directing groups and demonstrate their utility through a series of synthetic transformations. Regioselective installation was achieved using (bromomethyl)trimethylsilane for *ortho* selectivity and bis(trimethylsilyl)chloromethane for *meta* selectivity (Fig. 1e). In each case, optimized reaction conditions allowed for both excellent levels of reactivity as well as regioselectivity (Fig. 1f). These synthetic inventions underscore the powerful capabilities of ruthenium catalysis and the broad utility of silicon-based synthetic handles that serve to expand the breadth of downstream structures.

## Results

### Identification of *ortho*-silylmethylation reaction conditions

**Fig. 2:**
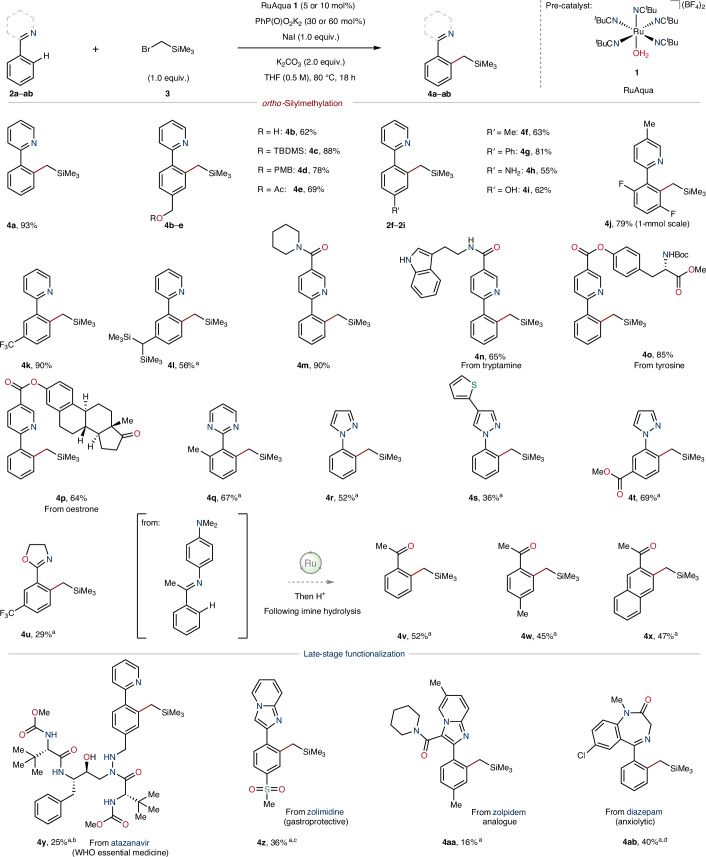
Application of ruthenium-catalysed *ortho*-silylmethylation to a range of arenes. Reaction scope using (bromomethyl)trimethylsilane (**3**). Reactions were performed on a 0.25-mmol scale using 5 mol% [Ru] unless otherwise noted. All yields correspond to isolated compounds. ^a^10 mol% [Ru] and 60 mol% PhP(O)O_2_K_2_ were used. ^b^36-µmol scale. ^c^180-µmol scale. ^d^87-µmol scale. TBDMS, *tert*-butyldimethylsilyl; PMB, *para*-methoxybenzyl; Ac, acetyl; Boc, *tert*-butyloxycarbonyl; WHO, World Health Organization.

To discover reaction conditions for *ortho*-silylmethylation, we systematically assessed key reaction variables such as choice of (pre)catalyst, base, additives and solvent. Principally, *ortho*-alkylation reactions using halide electrophiles have used (pre)catalysts based on [(*p*-cymene)RuCl_2_]_2_ and its derivatives due to their widespread commercial availability. However, this class of (pre)catalyst possesses a substantial barrier to activation and consequently necessitates the use of high temperatures^45^ (≳100 °C) or light irradiation^46^. For *ortho*-silylmethylation, we selected (bromomethyl)trimethylsilane (**3**) as electrophile and 2-phenylpyridine (**2a**) as a model substrate (Fig. 2). Reaction at 80 °C in THF using [(*p*-cymene)RuCl_2_]_2_ or analogues such as [(C_6_H_6_)RuCl_2_]_2_ gave only low levels of reactivity (17% and 14% yield of **4a**, respectively, Supplementary Table 7). Key for high levels of reactivity was the use of [(^*t*^BuCN)_5_Ru(H_2_O)](BF_4_)_2_ (RuAqua, **1**) as (pre)catalyst, which gave **4a** in excellent yield (93%). We recently reported the robust air and moisture stability of **1** as well as its diverse reactivity enabled by its more labile ligand sphere^49^.

While polar aprotic solvents such as *N*-methyl-2-pyrrolidone (NMP) have been used previously to facilitate ruthenium-catalysed *ortho*-alkylation reactions^47^, we found that *ortho*-silylmethylation could be performed in a range of solvents, including ethyl acetate, dioxane and THF (Supplementary Table 6). Crucial to both high levels of reactivity and selectivity was the addition of NaI. We observed that in the absence of NaI, considerable *meta*-silylmethylation occurred as well as the formation of over-addition products (Supplementary Tables 1–4).

### Applicability of *ortho*-silylmethylation

To assess the generality of the developed *ortho*-silylmethylation reaction, a selection of arenes bearing N-heterocycle groups were subjected to the reaction conditions (Fig. 2). Free benzylic alcohol **2b** and analogues with silyl (**2c)**, *para*-methoxybenzyl (**2d**) and acetyl (**2e**) protecting groups all underwent selective *ortho*-silylmethylation in high-to-excellent yields. Similarly, 4-substituted free aniline **2h** and phenol **2i** were well tolerated, giving the corresponding silylmethylated species **4h** and **4i** in good yields (55% and 62%, respectively). 1,2,5-Trisubstituted arene **2j** was also *ortho*-silylmethylated on a 1-mmol scale to give **4j** in excellent yield (79%), highlighting the ability of the developed protocol to functionalize polysubstituted substrates. Arenes bearing *meta* electron-withdrawing (**2k**) and electron-donating (**2l**) substituents underwent productive functionalization, albeit with **2l** requiring increased catalyst loading (10 mol%). Amides **2m**,**n** and esters **2o**,**p**, including those containing structural units derived from biologically active compounds, also participated in *ortho*-silylmethylation, generally in good-to-excellent yields.

We recognized that it was key to demonstrate the value of this synthetic invention in both the context of broad functional group tolerance and its applicability across a diverse range of structures. Therefore we opted to look beyond pyridine-based N-heterocycles and explored the reactivity of pyrimidine, pyrazole, oxazoline, imine and related structures. Such broad applicability would be key to enable streamlined syntheses of a wide array of structures and thus allow accelerated discovery and exploration of chemical space. Accordingly, pyrimidine **2q** was subjected to the established reaction conditions, selectively giving the expected product **4q** in good yield (65%). Similarly, pyrazoles **2r**–**t** underwent successful *ortho*-silylmethylation to give **4r**–**t** in moderate-to-good yields (36–69%). The formal *ortho*-silylmethylation of ketones was facilitated using imines as temporary directing groups. Specifically, ketimines **2v**–**x** derived from 4-(dimethylamino)aniline were used to give the corresponding *ortho*-functionalized products. After acid hydrolysis, successful isolation of the *ortho*-silylmethylated ketones **4v**–**x** was achieved in appreciable yields (45–52%).

One powerful application of any C–H functionalization methodology is its capacity to functionalize targets bearing substantial functional group density and diversity. In particular, late-stage functionalization of biologically active compounds has served as a valuable tool for the rapid generation of medicinally relevant analogues. The *ortho*-silylmethylation protocol was therefore applied to a range of pharmaceutical targets (Fig. 1, bottom). Anti-retroviral compound atazanavir (**2y**) underwent successful *ortho*-silylmethylation to give **4y** in a low but useful yield (25%). Imidazo[1,2-*a*]pyridine-containing zolimidine (**2z**) and zolpidem analogue **2aa** similarly reacted to selectively give **4z** and **4aa** in yields of 36% and 16%, respectively. Lastly, the well-known anxiolytic diazepam (**2ab**) containing the tetrahydrobenzo[*e*][1,4]diazepin-2-one directing group underwent silylmethylation to give **4ab** in good yield (40%), with the transformation tolerant of the halide handle within **2ab**.

### Identification of *meta*-silylmethylation reaction conditions

In contrast to the *ortho* reactivity obtained with (bromomethyl)trimethylsilane (**3**), the use of bis(trimethylsilyl)chloromethane (**5**) necessitated an elevated temperature (100 °C) and an aqueous solvent mixture (^*i*^PrOH–H_2_O, 3:2) for good levels of *meta*-selective reactivity (Supplementary Tables 9–16). Akin to *ortho*-silylmethylation, the use of RuAqua (**1**) as the (pre)catalyst proved effective for *meta*-silylmethylation using **5**, allowing for the use of a single (pre)catalyst for both *ortho*- and *meta*-silylmethylation reactions. At the outset, we determined functional-group tolerance through a robustness screen^50^ under the optimized reaction conditions. For this screening process, we used 2-phenylpyridine (**2a**) as a model substrate and bis(trimethylsilyl)chloromethane (**5**; Supplementary Table 16). This rapid survey of additive compounds highlighted the tolerance towards amide, carbamate and amine groups, amongst others.

### Applicability of *meta*-silylmethylation

**Fig. 3:**
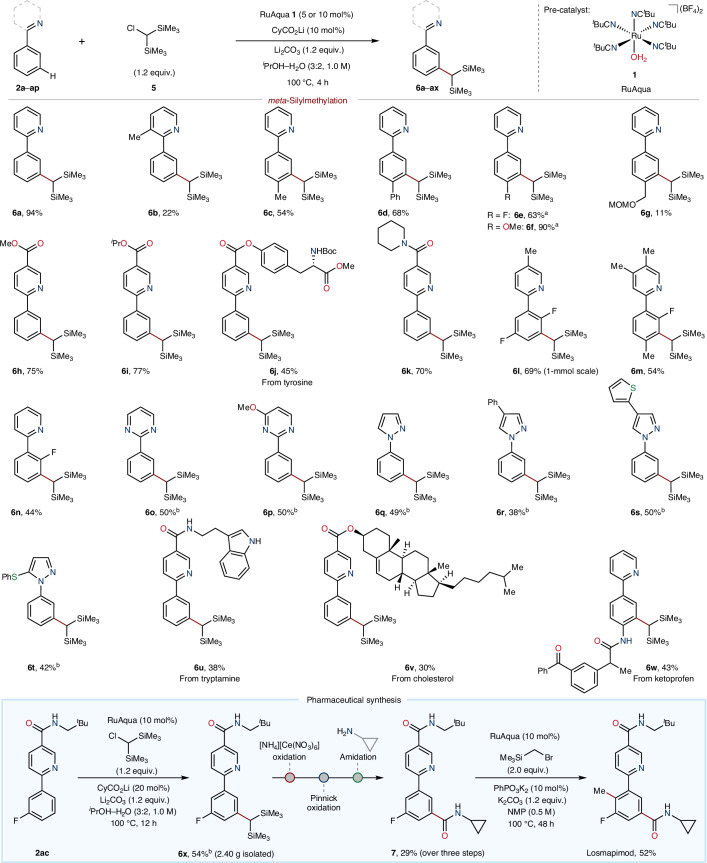
Application of ruthenium-catalysed *meta*-silylmethylation to a range of arenes and utility in pharmaceutical synthesis. Reaction scope using bis(trimethylsilyl)chloromethane (**5**). Reactions were performed on a 0.25-mmol scale using (5 mol%) [Ru] unless otherwise noted. All yields correspond to isolated compounds. The reaction yield for the [NH_4_][Ce(NO_3_)_6_] oxidation of **6x** on a gram scale was 57%, for Pinnick oxidation the yield was 70% and for amidation using cyclopropylamine the yield was 72%. ^a^Isolated as the desilylated compound following reaction with excess tetrabutylammonium fluoride. ^b^10 mol% [Ru] was used. Cy, cyclohexyl.

To further evaluate the generality of the developed *meta*-silylmethylation reaction, the optimized reaction conditions were applied to a selection of arenes bearing N-heterocycles (Fig. 3). Specifically, a range of substrates bearing pyridyl directing groups were subjected to the established reaction conditions. These reactions resulted in successful *meta*-silylmethylation using **5** to give the corresponding products **6a**–**m**, generally in good-to-excellent yields. Despite the presence of the sterically demanding geminal trimethylsilyl groups within electrophile **5**, installation could still be achieved at the *meta* position of *para*-substituted arylpyridines, as exemplified by the formation of products **6c**–**g**. A range of ester-containing arenes were successfully *meta*-silylmethylated using **5** to give **6h**–**j** in good yields (45–77%). The functional-group compatibility highlighted in the robustness screen was corroborated in the successful synthesis of tertiary amide **6k** in high yield (70%). The methodology was successfully expanded to include other N-heterocycles, specifically, arylpyrimidines, which gave the corresponding *meta*-silylmethylated compounds **6o**,**p**. Moreover, a selection of arylpyrazoles underwent successful functionalization, giving products **6q**–**t** in moderate-to-good yields (38–50%).

This ruthenium-catalysed method was further applied in the synthesis of the pharmaceutical compound losmapimod (**8**), which holds promise for the treatment of facioscapulohumeral muscular dystrophy^51^. Initially, arylpyridine **2ac** was *meta*-silylmethylated using the established reaction conditions to selectively give a multigram quantity of product **6x** in good yield (2.4 g, 54%). This compound **6x** was then oxidized to a carboxylic acid, followed by amide coupling with cyclopropylamine to give **7**. Application of the *ortho*-silylmethylation conditions to **7** using silane **3** in NMP selectively gave losmapimod (**8**) in 52% yield (Fig. 3, bottom).

### Synthetic utility of benzyltrimethylsilanes

**Fig. 4:**
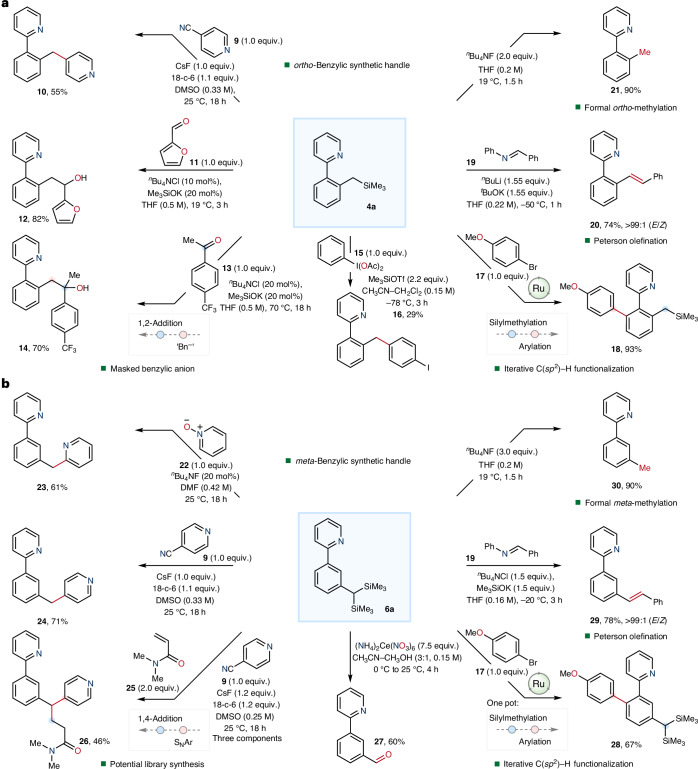
Synthetic utility of benzylic trimethylsilane and benzylic bis(trimethylsilane). **a**, Reactions showing the silyl group as a useful *ortho*-synthetic handle for diverse transformations. **b**, Reactions showing the bis(silyl) group as a useful *meta*-synthetic handle for diverse transformations. Benzylic anion reactivity was unveiled using fluoride or siloxide (KOSiMe_3_), reacting through S_N_Ar towards cyanopyridine or with pyridine *N*-oxide. Unmasking the silyl group also allowed for nucleophilic addition into carbonyl electrophiles. The reaction of **4a** with excess tetrabutylammonium fluoride gave *ortho*-tolylpyridine **21**, the product of formal *ortho*-methylation, while the reaction of **6a** gave *meta*-tolylpyridine **30**, the product of formal *meta*-methylation. Reaction conditions for the direct arylation of **4a** to give **18**: **17** (1.0 equiv.), **1** (10 mol%), KOAc (30 mol%), K_2_CO_3_ (2.0 equiv.), NMP (1.0 M), 35 °C, 24 h. Reaction conditions for the synthesis of **28** in a one-pot sequence: **17** (1.0 equiv.), **1** (5 mol%), CyCO_2_Li (10 mol%), Li_2_CO_3_ (1.2 equiv.), ^*i*^PrOH–H_2_O (3:2, 1.0 M), 100 °C, 4 h. 18-c-6, 18-crown-6; DMSO, dimethylsulfoxide.

Selective C(*sp*^2^)–H silylmethylation offers a synthetically useful step for the generation of benzylic trimethylsilanes that can be further elaborated by several strategies to produce diverse compounds. To highlight the synthetic utility of the products formed from both *ortho*- and *meta*-silylmethylation reactions, these were subjected to several different functionalization reactions (Fig. 4a). *ortho*-Silylmethylated compound **4a** was subjected to the conditions reported by Reidl and Bandar for nucleophilic aromatic substitution (S_N_Ar) coupling with 4-cyanopyridine (**9**), which gave coupled product **10** in good yield (55%)^33^. Similarly, benzylic anion reactivity was achieved using the conditions of Das and O’Shea^30^, which enabled nucleophilic addition into aldehyde **11** to give homobenzylic alcohol **12** in excellent yield (82%). Similar reactivity was achieved when applied to ketone **13**, which gave the corresponding alcohol **14**, once again in high yield (70%). *para*-Selective C(*sp*^2^)–H benzylation of (diacetoxyiodo)benzene **15** was achieved by reaction of **4a** in the presence of excess trimethylsilyl triflate to give **16** in moderate yield (29%)^52^. The benzylic trimethylsilane **4a** also served as a suitable precursor for Peterson-type olefination using imine **19** to give alkene **20** in excellent yield and diastereoselectivity (74%, >99:1 *E*/*Z*)^34^. The *ortho*-silylmethylation reaction could also serve as a valuable procedure for the selective formal *ortho*-methylation of arenes by reaction of **4a** with excess tetrabutylammonium fluoride to give *ortho*-methyl compound **21** in excellent yield (90%).

Analogous to the reactivity observed using the *ortho*-silylmethyl synthetic handle within **4a**, the geminal bis(trimethylsilyl)methane group in **6a** could also be used as a pro-nucleophile (Fig. 4b). To illustrate, **6a** underwent reaction with pyridine *N*-oxide (**22**) to give the coupled product **23** in good yield (61%)^32^. Application of Reidl and Bandar’s conditions enabled S_N_Ar with **9** to give the coupled compound **24** in high yield (71%)^33^. Iterative S_N_Ar using **9** and 1,4-addition with acrylamide **25** was achieved under similar conditions to give **26** in appreciable yield (46%). Formal arene *meta*-formylation to produce **27** was realized following conversion of the *gem*-silyl handle within **6a** using excess ammonium ceric nitrate. Direct olefination of the *gem*-silyl handle using imine **19** gave *E*-alkene **29** in high yield and excellent diastereoselectivity (78%, >99:1 *E*/*Z*)^31^. Similarly to the *ortho*-silyl group, the *meta*-silyl handle in **6a** also underwent protodesilylation using excess tetrabutylammonium fluoride to give *meta*-methyl compound **30** in excellent yield (90%). This reactivity therefore demonstrates a viable strategy for the formal C(*sp*^2^)–H *meta*-methylation of arenes. Taken together, the *meta*-selective silylmethylation reaction provides generic access to a broad suite of functional groups, thus offering a transformative approach to the diversification of aromatic compounds.

### Mechanistic considerations

**Fig. 5:**
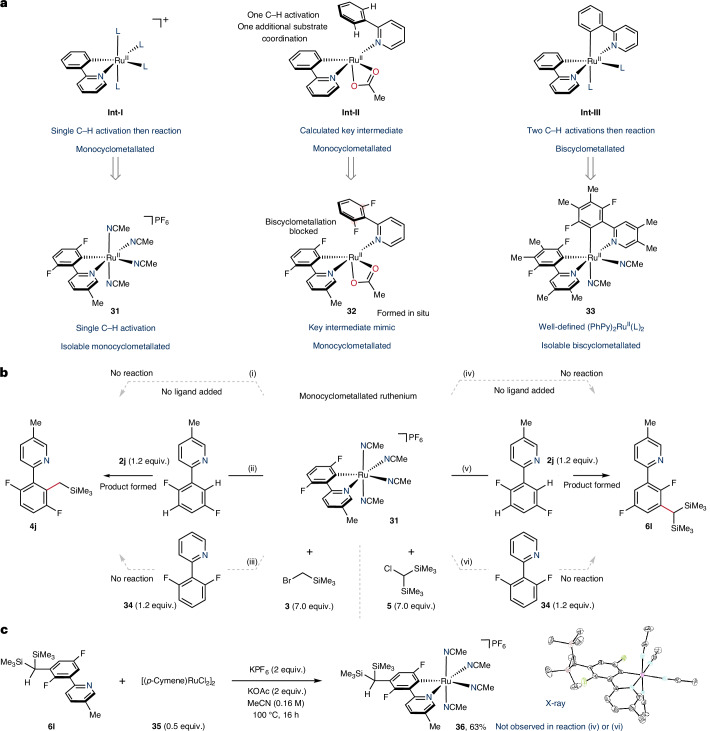
Mechanistic considerations for the key ruthenacycle intermediates responsible for reactivity. **a**, Proposed ruthenium intermediates include monocyclometallated ruthenium species with and without the interaction of other arenes. Alternatively, two C(*sp*^2^)–H activation steps give a bisruthenacycle that can serve as a reactive intermediate. **b**, Stoichiometric reactivity of monocyclometallated ruthenacycle **31**. Reaction conditions: (i) **3** (7.0 equiv.), KOAc (10 equiv.), (CD_3_)_2_CO (0.1 M), 40 °C, (ii) with added **2j** (1.2 equiv.), (iii) with added **34** (1.2 equiv.); (iv) **5** (5.0 equiv.), KOAc (10 equiv.), (CD_3_)_2_CO (0.1 M), 40 °C, (v) with added **2j** (1.2 equiv.), (vi) with added **34** (1.2 equiv.). Reactivity was only observed when an additional equivalent of arene bearing *ortho*-C(*sp*^2^)–H bonds was present. **c**, Independent synthesis of monoruthenacycle **36** to confirm the absence of its formation in mechanistic experiments. An X-ray structure of **36** is shown. with thermal ellipsoids at the 50% probability level.

Both *ortho*- and *meta*-selective C(*sp*^2^)–H functionalization reactions using ruthenium catalysts have been proposed to proceed through intermediate cyclometallated species (Fig. 5a). Mechanistic hypotheses have suggested the involvement of mono- and biscyclometallated ruthenium(II) species (for example, **Int-I** to **Int-III**) as key reactive intermediates that are formed before reaction with halide electrophiles^45–47^. Based on density functional theory calculations, Ackermann and co-workers proposed that monocyclometallated ruthenium(II) complexes (that is, with additional acetate and **2a** coordination, **Int-II**) react favourably by inner-sphere single-electron transfer (SET) with 1-bromohexane (Gibbs energy of activation, ∆*G*^‡^ = 16.6 kcal mol^−1^) and *tert-*butyl bromide (∆*G*^‡^ = 15.6 kcal mol^−1^)^53^. These values contrast with those determined for biscyclometallated species (for example, **Int-III**). For this class of complex, the reactions with primary and tertiary alkyl bromides were calculated to have considerably higher barriers (∆*G*^‡^ = 22.9 and 22.1 kcal mol^−1^, respectively). Therefore, these calculations suggest a marked difference in the reactivity of the monocyclometallated and biscyclometallated species.

To probe these mechanistic proposals in the context of *ortho*- and *meta*-silylmethylation, monocyclometallated ruthenium(II) complex **31** was subjected to a series of control experiments (Fig. 5b). When complex **31** was heated at 40 °C with *ortho*-selective bromide **3**, no reaction was observed (Fig. 5b(i)). In contrast, reaction in the presence of added arylpyridine **2j** gave the *ortho*-silylmethylation product **4j** (Fig. 5b(ii)). The addition of arylpyridine **2j** allows for further cyclometallation and formation of a biscyclometallated species (that is, complex **40**), which was observed in the reaction mixture (Supplementary Figs. 15 and 16). Similarly, to mimic the intermediate species proposed by Ackermann and co-workers, we conducted the reaction with added 2-(2,6-difluorophenyl)pyridine (**34**; Fig. 5b(iii)). The *ortho*-fluorine substitution within **34** precludes cyclometallation while still allowing for pyridine coordination; however, no silylmethylation was observed.

Similarly for *meta*-silylmethylation reactivity, in the stoichiometric reaction of monocyclometallated ruthenium(II) complex **31** at 40 °C with *meta*-selective chloride **5**, neither product **6l** nor its cyclometallated analogue (for example, **36**) was observed (Fig. 5b(iv)). However, in the presence of added arylpyridine **2j**, the same reaction gave the *meta*-silylmethylation product **6l** (Fig. 5b(v)). This outcome was not replicated when the same experiment was conducted with **34** (Fig. 5b(vi)). The independent synthesis of the cyclometallated ruthenium(II) complex bearing product **6l** facilitated direct comparison of the reaction spectra to exclude the formation of this type of species in the absence of added arylpyridine **2j** (Fig. 5c). Importantly, these experiments were reproducible with analogous monocyclometallated complexes bearing distinct substituents (Supplementary Fig. 17). These mechanistic observations were suggestive of a key role for the formation and reactivity of a biscyclometallated species, which stands in contrast to previously proposed pathways.

### Reactivity of biscyclometallated ruthenium(II) complexes

**Fig. 6:**
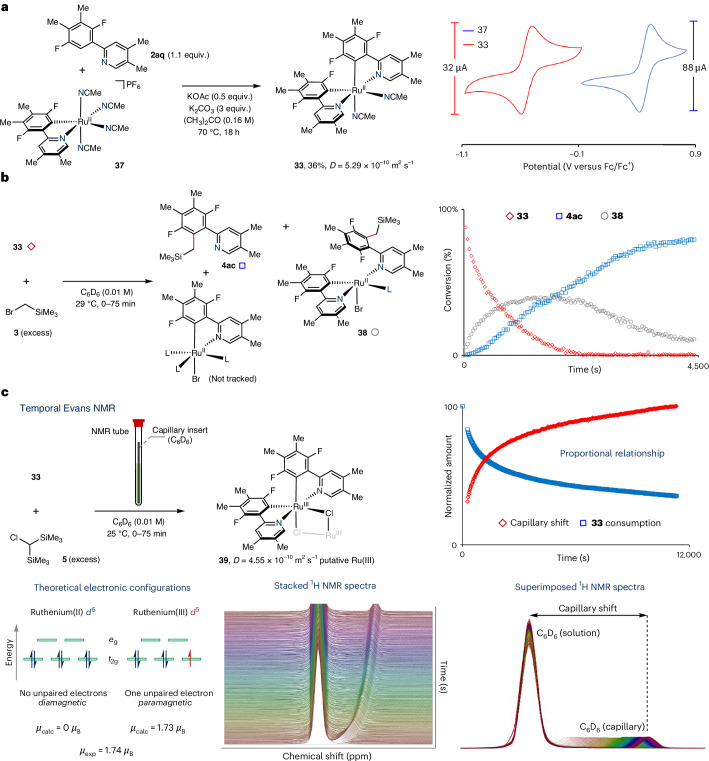
Stoichiometric reactivity of a reactive bisruthenacycle. **a**, Synthesis of bisruthenacycle **33** from monoruthenacycle **37** and comparison of the CVs of both species (see Supplementary Figs. 41 and 42 for full CVs). Fc, ferrocene. **b**, Stoichiometric reaction of bisruthenacycle **33** with (bromomethyl)trimethylsilane (**3**). **c**, Temporally resolved Evans NMR experiment for the reaction of **33** with bis(trimethylsilyl)chloromethane (**5**). A capillary containing [D_6_]benzene was inserted within the NMR tube containing the reactants. The consumption of bisruthenacycle **33** was monitored over time by ^1^H NMR spectroscopy and the divergence in the *δ*(solution) versus *δ*(capillary) signals measured from the maximum peak positions at each time point. *D*, diffusion coefficient, measured by diffusion-ordered NMR spectroscopy; *µ*, magnetic moment, measured in Bohr magnetons *µ*_B_ (*µ*_B_ = 9.27 × 10^−24^ J T^−1^); calc, calculated; exp, experimental.

Following the observed inactivity exhibited by monocyclometallated ruthenium(II) complexes, we next examined the stoichiometric reactivity of a biscyclometallated ruthenium(II) species. Thus, we initially synthesized biscyclometallated complex **33** from its monocyclometallated precursor **37** (Fig. 6a). This complex was treated with excess (bromomethyl)trimethylsilane (**3**) in [D_6_]benzene at 29 °C, tracking the reaction progress by ^1^H NMR spectroscopy (Fig. 6b). Notably, the product **4ac** and its ruthenium(II) coordinated analogue **38** were observed, without the need for any additives or base, and in less than 1 h.

We similarly explored the use of biscyclometallated ruthenium(II) complex **33** to understand the reactivity exhibited in the *meta*-silylmethylation reaction using bis(trimethylsilyl)chloromethane (**5**). However, in complex **33**, the site where electrophile **5** would initiate carbon–carbon bond formation is impeded by a methyl substituent (*para* to ruthenium) and thus no reactivity was anticipated. Despite this, when **33** was treated with excess **5** at room temperature, we noted the formation of a paramagnetic species in solution. This was identified by characteristic ^1^H NMR resonances that fell outside the typical sweep width (that is, *δ*^1^H = 72.0, –14.8 and −19.3 ppm, see Supplementary Fig. 34).

To garner information on the number of unpaired electrons within the newly formed paramagnetic ruthenium species, we used the Evans NMR method (Fig. 6c)^54^. The reaction of **33** and **5** in [D_6_]benzene was conducted in a J Young NMR tube incorporating a sealed inner capillary containing [D_6_]benzene to serve as a reference. We monitored the progress of the reaction in real time using ^1^H NMR spectroscopy and observed a clear divergence in the resonance frequency of the [D_6_]benzene residuals (that is, the signal from the reaction mixture versus the signal from the capillary). From this, we determined a spin-only magnetic moment *μ*_eff_ of 1.74 *μ*_B_, which is supportive of a single unpaired electron (Supplementary Equation (1))^54^. Given the theoretical electronic configurations of an octahedral ruthenium(II) species (*d*^6^, diamagnetic) and a singly oxidized octahedral ruthenium(III) species (*d*^5^, paramagnetic), these observations are coherent with the formation of a ruthenium(III) species. Based on the diffusion-ordered NMR spectra and mass spectrometry data, the reaction of **33** and **5** generated a putative ruthenium(III) dimer **39** (Supplementary Figs. 37–39).

**Fig. 7:**
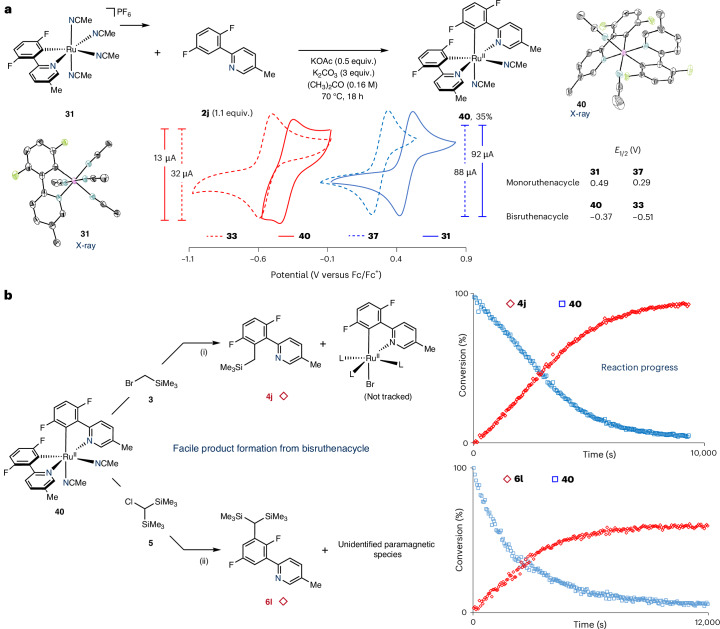
Synthesis and reactivity of a bisruthenacycle bearing available *meta* sites for functionalization. **a**, Synthesis of bisruthenacycle **40** from **31** and comparison of the Ru(II/III) redox potentials for both monoruthenacycles **31** and **37** and bisruthenacycles **40** and **33** (see Supplementary Figs. 39–42 for full CVs). The X-ray structures of **31** and **40** are shown with thermal ellipsoids at the 50% probability level. **b**, Stoichiometric reactivity of bisruthenacycle **40** with (bromomethyl)trimethylsilane (i) and bis(trimethylsilyl)chloromethane (ii). Reaction conditions (i) towards **4j**: (bromomethyl)trimethylsilane (7 equiv.), C_6_D_6_–(CD_3_)_2_CO (3:2, 0.05 M), 40 °C. Reaction conditions (ii) towards **6l**: bis(trimethylsilyl)chloromethane (18 equiv.), C_6_D_6_–(CD_3_)_2_CO (3:2, 0.05 M), 50 °C.

Given the limitations imposed by the densely substituted biscyclometallated species **33** to undergo *meta*-selective functionalization, we questioned the feasibility of producing an unsubstituted analogue. Biscyclometallated ruthenium(II) complex **40** was synthesized from its monocyclometallated analogue **31** and arylpyridine **2j** in useful yield (35%; Fig. 7a). Comparative cyclic voltammograms (CVs) of monocyclometallated ruthenium(II) complexes **31** and **37** and biscyclometallated ruthenium(II) complexes **40** and **33** showed distinctly different redox potentials. Specifically, the Ru(III)/Ru(II) redox potentials in **40** and **33 are** ***E***_1/2_ = −0.37 and −0.51 V, respectively, versus ferrocene/ferrocenium, while the Ru(III)/Ru(II) redox potentials in monocyclometallated **31** and **37 are** ***E***_1/2_ = 0.49 and 0.29 V, respectively, versus ferrocene/ferrocenium. The distinct differences in the redox potentials underscore the enhanced reducing properties of the biscyclometallated ruthenium(II) complexes compared with their monocyclometallated analogues (Δ*E*_1/2_ ≈ 0.8 V) and are consistent with the observed reactivity towards electrophiles.

Having synthetically established access to **40**, we treated this complex with *ortho*-silylmethylation reagent **3** at 40 °C, monitoring the reaction progress by ^1^H NMR spectroscopy (Fig. 7b, top). The formation of functionalized product **4j** was observed in conjunction with the consumption of complex **40**. Similarly, the reaction of *meta*-silylmethylation reagent **5** and complex **40** was also tracked using ^1^H NMR spectroscopy (Fig. 7b, bottom). In this instance, product **6l** was observed alongside uncharacterized paramagnetic species, concurrent with the consumption of complex **40**. The reactivities observed highlight the ability of the halide reagents **3** and **5** to readily engage with biscyclometallated complexes **40** and **33**. In stark contrast, no reactivity was observed when these reagents were applied to monocyclometallated ruthenium(II) analogues **31** and **37**. These findings are therefore supportive of a key role for biscyclometallated species during catalysis.

**Fig. 8:**
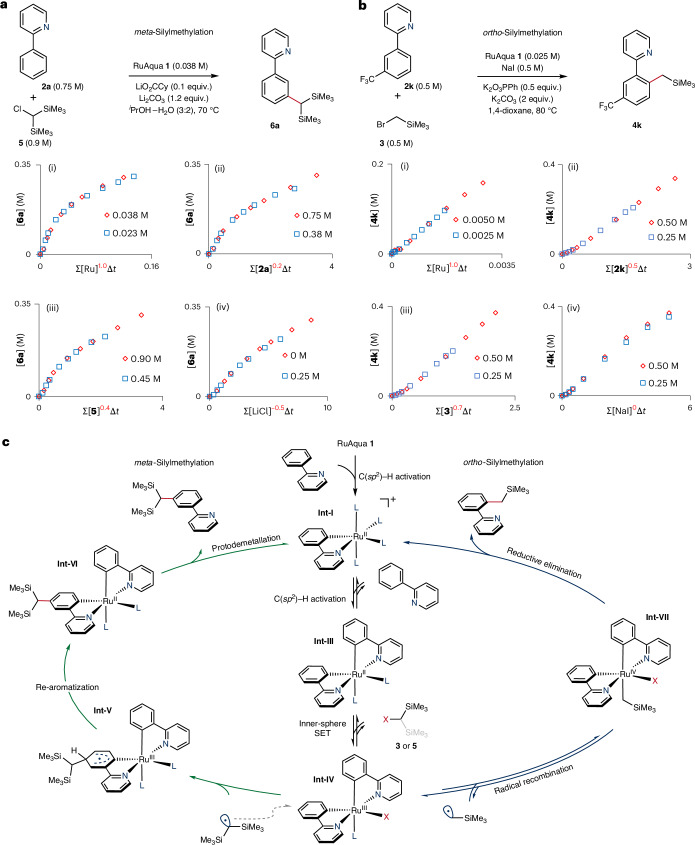
Variable-time normalization analysis for *ortho*- and *meta*-silylmethylation and overall mechanistic hypothesis. **a**,**b**, Normalized reaction plots to determine the kinetic orders for the ruthenium catalyst, arene, electrophile, lithium chloride and sodium iodide in the *meta*-silylmethylation (**a**) and *ortho*-silylmethylation (**b**) reactions. The progress in both reactions was monitored by gas chromatography with flame ionization detection using biphenyl as internal standard. **c**, Mechanistic overview of both *ortho* and *meta* selectivity in the silylmethylation reaction, diverting post-SET.

To gain further mechanistic insight, we measured the kinetic orders of the components in the reactions using variable-time normalization analysis^55^. For the *meta-*silylmethylation reaction, an order of 1 on the Ru catalyst, 0.2 on arene, 0.4 on electrophile **5** and −0.5 order on LiCl were observed (Fig. 8a), while for the *ortho*-silylmethylation reaction, an order of 1 on the Ru catalyst, 0.5 on arene, 0.7 on electrophile **3** and 0 order on NaI were observed (Fig. 8b).

Mechanistically, both pathways proceed by sequential C–H activation, forming **Int-III** via the monocyclometallated intermediate **Int-I** (Fig. 8c). **Int-III** was confirmed as the active catalytic intermediate through comparative stoichiometric experiments, showing the need for biscyclometallation over activation by N coordination (**Int-II**). The measured kinetic orders on the arene substrate of 0.2 and 0.5 for *meta*- and *ortho*-silylmethylation, respectively, are consistent with a reversible C–H activation step. This was also evidenced by the observation of H/D exchange in reactions carried out in the presence of D_2_O (Supplementary Information). While **Int-III** possesses considerably more reducing power than its predecessor **Int-I**, the reduction potentials of typical alkyl halides remain out of reach for an outer-sphere mechanism^56^. It is therefore proposed that the reaction proceeds via an inner-sphere complex, where the oxidation potential of the electrophile is reduced to an accessible value^57^. This pathway agrees with the partial orders measured for the electrophiles, which are indicative of a reversible reaction of the alkyl halide with a catalytic intermediate preceding the rate-determining step. The following SET to **Int-IV** was confirmed by Evans NMR experiments as well as by the observation of ^1^H NMR chemical shifts consistent with its formation. The divergence in reactivity originates from the propensity for the alkyl radicals to be sequestered by Ru in **Int-IV** (*ortho*) to form **Int-VII** or to attack the arene system (*meta*) to give **Int-V**.

## Conclusion

We have developed distinct site-selective synthetic methods for the installation of silylmethyl synthetic handles using ruthenium (pre)catalyst **1**. The versatility of the resulting silylmethyl products has been highlighted through their application to several different transformations, including protodesilylation of the installed handle to allow for formal arene *ortho*- and *meta*-methylation. Moreover, the regiodivergent installation of silylmethyl groups enables an alternative synthetic route to the active pharmaceutical losmapimod (**8**). This type of synthetic strategy using silyl synthetic handles allows broad composability for the potential synthesis of derivative compounds along the synthetic path. Given that the PubChem database contains more than 6 million (hetero)aromatic compounds bearing a suitable N(*sp*^2^) for *ortho*-C(*sp*^2^)–H cyclometallation, these synthetic inventions provide substantial utility. Mechanistic investigations conveyed the key role of reducing biscyclometallated ruthenium(II) species for reactivity with both primary and secondary electrophiles, with Evans NMR experiments revealing the formation of a putative ruthenium(III) species that is likely reflective of relevant intermediates formed during the catalytic process. By expanding the mechanistic understanding within ruthenium-catalysed C–H functionalization, these findings provide long-awaited insights that will aid the development of future protocols.

## Methods

### General procedure for the *meta*-silylmethylation of arenes

Arene (0.250 mmol), bis(trimethylsilyl)chloromethane (59.0 mg, 0.300 mmol, 1.20 equiv.), [(^*t*^BuCN)_5_Ru(H_2_O)](BF_4_)_2_ (9.0 mg, 13.0 µmol, 5 mol%), lithium carbonate (22.0 mg, 0.300 mmol, 1.20 equiv.) and lithium cyclohexanecarboxylate (3.4 mg, 25.0 µmol, 10 mol%) were added to a vial, which was sealed (crimp-capped) and purged for ~40 s with N_2_. N_2_-sparged isopropanol–water (3:2, 250 µl) was injected into the vial using a syringe and the reaction mixture was heated at 100 °C for 4 h. The reaction mixture was then cooled to room temperature, the cap was removed and the solvent removed in vacuo before direct purification by flash column chromatography.

### General procedure for the *ortho*-silylmethylation of arenes

Arene (0.250 mmol), (trimethylsilyl)bromomethane (41.8 mg, 0.250 mmol, 1.00 equiv.), [(^*t*^BuCN)_5_Ru(H_2_O)](BF_4_)_2_ (9.0 mg, 13.0 µmol, 5 mol%), potassium carbonate (69.0 mg, 0.500 mmol, 2.00 equiv.), potassium phenylphosphonate (17.5 mg, 75.0 µmol, 30 mol%) and sodium iodide (37.0 mg, 0.250 mmol, 1.00 equiv.) were added to a vial, which was sealed (crimp-capped) and purged for ~40 s with N_2_. Anhydrous THF (500 µl) was added using a syringe and the reaction mixture was heated at 80 °C for 18 h. The reaction mixture was then cooled to room temperature, the cap was removed and the solvent removed in vacuo before direct purification by flash column chromatography.

### Procedure for the synthesis of bis[2-(2,5-difluorophenyl)-5-methylpyridine]ruthenium(II) bis(acetonitrile) (40)

In a glove box under an atmosphere of purified argon, 2-(2,5-difluorophenyl)-5-methylpyridineruthenium(II) tetrakis(acetonitrile) hexafluorophosphate (123.0 mg, 0.200 mmol), 2-(2,5-difluorophenyl)-5-methylpyridine (45.1 mg, 0.220 mmol, 1.10 equiv.), potassium carbonate (82.8 mg, 0.600 mmol, 3.00 equiv.), potassium acetate (14.0 mg, 0.100 mmol, 0.500 equiv.) and acetone (2.0 ml) were stirred in a sealed vial (crimp-capped) at 70 °C for 18 h. The reaction mixture was then cooled to room temperature and filtered (2.5 µm polytetrafluoroethylene filter). The solvent was concentrated in vacuo and pentane (20.0 ml) was slowly added whilst stirring until a solid precipitated. The precipitate was collected by filtration and washed with pentane to give bis[2-(2,5-difluorophenyl)-5-methylpyridine]ruthenium(II) bis(acetonitrile) (42.0 mg, 0.070 mmol, 35%) as an amorphous red solid. Note that complex **40** is air-sensitive and should be stored in a glove box under an atmosphere of purified argon.

## Supplementary information

Supplementary Information Supplementary Tables 1–17, Figs. 1–264 and Equation (1).

Supplementary Data 1 Crystallographic information file for compound **31** (CCDC 2384328).

Supplementary Data 2 Crystallographic information file for compound **36** (CCDC 2383351).

Supplementary Data 3 Crystallographic information file for compound **40** (CCDC 2384329).

## Data Availability

The data supporting the findings of this work are provided within the main text and the Supplementary Information. The crystallographic data for complexes **31**, **36** and **40** have been deposited at the Cambridge Crystallographic Data Centre (CCDC) under CCDC numbers 2384328 (**31**), 2383351 (**36**) and 2384329 (**40**). Copies of the data can be obtained free of charge via https://www.ccdc.cam.ac.uk/structures/. Data are available from the corresponding author upon request.
