# Supplementary material for: Precision installation of silyl synthetic handles within arenes by regiocontrolled ruthenium C(sp2)–H functionalization
Source: Nat Catal. 2025 Apr 2;8(4):301–14. doi: 10.1038/s41929-025-01309-6 (PMC12031671; doi:10.1038/s41929-025-01309-6)
Supplement: Supplementary file 1 — Supplementary Tables 1–17, Figs. 1–264 and Equation (1). [file 41929_2025_1309_MOESM1_ESM.pdf]

nature catalysis

Supplementary information

https://doi.org/10.1038/s41929-025-01309-6

Precision installation of silyl synthetic handles within arenes by regiocontrolled ruthenium C(sp^2)–H functionalization

In the format provided by the authors and unedited

Table of Contents

Supplementary Methods 2

General Experimental 2

Reaction optimisation 4

ortho-silylmethylation 4

meta-silylmethylation 10

Mechanistic investigations 19

Reaction progress monitoring and variable time normalisation analysis – *ortho*-silylmethylation 19

Reaction progress monitoring and variable time normalisation analysis – *meta*-silylmethylation 25

Stoichiometric reactivity of monocyclometallated ruthenium(II) complexes 33

Stoichiometric reactivity of biscyclometallated ruthenium(II) complexes 41

Reaction of biscyclometallated ruthenium complex with *gem*-bistrimethylsilyl chloromethane reagent – Monitored by ^{1}H NMR 47

Cyclic voltammetry (CV) of ruthenacycles 58

Procedures and characterisation for synthesised reagents and substrates 64

Synthesis of Losmapimod 83

Synthesis and characterisation of cyclometallated ruthenium(II) complexes 94

General procedures for ruthenium-catalysed *ortho*- and *meta*-silylmethylation 97

Reactivity in the absence of sodium iodide 98

Ruthenium-catalysed *ortho*-silylmethylation 100

Ruthenium-catalysed *meta*-silylmethylation 130

Product characterisation – *ortho*-silylmethylation product further reactivity 156

Product characterisation – *meta*-silylmethylation product further reactivity 163

NMR Spectra 170

X-ray crystallography data 388

Supplementary References 391

Supplementary Methods

General Experimental

Reaction Setup: All silylmethylation reactions were carried out in nitrogen-purged crimp-cap microwave vials (10 mL) and heated using aluminium pie-blocks. All air- and moisture sensitive reactions were carried out in a glovebox with a purified argon atmosphere. Glassware was cleaned using aqueous bleach solution (NaClO), water and acetone. Room temperature (*r.t.*) was approximately 18-20 °C. "Brine" refers to a saturated solution of sodium chloride in water.

Chemicals: All starting materials and solvents were purchased from Acros (Fisher), Aldrich (Merck), Alfa Aesar (Fisher), Fluorochem, Generon, Manchester Organics, Molekula, MP Biomedicals and TCI, and used without further purification unless otherwise stated.

Chromatography: Analytical thin-layer chromatography was performed on aluminium-backed silica plates (Merck 60 F_{254}). Product spots were visualised by UV light at 254 nm and subsequently developed using potassium permanganate solution if appropriate. Flash column chromatography was carried out using silica gel, particle size 40-63 μm, using standard techniques, or using an automated Biotage isolera.

Infrared Spectroscopy: IR spectra were recorded using a Thermo Scientific Nicolet iS5 FTIR machine, relevant bands are quoted in cm^{-1}.

NMR Spectroscopy: ^{1}H NMR, ^{19}F NMR and ^{13}C NMR spectra were recorded at 400 or 500 MHz on Bruker machines. ^{1}H NMR are referenced to the residual solvent peak at 7.26 ppm ($CDCl_3$), 2.05 ppm ($(CD_3)_2CO$), 2.50 ppm ($(CD_3)_2SO$), 1.94 ppm (CD_3CN), 3.31 ppm (CD_3OD) and quoted in ppm to 2 decimal places with coupling constants (J) to the nearest 0.1 Hz. ^{13}C NMR spectra, recorded at 101 MHz or 126 MHz, are referenced to the solvent peaks at 77.00 ppm ($CDCl_3$), 29.84 ppm ($(CD_3)_2CO$), 39.52 ppm ($(CD_3)_2SO$), 1.32 ppm (CD_3CN), 49.00 ppm (CD_3OD) and quoted in ppm to 1 decimal place with coupling constants (J) to the nearest 0.1 Hz. ^{19}F NMR spectra were recorded at 376 or 471 MHz in $CDCl_3$, $(CD_3)_2CO$, $(CD_3)_2SO$, CD_3CN, CD_3OD and quoted in ppm to 2 decimal places and with coupling constants (J) to the nearest 0.1 Hz. Multiplicities are indicated by app. (apparent), br. (broad), s (singlet), d (doublet), t (triplet), q (quartet), quin. (quintet), sext. (sextet), sept. (septet), non. (nonet). Coupling constants, J, are reported in Hertz and rounded to the nearest 0.1 Hz. ^{1}H and ^{13}C assignments are corroborated through 2-D NMR experiments (COSY, HSQC, HMBC).

Cyclic voltammetry (CV): Experiments were conducted using PalmSens4 potentiostat, controlled and processed using PSTrace 5 software. The working electrode was a GC disc (3 mm diameter, BASi part number MF-2012), the counter electrode was a Pt-wire

(BASi part number MW-4130) and an Ag/AgCl reference electrode was used (BASi part number – MF-2052). Cyclic voltammetry experiments were conducted inside an argon filled glovebox in a 10 mL glass vial. The final voltammogram of respective complexes and ligands were referenced relative to the $Fc^{+/0}$.

Mass Spectrometry:

High resolution mass spectra were performed by the School of Chemistry Mass Spectrometry Service (University of Manchester) using a Thermo Finnigan MAT95XP spectrometer. Mass spectra for the characterization of ruthenium complexes were performed by the School of Chemistry Mass Spectrometry Service (University of Manchester) employing a Waters SQD2 spectrometer.

Melting Points: Melting points (mp) were obtained in capillary tubes using a Stuart SMP11 apparatus and are uncorrected.

Reaction optimisation

The rows of the optimisation tables highlighted in light blue represent the optimal conditions which were used in the next stages of optimisation.

ortho-silylmethylation

Supplementary Table 1. Solvent selection for the ortho-selective C-H silylmethylation. Yields determined by ^{1}H NMR spectroscopy using 1,3,5-trimethoxybenzene as an internal standard.

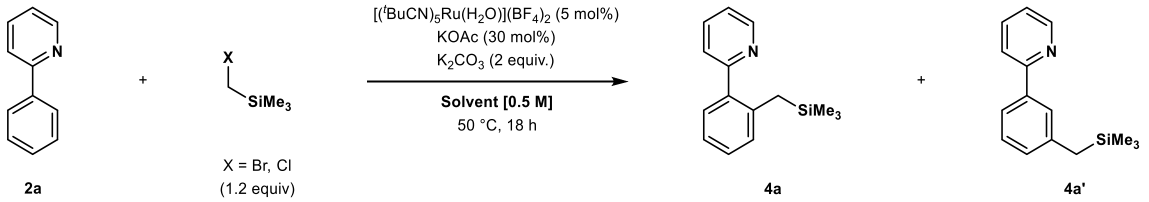

Entry	Solvent	2a (%)	4a (%)	4a' (%)
1	Dioxane (X = Br)	22	57	4
2	Dioxane (X = Cl)	88	4	0
3	^{i}PrOH/H_2O (3:2, X = Br)	50	22	18
4	^{i}PrOH/H_2O (3:2, X = Cl)	93	trace	0
5	THF (X = Br)	46	41	7
6	THF (X = Cl)	94	0	0
7	EtOAc (X = Br)	26	47	6
8	Bu_2O (X = Br)	38	41	9
9	$PhCF_3$ (X = Br)	9	55	*n.d*

Supplementary Table 2. Variation of base and carboxylate/phosphonate additive. Yields determined by ^{1}H NMR spectroscopy using 1,3,5-trimethoxybenzene as an internal standard.

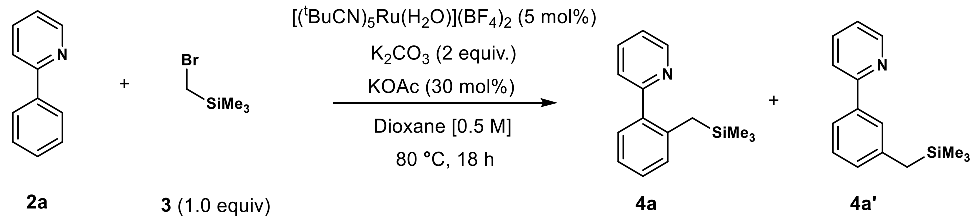

Entry	Variation	2a (%)	4a (%)	4a' (%)
1	No K_2CO_3	73	18	6
2	No KOAc	34	50	6
3	Li_2CO_3	64	17	4
4	Na_2CO_3	31	51	7
5	Cs_2CO_3	25	53	7
6	$KHCO_3$	30	53	10
7	K_3PO_4	29	50	7
8	$PhP(O)O_2Na_2$	30	52	9
9	$PhP(O)O_2K_2$	20	58	6

Supplementary Table 3. Equivalents of bromotrimethylsilyl methane. Yields determined by ^{1}H NMR spectroscopy using 1,3,5-trimethoxybenzene as an internal standard.

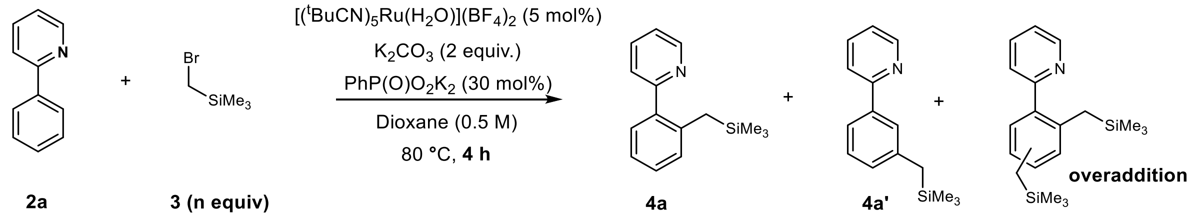

Entry	4a (n equiv.)	2a (%)	4a (%)	4a' (%)	Overaddition (%)
1	1.0	34	52	7	3
2	1.2	19	60	6	10
3	1.4	11	65	6	13
4	1.6	4	67	3	27

Supplementary Table 4. Effect of halide additive on yield and selectivity. Yields determined by ^1H NMR spectroscopy using 1,3,5-trimethoxybenzene as an internal standard.

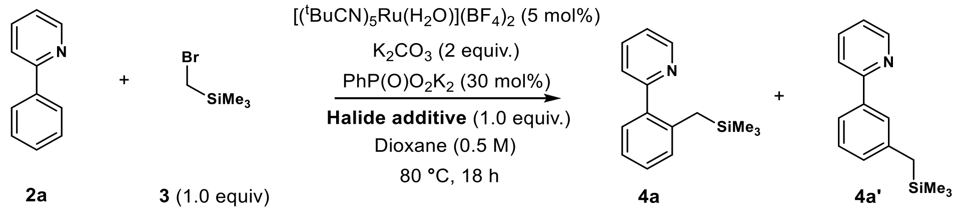

Entry	Halide additive	2a (%)	4a (%)	4a' (%)
1	NaI	14	90	Trace
2	LiI	20	73	2
3	TBAI	32	65	Trace
4	KI	23	69	3
5	KOTf	30	56	9
6	KBF_4	27	51	9
7	KBr	33	50	7
8	KCl	25	55	10

Supplementary Table 5. Effect of reaction temperature on selectivity and yield. Yields determined by ^1H NMR spectroscopy using 1,3,5-trimethoxybenzene as an internal standard. n.b reaction time = 4 hours.

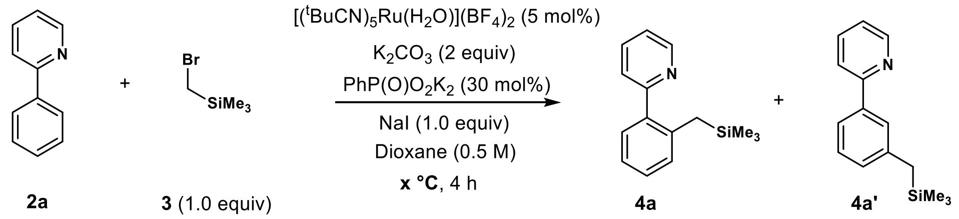

Entry	Temperature °C	2a (%)	4a (%)	4a' (%)
1	80	42	57	0
2	60	90	10	0
3	40	90	10	0

Supplementary Table 6. Solvent selection for the ortho-selective C-H silylmethylation in the presence of NaI. Yields determined by ^{1}H NMR spectroscopy using 1,3,5-trimethoxybenzene as an internal standard.

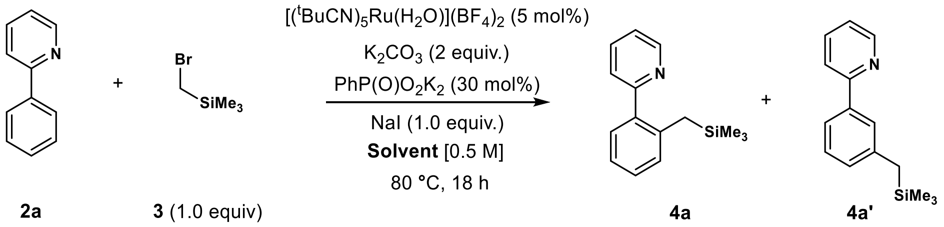

Entry	Solvent	2a (%)	4a (%)	4a’ (%)
1	Toluene	20	44	0
2	Dioxane	11	92	0
3	TBME	63	32	0
4	EtOAc	3	87	0
5	THF	4	93	0
6	Bu_2O	47	32	8

Supplementary Table 7. Variation in reaction conditions for the ortho-selective C-H silylmethylation in the presence of NaI. Yields determined by 1H NMR spectroscopy using 1,3,5-trimethoxybenzene as an internal standard. a2% overaddition product detected. b7% overaddition product detected.

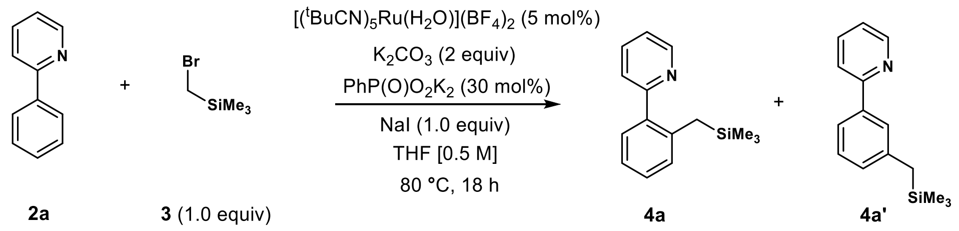

Entry	Variation	2a (%)	4a (%)	4a’ (%)
1	70 °C	20	75	0
2	K_2CO_3 (1.2 equiv.)	10	90	0
3	No $PhP(O)O_2K_2$	45	53	0
4	$[(C_6H_6)RuCl_2]_2$ (2.5 mol%)	80	17	0
5	[(p-cymene)$RuCl_2]_2$ (2.5 mol%)	85	14	0
6	NaI (0.5 equiv.)	13	88	0
7[a]	**4a** (1.25 equiv.)	5	86	0
8[b]	**4a** (1.50 equiv.)	6	88	0
9	none	4	93	0

Supplementary Table 8. Test silylmethylation using iodotrimethylsilyl methane. Yields determined by ^1H NMR spectroscopy using 1,3,5-trimethoxybenzene as an internal standard.

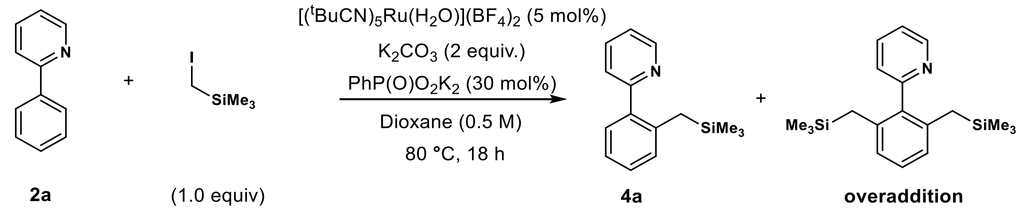

Entry	Variation	2a (%)	4a (%)	overaddition (%)
1	*none*	0	87	6
2	$I(CH_2)SiMe_3$ (3 equiv)	0	38	56
3	+ NaI (1.0 equiv.)	33	66	0

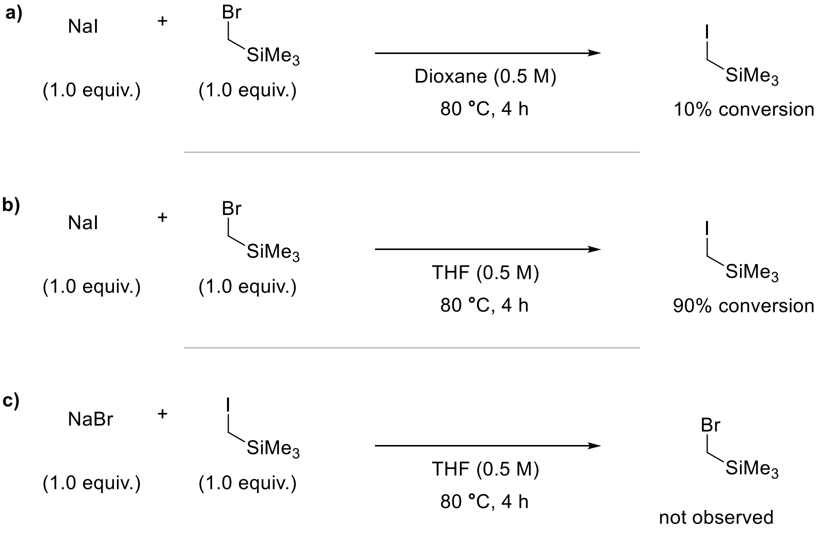

Supplementary Figure 1. Test Finkelstein reactions in both dioxane and THF, conversions determined by ^1H NMR spectroscopy.

***meta*-silylmethylation**

Supplementary Table 9. Solvent selection. Yields determined by ^{1}H NMR spectroscopy using 1,3,5-trimethoxybenzene as an internal standard. [a]Electrophile used = bromobis(trimethylsilyl)methane.

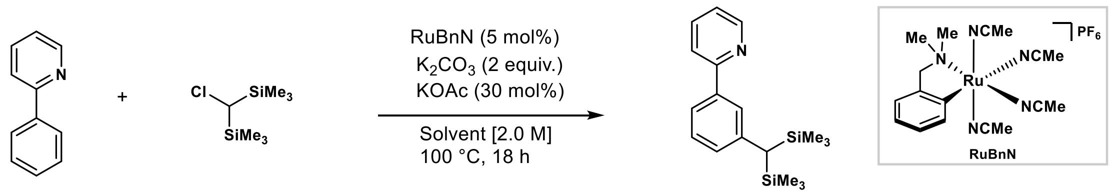

Entry	Solvent	2a (%)	6a (%)
1	*neat*	41	54
2	Water	40	46
3	Isoamyl alcohol	50	26
4	^{i}PrOH	66	36
5	Acetone	80	7
6	Cyclopentanone	>95	*trace*
7	Cyclohexanone	71	21
8	^{i}PrOAc	56	32
9	^{n}PrOAc	75	25
10[a]	^{i}PrOAc	84	8
11[a]	^{n}PrOAc	87	4
12	Dimethylcarbonate	75	30
13	Ethylene Glycol	>95	0
14	1-Octanol	70	18
15	Octane	63	32
16	Water [1 M]	30	23
17	Water [0.5 M]	56	21
18	Water [0.25 M]	30	24
19	^{i}PrOH:Water [9:1, 1 M]	55	46
20	^{i}PrOH:Water [1:1, 1 M]	34	60
21	^{i}PrOH:Water [1:1, 2 M]	45	38

Supplementary Table 10. Additive selection. Yields determined by ^{1}H NMR spectroscopy using 1,3,5-trimethoxybenzene as an internal standard. [a]Using Li_2CO_3 (2 equiv.).

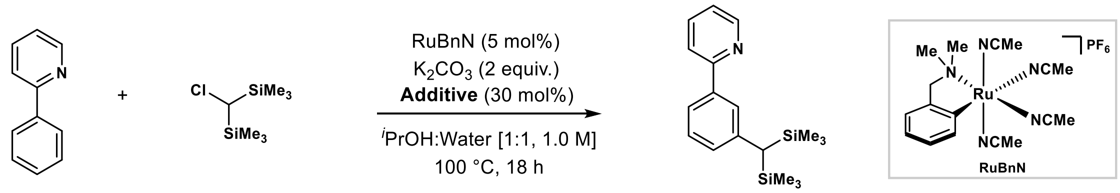

2a **5** (1.2 equiv.) **6a**

Entry	Additive	2a (%)	6a (%)
1	*none*	32	44
2	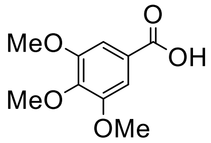	45	45
3	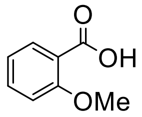	50	50
4	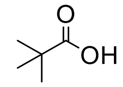	40	62
5	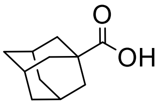	58	41
6	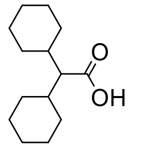	45	60
7	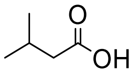	29	70
8	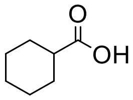	25	74
9	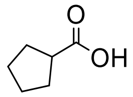	10	84
10	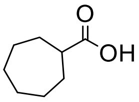	10	90
11	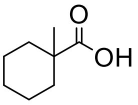	8	93
12	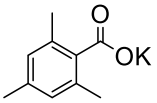	24	71
13[a]	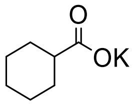	4	94

14[a]	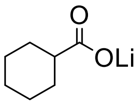	5	95

Supplementary Table 11. Base selection. Yields determined by ^{1}H NMR spectroscopy using 1,3,5-trimethoxybenzene as an internal standard. [a]Reaction time = 2 hours.

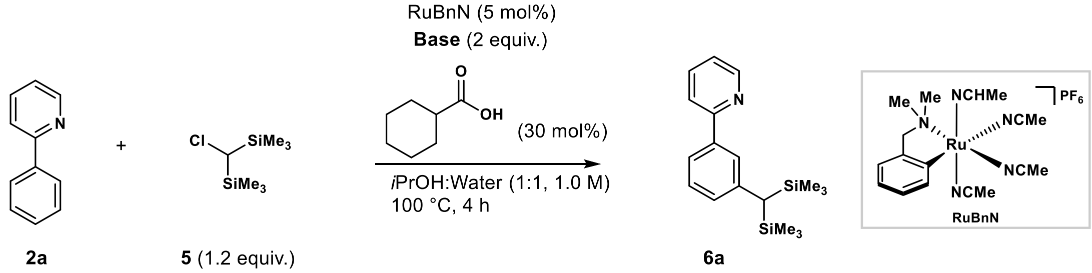

Entry	Base	2a (%)	6a (%)
1	K_3PO_4	30	58
2	Li_2CO_3	20	78
3	Na_2CO_3	20	69
4	NEt_3	68	19
5	KOMe	>95	0
6	NaO^{t}Bu	>95	0
7[a]	Li_2CO_3 (1.0 equiv.)	14	85
8[a]	Li_2CO_3 (1.5 equiv.)	17	81
9[a]	Li_2CO_3 (3.0 equiv.)	15	85

Supplementary Table 12. Pre-catalyst selection. Yields determined by ^{1}H NMR spectroscopy using 1,3,5-trimethoxybenzene as an internal standard. [a]Using $CyCO_2Li$.

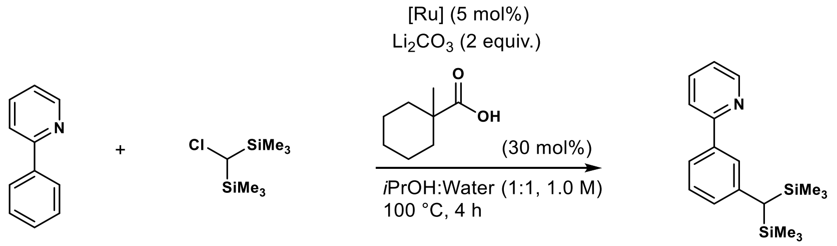

2a **5** (1.2 equiv.) **6a**

Entry	[Ru]	2a (%)	6a (%)
1	$[(C_6H_6)RuCl_2]_2$	20	72
2	$[CpRu(p\text{-cymene})]PF_6$	>95	0
3	$[(p\text{-cymene})RuCl_2]_2$	14	69
4	$[(C_6Me_6)RuCl_2]_2$	>95	0
5	$(COD)Ru(2\text{-Me-allyl})_2$	80	18
6	$[(^{t}BuCN)_5Ru(H_2O)](BF_4)_2$	12	85
7[a]	$[(^{t}BuCN)_5Ru(H_2O)](BF_4)_2$	8	92
8	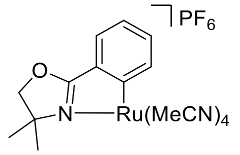	75	25

Supplementary Table 13. ^{i}PrOH/water co-solvent ratio optimisation. Yields determined by ^{1}H NMR spectroscopy using 1,3,5-trimethoxybenzene as an internal standard. *n.b* reaction time = 0.5 hours.

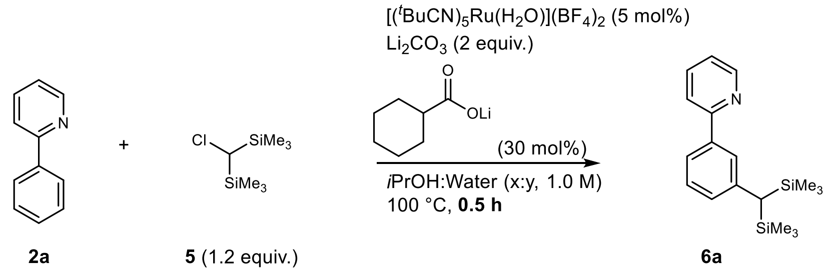

Entry	^{i}PrOH:Water	2a (%)	6a (%)
1	4:1	75	33
2	1:4	50	54
3	2:3	43	56
4	3:2	35	62

Supplementary Table 14. Carboxylate loading variation. Yields determined by ^{1}H NMR spectroscopy using 1,3,5-trimethoxybenzene as an internal standard. ^{a}Using $CyCO_2Li$. *n.b* reaction time = 2 hours.

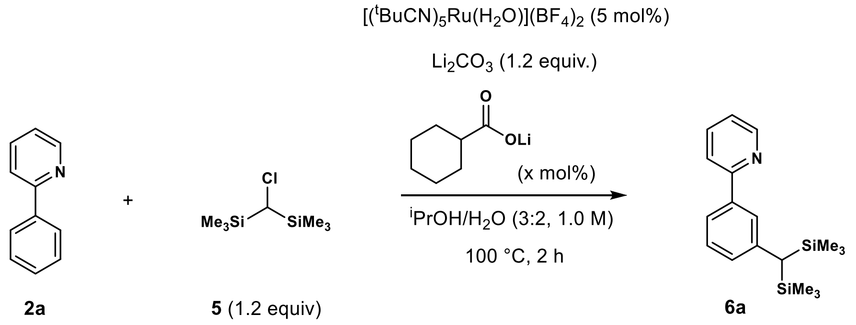

Entry	Carboxylate Loading (mol%)	2a (%)	6a (%)
1	100	15	86
2	50	15	83
3	10	18	81
4	5	18	81
5	2.5	33	70
6	0	30	75

Supplementary Table 15. Variation in reaction conditions – Temperature and [Ru] loading. Yields determined by ^{1}H NMR spectroscopy using 1,3,5-trimethoxybenzene as an internal standard.

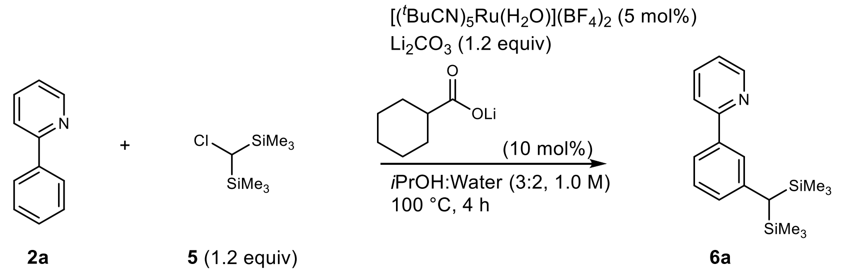

Entry	Variation	2a (%)	6a (%)
1	none	8	93
2	85 °C	22	80
3	70 °C	20	80
4	[Ru] (2.5 mol%)	61	41

Supplementary Table 16. Robustness screen for the ruthenium-catalysed *meta*-silylmethylation of 2-phenylpyridine. Reaction conditions: [Ru] (5 mol%), Li_2CO_3 (1.2 equiv.), CyCOOLi (10 mol%), $^{i}PrOH/H_2O$ (3:2, 1.0 M), arene (1.0 equiv.), chlorobis(trimethylsilyl)methane (1.2 equiv.) and an additive (1.0 equiv.) reacted at 100 °C for 4 hours. Yields and recoveries determined by ^{1}H NMR spectroscopy using 1,3,5-trimethoxybenzene as an internal standard.

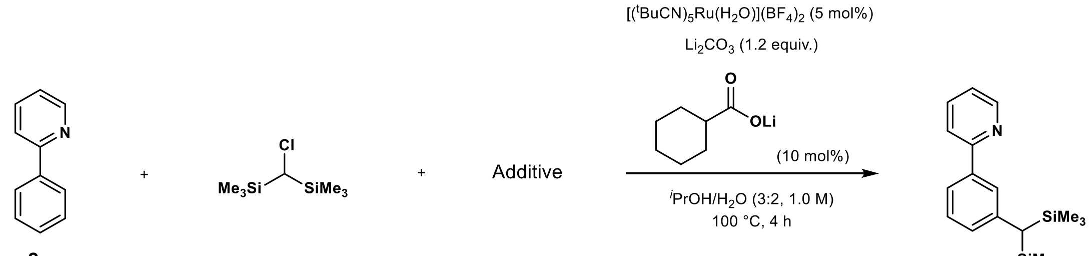

2a · 5 (1.2 equiv.) · (1.0 equiv.) · 6a

Entry	Additive (1 equiv)	Yield 6a (%)	Additive remaining (%)	2a remaining (%)
1	*none*	93	-	-
2	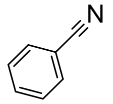	31	84	51
3	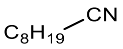	20	87	64
4	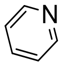	0	>95	>95
5	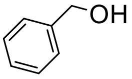	18	80	70
6	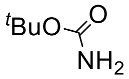	79	90	17
7	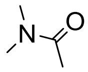	92	>95	10
8	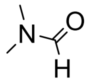	75	85	23

9	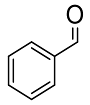	0	✗	>95%	✓	>95%
10	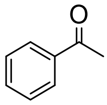	42	–	87	✓	55
11	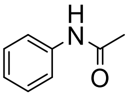	88	✓	>95%	✓	7
12	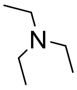	86	✓	>95%	✓	15
13	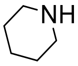	0	✗	86	✓	>95%
14	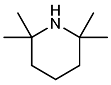	66	✓	82	✓	27
15	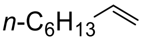	21	–	71	–	71
16	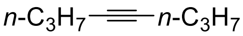	0	✗	84	✓	>95
17	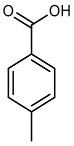	70	✓	33	✗	26
18	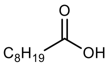	84	✓	Trace	✗	22
19	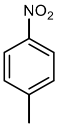	6	✗	95	✓	70
20	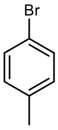	<5	✗	0	✗	0

21	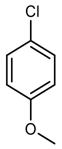	50		58		<5
22	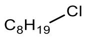	75		86		16
23	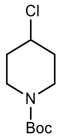	53		>95		28
24	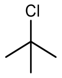	62		<5		27

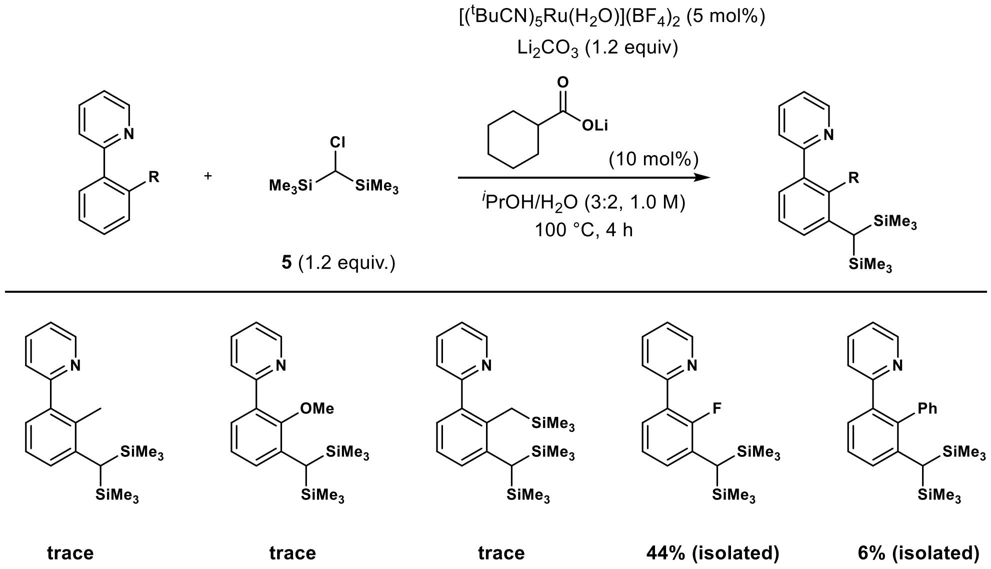

Supplementary Figure 2. Exploration of the tolerance of *meta*-selective ruthenium C-H silylmethylation to *ortho* substitution of 2-phenylpyridine.

Mechanistic investigations

Reaction progress monitoring and variable time normalisation analysis – *ortho*-silylmethylation

General Procedure for kinetic data

Stock solutions of 2-[3-(trifluoromethyl)phenyl]pyridine **2k** [3.0 M] and (bromomethyl)trimethylsilane **3** [3.5 M] were prepared in 1,4-dioxane. $[(^tBuCN)_5Ru(H_2O)](BF_4)_2$ **1** (26.0 mg, 38.0 μmol, 5 mol%), sodium iodide (112.4 mg, 0.750 mmol, 1.0 equiv), potassium carbonate (207.0 mg, 1.500 mmol, 2.0 equiv.) and potassium phenyl phosphonate (53.8 mg, 0.230 mmol, 30 mol%) were added to a 10 mL microwave vial. In a glovebox with a purified argon atmosphere, biphenyl (500 μL, 0.15 M, 1,4-dioxane) was added as a solution in 1,4-dioxane. Degassed 1,4-dioxane was then added such that the final total volume would be 1.50 mL. The vial was capped with a septum and heated at 80 ˚C for 20 min with a stirring rate of 500 rpm before the addition of the 2-[3-(trifluoromethyl)phenyl]pyridine **2k** and (bromomethyl)trimethylsilane **3** solutions simultaneously by microsyringe at t = 0 min to start the reaction. Aliquots of approximately 50 μL were then taken throughout the first 7 h of the reaction at specified time points. Each aliquot was added to approximately 1 mL of a solution of 10% pyridine in Et_2O (v/v), before being passed through a short plug of silica, using Et_2O as eluent into a GC vial. The reaction was then monitored by GC-FID.

Determination of Order in Catalyst

Order in catalyst was determined using variable-time normalisation analysis (VTNA). Reactions were carried out with different concentrations of catalyst and their temporal profiles were normalized according to the catalyst loading raised to the power of the order in the catalyst. Resulting data were plotted together and the order in catalyst determined by visual overlay.

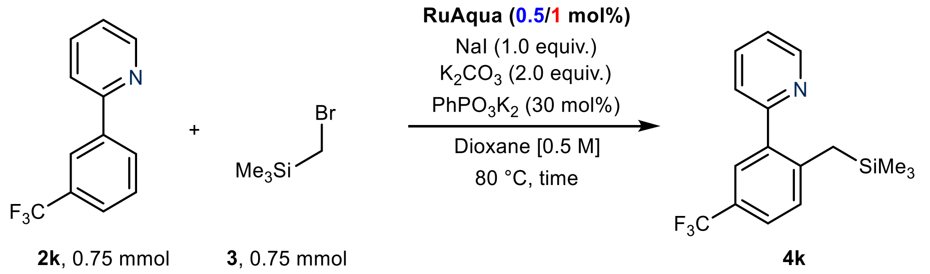

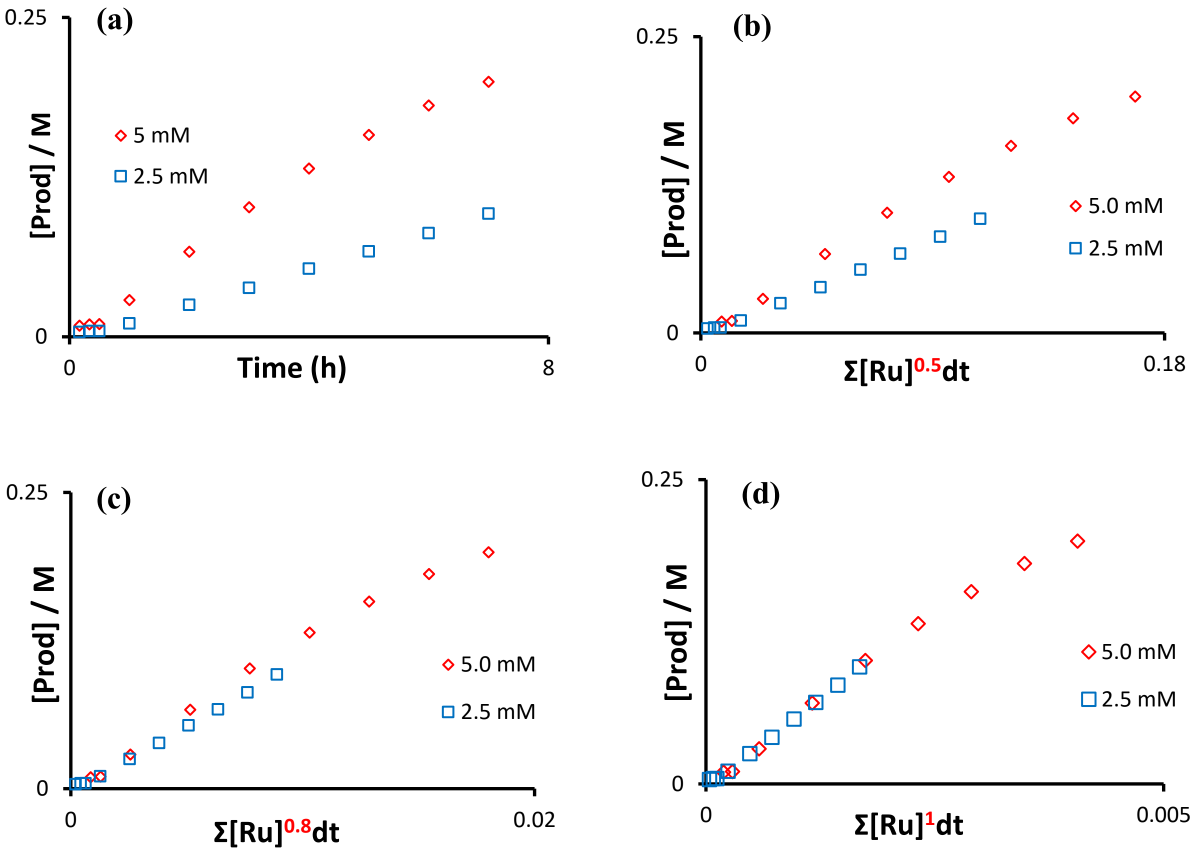

Supplementary Figure 3. (a) Temporal reaction profiles of reactions carried out with 0.5/1 mol % of [Ru]; **(b)** Normalised time scale profiles for order 0.5 in [Ru]; (c) Normalized time scale profiles for order 0.8 in [RuAqua]; (d) Normalised time scale pofiles for order 1.0 in [Ru].

The overlap between normalised time scale reaction profiles for these two reactions with differing starting concentrations of [Ru] shows an order of 1.0 at these concentrations.

Determination of Order in Starting Material

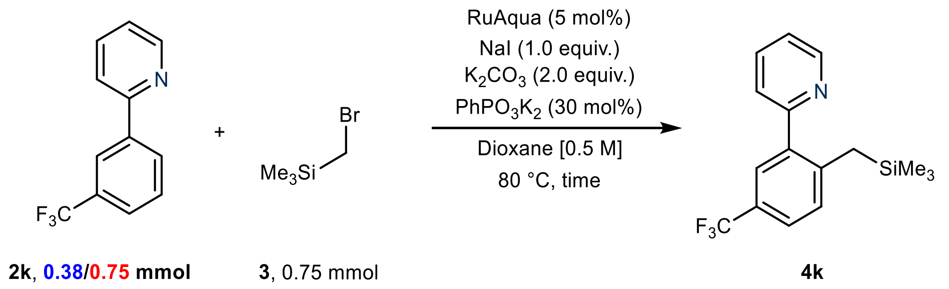

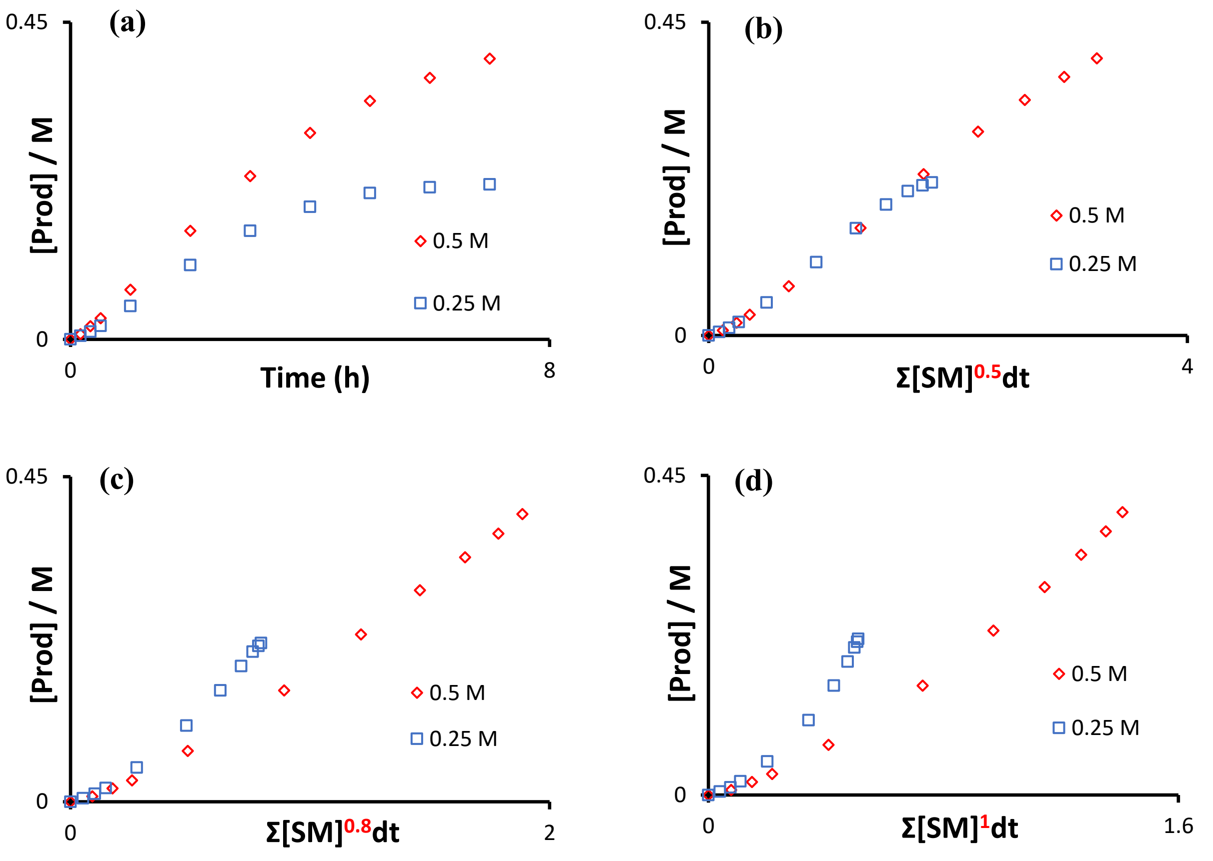

Supplementary Figure 4. (a) Temporal reaction profiles of reactions carried out with 0.25/0.50 M of [X]; **(b)** Normalised time scale profiles for order 0.5 in [X]; (c) Normalised time scale profiles for order 0.8 in [X]; (d) Normalised time scale profiles for order 1.0 in [X].

The overlap between normalised time scale reaction profiles for these two reactions with differing starting concentrations of [SM] shows an order of 0.5 at these concentrations.

Order in Electrophile

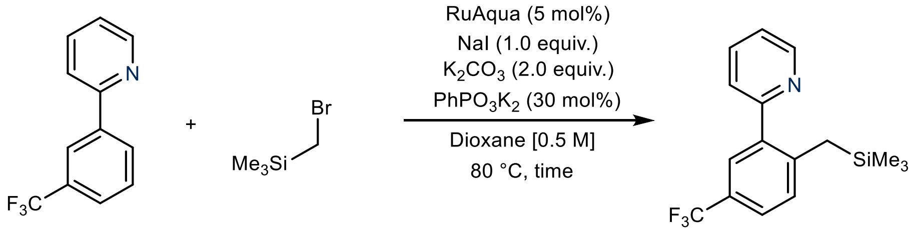

2k, 0.75 mmol **3**, **0.38**/**0.75 mmol** **4k**

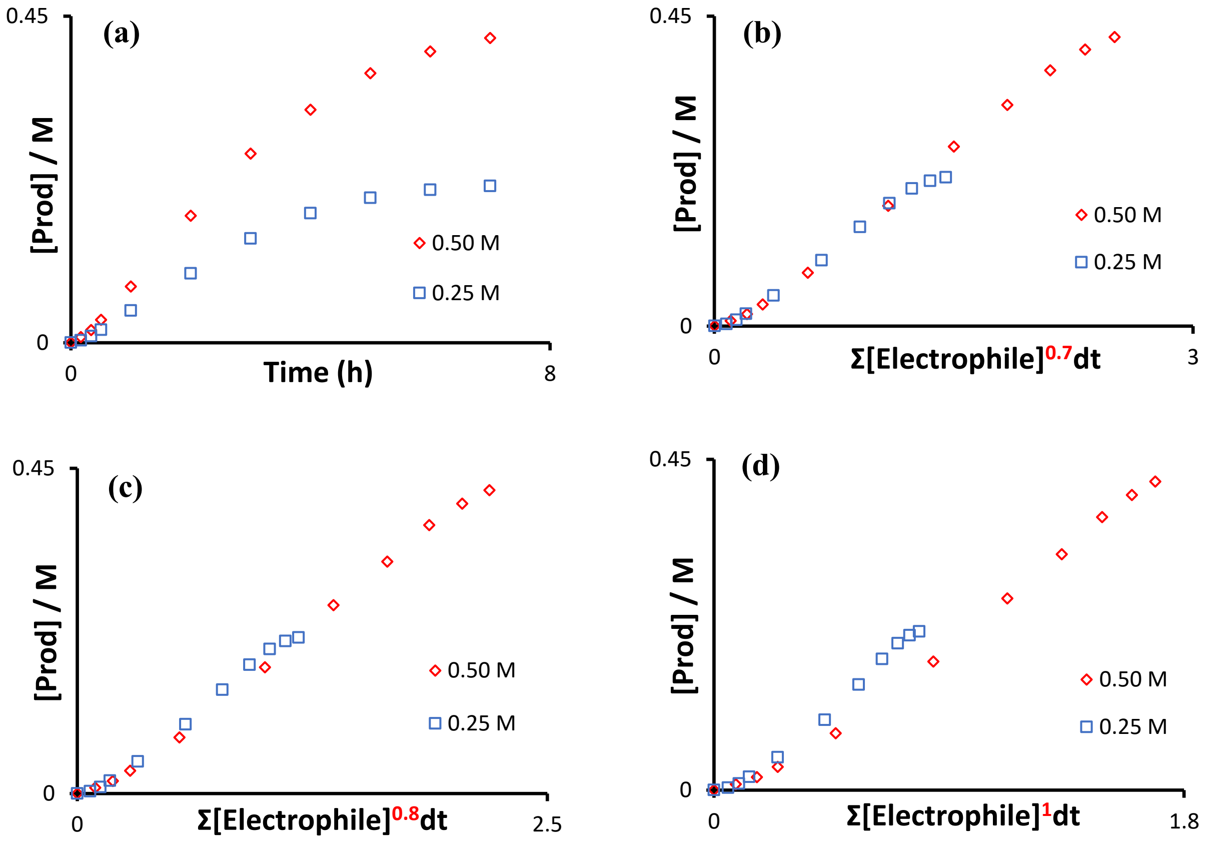

Supplementary Figure 5. (a) Temporal reaction profiles of reactions carried out with 0.25/0.50 M of [X]; **(b)** Normalised time scale profiles for order 0.7 in [X]; (c) Normalised time scale profiles for order 0.8 in [X]; (d) Normalized time scale profiles for order 1 in [X].

The overlap between normalised time scale reaction profiles for these two reactions with differing starting concentrations of [Elect] shows an order of 0.7 at these concentrations.

Order in Sodium Iodide

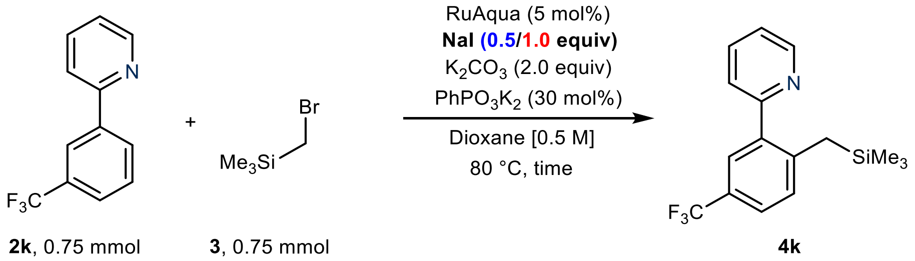

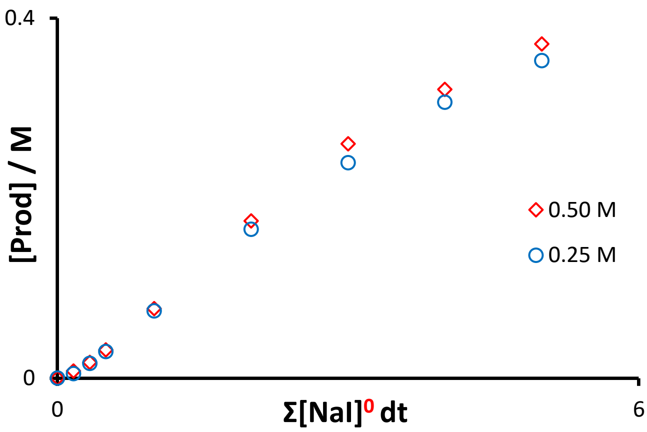

Supplementary Figure 6. Temporal reaction profile carried out with 1/0.5 equivalents of sodium iodide.

The overlap between normalised time scale reaction profiles for these two reactions with differing starting concentrations of [NaI] shows an order of 0 at these concentrations.

Order in Base

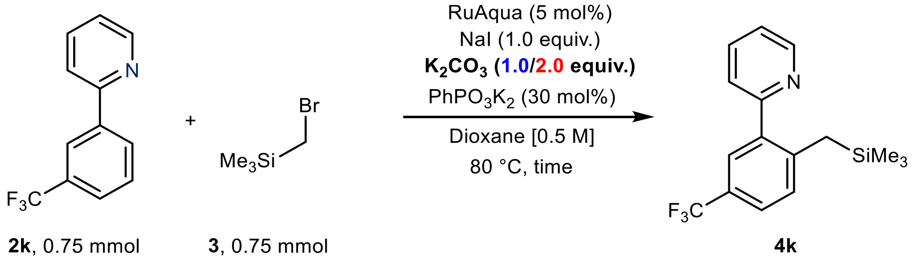

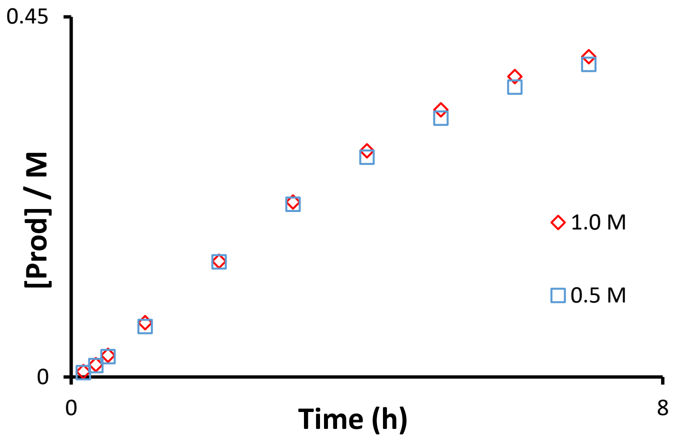

Supplementary Figure 7. Temporal reaction profile carried out with 1/2 equivalents of potassium carbonate.

The overlap between normalised time scale reaction profiles for these two reactions with differing starting concentrations of [K_2CO_3] shows an order of 0 at these concentrations.

Reaction progress monitoring and variable time normalisation analysis – *meta*-silylmethylation

Stock solutions of 2-phenylpyridine **2a** [5.0 M] and chlorobis(trimethylsilyl)methane **5** [4.0 M] were prepared in isopropanol, and $[(^tBuCN)_5Ru(H_2O)](BF_4)_2$ in isopropanol/water (3:2, 0.20 M). Biphenyl (0.75 mL, 0.1 M, ^{i}PrOH) was added as an internal standard to a 10 mL microwave vial and the solvent removed *in vacuo* (500 bar to 50 mbar, 40 °C). Lithium carbonate (66 mg, 0.9 mmol, 1.2 equiv.) and lithium cyclohexane carboxylate (10 mg, 0.076 mmol, 10 mol%) were then added. In a glovebox with a purified argon atmosphere, **2a** and **5** were added to the 10 mL vial using a microsyringe. Degassed isopropanol and water were added to the vial to a total volume of 1 mL while maintaining the 3:2 co-solvent ratio. The vial was capped with a septum and heated at 70 ˚C for 20 min with a stirring rate of 500 rpm before addition of catalyst solution was added using a microsyringe at t = 0 min to start the reaction. Aliquots of approximately 50 µL were then taken throughout the first 4 h of the reaction at specified time points. Each aliquot was added to approximately 1 mL of a solution of 10% pyridine in Et_2O (v/v), before being passed through a short plug of silica, using Et_2O as eluent into a GC vial. The reaction was then monitored by GC-FID.

Order in catalyst

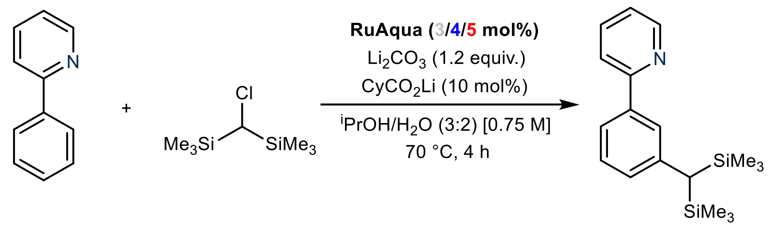

2a, 0.75 mmol **5**, 0.90 mmol **6a**

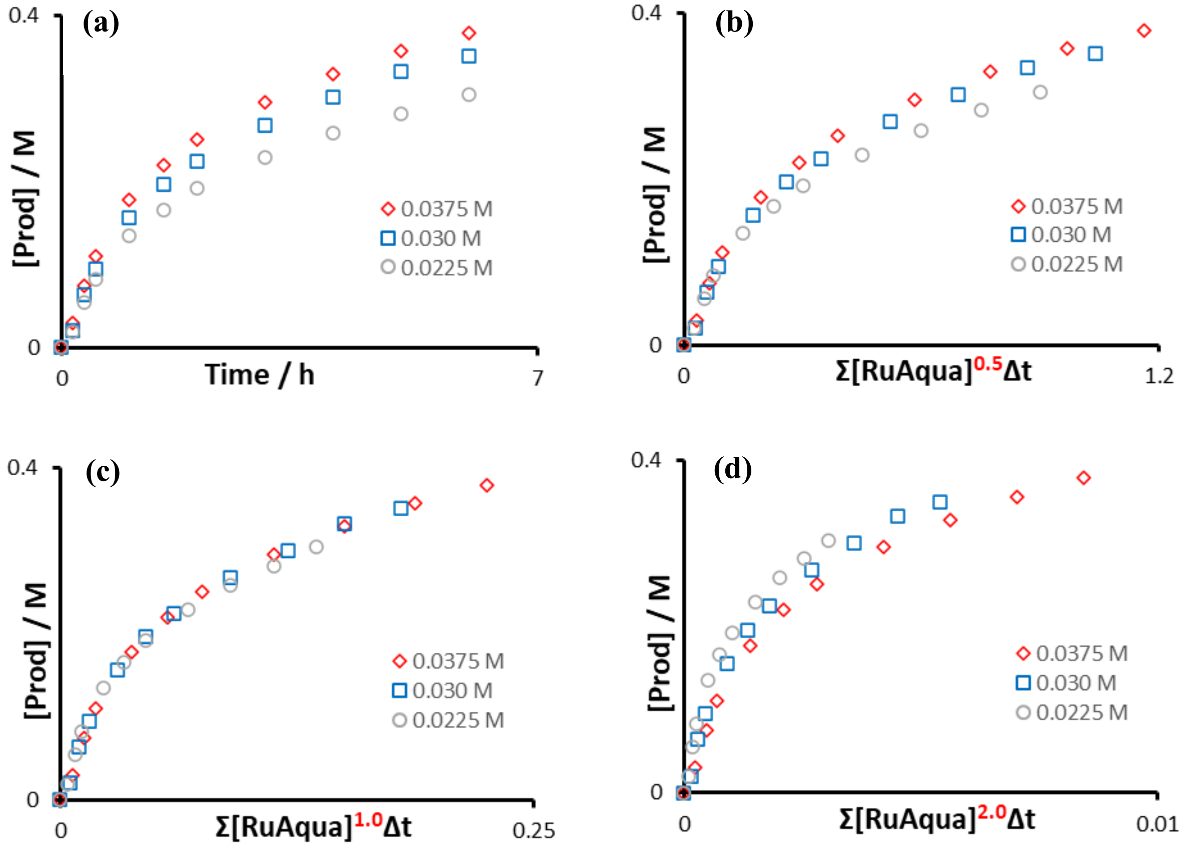

Supplementary Figure 8. (a) Temporal reaction profiles of reactions carried out with 3/4/5 mol % of [RuAqua]; **(b)** Normalized time scale profiles for order 0.5 in [RuAqua]; (c) Normalized time scale profiles for order 1.0 in [RuAqua]; (d) Normalized time scale profiles for order 2.0 in [RuAqua].

The overlap between normalised time scale reaction profiles for these three reactions with differing starting concentrations of [RuAqua] shows an order of 1.0 at these concentrations.

Order in SM

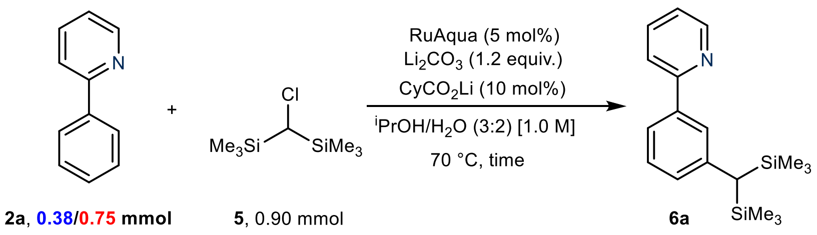

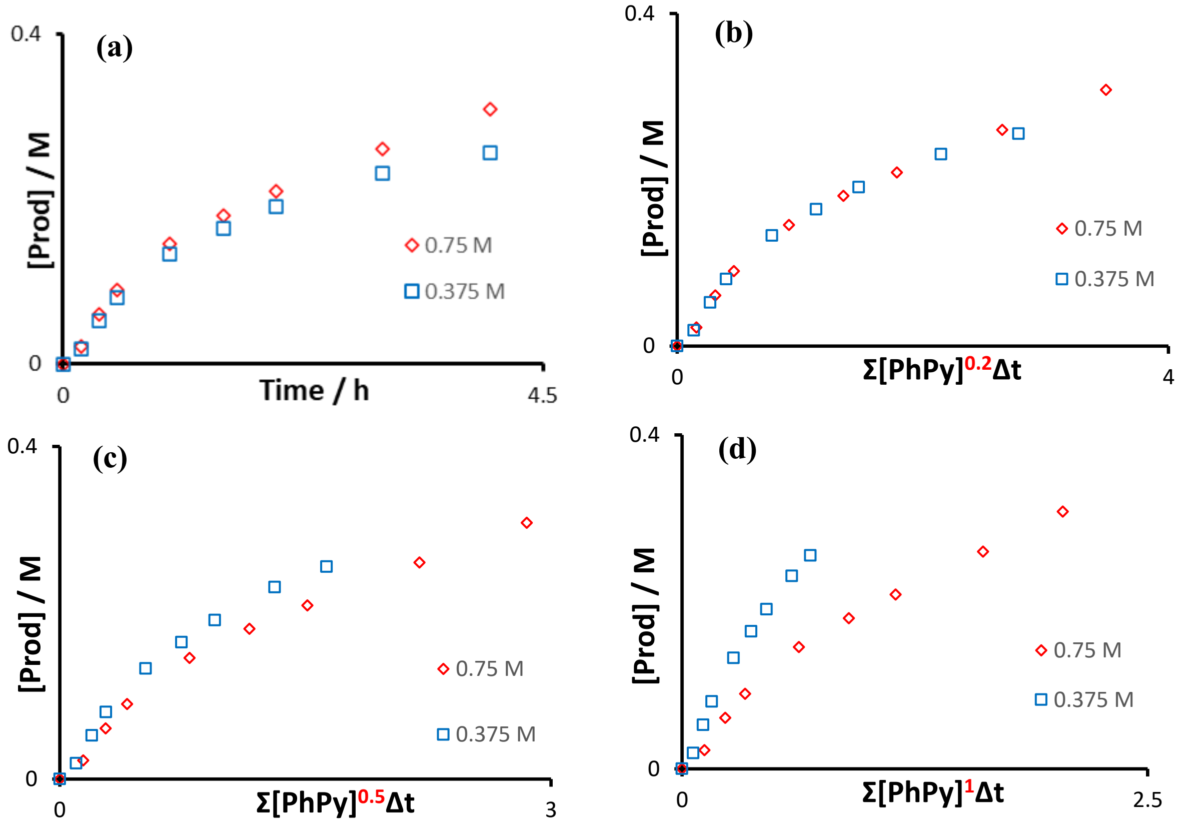

Supplementary Figure 9. (a) Temporal reaction profiles of reactions carried out with 0.38/0.75 M of [SM]; **(b)** Normalised time scale profiles for order 0.2 in [SM]; (c) Normalised time scale profiles for order 0.5 in [SM]; **(d)** Normalised time scale profiles for order 1.0 in [SM].

The overlap between normalised time scale reaction profiles for these two reactions with differing starting concentrations of [SM] shows an order of 0.2 at these concentrations.

Order in Electrophile

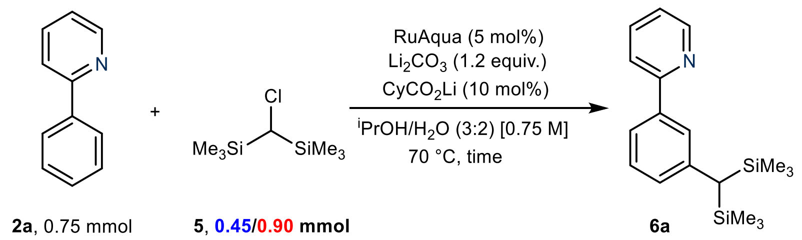

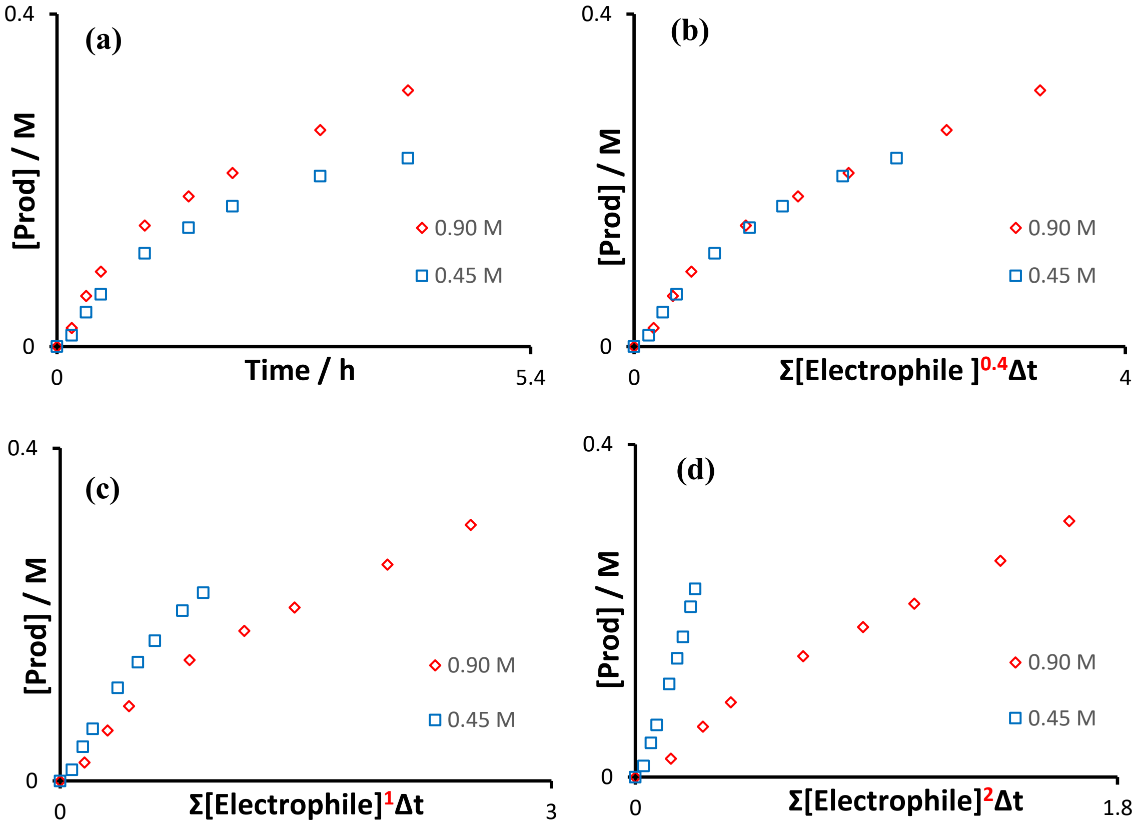

Supplementary Figure 10. (a) Temporal reaction profiles of reactions carried out with 0.45/0.90 M of [X]; **(b)** Normalised time scale profiles for order 0.4 in [X]; **(c)** Normalised time scale profiles for order 1 in [X]; **(d)** Normalised time scale profiles for order 2.0 in [X].

The overlap between normalised time scale reaction profiles for these two reactions with differing starting concentrations of [SM] shows an order of 0.4 at these concentrations.

Order in $CyCO_2Li$

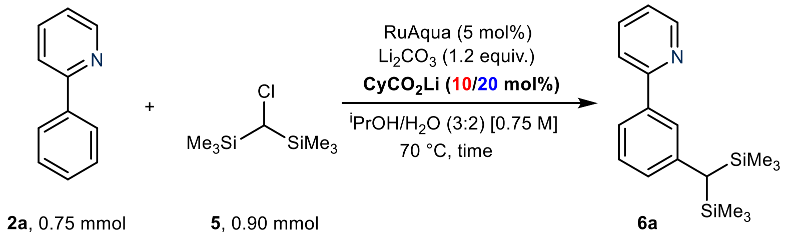

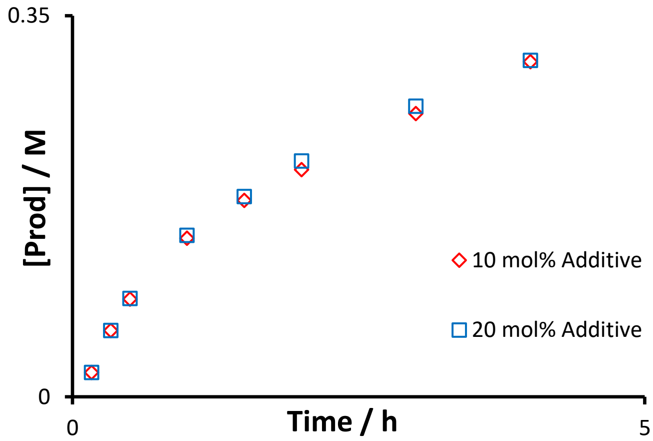

Supplementary Figure 11. Temporal reaction profile carried out with 10/20 mol% of lithium cyclohexane carboxylate.

The overlap between normalised time scale reaction profiles for these two reactions with differing starting concentrations of additive shows an order of 0 at these concentrations.

Order in Base

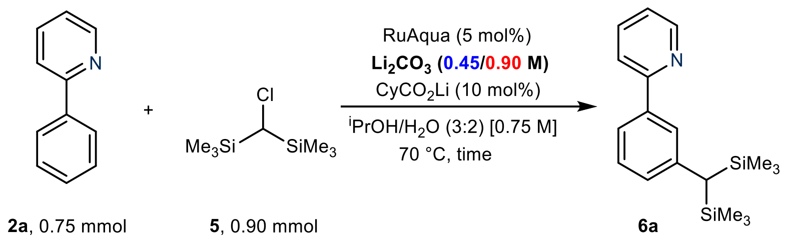

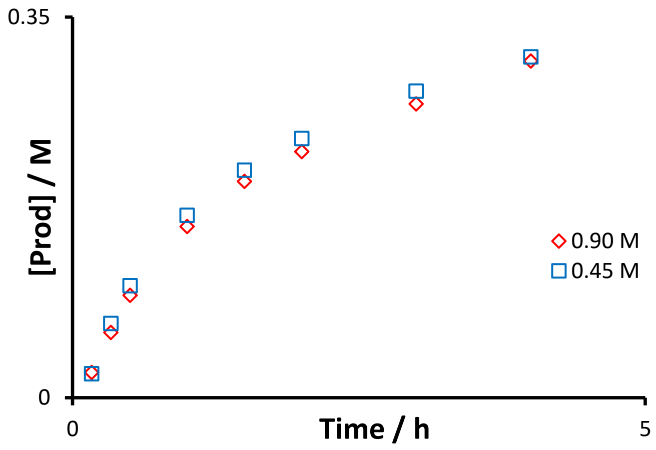

Supplementary Figure 12. Temporal reaction profile carried out with 0.45/0.90 M of lithium carbonate.

The overlap between normalised time scale reaction profiles for these two reactions with differing starting concentrations of base shows an order of 0 at these concentrations.

Order in organic product

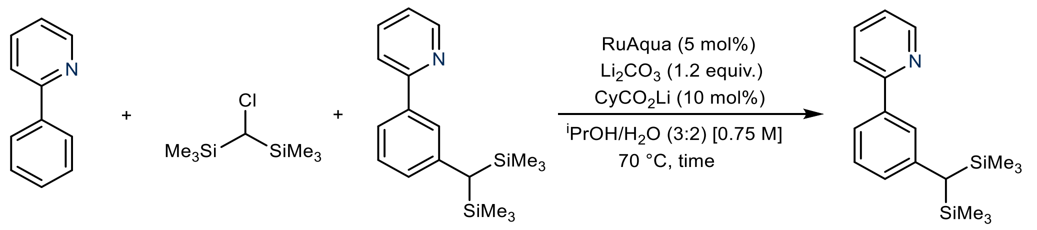

2a, 0.75 mmol **5**, 0.90 mmol **6a**, **0.0/0.10 mmol** **6a**

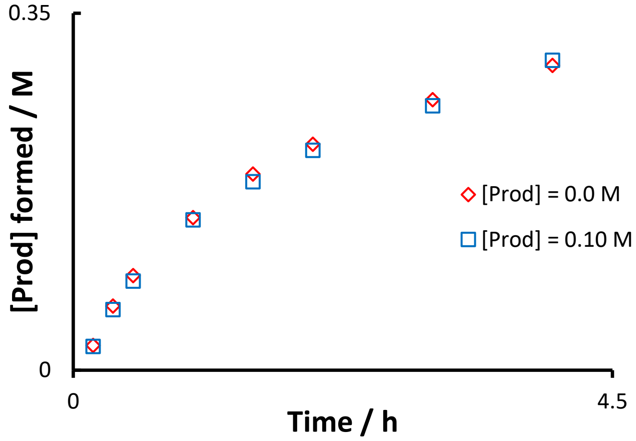

Supplementary Figure 13. Temporal reaction profiles of reactions carried out with 0.00/0.10 M of [Prod].

The overlap between normalised time scale reaction profiles for these two reactions with differing starting concentrations of the organic product shows an order of 0 at these concentrations suggesting there is not product inhibition from the organic product.

Order in LiCl

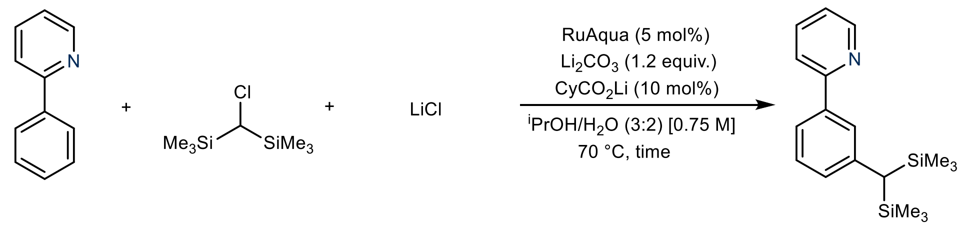

2a, 0.75 mmol   **5**, 0.90 mmol   **0.0/0.25 mmol**   **6a**

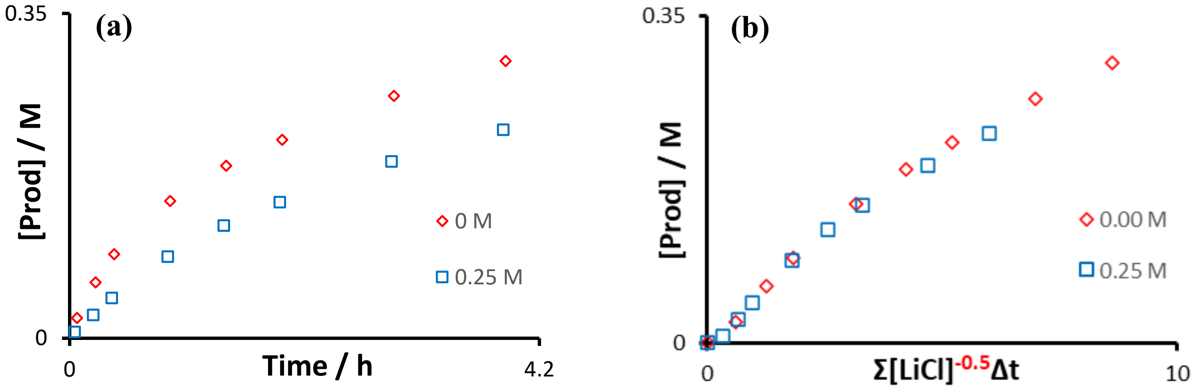

Supplementary Figure 14. a) Temporal reaction profiles of reactions carried out with 0.00/0.25 M of [LiCl]; **(b)** Normalised time scale profiles for order -0.5 in [LiCl].

The overlap between normalised time scale reaction profiles for these two reactions with differing starting concentrations of lithium chloride shows an order of -0.5 at these concentrations.

Stoichiometric reactivity of monocyclometallated ruthenium(II) complexes

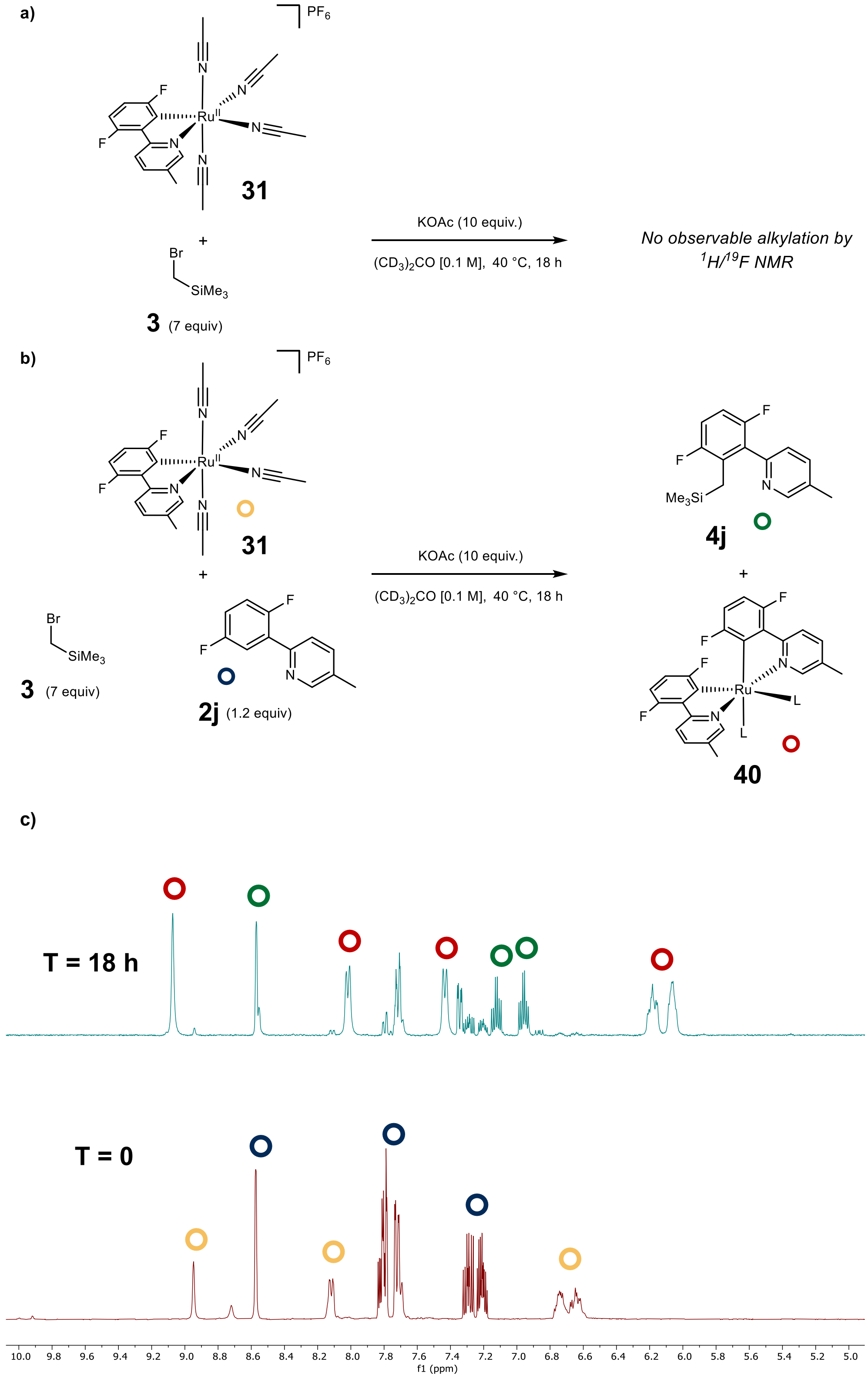

Supplementary Figure 15. Stoichiometric reactivity of monocyclometallated species **31**. **a)** without additional arene. **b)** with additional equivalent of arene 2j **c)** ^{1}H NMR Spectra [$(CD_3)_2CO$, 400 MHz] at the start (T = 0) and after (T = 18 h) the stoichiometric reaction of **31** (○) with **2j** (○) producing **4j** (○) and **40** (○).

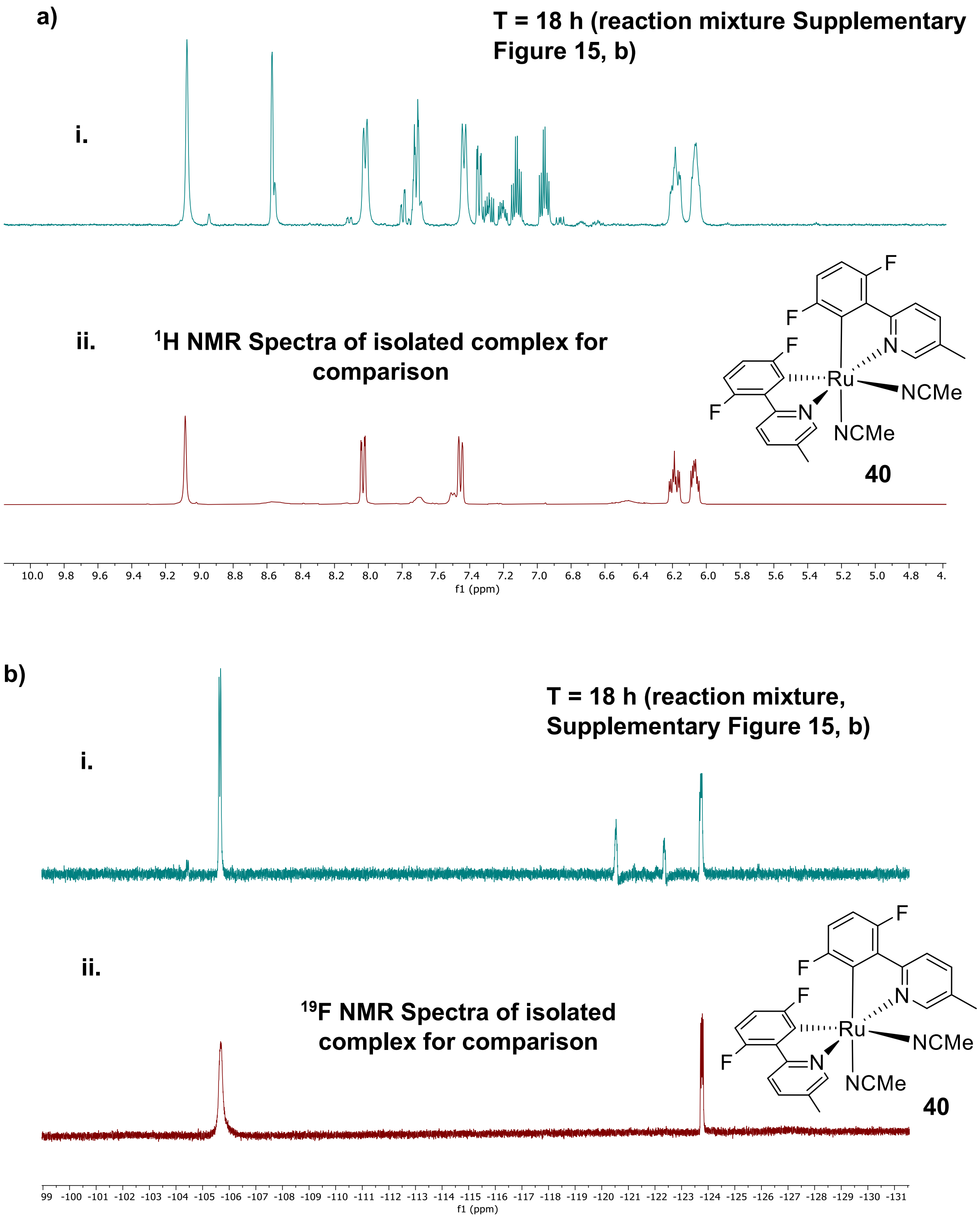

Supplementary Figure 16. a) ^{1}H and **b)** ^{19}F NMR spectra [$(CD_3)_2CO$, 400 MHz, 376 MHz] comparison of reaction mixture containing monocyclometallated species 31, additional arene 2j and (bromomethane)trimethylsilane 3 after 18 hours **i.** and isolated bis[2-(2,5-difluorophenyl)-5-methylpyridine] ruthenium(II) bisacetonitrile **40 ii.**

a)

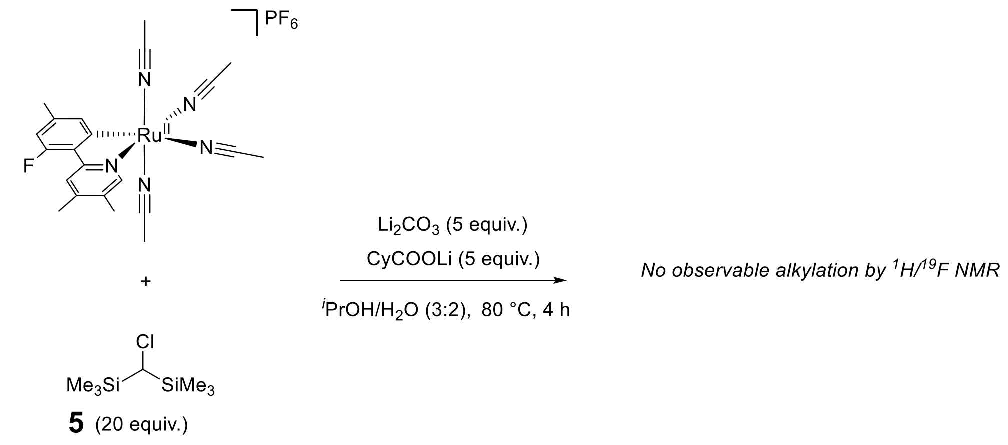

b)

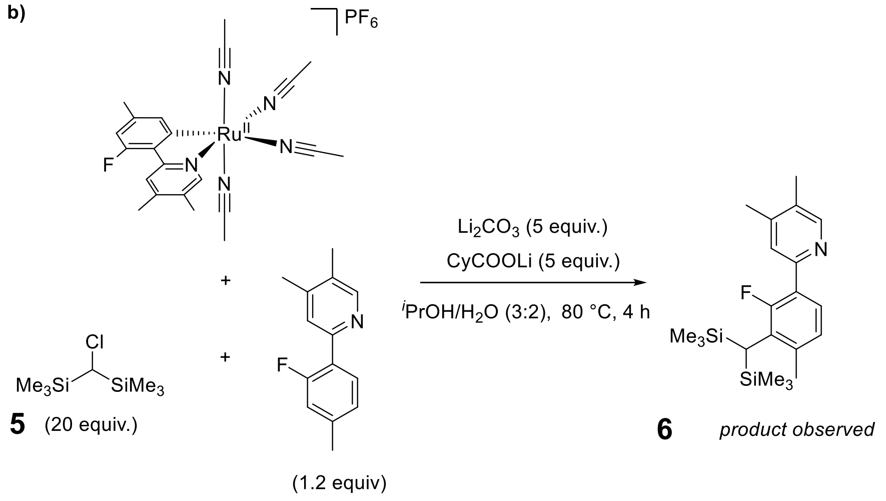

Supplementary Figure 17. Reaction of stoichiometric mono-cyclometallated ruthenium complex using chlorobistrimethylsilyl methane **5. a)** Reaction of excess halide electrophile with mono-cyclometallated ruthenium complex gave no alkylated arene. **b)** Repeat reaction with added phenylpyridine derivative (1.2 equiv.) showed product formation **6m.**

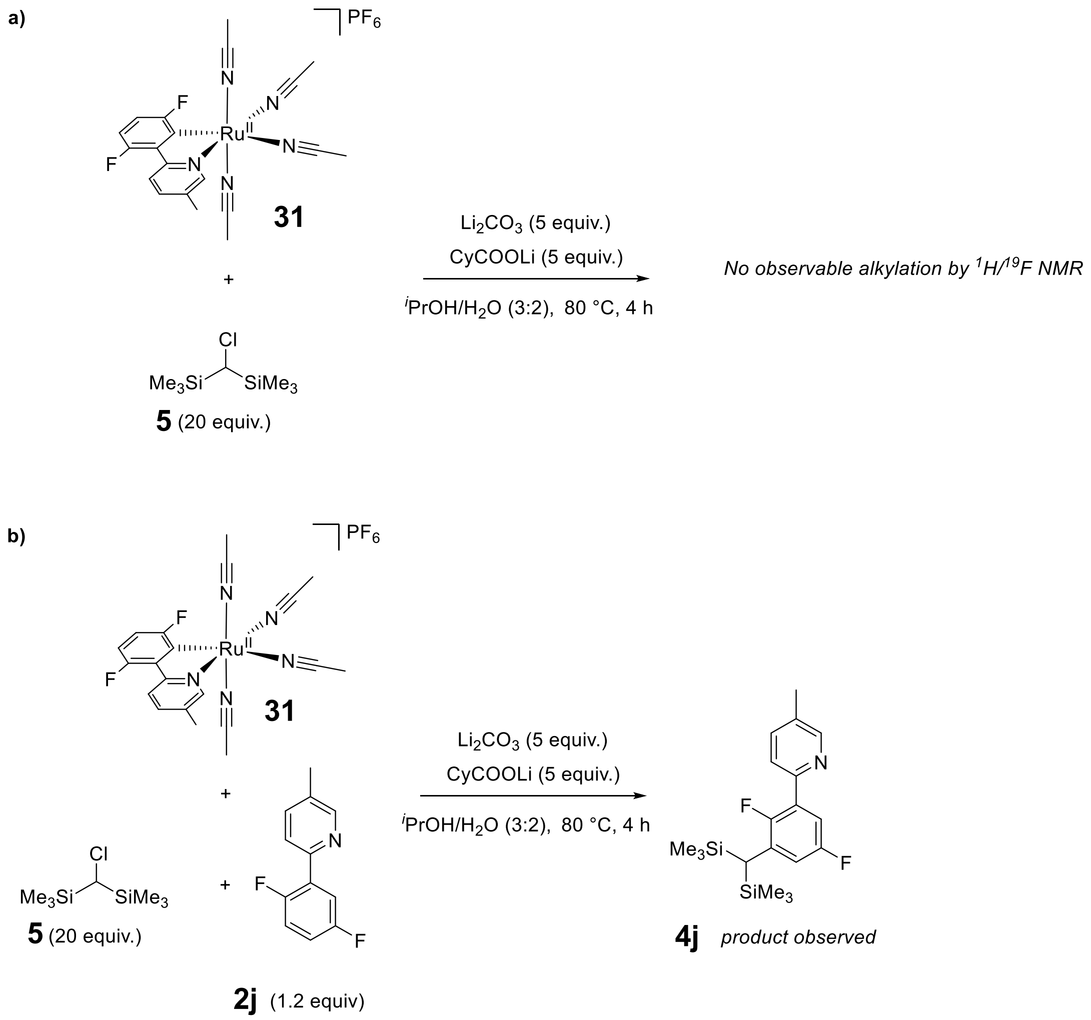

Supplementary Figure 18. Reaction of stoichiometric mono-cyclometallated ruthenium complex **31** using chlorobistrimethylsilyl methane **5**. **a)** Reaction of excess halide electrophile **5** with mono-cyclometallated ruthenium complex **31** gave no alkylated arene. **b)** Repeat reaction with added phenylpyridine derivative **2j** (1.2 equiv) showed product formation **4j**.

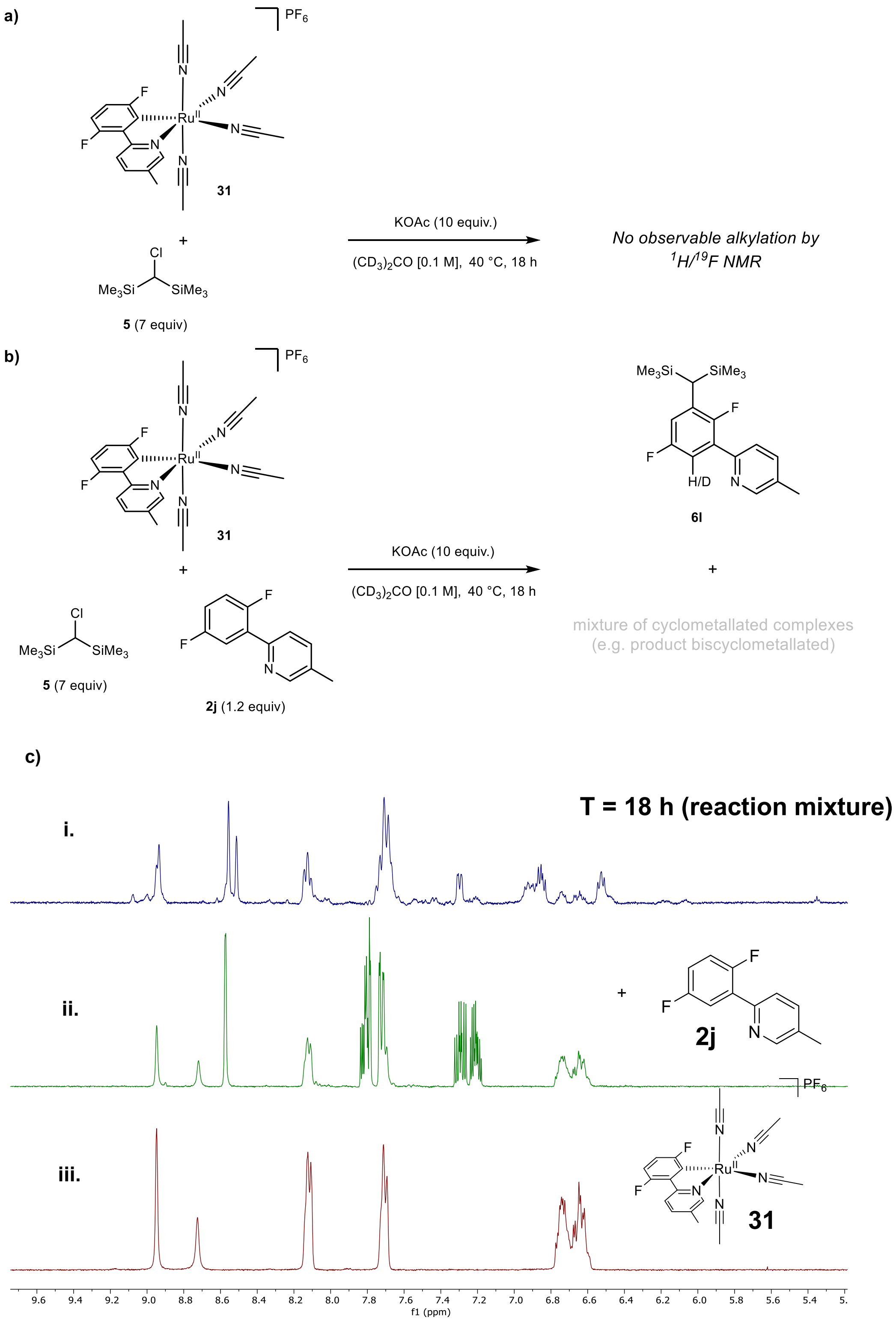

Supplementary Figure 19. Stoichiometric reactivity of monocyclometallated species **31**. **a)** without additional arene **2j**. **b)** with additional equivalent of arene **2j**. **c)** ^{1}H NMR Spectra [$(CD_3)_2CO$, 400 MHz] comparison of **i.** starting monocyclometallated species **31**, with **ii.** subsequent addition of arene **2j** and **iii.** complete reaction mixture. *n.b.* meta-functionalised products can undergo additional *ortho*-cyclometallation and as such leads to complex *in situ* mixtures before work-up.

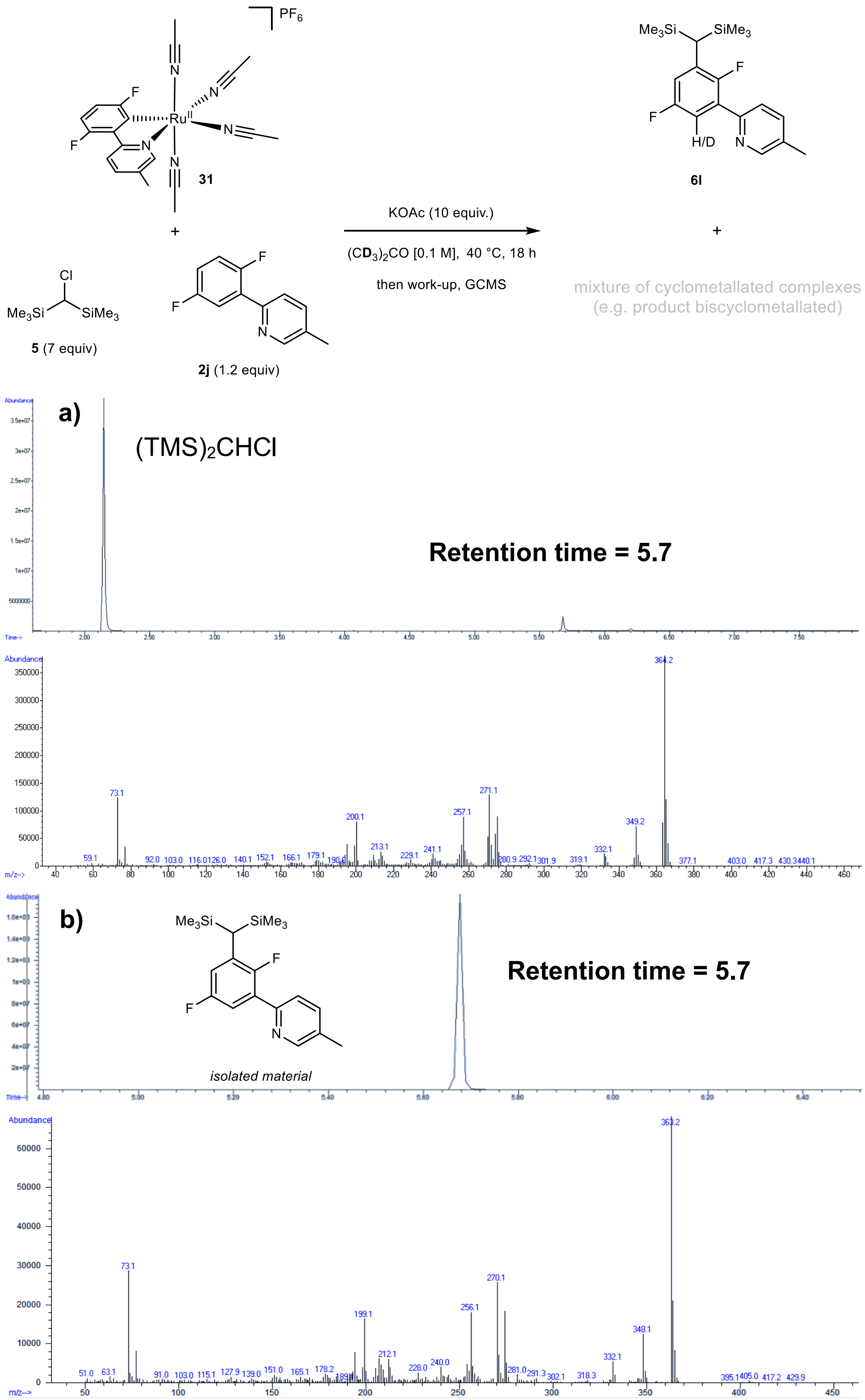

Supplementary Figure 20. Stoichiometric reactivity of monocyclometallated species with additional equivalent of arene. **a)**, GCMS chromatogram and m/z of the reaction mixture after work-up. **b)**, comparison of GCMS data with isolated sample of 2-{3-[bis(trimethylsilyl)methyl]-2,5-difluorophenyl}-5-methylpyridine **6l**.

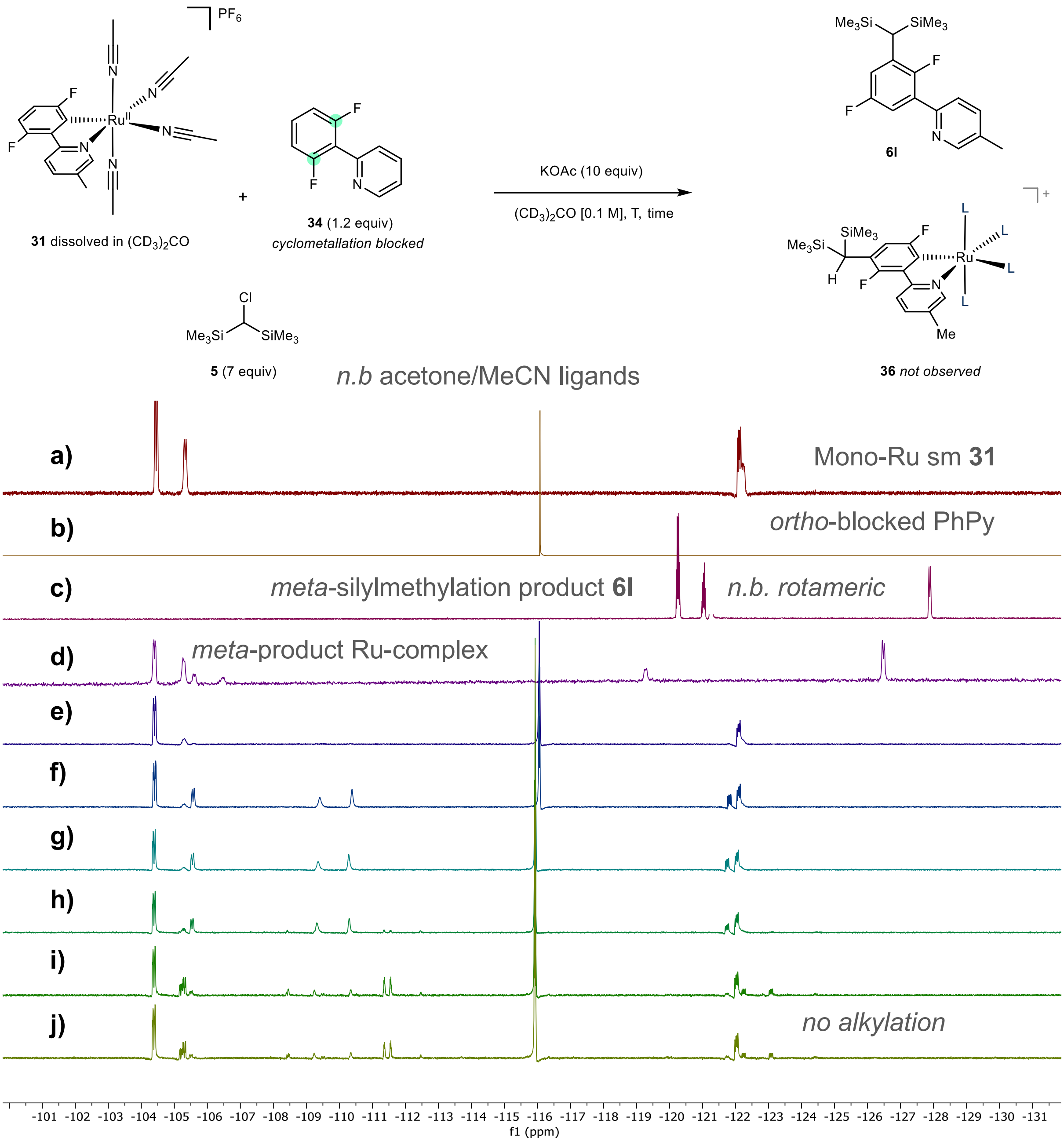

Supplementary Figure 21. Reaction of monocyclometallated ruthenium complex with the addition of additives and *ortho*-substituted 2-phenylpyridine **34** using ^{19}F NMR spectroscopy. **a**, Monocyclometallated complex **31** in d_6-$(CD_3)_2CO$ heated at 40 °C for 4 h. **b**, 2-(2,6-difluorophenyl)pyridine **34** in d_6-$(CD_3)_2CO$ heated at 40 °C for 4 h. **c**, 2-(3-[bis(trimethylsilyl)methyl)-2,5-difluorophenyl)-5-methylpyridine **6l**, *n.b.* that this compound and complexes thereof are rotameric – *see section 7.3*. **d**, Monocyclometallated complex of the *meta*-functionalised product **36**. **e**, Monocyclometallated ruthenium complex **31** + difluorophenylpyridine **34** in d_6-$(CD_3)_2CO$. **f**, Monocyclometallated ruthenium complex **31** + difluorophenylpyridine **34** in d_6-$(CD_3)_2CO$ heated at 40 °C for 4 h. **g**, Monocyclometallated ruthenium complex **31** + difluorophenyl)pyridine **34** + bis(trimethylsilyl)chloromethane **5** (7 equiv) in d_6-$(CD_3)_2CO$ heated at 40 °C for 4 h. **h**, Reaction **g** + KOAc (10 equiv), heated at 40 °C for 4 h. **i**, Reaction **h**, heated at 40 °C for 18 h. **j**, Reaction **h**, heated at 70 °C for 24 h.

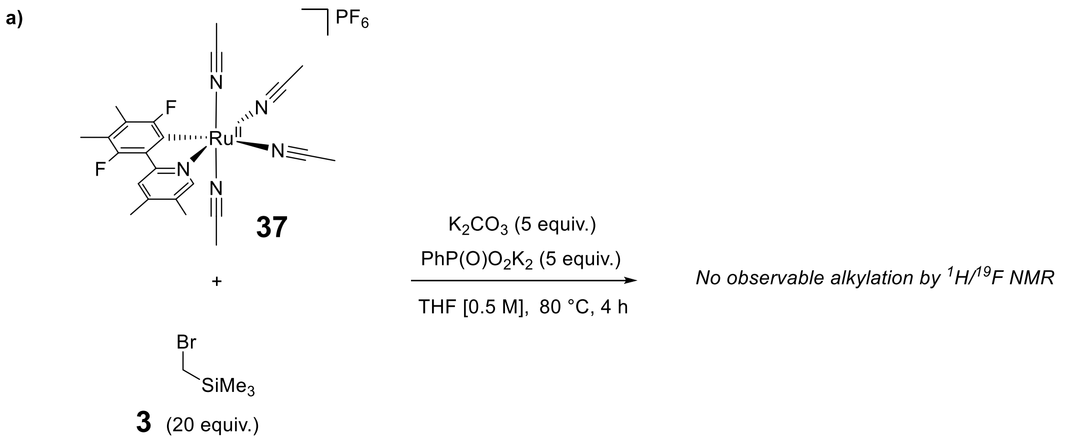

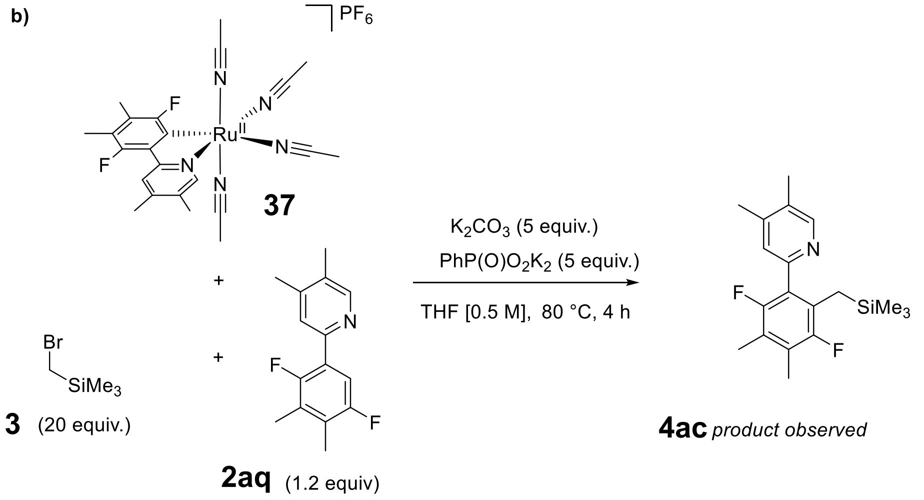

Supplementary Figure 22. Reaction of a different stoichiometric mono-cyclometallated ruthenium complex **37** using bromotrimethylsilyl methane **3**. **a)** Reaction of excess halide electrophile **3** with mono-cyclometallated ruthenium complex **37** gave no alkylated arene. **b)** Repeat reaction with added phenylpyridine derivative **2aq** (1.2 equiv) showed product formation **4ac**.

Stoichiometric reactivity of biscyclometallated ruthenium(II) complexes

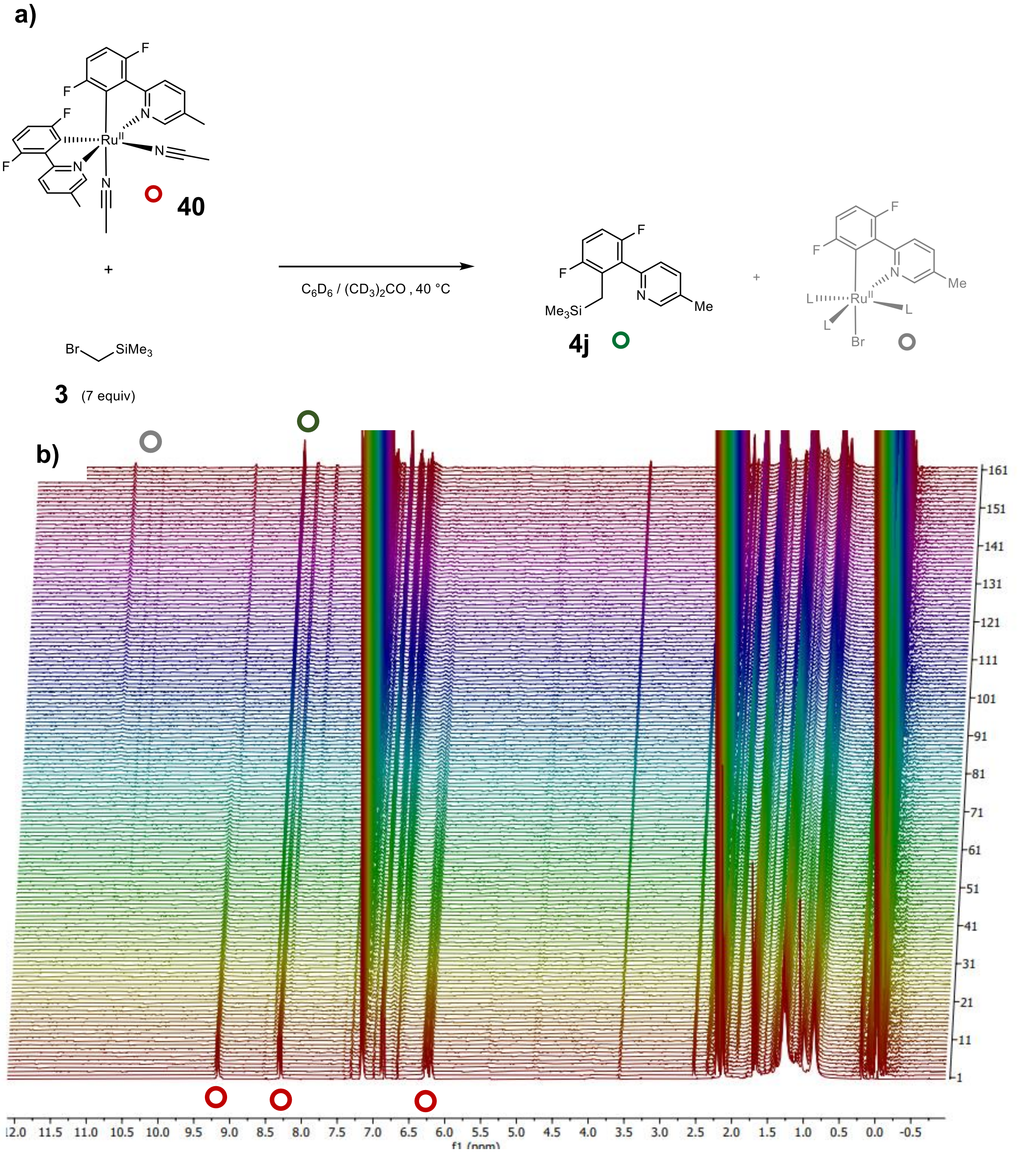

Supplementary Figure 23. a) Reaction of bis[2-(2,5-difluorophenyl)-5-methylpyridine] ruthenium(II) bisacetonitrile **40** with trimethylsilyl bromomethane **3**. **b)** Stacked ^{1}H NMR spectra (C_6D_6/$(CD_3)_2CO$, 500 MHz) during the reaction with the reaction components assigned using coloured circles (see key in section **a**).

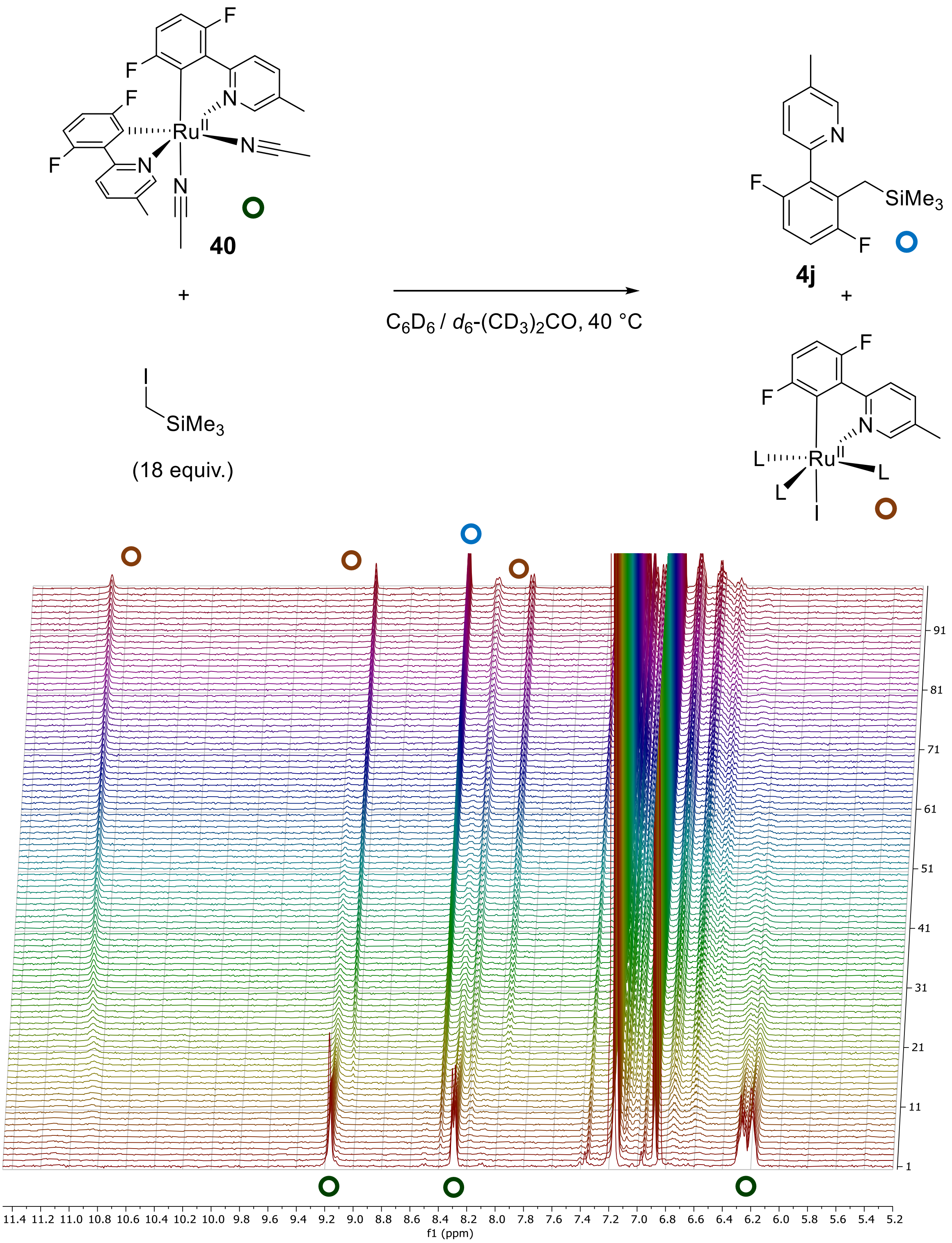

Supplementary Figure 24. Reaction of bis-[2-(2,5-difluorophenyl)-5-methylpyridine]ruthenium(II) bisacetonitrile **40** and iodotrimethylsilyl methane in C_6D_6 / d_6-$(CD_3)CO$ at 40 °C. Monitored by ^1H NMR spectroscopy – sample 98 NMR spectra showing consumption of ruthenium biscyclometallated starting complex **40** (O) and formation of

silylmethane-PhPy product **4j** (**O**), mono-cyclometallated PhPyRuI (**O**). Note that the stacked ^{1}H NMR spectra periodicity changed after approximately 8000 seconds.

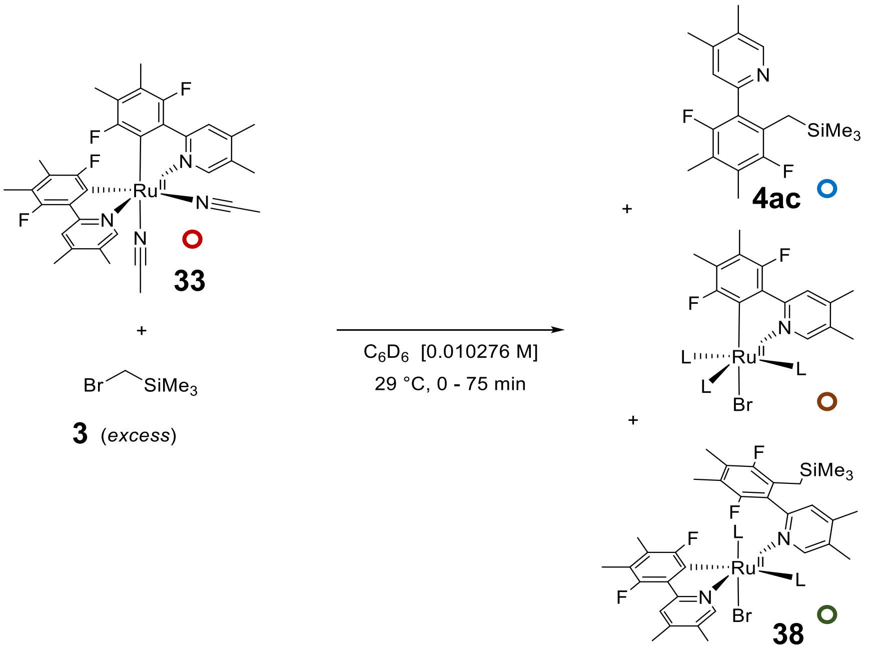

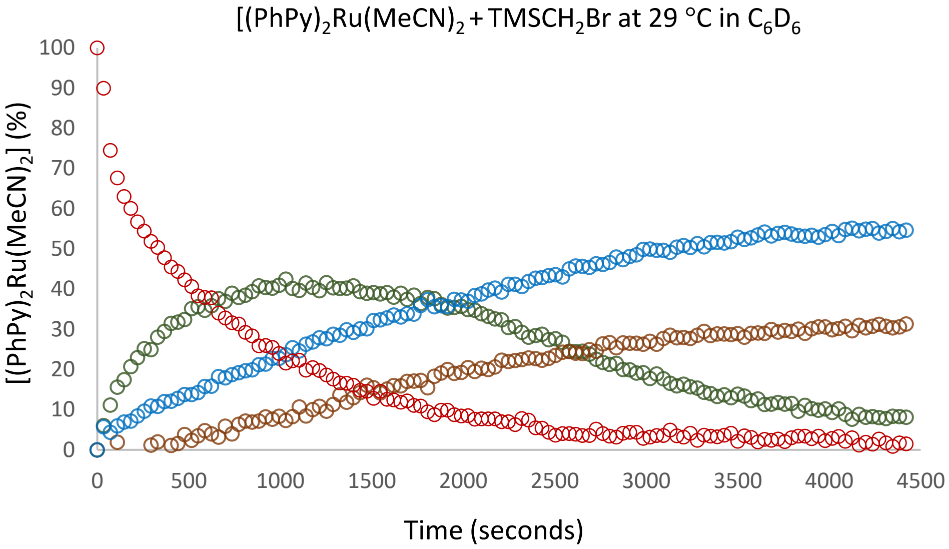

Supplementary Figure 25. Reaction of bis-[2-(2,5-difluoro-3,4-dimethylphenyl)-4,5-dimethylpyridine] ruthenium(II) bisacetonitrile **33** (**O**) and bromotrimethylsilyl methane **3** in C_6D_6 producing **2ac** (**O**). Reaction setup: ruthenium complex **33** (17 mg, 0.0206 mmol – 80% purity) was dissolved in anhydrous C_6D_6 (0.5 mL) in a septum-capped NMR tube and bromotrimethylsilyl methane **3** (20 equiv) added quickly before loading into a 500 MHz NMR spectrometer. The spin-lattice relaxation time (T_1) for analyses resonances was determined to be <1 seconds using the inversion recovery method and thus a d_1 = 5 s was used to allow adequate relaxation.

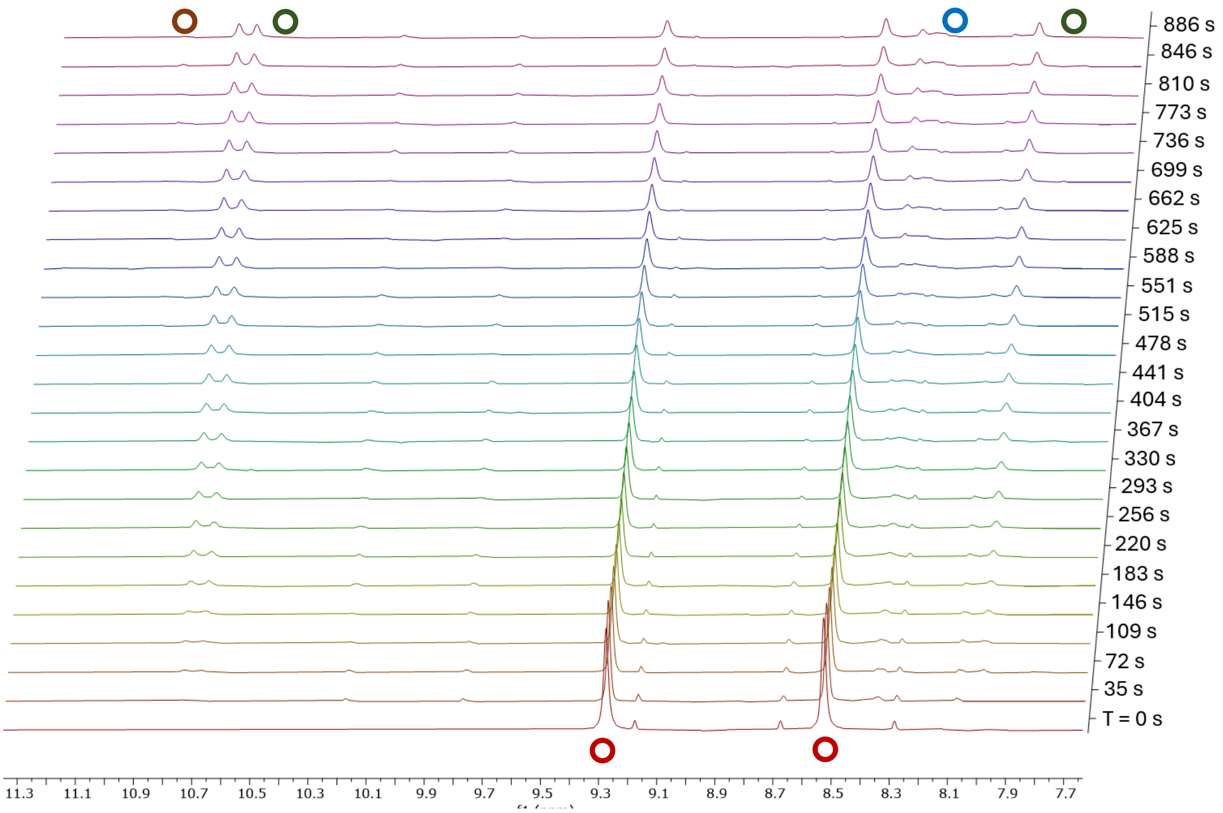

Supplementary Figure 26. Reaction of bis-[2-(2,5-difluoro-3,4-dimethylphenyl)-4,5-dimethylpyridine] ruthenium(II) bisacetonitrile **33** and bromotrimethylsilyl methane **3** in C_6D_6. Monitored by ^{1}H NMR spectroscopy – sample 25 NMR spectra showing consumption of ruthenium biscyclometallated starting complex **33** ( ) and formation of silylmethane-PhPy product **4ac** ( ), mono-cyclometallated PhPyRuBr ( ) and mono-cyclometallated PhPyRuBr(product) complex **38** ( ) ligated. Note that product de-complexation from PhPyRuBr(product) complex **38** ( ) was observed at higher temperatures.

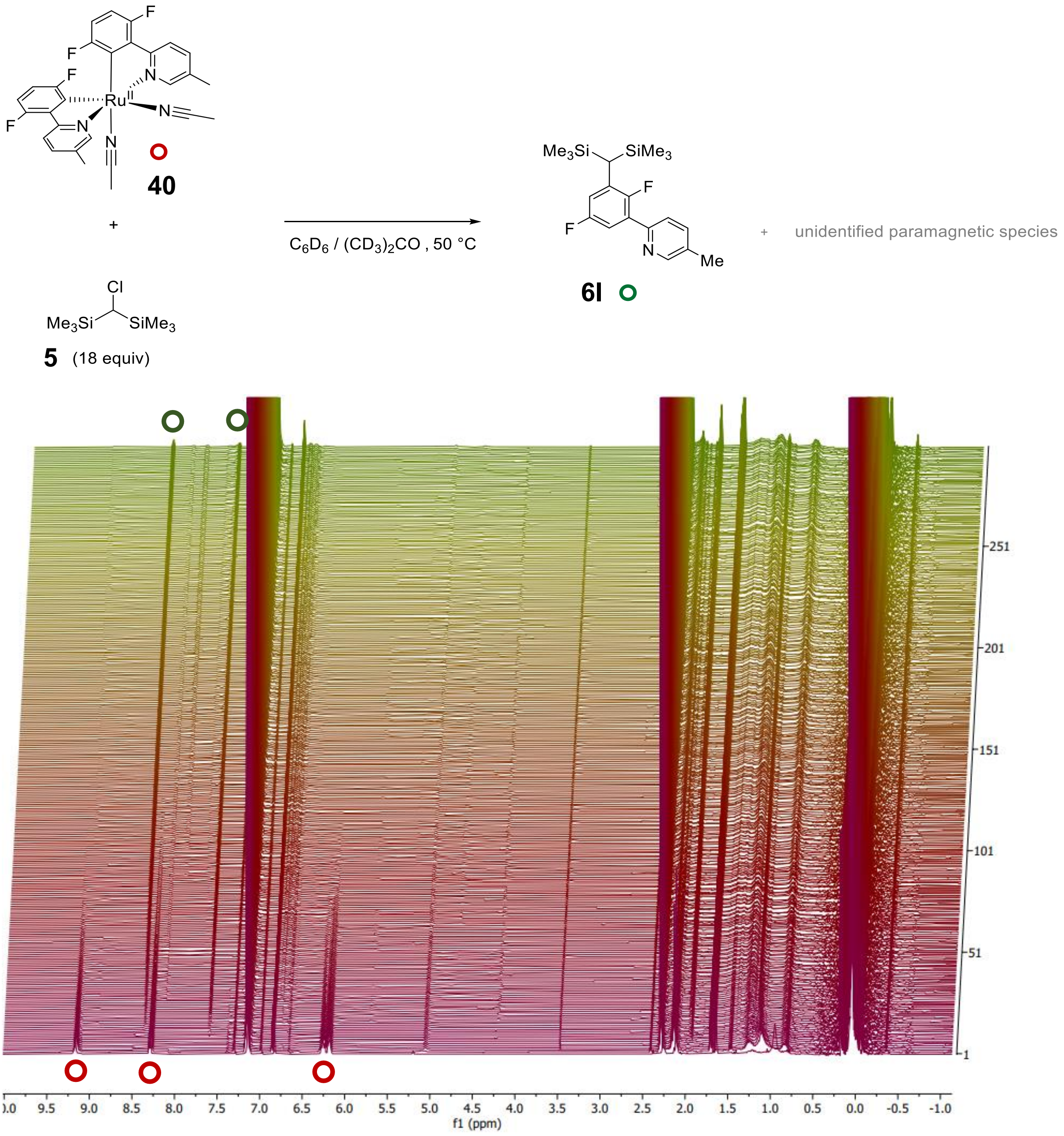

Supplementary Figure 27. Reaction monitoring of bis[2-(2,5-difluorophenyl)-5-methylpyridine] ruthenium(II) bisacetonitrile **40** with bis(trimethylsilyl)chloromethane **5**. Stacked ^{1}H NMR spectra (C_6D_6/$(CD_3)_2CO$, 500 MHz) during the reaction.

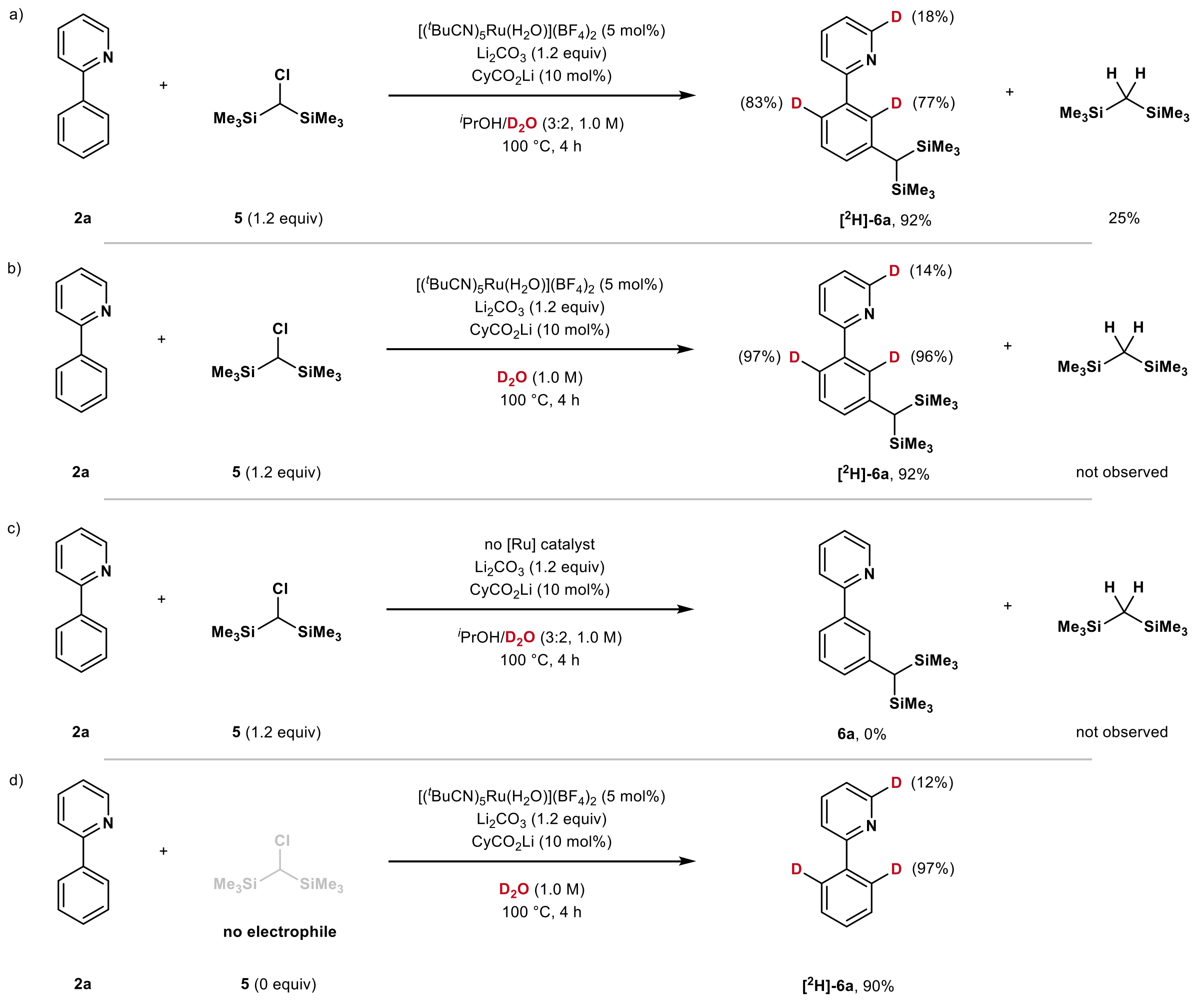

Supplementary Figure 28. Ruthenium-catalysed *meta*-selective *gem*-bissilylmethylation in the presence of d2-D_2O. **a)** standard reaction conditions using d2-D_2O instead of H_2O. **b)** Standard reaction conditions in the absence of ^{i}PrOH. **c)** Control reaction in the absence of ruthenium pre-catalyst. **d)** Control reaction in the absence of electrophile.

Reaction of biscyclometallated ruthenium complex with *gem*-bistrimethylsilyl chloromethane reagent – Monitored by ^{1}H NMR

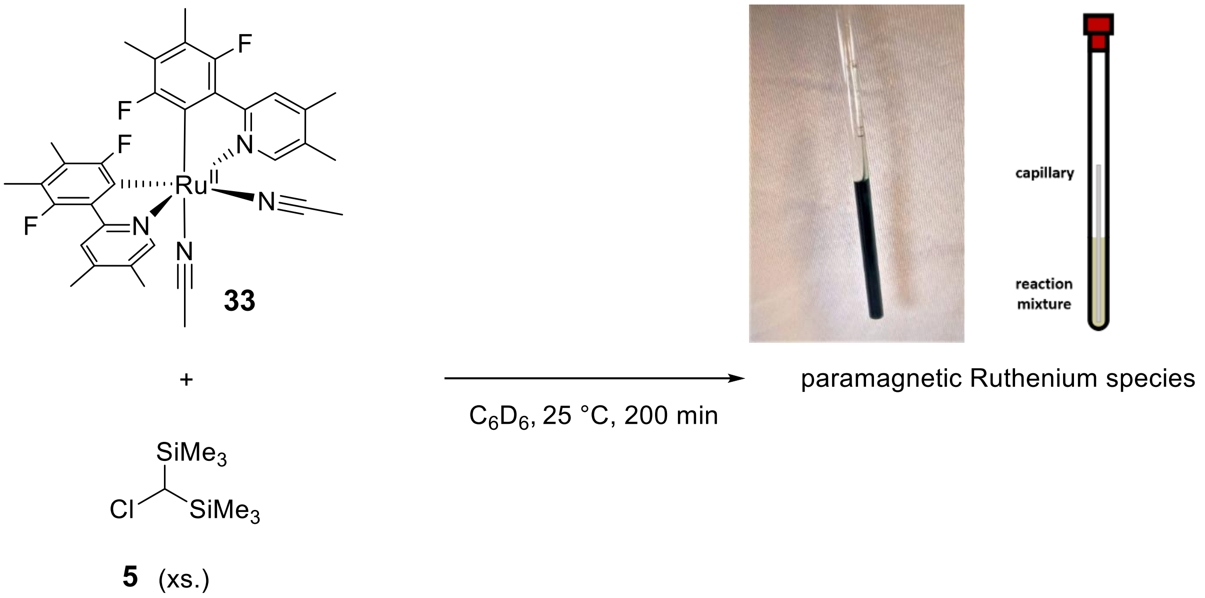

Supplementary Figure 29. Reaction of stoichiometric ruthenium complex in C_6D_6 at 25 °C monitored by ^{1}H NMR. Picture right: *J*-Youngs NMR tube containing the reaction mixture with a capillary insert containing C_6D_6. The separation of C_6D_6 (reaction mixture) and C_6D_6 (capillary) allows for use of the Evans NMR method for determination of magnetic susceptibility of the solution.

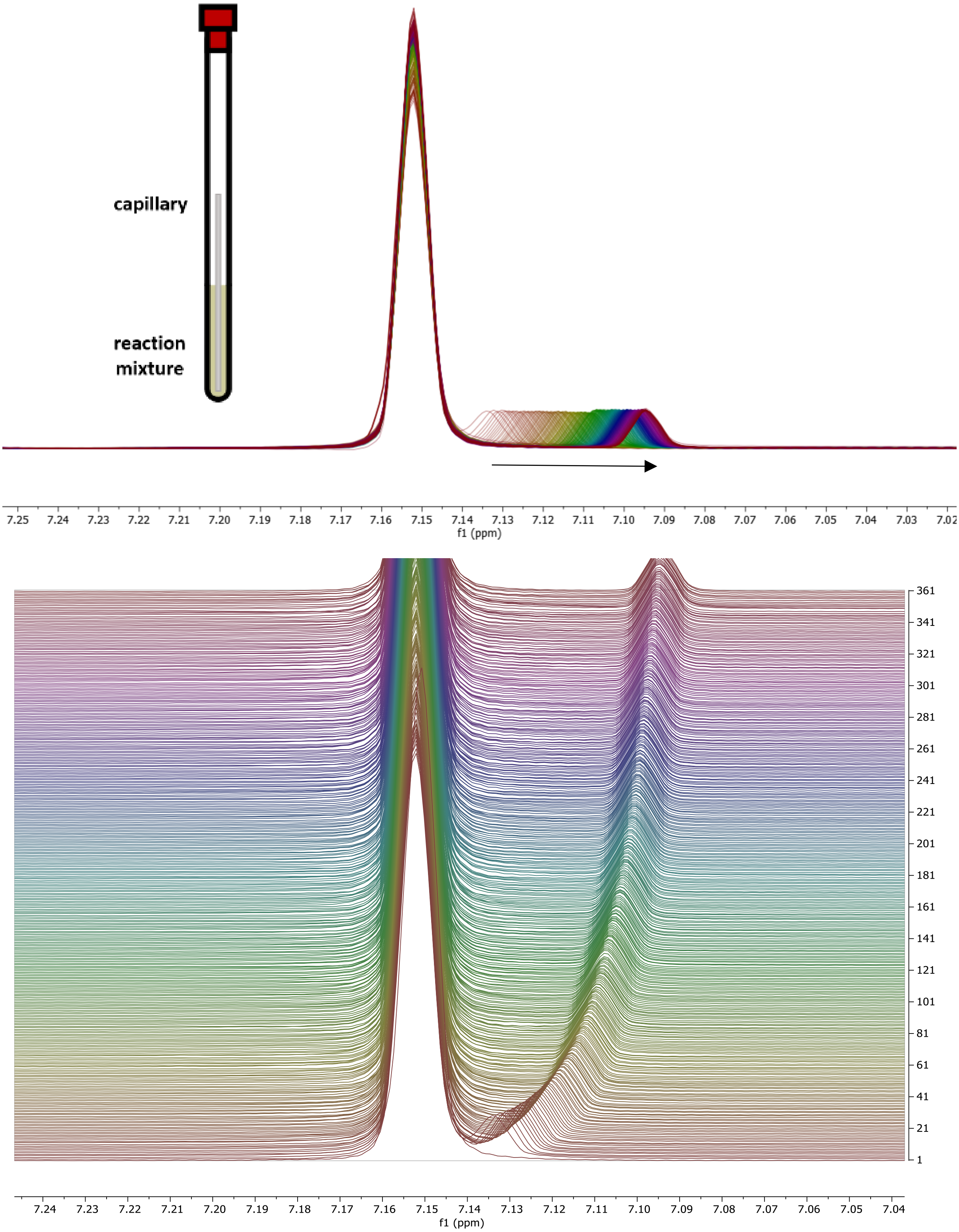

Supplementary Figure 30. Superimposed spectra (^{1}H, 361 NMR spectra) for the reaction of ruthenium biscyclometallated complex **33** with *gem*-bistrimethylsilyl chloromethane **5** showing δ 7.04 – 7.24 ppm region. Note: as paramagnetic material is formed, the chemical shifts of the proton residual C_6D_6 (solution) and C_6D_6 (capillary) diverge.

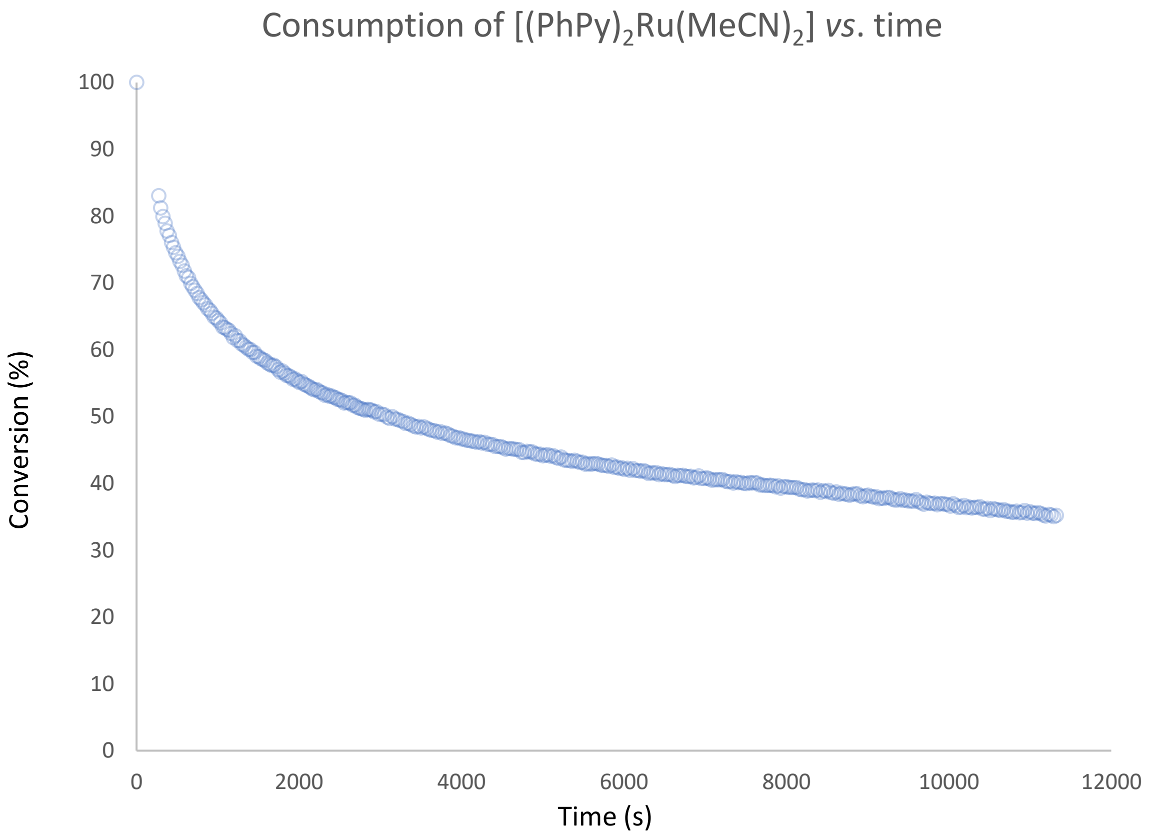

Supplementary Figure 31. Reaction monitored consumption of ruthenium biscyclometallated complex **33** on reaction with *gem*-bistrimethylsilyl chloromethane **5**.

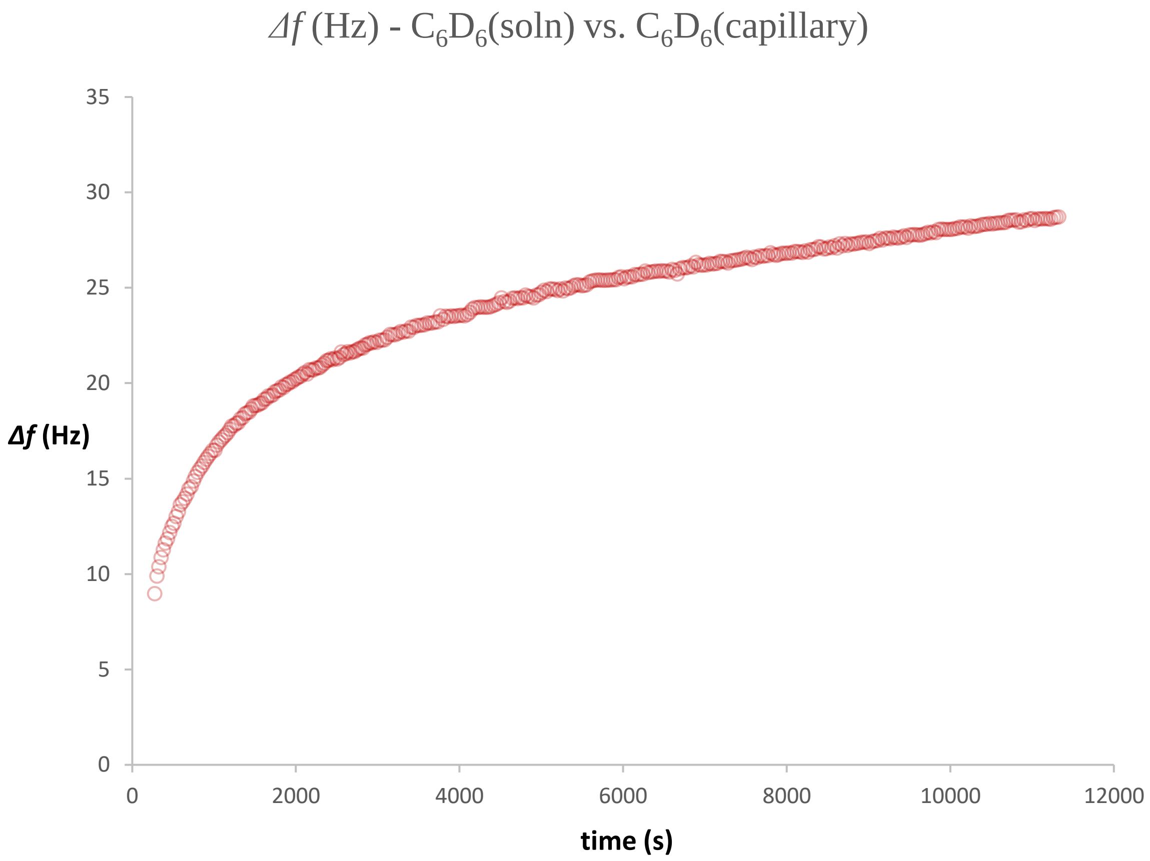

Supplementary Figure 32. Reaction monitoring – change in chemical shift of residual C_6D_6 signal (solution) *vs.* C_6D_6 signal (capillary) over time. Note: plot measures the difference in chemical shift (or frequency) between both signals during the reaction.

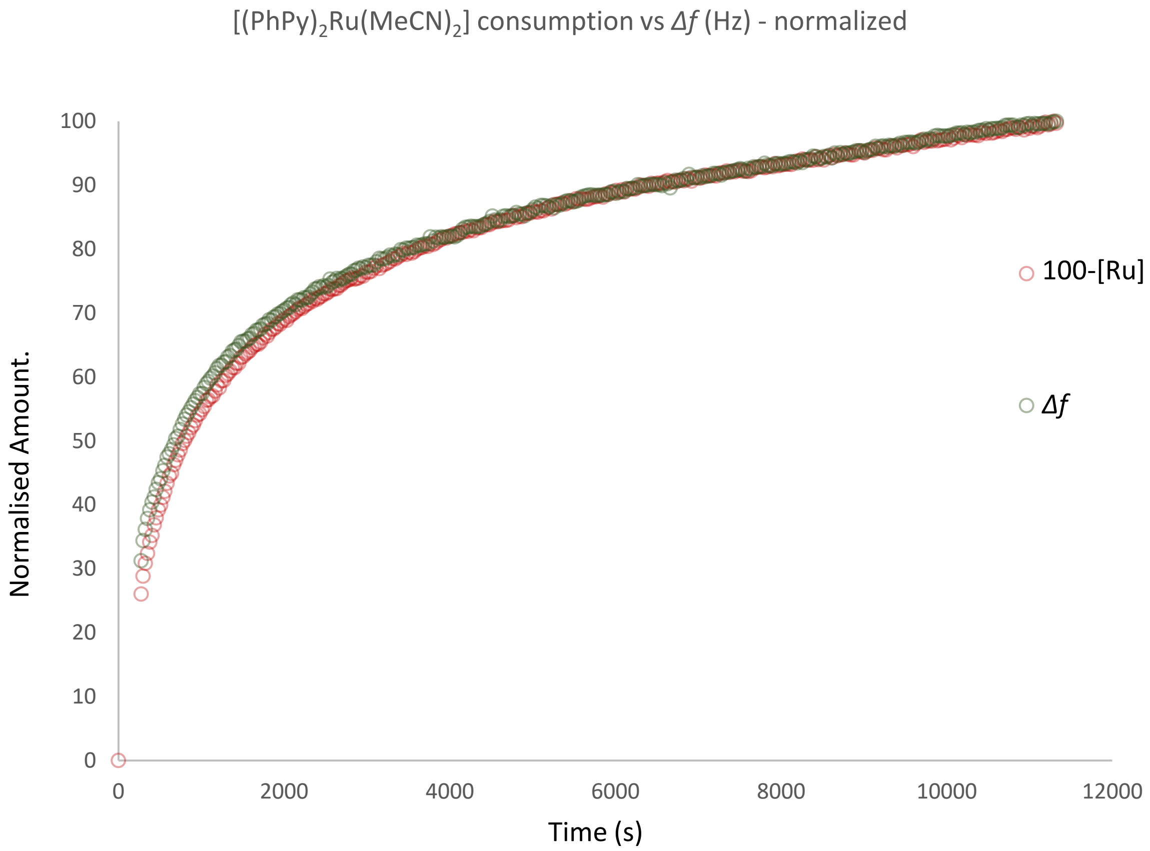

Supplementary Figure 33. Reaction monitoring – Graphical overlay of the consumption of biscyclometallated ruthenium complex **33** and the frequency difference observed between the residual C_6D_6 (solution) signal and C_6D_6 (capillary) signal.

Evans method calculation:

Number of unpaired electrons determined from magnetic moment μ, in units of Bohr magneton μ_B = 9.27 x 10^{-24} J T^{-1}.

Magnetic susceptibility of solution (χ_M) is related to the magnetic moment μ:

$$\mu = \sqrt{8(X_M T)}$$

T = temperature (K)

χ_M = molar magnetic susceptibility (cm^3/mol)

$$\chi_M = \frac{3\Delta f}{4\pi F c} = \frac{3(28.72)}{4\pi(500 * 10^6)(1.666 * 10^{-5})(0.65)} = 0.00126585\, mL/mol$$

$$\mu = \sqrt{8\chi_M T} = \sqrt{8 * 0.00126585 * 298} = 1.74\, \mu_B$$

Supplementary Equation 1. Determination of magnetic susceptibility of solution (χ_M) when Δf = 28.72 Hz. Note: NMR field = 500 MHz and concentration, c = 1.666 x 10^{-5} mL/mol and c*(0.65) represents conversion of [$(PhPy)_2Ru(MeCN)_2$] (*i.e* 65% conversion when Δf = 28.72 Hz) . The calculated magnetic moment, 1.74 μ_B corresponds to 1 unpaired electron.

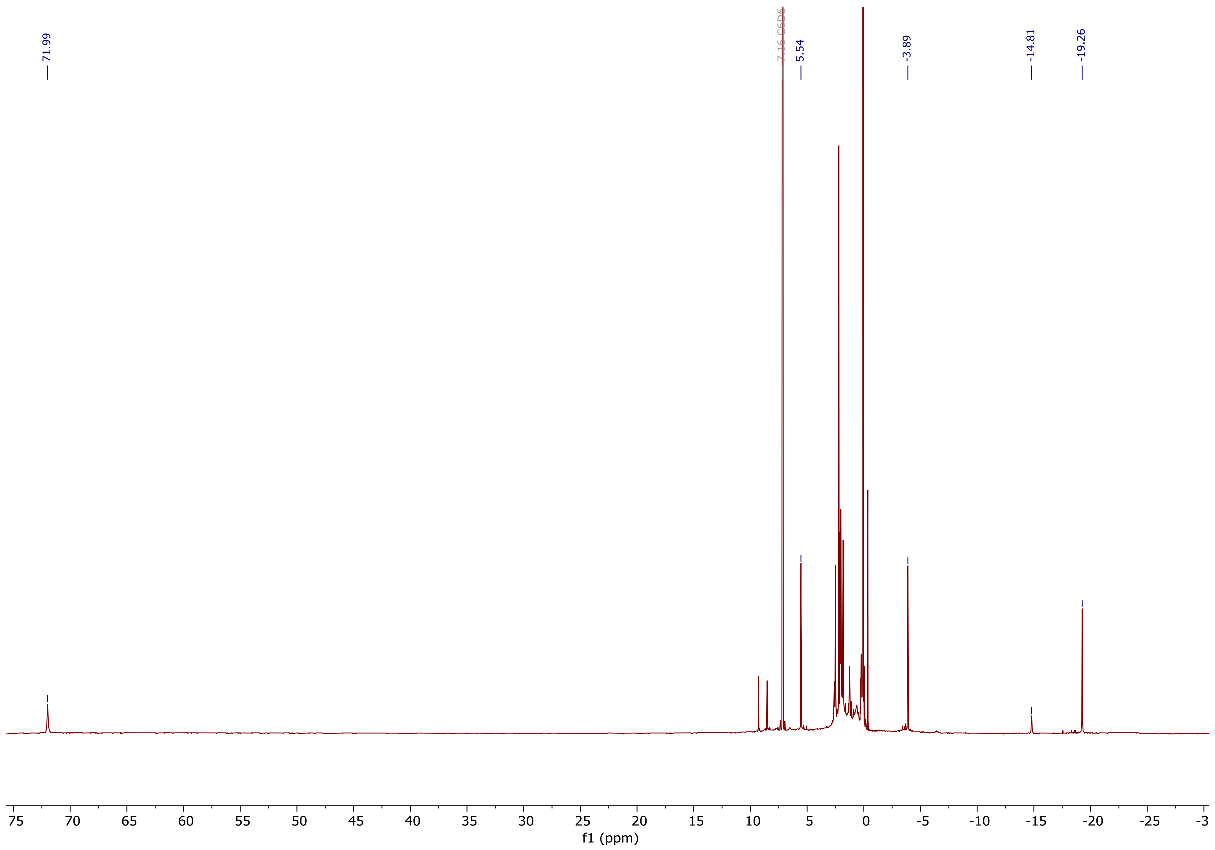

Supplementary Figure 34. Wide sweep width ^{1}H NMR (500 MHz, *d*$_6$-C_6D_6) of the reaction of ruthenium biscyclometallated complex **33** with *gem*-bistrimethylsilyl chloromethane **5**. Characteristic resonances: δ 71.99, 5.54, –3.89, –14.81, –19.26.

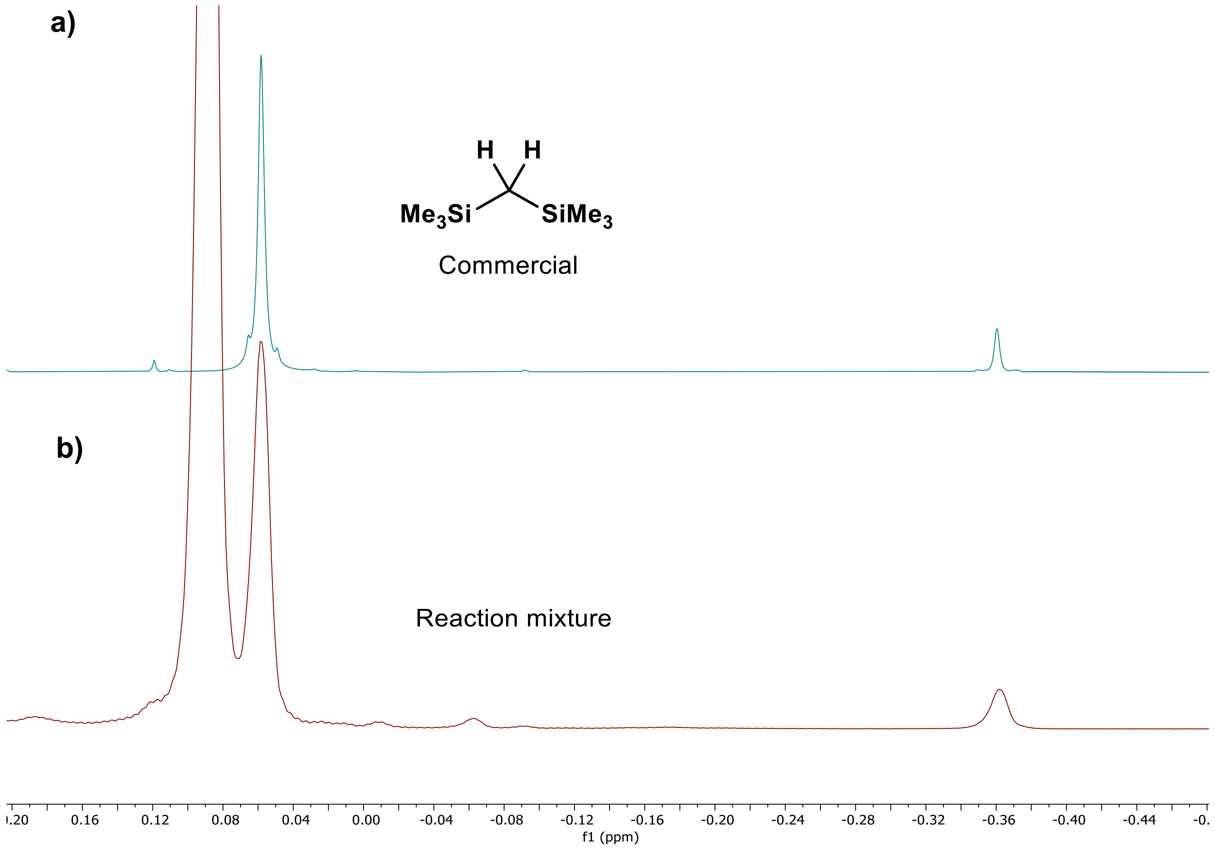

Supplementary Figure 35. ^{1}H NMR (500 MHz, d_6-C_6D_6) 0.00 ppm region - Fate of the chloride electrophile in the reaction of ruthenium biscyclometallated complex **33** with *gem*-bistrimethylsilyl chloromethane **5**. **a**) spectra for a commercial sample of bis(trimethylsilyl)methane. **b)** reaction mixture.

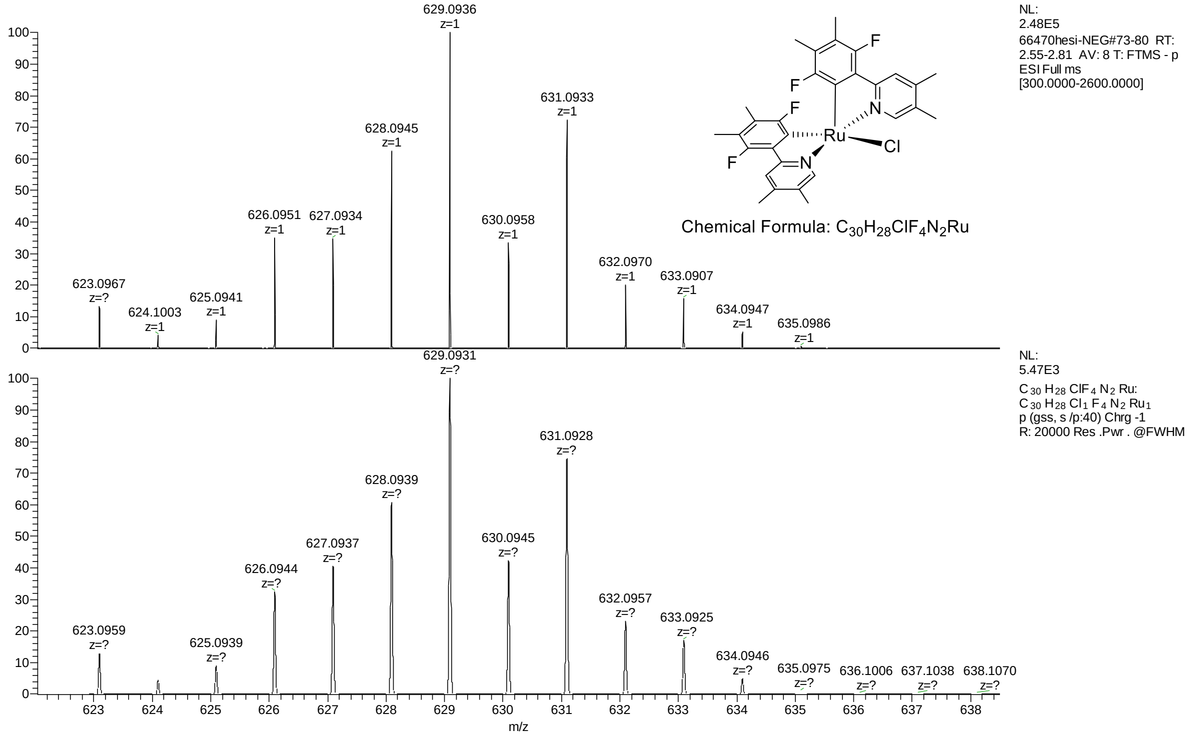

Supplementary Figure 36. Mass spectroscopy (HRMS – ESI$^-$) of the reaction mixture containing paramagnetic ruthenium species – reaction of $(PhPy)_2Ru(MeCN)_2$ with chlorobistrimethylsilyl methane. **a)** detected m/z. **b)** simulated m/z for $C_{30}H_{28}ClF_4N_2Ru$.

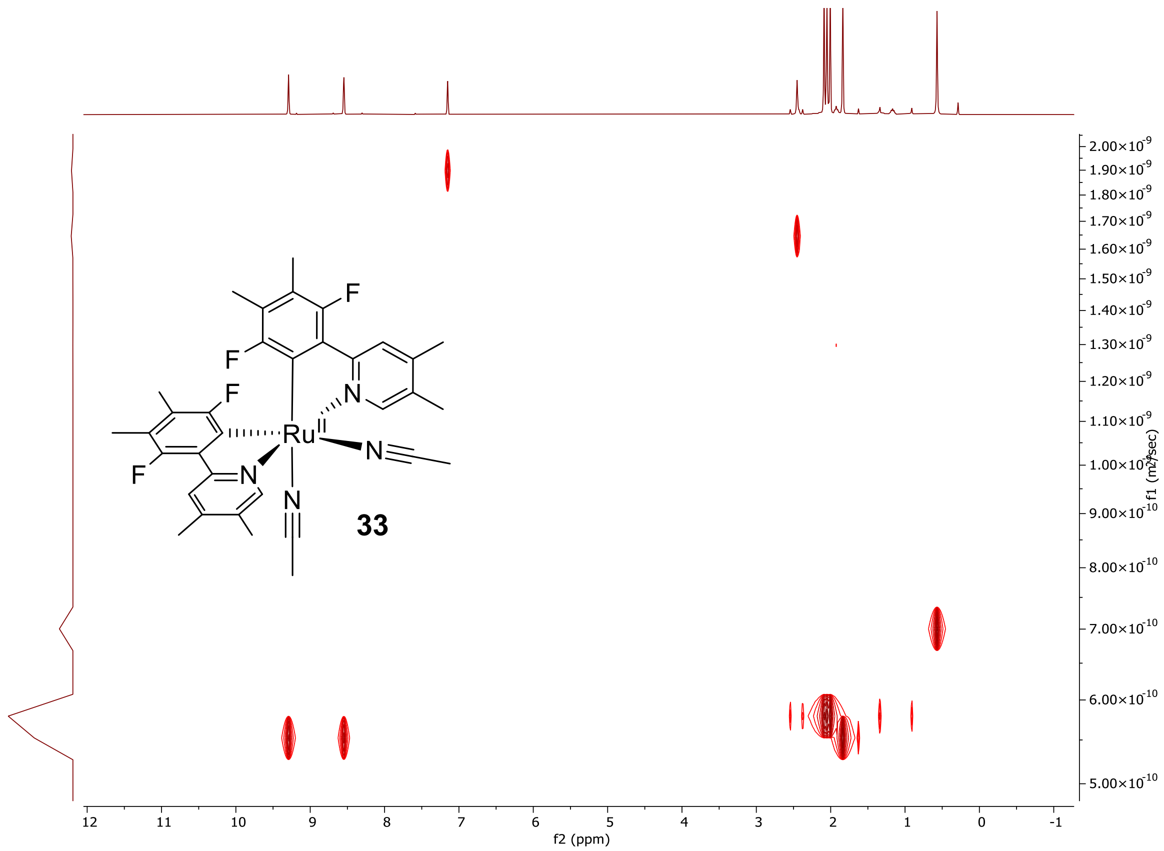

Supplementary Figure 37. Diffusion ordered spectroscopy (DOSY NMR) of bis-[2-(2,5-difluoro-3,4-dimethylphenyl)-4,5-dimethylpyridine] ruthenium(II) bisacetonitrile [$(PhPy)_2Ru(MeCN)_2$] **33** – (400 MHz, d_6-C_6D_6, 298 K). Observed diffusion coefficient, D = 5.513 m^2s^{-1} x 10^{-10}.

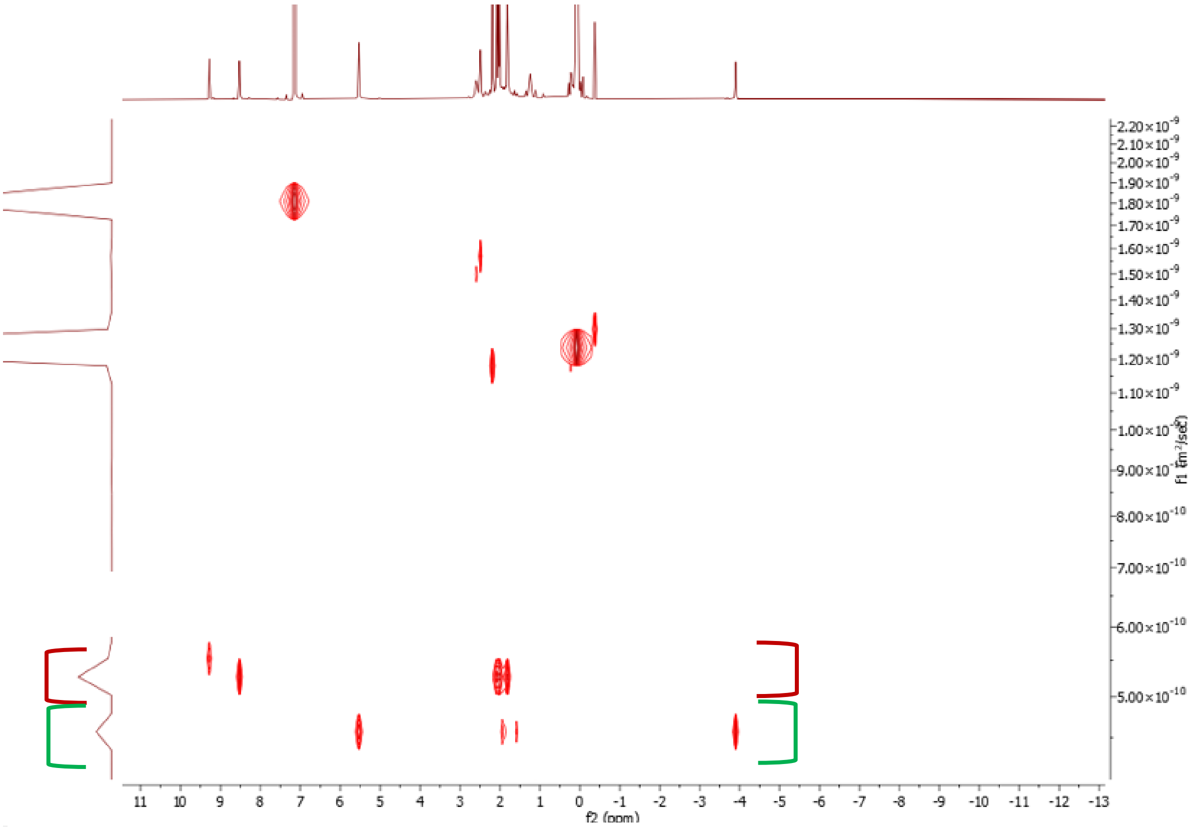

Supplementary Figure 38. Diffusion ordered spectroscopy (DOSY NMR) of a mixture containing both bis-[2-(2,5-difluoro-3,4-dimethylphenyl)-4,5-dimethylpyridine] ruthenium(II) bisacetonitrile [$(PhPy)_2Ru(MeCN)_2$] **33** and the formed paramagnetic species **39** from reaction with chlorobistrimethylsilyl methane – (400 MHz, d_6-C_6D_6, 298 K). Observed diffusion coefficient for [$(PhPy)_2Ru(MeCN)_2$] **33** D = 5.284 m^2s^{-1} x 10^{-10} ( ) and for Ru(III) species D = 4.554 m^2s^{-1} x 10^{-10} ( ).

Cyclic voltammetry (CV) of ruthenacycles

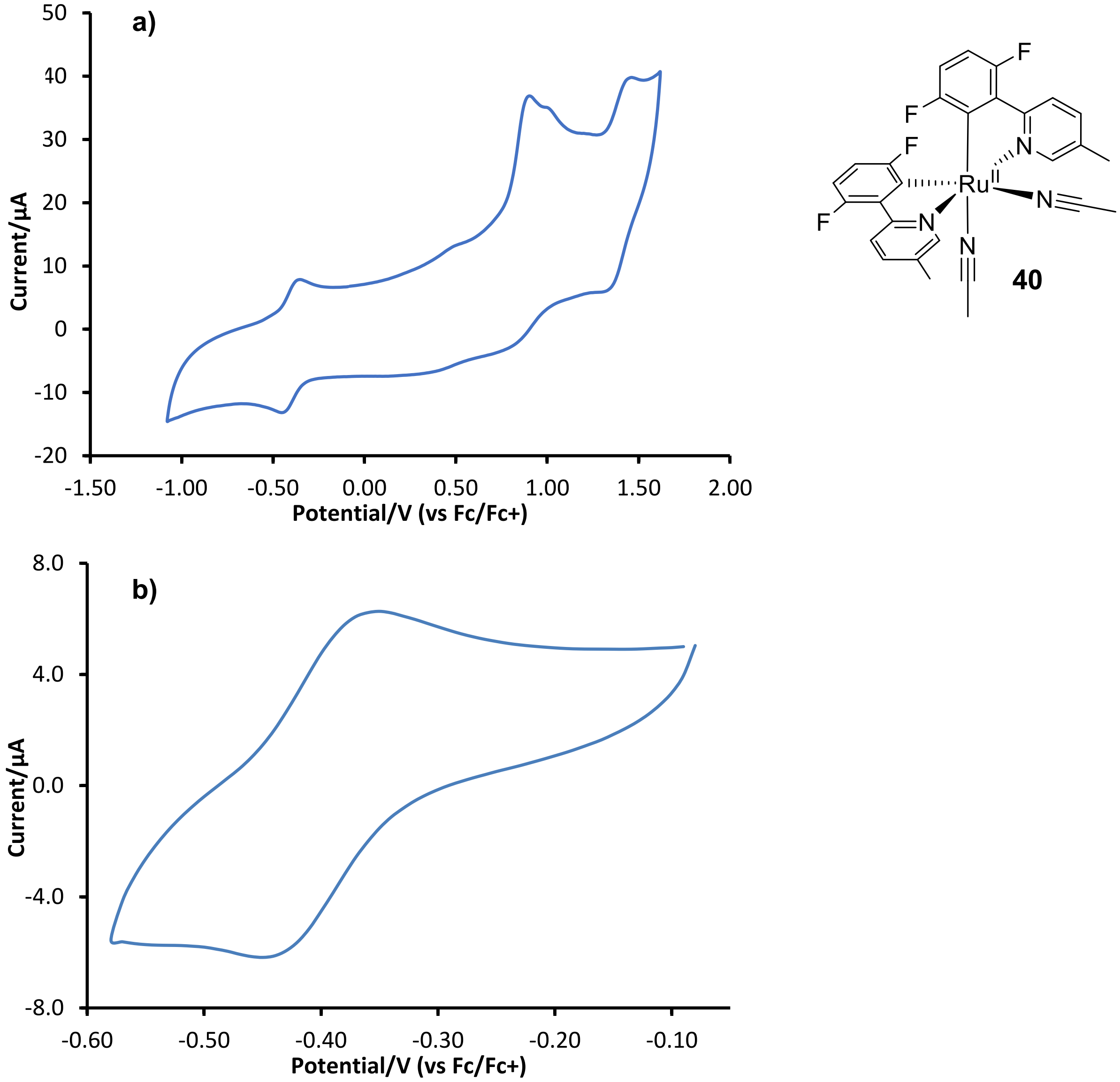

Supplementary Figure 39. Cyclic voltammogram of bis[2-(2,5-difluorophenyl)-5-methylpyridine] ruthenium(II) bisacetonitrile [$(PhPy)_2Ru(MeCN)_2$] (5 mM) in MeCN and $LiClO_4$ (0.1 M) **40**. **a)**, full CV. **b)**, CV of –0.37 V wave. Scan rate: 100 mV/s.

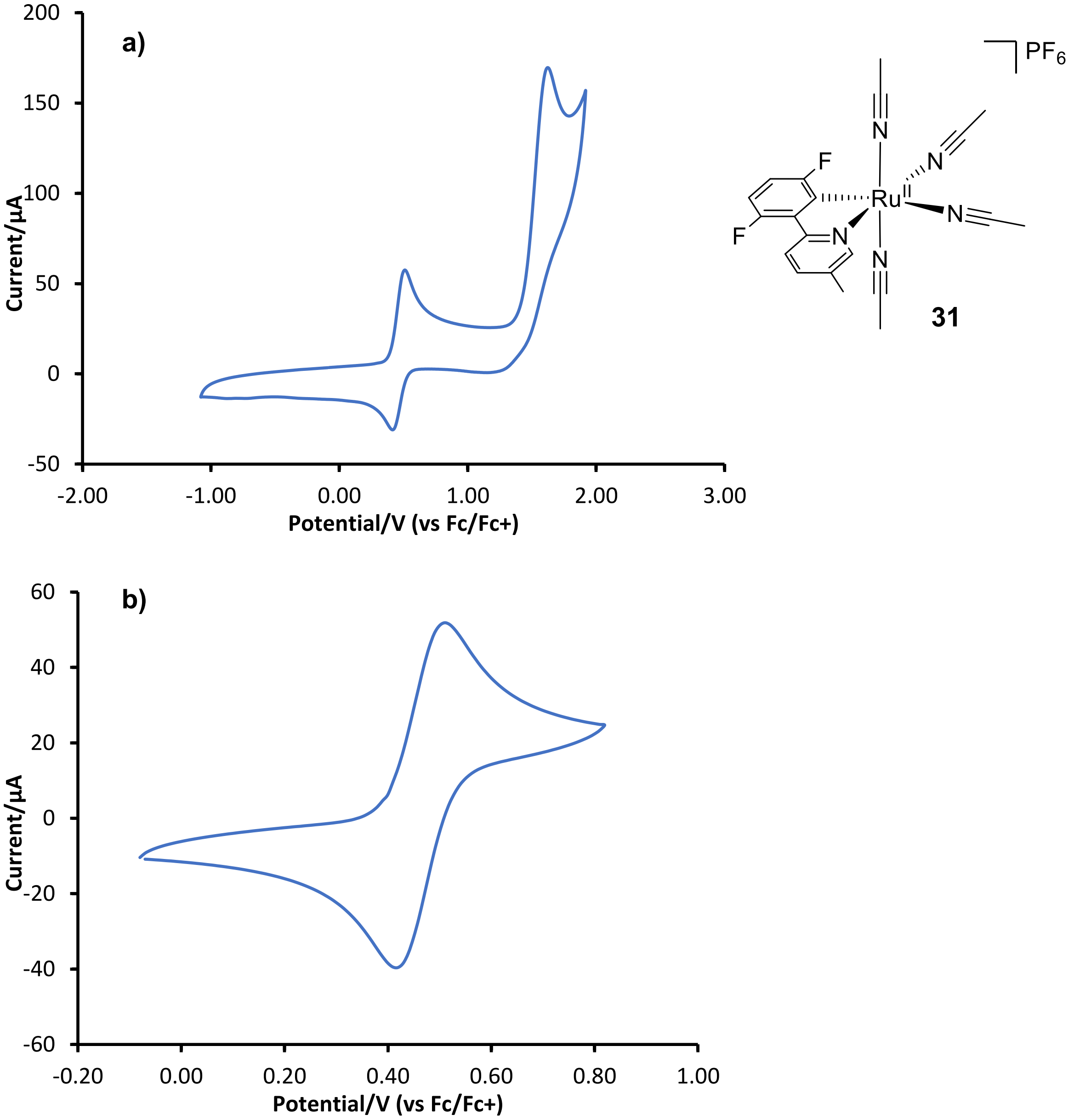

Supplementary Figure 40. Cyclic voltammogram of [2-(2,5-difluorophenyl)-5-methylpyridine] ruthenium(II) tetraacetonitrile [(PhPy)Ru(MeCN)$_4$] **31**. (5 mM) in MeCN and $LiClO_4$ (0.1 M) **a)**, full CV. **b)**, CV of 0.49 V wave. Scan rate: 100 mV/s.

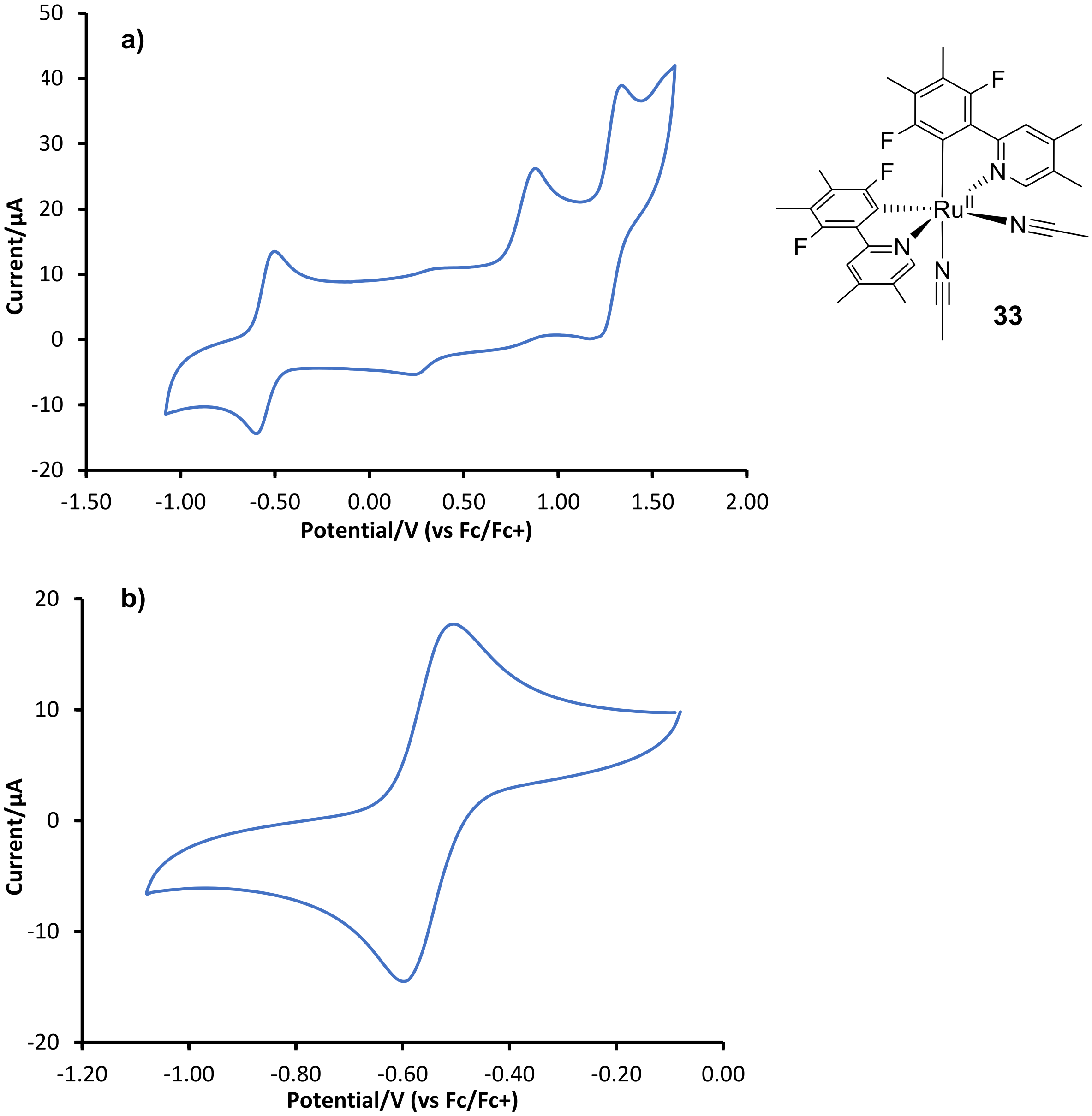

Supplementary Figure 41. Cyclic voltammogram of bis[2-(2,5-difluoro-3,4-dimethylphenyl)-4,5-dimethylpyridine] ruthenium(II) bisacetonitrile [$(PhPy)_2Ru(MeCN)_2$] (5 mM) in MeCN and $LiClO_4$ (0.1 M) **33**. **a)**, full CV. **b)**, – 0.51 V wave CV. Scan rate: 100 mV/s.

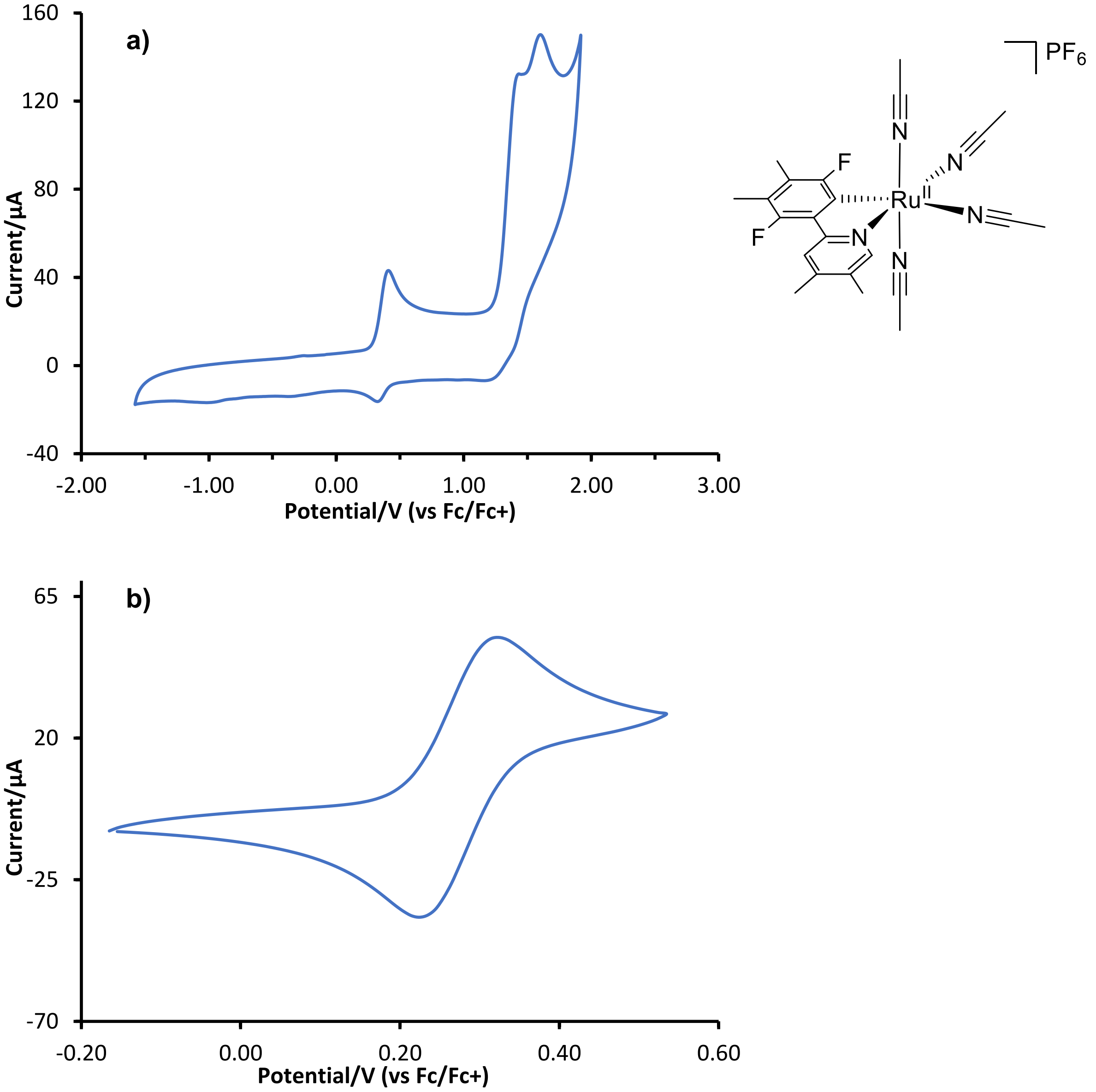

Supplementary Figure 42. Cyclic voltammogram of [2-(2,5-difluoro-3,4-dimethylphenyl)-4,5-dimethylpyridine] ruthenium(II) tetraacetonitrile [(PhPy)Ru(MeCN)$_4$] **37** (5 mM) in MeCN and $LiClO_4$ (0.1 M). **a)**, full CV. **b)**, 0.29 V wave CV. Scan rate: 100 mV/s.

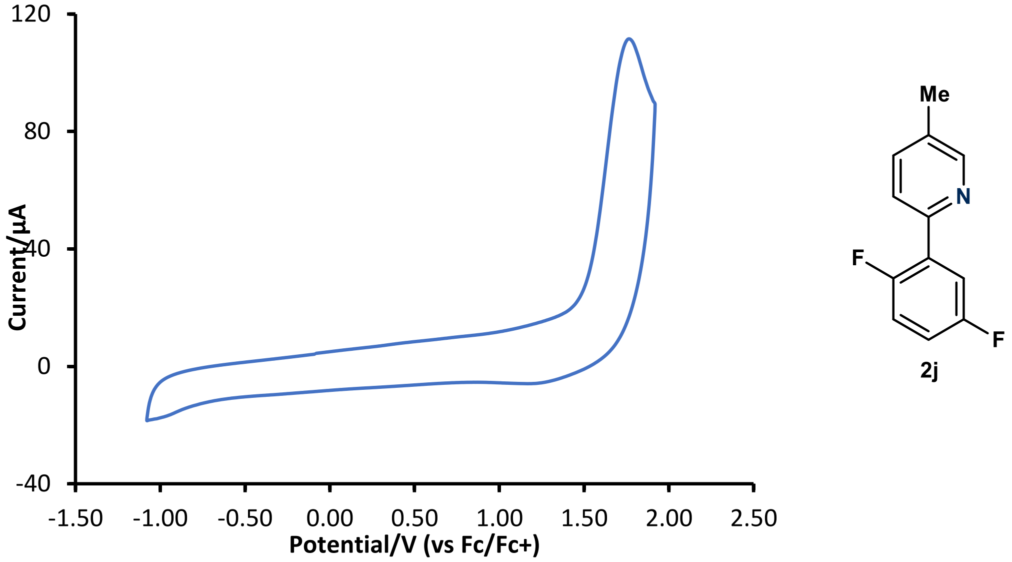

Supplementary Figure 43. Cyclic voltammogram of bis[2-(2,5-difluorophenyl)-5-methylpyridine] (5 mM) in MeCN and $LiClO_4$ (0.1 M) **2j**. Scan rate: 100 mV/s.

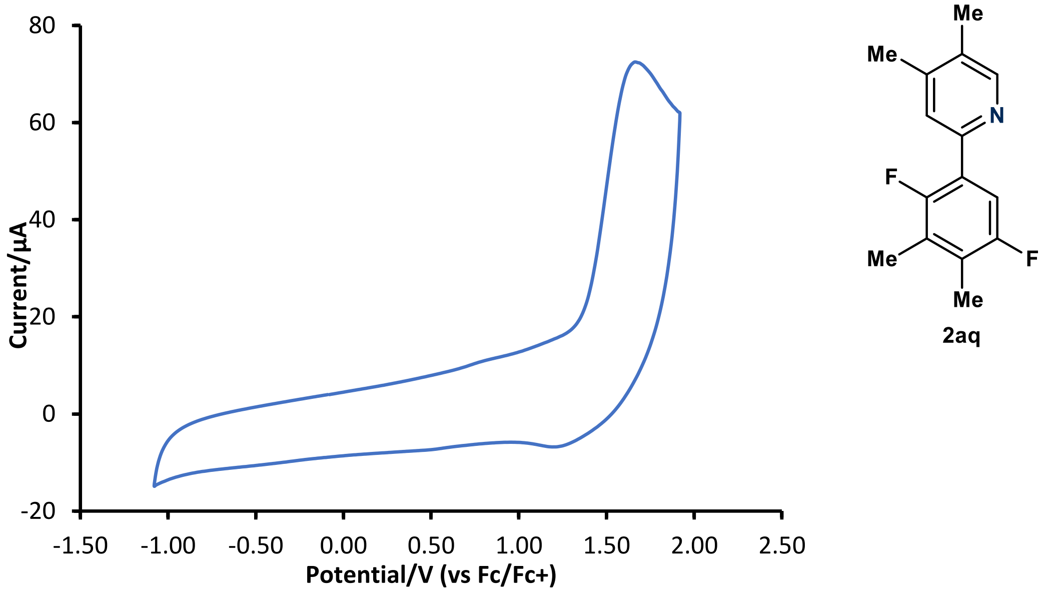

Supplementary Figure 44. Cyclic voltammogram of [2-(2,5-difluoro-3,4-dimethylphenyl)-4,5-dimethylpyridine] **2aq** (5 mM) in MeCN and $LiClO_4$ (0.1 M). Scan rate: 100 mV/s.

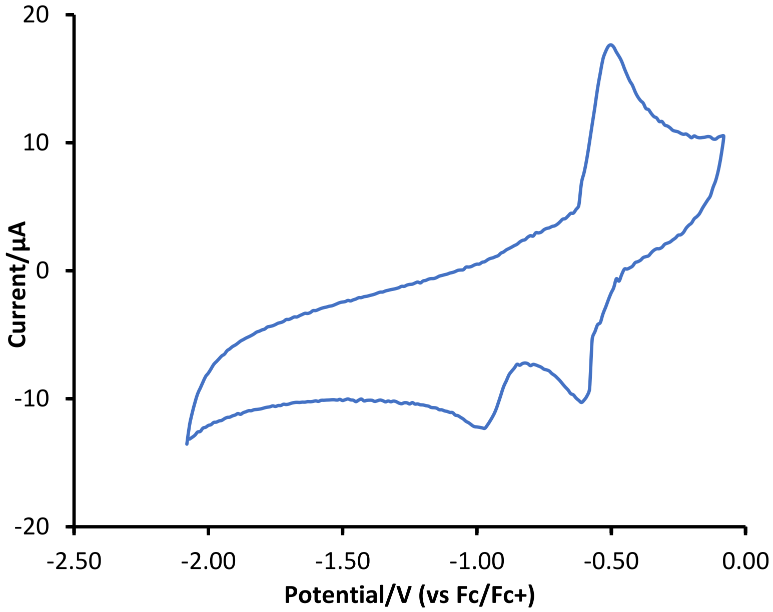

Supplementary Figure 45. Cyclic voltammogram of paramagnetic ruthenium species formed from reaction of bis[2-(2,5-difluoro-3,4-dimethylphenyl)-4,5-dimethylpyridine]ruthenium(II) bisacetonitrile [$(PhPy)_2Ru(MeCN)_2$] **33** (5 mM) in MeCN and $LiClO_4$ (0.1 M) with bis(trimethylsilyl)chloromethane **5**. Scan rate: 100 mV/s.

Procedures and characterisation for synthesised reagents and substrates

Lithium cyclohexane carboxylate

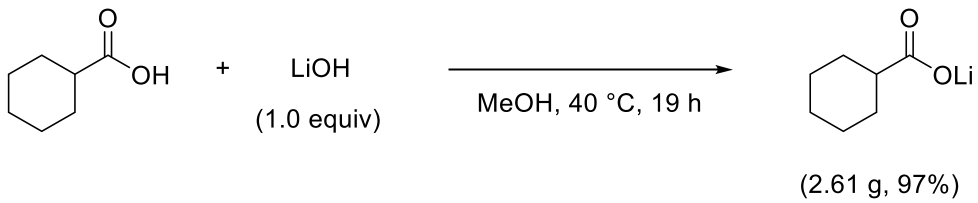

Cyclohexane carboxylic acid (2.56 g, 20.0 mmol) and lithium hydroxide (479.0 mg, 20.0 mmol, 1.0 equiv.) were reacted in methanol (40 mL) at 40 °C for 19 hours. The solvent was removed *in vacuo* (500 to 15 mbar, 40 °C) and the solid washed with pentane (150 mL) to give lithium cyclohexane carboxylate (2.61 g, 19.4 mmol, 97%) as an amorphous colourless solid.

IR: (*neat*, cm^{-1})

2924.8 (s), 2851.0 (s), 1704.8 (s), 1644.0 (s), 1577.7 (s), 1419.7 (s).

^{1}H NMR: (500 MHz, d_4-MeOD)

2.14 (tt, *J* = 11.5, 3.5 Hz, 1H), 1.92 – 1.83 (m, 2H), 1.78-1.69 (m, 2H), 1.68 – 1.59 (m, 1H), 1.40 (qd, *J* = 12.0, 3.2 Hz, 2H), 1.33 – 1.16 (m, 3H).

^{13}C NMR: (126 MHz, d_4-MeOD)

182.8, 46.1, 29.8, 25.9, 25.7.

2-Phenyl-3-methylpyridine

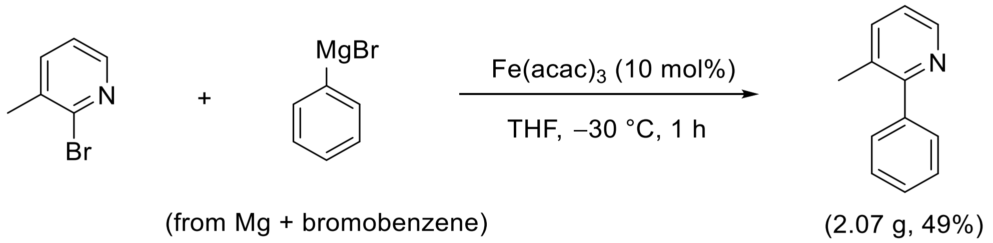

Bromobenzene (5.2 mL, 50 mmol) was added dropwise (over approximately 30 minutes) to a stirred mixture of magnesium (1.82 g, 75.0 mmol) and iodine (4.0 mg, 16 µmol) in anhydrous tetrahydrofuran (100 mL) at 19 °C. *n.b* Reaction was exothermic (19 °C to 30 °C). The reaction was stirred for 2 hours before use. The formed Grignard reagent – phenylmagnesium bromide – was transferred to a stirred mixture of 2-bromo-3-methylpyridine (2.8 mL, 25.0 mmol) and iron(III) acetylacetonate (871 mg, 2.5 mmol) in anhydrous tetrahydrofuran (30 mL) at –30 °C. The reaction was stirred for 1 hour and warmed to room temperature before the addition of water (100 mL). The organic phase was extracted using diethylether (100 mL x 3), dried ($MgSO_4$), filtered and the solvent removed *in vacuo* (500 to 200 mbar, 40 °C) to give an orange oil that was purified by flash column chromatography (SiO_2, 50 g, biotage isolera, hexane/ethyl acetate – 100/0 to 60/40, 60 mL/min) to give 2-phenyl-3-methylpyridine (2.07 g, 12.2 mmol, 49%) as a yellow oil.

TLC: R_f = 0.5 (1:1 hexane/diethylether) [UV]

^{1}H NMR: (500 MHz, $CDCl_3$)

8.55 (dd, *J* = 4.9, 1.7 Hz, 1H), 7.64 – 7.57 (m, 1H), 7.57 – 7.50 (m, 2H), 7.50 – 7.44 (m, 2H), 7.43 – 7.37 (m, 1H), 7.20 (dd, *J* = 7.7, 4.8 Hz, 1H), 2.37 (s, 3H).

^{13}C NMR: (126 MHz, $CDCl_3$)

158.6, 146.8, 140.4, 138.7, 130.9, 129.0, 128.2, 128.0, 122.1, 20.1.

Data were in accordance with those previously reported[1].

2-(2,5-Difluorophenyl)-5-methylpyridine 2j

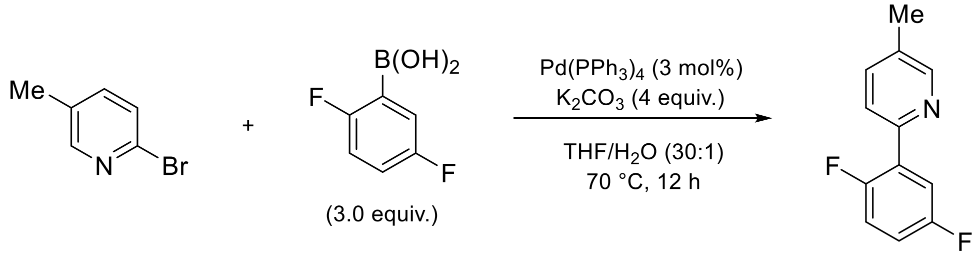

2-Bromo-5-methylpyridine (1.72 g, 10.0 mmol) was reacted with (2,5-difluorophenyl)boronic acid (4.70 g, 30.0 mmol, 3.0 equiv.) in the presence of tetrakis(triphenylphosphine)palladium(0) (346.6 mg, 0.300 mmol, 3 mol%) and potassium carbonate (5.52 g, 40.0 mmol, 4 equiv) in tetrahydrofuran (30 mL) and water (1 mL). The reaction mixture was heated at 70 °C for 12 hours, cooled to room temperature and the organics extracted with ethyl acetate (25 mL x 2), dried ($MgSO_4$), filtered and the solvent removed *in vacuo*. The crude product was purified by flash column chromatography (SiO_2, 50 g, biotage isolera, hexane/ethyl acetate – 100/0 to 95/5, 120 mL/min) to give 2-(2,5-difluorophenyl)-5-methylpyridine (1.40 g, 6.82 mmol, 68%) as a colourless oil.

TLC: R_f = 0.28 (9:1 hexane/ethyl acetate) [UV]

^{1}H NMR: (500 MHz, $CDCl_3$)

8.54 (d, *J* = 2.3 Hz, 1H), 7.78 – 7.66 (m, 2H), 7.55 (dd, *J* = 8.2, 2.3 Hz, 1H), 7.12 – 7.05 (m, 1H), 7.04 – 6.97 (m, 1H), 2.37 (s, 3H).

^{13}C NMR: (126 MHz, $CDCl_3$)

159.1 (dd, *J* = 244.0, 2.0 Hz), 156.5 (dd, *J* = 243.2, 2.0 Hz), 150.4, 159.5 (dd, *J* = 2.6, 2.0 Hz), 137.1, 132.7, 128.8 (dd, *J* = 14.2, 7.6 Hz), 123.9 (d, *J* = 10.5 Hz), 117.4 (dd, *J* = 26.5, 9.0 Hz), 117.0 (dd, *J* = 25.6, 4.2 Hz), 116.5 (dd, *J* = 24.3, 9.0 Hz), 18.3.

^{19}F NMR: (471 MHz, $CDCl_3$)

-118.8, -123.2.

MS: (HRMS - ESI$^+$)

Found 228.0600 ($C_{12}H_9NF_2Na$), requires 228.0595.

6-Phenylpyridine-3-carboxylic acid hydrochloride

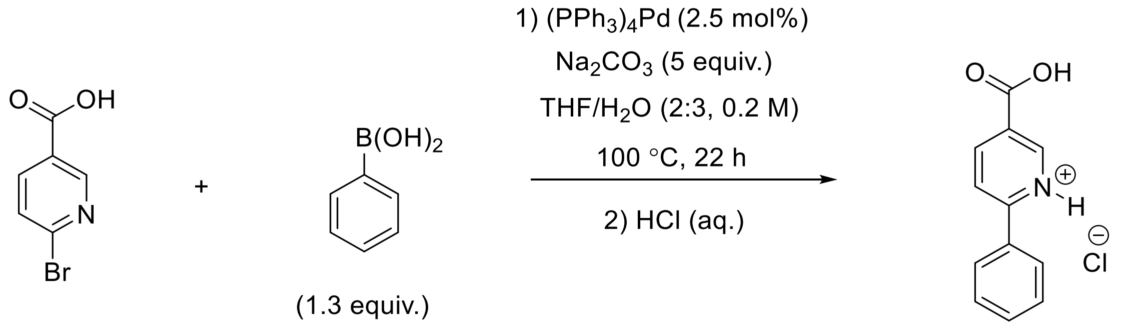

2-Bromonicotinic acid (6.0 g, 30 mmol), phenylboronic acid (4.8 g, 42 mmol) tetrakis(triphenylphosphine)palladium(0) (870 mg, 0.750 mmol, 2.5 mol%) and sodium carbonate (16.3 g, 154 mmol, 5.1 equiv.) were reacted (sealed-tube) in tetrahydrofuran (60 mL) and water (90 mL) at 100 °C for 22 hours. The reaction mixture was cooled to room temperature and the tetrahydrofuran removed *in vacuo* (500 to 210 mbar, 40 °C). The aqueous phase was acidified using aqueous hydrochloric acid (3 M, 200 mL) and the solid collected by vacuum filtration. The crude product was recrystallized using acetone to give 6-phenylpyridine-3-carboxylic acid hydrochloride (2.1 g, 8.9 mmol, 30%) as colourless needles.

mpt: 222-224 °C (acetone)

IR: (*neat*, cm^{-1})

1676.0 (m), 1671.7 (m), 1295.3 (m), 1269.5 (m), 746.4 (s).

^{1}H NMR: (400 MHz, d_6-acetone)

9.24 (d, *J* = 2.3, 1H), 8.41 (dd, *J* = 8.3, 2.2 Hz, 1H), 8.28 – 8.20 (m, 2H), 8.11 (d, *J* = 8.3, Hz, 1H), 7.61 – 7.46 (m, 3H).

^{13}C NMR: (101 MHz, d_6-acetone)

165.6, 160.3, 150.8, 138.2, 138.1, 129.9, 128.8, 127.2, 124.7, 119.7.

Isopropyl 6-phenylnicotinate

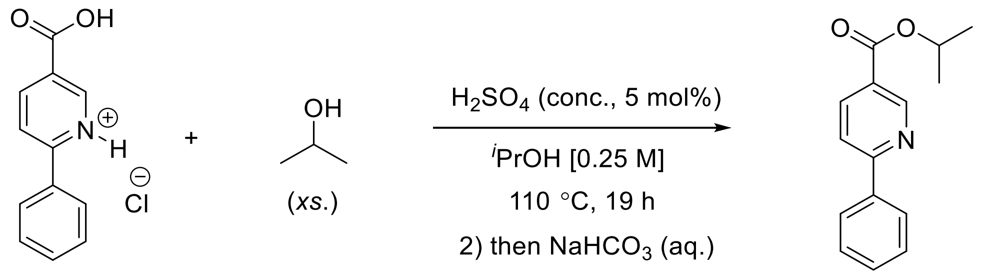

6-Phenylpyridine-3-carboxylic acid hydrochloride (600 mg, 2.54 mmol) was reacted with isopropanol (10 mL) and sulfuric acid (conc., approximately 20 µL, 5 mol%) at 110 °C (sealed-tube) for 19 h. The reaction mixture was cooled to room temperature, diluted with aqueous sodium hydrogen carbonate solution (60 mL, *caution*: gas evolution), extracted using dichloromethane (30 mL x 3), dried (Na_2SO_4), filtered and the solvent removed *in vacuo* (600 to 10 mbar, 40 °C). The crude material was purified by flash column chromatography (SiO_2, 10 g, biotage isolera, hexane/ethyl acetate – 100/0 to 85/15, 40 mL/min) to give isopropyl 6-phenylnicotinate (218.0 mg, 0.903 mmol, 36%) as a colourless amorphous solid.

mpt: 66-68 °C (ethyl acetate)

TLC: R_f = 0.8 (1:1 hexane/ethyl acetate) [UV]

IR: (*neat*, cm^{-1})

2977.8 (w), 1705.4 (s), 1594.6 (m), 1474.0 (w), 1111.8 (m), 744.2 (s).

^{1}H NMR: (400 MHz, $CDCl_3$)

9.28 (dd, *J* = 2.2, 0.9 Hz, 1H), 8.34 (dd, *J* = 8.3, 2.2 Hz, 1H), 8.09 – 8.02 (m, 2H), 7.80 (dd, *J* = 8.3, 0.9 Hz, 1H), 7.55 – 7.42 (m, 3H), 5.30 (hept., *J* = 6.2 Hz, 1H), 1.41 (d, *J* = 6.3 Hz, 6H).

^{13}C NMR: (101 MHz, $CDCl_3$)

164.9, 160.8, 151.0, 138.4, 137.8, 129.9, 128.9, 127.4, 124.9, 119.8, 69.0, 22.0.

(6-Phenylpyridin-3-yl)-piperidin-1-ylmethanone 2m

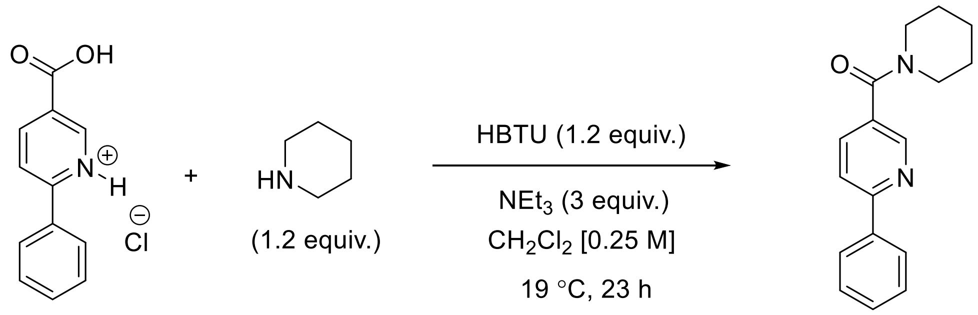

6-Phenylpyridine-3-carboxylic acid hydrochloride (600 mg, 2.54 mmol) was reacted with piperidine (300 μL, 3.05 mmol), 2-(1*H*-benzotriazol-1-yl)-1,1,3,3-tetramethyluronium hexafluorophosphate (1.16 g, 3.05 mmol), triethylamine (1.40 mL, 10 mmol) in dichloromethane (10 mL) at 19 °C for 23 hours. The solvent was removed *in vacuo* (500 to 50 mbar, 40 °C) and the crude reaction mixture purified by flash column chromatography (SiO_2, 25 g, biotage isolera, hexane/ethyl acetate – 100/0 to 60/40, 80 mL/min). The obtained solid was recrystallised from ethyl acetate (15 mL) to give *(6-phenylpyridin-3-yl)-piperidin-1-ylmethanone* (450 mg, 1.69 mmol, 67%) as colourless rods.

TLC: R_f = 0.16 (1:1 hexane/ethyl acetate) [UV]

IR: (*neat*, cm^{-1})

2933.7 (w), 2854.3 (w) 1609.2 (s), 1560.2 (w), 1370.9 (m), 835.8 (s).

^{1}H NMR: (400 MHz, $CDCl_3$)

8.73 (dd, *J* = 2.2, 1.0 Hz, 1H), 8.05 – 7.97 (m, 2H), 7.87 – 7.75 (m, 2H), 7.54 – 7.40 (m, 3H), 3.75 (br. s, 2H), 3.44 (br. s, 2H) 1.81 – 1.51 (br. m, 6H).

^{13}C NMR: (101 MHz, C_6D_6)

167.0, 157.7, 148.0, 138.8, 136.0, 130.7, 129.3, 128.7, 127.1, 119.4, 48.1 (br., $\Delta_{1/2}$ = 100 Hz), 43.0 (br., $\Delta_{1/2}$ = 91 Hz), 25.8 (br., $\Delta_{1/2}$ = 87 Hz), 24.3.

MS: (HRMS - ESI^+)

Found 289.1316 ($C_{17}H_{18}ON_2Na$), requires 289.1311.

(*S*)-4-{2-[(*tert*-Butoxycarbonyl)amino]-3-methoxy-3-oxopropyl}phenyl 6-phenylnicotinate 2p

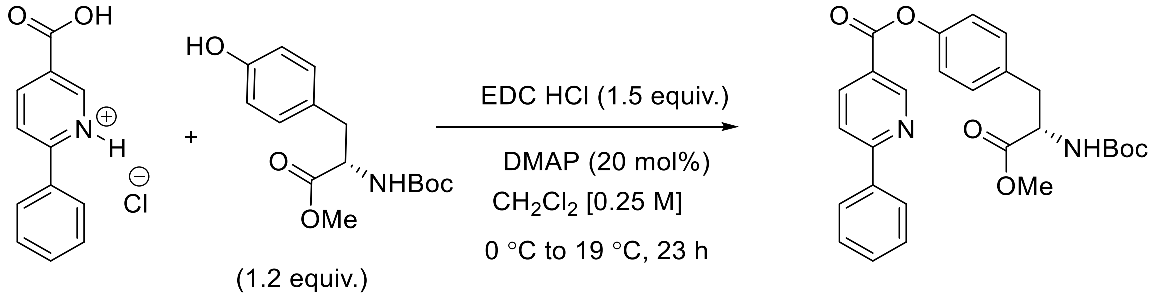

6-Phenylpyridine-3-carboxylic acid hydrochloride (500 mg, 2.20 mmol), *N*-(*tert*-butoxycarbonyl)-L-tyrosine methyl ester (750 mg, 2.54 mmol), *N,N*-dimethylaminopyridine (54.0 mg, 0.44 mmol) and *N*-ethyl-*N′*-(3-dimethylaminopropyl)carbodiimide hydrochloride (630 mg, 3.3 mmol) were reacted in dichloromethane (9 mL) at an initial temperature of 0 °C that was allowed to warm to 19 °C (over approximately 1 h), the reaction mixture was then stirred for a further 18 hours. Saturated aqueous sodium hydrogen carbonate (30 mL) was added to the reaction mixture and the products extracted using dichlormethane (50 mL x 3), dried (Na_2SO_4), filtered and the solvent removed *in vacuo* (500 to 10 mbar, 40 °C). The crude reaction mixture was purified by flash column chromatography (SiO_2, 25 g, biotage isolera, hexane/ethyl acetate – 100/0 to 60/40, 80 mL/min) and the obtained solid recrystallised from ethyl acetate (40 mL) to give *(S)-4-{2-[(tert-butoxycarbonyl)amino]-3-methoxy-3-oxopropyl}phenyl 6-phenylnicotinate* (872.0 mg, 1.83 mmol, 83%) as colourless needles.

mpt: 138-140 °C (ethyl acetate)

TLC: R_f = 0.40 (1:1 hexane/ethyl acetate) [UV]

IR: (*neat*, cm^{-1})

1738.8 (w), 1726.7 (m), 1696.5 (s), 1363.2 (w), 1127.4 (s), 749.1 (s).

^{1}H NMR: (400 MHz, $CDCl_3$)

9.46 (dd, *J* = 2.3, 0.8 Hz, 1H), 8.51 (dd, *J* = 8.3, 2.2 Hz, 1H), 8.19 – 8.07 (m, 2H), 7.91 (d, *J* = 8.3, 1H), 7.63 – 7.42 (m, 3H), 7.27 – 7.16 (m, 4H), 5.04 (d, *J* = 8.2 Hz, 1H), 4.86 – 4.51 (m, 1H), 3.76 (s, 3H), 3.15 (qd, *J* = 13.9, 5.9 Hz, 2H), 1.46 (s, 9H).

^{13}C NMR: (101 MHz, $CDCl_3$)

172.2, 164.0, 161.5, 155.1, 151.5, 149.7, 138.4, 138.2, 134.1, 130.5, 130.2, 129.0, 127.5, 123.7, 121.7, 120.0, 80.1, 54.4, 52.3, 37.8, 28.3.

MS: (HRMS - ESI$^+$)

Found 499.1845 ($C_{27}H_{28}O_6N_2Na$), requires 499.1840.

2-[(1,1'-Biphenyl)-4-yl]-pyridine 2g

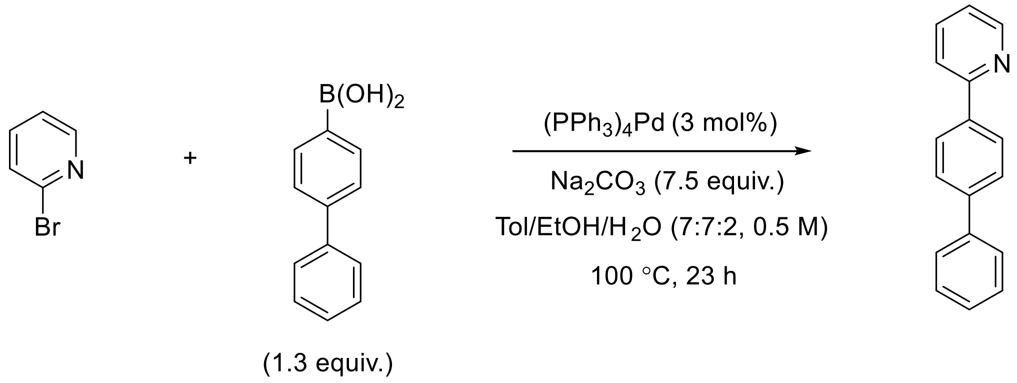

4-Biphenylboronic acid (3.35 g, 16.9 mmol), 2-bromopyridine (1.24 mL, 13.0 mmol), tetrakis(triphenylphosphine)palladium(0) (450 mg, 0.390 mmol, 3 mol%) and sodium carbonate (10.3 g, 97.5 mmol) were reacted in toluene (21 mL), ethanol (6 mL) and water (21 mL) at 100 °C for 23 hours. The reaction mixture was cooled to room temperature and filtered (Celite), eluting with dichloromethane (approximately 50 mL). The organics were extracted using dichloromethane (30 mL x 3), dried (Na_2SO_4), filtered, and the solvent removed *in vacuo* (500 to 30 mbar, 40 °C). The crude reaction mixture was purified by flash column chromatography (SiO_2, 30 g, biotage isolera, hexane/ethyl acetate – 100/0 to 90/10, 45 mL/min) to give a solid that was recrystallised from hexane/diethylether (50 mL, 95/5) to give 2-[(1,1'-biphenyl)-4-yl]-pyridine (785 mg, 3.39 mmol, 26%) as colourless plates.

mpt: 136-138 °C (hexane)

TLC: R_f = 0.16 (1:1 hexane/ethyl acetate) [UV]

^{1}H NMR: (400 MHz, $CDCl_3$)

8.72 (dt, *J* = 4.7, 1.4 Hz, 1H), 8.14 – 8.04 (m, 2H), 7.81 – 7.76 (m, 2H), 7.76 – 7.70 (m, 2H), 7.70 – 7.63 (m, 2H), 7.51 – 7.43 (m, 2H), 7.41 – 7.34 (m, 1H), 7.26 – 7.21 (m, 1H).

^{13}C NMR: (101 MHz, $CDCl_3$)

157.1, 149.8, 141.7, 140.6, 138.3, 136.8, 128.8, 127.5, 127.5, 127.3, 127.1, 122.1, 120.5.

Data were in accordance with those previously reported[1].

2-(2-Fluoro-4-methylphenyl)-4,5-dimethylpyridine

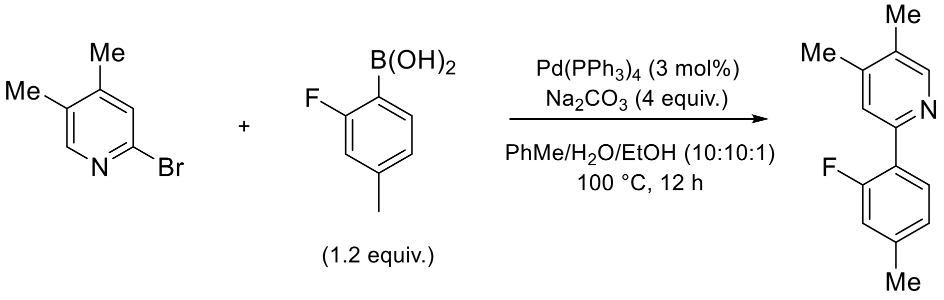

2-Bromo-4,5-dimethylpyridine (100.0 mg, 0.537 mmol) was reacted with (2-fluoro-4-methylphenyl) boronic acid (91.0 mg, 0.590, 1.1 equiv.) in the presence of tetrakis(triphenylphosphine)palladium(0) (17.3 mg, 15.0 μmol, 3 mol%) and sodium carbonate (224 mg, 2.12 mmol, 4.0 equiv.) in toluene (500 μL), water (500 μL) and ethanol (100 μL) at 100 °C for 12 hours. The reaction mixture was cooled to room temperature, water added and the organics extracted with ethyl acetate (25 mL x 2), dried ($MgSO_4$), filtered and the solvent removed *in vacuo*. The product was purified by flash column chromatography (SiO_2, manual column, hexane/ethyl acetate – 90/100 to 85/15) to give 2-(2-fluoro-4-methylphenyl)-4,5-dimethylpyridine (110 mg, 0.511 mmol, 95%) as a colourless amorphous solid.

TLC: R_f = 0.38 (85:15 hexane/ethyl acetate) [UV]

^{1}H NMR: (500 MHz, $CDCl_3$)

8.41 (s, 1H), 7.82 (t, *J* = 8.1 Hz, 1H), 7.53 (d, *J* = 2.3 Hz, 1H), 7.05 (dd, *J* = 8.0, 1.6 Hz, 1H), 6.95 (d, *J* = 12.3 Hz, 1H), 2.38 (s, 3H), 2.31 (s, 3H), 2.28 (s, 3H).

^{13}C NMR: (126 MHz, $CDCl_3$)

160.3 (d, *J* = 248.5 Hz), 151.4 (d, *J* = 2.4 Hz), 150.0, 145.9, 140.7 (d, *J* = 8.3 Hz), 130.9, 130.6 (d, *J* = 3.7 Hz), 125.3 (d, *J* = 3.1 Hz), 125.2 (d, *J* = 8.7 Hz), 124.8 (d, *J* = 11.8 Hz), 21.2 (d, *J* = 1.6 Hz), 19.5, 16.3.

^{19}F NMR: (471 MHz, $CDCl_3$)

-118.3.

MS: (HRMS - ESI^+)

Found 216.1194 ($C_{14}H_{15}NF$), requires 216.1189.

2-Phenyl-4-methoxypyrimidine

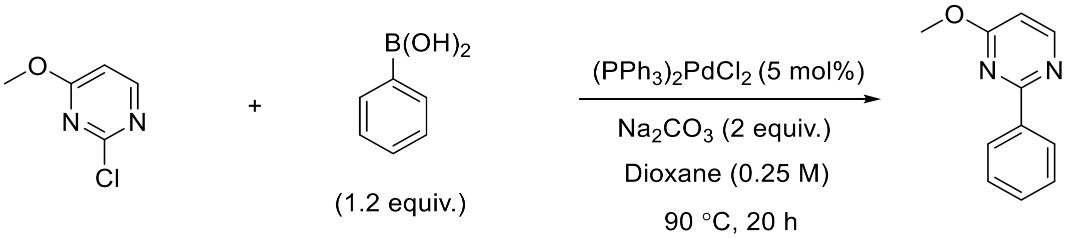

2-Chloro-4-methoxypyrimidine (2.17 g, 15.0 mmol), phenyl boronic acid (2.22 g, 18.2 mmol), bis(triphenylphosphine)palladium(II) dichloride (530 mg, 0.755 mmol, 5 mol%) and sodium carbonate (3.2 g, 30 mmol) were reacted in dioxane (60 mL) at 90 °C (sealed-tube) for 20 hours. The reaction mixture was cooled to room temperature, filtered (Celite) – eluting with dichloromethane (approximately 50 mL) and the solvent removed *in vacuo* (500 to 20 mbar, 40 °C). The crude reaction mixture was purified by flash column chromatography (SiO_2, 25 g, biotage isolera, hexane/ethyl acetate – 100/0 to 85/15, 80 mL/min) to give a solid that was recrystallised from hexane/diethyl ether (10 mL, 90/10) to give 2-phenyl-4-methoxypyrimidine (150 mg – first crop, 142 mg – second crop, 292 mg total, 1.568 mmol, 10%) as colourless needles.

mpt: 42-44 °C (hexane)

TLC: R_f = 0.63 (4:1) (hexane/ethyl acetate) [UV]

^{1}H NMR: (400 MHz, $CDCl_3$)

8.51 (d, *J* = 5.7 Hz, 1H), 8.48 – 8.41 (m, 2H), 7.53 – 7.43 (m, 3H), 6.64 (d, *J* = 5.7 Hz, 1H), 4.10 (s, 3H).

^{13}C NMR: (101 MHz, $CDCl_3$)

169.5, 164.4, 157.4, 137.6, 130.7, 128.5, 128.2, 106.2, 53.5.

Data were in accordance with those previously reported[2].

2-{4-[(Methoxymethoxy)methyl]phenyl}pyridine

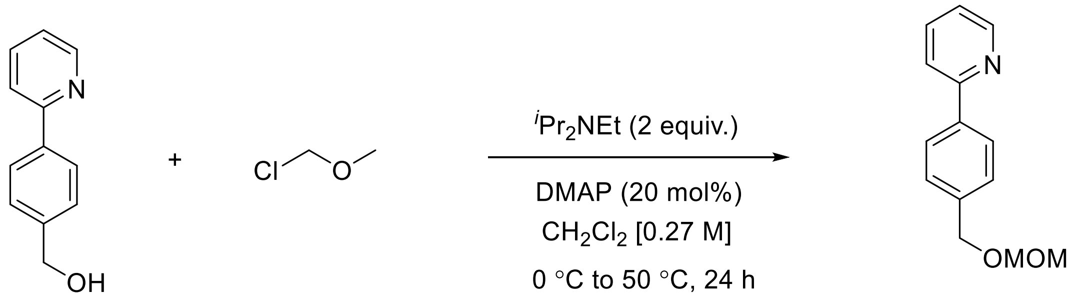

Chloromethyl methyl ether (410 µL, 5.40 mmol, 2 equiv.) was added dropwise (over approximately 1 minute) to a stirred solution of 2-(4-hydroxymethylphenyl)pyridine (500 mg, 2.70 mmol), *N,N*-diisopropylethylamine (940 µL, 5.40 mmol, 2 equiv.) and *N,N*-dimethylaminopyridine (66 mg, 0.540 mmol, 20 mol%) in dichloromethane (10 mL) at 0 °C. The mixture was reacted at 50 °C for 24 hours. The reaction mixture was cooled to room temperature and the solvent removed *in vacuo* (500 to 15 mbar, 40 °C). The crude reaction mixture was purified by flash column chromatography (SiO_2, 50 g, biotage isolera, hexane/ethyl acetate – 100/0 to 95/5, 45 mL/min) to give 2-{4-[(methoxymethoxy)methyl]phenyl}pyridine (610 mg, 2.66 mmol, 98%) as a colourless oil.

TLC: R_f = 0.28 (4:1) (hexane/ethyl acetate) [UV]

^{1}H NMR: (400 MHz, $CDCl_3$)

8.69 (dt, *J* = 4.7, 1.4 Hz, 1H), 8.04 – 7.95 (m, 2H), 7.79 – 7.68 (m, 2H), 7.52 – 7.43 (m, 2H), 7.23 (ddd, *J* = 6.5, 4.8, 2.2 Hz, 1H), 4.74 (s, 2H), 4.66 (s, 2H), 3.43 (s, 3H).

^{13}C NMR: (101 MHz, $CDCl_3$)

157.2, 149.7, 138.9, 138.7, 136.8, 128.2, 127.0, 122.1, 120.5, 95.8, 68.9, 55.4.

Data were in accordance with those previously reported[3].

4-Bromo-1-phenyl-1H-pyrazole

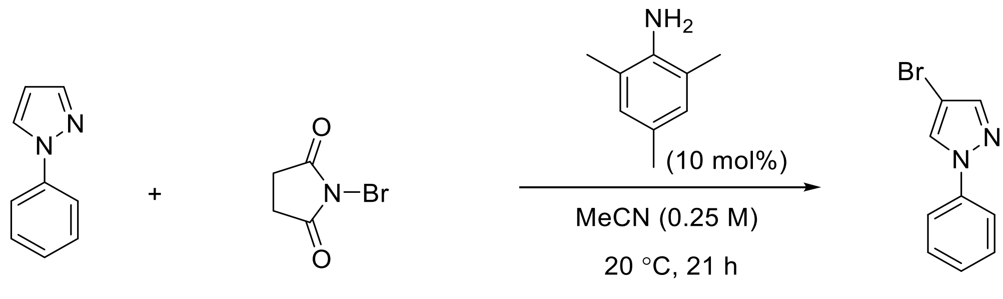

A mixture of 1-phenylpyrazole (1.73 g, 12.0 mmol) and 2,4,6-trimethylaniline (162 mg, 1.20 mmol, 10 mol%) was added dropwise (over approximately 2 minutes) to a stirred solution of *N*-bromosuccinimide (2.56 g, 14.4 mmol, 1.2 equiv.) – *caution: exotherm* – in acetonitrile (50 mL) at 20 °C. The reaction mixture was stirred for 21 hours, the solvent removed *in vacuo* (300 to 20 mbar, 40 °C) and the crude product purified by flash column chromatography (SiO_2, 25 g, biotage isolera, hexane/ethyl acetate – 100/0 to 94/6, 50 mL/min). The obtained solid was recrystallised from hexane (25 mL) to give 4-bromo-1-phenyl-1H-pyrazole (1.61 g, 4.20 mmol, 35%) as an off-white microcrystalline solid.

mpt: 78-80 °C (hexane)

IR: (*neat*, cm^{-1})

3113.3 (w), 1685.8 (w), 1333.3 (w), 949.1 (m), 751.4 (s).

TLC: R_f = 0.40 (4:1) (hexane/ethyl acetate) [UV]

^{1}H NMR: (500 MHz, $CDCl_3$)

7.94 (s, 1H), 7.68 (s, 1H), 7.66 – 7.61 (m, 2H), 7.51 – 7.43 (m, 2H), 7.36 – 7.27 (m, 1H).

^{13}C NMR: (101 MHz, $CDCl_3$)

141.5, 139.7, 129.6, 127.1, 127.0, 119.1, 95.6.

Data were in accordance with those previously reported[4].

1,4-Diphenyl-1H-pyrazole

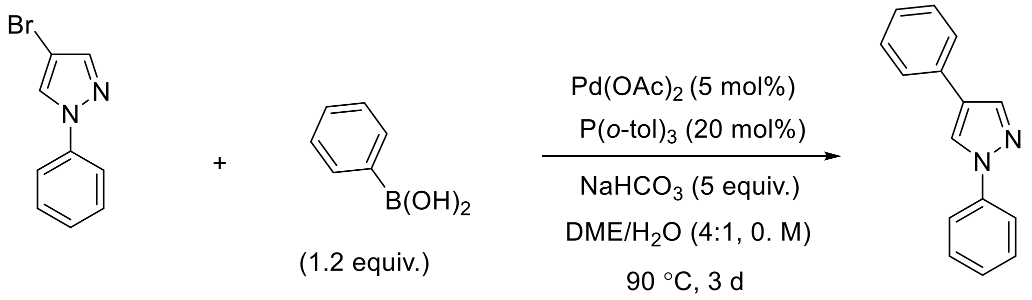

4-Bromo-1-phenyl-1H-pyrazole (500 mg, 2.24 mmol), phenylboronic acid (322 mg, 2.64 mmol, 1.2 equiv.), palladium(II) acetate (25.0 mg, 0.111 mmol, 5 mol%), tris(o-tolyl)phosphine (133 mg, 0.440 mmol, 20 mol%) and sodium hydrogen carbonate (924 mg, 11.0 mmol, 5 equiv.) were reacted in dimethoxyethane (20 mL) and water (5 mL) at 90 °C (sealed-tube) for 3 days. The reaction mixture was filtered (Celite) eluting with dichloromethane (50 mL), dried (Na_2SO_4), filtered and the solvent removed *in vacuo* (500 to 30 mbar, 40 °C). The crude reaction mixture was purified by flash column chromatography (SiO_2, 25 g, biotage isolera, hexane/ethyl acetate – 100/0 to 90/10, 40 mL/min) to give 1,4-diphenyl-1H-pyrazole (380 mg, 1.725 mmol, 78%) as a colourless amorphous solid.

mpt: 88-90 °C (hexane)

IR: (*neat*, cm^{-1})

3036.7 (w), 1594.7 (m), 1463.2 (m), 953.0 (m), 748.1 (s).

TLC: R_f = 0.40 (4:1) (hexane/ethyl acetate) [UV]

^{1}H NMR: (400 MHz, $CDCl_3$)

8.18 – 8.15 (m, 1H), 8.01 (s, 1H), 7.80 – 7.69 (m, 2H), 7.61 – 7.53 (m, 2H), 7.52 – 7.45 (m, 2H), 7.45 – 7.35 (m, 2H), 7.35 – 7.26 (m, 2H).

^{13}C NMR: (101 MHz, $CDCl_3$)

140.1, 138.8, 132.1, 129.5, 129.0, 126.9, 126.6, 125.7, 124.9, 123.3, 119.1.

Data were in accordance with those previously reported[5].

N-[2-(1H-Indol-3-yl)ethyl]-6-phenylnicotinamide

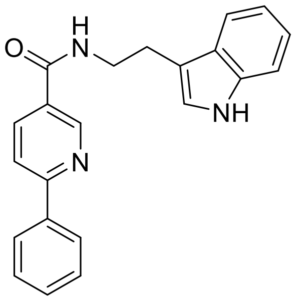

6-Phenylnicotinic acid hydrochloride (600 mg, 2.55 mmol), tryptamine (481 mg, 3.00 mmol, 1.20 equiv.), 2-(1*H*-benzotriazol-1-yl)-1,1,3,3-tetramethyluronium hexafluorophosphate (1.16 g, 3.05 mmol, 1.22 equiv.) and triethylamine (1.4 mL, 10.0 mmol, 4.00 equiv.) were reacted in dichloromethane (15 mL) at room temperature for 3 days. The solvent was removed *in vacuo* (500 to 300 mbar, 40 °C), the material filtered through a silica plug (SiO_2, 50 g, CH_2Cl_2/MeOH 95:5), the solvent removed *in vacuo* (500 to 50 mbar, 40 °C) to give a solid that was washed with cold dichloromethane (50 mL, approximately 5 °C) to give *N*-[2-(1*H*-indol-3-yl)ethyl]-6-phenylnicotinamide (295 mg, 0.864 mmol, 29%) as an off-while amorphous solid.

TLC: R_f = 0.73 (ethyl acetate) [UV]

mpt: 166 – 168 °C (ethyl acetate)

^{1}H NMR: (400 MHz, d_4-MeOD)

8.98 (d, *J* = 2.2 Hz, 1H), 8.21 (dd, *J* = 8.3, 2.4 Hz, 1H), 8.10 – 7.98 (m, 2H), 7.94 (d, *J* = 8.3 Hz, 1H), 7.62 (d, *J* = 7.9 Hz, 1H), 7.57 – 7.41 (m, 3H), 7.34 (d, *J* = 8.1 Hz, 1H), 7.16 – 7.04 (m, 2H), 7.00 (t, *J* = 7.4 Hz, 1H), 3.71 (t, *J* = 7.4 Hz, 2H), 3.11 (t, *J* = 7.3 Hz, 2H).

^{13}C NMR: (101 MHz, d_4-MeOD)

166.5, 159.7, 148.0, 138.1, 136.8, 136.2, 129.5, 128.8, 128.6, 127.5, 126.9, 122.1, 121.0, 120.3, 118.2, 117.9, 111.9, 110.9, 40.9, 24.8.

IR: (*neat*, cm^{-1})

3351.3 (m), 1626.1 (m), 1541.5 (m), 738.2 (s).

MS: (HRMS - ESI$^+$)

Found 364.1417 ($C_{22}H_{19}ON_3Na$), requires 364.1420.

(8R,9S,13S,14S)-13-Methyl-17-oxo-7,8,9,11,12,13,14,15,16,17-decahydro-6H-cyclopenta[a]phenanthren-3-yl 6-phenylnicotinate

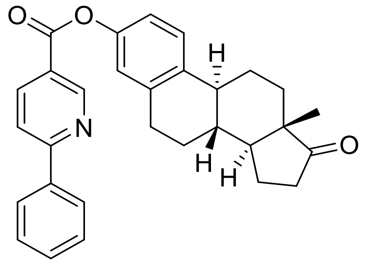

6-Phenylnicotinic acid hydrochloride (600 mg, 2.55 mmol), estrone (811 mg, 3.00 mmol, 1.2 equiv.), 4-dimethylaminopyridine (61.0 mg, 0.5 mmol, 20 mol%) and 1-ethyl-3-(3-dimethylaminopropyl)carbodiimide hydrochloride (720 mg, 3.756 mmol, 1.47 equiv.) were reacted in dichloromethane (10 mL) at 40 °C for 18 hours. The reaction was cooled to room temperature, sat. aqueous sodium hydrogen carbonate (60 mL) added, the organic products extracted using dichloromethane (3 x 50 mL), dried ($MgSO_4$) filtered and the solvent removed *in vacuo* (500 to 50 mbar, 40 °C). The solid product was washed with hot hexane/ethyl acetate (1:1) and the product collected by filtration to give (8R,9S,13S,14S)-13-methyl-17-oxo-7,8,9,11,12,13,14,15,16,17-decahydro-6H-cyclopenta[a]phenanthren-3-yl 6-phenylnicotinate (1.05 g, 2.32 mmol, 91%) as a pink amorphous solid.

TLC: R_f = 0.29 (dichloromethane) [UV]

mpt: 198 – 200 °C (ethyl acetate)

^{1}H NMR: (400 MHz, $CDCl_3$)

9.44 (dd, *J* = 2.3, 0.9 Hz, 1H), 8.49 (dd, *J* = 8.3, 2.3 Hz, 1H), 8.15 – 8.03 (m, 2H), 7.89 (dd, *J* = 8.3, 0.9 Hz, 1H), 7.59 – 7.43 (m, 3H), 7.36 (dd, *J* = 8.6, 1.1 Hz, 1H), 7.07 – 6.90 (m, 2H), 2.96 (dd, *J* = 9.2, 4.2 Hz, 2H), 2.58 – 2.27 (m, 3H), 2.22 – 1.95 (m, 4H), 1.68 – 1.48 (m, 6H), 0.94 (s, 3H).

^{13}C NMR: (101 MHz, $CDCl_3$)

220.8, 164.3, 161.5, 151.5, 148.5, 138.4, 138.3, 138.2, 137.8, 130.2, 129.0, 127.5, 126.6, 123.8, 121.6, 120.0, 118.8, 50.5, 48.0, 44.2, 38.0, 35.9, 31.6, 29.5, 26.4, 25.8, 21.6, 13.9.

IR: (*neat*, cm^{-1})

2930.0 (w), 1727.0 (s), 1685.3 (m), 1004.9 (m), 746.9 (s).

MS: (HRMS - ESI^+)

Found 474.2041 ($C_{30}H_{29}O_3NNa$), requires 474.2040.

(3S,8S,9S,10R,13R,14S,17R)-10,13-Dimethyl-17-[(*R*)-6-methylheptan-2-yl]-2,3,4,7,8,9,10,11,12,13,14,15,16,17-tetradecahydro-1H-cyclopenta[a]phenanthren-3-yl 6-phenylnicotinate

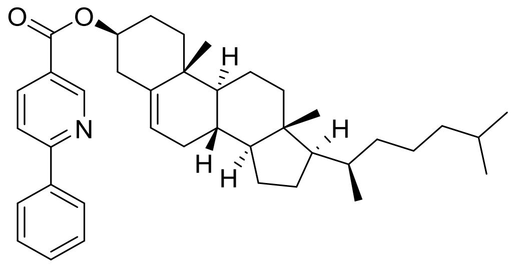

6-Phenylnicotinic acid hydrochloride (600 mg, 2.55 mmol), cholesterol (1.16 g, 3.00 mmol, 1.2 equiv.), 4-dimethylaminopyridine (61.0 mg, 0.500 mmol, 20 mol%) and 1-ethyl-3-(3-dimethylaminopropyl)carbodiimide hydrochloride (720 mg, 3.76 mmol, 1.50 equiv.) were added to cold dichloromethane (10 mL) at 0 °C. The reaction mixture was warmed to 19 °C and reacted for 3 days. Sat. aqueous sodium hydrogen carbonate (50 mL) was added and the organic products extracted using dichloromethane (3 x 50 mL), dried ($MgSO_4$), filtered and the solvent removed *in vacuo* (500 to 50 mbar, 40 °C). The product was purified by flash column chromatography (SiO_2, 25 g, biotage isolera, hexane/ethyl acetate – 100/0 to 90/10, 80 mL/min) to give (3S,8S,9S,10R,13R,14S,17R)-10,13-dimethyl-17-[(*R*)-6-methylheptan-2-yl]-2,3,4,7,8,9,10,11,12,13,14,15,16,17-tetradecahydro-1H-cyclopenta[a]phenanthren-3-yl 6-phenylnicotinate (500 mg, 0.881 mmol, 35%) as colourless plates.

TLC: R_f = 0.5 (4:1, hexane/ethyl acetate) [UV]

mpt: 188 – 190 °C (ethyl acetate)

^{1}H NMR: (500 MHz, $CDCl_3$)

9.28 (d, *J* = 2.2 Hz, 1H), 8.34 (dd, *J* = 8.3, 2.2 Hz, 1H), 8.06 (dd, *J* = 7.8, 1.6 Hz, 2H), 7.81 (d, *J* = 8.3 Hz, 1H), 7.59 – 7.39 (m, 3H), 5.50 – 5.37 (m, 1H), 4.95 – 4.85 (m, 1H), 2.49 (d, *J* = 8.0 Hz, 2H), 2.10 – 1.72 (m, 6H), 1.63 – 1.56 (m, 2H), 1.53 – 1.43 (m, 4H), 1.43 – 1.09 (m, 10H), 1.08 – 0.95 (m, 5H), 0.92 (d, *J* = 6.5 Hz, 3H), 0.87 (dd, *J* = 6.7, 2.3 Hz, 6H), 0.69 (s, 3H).

^{13}C NMR: (101 MHz, $CDCl_3$)

164.8, 160.8, 151.0, 139.5, 138.4, 137.9, 129.9, 128.9, 127.4, 124.8, 123.0, 119.8, 75.1, 56.7, 56.2, 50.1, 42.4, 39.8, 39.5, 38.2, 37.0, 36.7, 36.2, 35.8, 32.0, 31.9, 28.3, 28.0, 27.9, 24.3, 23.9, 22.8, 22.6, 21.1, 19.4, 18.7, 11.9.

IR: (*neat*, cm^{-1})

2934.8 (m), 1706.0 (s), 1595.5 (w), 1375.0 (m), 1284.5 (m), 1124.0 (m), 742.6 (m).

MS: (HRMS - ESI^+)

Found 568.4158 ($C_{39}H_{54}O_2N$), requires 568.4149.

1-Phenyl-5-(phenylthio)-*1H*-pyrazole

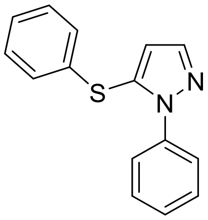

1-Phenyl-1*H*-pyrazole (720 mg, 5.00 mmol), diphenyl disulfide (2.20 g, 10.1 mmol, 2.0 equiv.), potassium *tert*-butoxide (1.12 g, 10.0 mmol) were heated at 130 °C in dimethylformamide (5 mL) for 40 hours. The reaction mixture was cooled to room temperature, sat. aqueous sodium hydrogen carbonate (50 mL) was added and the organic products extracted using dichloromethane (3 x 40 mL), dried ($MgSO_4$), filtered and the solvent removed *in vacuo* (500 to 300 mbar, 40 °C). The product was purified by flash column chromatography (SiO_2, 50 g, biotage isolera, hexane/ethyl acetate – 100/0 to 92/8, 100 mL/min) to give 1-phenyl-5-(phenylthio)-*1H*-pyrazole (230 mg, 0.91 mmol, 18%) as a yellow oil.

TLC: R_f = 0.4 (9:1, hexane/ethyl acetate) [UV]

^{1}H NMR: (400 MHz, $CDCl_3$)

7.74 (d, *J* = 1.9 Hz, 1H), 7.50 – 7.43 (m, 2H), 7.42 – 7.33 (m, 3H), 7.25 – 7.15 (m, 3H), 7.13 – 7.04 (m, 2H), 6.61 (d, *J* = 1.9 Hz, 1H).

^{13}C NMR: (126 MHz, $CDCl_3$)

140.5, 139.3, 135.2, 132.4, 129.2, 128.7, 128.5, 128.1, 126.8, 125.4, 114.7.

MS: (HRMS - ESI$^+$)

Found 275.0614 ($C_{15}H_{12}N_2NaS$), requires 275.0613.

Data were in accordance with those previously reported[6].

Synthesis of Losmapimod

6-(3-Fluorophenyl)-*N*-neopentylnicotinamide 2ac

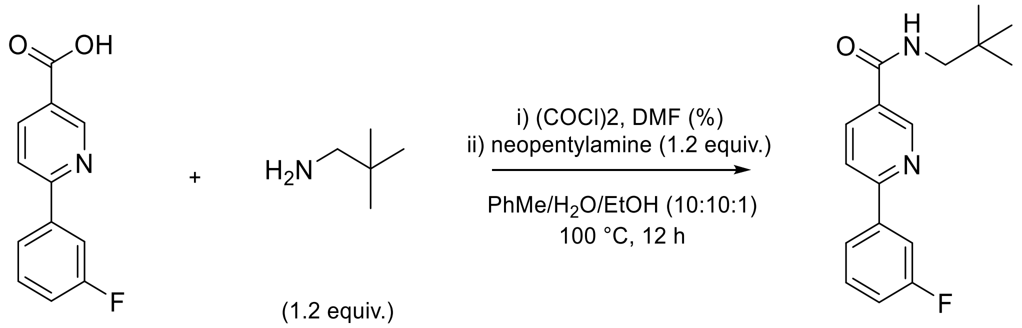

6-(3-Fluorophenyl)nicotinic acid (4.50 g, 20.7 mmol) was reacted with oxalyl chloride (3.99 g, 2.6 mL, 31.4 mmol, 1.52 equiv.) and *N*,*N*-dimethylformamide (2 drops, catalytic) in dichloromethane (138 mL) at 0 °C using ice bath and the reaction mixture was stirred for 4 hours at room temperature. After the effervescence was stopped, reaction mixture was concentrated *in-vacuo* and the acid chloride (pink solid) thus obtained was used without further purification in the next step.

In a 250 mL RBF containing acid chloride was added dichloromethane (150 mL) and cooled to 0 °C using ice bath and sequentially added neopentylamine (2.70 g, 3.6 mL, 31.0 mmol, 1.50 equiv.) and triethylamine (4.10 g, 5.76 mL, 40.5 mmol, 2.0 equiv.). The resulting reaction mixture stirred for 18 hours and monitored by TLC. The reaction mixture was quenched with aq. NH_4Cl (50 mL) and extracted with dichloromethane (3 x 50 mL). The combined organic layers were dried over $MgSO_4$, filtered and, concentrated *in vacuo* to give crude 6-(3-fluorophenyl)-*N*-neopentylnicotinamide. The crude was suspended again in dichloromethane (40 mL) and sonicated to dissolve all the solid and slowly added hexane (150 mL) under strong stirring to precipitate pure 6-(3-fluorophenyl)-*N*-neopentylnicotinamide (5.32 g, 18.58 mmol, 89%) as a colourless amorphous solid.

TLC: R_f = 0.42 (70:30 hexane/ethyl acetate) [UV]

Mpt: 142-146 °C (ethyl acetate)

IR: (*neat*, cm^{-1})

3272.4 (w), 2957.8 (w), 1643.8 (s), 1563.35 (s), 1436.3 (m), 772.7 (s).

^{1}H NMR: (400 MHz, d_6-Me_2SO)

9.09 (d, J = 2.3 Hz, 1H), 8.58 (t, J = 6.4 Hz, 1H), 8.30 (dd, J = 8.4, 2.3 Hz, 1H), 8.14 (d, J = 8.3 Hz, 1H), 8.07 – 7.89 (m, 2H), 7.57 (td, J = 8.0, 6.1 Hz, 1H), 7.32 (td, J = 8.5, 2.6 Hz, 1H), 3.15 (d, J = 6.3 Hz, 2H), 0.92 (s, 9H).

^{13}C NMR: (101 MHz, d_6-Me_2SO)

164.9, 162.7 (d, J = 243 Hz), 156.4, 156.3, 148.6, 140.3 (d, J = 7.7 Hz), 136.3, 130.9 (d, J = 8.2 Hz), 129.4, 122.9, 122.9, 120.0, 116.4 (d, J = 22.3 Hz), 113.5 (d, J = 23.5 Hz), 50.1, 32.6, 27.5.

^{19}F NMR: (471 MHz, d_6-Me_2SO)

-112.7.

MS: (HRMS - ESI$^+$)

Found 309.1385 ($C_{17}H_{19}ON_2FNa$), requires 309.1374.

6-{3-[Bis(trimethylsilyl)methyl]-5-fluorophenyl}-*N*-neopentylnicotinamide 6w

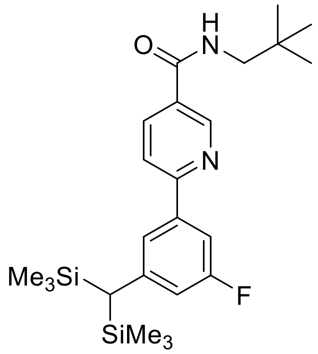

Following the modification of general procedure B, the reaction was setup in an argon filled glovebox using a 100 mL oven dried pressure tube, 6-(3-fluorophenyl)-*N*-neopentylnicotinamide (2.82 g, 10.0 mmol) was reacted with bis(trimethylsilyl)chloromethane (2.63 mL, 12.0 mmol, 1.2 equiv.) in the presence of $[(^tBuCN)_5Ru(H_2O)](BF_4)_2$ (708 mg, 1.00 mmol, 10 mol%), lithium carbonate (887 mg, 12.0 mmol, 1.2 equiv.), lithium cyclohexane carboxylate (150 mg, 1.10 mmol, 10 mol%), water (4.0 mL) and isopropanol (6.0 mL) at 100 °C for 18 hours. The crude reaction mixture was purified by flash column chromatography (SiO_2, 5–20% EtOAc in hexane) to give 6-{3-[bis(trimethylsilyl)methyl]-5-fluorophenyl}-*N*-neopentylnicotinamide (2.40 g, 5.40 mmol, 54%) as a colourless amorphous solid. (Note: during the chromatographic separation of the product, a green contaminant may appear in the fractions which can give overall green colour to the separated product. This can be avoided by running the column slowly or if the colour present, the product can be treated with activated charcoal in methanol at 50 °C. The presence of green colour however does not affect the purity of the product by ^{1}H NMR or affect subsequent reactions)

TLC: R_f = 0.36 (85:15 hexane/ethyl acetate) [UV]

Mpt: 112-114 °C (methanol)

^{1}H NMR: (400 MHz, $CDCl_3$)

9.02 (d, *J* = 2.3 Hz, 1H), 8.18 (dd, *J* = 8.3, 2.3 Hz, 1H), 7.73 (d, *J* = 8.2 Hz, 1H), 7.44 (dt, *J* = 10.0, 1.9 Hz, 1H), 7.38 (t, *J* = 1.6 Hz, 1H), 6.73 (dt, *J* = 10.2, 2.2 Hz, 1H), 6.29 (t, *J* = 6.3 Hz, 1H), 3.31 (d, *J* = 6.3 Hz, 2H), 0.99 (s, 9H), 0.06 (s, 18H).

^{13}C NMR: (101 MHz, $CDCl_3$)

165.5, 163.2 (d, *J* = 242.7 Hz), 158.8 (d, *J* = 3.0 Hz), 147.4, 146.6 (d, *J* = 8.3 Hz), 139.6 (d, *J* = 8.6 Hz), 136.0, 128.8, 123.0 (br., $\Delta\nu_{1/2}$ = 32 Hz), 120.0, 116.2 (br., $\Delta\nu_{1/2}$ = 50 Hz), 109.1 (d, *J* = 23.7 Hz), 50.9, 32.1, 30.1, 30.1, 27.2.

^{19}F NMR: (471 MHz, $CDCl_3$)

-113.6.

MS: (HRMS - ESI^+)

Found 467.2336 ($C_{24}H_{37}ON_2FNaSi_2$), requires 467.2321.

6-(3-Fluoro-5-formylphenyl)-*N*-neopentylnicotinamide

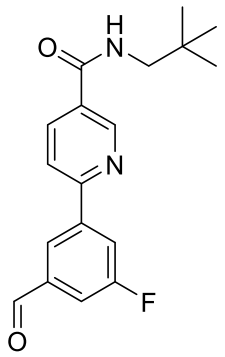

Ammonium ceric nitrate (1.20 g, 2.20 mmol, 4.89 equiv) was added to a stirred solution of 6-{3-[bis(trimethylsilyl)methyl]-5-fluorophenyl}-*N*-neopentylnicotinamide (200 mg, 0.450 mmol) in acetonitrile/water (30 mL, 5:1) at 0 °C. The reaction mixture was stirred for 30 minutes, warmed to room temperature, and stirred for a further 16 hours. Saturated aqueous ammonium chloride solution (30 mL) was added, and the organic products extracted using ethyl acetate (3 x 30 mL), dried ($MgSO_4$), filtered and the solvent removed *in vacuo* (250 to 15 mbar, 40 °C). The product was purified by flash column chromatography (SiO_2, 10 g, biotage isolera, hexane/ethyl acetate – 95/5 to 70/30, 40 mL/min) to give 6-(3-fluoro-5-formylphenyl)-*N*-neopentylnicotinamide (80.5 mg, 0.256 mmol, 57%) as a colourless oil.

TLC: R_f = 0.40 (60:40 hexane/ethyl acetate) [UV]

^{1}H NMR: (500 MHz, CD_2Cl_2)

10.04 (d, *J* = 1.7 Hz, 1H), 9.43 – 9.18 (m, 1H), 8.53 (dd, *J* = 8.3, 2.1 Hz, 1H), 8.31 (d, *J* = 1.5 Hz, 1H), 8.05 – 7.98 (m, 2H), 7.73 – 7.65 (m, 1H), 7.26 (t, *J* = 6.3 Hz, 1H), 3.29 (d, *J* = 6.4 Hz, 2H), 0.97 (s, 9H).

^{13}C NMR: (126 MHz, CD_2Cl_2)

190.6 (d, *J* = 2.1 Hz), 164.8, 164.4, 162.9, 155.0, 146.4, 140.4, 139.5 (d, *J* = 6.4 Hz), 138.8 (d, *J* = 7.5 Hz), 131.8, 125.2 (d, *J* = 2.7 Hz), 122.7, 120.5 (d, *J* = 24.2 Hz), 117.6 (d, *J* = 22.3 Hz), 51.6, 32.7, 27.4.

^{19}F NMR: (471 MHz, CD_2Cl_2)

-110.3.

MS: (HRMS - ESI$^+$)

Found 313.1363 ($C_{18}H_{18}O_2N_2F$), requires 313.1358.

3-Fluoro-5-[5-(neopentylcarbamoyl)pyridin-2-yl]benzoic acid

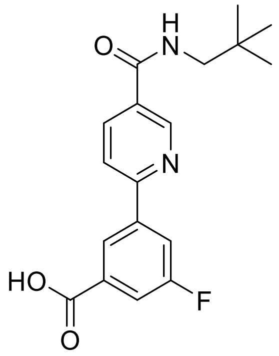

6-(3-Fluoro-5-formylphenyl)-*N*-neopentylnicotinamide (324 mg, 1.03 mmol) was stirred in a solution of THF/H_2O at room temperature and 2-methyl-2-butene (1.09 mL, 10.0 mmol, 10 equiv.), sodium dihydrogen phosphate (494 mg, 4.11 mmol, 4.0 equiv.) and sodium chlorite (373 mg, 4.11 mmol, 4.0 equiv.) was added sequentially to the reaction mixture. The reaction mixture was stirred for 3 h at room temperature and monitored by TLC. The reaction was quenched with aq. $NaHSO_3$ and extracted 3 times with EtOAc. The organic layer was dried over $MgSO_4$. After filtration and removal of solvent *in vacuo* the crude was dissolved again in minimum amount of EtOAc and added pentane until the precipitation of colourless solid. The solid thus obtained was filtered and washed with 10% EtOAc/pentane dried under vacuum to give 3-fluoro-5-[5-(neopentylcarbamoyl)pyridin-2-yl]benzoic acid (238 mg, 0.720 mmol, 70%) as off-white cuboids.

TLC: R_f = 0.17 (40:60 hexane/ethyl acetate) [UV]

Mpt: 138-140 °C (dichloromethane)

^{1}H NMR: (500 MHz, $(CD_3)_2SO$)

13.33 (br. s, 1H), 9.11 (s, 1H), 8.75 – 8.48 (m, 2H), 8.42 – 8.09 (m, 3H), 7.76 (d, *J* = 8.7 Hz, 1H), 3.15 (d, *J* = 6.3 Hz, 2H), 0.92 (s, 9H).

^{13}C NMR: (126 MHz, $(CD_3)_2SO$)

166.0, 164.8, 162.5 (d, *J* = 246.0 Hz), 155.4, 148.8, 140.6 (d, *J* = 7.9 Hz), 133.8 (d, *J* = 7.4 Hz), 129.7, 123.6, 120.2, 117.8 (d, *J* = 23.5 Hz), 116.7 (d, *J* = 22.9 Hz), 50.1, 27.5.

^{19}F NMR: (471 MHz, $(CD_3)_2SO$)

-112.0.

MS: (HRMS - ESI$^+$)

Found 329.1311 ($C_{18}H_{18}O_3N_2F$), requires 329.1307.

6-[3-(Cyclopropylcarbamoyl)-5-fluorophenyl]-*N*-neopentylnicotinamide 7

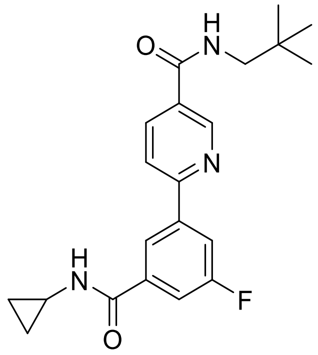

3-Fluoro-5-[5-(neopentylcarbamoyl)pyridin-2-yl]benzoic acid (340 mg, 1.03 mmol, 1.0 equiv.) was dissolved in dichloromethane (50 mL) in a 250 mL flame dried RBF and cyclopropyl amine (107 µL, 1.54 mmol, 1.5 equiv.), 1-ethyl-3-(3-dimethylaminopropyl) carbodiimide hydrochloride (293 mg, 1.52 mmol, 1.50 equiv.) and catalytic amount of DMAP was added to the stirred reaction mixture. The reaction mixture was stirred overnight (approximately 16 h). The reaction was quenched with water and extracted 3 times with dichloromethane. The organic layers were dried over $MgSO_4$, filtered, and evaporated *in vacuo* to yield the crude diamide. The crude was purified by flash column chromatography (SiO_2, 60% EtOAc/hexane) to give 6-[3-(cyclopropylcarbamoyl)-5-fluorophenyl]-*N*-neopentylnicotinamide (274 mg, 0.742 mmol, 72%) as off-white solid.

Mpt: 200-202 °C (methanol)

IR: (*neat*, cm^{-1})

3291, 2956, 1641, 1593, 1537, 1364, 1316, 1159, 892, 678.

^{1}H NMR: (400 MHz, $CDCl_3$)

9.01 (d, *J* = 2.3 Hz, 1H), 8.22 – 8.08 (m, 2H), 7.93 – 7.76 (m, 2H), 7.54 (dt, *J* = 8.6, 2.0 Hz, 1H), 6.54 (s, 1H), 6.32 (t, *J* = 6.4 Hz, 1H), 3.32 (d, *J* = 6.3 Hz, 2H), 2.93 (tq, *J* = 7.2, 3.6 Hz, 1H), 1.01 (s, 9H), 0.90 (td, *J* = 7.1, 5.3 Hz, 2H), 0.73 – 0.59 (m, 2H).

^{13}C NMR: (101 MHz, $CDCl_3$)

167.5, 165.6, 163.3 (d, *J* = 248.0 Hz), 157.3 (d, *J* = 2.5 Hz), 148.0, 140.9 (d, *J* = 7.6 Hz), 137.4 (d, *J* = 7.0 Hz), 136.3, 129.9, 121.0 (d, *J* = 2.7 Hz), 120.4, 117.1 (d, *J* = 23.3 Hz), 115.6 (d, *J* = 23.2 Hz), 51.3, 32.4, 27.5, 23.5, 6.9.

^{19}F NMR: (376 MHz, $CDCl_3$)

-111.14.

MS: (HRMS - ESI$^+$)

Found 369.1858 ($C_{21}H_{24}FN_3O_2$), requires 369.1853.

6-[5-(Cyclopropylcarbamoyl)-3-fluoro-2-methylphenyl]-*N*-neopentylnicotinamide Losmapimod 8

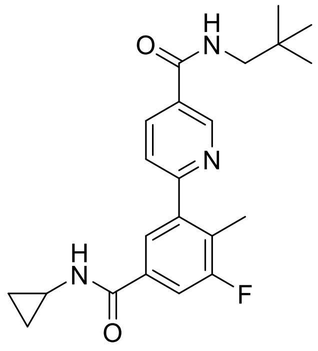

Following a modified general procedure A, the reaction was setup in an argon filled glovebox in a 10 mL ace pressure tube. 6-[3-(Cyclopropylcarbamoyl)-5-fluorophenyl]-N-neopentylnicotinamide (100 mg, 0.271 mmol) was reacted with trimethylsilylbromomethane (106 µL, 0.74 mmol, 2.0 equiv.) in the presence of $[(^tBuCN)_5Ru(H_2O)](BF_4)_2$ (26 mg, 0.03 mmol, 10 mol%), potassium carbonate (102 mg, 0.740 mmol, 2.0 equiv), potassium phenylphosphonate (19.0 mg, 0.081 mmol, 30 mol%), and anhydrous NMP (700 µL) at 100 °C for 48 hours to give 6-[5-(cyclopropylcarbamoyl)-3-fluoro-2-methylphenyl]-*N*-neopentylnicotinamide Losmapimod (54 mg, 0.141 mmol, 52%) as colourless amorphous solid.

TLC: R_f = 0.28 (70:30 hexane/EtOAc) [UV]

Mpt: 233 – 235 °C (EtOAc)

IR: (*neat*, cm^{-1})

3294, 2959, 1640, 1593, 1474, 1312, 731.

^{1}H NMR: (500 MHz, $CDCl_3$)

9.04 (d, *J* = 2.3 Hz, 1H), 8.18 (dd, *J* = 8.1, 2.3 Hz, 1H), 7.56 – 7.42 (m, 3H), 6.50 – 6.16 (m, 2H), 3.33 (d, *J* = 6.3 Hz, 2H), 2.88 (oct., *J* = 3.7, 1H), 2.28 (d, *J* = 2.4 Hz, 3H), 1.01 (s, 9H), 0.86 (td, *J* = 7.0, 5.3 Hz, 2H), 0.66 – 0.56 (m, 2H).

^{13}C NMR: (126 MHz, $CDCl_3$)

167.4, 165.6, 161.6 (d, *J* = 246.0 Hz), 147.6, 141.9, 135.7, 133.7 (d, *J* = 7.8 Hz), 129.4, 127.5 (d, *J* = 17.6 Hz), 124.2, 123.5, 114.3 (d, *J* = 26.0 Hz) 51.3, 32.4, 27.5, 23.3, 12.4, 12.3, 6.9.

^{19}F NMR: (471 MHz, $CDCl_3$)

-113.69 (d, *J* = 9.5 Hz).

MS: (HRMS - ESI$^+$)

Found 406.1901 ($C_{22}H_{26}N_3O_2FNa$), requires 406.1901.

6-{4-[Bis(trimethylsilyl)methyl]-6-fluoro-4'-methoxy-[1,1'-biphenyl]-2-yl}-*N*-neopentylnicotinamide

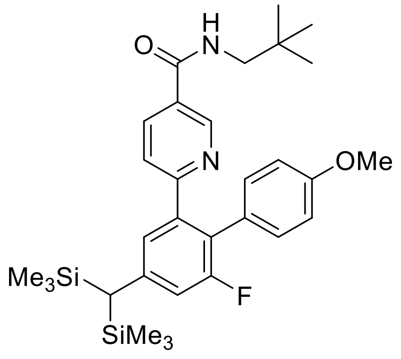

6-{3-[Bis(trimethylsilyl)methyl]-5-fluorophenyl}-*N*-neopentylnicotinamide (50.0 mg, 0.112 mmol) and 4-bromoanisole (14.0 µL, 0.112 mmol) were reacted in the presence of $[(^tBuCN)_5Ru(H_2O)](BF_4)_2$ (8.0 mg, 11 µmol, 10 mol%), potassium acetate (3.0 mg, 33 µmol, 30 mol%) and potassium carbonate (31.0 mg, 0.224 mmol) in *N*-methyl-2-pyrollidone (110 µL) at 50 °C for 18 hours. The crude reaction mixture was purified directly by flash column chromatography (SiO_2, 10 g, biotage isolera, hexane/ethyl acetate – 100/0 to 75/25, 40 mL/min) to give 6-{4-[bis(trimethylsilyl)methyl]-6-fluoro-4'-methoxy-[1,1'-biphenyl]-2-yl}-*N*-neopentylnicotinamide (41 mg, 74 µmol, 66%) as a colourless semi-solid.

TLC: R_f = 0.30 (80:20 hexane/ethyl acetate) [UV]

^{1}H NMR: (400 MHz, $CDCl_3$)

8.93 (d, *J* = 2.1 Hz, 1H), 7.80 (dd, *J* = 8.2, 2.3 Hz, 1H), 7.11 – 7.00 (m, 3H), 6.95 (dd, *J* = 8.2, 0.9 Hz, 1H), 6.86 – 6.70 (m, 3H), 6.15 (t, *J* = 6.3 Hz, 1H), 3.78 (s, 3H), 3.27 (d, *J* = 6.3 Hz, 2H), 1.63 (s, 1H), 0.98 (s, 9H), 0.09 (s, 18H).

^{13}C NMR: (126 MHz, $CDCl_3$)

165.9, 161.5 (d, *J* = 3.6 Hz), 159.9 (d, *J* = 244.0 Hz), 158.8, 147.4, 144.9 (d, *J* = 8.6 Hz), 140.4 (d, *J* = 3.8 Hz), 134.4, 132.1, 128.1, 126.3, 125.2, 123.0 (d, *J* = 16.6 Hz), 116.0 (d, *J* = 27.0 Hz), 113.7, 55.3, 51.1, 32.3, 30.0, 27.4, 0.4.

^{19}F NMR: (471 MHz, $CDCl_3$)

-117.0.

MS: (HRMS - ESI$^+$)

573.2748 ($C_{31}H_{43}O_2N_2FNaSi_2$), requires 573.2739.

Synthesis and characterisation of cyclometallated ruthenium(II) complexes

2-(2,5-Difluorophenyl)-5-methylpyridine ruthenium(II) tetraacetonitrile hexafluorophosphate

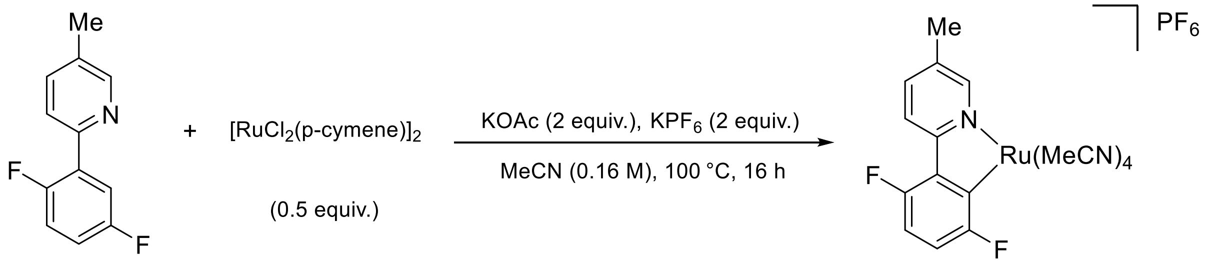

Dichloro(p-cymene)ruthenium(II) dimer (980.0 mg, 1.60 mmol, 0.50 equiv.), 2-(2,5-difluorophenyl)-5-methylpyridine (657.0 mg, 3.20 mmol), potassium acetate (628.0 mg, 6.4 mmol, 2.0 equiv.) and potassium hexafluorophosphate (1.28 g, 6.40 mmol, 2.0 equiv.) were heated in a sealed Ace pressure tube at 100 °C for 24 hours. The reaction mixture was cooled to room temperature and the product purified directly by flash column chromatography (neutral Al_2O_3, manual column, acetonitrile/dichloromethane – 1:1, N_2). The solvent was removed *in vacuo* to give 2-(2,5-difluorophenyl)-5-methylpyridine ruthenium(II) tetraacetonitrile hexafluorophosphate (1.60 g, 2.60 mmol, 82%) as a yellow amorphous solid. ***n.b.*** complex is air-sensitive and should be stored under an inert atmosphere.

^{1}H NMR: (400 MHz, d_3-CD_3CN)

8.87 (s, 1H), 8.15 (dd, *J* = 8.4, 2.3 Hz, 1H), 7.72 – 7.58 (m, 1H), 6.78 (ddd, *J* = 8.6, 7.1, 3.9 Hz, 1H), 6.67 (ddd, *J* = 11.5, 8.7, 3.8 Hz, 1H), 2.46 (s, 3H), 2.43 (s, 3H), 2.08 (s, 6H), 1.99 (s, 3H).

^{13}C NMR: (126 MHz, d_3-CD_3CN)

169.5 (d, *J* = 225.0 Hz), 166.6 (d, *J* = 48.6 Hz), 163.3 (d, *J* = 8.0 Hz), 157.5 (d, *J* = 250.0 Hz), 153.1, 137.9, 136.3 (dd, *J* = 19.0, 5.1 Hz), 132.6, 123.8, 122.6 (d, *J* = 21.6 Hz), 122.3, 121.8, 114.7 (dd, *J* = 35.4, 11.3 Hz), 109.5 (dd, *J* = 27.4, 8.9 Hz), 17.8, 3.6, 3.5, 1.4.

^{19}F NMR: (471 MHz, d_3-CD_3CN)

-72.2. -73.7, -104.8, -122.0.

2-{3-[Bis(trimethylsilyl)methyl]-2,5-difluorophenyl}-5-methylpyridine ruthenium(II) tetraacetonitrile hexafluorophosphate

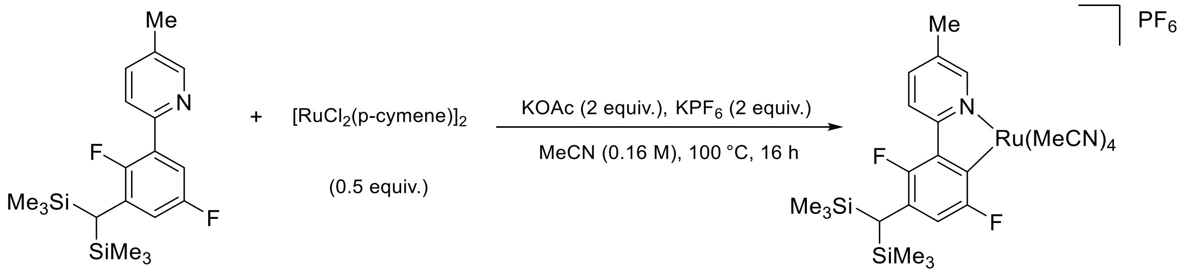

Dichloro(p-cymene)ruthenium(II) dimer (83.0 mg, 0.130 mmol, 0.5 equiv.), 2-{3-[bis(trimethylsilyl)methyl]-2,5-difluorophenyl}-5-methylpyridine (100.0 mg, 0.270 mmol), potassium acetate (53.0 mg, 0.530 mmol, 2.0 equiv.) and potassium hexafluorophosphate (99.0 mg, 0.530 mmol, 2.0 equiv.) were heated in a sealed Ace pressure tube at 100 °C for 24 hours. The reaction mixture was cooled to room temperature and the product purified directly by flash column chromatography (neutral Al_2O_3, manual column, acetonitrile/dichloromethane – 1:1, N_2). The solvent was concentrated *in vacuo* and diethylether (50 mL) added to precipitate 2-{3-[bis(trimethylsilyl)methyl]-2,5-difluorophenyl}-5-methylpyridine ruthenium(II) tetraacetonitrile hexafluorophosphate (132 mg, 0.171 mmol, 63%) as a yellow amorphous solid. ***n.b.*** complex is air-sensitive and should be stored under an inert atmosphere.

^{1}H NMR: (400 MHz, d_3-CD_3CN)

8.74 (s, 1H), 8.03 (dd, J = 8.6, 2.5 Hz, 1H), 7.60 – 7.46 (m, 1H), 6.54 – 6.27 (m, 1H), 2.35 (s, 3H), 2.32 (s, 3H), 1.98 (s, 6H), 1.89 (s, 3H), 0.00 (s, 18H).

^{13}C NMR: (101 MHz, d_3-CD_3CN)

169.0 (d, J = 225.0 Hz), 164.2 (d, J = 7.6 Hz), 158.2, 153.6, 138.3, 136.5, 132.7, 124.3, 123.1 (d, J = 23.4 Hz), 122.6, 115.6 (d, J = 32.8 Hz), 18.6, 18.2, 4.0, 3.9, 0.4.

^{19}F NMR: (471 MHz, d_3-CD_3CN)

-72.0, -74.0, -104.8, -126.2.

Bis[2-(2,5-Difluorophenyl)-5-methylpyridine] ruthenium(II) bisacetonitrile

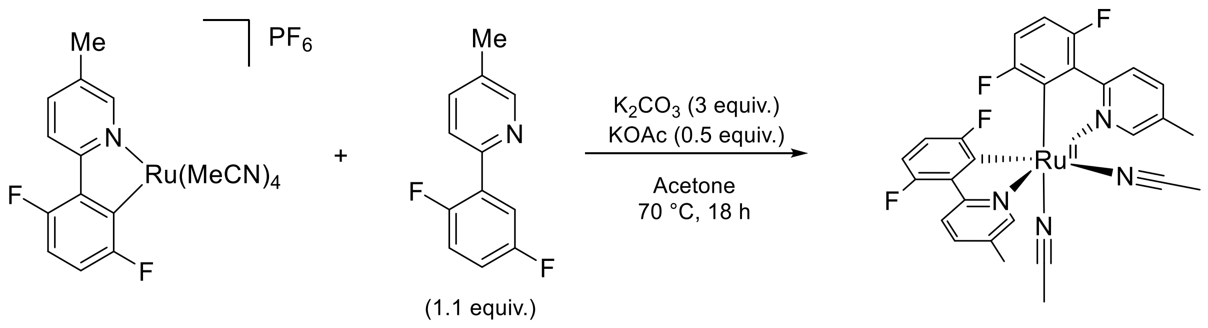

2-(2,5-Difluorophenyl)-5-methylpyridine ruthenium(II) tetraacetonitrile hexafluorophosphate (123.0 mg, 0.200 mmol), 2-(2,5-difluorophenyl)-5-methylpyridine (45.0 mg, 0.220 mmol, 1.1 equiv.), potassium carbonate (82.8 mg, 0.600 mmol, 3.0 equiv.) and potassium acetate (14.0 mg, 0.100 mmol, 0.5 equiv.) were stirred in acetone at 70 ºC for 18 hours. The reaction mixture was cooled to room temperature and filtered (2.5 µm PTFE filter), the solvent concentrated *in vacuo* and pentane (20 mL) added slowly whilst stirring until a solid precipitated. The precipitate was collected by filtration and washed with pentane to give bis[2-(2,5-difluorophenyl)-5-methylpyridine] ruthenium(II) bisacetonitrile (42.0 mg, 0.071 mmol, 36%) as an amorphous red solid. ***n.b.*** complex is air-sensitive and should be synthesised and stored in an argon-purified glovebox.

^{1}H NMR: (500 MHz, *d*$_6$-$(CD_3)_2CO$)

9.06 (s, 2H), 8.01 (d, *J* = 8.5 Hz, 2H), 7.43 (d, *J* = 8.4 Hz, 2H), 6.22 – 6.09 (m, 2H), 6.09 – 5.94 (m, 2H), 2.36 (s, 6H).

^{13}C NMR: (176 MHz, *d*$_6$-$(CD_3)_2CO$)

177.2 (d, *J* = 51.4 Hz), 163.6, 158.0 (d, *J* = 247.6 Hz), 152.2, 136.6, 133.9, 129.8, 121.6 (d, *J* = 21.7 Hz), 112.4 (d, *J* = 33.5 Hz), 105.8, 69.2, 18.3. (2 x resonances not observed and acetone/acetonitrile exchange observed).

^{19}F{^{1}H} NMR: (471 MHz, *d*$_6$-$(CD_3)_2CO$)

-105.23 (dq, *J* = 24.0, 4.2 Hz), -121.86 (dd, J = 23.0, 12.0 Hz).

General procedures for ruthenium-catalysed *ortho*- and *meta*-silylmethylation

General procedure A – *ortho*-selective silylmethylation: Arene (0.250 mmol), trimethylsilylbromomethane (0.250 mmol, 1.0 equiv.), $[(^{t}BuCN)_5Ru(H_2O)](BF_4)_2$ (9.0 mg, 13 µmol, 5 mol%), potassium carbonate (69.0 mg, 0.500 mmol, 2.0 equiv.), potassium phenylphosphonate (17.6 mg, 75 µmol, 30 mol%) and sodium iodide (37.5 mg, 0.250 mmol, 1.0 equiv.) were added to a vial that was sealed (crimp capped) and purged for approximately 40 seconds with N_2. Anhydrous tetrahydrofuran (500 µL) was added using a syringe and the reaction mixture heated at 80 °C for 18 hours. The reaction mixture was cooled to room temperature, the cap removed, and the solvent removed *in vacuo* before direct purification by flash column chromatography.

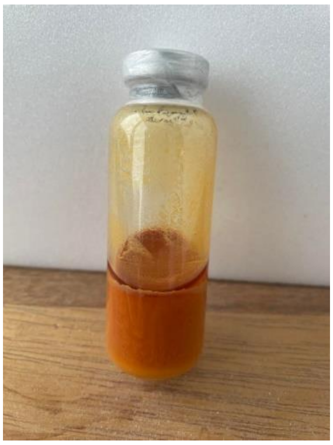

Supplementary Figure 46: Appearance post-reaction, after 18 hours of heating (substrate **2a** to **4a**).

General procedure B – *meta*-selective silylmethylation: Arene (0.250 mmol), bis(trimethylsilyl)chloromethane (58.5 mg, 0.300 mmol, 1.2 equiv.), $[(^{t}BuCN)_5Ru(H_2O)](BF_4)_2$ (13 µmol, 5 mol%), lithium carbonate (22.2 mg, 0.300 mmol, 1.2 equiv.) and lithium cyclohexane carboxylate (3.4 mg, 25 µmol, 10 mol%) were added to a vial that was sealed (crimp capped) and purged for approximately 40 seconds with N_2. Water (0.1 mL) and isopropanol (0.15 mL) were injected using a syringe and reaction mixture heated at 100 °C for 4 hours. The reaction mixture was cooled to room temperature, the cap removed, and the solvent removed *in vacuo* before direct purification by flash column chromatography.

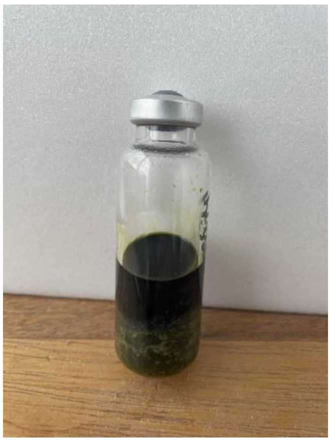

Supplementary Figure 47: Appearance post-reaction, after 4 hours of heating (substrate **2a** to **6a**).

Reactivity in the absence of sodium iodide

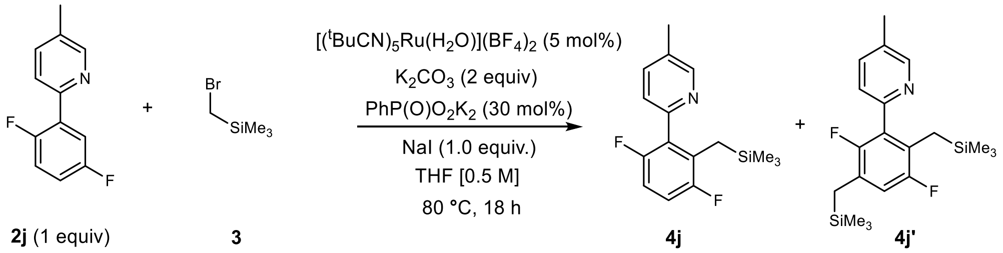

2j (1 equiv) **3** (1.0 equiv) **4j** **4j'**

	4j	4j'
with NaI:	86%	0%
without NaI:	66%	17%

Following general procedure A, 2-(2,5-difluorophenyl)-5-methylpyridine (103.0 mg, 0.500 mmol) was reacted with trimethylsilylbromomethane (72 μL, 0.250 mmol, 1.0 equiv) in the presence of $[(^tBuCN)_5Ru(H_2O)](BF_4)_2$ (18.0 mg, 25.0 μmol, 5 mol%), potassium carbonate (138.0 mg, 1.00 mmol, 2.0 equiv), potassium phenylphosphonate (35.2 mg, 0.150 mmol, 30 mol%), sodium iodide (37.5 mg, 0.250 mmol, 1.0 equiv) and anhydrous benzene (1.0 mL) at 80 °C for 18 hours.

In the reaction performed without sodium iodide, the same above stoichiometry was used without including NaI.

The crude reaction mixture was purified by flash column chromatography (SiO_2, 2 to 15% EtOAc/Hexane) to give 2-{3,6-difluoro-2-[(trimethylsilyl)methyl]phenyl}-5-methylpyridine (126.0 mg, 0.430 mmol, 86%) with NaI and (97.0 mg, 0.330 mmol, 66%) without NaI and 2-(2,5-difluoro-3,6-bis((trimethylsilyl)methyl)phenyl)-5-methylpyridine (32.0 mg, 85.0 μmol, 17%) in the reaction without NaI as a colourless oil.

Data for *2-{3,6-difluoro-2-[(trimethylsilyl)methyl]phenyl}-5-methylpyridine:*

TLC: R_f = 0.48 (85:15 hexane/EtOAc) [UV]

^{1}H NMR: (400 MHz, $CDCl_3$)

8.74 (d, *J* = 2.3 Hz, 1H), 7.75 (dd, *J* = 7.9, 2.3 Hz, 1H), 7.49 – 7.41 (m, 1H), 7.15 (td, *J* = 9.0, 4.5 Hz, 1H), 7.00 (td, *J* = 8.9, 4.3 Hz, 1H), 2.58 (s, 3H), 2.42 (d, *J* = 3.5 Hz, 2H), 0.04 (d, *J* = 5.6 Hz, 9H).

^{13}C NMR: (101 MHz, $CDCl_3$)

158.8 (dd, *J* = 17.0, 2.3 Hz), 156.5 (dd, *J* = 19.1, 2.3 Hz), 151.8 (d, *J* = 2.3 Hz), 151.1, 137.8, 133.2, 130.9 (dd, *J* = 19.3, 2.7 Hz), 129.1 (dd, *J* = 17.3, 4.9 Hz),

127.0 (d, J = 2.4 Hz), 116.3 (dd, J = 26.1, 9.5 Hz), 112.7 (dd, J = 25.9, 9.0 Hz), 19.5, 17.5 (app. t, J = 2.4 Hz), 0.01.

^{19}F NMR: (376 MHz, $CDCl_3$)

-119.2, -121.5.

MS: (HRMS - ESI$^+$)

Found 313.1146 ($C_{16}H_{19}NF_2SiNa$), requires 314.1147.

Data for *2-(2,5-difluoro-3,6-bis((trimethylsilyl)methyl)phenyl)-5-methylpyridine:*

TLC: R_f = 0.57 (85:15 hexane/EtOAc) [UV]

^{1}H NMR: (400 MHz, $CDCl_3$)

8.54 (d, J = 2.3 Hz, 1H), 7.56 (dd, J = 8.0, 2.3 Hz, 1H), 7.22 (dd, J = 7.8, 2.2 Hz, 1H), 6.69 (dd, J = 10.2, 6.5 Hz, 1H), 2.39 (s, 3H), 2.16 (d, J = 3.3 Hz, 2H), 2.03 (d, J = 2.2 Hz, 2H), -0.20 (s, 9H).

^{13}C NMR: (101 MHz, $CDCl_3$)

157.8 (d, J = 255 Hz), 155.5 (d, J = 254 Hz), 153.0, 151.6, 138.3, 133.6, 129.2 (dd, J = 18.0, 6.1 Hz), 127.6, 126.2 (d, J = 21.0 Hz), 125.9 (dd, J = 22.7, 8.5 Hz), 117.2 (dd, J = 26.2, 5.8 Hz), 21.3, 20.1, 17.4, 0.6, 0.0.

^{19}F NMR: (376 MHz, $CDCl_3$)

-120.4, -125.4.

MS: (HRMS - ESI$^+$)

Found 400.1697 ($C_{20}H_{29}NF_2Si_2Na$), requires 400.1699.

Ruthenium-catalysed *ortho*-silylmethylation

2-{2-[(Trimethylsilyl)methyl]phenyl}pyridine 4a

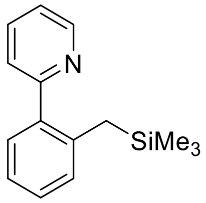

Following general procedure A, 2-phenylpyridine (39.0 mg, 0.250 mmol) was reacted with trimethylsilylbromomethane (41.8 mg, 0.250 mmol, 1.0 equiv.) in the presence of $[(^{t}BuCN)_5Ru(H_2O)](BF_4)_2$ (9.0 mg, 13 μmol, 5 mol%), potassium carbonate (69.0 mg, 0.500 mmol, 2.0 equiv.), potassium phenylphosphonate (17.6 mg, 75 μmol, 30 mol%), sodium iodide (37.5 mg, 0.250 mmol, 1 equiv.) and anhydrous tetrahydrofuran (500 μL) at 80 °C for 18 hours. The crude reaction mixture was purified by flash column chromatography (SiO_2, 25 g, biotage isolera, hexane/ethyl acetate – 100/0 to 92/8, 80 mL/min) to give 2-{2-[(trimethylsilyl)methyl]phenyl}pyridine (56.0 mg, 0.232 mmol, 93%) as a colourless oil.

TLC: R_f = 0.40 (9:1, hexane/ethyl acetate) [UV]

^{1}H NMR: (400 MHz, $CDCl_3$)

8.68 (ddd, *J* = 4.9, 1.8, 0.9 Hz, 1H), 7.73 (td, *J* = 7.7, 1.9 Hz, 1H), 7.40 (dt, *J* = 7.8, 1.1 Hz, 1H), 7.32 (dd, *J* = 7.5, 1.6 Hz, 1H), 7.26 – 7.08 (m, 4H), 2.44 (s, 2H), -0.22 (s, 9H).

^{13}C NMR: (101 MHz, $CDCl_3$)

161.3, 149.4, 139.3, 139.3, 136.6, 130.6, 130.4, 128.5, 125.0, 124.7, 121.9, 23.9, -1.0.

MS: (HRMS - ESI$^+$)

Found 242.1353 ($C_{15}H_{20}NSi$), requires 242.1360.

{4-(Pyridin-2-yl)-3-[(trimethylsilyl)methyl]phenyl}methanol 4b

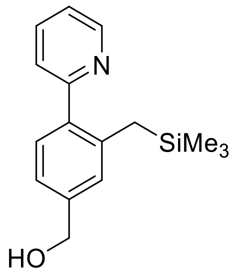

Following general procedure A, [4-(pyridin-2-yl)phenyl]methanol (46.3 mg, 0.250 mmol) was reacted with trimethylsilylbromomethane (41.7 mg, 0.250 mmol, 1.0 equiv.) in the presence of $[(^tBuCN)_5Ru(H_2O)](BF_4)_2$ (9.0 mg, 13 µmol, 5 mol%), potassium carbonate (69.0 mg, 0.500 mmol, 2.0 equiv.), potassium phenylphosphonate (17.6 mg, 75 µmol, 30 mol%), sodium iodide (37.5 mg, 0.250 mmol, 1.0 equiv.) and anhydrous tetrahydrofuran (500 µL) at 80 °C for 18 hours. The crude reaction mixture was purified by flash column chromatography (SiO_2, 25 g, biotage isolera, hexane/ethyl acetate – 100/0 to 60/40, 80 mL/min) to give {4-(pyridin-2-yl)-3-[(trimethylsilyl)methyl]phenyl}methanol (42.0 mg, 0.155 mmol, 62%) as a pale yellow oil.

TLC: R_f = 0.17 (7:3, hexane/ethyl acetate) [UV]

^{1}H NMR: (500 MHz, $CDCl_3$)

8.70 – 8.61 (m, 1H), 7.73 (td, J = 7.7, 1.8 Hz, 1H), 7.41 – 7.35 (m, 1H), 7.30 – 7.26 (m, 1H), 7.22 (ddd, J = 7.5, 4.9, 1.2 Hz, 1H), 7.14 – 7.09 (m, 1H), 7.08 (bs., 1H), 4.66 (s, 2H), 2.44 (s, 2H), -0.23 (s, 9H).

^{13}C NMR: (126 MHz, $CDCl_3$)

160.4, 148.8, 140.8, 139.0, 137.9, 136.1, 130.3, 128.1, 124.5, 122.6, 121.4, 64.9, 23.4, -1.6.

IR: (*neat*, cm^{-1})

3282.2 (br.), 2951.9 (w), 1609.8 (m), 1570.7 (m), 1246.7 (m), 847.2 (s).

MS: (HRMS - ESI^+)

Found 294.1278 ($C_{16}H_{21}NNaSi$), requires 294.1285.

2-{4-[((*tert*-Butyldimethylsilyl)oxy)methyl]-2-[(trimethylsilyl)methyl]phenyl}pyridine 4c

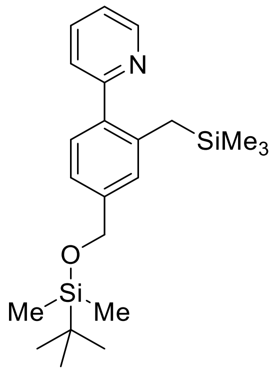

Following general procedure A, 2-{4-[((*tert*-butyldimethylsilyl)oxy)methyl]phenyl}pyridine (74.9 mg, 0.250 mmol, 1.0 equiv.) in the presence of $[(^{t}BuCN)_5Ru(H_2O)](BF_4)_2$ (9.0 mg, 13 µmol, 5 mol%), potassium carbonate (69.0 mg, 0.500 mmol, 2.0 equiv.), potassium phenylphosphonate (17.5 mg, 75 µmol, 30 mol%), sodium iodide (37.5 mg, 0.250 mmol, 1.0 equiv.) and anhydrous tetrahydrofuran (500 µL) at 80 °C for 18 hours. The crude reaction mixture was purified by flash column chromatography (SiO_2, 10 g, biotage isolera, hexane/ethyl acetate – 100/0 to 97/3, 40 mL/min) to give 2-{4-[((*tert*-butyldimethylsilyl)oxy)methyl]-2-[(trimethylsilyl)methyl]phenyl}pyridine (85.0 mg, 0.220 mmol, 88%) as a colourless oil.

TLC: R_f = 0.48 (4:1, hexane/ethyl acetate) [UV]

^{1}H NMR: (400 MHz, $CDCl_3$)

8.74 – 8.61 (m, 1H), 7.71 (td, *J* = 7.7, 1.9 Hz, 1H), 7.39 (dt, *J* = 7.8, 1.1 Hz, 1H), 7.28 (d, *J* = 7.8 Hz, 1H), 7.20 (ddd, *J* = 7.6, 4.9, 1.2 Hz, 1H), 7.15 – 7.03 (m, 2H), 4.74 (s, 2H), 2.44 (s, 2H), 0.95 (s, 9H), 0.11 (s, 6H), -0.22 (s, 9H).

^{13}C NMR: (126 MHz, $CDCl_3$)

162.3, 150.4, 142.6, 140.2, 139.0, 137.6, 131.6, 129.0, 126.0, 123.5, 122.8, 66.3, 27.5, 27.5, 25.0, 19.9, -3.7.

MS: (HRMS - ESI$^+$)

Found 408.2164 ($C_{22}H_{35}ONNaSi_2$), requires 408.2149.

2-{4-[((4-Methoxybenzyl)oxy)methyl]-2-[(trimethylsilyl)methyl]phenyl}pyridine 4d

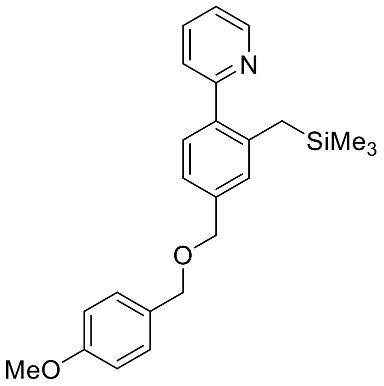

Following general procedure A, 2-{4-[((4-methoxybenzyl)oxy)methyl]phenyl}pyridine (76.0 mg, 0.250 mmol, 1.0 equiv.) in the presence of $[(^tBuCN)_5Ru(H_2O)](BF_4)_2$ (9.0 mg, 13 μmol, 5 mol%), potassium carbonate (69.0 mg, 0.500 mmol, 2.0 equiv.), potassium phenylphosphonate (17.6 mg, 75 μmol, 30 mol%), sodium iodide (37.5 mg, 0.250 mmol, 1.0 equiv.) and anhydrous tetrahydrofuran (500 μL) at 80 °C for 18 hours. The crude reaction mixture was purified by flash column chromatography (SiO_2, 10 g, biotage isolera, hexane/ethyl acetate – 100/0 to 93/7, 40 mL/min) to give 2-{4-[((4-methoxybenzyl)oxy)methyl]-2-[(trimethylsilyl)methyl]phenyl}pyridine (76.0 mg, 0.194 mmol, 78%) as a colourless oil.

TLC: R_f = 0.38 (4:1, hexane/ethyl acetate) [UV]

^{1}H NMR: (500 MHz, $CDCl_3$)

8.68 (ddd, *J* = 4.9, 1.9, 0.9 Hz, 1H), 7.73 (td, *J* = 7.7, 1.9 Hz, 1H), 7.40 (dt, *J* = 7.9, 1.1 Hz, 1H), 7.34 – 7.27 (m, 3H), 7.22 (ddd, *J* = 7.6, 4.8, 1.2 Hz, 1H), 7.16 (dd, *J* = 7.8, 1.7 Hz, 1H), 7.11 (d, *J* = 1.7 Hz, 1H), 6.93 – 6.85 (m, 2H), 4.54 (s, 2H), 4.48 (s, 2H), 3.81 (s, 3H), 2.46 (s, 2H), -0.21 (s, 9H).

^{13}C NMR: (126 MHz, $CDCl_3$)

162.1, 160.7, 150.4, 140.5, 139.6, 139.5, 137.6, 131.9, 131.8, 130.9, 130.8, 126.0, 125.3, 122.9, 115.3, 73.1, 72.9, 56.8, 25.0, 0.0.

MS: (HRMS - ESI$^+$)

Found 414.1856 ($C_{24}H_{29}O_2NNaSi$), requires 414.1860.

4-(Pyridin-2-yl)-3-[(trimethylsilyl)methyl]benzyl acetate 4e

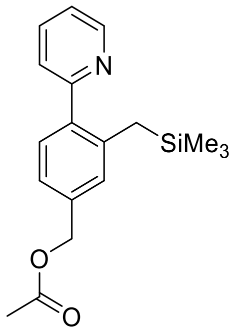

Following general procedure A, 4-(pyridin-2-yl)benzyl acetate (57.0 mg, 0.25 mmol, 1.0 equiv.) in the presence of $[(^{t}BuCN)_5Ru(H_2O)](BF_4)_2$ (9.0 mg, 13 μmol, 5 mol%), potassium carbonate (69.0 mg, 0.500 mmol, 2.0 equiv.), potassium phenylphosphonate (17.6 mg, 75 μmol, 30 mol%), sodium iodide (37.5 mg, 0.250 mmol, 1.0 equiv) and anhydrous tetrahydrofuran (500 μL) at 80 °C for 18 hours. The crude reaction mixture was purified by flash column chromatography (SiO_2, 25 g, biotage isolera, hexane/ethyl acetate – 100/0 to 70/30, 80 mL/min) to give 4-(pyridin-2-yl)-3-[(trimethylsilyl)methyl]benzyl acetate (54.0 mg, 0.172 mmol, 69%) as a colourless oil.

TLC: R_f = 0.38 (9:1, hexane/ethyl acetate) [UV]

^{1}H NMR: (500 MHz, $CDCl_3$)

8.68 (ddd, *J* = 4.9, 1.9, 1.0 Hz, 1H), 7.73 (td, *J* = 7.7, 1.9 Hz, 1H), 7.39 (dt, *J* = 7.9, 1.1 Hz, 1H), 7.32 (d, *J* = 7.8 Hz 1H), 7.22 (dd, *J* = 7.6, 4.9 Hz, 1H), 7.15 (dd, *J* = 7.9, 1.9 Hz, 1H), 7.09 (d, *J* = 1.7 Hz, 1H), 5.11 (s, 2H), 2.45 (s, 2H), 2.12 (s, 3H), -0.22 (s, 9H).

^{13}C NMR: (126 MHz, $CDCl_3$)

172.4, 161.9, 150.5, 140.9, 140.3, 137.7, 137.2, 131.9, 131.2, 126.0, 125.6, 123.1, 67.7, 25.1, 22.6, 0.0.

MS: (HRMS - ESI$^+$)

Found 336.1388 ($C_{18}H_{23}O_2NNaSi$), requires 336.1390.

2-{4-Methyl-2-[(trimethylsilyl)methyl]phenyl}pyridine 4f

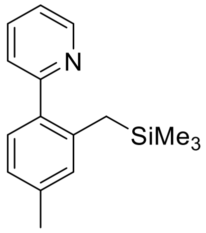

Following general procedure A, 2-(4-methylphenyl)pyridine (42 mg, 0.25 mmol) was reacted with trimethylsilylbromomethane (41.7 mg, 0.250 mmol, 1.0 equiv.) in the presence of $[(^{t}BuCN)_5Ru(H_2O)](BF_4)_2$ (9.0 mg, 13 µmol, 5 mol%), potassium carbonate (69.0 mg, 0.500 mmol, 2.0 equiv.), potassium phenylphosphonate (17.6 mg, 75.0 µmol, 30 mol%), sodium iodide (37.5 mg, 0.250 mmol, 1.0 equiv.) and anhydrous tetrahydrofuran (500 µL) at 80 °C for 18 hours. The crude reaction mixture was purified by flash column chromatography (SiO_2, 25 g, biotage isolera, hexane/ethyl acetate – 100/0 to 93/7, 40 mL/min) to give 2-{4-methyl-2-[(trimethylsilyl)methyl]phenyl}pyridine (40.0 mg, 0.157 mmol, 63%) as a colourless oil.

TLC: R_f = 0.70 (3:2, hexane/ethyl acetate) [UV]

^{1}H NMR: (500 MHz, $CDCl_3$)

8.66 (ddd, *J* = 4.9, 1.8, 0.9 Hz, 1H), 7.71 (td, *J* = 7.7, 1.9 Hz, 1H), 7.38 (dt, *J* = 7.7, 1.0 Hz, 1H), 7.24 – 7.14 (m, 2H), 6.97 (ddd, *J* = 7.7, 1.8, 0.8 Hz, 1H), 6.92 (d, *J* = 1.8 Hz, 1H), 2.42 (s, 2H), 2.34 (s, 3H), -0.22 (s, 9H).

^{13}C NMR: (101 MHz, $CDCl_3$)

162.4, 150.4, 140.2, 139.1, 137.6, 132.1, 131.6, 126.6, 126.0, 122.7, 24.9, 22.8, 0.0.

MS: (HRMS - ESI^{+})

Found 278.1338 ($C_{16}H_{21}NNaSi$), requires 278.1335.

2-{3-[(Trimethylsilyl)methyl]-[1,1'-biphenyl]-4-yl}pyridine 4g

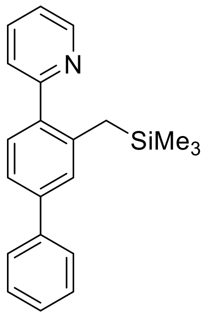

Following general procedure A, 2-[(1,1'-biphenyl)-4-yl]pyridine (58.0 mg, 0.250 mmol, 1.0 equiv.) in the presence of $[(^tBuCN)_5Ru(H_2O)](BF_4)_2$ (9.0 mg, 13 µmol, 5 mol%), potassium carbonate (69.0 mg, 0.500 mmol, 2.0 equiv.), potassium phenylphosphonate (17.6 mg, 75 µmol, 30 mol%), sodium iodide (37.5 mg, 0.250 mmol, 1.0 equiv.) and anhydrous tetrahydrofuran (500 µL) at 80 °C for 18 hours. The crude reaction mixture was purified by flash column chromatography (SiO_2, 25 + 10 g, biotage isolera, hexane/ethyl acetate – 100/0 to 97/3, 80 mL/min) to give 2-{3-[(trimethylsilyl)methyl]-[1,1'-biphenyl]-4-yl}pyridine (64.0 mg, 0.201 mmol, 81%) as a colourless oil.

TLC: R_f = 0.33 (4:1, hexane/ethyl acetate) [UV]

^{1}H NMR: (500 MHz, $CDCl_3$)

8.73 – 8.67 (m, 1H), 7.75 (td, *J* = 7.7, 1.9 Hz, 1H), 7.63 (dt, *J* = 7.8, 1.2 Hz, 2H), 7.49 – 7.42 (m, 3H), 7.42 – 7.38 (m, 2H), 7.38 – 7.32 (m, 2H), 7.23 (ddd, *J* = 7.5, 4.9, 1.2 Hz, 1H), 2.54 (s, 2H), -0.17 (d, *J* = 0.9 Hz, 9H).

^{13}C NMR: (126 MHz, $CDCl_3$)

162.0, 150.4, 142.5, 142.2, 140.8, 139.3, 137.6, 132.1, 130.2, 130.1, 128.7, 128.6, 125.9, 124.6, 122.9, 25.2, 0.0.

MS: (HRMS - ESI^+)

Found 340.1489 ($C_{21}H_{23}NNaSi$), requires 340.1492.

4-(Pyridin-2-yl)-3-[(trimethylsilyl)methyl]aniline 4h

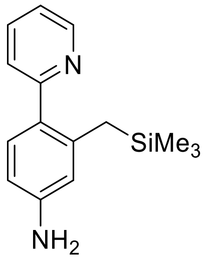

Following general procedure A, 4-(pyridin-2-yl)aniline (43.0 mg, 0.25 mmol, 1.0 equiv.) in the presence of $[(^{t}BuCN)_5Ru(H_2O)](BF_4)_2$ (9.0 mg, 13 μmol, 5 mol%), potassium carbonate (69.0 mg, 0.500 mmol, 2.0 equiv.), potassium phenylphosphonate (17.6 mg, 75 μmol, 30 mol%), sodium iodide (37.5 mg, 0.250 mmol, 1.0 equiv.) and anhydrous tetrahydrofuran (500 μL) at 80 °C for 18 hours. The crude reaction mixture was purified by flash column chromatography (SiO_2, 25 g, biotage isolera, hexane/ethyl acetate – 100/0 to 70/30, 60 mL/min) to give 4-(pyridin-2-yl)-3-[(trimethylsilyl)methyl]aniline (35.0 mg, 0.137 mmol, 55%) as a colourless oil.

TLC: R_f = 0.42 (7:3, hexane/ethyl acetate) [UV]

^{1}H NMR: (400 MHz, $CDCl_3$)

8.63 (ddd, *J* = 4.9, 1.9, 1.0 Hz, 1H), 7.67 (td, *J* = 7.7, 1.9 Hz, 1H), 7.35 (dt, *J* = 7.9, 1.1 Hz, 1H), 7.21 – 7.09 (m, 2H), 6.50 (dd, *J* = 8.2, 2.4 Hz, 1H), 6.42 (d, *J* = 2.4 Hz, 1H), 3.85 – 3.48 (m, 2H), 2.42 (s, 2H), -0.22 (s, 9H).

^{13}C NMR: (101 MHz, $CDCl_3$)

162.5, 150.2, 147.6, 141.7, 137.4, 132.9, 131.3, 125.8, 122.1, 117.5, 112.9, 25.1, 0.0.

IR: (*neat*, cm^{-1})

3455.5 (br.), 2952.1 (w), 1605.6 (s), 1507.5 (m), 848.6 (s).

MS: (HRMS - ESI^+)

Found 279.1284 ($C_{15}H_{20}N_2NaSi$), requires 279.1288.

4-(Pyridin-2-yl)-3-[(trimethylsilyl)methyl]phenol 4i

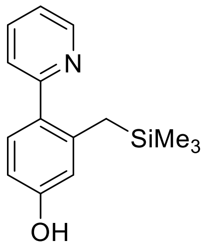

Following general procedure A, 4-(pyridin-2-yl)phenol (43.0 mg, 0.250 mmol, 1.0 equiv.) in the presence of $[({}^{t}BuCN)_5Ru(H_2O)](BF_4)_2$ (9.0 mg, 13 μmol, 5 mol%), potassium carbonate (69.0 mg, 0.500 mmol, 2.0 equiv.), potassium phenylphosphonate (17.6 mg, 75 μmol, 30 mol%), sodium iodide (37.5 mg, 0.250 mmol, 1.0 equiv.) and anhydrous tetrahydrofuran (500 μL) at 80 °C for 18 hours. The crude reaction mixture was purified by flash column chromatography (SiO_2, 25 g, biotage isolera, hexane/ethyl acetate – 100/0 to 75/25, 60 mL/min) to give 4-(pyridin-2-yl)-3-[(trimethylsilyl)methyl]phenol (40.0 mg, 0.155 mmol, 62%) as a colourless amorphous solid.

TLC: R_f = 0.26 (4:1, hexane/ethyl acetate) [UV]

mpt: 132 – 134 °C (hexane)

^{1}H NMR: (400 MHz, $CDCl_3$)

8.64 (ddd, *J* = 5.0, 1.9, 0.9 Hz, 1H), 7.73 (td, *J* = 7.7, 1.8 Hz, 1H), 7.59 (bs., 1H), 7.38 (dt, *J* = 7.8, 1.1 Hz, 1H), 7.22 (ddd, *J* = 7.6, 5.0, 1.2 Hz, 1H), 7.11 (d, *J* = 8.3 Hz, 1H), 6.50 (dd, *J* = 8.2, 2.6 Hz, 1H), 6.46 (d, *J* = 2.5 Hz, 1H), 2.31 (s, 2H), -0.25 (s, 9H).

^{13}C NMR: (101 MHz, $CDCl_3$)

162.2, 157.9, 149.9, 142.0, 138.2, 132.9, 132.2, 126.4, 122.8, 118.4, 113.7, 25.1, 0.0.

IR: (*neat*, cm^{-1})

2950.6 (w), 1603.1 (m), 1468.7 (m), 1286.7 (m), 852.3 (s).

MS: (HRMS - ESI^+)

Found 280.1125 ($C_{15}H_{19}ONNaSi$), requires 280.1128.

2-{5-(Trifluoromethyl)-2-[(trimethylsilyl)methyl]phenyl}pyridine 4k

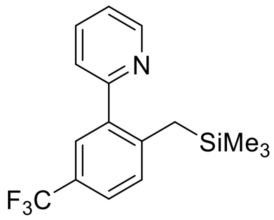

Following general procedure A, 2-[3-(trifluoromethyl)phenyl]pyridine (56.0 mg, 0.250 mmol) was reacted with trimethylsilylbromomethane (41.8 mg, 0.250 mmol, 1.0 equiv.) in the presence of $[(^t BuCN)_5Ru(H_2O)](BF_4)_2$ (9.0 mg, 13 µmol, 5 mol%), potassium carbonate (69.0 mg, 0.500 mmol, 2.0 equiv.), potassium phenylphosphonate (17.6 mg, 75.0 µmol, 30 mol%), sodium iodide (37.5 mg, 0.250 mmol, 1.0 equiv.) and anhydrous tetrahydrofuran (500 µL) at 80 °C for 18 hours. The crude reaction mixture was purified by flash column chromatography (SiO_2, 25 g, biotage isolera, hexane/ethyl acetate – 100/0 to 93/7, 40 mL/min) to give 2-{5-(trifluoromethyl)-2-[(trimethylsilyl)methyl]phenyl}pyridine (70.0 mg, 0.226 mmol, 90%) as a colourless oil.

TLC: R_f = 0.34 (9:1, hexane/ethyl acetate) [UV]

^{1}H NMR: (500 MHz, $CDCl_3$)

8.70 (ddd, *J* = 4.9, 1.8, 0.9 Hz, 1H), 7.78 (td, *J* = 7.7, 1.8 Hz, 1H), 7.57 (d, *J* = 2.0 Hz, 1H), 7.49 (dd, *J* = 8.2, 2.0 Hz, 1H), 7.42 (dt, *J* = 7.9, 1.1 Hz, 1H), 7.28 (ddd, *J* = 7.6, 4.8, 1.1 Hz, 1H), 7.21 (s, 1H), 2.52 (s, 2H), -0.20 (s, 9H).

^{13}C NMR: (101 MHz, $CDCl_3$)

160.9, 150.7, 140.5, 138.0, 131.7, 128.5 (q, *J* = 3.9 Hz), 128.1 (q, *J* = 32 Hz), 126.2 (q, *J* = 3.7 Hz), 126.0, 125.8 (q, *J* = 272 Hz), 124.6, 123.6, 25.6, 0.0.

^{19}F NMR: (376 MHz, $CDCl_3$)

-62.1.

MS: (HRMS - ESI$^+$)

Found 310.1248 ($C_{16}H_{19}NF_3Si$), requires 310.1233.

2-{5-[Bis(trimethylsilyl)methyl]-2-[(trimethylsilyl)methyl]phenyl}pyridine 4l

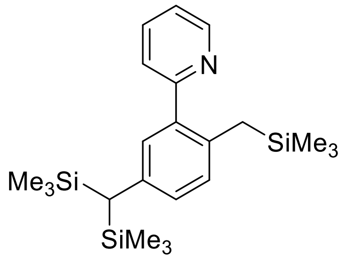

Following general procedure A, 2-{3-[bis(trimethylsilyl)methyl]phenyl}pyridine (78.0 mg, 0.250 mmol) was reacted with trimethylsilylbromomethane (41.8 mg, 0.250 mmol, 1.0 equiv.) in the presence of $[(^t\text{BuCN})_5\text{Ru}(\text{H}_2\text{O})](\text{BF}_4)_2$ (18.0 mg, 26 μmol, 10 mol%), potassium carbonate (69.0 mg, 0.500 mmol, 2.0 equiv.), potassium phenylphosphonate (35.0 mg, 150 μmol, 60 mol%), sodium iodide (37.5 mg, 0.250 mmol, 1.0 equiv.) and anhydrous tetrahydrofuran (500 μL) at 80 °C for 18 hours. The crude reaction mixture was purified by flash column chromatography (SiO_2, 25 g + 10 g, biotage isolera, hexane/ethyl acetate – 100/0 to 96/4, 20 mL/min) to give 2-{5-[bis(trimethylsilyl)methyl]-2-[(trimethylsilyl)methyl]phenyl}pyridine (56.0 mg, 0.140 mmol, 56%) as a colourless oil.

TLC: R_f = 0.27 (9:1, hexane/ethyl acetate) [UV]

^{1}H NMR: (500 MHz, $CDCl_3$)

8.66 (ddd, *J* = 4.9, 1.9, 0.9 Hz, 1H), 7.71 (td, *J* = 7.7, 1.9 Hz, 1H), 7.33 (dt, *J* = 7.8, 1.1 Hz, 1H), 7.19 (ddd, *J* = 7.5, 4.9, 1.2 Hz, 1H), 6.93 (d, *J* = 7.9 Hz, 1H), 6.87 (d, *J* = 2.1 Hz, 1H), 6.82 (dd, *J* = 7.9, 2.1 Hz, 1H), 2.40 (s, 2H), 1.48 – 1.43 (m, 1H), -0.26 (s, 9H).

^{13}C NMR: (126 MHz, $CDCl_3$)

161.31, 148.49, 138.24, 138.17, 135.97, 133.42, 130.23, 129.52, 128.26, 124.24, 120.98, 28.29, 22.27, -1.71.

MS: (HRMS - ESI^+)

Found 422.2125 ($C_{22}H_{27}NNaSi_3$), requires 422.2126.

Piperidin-1-yl{6-[2-((trimethylsilyl)methyl)phenyl]pyridin-3-yl}methanone 4m

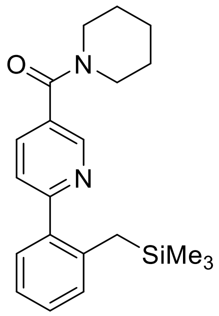

Following general procedure A, (6-phenylpyridin-3-yl)(piperidin-1-yl)methanone (66.6 mg, 0.250 mmol, 1.0 equiv.) in the presence of $[(^{t}BuCN)_5Ru(H_2O)](BF_4)_2$ (9.0 mg, 13 µmol, 5 mol%), potassium carbonate (69.0 mg, 0.500 mmol, 2.0 equiv.), potassium phenylphosphonate (17.6 mg, 75 µmol, 30 mol%), sodium iodide (37.5 mg, 0.250 mmol, 1 equiv.) and anhydrous tetrahydrofuran (500 µL) at 80 °C for 18 hours. The crude reaction mixture was purified by flash column chromatography (SiO_2, 10 g, biotage isolera, hexane/ethyl acetate – 100/0 to 75/25, 40 mL/min) to give piperidin-1-yl{6-[2-((trimethylsilyl)methyl)phenyl]pyridin-3-yl}methanone (80.0 mg, 0.225 mmol, 90%) as a pale yellow oil.

TLC: R_f = 0.34 (1:1, hexane/ethyl acetate) [UV]

^{1}H NMR: (400 MHz, $CDCl_3$)

8.72 (dd, *J* = 2.3, 0.9 Hz, 1H), 7.81 (dd, *J* = 8.0, 2.3 Hz, 1H), 7.46 (dd, *J* = 8.0, 0.9 Hz, 1H), 7.34 – 7.24 (m, 2H), 7.20 – 7.08 (m, 2H), 4.00 – 3.13 (m, 4H), 2.44 (s, 2H), 1.79 – 1.48 (m, 6H), -0.20 (s, 9H).

^{13}C NMR: (101 MHz, $CDCl_3$)

169.3, 163.2, 148.5, 140.5, 139.4, 136.7, 131.6, 131.4, 131.1, 129.8, 125.7, 125.5, 50.4, 44.8, 28.0, 27.0, 25.9, 25.0, 0.0.

IR: (*neat*, cm^{-1})

2932.4 (w), 1737.3 (w), 1630.6 (m), 1591.3 (m), 1465.8 (m), 838.1 (w).

MS: (HRMS - ESI^+)

Found 375.1858 ($C_{21}H_{28}ON_2NaSi$), requires 375.1863.

N-[2-(1H-Indol-3-yl)ethyl]-6-{2-[(trimethylsilyl)methyl]phenyl}nicotinamide 4n

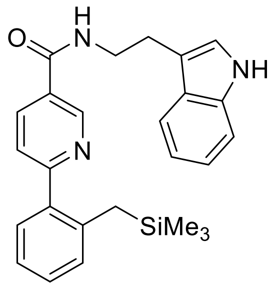

Following general procedure A, *N*-[2-(*1H*-indol-3-yl)ethyl]-6-phenylnicotinamide (85.3 mg, 0.250 mmol, 1.0 equiv.) in the presence of $[(^tBuCN)_5Ru(H_2O)](BF_4)_2$ (9.0 mg, 13 µmol, 5 mol%), potassium carbonate (69.0 mg, 0.500 mmol, 2.0 equiv.), potassium phenylphosphonate (17.6 mg, 75 µmol, 30 mol%), sodium iodide (37.5 mg, 0.250 mmol, 1.0 equiv.) and anhydrous tetrahydrofuran (500 µL) at 80 °C for 18 hours. The crude reaction mixture was purified by flash column chromatography (SiO_2, 10 g, biotage isolera, hexane/ethyl acetate – 100/0 to 60/40, 40 mL/min) to give *N*-[2-(*1H*-indol-3-yl)ethyl]-6-{2-[(trimethylsilyl)methyl]phenyl}nicotinamide (69.0 mg, 0.161 mmol, 65%) as a yellow oil.

TLC: R_f = 0.40 (1:1, hexane/ethyl acetate) [UV]

^{1}H NMR: (500 MHz, $CDCl_3$)

8.88 (dd, *J* = 2.4, 0.8 Hz, 1H), 8.19 – 8.00 (m, 2H), 7.67 (d, *J* = 7.9 Hz, 1H), 7.46 (d, *J* = 8.1 Hz, 1H), 7.40 (d, *J* = 8.2 Hz, 1H), 7.31 – 7.26 (m, 2H), 7.26 – 7.21 (m, 1H), 7.20 – 7.07 (m, 4H), 6.26 (t, *J* = 6.0 Hz, 1H), 3.85 (q, *J* = 6.4 Hz, 2H), 3.15 (t, *J* = 6.6 Hz, 2H), 2.41 (s, 2H), -0.22 (s, 9H).

^{13}C NMR: (101 MHz, $CDCl_3$)

167.0, 165.0, 148.4, 140.5, 139.3, 138.0, 136.9, 131.6, 131.5, 130.0, 129.3, 128.7, 125.8, 125.7, 123.9, 123.6, 121.2, 120.2, 114.4, 112.9, 41.8, 26.7, 25.0, 0.0.

IR: (*neat*, cm^{-1})

3305.6 (br.), 2952.1 (w), 1735.9 (w), 1638.7 (m), 1245.0 (s), 838.6 (s).

MS: (HRMS - ESI^+)

Found 450.1969 ($C_{26}H_{29}ON_3NaSi$), requires 450.1972.

(*S*)-4-{2-[(*t*ert-Butoxycarbonyl)amino]-3-methoxy-3-oxopropyl}phenyl 4o

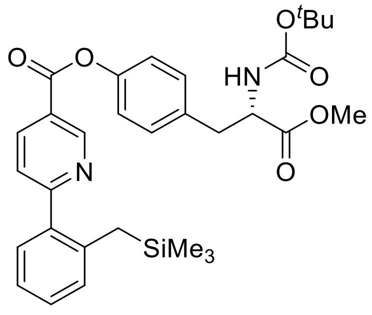

Following general procedure A, (*S*)-4-{2-[(*tert*-butoxycarbonyl)amino]-3-methoxy-3-oxopropyl}phenyl 6-phenylnicotinate (119.0 mg, 0.250 mmol, 1.0 equiv.) in the presence of $[(^tBuCN)_5Ru(H_2O)](BF_4)_2$ (9.0 mg, 13 µmol, 5 mol%), potassium carbonate (69.0 mg, 0.500 mmol, 2.0 equiv.), potassium phenylphosphonate (17.6 mg, 75 µmol, 30 mol%), sodium iodide (37.5 mg, 0.250 mmol, 1.0 equiv.) and anhydrous tetrahydrofuran (500 µL) at 80 °C for 18 hours. The crude reaction mixture was purified by flash column chromatography (SiO_2, 10 g, biotage isolera, hexane/ethyl acetate – 100/0 to 70/30, 40 mL/min) to give (*S*)-4-{2-[(*t*ert-Butoxycarbonyl)amino]-3-methoxy-3-oxopropyl}phenyl 6-{2-[(trimethylsilyl)methyl]phenyl}nicotinate (120.0 mg, 0.213 mmol, 85%) as a colourless oil.

TLC: R_f = 0.22 (4:1, hexane/ethyl acetate) [UV]

^{1}H NMR: (400 MHz, $CDCl_3$)

9.43 (dd, *J* = 2.3, 0.9 Hz, 1H), 8.47 (dd, *J* = 8.2, 2.3 Hz, 1H), 7.58 (dd, *J* = 8.2, 0.9 Hz, 1H), 7.40 – 7.27 (m, 2H), 7.25 – 7.10 (m, 6H), 5.14 – 4.90 (m, 1H), 4.71 – 4.42 (m, 1H), 3.74 (s, 3H), 3.28 – 2.97 (m, 2H), 2.49 (s, 2H), 1.44 (s, 9H), -0.17 (s, 9H).

^{13}C NMR: (101 MHz, $CDCl_3$)

173.6, 167.0, 165.5, 156.5, 152.1, 151.1, 140.8, 139.3, 139.1, 135.5, 131.9, 131.7, 131.6, 130.3, 125.9, 125.7, 124.5, 123.1, 81.5, 55.8, 53.8, 39.2, 29.8, 25.1, 0.0.

IR: (*neat*, cm^{-1})

2953.2 (w), 1736.4 (s), 1713.1 (s), 1592.7 (m), 1163.0 (s), 1016.5 (s), 840.1 (s).

MS: (HRMS - ESI^+)

Found 585.2391 ($C_{31}H_{38}O_6N_2NaSi$), requires 585.2391.

(8R,9S,13S,14S)-13-Methyl-17-oxo-7,8,9,11,12,13,14,15,16,17-decahydro-*6H*-cyclopenta[a]phenanthren-3-yl 6-{2-[(trimethylsilyl)methyl]phenyl}nicotinate 4p

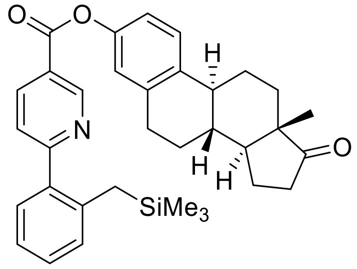

Following general procedure A, (8R,9S,13S,14S)-13-methyl-17-oxo-7,8,9,11,12,13,14,15,16,17-decahydro-6H-cyclopenta[a]phenanthren-3-yl 6-phenylnicotinate (112.8 mg, 0.250 mmol) was reacted with trimethylsilylbromomethane (41.8 mg, 0.250 mmol, 1.0 equiv.) in the presence of [(^{t}BuCN)$_5$Ru(H_2O)](BF_4)$_2$ (9.0 mg, 13 μmol, 5 mol%), potassium carbonate (69.0 mg, 0.500 mmol, 2.0 equiv.), potassium phenylphosphonate (17.6 mg, 75.0 μmol, 30 mol%), sodium iodide (37.5 mg, 0.250 mmol, 1.0 equiv.) and anhydrous tetrahydrofuran (500 μL) at 80 °C for 18 hours. The crude reaction mixture was purified by flash column chromatography (SiO_2, 10 g, biotage isolera, hexane/ethyl acetate – 100/0 to 90/10, 40 mL/min) to give (8R,9S,13S,14S)-13-methyl-17-oxo-7,8,9,11,12,13,14,15,16,17-decahydro-*6H*-cyclopenta[a]phenanthren-3-yl 6-{2-[(trimethylsilyl)methyl]phenyl}nicotinate (86 mg, 0.160 mmol, 64%) as colourless plates.

TLC: R_f = 0.33 (4:1, hexane/ethyl acetate) [UV]

mpt: 146 – 148 °C (95:5, hexane/ethyl acetate)

^{1}H NMR: (500 MHz, $CDCl_3$)

9.43 (dd, *J* = 2.3, 0.8 Hz, 1H), 8.48 (dd, *J* = 8.2, 2.3 Hz, 1H), 7.58 (d, *J* = 8.2 Hz, 1H), 7.40 – 7.34 (m, 2H), 7.31 (td, *J* = 7.5, 1.5 Hz, 1H), 7.20 (td, *J* = 7.5, 1.3 Hz, 1H), 7.15 (dd, *J* = 7.7, 1.3 Hz, 1H), 7.03 (dd, *J* = 8.4, 2.6 Hz, 1H), 7.00 (d, *J* = 2.5 Hz, 1H), 3.00 – 2.92 (m, 2H), 2.57 – 2.39 (m, 4H), 2.34 (td, *J* = 11.2, 4.6 Hz, 1H), 2.21 – 1.93 (m, 4H), 1.71 – 1.43 (m, 6H), 0.94 (s, 3H), -0.17 (s, 9H).

^{13}C NMR: (101 MHz, $CDCl_3$)

222.2, 166.9, 165.8, 152.1, 150.0, 140.8, 139.7, 139.2 (2 x resonances), 139.1, 131.7, 131.6, 130.3, 128.0, 125.9, 125.6, 124.6, 123.1, 120.2, 51.9, 49.4, 45.6, 39.5, 37.3, 33.0, 30.9, 27.8, 27.2, 25.1, 23.1, 15.3, 0.0.

IR: (*neat*, cm^{-1})

2932.1 (w), 1736.7 (s), 1592.0 (s), 1251.8 (s), 846.9 (s), 733.7 (s).

MS: (HRMS - ESI$^+$)

Found 560.2599 ($C_{34}H_{39}O_3NNaSi$), requires 560.2591.

2-{2-Methyl-6-[(trimethylsilyl)methyl]phenyl}pyrimidine 4q

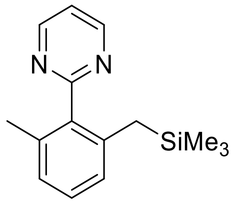

Following general procedure A, 2-(*o*-tolyl)pyrimidine (42.6 mg, 0.250 mmol) was reacted with trimethylsilylbromomethane (41.8 mg, 0.250 mmol, 1.0 equiv.) in the presence of $[(^t BuCN)_5Ru(H_2O)](BF_4)_2$ (18.0 mg, 25 μmol, 10 mol%), potassium carbonate (69.0 mg, 0.500 mmol, 2.0 equiv.), potassium phenylphosphonate (35.2 mg, 150 μmol, 60 mol%), sodium iodide (37.5 mg, 0.250 mmol, 1.0 equiv.) and anhydrous tetrahydrofuran (500 μL) at 80 °C for 18 hours. The crude reaction mixture was purified by flash column chromatography (SiO_2, 25 g, biotage isolera, hexane/ethyl acetate – 100/0 to 95/5, 80 mL/min) to give 2-{2-methyl-6-[(trimethylsilyl)methyl]phenyl}pyrimidine (43.0 mg, 0.168 mmol, 67%) as a colourless oil.

TLC: R_f = 0.30 (4:1, hexane/ethyl acetate) [UV]

^{1}H NMR: (400 MHz, $CDCl_3$)

8.87 (d, *J* = 4.9 Hz, 2H), 7.24 (t, *J* = 4.9 Hz, 1H), 7.17 (t, *J* = 7.6 Hz, 1H), 7.04 – 6.88 (m, 2H), 2.06 (s, 3H), 2.00 (s, 2H), -0.15 (s, 9H).

^{13}C NMR: (126 MHz, $CDCl_3$)

170.0, 158.2, 139.7, 138.9, 137.0, 129.3, 128.1, 127.4, 120.0, 24.9, 21.6, 0.0.

MS: (HRMS - ESI$^+$)

Found 279.1297 ($C_{15}H_{20}N_2NaSi$), requires 279.1288.

1-{2-[(Trimethylsilyl)methyl]phenyl}-*1H*-pyrazole 4r

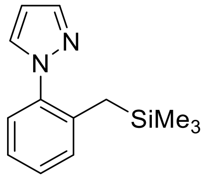

Following general procedure A, 1-phenyl-*1H*-pyrazole (36.0 mg, 0.250 mmol) was reacted with trimethylsilylbromomethane (41.8 mg, 0.250 mmol, 1.0 equiv.) in the presence of $[(^{t}BuCN)_5Ru(H_2O)](BF_4)_2$ (18.0 mg, 25 µmol, 10 mol%), potassium carbonate (69.0 mg, 0.500 mmol, 2.0 equiv.), potassium phenylphosphonate (35.2 mg, 150 µmol, 60 mol%), sodium iodide (37.5 mg, 0.250 mmol, 1.0 equiv.) and anhydrous tetrahydrofuran (500 µL) at 80 °C for 18 hours. The crude reaction mixture was purified by flash column chromatography (SiO_2, 25 g, biotage isolera, hexane/ethyl acetate – 100/0 to 96/4, 60 mL/min) to give 1-{2-[(trimethylsilyl)methyl]phenyl}-*1H*-pyrazole (30.0 mg, 0.130 mmol, 52%) as a colourless oil.

TLC: R_f = 0.45 (4:1, hexane/ethyl acetate) [UV]

^{1}H NMR: (500 MHz, $CDCl_3$)

7.77 – 7.70 (m, 1H), 7.62 (dd, *J* = 2.3, 0.7 Hz, 1H), 7.35 – 7.24 (m, 2H), 7.24 – 7.15 (m, 2H), 6.45 (t, *J* = 2.1 Hz, 1H), 2.28 (s, 2H), -0.11 (s, 9H).

^{13}C NMR: (126 MHz, $CDCl_3$)

141.6, 140.1, 138.5, 132.2, 131.9, 129.7, 128.2, 126.3, 107.6, 23.5, 0.0.

MS: (HRMS - ESI^+)

Found 253.1127 ($C_{13}H_{18}N_2NaSi$), requires 253.1131.

4-(Thiophen-2-yl)-1-{2-[(trimethylsilyl)methyl]phenyl}-*1H*-pyrazole 4s

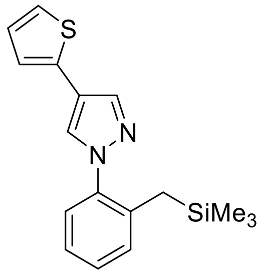

Following general procedure A, 1-phenyl-4-(thiophen-2-yl)-*1H*-pyrazole (56.6 mg, 0.250 mmol) was reacted with trimethylsilylbromomethane (41.8 mg, 0.250 mmol, 1.0 equiv.) in the presence of $[(^tBuCN)_5Ru(H_2O)](BF_4)_2$ (18.0 mg, 25 μmol, 10 mol%), potassium carbonate (69.0 mg, 0.500 mmol, 2.0 equiv.), potassium phenylphosphonate (35.2 mg, 150 μmol, 60 mol%), sodium iodide (37.5 mg, 0.250 mmol, 1.0 equiv.) and anhydrous tetrahydrofuran (500 μL) at 80 °C for 18 hours. The crude reaction mixture was purified by flash column chromatography (SiO_2, 25 g, biotage isolera, hexane/ethyl acetate – 100/0 to 93/7, 80 mL/min) to give 4-(thiophen-2-yl)-1-{2-[(trimethylsilyl)methyl]phenyl}-*1H*-pyrazole (28 mg, 0.090 mmol, 36%) as a colourless oil.

TLC: R_f = 0.43 (4:1, hexane/ethyl acetate) [UV]

^{1}H NMR: (400 MHz, $CDCl_3$)

7.88 (s, 1H), 7.76 (s, 1H), 7.32 – 7.26 (m, 2H), 7.23 – 7.11 (m, 4H), 7.05 (dd, *J* = 5.1, 3.5 Hz, 1H), 2.27 (s, 2H), -0.11 (s, 9H).

^{13}C NMR: (126 MHz, $CDCl_3$)

139.6, 139.3, 138.4, 136.3, 131.9, 129.9, 129.2, 129.0, 128.0, 126.3, 124.6, 124.1, 119.0, 23.5, 0.0.

MS: (HRMS - ESI$^+$)

Found 313.1205 ($C_{17}H_{21}N_2SSi$), requires 313.1189.

Methyl 3-(*1H*-pyrazol-1-yl)-4-[(trimethylsilyl)methyl]benzoate 4t

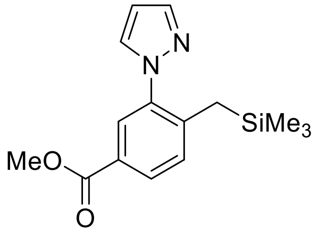

Following general procedure A, methyl 3-(*1H*-pyrazol-1-yl)benzoate (50.6 mg, 0.250 mmol) was reacted with trimethylsilylbromomethane (41.8 mg, 0.250 mmol, 1.0 equiv.) in the presence of $[(^tBuCN)_5Ru(H_2O)](BF_4)_2$ (18.0 mg, 25 µmol, 10 mol%), potassium carbonate (69.0 mg, 0.500 mmol, 2.0 equiv.), potassium phenylphosphonate (35.2 mg, 150 µmol, 60 mol%), sodium iodide (37.5 mg, 0.250 mmol, 1.0 equiv.) and anhydrous tetrahydrofuran (500 µL) at 80 °C for 18 hours. The crude reaction mixture was purified by flash column chromatography (SiO_2, 25 g, biotage isolera, hexane/ethyl acetate – 100/0 to 90/10, 80 mL/min) to give methyl 3-(*1H*-pyrazol-1-yl)-4-[(trimethylsilyl)methyl]benzoate (50.0 mg, 0.173 mmol, 69%) as a colourless oil.

TLC: R_f = 0.48 (4:1, hexane/ethyl acetate) [UV]

^{1}H NMR: (400 MHz, $CDCl_3$)

7.96 – 7.87 (m, 2H), 7.72 (d, *J* = 1.9 Hz, 1H), 7.62 (dd, *J* = 2.4, 0.6 Hz, 1H), 7.22 (d, *J* = 8.0 Hz, 1H), 6.44 (t, *J* = 2.1 Hz, 1H), 3.90 (s, 3H), 2.38 (s, 2H), -0.15 (s, 9H).

^{13}C NMR: (126 MHz, $CDCl_3$)

167.9, 144.8, 142.0, 139.9, 132.3, 132.0, 130.6, 129.3, 128.6, 108.1, 53.7, 24.7, 0.0.

MS: (HRMS - ESI$^+$)

Found 311.1180 ($C_{15}H_{20}O_2N_2NaSi$), requires 311.1186.

2-{5-(Trifluoromethyl)-2-[(trimethylsilyl)methyl]phenyl}-4,5-dihydrooxazole 4u

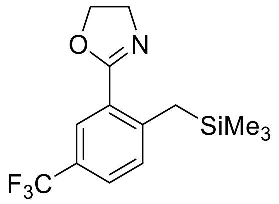

Following general procedure A, 2-[3-(trifluoromethyl)phenyl]-4,5-dihydrooxazole (53.8 mg, 0.25 mmol) was reacted with trimethylsilylbromomethane (41.8 mg, 0.250 mmol, 1.0 equiv.) in the presence of $[(^{t}BuCN)_5Ru(H_2O)](BF_4)_2$ (18.0 mg, 25 µmol, 10 mol%), potassium carbonate (69.0 mg, 0.50 mmol, 2.0 equiv.), potassium phenylphosphonate (35.2 mg, 150 µmol, 60 mol%), sodium iodide (37.5 mg, 0.250 mmol, 1.0 equiv.) and anhydrous tetrahydrofuran (500 µL) at 80 °C for 18 hours. The crude reaction mixture was purified by flash column chromatography (SiO_2, 10 g, biotage isolera, hexane/ethyl acetate – 100/0 to 92/8, 40 mL/min) to give 2-{5-(trifluoromethyl)-2-[(trimethylsilyl)methyl]phenyl}-4,5-dihydrooxazole (22.0 mg, 0.073 mmol, 29%) as a yellow oil.

TLC: R_f = 0.70 (9:1) (hexane/ethyl acetate) [UV]

^{1}H NMR: (400 MHz, $CDCl_3$)

8.10 – 8.00 (m, 1H), 7.50 (dd, *J* = 8.2, 2.1 Hz, 1H), 7.16 (d, *J* = 8.1 Hz, 1H), 4.37 (t, *J* = 9.6 Hz, 2H), 4.08 (t, *J* = 9.6 Hz, 2H), 2.85 (s, 2H), -0.05 (s, 9H).

^{13}C NMR: (126 MHz, $CDCl_3$)

165.72, 148.30, 132.11, 128.9 (q, *J* = 3.9 Hz), 128.2 (q, *J* = 3.8 Hz), 127.8 (q, *J* = 32.8 Hz), 127.4, 125.8 (q, *J* = 272 Hz), 68.3, 57.0, 27.7, 0.0.

^{19}F NMR: (376 MHz, $CDCl_3$)

-62.30.

MS: (HRMS - ESI^+)

Found 302.1176 ($C_{14}H_{19}ONF_3Si$), requires 302.1183.

1-{2-[(Trimethylsilyl)methyl]phenyl}ethan-1-one 4v

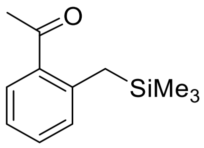

Following general procedure A, (*E*)-*N*,*N*-dimethyl-4-[(1-phenylethylidene)amino]aniline (59.6 mg, 0.250 mmol) was reacted with trimethylsilylbromomethane (41.8 mg, 0.250 mmol, 1.0 equiv.) in the presence of $[(^{t}BuCN)_5Ru(H_2O)](BF_4)_2$ (18.0 mg, 25 μmol, 10 mol%), potassium carbonate (69.0 mg, 0.500 mmol, 2.0 equiv.), potassium phenylphosphonate (35.2 mg, 150 μmol, 60 mol%), sodium iodide (37.5 mg, 0.250 mmol, 1.0 equiv.) and anhydrous tetrahydrofuran (500 μL) at 80 °C for 18 hours. Hydrochloric acid (3 M, 3 mL) was added at 20 °C, the reaction mixture stirred for 3 hours and the organics extracted using ethyl acetate (3 x 2 mL). The crude reaction mixture was purified by flash column chromatography (SiO_2, 50 g, biotage isolera, hexane/ethyl acetate – 100/0 to 95/5, 120 mL/min) to give 1-{2-[(trimethylsilyl)methyl]phenyl}ethan-1-one (27.0 mg, 0.131 mmol, 52%) as a colourless oil.

TLC: R_f = 0.68 (17:1) (hexane/ethyl acetate) [UV]

^{1}H NMR: (500 MHz, $CDCl_3$)

7.70 (dd, *J* = 7.9, 1.4 Hz, 1H), 7.32 (td, *J* = 7.5, 1.5 Hz, 1H), 7.14 (td, *J* = 7.6, 1.3 Hz, 1H), 7.07 (dd, *J* = 7.8, 1.3 Hz, 1H), 2.61 (s, 2H), 2.57 (s, 3H), -0.05 (s, 9H).

^{13}C NMR: (126 MHz, $CDCl_3$)

201.6, 142.5, 135.7, 131.5, 131.2, 130.2, 123.9, 29.7, 25.4, -1.4.

MS: (HRMS - ESI$^+$)

Found 229.1016 ($C_{12}H_{18}ONaSi$), requires 229.1019.

1-{4-Methyl-2-[(trimethylsilyl)methyl]phenyl}ethan-1-one 4w

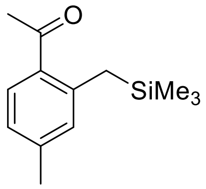

Following general procedure A, (*E*)-*N*,*N*-dimethyl-4-{[1-(p-tolyl)ethylidene]amino}aniline (63.1 mg, 0.250 mmol) was reacted with trimethylsilylbromomethane (41.8 mg, 0.250 mmol, 1.0 equiv.) in the presence of $[(^tBuCN)_5Ru(H_2O)](BF_4)_2$ (18.0 mg, 25 µmol, 10 mol%), potassium carbonate (69.0 mg, 0.500 mmol, 2.0 equiv.), potassium phenylphosphonate (35.2 mg, 150 µmol, 60 mol%), sodium iodide (37.5 mg, 0.250 mmol, 1.0 equiv.) and anhydrous tetrahydrofuran (500 µL) at 80 °C for 18 hours. Hydrochloric acid (3 M, 3 mL) was added at 20 °C, the reaction mixture stirred for 3 hours and the organics extracted using ethyl acetate (3 x 2 mL). The crude reaction mixture was purified by flash column chromatography (SiO_2, 25 g, biotage isolera, hexane/ethyl acetate – 100/0 to 95/5, 80 mL/min) to give 1-{4-methyl-2-[(trimethylsilyl)methyl]phenyl}ethan-1-one (25 mg, 0.113 mmol, 45%) as a colourless oil.

TLC: R_f = 0.49 (4:1, hexane/ethyl acetate) [UV]

^{1}H NMR: (500 MHz, d_3-CD_3OD)

7.75 (d, *J* = 7.9 Hz, 1H), 7.01 (dd, *J* = 8.0, 1.8 Hz, 1H), 6.97 – 6.86 (m, 1H), 2.61 (s, 2H), 2.54 (s, 3H), 2.33 (s, 3H), -0.07 (s, 9H).

^{13}C NMR: (126 MHz, d_3-CD_3OD)

203.29, 143.93, 143.64, 133.78, 132.76, 132.17, 125.92, 29.47, 26.05, 21.45, -1.42.

IR: (*neat*, cm^{-1})

2953.6 (w), 1677.6 (s), 1258.4 (s), 850.2 (s).

MS: (HRMS - ESI^+)

Found 243.1184 ($C_{13}H_{20}ONaSi$), requires 243.1176.

1-{3-[(Trimethylsilyl)methyl]naphthalen-2-yl}ethan-1-one 4x

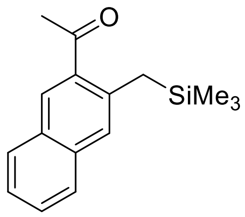

Following general procedure A, (*E*)-*N*,*N*-dimethyl-4-{[1-(naphthalen-2-yl)ethylidene]amino}aniline (72.1 mg, 0.250 mmol) was reacted with trimethylsilylbromomethane (41.8 mg, 0.250 mmol, 1.0 equiv.) in the presence of $[(^tBuCN)_5Ru(H_2O)](BF_4)_2$ (18.0 mg, 25 µmol, 10 mol%), potassium carbonate (69.0 mg, 0.500 mmol, 2.0 equiv.), potassium phenylphosphonate (35.2 mg, 150 µmol, 60 mol%), sodium iodide (37.5 mg, 0.250 mmol, 1 equiv.) and anhydrous tetrahydrofuran (500 µL) at 80 °C for 18 hours. Hydrochloric acid (3 M, 3 mL) was added at 20 °C, the reaction mixture stirred for 3 hours and the organics extracted using ethyl acetate (3 x 2 mL) The crude reaction mixture was purified by flash column chromatography (SiO_2, 25 g, biotage isolera, hexane/ethyl acetate – 100/0 to 90/10, 80 mL/min) to give 1-{3-[(trimethylsilyl)methyl]naphthalen-2-yl}ethan-1-one (30.0 mg, 0.117 mmol, 47%) as a yellow/orange gel.

TLC: R_f = 0.72 (9:1, hexane/ethyl acetate) [UV]

^{1}H NMR: (400 MHz, $CDCl_3$)

8.23 (s, 1H), 7.84 (d, *J* = 8.2 Hz, 1H), 7.72 (d, *J* = 8.2 Hz 1H), 7.52 (ddd, *J* = 8.2, 6.8, 1.3 Hz, 1H), 7.46 (s, 1H), 7.42 (ddd, *J* = 8.1, 6.8, 1.2 Hz, 1H), 2.74 (s, 2H), 2.71 (s, 3H), -0.03 (s, 9H).

^{13}C NMR: (126 MHz, $CDCl_3$)

203.0, 139.6, 136.8, 136.4, 132.6, 131.5, 130.1, 129.8, 129.7, 128.2, 126.8, 31.1, 26.3, 0.0.

MS: (HRMS - ESI$^+$)

Found 257.1363 ($C_{16}H_{21}OSi$), requires 257.1356.

Methyl {(5S,8S,9S,13S)-8-benzyl-5-(*tert*-butyl)-9-hydroxy-14,14-dimethyl-3,6,12-trioxo-11-[(4-(pyridin-2-yl)-3-((trimethylsilyl)methyl)benzyl)amino]-2-oxa-4,7,11-triazapentadecan-13-yl}carbamate 4y

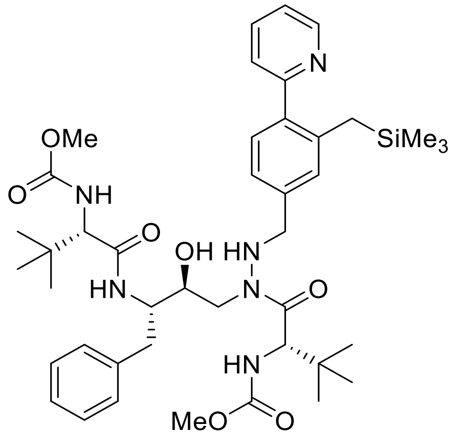

Following general procedure A, methyl {(5S,8S,9S,13S)-8-benzyl-5-(*tert*-butyl)-9-hydroxy-14,14-dimethyl-3,6,12-trioxo-11-[(4-(pyridin-2-yl)benzyl)amino]-2-oxa-4,7,11-triazapentadecan-13-yl}carbamate (25.0 mg, 35.5 µmol) was reacted with trimethylsilylbromomethane (6.0 mg, 35.5 µmol, 1.0 equiv.) in the presence of $[(^tBuCN)_5Ru(H_2O)](BF_4)_2$ (2.5 mg, 3.6 µmol, 10 mol%), potassium carbonate (10.0 mg, 72 µmol, 2.0 equiv.), potassium phenylphosphonate (5.0 mg, 20 µmol, 60 mol%), sodium iodide (5.3 mg, 36.0 µmol, 1.0 equiv.) and anhydrous tetrahydrofuran (70 µL) at 80 °C for 18 hours. *n.b* the inorganic additives and catalyst (except K_2CO_3) were added to the reaction vial as stock solutions in water, and the water removed *in vacuo* before addition of reagents and tetrahydrofuran. The crude reaction mixture was purified by flash column chromatography (SiO_2, 10 g, biotage isolera, hexane/acetone – 90/10 to 50/50, 40 mL/min) to give methyl {(5S,8S,9S,13S)-8-benzyl-5-(*tert*-butyl)-9-hydroxy-14,14-dimethyl-3,6,12-trioxo-11-[(4-(pyridin-2-yl)-3-((trimethylsilyl)methyl)benzyl)amino]-2-oxa-4,7,11-triazapentadecan-13-yl}carbamate (7.0 mg, 8.90 µmol, 25%) as an off-while amorphous solid.

TLC: R_f = 0.35 (3:2, hexane/acetone) [UV/$KMnO_4$]

^{1}H NMR: (400 MHz, CD_2Cl_2)

8.81 – 8.48 (m, 1H), 7.73 (td, *J* = 7.7, 1.9 Hz, 1H), 7.38 – 7.10 (m, 9H), 7.03 (d, *J* = 1.7 Hz, 1H), 6.65 (s, 1H), 6.43 (d, *J* = 9.1 Hz, 1H), 5.25 (d, *J* = 8.2 Hz, 1H), 4.84 (s, 1H), 4.08 – 3.94 (m, 2H), 3.92 – 3.84 (m, 1H), 3.77 (d, *J* = 8.5 Hz, 1H), 3.69 – 3.56 (m, 8H), 2.97 – 2.75 (m, 3H), 2.62 – 2.28 (m, 3H), 0.92 – 0.67 (m, 18H), -0.22 (s, 9H).

^{13}C NMR: (101 MHz, CD_2Cl_2)

171.3, 170.9, 160.9, 157.2, 149.2, 140.0, 138.9, 138.9, 136.5, 135.8, 130.8, 129.8, 128.6, 126.5, 125.1, 124.8, 121.9, 67.9, 64.0, 62.9, 61.9, 61.7, 52.7, 52.6, 52.4, 39.2, 34.6, 34.2, 26.8, 26.7, 26.5, 23.8, -1.4.

MS: (HRMS - ESI$^+$)

Found 813.4343 ($C_{42}H_{62}O_7N_6NaSi$), requires 813.4341.

2-{4-(Methylsulfonyl)-2-[(trimethylsilyl)methyl]phenyl}imidazo[1,2-a]pyridine 4z

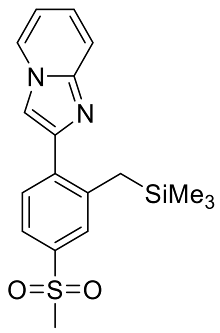

Following general procedure A, 2-[4-(methylsulfonyl)phenyl]imidazo[1,2-a]pyridine (50.0 mg, 0.184 mmol) was reacted with trimethylsilylbromomethane (30.8 mg, 0.184 mmol, 1.0 equiv.) in the presence of $[({}^{t}BuCN)_5Ru(H_2O)](BF_4)_2$ (12.7 mg, 18 μmol, 10 mol%), potassium carbonate (50.9 mg, 0.368 mmol, 2.0 equiv.), potassium phenylphosphonate (26.0 mg, 110 μmol, 60 mol%), sodium iodide (27.6 mg, 0.184 mmol, 1.0 equiv.) and anhydrous tetrahydrofuran (360 μL) at 80 °C for 18 hours. The crude reaction mixture was purified by flash column chromatography (SiO_2, 25 g, biotage isolera, hexane/ethyl acetate – 75/25 to 30/70, 80 mL/min) to give 2-{4-(methylsulfonyl)-2-[(trimethylsilyl)methyl]phenyl}imidazo[1,2-a]pyridine (23.0 mg, 65.0 μmol, 36%) as a yellow oil.

TLC: R_f = 0.34 (2:3) (hexane/ethyl acetate) [UV]

^{1}H NMR: (500 MHz, $CDCl_3$)

8.17 (d, *J* = 6.9 Hz, 1H), 7.79 (d, *J* = 8.0 Hz, 1H), 7.73 (s, 1H), 7.71 – 7.65 (m, 2H), 7.65 – 7.61 (m, 1H), 7.21 (ddd, *J* = 9.2, 6.7, 1.3 Hz, 1H), 6.83 (td, *J* = 6.7, 1.2 Hz, 1H), 3.06 (s, 3H), 2.80 (s, 2H), -0.15 (s, 9H).

^{13}C NMR: (126 MHz, $CDCl_3$)

146.6, 146.4, 143.1, 140.5, 138.9, 132.7, 129.9, 127.1, 126.4, 124.3, 119.3, 114.2, 112.5, 46.1, 26.3.

IR: (*neat*, cm^{-1})

2952.6 (w), 1305.1 (s), 1142.1 (s), 849.7 (s).

MS: (HRMS - ESI^+)

Found 381.1057 ($C_{18}H_{22}O_2N_2NaSSi$), requires 381.1063.

2-{6-Methyl-2-[4-methyl-2-((trimethylsilyl)methyl)phenyl]imidazo[1,2-a]pyridin-3-yl}-1-(piperidin-1-yl)ethan-1-one 4aa

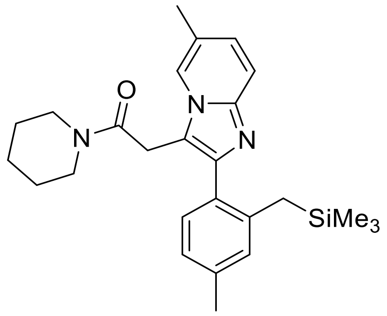

Following general procedure A, [6-methyl-2-(*p*-tolyl)imidazo[1,2-a]pyridin-3-yl](piperidin-1-yl)methanone (83.4 mg, 0.250 mmol) was reacted with trimethylsilylbromomethane (41.8 mg, 0.250 mmol, 1.0 equiv.) in the presence of $[(^{t}BuCN)_5Ru(H_2O)](BF_4)_2$ (18.0 mg, 25 μmol, 10 mol%), potassium carbonate (69.0 mg, 0.500 mmol, 2.0 equiv.), potassium phenylphosphonate (35.2 mg, 150 μmol, 60 mol%), sodium iodide (37.5 mg, 0.500 mmol, 1 equiv.) and anhydrous tetrahydrofuran (500 μL) at 80 °C for 18 hours. The crude reaction mixture was purified by flash column chromatography (SiO_2, 25 g, biotage isolera, hexane/ethyl acetate – 90/10 to 10/90, 80 mL/min) to give 2-{6-methyl-2-[4-methyl-2-((trimethylsilyl)methyl)phenyl]imidazo[1,2-a]pyridin-3-yl}-1-(piperidin-1-yl)ethan-1-one (17.0 mg, 39.0 μmol, 16%) as a brown oil.

TLC: R_f = 0.50 (ethyl acetate) [UV]

IR: (*neat*, cm^{-1})

2938.4 (m), 1639.3 (s), 1442.9 (m), 1225.3 (s), 850.2 (s).

^{1}H NMR: (400 MHz, d_3-CD_3CN)

7.85 (s, 1H), 7.40 (dd, *J* = 9.1, 1.0 Hz, 1H), 7.19 – 6.77 (m, 4H), 3.80 (s, 2H), 3.55 – 3.47 (m, 2H), 3.43 – 3.37 (m, 2H), 2.40 (s, 2H), 2.34 – 2.32 (m, 6H), 1.67 – 1.59 (m, 2H), 1.55 – 1.45 (m, 4H), -0.24 (s, 9H).

^{13}C NMR: (101 MHz, d_3-CD_3CN)

167.4, 145.5, 144.0, 141.5, 138.1, 131.6, 131.2, 130.6, 127.6, 125.5, 123.3, 121.9, 117.2, 116.9, 47.4, 43.6, 29.9, 27.2, 26.5, 25.1, 24.1, 21.3, 18.24, -1.4.

7-Chloro-1-methyl-5-{2-[(trimethylsilyl)methyl]phenyl}-1,3-dihydro-2H-benzo[e][1,4]diazepin-2-one 4ab

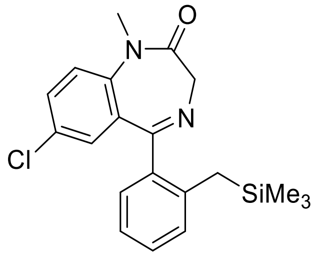

Following general procedure A, 7-chloro-1-methyl-5-phenyl-1,3-dihydro-2H-benzo[e][1,4]diazepin-2-one (25.0 mg, 87.8 µmol) was reacted with trimethylsilylbromomethane (14.6 mg, 87.8 µmol, 1.0 equiv.) in the presence of $[(^tBuCN)_5Ru(H_2O)](BF_4)_2$ (6.2 mg, 8.8 µmol, 10 mol%), potassium carbonate (23.3 mg, 0.176 mmol, 2.0 equiv.), potassium phenylphosphonate (12.4 mg, 52.8 µmol, 60 mol%), sodium iodide (13.1 mg, 87.8 µmol, 1.0 equiv.) and anhydrous tetrahydrofuran (180 µL) at 80 °C for 18 hours. The crude reaction mixture was purified by flash column chromatography (SiO_2, 25 g, biotage isolera, hexane/ethyl acetate – 100/0 to 60/40, 80 mL/min) to give 7-chloro-1-methyl-5-{2-[(trimethylsilyl)methyl]phenyl}-1,3-dihydro-2H-benzo[e][1,4]diazepin-2-one (13 mg, 35.1 µmol, 40%) as a pale-yellow wax.

TLC: R_f = 0.57 (1:1) (hexane/ethyl acetate) [UV]

^{1}H NMR: (500 MHz, $CDCl_3$)

7.45 (dd, *J* = 8.8, 2.5 Hz, 1H), 7.32 – 7.26 (m, 2H), 7.24 (s, 1H), 7.15 (td, *J* = 7.5, 1.2 Hz, 1H), 7.04 – 6.98 (dd, *J* = 6.6, 1.9 Hz, 2H), 4.84 (d, *J* = 10.9 Hz, 1H), 3.77 (d, *J* = 11.0 Hz, 1H), 3.41 (s, 3H), 1.81 (d, *J* = 13.5 Hz, 1H), 1.69 (d, *J* = 13.5 Hz, 1H), -0.16 (s, 9H).

^{13}C NMR: (126 MHz, $CDCl_3$)

172.6, 171.3, 143.1, 140.9, 138.7, 133.5, 132.8, 131.8, 131.1, 131.0, 130.8, 130.3, 125.7, 123.7, 58.0, 36.2, 25.6, 0.0.

IR: (*neat*, cm^{-1})

2924.0 (w), 1682.6 (s), 1481.3 (m), 914.2 (s).

MS: (HRMS - ESI$^+$)

Found 393.1155 ($C_{20}H_{23}ON_2NaClSi$), requires 393.1160.

3-Methyl-2-{2-[(trimethylsilyl)methyl]phenylpyridine}

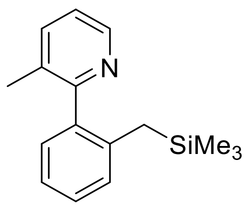

Following general procedure A, 3-methyl-2-phenylpyridine (169.2 mg, 1.00 mmol) was reacted with trimethylsilylbromomethane (167.1 mg, 1.00 mmol, 1.0 equiv.) in the presence of $[(^{t}BuCN)_5Ru(H_2O)](BF_4)_2$ (70.8 mg, 100 μmol, 10 mol%), potassium carbonate (276.4 mg, 2.00 mmol, 2.0 equiv.), potassium phenylphosphonate (70.3 mg, 300 μmol, 30 mol%), sodium iodide (149.9 mg, 1.00 mmol, 1.0 equiv.) and anhydrous tetrahydrofuran (2 mL) at 80 °C for 18 hours. The crude reaction mixture was purified by flash column chromatography (Celite loaded, SiO_2, 50 g, biotage isolera, hexane/ethyl acetate – 100/0 to 70/30, 120 mL/min) to give 3-methyl-2-{2-[(trimethylsilyl)methyl]phenyl}pyridine (89.4 mg, 0.35 mmol, 35%) as a light yellow oil.

TLC: R_f = 0.76 (2:1, hexane/ethyl acetate) [UV]

^{1}H NMR: (400 MHz, $CDCl_3$)

8.58 – 8.39 (m, 1H), 7.62 – 7.48 (m, 1H), 7.26 – 7.20 (m, 1H), 7.20 – 7.06 (m, 4H), 2.15 (s, 3H), 2.01 (s, 2H), -0.17 (s, 9H).

^{13}C NMR: (101 MHz, $CDCl_3$)

159.8, 146.6, 138.6, 138.5, 137.8, 131.6, 129.4, 129.2, 127.6, 124.1, 122.0, 23.4, 19.3, -1.2.

MS: (HRMS - ESI^+)

Found 256.1514 ($C_{16}H_{22}NSi$), requires 256.1516.

Ruthenium-catalysed *meta*-silylmethylation

2-{3-[Bis(trimethylsilyl)methyl]phenyl}pyridine 6a

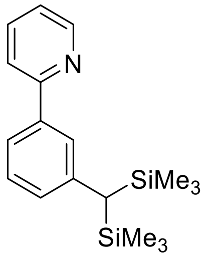

Following general procedure B, 2-phenylpyridine (38.8 mg, 0.25 mmol) was reacted with bis(trimethylsilyl)chloromethane (58.5 mg, 0.300 mmol, 1.2 equiv.) in the presence of $[(^tBuCN)_5Ru(H_2O)](BF_4)_2$ (9.0 mg, 13 μmol, 5 mol%), lithium carbonate (22.2 mg, 0.300 mmol, 1.2 equiv.), lithium cyclohexane carboxylate (3.4 mg, 25 μmol, 10 mol%), water (100 μL) and isopropanol (150 μL) at 100 °C for 4 hours. The crude reaction mixture was purified by flash column chromatography (Celite loaded, SiO_2, 10 g, biotage isolera, hexane/ethyl acetate – 100/0 to 90/10, 25 mL/min) to give *2-{3-[bis(trimethylsilyl)methyl]phenyl}pyridine* (74.0 mg, 0.236 mmol, 94%) as a colourless oil.

TLC: R_f = 0.60 (9:1 hexane/diethylether) [UV]

^{1}H NMR: (400 MHz, $CDCl_3$)

8.77 – 8.64 (m, 1H), 7.83 – 7.65 (m, 3H), 7.55 (t, *J* = 1.9 Hz, 1H), 7.31 (t, *J* = 7.7 Hz, 1H), 7.25 – 7.18 (m, 1H), 7.01 (d, *J* = 7.9, 1H), 1.63 (s, 1H), 0.05 (s, 18H).

^{13}C NMR: (126 MHz, $CDCl_3$)

157.5, 149.1, 143.6, 136.8, 129.2, 128.3, 127.1, 122.0, 121.7, 120.5, 29.5.

MS: (HRMS - ESI^+)

Found 314.1756 ($C_{18}H_{28}NSi_2$), requires 314.1755.

2-{3-[Bis(trimethylsilyl)methyl]phenyl}-3-methylpyridine 6b

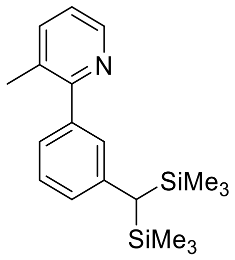

Following general procedure B, 2-phenyl-3-methylpyridine (42.3 mg, 0.250 mmol) was reacted with bis(trimethylsilyl)chloromethane (58.5 mg, 0.300 mmol, 1.2 equiv.) in the presence of $[(^tBuCN)_5Ru(H_2O)](BF_4)_2$ (9.0 mg, 13 µmol, 5 mol%), lithium carbonate (22.2 mg, 0.300 mmol, 1.2 equiv.), lithium cyclohexane carboxylate (3.4 mg, 25 µmol, 10 mol%), water (100 µL) and isopropanol (150 µL) at 100 °C for 4 hours. The crude reaction mixture was purified by flash column chromatography (Celite loaded, SiO_2, 10 g, biotage isolera, hexane/ethyl acetate – 100/0 to 85/15, 25 mL/min) to give *2-{3-[bis(trimethylsilyl)methyl]phenyl}-3-methylpyridine* (18.0 mg, 5.50 µmol, 22%) as a colourless oil.

TLC: R_f = 0.8 (9:1 hexane/ethyl acetate) [UV]

^{1}H NMR: (500 MHz, $CDCl_3$)

8.52 (d, *J* = 4.7, 1H), 7.57 (d, *J* = 7.7, 1H), 7.29 (t, *J* = 8.4, 1H), 7.22 – 7.13 (m, 2H), 7.02 (t, *J* = 1.7 Hz, 1H), 6.97 (d, *J* = 7.7, 1H), 2.32 (s, 3H), 1.56 (s, 1H), 0.04 (s, 18H).

^{13}C NMR: (126 MHz, $CDCl_3$)

158.9, 146.3, 142.6, 139.9, 138.3, 130.7, 127.9 (2 x resonances), 123.9, 121.7, 29.4, 19.9, 0.00.

MS: (HRMS - ESI$^+$)

Found 328.1912 ($C_{19}H_{30}NSi_2$), requires 328.1911.

2-{3-[Bis(trimethylsilyl)methyl]-4-methylphenyl}pyridine 6c

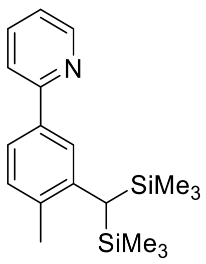

Following general procedure B, 2-(4-methylphenyl)pyridine (42.3 mg, 0.250 mmol) was reacted with bis(trimethylsilyl)chloromethane (58.5 mg, 0.300 mmol, 1.2 equiv.) in the presence of $[(^t\text{BuCN})_5\text{Ru}(\text{H}_2\text{O})](\text{BF}_4)_2$ (9.0 mg, 13 µmol, 5 mol%), lithium carbonate (22.2 mg, 0.300 mmol, 1.2 equiv.), lithium cyclohexane carboxylate (3.4 mg, 25 µmol, 10 mol%), water (100 µL) and isopropanol (150 µL) at 100 °C for 4 hours. The crude reaction mixture was purified by flash column chromatography (neutral Al_2O_3, 40 g, hexane/ethyl acetate – 100/0 to 90/10) to give *2-{3-[bis(trimethylsilyl)methyl]-4-methylphenyl}pyridine* (44.0 mg, 0.134 mmol, 54%) as a colourless oil.

TLC: R_f = 0.80 – Al_2O_3 (9:1) (hexane/diethylether) [UV]

^{1}H NMR: (500 MHz, C_6D_6)

8.60 (ddd, *J* = 4.7, 1.9, 0.9 Hz, 1H), 8.22 (d, *J* = 1.9 Hz, 1H), 7.80 (dd, *J* = 7.8, 1.9 Hz, 1H), 7.53 (dt, *J* = 8.0, 1.1 Hz, 1H), 7.18 (d, *J* = 7.8 Hz, 1H), 7.10 (td, *J* = 7.5, 1.8 Hz, 1H), 6.61 (ddd, *J* = 7.5, 4.8, 1.1 Hz, 1H), 2.15 (s, 3H), 1.81 (s, 1H), 0.14 (s, 18H).

^{13}C NMR: (126 MHz, C_6D_6)

157.6, 149.9, 142.0, 137.1, 136.0, 135.8, 130.8, 126.7, 122.1, 121.3, 119.2, 24.1, 20.8, 0.2.

MS: (HRMS - ESI$^+$)

Found 328.1913 ($C_{19}H_{30}NSi_2$), requires 328.1911.

2-{2-[Bis(trimethylsilyl)methyl]-[1,1'-biphenyl]-4-yl}pyridine 6d

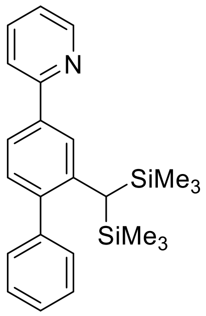

Following general procedure B, 2-[(1,1'-biphenyl)-4-yl]-pyridine (57.8 mg, 0.250 mmol) was reacted with bis(trimethylsilyl)chloromethane (58.5 mg, 0.300 mmol, 1.2 equiv.) in the presence of $[(^{t}BuCN)_5Ru(H_2O)](BF_4)_2$ (9.0 mg, 13 µmol, 5 mol%), lithium carbonate (22.2 mg, 0.300 mmol, 1.2 equiv.), lithium cyclohexane carboxylate (3.4 mg, 25 µmol, 10 mol%), water (100 µL) and isopropanol (150 µL) at 100 °C for 4 hours. The crude reaction mixture was purified by flash column chromatography (SiO_2, 10 g, biotage isolera, hexane/ethyl acetate – 100/0 to 98/2, 40 mL/min) to give *2-{2-[bis(trimethylsilyl)methyl]-[1,1'-biphenyl]-4-yl}pyridine* (66.0 mg, 0.169 mmol, 68%) as a yellow amorphous solid.

TLC: R_f = 0.60 (4:1) (hexane/ethyl acetate) [UV]

mpt: 72-74 °C (hexane)

^{1}H NMR: (500 MHz, C_6D_6)

8.61 (ddd, *J* = 4.7, 1.9, 1.0 Hz, 1H), 8.37 (d, *J* = 1.9 Hz, 1H), 7.82 (dd, *J* = 8.0, 1.9 Hz, 1H), 7.54 (dt, *J* = 8.1, 1.0 Hz, 1H), 7.43 – 7.35 (m, 2H), 7.32 (d, *J* = 7.9 Hz, 1H), 7.28 – 7.22 (m, 2H), 7.15 – 7.08 (m, 2H), 6.63 (ddd, *J* = 7.5, 4.8, 1.1 Hz, 1H), 2.23 (s, 1H), 0.12 (s, 18H).

^{13}C NMR: (101 MHz, $CDCl_3$)

157.1, 149.8, 142.5, 142.1, 141.1, 138.0, 135.9, 130.8, 129.7, 127.9, 127.0, 126.7, 121.8, 121.4, 119.3, 24.0, 0.2.

MS: (HRMS - ESI$^+$)

Found 390.2080 ($C_{24}H_{32}NSi_2$), requires 390.2068.

2-(4-Fluoro-3-methylphenyl)pyridine 6e

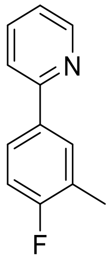

Following general procedure B, 4-fluorophenylpyridine (43.3 mg, 0.250 mmol) was reacted with bis(trimethylsilyl)chloromethane (58.5 mg, 0.300 mmol, 1.2 equiv.) in the presence of $[(^tBuCN)_5Ru(H_2O)](BF_4)_2$ (9.0 mg, 13 µmol, 5 mol%), lithium carbonate (22.2 mg, 0.300 mmol, 1.2 equiv.), lithium cyclohexane carboxylate (3.4 mg, 25 µmol, 10 mol%), water (100 µL) and isopropanol (150 µL) at 100 °C for 4 hours. The reaction solvent was removed *in vacuo* (500 to 10 mbar, 40 °C) and tetra-*n*-butylammonium fluoride (750 µL, 0.750 mmol, 1.0 M in THF) and anhydrous THF (500 µL) added. The mixture was stirred for 1.5 hours at room temperature, the solvent removed *in vacuo* (400 to 30 mbar, 40 °C) and the product purified by flash column chromatography (SiO_2, 10 g, biotage isolera, hexane/ethyl acetate – 100/0 to 94/6, 25 mL/min) to give *2-(4-fluoro-3-methylphenyl)pyridine* (39.0 mg, 0.208 mmol, 83%) as a yellow oil.

TLC: R_f = 0.40 (9:1) (hexane/diethylether) [UV]

^{1}H NMR: (400 MHz, C_6D_6)

8.56 (ddd, *J* = 4.8, 1.9, 1.0 Hz, 1H), 7.94 (ddd, *J* = 7.5, 2.3, 0.9 Hz, 1H), 7.83 – 7.71 (m, 1H), 7.21 (dt, *J* = 8.0, 1.1 Hz, 1H), 7.10 (td, *J* = 7.7, 1.9 Hz, 1H), 6.90 (t, *J* = 9.0 Hz, 1H), 6.65 (ddd, *J* = 7.5, 4.8, 1.1 Hz, 1H), 2.10 (d, *J* = 2.0 Hz, 3H).

^{13}C NMR: (101 MHz, C_6D_6)

162.1 (d, *J* = 245 Hz), 156.4, 149.6, 135.9, 135.3 (d, *J* = 4 Hz), 130.3 (d, *J* = 5.4 Hz), 126.0 (d, *J* = 8.3 Hz), 121.4, 119.4, 115.0 (d, *J* = 23 Hz), 14.2 (d, *J* = 4 Hz).

^{19}F NMR: (376 MHz, C_6D_6)

–117.6.

2-(4-Methoxy-3-methylphenyl)pyridine 6f

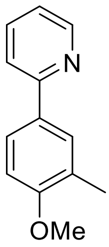

Following general procedure B, 2-(4-methoxyphenyl)pyridine (46.3 mg, 0.250 mmol) was reacted with bis(trimethylsilyl)chloromethane (58.5 mg, 0.300 mmol, 1.2 equiv.) in the presence of $[(^{t}BuCN)_5Ru(H_2O)](BF_4)_2$ (9.0 mg, 13 µmol, 5 mol%), lithium carbonate (22.2 mg, 0.300 mmol, 1.2 equiv.), lithium cyclohexane carboxylate (3.4 mg, 25 µmol, 10 mol%), water (100 µL) and isopropanol (150 µL) at 100 °C for 4 hours. The solvent was removed *in vacuo* (500 mbar to 30 mbar, 40 °C), tetrabutylammonium fluoride solution (750 µL, 0.75 mmol, 3 equiv.) added and the reaction mixture stirred for 1.5 hours. The crude reaction mixture was purified by flash column chromatography (SiO_2, 10 g, biotage isolera, hexane/ethyl acetate – 100/0 to 93/7, 40 mL/min) to give methyl 2-(4-methoxy-3-methylphenyl)pyridine (45.0 mg, 0.226 mmol, 90%) as a colourless oil.

TLC: R_f = 0.46 (4:1) (hexane/ethyl acetate) [UV]

^{1}H NMR: (500 MHz, $CDCl_3$)

8.65 (d, *J* = 4.9 Hz, 1H), 7.85 – 7.76 (m, 2H), 7.73 – 7.62 (m, Hz, 2H), 7.16 (dd, *J* = 5.8, 4.9 Hz, 1H), 6.91 (d, *J* = 8.4 Hz, 1H), 3.89 (s, 3H), 2.30 (s, 3H).

^{13}C NMR: (101 MHz, $CDCl_3$)

158.7, 157.4, 149.5, 136.6, 131.5, 129.2, 126.9, 125.5, 121.3, 119.9, 110.0, 55.4, 16.4.

2-{3-[Bis(trimethylsilyl)methyl]-4-[(methoxymethoxy)methyl]phenyl}pyridine 6g

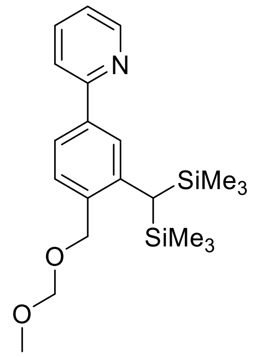

Following general procedure B, 2-{4-[(methoxymethoxy)methyl]phenyl}pyridine (57.3 mg, 0.250 mmol) was reacted with bis(trimethylsilyl)chloromethane (58.5 mg, 0.300 mmol, 1.2 equiv.) in the presence of $[(^t\text{BuCN})_5\text{Ru}(\text{H}_2\text{O})](\text{BF}_4)_2$ (9.0 mg, 13 µmol, 5 mol%), lithium carbonate (22.2 mg, 0.300 mmol, 1.2 equiv.), lithium cyclohexane carboxylate (3.4 mg, 25 µmol, 10 mol%), water (100 µL) and isopropanol (150 µL) at 100 °C for 4 hours. The crude reaction mixture was purified by flash column chromatography (SiO_2, 10 g, biotage isolera, hexane/ethyl acetate – 100/0 to 95/5, 40 mL/min) to give *2-{3-[bis(trimethylsilyl)methyl)-4-((methoxymethoxy)methyl]phenyl}pyridine* (11.0 mg, 28.4 µmol, 11%) as a colourless oil.

TLC: R_f = 0.60 (1:1) (hexane/diethylether) [UV]

^{1}H NMR: (400 MHz, $CDCl_3$)

8.68 (ddd, *J* = 4.8, 1.8, 0.9 Hz, 1H), 7.76 – 7.70 (m, 2H), 7.70 – 7.58 (m, 2H), 7.42 (d, *J* = 8.0 Hz, 1H), 7.20 (ddd, *J* = 7.3, 4.9, 1.2 Hz, 1H), 4.68 (s, 2H), 4.56 (s, 2H), 3.44 (s, 3H), 1.93 (d, *J* = 9.9 Hz, 1H), 0.07 (s, 18H).

^{13}C NMR: (101 MHz, $CDCl_3$)

157.2, 149.4, 142.4, 138.1, 136.2, 134.7, 129.8, 126.6, 121.5, 121.5, 119.8, 94.9, 67.2, 55.1, 23.0.

MS: (HRMS - ESI$^+$)

Found 410.1941 ($C_{21}H_{33}O_2NNaSi_2$), requires 410.1942.

Methyl 6-{3-[bis(trimethylsilyl)methyl]phenyl}nicotinate 6h

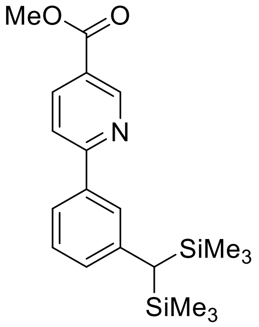

Following general procedure B, methyl 6-phenylnicotinate (53.3 mg, 0.250 mmol) was reacted with bis(trimethylsilyl)chloromethane (58.5 mg, 0.300 mmol, 1.2 equiv.) in the presence of $[(^{t}BuCN)_5Ru(H_2O)](BF_4)_2$ (9.0 mg, 13 μmol, 5 mol%), lithium carbonate (22.2 mg, 0.300 mmol, 1.2 equiv.), lithium cyclohexane carboxylate (3.4 mg, 25 μmol, 10 mol%), water (100 μL) and isopropanol (150 μL) at 100 °C for 4 hours. The crude reaction mixture was purified by flash column chromatography (SiO_2, 10 g, biotage isolera, hexane/ethyl acetate – 100/0 to 94/6, 40 mL/min) to give methyl *6-{3-[bis(trimethylsilyl)methyl]phenyl}nicotinate* (70.0 mg, 0.188 mmol, 75%) as a colourless amorphous solid.

TLC: R_f = 0.70 (9:1) (hexane/ethyl acetate) [UV]

mpt: 74 – 76 °C (ethyl acetate)

^{1}H NMR: (400 MHz, $CDCl_3$)

9.27 (dd, *J* = 2.3, 0.8 Hz, 1H), 8.32 (dd, *J* = 8.3, 2.2 Hz, 1H), 7.82 – 7.68 (m, 2H), 7.63 (t, *J* = 1.9 Hz, 1H), 7.32 (t, *J* = 7.7 Hz, 1H), 7.05 (ddd, *J* = 7.6, 2.0, 1.1 Hz, 1H), 3.97 (s, 3H), 1.71 – 1.58 (m, 1H), 0.06 (s, 18H).

^{13}C NMR: (101 MHz, $CDCl_3$)

165.8, 161.2, 150.8, 143.8, 137.8, 137.6, 130.0, 128.4, 127.5, 123.7, 122.3, 119.6, 52.1, 29.6, 0.0.

IR: (*neat*, cm^{-1})

2953.3 (w), 1718.6 (m), 1593.3 (m), 794.3 (s).

MS: (HRMS - ESI^+)

Found 394.1624 ($C_{20}H_{29}O_2NNaSi_2$), requires 394.1629.

Isopropyl 6-{3-[bis(trimethylsilyl)methyl]phenyl}nicotinate 6i

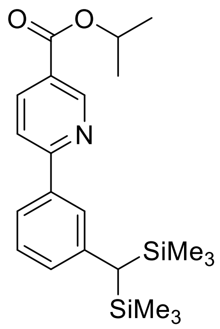

Following general procedure B, isopropyl 6-phenylnicotinate (60.3 mg, 0.250 mmol) was reacted with bis(trimethylsilyl)chloromethane (58.5 mg, 0.300 mmol, 1.2 equiv.) in the presence of $[(^tBuCN)_5Ru(H_2O)](BF_4)_2$ (9.0 mg, 13 µmol, 5 mol%), lithium carbonate (22.2 mg, 0.300 mmol, 1.2 equiv.), lithium cyclohexane carboxylate (3.4 mg, 25 µmol, 10 mol%), water (100 µL) and isopropanol (150 µL) at 100 °C for 4 hours. The crude reaction mixture was purified by flash column chromatography (SiO_2, 10 g, biotage isolera, hexane/ethyl acetate – 100/0 to 95/5, 40 mL/min) to give *isopropyl 6-{3-[bis(trimethylsilyl)methyl]phenyl}nicotinate* (77.0 mg, 0.193 mmol, 77%) as a colourless oil.

TLC: R_f = 0.88 (4:1) (hexane/ethyl acetate) [UV]

^{1}H NMR: (400 MHz, $CDCl_3$)

9.27 (dd, *J* = 2.2, 0.9 Hz, 1H), 8.31 (dd, *J* = 8.3, 2.2 Hz, 1H), 7.81 – 7.67 (m, 2H), 7.63 (t, *J* = 1.9 Hz, 1H), 7.32 (t, *J* = 7.7 Hz, 1H), 7.07 – 7.00 (m, 1H), 5.30 (hept., *J* = 6.2 Hz, 1H), 1.66 – 1.60 (m, 1H), 1.40 (d, *J* = 6.3 Hz, 6H), 0.06 (s, 18H).

^{13}C NMR: (101 MHz, $CDCl_3$)

164.8, 161.0, 150.8, 143.8, 137.9, 137.5, 128.4, 124.4, 122.3, 119.5, 68.6, 29.5, 21.8, 0.0.

MS: (HRMS - ESI$^+$)

Found 422.1945 ($C_{22}H_{33}O_2NNaSi_2$), requires 422.1942.

(*S*)-4-{2-[(t*ert*-Butoxycarbonyl)amino]-3-methoxy-3-oxopropyl}phenyl 6-{3-[bis(trimethylsilyl)methyl]phenyl}nicotinate 6j

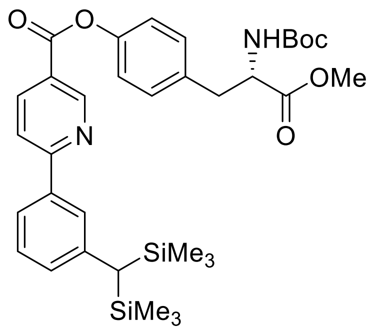

Following general procedure B, (*S*)-4-{2-[(*tert*-butoxycarbonyl)amino]-3-methoxy-3-oxopropyl}phenyl 6-phenylnicotinate (119.1 mg, 0.250 mmol) was reacted with bis(trimethylsilyl)chloromethane (58.5 mg, 0.300 mmol, 1.2 equiv.) in the presence of $[(^tBuCN)_5Ru(H_2O)](BF_4)_2$ (9.0 mg, 13 µmol, 5 mol%), lithium carbonate (22.2 mg, 0.300 mmol, 1.2 equiv.), lithium cyclohexane carboxylate (3.4 mg, 25 µmol, 10 mol%), water (100 µL) and isopropanol (150 µL) at 100 °C for 4 hours. The crude reaction mixture was purified by flash column chromatography (SiO_2, 10 g, biotage isolera, hexane/ethyl acetate – 100/0 to 92/8, 25 mL/min) to give *(S)-4-{2-[(tert-butoxycarbonyl)amino]-3-methoxy-3-oxopropyl}phenyl 6-{3-[bis(trimethylsilyl)methyl]phenyl}nicotinate* (72.0 mg, 0.113 mmol, 45%) as a colourless amorphous solid.

TLC: R_f = 0.48 (9:1) (hexane/ethyl acetate) [UV]

mpt: 56 – 58 °C (ethyl acetate)

^{1}H NMR: (400 MHz, $CDCl_3$)

9.42 (dd, *J* = 2.2, 0.8 Hz, 1H), 8.45 (dd, *J* = 8.4, 2.3 Hz, 1H), 7.83 (dd, *J* = 8.4, 0.9 Hz, 1H), 7.77 (ddd, *J* = 7.8, 1.8, 1.1 Hz, 1H), 7.68 (t, *J* = 1.9 Hz, 1H), 7.35 (t, *J* = 7.7 Hz, 1H), 7.24 – 7.12 (m, 4H), 7.13 – 7.00 (m, 1H), 5.02 (d, *J* = 8.3 Hz, 1H), 4.61 (q, *J* = 7.7 Hz, 1H), 3.74 (s, 3H), 3.13 (qd, *J* = 13.9, 5.9 Hz, 2H), 1.65 (s, 1H), 1.44 (s, 9H), 0.07 (s, 18H).

^{13}C NMR: (101 MHz, $CDCl_3$)

172.0, 163.8, 161.8, 154.9, 151.3, 149.5, 144.0, 138.1, 137.7, 133.8, 130.2, 128.5, 127.6, 123.2, 122.4, 121.5, 119.7, 79.9, 54.2, 52.1, 37.6, 29.6, 28.1, 0.0.

IR: (*neat*, cm^{-1})

2952.0 (w), 1753.1 (m), 1711.3 (m), 1592.5 (m), 1164.0 (m), 837.6 (s).

MS: (HRMS - ESI$^+$)

Found 657.2786 ($C_{34}H_{46}O6N_2NaSi_2$), requires 657.2787.

{6-[3-(Bis(trimethylsilyl)methyl)phenyl]pyridin-3-yl}(piperidin-1-yl)methanone 6k

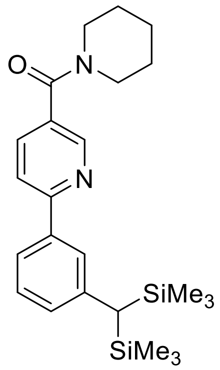

Following general procedure B, (6-phenylpyridin-3-yl)-piperidin-1-ylmethanone (66.6 mg, 0.250 mmol) was reacted with bis(trimethylsilyl)chloromethane (58.5 mg, 0.300 mmol, 1.2 equiv.) in the presence of $[(^{t}BuCN)_5Ru(H_2O)](BF_4)_2$ (9.0 mg, 13 µmol, 5 mol%), lithium carbonate (22.2 mg, 0.300 mmol, 1.2 equiv.), lithium cyclohexane carboxylate (3.4 mg, 25 µmol, 10 mol%), water (100 µL) and isopropanol (150 µL) at 100 °C for 4 hours. The crude reaction mixture was purified by flash column chromatography (SiO_2, 10 g, biotage isolera, hexane/ethyl acetate – 100/0 to 90/10, 40 mL/min) to give *{6-[3-(bis(trimethylsilyl)methyl)phenyl]pyridin-3-yl}(piperidin-1-yl)methanone* (74.0 mg, 0.174 mmol, 70%) as a colourless oil.

TLC: R_f = 0.31 (4:1) (hexane/ethyl acetate) [UV]

^{1}H NMR: (400 MHz, $CDCl_3$)

8.71 (dd, *J* = 2.2, 0.9 Hz, 1H), 7.81 (dd, *J* = 8.1, 2.2 Hz, 1H), 7.70 (ddd, *J* = 14.4, 8.0, 1.1 Hz, 2H), 7.57 (t, *J* = 1.9 Hz, 1H), 7.31 (t, *J* = 7.7 Hz, 1H), 7.05 – 6.98 (m, 1H), 3.74 (br. s, 2H), 3.44 (br. s, 2H), 1.78 – 1.50 (m, 7H), 0.06 (s, 18H).

^{13}C NMR: (101 MHz, $CDCl_3$)

167.7, 158.6, 147.5, 143.7, 138.1, 135.7, 129.9, 128.4, 122.0, 119.9, 48.8, 43.1, 29.5, 26.5 (br., $\Delta_{1/2}$ = 17 Hz), 25.5 (br., $\Delta_{1/2}$ = 18 Hz), 24.3, 0.0.

MS: (HRMS - ESI$^+$)

Found 447.2279 ($C_{24}H_{36}ON_2NaSi_2$), requires 447.2258.

2-{3-[Bis(trimethylsilyl)methyl]-2,5-difluorophenyl}-5-methylpyridine 6l

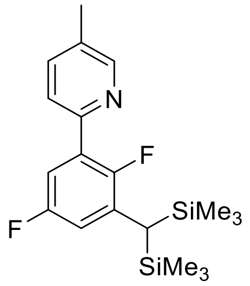

Following a modification of general procedure B on 1 mmol scale, 2-(2,5-difluorophenyl)-5-methylpyridine (205.2 mg, 1.00 mmol) was reacted with bis(trimethylsilyl)chloromethane (262.0 mg, 1.34 mmol, 1.34 equiv.) in the presence of $[(^t\text{BuCN})_5\text{Ru}(H_2O)](BF_4)_2$ (35 mg, 50 μmol, 5 mol%), lithium carbonate (89.0 mg, 1.20 mmol, 1.2 equiv.), methylcyclohexane-1-carboxylic acid (43.0 mg, 0.300 mmol, 30 mol%), water (340 μL) and isopropanol (660 μL) at 100 °C for 12 hours. The reaction was cooled to room temperature, the organics extracted with EtOAc, dried ($MgSO_4$) and the solvent removed *in vacuo*. The crude reaction mixture was purified by flash column chromatography (SiO_2, manual column, hexane/ethyl acetate – 98/2 to 95/5) to give methyl *2-{3-[bis(trimethylsilyl)methyl]-2,5-difluorophenyl}-5-methylpyridine* (250.0 mg, 0.688 mmol, 69%) as a colourless semi-solid.

TLC: R_f = 0.40 (9:1) (hexane/ethyl acetate) [UV]

^{1}H NMR: (500 MHz, C_6D_6)

8.42 (s, 0.7H), 8.40 (s, 0.3H), 8.04 – 7.87 (m, 0.7H), 7.87 – 7.80 (m, 0.3H), 7.73 (d, *J* = 7.8 Hz, 0.7H), 7.67 (d, *J* = 7.8 Hz, 0.3H), 7.00 – 6.89 (m, 1.7H), 6.56 – 6.48 (m, 0.3H), 2.22 (s, 0.7H), 1.83 – 1.74 (m, 3.3H), 0.10 (s, 5H), 0.01 (s, 13H). *n.b compound was rotameric.*

^{13}C NMR: (101 MHz, $CDCl_3$)

160.1, 158.1, 155.6, 153.6, 150.7 (2 x resonances), 150.5, 136.6, 133.6 (dd, *J* = 22.3, 7.8 Hz), 132.3 (dd, *J* = 16.0, 8.5 Hz), 124.0 (d, *J* = 11.8 Hz), 123.8 (d, *J* = 11.8 Hz), 118.7 (dd, *J* = 26.8, 6.9 Hz), 116.1 (dd, *J* = 24.2, 3.6 Hz), 113.3 (dd, *J* = 25.5, 4.1 Hz), 112.9 (dd, *J* = 25.0, 3.6 Hz), 27.0, 19.7, 17.8, 0.3 (2 x resonances). *n.b compound was rotameric.*

^{19}F NMR: (471 MHz, C_6D_6)

-118.5 (dt, *J* = 17.9, 9.2 Hz), -119.2 (dt, *J* = 17.7, 8.8 Hz), -120.8 (d, *J* = 17.5 Hz), -127.3 (d, *J* = 17.1 Hz).

MS: (HRMS - ESI$^+$)

Found 386.1550 ($C_{19}H_{27}F_2NNaSi_2$), requires 386.1542.

2-{3-[Bis(trimethylsilyl)methyl]-2-fluoro-4-methylphenyl}-4,5-dimethylpyridine 6m

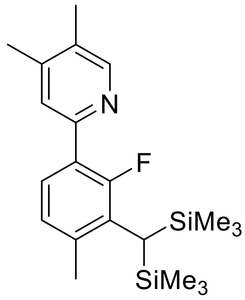

Following a modification of general procedure B, 2-(2-fluoro-4-methylphenyl)-4,5-dimethylpyridine (43.0 mg, 0.200 mmol) was reacted with bis(trimethylsilyl)chloromethane (47.0 mg, 0.240 mmol, 1.20 equiv.) in the presence of $[(^t\text{BuCN})_5\text{Ru}(\text{H}_2\text{O})](\text{BF}_4)_2$ (7.1 mg, 10.0 µmol, 5 mol%), lithium carbonate (17.7 mg, 0.24 mmol, 1.2 equiv.), lithium cyclohexane carboxylate (2.7 mg, 20 µmol, 10 mol%), water (80 µL) and isopropanol (120 µL) at 100 °C for 12 hours. The crude reaction mixture was purified by flash column chromatography (SiO_2, 10 g, biotage isolera, hexane/ethyl acetate – 100/0 to 94/6, 40 mL/min) to give *2-{3-[bis(trimethylsilyl)methyl]-2-fluoro-4-methylphenyl}-4,5-dimethylpyridine* (40.0 mg, 0.107 mmol, 54%) as a colourless amorphous semi-solid.

TLC: R_f = 0.48 (85:15) (hexane/ethyl acetate) [UV]

^{1}H NMR: (500 MHz, $CDCl_3$)

8.41 (s, 1H), 7.50 – 7.39 (m, 2H), 7.02 (d, *J* = 7.9 Hz, 1H), 2.32 (s, 3H), 2.28 (s, 3H), 2.26 (s, 3H), 1.73 (d, *J* = 4.5 Hz, 1H), 0.06 (s, 18H).

^{13}C NMR: (126 MHz, $CDCl_3$)

157.3 (d, *J* = 243.4 Hz), 151.4, 149.3, 44.9, 137.2 (d, *J* = 6.3 Hz), 130.0, 129.1 (d, *J* = 18.3 Hz), 125.4, 124.8, 124.6 (d, *J* = 8.6 Hz), 124.5 (d, *J* = 4.0 Hz), 29.1, 20.6, 20.0, 19.0, 15.6, 0.0.

^{19}F NMR: (471 MHz, $CDCl_3$)

-113.6.

MS: (HRMS - ESI$^+$)

Found 374.2139 ($C_{21}H_{33}FNSi_2$), requires 374.2136.

2-{3-[Bis(trimethylsilyl)methyl]-2-fluorophenyl}pyridine 6n

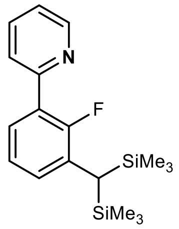

Following general procedure B, 2-(2-fluorophenyl)pyridine (90.2 mg, 0.52 mmol) was reacted with bis(trimethylsilyl)chloromethane (116 mg, 0.60 mmol, 1.2 equiv.) in the presence of $[(^tBuCN)_5Ru(H_2O)](BF_4)_2$ (18 mg, 25 μmol, 5 mol%), lithium carbonate (43.4 mg, 0.590 mmol, 1.2 equiv.), lithium cyclohexane carboxylate (7.2 mg, 50 μmol, 10 mol%), water (200 μL) and isopropanol (300 μL) at 100 °C for 4 hours. The crude reaction mixture was purified by flash column chromatography (SiO_2) to give 2-{3-[bis(trimethylsilyl)methyl]-2-fluorophenyl}pyridine as an colourless oil (75 mg, 0.23 mmol, 44%). *n.b compound was rotameric.*

TLC: R_f = 0.18 (hexane/Et_2O, 25:1) [UV]

^{1}H NMR: (500 MHz, $CDCl_3$)

8.72 (dt, *J* = 5.0, 1.5 Hz, 1H), 7.77 – 7.67 (m, 2H), 7.66 – 7.53 (m, 1H), 7.23 (m, 1H), 7.12 (m, 1H), 7.07 (m, 1H), 6.97 (t, *J* = 7.6 Hz, 0.30H), 2.08 (s, 0.70H), 1.54 – 1.48 (m, 0.3 H), 0.07 (s, 18H). *n.b compound was rotameric*

^{13}C NMR: (126 MHz, $CDCl_3$)

158.62, 156.68, 154.56, 149.82, 136.24, 133.19, 131.07 (d, *J* = 18.8 Hz), 127.59 (d, *J* = 15.2 Hz), 126.14, 124.81 (d, *J* = 8.9 Hz), 123.78, 122.25, 26.46, 18.72, 0.32. *n.b compound was rotameric*

^{19}F NMR (376 MHz, $CDCl_3$)

-115.67, -123.03. *n.b compound was rotameric.*

MS: (HRMS - ESI^+)

Found 354.1467 ($C_{18}H_{26}NFNaSi_2$), requires 354.1480.

2-{3-[Bis(trimethylsilyl)methyl]phenyl}pyrimidine 6o

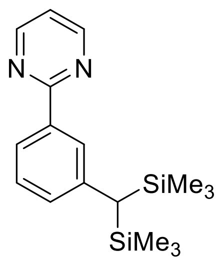

Following general procedure B, 2-phenylpyrimidine (39 mg, 0.250 mmol) was reacted with bis(trimethylsilyl)chloromethane (58.5 mg, 0.300 mmol, 1.2 equiv.) in the presence of $[(^tBuCN)_5Ru(H_2O)](BF_4)_2$ (18.0 mg, 25 µmol, 10 mol%), lithium carbonate (22.2 mg, 0.300 mmol, 1.2 equiv.), lithium cyclohexane carboxylate (3.4 mg, 25 µmol, 10 mol%), water (100 µL) and isopropanol (150 µL) at 100 °C for 4 hours. The crude reaction mixture was purified by flash column chromatography (Celite loaded, SiO_2, 10 g, biotage isolera, hexane/ethyl acetate – 100/0 to 85/15, 25 mL/min) to give *2-{3-[bis(trimethylsilyl)methyl]phenyl}pyrimidine* (39.0 mg, 0.124 mmol, 50%) as a colourless oil.

TLC: R_f = 0.85 (1:1 hexane/ethyl acetate) [UV]

^{1}H NMR: (500 MHz, $CDCl_3$)

8.80 – 8.66 (m, 2H), 8.07 (d, *J* = 7.8, 1H), 8.00 (s, 1H), 7.26 (t, *J* = 7.7 Hz, 1H), 7.13-7.08 (m, 1H), 7.02 (d, *J* = 7.7 Hz, 1H), 1.60 (s, 1H), 0.00 (s, 18H).

^{13}C NMR: (126 MHz, $CDCl_3$)

164.9, 157.0, 143.5, 137.0, 128.1, 123.1, 118.7, 29.5, 0.0.

MS: (HRMS - ESI$^+$)

Found 337.1530 ($C_{17}H_{26}N_2Si_2$), requires 337.1527.

2-{3-[Bis(trimethylsilyl)methyl]phenyl}-4-methoxypyrimidine 6p

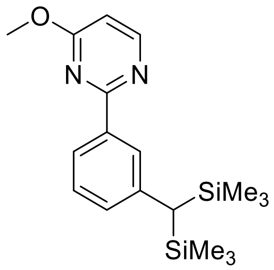

Following general procedure B, 2-phenyl-4-methoxypyrimidine (46.6 mg, 0.250 mmol) was reacted with bis(trimethylsilyl)chloromethane (58.5 mg, 0.300 mmol, 1.2 equiv.) in the presence of $[(^t\text{BuCN})_5\text{Ru}(\text{H}_2\text{O})](\text{BF}_4)_2$ (18.0 mg, 25 μmol, 10 mol%), lithium carbonate (22.2 mg, 0.300 mmol, 1.2 equiv.), lithium cyclohexane carboxylate (3.4 mg, 25 μmol, 10 mol%), water (100 μL) and isopropanol (150 μL) at 100 °C for 4 hours. The crude reaction mixture was purified by flash column chromatography (SiO_2, 10 g, biotage isolera, hexane/ethyl acetate – 100/0 to 98/2, 40 mL/min) to give *2-{3-[bis(trimethylsilyl)methyl]phenyl}-4-methoxypyrimidine* (39 mg, 0.113 mmol, 45%) as a colourless oil.

TLC: R_f = 0.66 (4:1) (hexane/ethyl acetate) [UV]

^{1}H NMR: (400 MHz, $CDCl_3$)

8.49 (d, *J* = 5.7 Hz, 1H), 8.24 – 7.96 (m, 2H), 7.30 (t, *J* = 7.8 Hz, 1H), 7.05 (t, *J* = 7.9, 1.4 Hz, 1H), 6.61 (d, *J* = 5.7 Hz, 1H), 4.07 (s, 3H), 1.64 – 1.61 (m, 1H), 0.06 (s, 18H).

^{13}C NMR: (101 MHz, $CDCl_3$)

169.2, 164.6, 157.2, 143.2, 137.0, 131.0, 128.3, 128.1, 123.1, 105.8, 53.2, 29.4, 0.0.

MS: (HRMS - ESI$^+$)

Found 367.1633 ($C_{18}H_{28}ON_2NaSi_2$), requires 367.1632.

1-{3-[Bis(trimethylsilyl)methyl]phenyl}-1H-pyrazole 6q

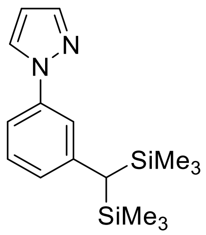

Following general procedure B, 1-phenylpyrazole (36.0 mg, 0.250 mmol) was reacted with bis(trimethylsilyl)chloromethane (58.5 mg, 0.300 mmol, 1.2 equiv.) in the presence of $[(^tBuCN)_5Ru(H_2O)](BF_4)_2$ (18.0 mg, 25 µmol, 10 mol%), lithium carbonate (22.2 mg, 0.300 mmol, 1.2 equiv.), lithium cyclohexane carboxylate (3.4 mg, 25 µmol, 10 mol%), water (100 µL) and isopropanol (150 µL) at 100 °C for 4 hours. The crude reaction mixture was purified by flash column chromatography (SiO_2, 10 g, biotage isolera, hexane/ethyl acetate – 100/0 to 92/8, 25 mL/min) to give *1-{3-[bis(trimethylsilyl)methyl]phenyl}-1H-pyrazole* (37.0 mg, 0.122 mmol, 49%) as a colourless oil.

TLC: R_f = 0.33 (4:1) (hexane/diethylether) [UV]

^{1}H NMR: (500 MHz, C_6D_6)

7.76 – 7.63 (m, 2H), 7.49 (dd, *J* = 2.4, 0.6 Hz, 1H), 7.21 – 7.17 (m, 1H), 7.02 (t, *J* = 7.9 Hz, 1H), 6.75 (d, *J* = 7.6 Hz, 1H), 6.13 (dd, *J* = 2.5, 1.7 Hz, 1H), 1.43 (s, 1H), 0.05 (s, 18H).

^{13}C NMR: (126 MHz, C_6D_6)

145.2, 141.0, 140.8, 129.2, 126.6 (br., $\Delta_{1/2}$ = 25 Hz), 126.1, 119.6 (br., $\Delta_{1/2}$ = 23 Hz), 114.1, 107.6, 30.0, 0.2.

MS: (HRMS - ESI$^+$)

Found 303.1708 ($C_{16}H_{27}N_2Si_2$), requires 303.1707.

1-{3-[Bis(trimethylsilyl)methyl]phenyl}-4-phenyl-1H-pyrazole 6r

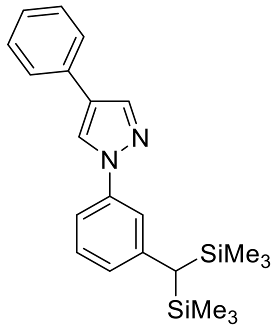

Following general procedure B, 1,4-diphenyl-1H-pyrazole (55.1 mg, 0.25 mmol) was reacted with bis(trimethylsilyl)chloromethane (58.5 mg, 0.300 mmol, 1.2 equiv.) in the presence of $[({}^{t}BuCN)_5Ru(H_2O)](BF_4)_2$ (18.0 mg, 25 μmol, 10 mol%), lithium carbonate (22.2 mg, 0.300 mmol, 1.2 equiv.), lithium cyclohexane carboxylate (3.4 mg, 25 μmol, 10 mol%), water (100 μL) and isopropanol (150 μL) at 100 °C for 4 hours. The crude reaction mixture was purified by flash column chromatography (SiO_2, 10 g, biotage isolera, hexane/ethyl acetate – 100/0 to 90/10, 25 mL/min) to give *1-{3-[bis(trimethylsilyl)methyl]]phenyl}-4-phenyl-1H-pyrazole* (36 mg, 95.1 μmol, 38%) as a colourless oil.

TLC: R_f = 0.78 (4:1) (hexane/diethylether) [UV]

^{1}H NMR: (500 MHz, $CDCl_3$)

8.04 (d, *J* = 0.8 Hz, 1H), 7.91 (d, *J* = 0.8 Hz, 1H), 7.56 – 7.44 (m, 2H), 7.36 – 7.28 (m, 3H), 7.26 (t, *J* = 2.0 Hz, 1H), 7.23 – 7.19 (m, 2H), 6.82 (ddd, *J* = 7.7, 1.8, 1.0 Hz, 1H), 1.55 (s, 1H), 0.00 (s, 18H).

^{13}C NMR: (101 MHz, $CDCl_3$)

145.1, 140.6, 139.7, 138.4, 132.0, 128.7, 126.6, 125.5, 124.5, 123.1, 113.9, 107.1, 29.9, 0.0.

MS: (HRMS - ESI$^+$)

Found 379.2037 ($C_{22}H_{31}N_2Si_2$), requires 379.2020.

1-{3-[Bis(trimethylsilyl)methyl]phenyl}-4-(thiophen-2-yl)-1H-pyrazole 6s

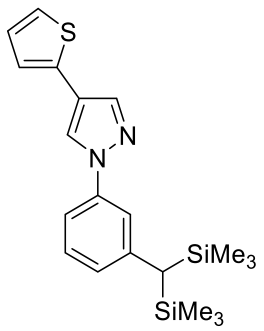

Following general procedure B, 1-phenyl-4-(thiophen-2-yl)-1H-pyrazole (56.6 mg, 0.250 mmol) was reacted with bis(trimethylsilyl)chloromethane (58.5 mg, 0.300 mmol, 1.2 equiv.) in the presence of $[(^{t}BuCN)_5Ru(H_2O)](BF_4)_2$ (18.0 mg, 25 µmol, 10 mol%), lithium carbonate (22.2 mg, 0.300 mmol, 1.2 equiv.), lithium cyclohexane carboxylate (3.4 mg, 25 µmol, 10 mol%), water (100 µL) and isopropanol (150 µL) at 100 °C for 4 hours. The crude reaction mixture was purified by flash column chromatography (SiO_2, 10 g, biotage isolera, hexane/ethyl acetate – 100/0 to 90/10, 25 mL/min) to give *1-{3-[bis(trimethylsilyl)methyl]phenyl}-4-(thiophen-2-yl)-1H-pyrazole* (48.0 mg, 0.125 mmol, 50%) as a colourless oil.

TLC: R_f = 0.63 (4:1) (hexane/ethyl acetate) [UV]

^{1}H NMR: (500 MHz, $CDCl_3$)

7.96 (s, 1H), 7.82 (s, 1H), 7.30 (dd, *J* = 8.2, 2.2 Hz, 1H), 7.27 – 7.20 (m, 2H), 7.15 (d, *J* = 5.1 Hz, 1H), 7.09 (d, *J* = 3.5 Hz, 1H), 6.99 (dd, *J* = 5.0, 3.7 Hz, 1H), 6.85 – 6.79 (m, 1H), 1.57 – 1.52 (m, 1H), 0.00 (s, 18H).

^{13}C NMR: (101 MHz, $CDCl_3$)

145.2, 139.6, 138.5, 134.4, 128.8, 127.5, 123.2, 123.2, 122.8, 118.4, 113.9, 29.9, 0.0. *(2 x broad ^{13}C resonances not observed)*

MS: (HRMS - ESI$^+$)

Found 407.1405 ($C_{20}H_{28}N_2NaSSi_2$), requires 407.1404.

1-{3-[Bis(trimethylsilyl)methyl]phenyl}-5-(phenylthio)-1H-pyrazole 6t

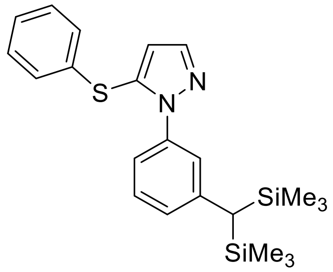

Following general procedure B, 1-phenyl-5-(phenylthio)-1H-pyrazole (63.0 mg, 0.250 mmol) was reacted with bis(trimethylsilyl)chloromethane (58.5 mg, 0.300 mmol, 1.2 equiv.) in the presence of $[(^tBuCN)_5Ru(H_2O)](BF_4)_2$ (18.0 mg, 25 μmol, 10 mol%), lithium carbonate (22.2 mg, 0.300 mmol, 1.2 equiv.), lithium cyclohexane carboxylate (3.4 mg, 25 μmol, 10 mol%), water (100 μL) and isopropanol (150 μL) at 100 °C for 4 hours. The crude reaction mixture was purified by flash column chromatography (SiO_2, 10 g, biotage isolera, hexane/ethyl acetate – 100/0 to 90/10, 25 mL/min) to give *1-{3-[bis(trimethylsilyl)methyl]phenyl}-5-(phenylthio)-1H-pyrazole* (43.0 mg, 0.105 mmol, 42%) as a brown oil.

TLC: R_f = 0.75 (4:1) (hexane/ethyl acetate) [UV]

^{1}H NMR: (500 MHz, $CDCl_3$)

7.96 (d, *J* = 0.8 Hz, 1H), 7.82 (d, *J* = 0.7 Hz, 1H), 7.33 – 7.20 (m, 5H), 7.15 (dd, *J* = 5.1, 1.2 Hz, 1H), 7.08 (dd, *J* = 3.5, 1.2 Hz, 1H), 6.99 (dd, *J* = 5.1, 3.5 Hz, 1H), 6.82 (ddd, *J* = 7.7, 1.8, 1.1 Hz, 1H), 1.54 (d, *J* = 5.1 Hz, 1H), 0.00 (s, 18H).

^{13}C NMR: (126 MHz, $CDCl_3$)

145.2, 139.5, 138.4, 134.3, 128.8, 127.5, 123.3, 123.2, 122.8, 118.4, 114.0, 29.9, 0.0. *(2 x broad ^{13}C resonances not observed)*

MS: (HRMS - ESI^+)

Found 433.1564 ($C_{22}H_{30}N_2NaSSi_2$), requires 433.1560.

N-[2-($1H$-Indol-3-yl)ethyl]-6{[3-(bis(trimethylsilyl)methyl]phenyl}nicotinamide 6u

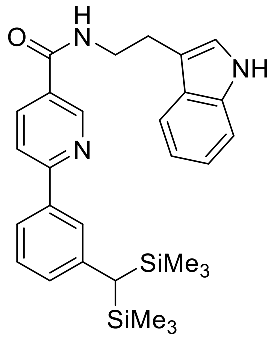

Following general procedure B, *N*-(2-(*1H*-indol-3-yl)ethyl)-6-phenylnicotinamide (85.4 mg, 0.250 mmol) was reacted with bis(trimethylsilyl)chloromethane (58.5 mg, 0.300 mmol, 1.2 equiv.) in the presence of $[(^tBuCN)_5Ru(H_2O)](BF_4)_2$ (9.0 mg, 13 μmol, 5 mol%), lithium carbonate (22.2 mg, 0.300 mmol, 1.2 equiv.), lithium cyclohexane carboxylate (3.4 mg, 25 μmol, 10 mol%), water (100 μL) and isopropanol (150 μL) at 100 °C for 4 hours. The crude reaction mixture was purified by flash column chromatography (SiO_2, 10 g, biotage isolera, hexane/ethyl acetate – 100/0 to 80/20, 25 mL/min) to give *N-[2-(1H-indol-3-yl)ethyl]-6{[3-(bis(trimethylsilyl)methyl]phenyl}nicotinamide* (48.0 mg, 96.0 μmol, 38%) as a yellow amorphous solid.

TLC: R_f = 0.50 (1:1) (hexane/ethyl acetate) [UV]

mpt: 140 – 142 °C (ethyl acetate)

^{1}H NMR: (500 MHz, $CDCl_3$)

8.98 (d, J = 2.2 Hz, 1H), 8.20 (dd, J = 8.2, 2.3 Hz, 1H), 7.86 (d, J = 8.3 Hz, 1H), 7.70 (dd, J = 7.8, 1.6 Hz, 1H), 7.67 – 7.58 (m, 2H), 7.39 – 7.30 (m, 2H), 7.16 – 7.06 (m, 3H), 7.00 (t, J = 7.5 Hz, 1H), 3.71 (t, J = 7.3 Hz, 2H), 3.10 (t, J = 7.3 Hz, 2H), 1.73 (s, 1H), 0.07 (d, J = 0.9 Hz, 18H).

^{13}C NMR: (101 MHz, $CDCl_3$)

168.0, 161.4, 149.4, 145.4, 139.4, 138.2, 137.5, 130.0, 129.7, 128.9, 123.7, 123.5, 122.4, 121.5, 119.6, 119.3, 113.3, 112.3, 42.2, 30.7, 26.3.

IR: (*neat*, cm^{-1})

3305.5 (br.), 2951.6 (w), 1640.1 (m), 1247.8 (s), 837.7 (s).

MS: (HRMS - ESI^+)

Found 522.2393 ($C_{39}H_{37}ON_3NaSi_2$), requires 522.2367.

(3S,8S,9S,10R,13R,14S,17R)-10,13-Dimethyl-17-[(*R*)-6-methylheptan-2-yl]-2,3,4,7,8,9,10,11,12,13,14,15,16,17-tetradecahydro-1H-cyclopenta[a]phenanthren-3-yl 6-{3-[bis(trimethylsilyl)methyl]phenyl}nicotinate 6v

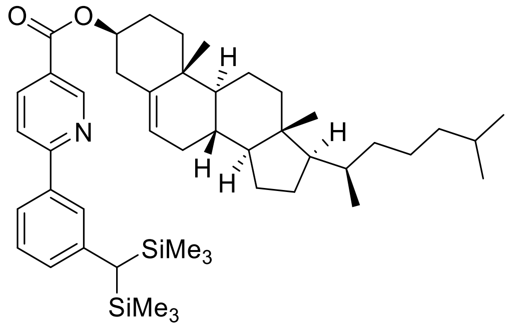

Following general procedure B, (3S,8S,9S,10R,13R,14S,17S)-10,13-dimethyl-17-(5-methylhexyl)-2,3,4,7,8,9,10,11,12,13,14,15,16,17-tetradecahydro-1H-cyclopenta[a]phenanthren-3-yl 6-phenylnicotinate (138.5 mg, 0.250 mmol) was reacted with bis(trimethylsilyl)chloromethane (58.5 mg, 0.300 mmol, 1.2 equiv.) in the presence of $[(^tBuCN)_5Ru(H_2O)](BF_4)_2$ (9.0 mg, 13 µmol, 5 mol%), lithium carbonate (22.2 mg, 0.300 mmol, 1.2 equiv.), lithium cyclohexane carboxylate (3.4 mg, 25 µmol, 10 mol%), water (100 µL) and isopropanol (150 µL) at 100 °C for 4 hours. The crude reaction mixture was purified by flash column chromatography (SiO_2, 10 g, biotage isolera, hexane/ethyl acetate – 100/0 to 92/8, 25 mL/min) to give *(3S,8S,9S,10R,13R,14S,17R)-10,13-dimethyl-17-((R)-6-methylheptan-2-yl)-2,3,4,7,8,9,10,11,12,13,14,15,16,17-tetradecahydro-1H-cyclopenta[a]phenanthren-3-yl 6-(3-(bis(trimethylsilyl)methyl)phenyl)nicotinate* (54 mg, 75.8 µmol, 30%) as a pale yellow gel.

TLC: R_f = 0.57 (9:1) (hexane/ethyl acetate) [UV]

^{1}H NMR: (500 MHz, $CDCl_3$)

9.21 (d, *J* = 2.2 Hz, 1H), 8.26 (dd, *J* = 8.3, 2.2 Hz, 1H), 7.68 (t, *J* = 7.8 Hz, 2H), 7.57 (bs., *J* = 2.1 Hz, 1H), 7.26 (t, *J* = 7.7 Hz, 1H), 7.03 – 6.93 (m, 1H), 5.38 (d, *J* = 5.1 Hz, 1H), 4.91 – 4.71 (m, 1H), 2.43 (d, *J* = 8.2 Hz, 2H), 2.01 – 1.65 (m, 6H), 1.56 – 0.92 (m, 22H), 0.87 (d, *J* = 6.4 Hz, 3H), 0.81 (dd, *J* = 6.8, 2.3 Hz, 6H), 0.64 (s, 3H), 0.00 (18H).

^{13}C NMR: (126 MHz, $CDCl_3$)

164.7, 161.0, 150.8, 143.8, 139.3, 137.9, 137.5, 130.0, 128.4, 127.5, 124.3, 122.8, 122.3, 119.5, 74.8, 56.5, 56.0, 49.9, 42.1, 39.6, 39.3, 38.0, 36.8, 36.5, 36.0, 35.6, 31.8, 31.7, 29.5, 28.0, 27.8, 27.7, 24.1, 23.6, 22.6, 22.4, 20.9, 19.2, 18.5, 11.7, 0.0.

IR: (*neat*, cm^{-1})

2934.8 (m), 1706.0 (s), 1284.5 (m), 1124.0 (m), 742.6 (s).

MS: (HRMS - ESI$^+$)

Found 726.5098 ($C_{46}H_{72}O_2NSi_2$), requires 726.5096.

2-(3-Benzoylphenyl)-*N*-{2-[bis(trimethylsilyl)methyl]-4-[pyridin-2-yl]phenyl}propenamide 6w

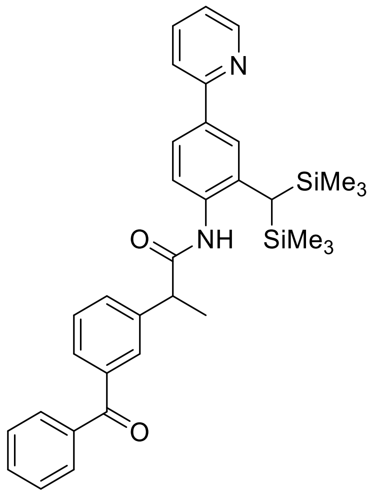

Following general procedure B, 2-(3-benzoylphenyl)-N-(4-(pyridin-2-yl)phenyl)propanamide (101 mg, 0.25 mmol) was reacted with bis(trimethylsilyl)chloromethane (58.5 mg, 0.300 mmol, 1.2 equiv.) in the presence of $[(^{t}BuCN)_5Ru(H_2O)](BF_4)_2$ (9.0 mg, 13 µmol, 5 mol%), lithium carbonate (22.2 mg, 0.300 mmol, 1.2 equiv.), lithium cyclohexane carboxylate (3.4 mg, 25 µmol, 10 mol%), water (100 µL) and isopropanol (150 µL) at 100 °C for 4 hours. The crude reaction mixture was purified by flash column chromatography (SiO_2, 10 g, biotage isolera, hexane/ethyl acetate – 100/0 to 85/15, 40 mL/min) to give *2-(3-benzoylphenyl)-N-{2-[bis(trimethylsilyl)methyl]-4-[pyridin-2-yl]phenyl}propanamide* (61 mg, 0.108 mmol, 43%) as a yellow gel.

TLC: R_f = 0.48 (9:1) (hexane/ethyl acetate) [UV]

^{1}H NMR: (400 MHz, $CDCl_3$)

8.65 (d, *J* = 4.9 Hz, 1H), 7.91 (t, *J* = 1.8 Hz, 1H), 7.82 – 7.76 (m, 4H), 7.74 – 7.65 (m, 3H), 7.65 – 7.53 (m, 4H), 7.52 – 7.44 (m, 2H), 7.18 (dd, *J* = 7.5, 4.9, 1.2 Hz, 1H), 6.72 (s, 1H), 3.89 (q, *J* = 7.2 Hz, 1H), 1.71 (d, *J* = 7.3 Hz, 3H), 0.98 (s, 1H), -0.06 (d, *J* = 2.5 Hz, 18H).

^{13}C NMR: (101 MHz, $CDCl_3$)

195.8, 171.3, 157.0, 149.7, 141.6, 138.4, 137.2, 136.5, 136.0, 135.4, 134.4, 132.6, 131.6, 129.9, 129.7, 129.4, 129.2, 128.3, 127.1, 124.6, 122.63, 121.68, 119.8, 47.9, 27.0, 22.2, 18.0, 0.2, 0.0.

IR: (*neat*, cm^{-1})

2952.3 (w), 1689.8 (m), 1244.4 (s), 758.9 (s).

MS: (HRMS - ESI$^+$)

Found 587.2520 ($C_{34}H_{40}O_2N_2NaSi_2$), requires 587.2521.

Product characterisation – *ortho*-silylmethylation product further reactivity

2-[2-(Pyridin-4-ylmethyl)phenyl]pyridine 10

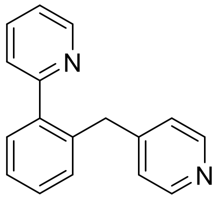

Caesium fluoride (38.0 mg, 0.250 mmol, 1.0 equiv.) was added to a stirred mixture of 2-{2-[(trimethylsilyl)methyl]phenyl}pyridine (60.4 mg, 0.250 mmol), 4-cyanopyridine (26.0 mg, 0.250 mmol, 1.0 equiv.) and 18-crown-6 (72.7 mg, 0.275 mmol, 1.1 equiv.) in dimethylsulfoxide (750 µL). The reaction mixture was stirred at 25 °C for 18 hours before addition of brine (15 mL). The organic phase was extracted with ethyl acetate (3 x 10 mL), dried (Na_2SO_4), the solvent removed *in vacuo* (500 to 25 mbar, 40 °C), and the product purified by flash column chromatography (SiO_2, 10 g, biotage isolera, hexane/ethyl acetate – 90/10 to 1/99, 40 mL/min) to give 2-[2-(pyridin-4-ylmethyl)phenyl]pyridine (34.0 mg, 0.138 mmol, 55%) as a yellow oil. ***Safety note****: the aqueous phase during work-up was monitored by pH indicator paper to ensure that this phase never became acidic.*

TLC: R_f = 0.22 (ethyl acetate) [UV]

^{1}H NMR: (500 MHz, $CDCl_3$)

8.67 – 8.61 (m, 1H), 8.34 (d, *J* = 4.7 Hz, 2H), 7.70 – 7.61 (m, 1H), 7.40 – 7.32 (m, 3H), 7.26 – 7.18 (m, 3H), 6.87 (d, *J* = 4.8 Hz, 2H), 4.16 (d, *J* = 2.2 Hz, 2H).

^{13}C NMR: (126 MHz, $CDCl_3$)

159.7, 150.8, 150.4, 149.5, 149.1, 140.7, 136.9, 136.4, 130.9, 130.1, 128.7, 127.0, 124.1, 121.9, 38.4.

MS: (HRMS - ESI$^+$)

Found 247.1225 ($C_{17}H_{15}N_2$), requires 247.1230.

1-(Furan-2-yl)-2-[2-(pyridin-2-yl)phenyl]ethan-1-ol 12

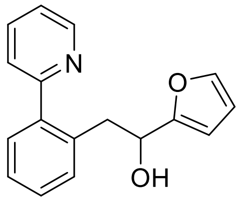

A solution of 2-{2-[(trimethylsilyl)methyl]phenyl}pyridine (60.4 mg, 0.250 mmol) and furfural (24.0 mg, 0.250 mmol) in anhydrous tetrahydrofuran (500 µL) was added to a mixture of tetrabutylammonium chloride (7.0 mg, 25 µmol, 10 mol%) and potassium trimethylsilanolate (2M in THF, 15 µL, 25 µmol, 20 mol%) in anhydrous tetrahydrofuran (500 µL). The reaction mixture was stirred at room temperature for 3 hours before the addition of water (2 mL). The organic phase was extracted with ethyl acetate (3 x 10 mL), dried (Na_2SO_4), the solvent removed *in vacuo* (500 to 150 mbar, 40 °C), and the product purified by flash column chromatography (SiO_2, 25 g, biotage isolera, pentane/diethylether – 100/0 to 10/90, 80 mL/min) to give 1-(furan-2-yl)-2-[2-(pyridin-2-yl)phenyl]ethan-1-ol (54.0 mg, 0.204 mmol, 82%) as a yellow oil.

TLC: R_f = 0.30 (4:1, pentane/diethylether) [UV]

^{1}H NMR: (500 MHz, d_4-CD_3OD)

8.60 (d, J = 5.4, 1H), 7.98 – 7.89 (m, 1H), 7.60 – 7.49 (m, 1H), 7.47 – 7.40 (m, 1H), 7.40 – 7.27 (m, 5H), 6.30 (dd, J = 3.3, 1.8 Hz, 1H), 6.17 – 6.13 (m, 1H), 4.84 – 4.77 (m, 1H), 3.17 (d, J = 7.1 Hz, 2H).

^{13}C NMR: (126 MHz, d_4-CD_3OD)

160.3, 158.4, 158.4, 148.7, 142.8, 141.3, 139.3, 137.6, 132.1, 130.9, 129.9, 127.8, 126.2, 123.7, 111.1, 111.0, 106.6, 69.8, 39.9.

MS: (HRMS - ESI$^+$)

Found 269.1047 ($C_{17}H_{14}N_2Na$), requires 269.1049.

1-[2-(Pyridin-2-yl)phenyl]-2-[4-(trifluoromethyl)phenyl]propan-2-ol 14

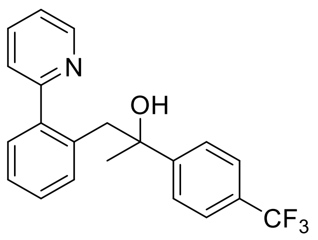

A solution of 2-{2-[(trimethylsilyl)methyl]phenyl}pyridine (60.4 mg, 0.250 mmol) and 4'-(trifluoromethyl)acetophenone (47.2 mg, 0.250 mmol) in anhydrous tetrahydrofuran (500 µL) was added to a mixture of tetrabutylammonium chloride (14 mg, 0.05 mmol, 20 mol%) and potassium trimethylsilanolate (2M in THF, 25 µL, 50.0 µmol, 20 mol%) in anhydrous tetrahydrofuran (500 µL). The reaction mixture was heated at 70 °C for 18 hours, cooled to room temperature before the addition of water (2 mL). The organic phase was extracted with ethyl acetate (3 x 3 mL), dried (Na_2SO_4), the solvent removed *in vacuo* (500 to 150 mbar, 40 °C) and the product purified by flash column chromatography (SiO_2, 25 g, biotage isolera, pentane/diethylether – 100/0 to 0/100, 80 mL/min) to give 1-[2-(pyridin-2-yl)phenyl]-2-[4-(trifluoromethyl)phenyl]propan-2-ol (62.0 mg, 0.173 mmol, 70%) as a microcrystalline colourless solid.

TLC: R_f = 0.23 (7:3, pentane/diethylether) [UV]

mpt: 136 – 138 °C (diethylether)

^{1}H NMR: (500 MHz, $CDCl_3$)

8.88 (s, 1H), 8.64 (ddd, *J* = 5.0, 1.9, 0.9 Hz, 1H), 7.85 (td, *J* = 7.7, 1.8 Hz, 1H), 7.71 – 7.66 (m, 2H), 7.60 (d, *J* = 8.2 Hz, 2H), 7.52 (dt, *J* = 7.9, 1.1 Hz, 1H), 7.44 – 7.40 (m, 1H), 7.37 – 7.29 (m, 2H), 7.24 (td, *J* = 7.5, 1.5 Hz, 1H), 6.81 (d, *J* = 7.7 Hz, 1H), 3.16 (d, *J* = 13.7 Hz, 1H), 3.05 (d, *J* = 13.7 Hz, 1H), 1.69 (s, 3H).

^{13}C NMR: (126 MHz, $CDCl_3$)

158.7, 154.3, 146.9, 139.9, 138.0, 135.7, 132.3, 130.3, 128.3 (q, *J* = 32.0 Hz), 128.3, 126.8, 125.7, 125.2, 124.8 (q, *J* = 3.9 Hz), 124.5 (q, *J* = 269.8 Hz) 122.3, 73.4, 46.6, 30.9.

^{19}F NMR: (471 MHz, $CDCl_3$)

62.1.

MS: (HRMS - ESI$^+$)

Found 380.1228 ($C_{21}H_{18}ONF_3Na$), requires 380.1233.

2-[2-(4-Iodobenzyl)phenyl]pyridine 16

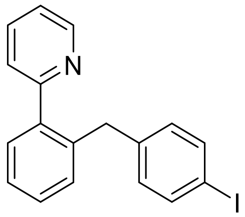

Trifluoromethanesulfonic acid trimethylsilylester (100 µL, 0.550 mmol, 2.2 equiv.) was added to a stirred solution of 2-{2-[(trimethylsilyl)methyl]phenyl}pyridine (60.4 mg, 0.250 mmol) and (diacetoxyiodo)benzene (80.5 mg, 0.250 mmol, 1.0 equiv.) in acetonitrile/dichloromethane (7:3, 1.7 mL, 0.15 M) at –78 °C. The reaction mixture was stirred for 3 hours at –78 °C, warmed to room temperature and aqueous sodium hydroxide (5 mL, 1 M) added. The organic phase was extracted using ethyl acetate (3 x 5 mL) and the product purified by flash column chromatography (SiO_2, 50 g, biotage isolera, hexane/ethyl acetate – 100/0 to 85/15, 100 mL/min) to give 2-[2-(4-iodobenzyl)phenyl]pyridine (27.0 mg, 73 µmol, 29%) as a colourless oil.

TLC: R_f = 0.14 (9:1, hexane/ethyl acetate) [UV]

^{1}H NMR: (500 MHz, $CDCl_3$)

8.67 (ddd, *J* = 4.8, 1.8, 1.0 Hz, 1H), 7.67 (td, *J* = 7.7, 1.8 Hz, 1H), 7.52 – 7.42 (m, 2H), 7.40 – 7.27 (m, 3H), 7.26 – 7.18 (m, 3H), 6.76 – 6.63 (m, 2H), 4.07 (s, 2H).

^{13}C NMR: (126 MHz, $CDCl_3$)

159.8, 149.1, 141.1, 140.6, 138.3, 137.2, 136.3, 131.0, 130.6, 130.0, 128.5, 126.6, 124.2, 121.8, 90.9, 38.4.

MS: (HRMS - ESI$^+$)

Found 372.0237 ($C_{18}H_{15}NI$), requires 372.0244.

2-{4'-Methoxy-3-[(trimethylsilyl)methyl]-(1,1'-biphenyl)-2-yl}pyridine 18

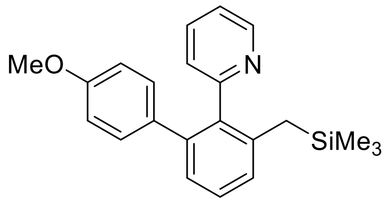

2-{2-[(Trimethylsilyl)methyl]phenyl}pyridine (60.4 mg, 0.250 mmol) was reacted with 4-bromoanisole (46.8 mg, 0.25 mmol, 1.0 equiv.) in the presence of potassium acetate (7.4 mg, 75 µmol, 30 mol%), potassium carbonate (69.0 mg, 0.500 mmol, 2.0 equiv.) and $[(^{t}BuCN)_5Ru(H_2O)](BF_4)_2$ (18.0 mg, 25 µmol, 10 mol%) in 1-methyl-2-pyrrolidinone (250 µL) at 35 °C for 24 hours. Brine (5 mL) was added, the organic products extracted with ethyl acetate (3 x 5 mL), dried (Na_2SO_4), the solvent removed *in vacuo* (500 to 50 mbar) and the product purified by flash column chromatography (SiO_2, 25 g, biotage isolera, hexane/ethyl acetate – 100/0 to 90/10, 80 mL/min) to give 2-{4'-methoxy-3-[(trimethylsilyl)methyl]-(1,1'-biphenyl)-2-yl}pyridine (81.0 mg, 0.233 mmol, 93%) as a colourless oil.

TLC: R_f = 0.50 (4:1, hexane/ethyl acetate) [UV]

^{1}H NMR: (500 MHz, $CDCl_3$)

8.63 (ddd, *J* = 4.9, 1.9, 1.0 Hz, 1H), 7.40 (td, *J* = 7.7, 1.9 Hz, 1H), 7.28 (t, *J* = 7.6 Hz, 1H), 7.12 (ddd, *J* = 11.2, 7.7, 1.3 Hz, 2H), 7.06 (ddd, *J* = 7.6, 4.9, 1.2 Hz, 1H), 6.99 – 6.91 (m, 2H), 6.82 (dt, *J* = 7.9, 1.1 Hz, 1H), 6.70 – 6.60 (m, 2H), 3.73 (s, 3H), -0.17 (s, 9H).

^{13}C NMR: (126 MHz, $CDCl_3$)

161.1, 159.1, 149.8, 142.3, 140.8, 138.9, 136.4, 135.9, 131.8, 129.5, 128.8, 128.2, 127.3, 122.2, 114.1, 56.3, 24.8.

MS: (HRMS - ESI$^+$)

Found 348.1781 ($C_{22}H_{26}NOSi$), requires 348.1778.

(*E*)-2-(2-Styrylphenyl)pyridine 20

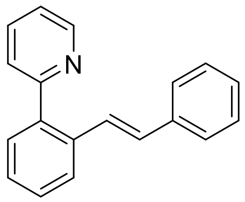

n-BuLi (1.6 M in hexanes, 160 μL, 0.260 mmol, 1.55 equiv.) was added dropwise (over approximately 30 seconds) to a stirred solution of 2-{2-[(trimethylsilyl)methyl]phenyl}pyridine (60.4 mg, 0.250 mmol) in anhydrous THF (50 μL) at –50 °C. Potassium *tert*-butoxide (1 M in THF, 260 μL, 0.26 mmol, 1.55 equiv.) was added dropwise (over approximately 30 seconds) and the reaction mixture stirred for 30 minutes before the addition of a solution of (*E*)-*N*,1-diphenylmethanimine (30.3 mg, 0.167 mmol) in anhydrous THF (0.5 mL). The reaction mixture was stirred for 1 hour at –50 °C, warmed to room temperature and quenched with saturated aqueous ammonium chloride (1 mL). The organic products were extracted using ethyl acetate (3 x 5 mL), dried (Na_2SO_4) and the product purified by flash column chromatography (SiO_2, 25 g, biotage isolera, hexane/ethyl acetate – 100/0 to 90/10, 80 mL/min) to give (*E*)-2-(2-styrylphenyl)pyridine (32.0 mg, 0.125 mmol, 75%) as a colourless oil.

TLC: R_f = 0.47 (4:1, hexane/ethyl acetate) [UV]

^{1}H NMR: (500 MHz, $CDCl_3$)

8.76 (d, *J* = 4.9 Hz, 1H), 7.81 – 7.69 (m, 2H), 7.56 (dd, *J* = 7.6, 1.6 Hz, 1H), 7.48 – 7.36 (m, 5H), 7.33 – 7.27 (m, 3H), 7.26 – 7.20 (m, 2H), 7.06 (d, *J* = 16.2 Hz, 1H).

^{13}C NMR: (126 MHz, $CDCl_3$)

158.9, 149.6, 139.6, 137.6, 136.0, 135.7, 130.2, 130.1, 128.7, 128.6, 127.7, 127.6, 127.6, 126.6, 126.3, 125.1, 121.9.

MS: (HRMS - ESI$^+$)

Found 258.1280 ($C_{19}H_{16}N$), requires 258.1277.

2-(*o*-Tolyl)pyridine 21

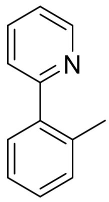

Following general procedure B, 2-phenylpyridine (39.0 mg, 0.250 mmol) was reacted with trimethylsilylbromomethane (60.4 mg, 0.250 mmol, 1.0 equiv.) in the presence of $[(^t BuCN)_5Ru(H_2O)](BF_4)_2$ (9.0 mg, 13 µmol, 5 mol%), potassium carbonate (69.0 mg, 0.500 mmol, 2 equiv.), potassium phenylphosphonate (17.6 mg, 75 µmol, 30 mol%), sodium iodide (37.5 mg, 0.250 mmol, 1.0 equiv.) and anhydrous tetrahydrofuran (500 µL) at 80 °C for 18 hours. Tetrabutylammonium fluoride solution (1M in THF, 500 µL, 0.500 mmol, 2.0 equiv.) was added to the reaction mixture at room temperature and stirred for 1.5 hours. The solvent was removed *in vacuo* (500 to 50 mbar, 40 °C) and the crude reaction mixture was purified by flash column chromatography (SiO_2, 25 g, biotage isolera, hexane/ethyl acetate – 100/0 to 94/6, 80 mL/min) to give 2-(*o*-tolyl)pyridine (38.0 mg, 0.225 mmol, 90%) as a yellow oil.

TLC: R_f = 0.32 (6:1, hexane/ethyl acetate) [UV]

^{1}H NMR: (500 MHz, $CDCl_3$)

8.70 (ddd, *J* = 4.9, 1.9, 0.9 Hz, 1H), 7.74 (td, *J* = 7.7, 1.9 Hz, 1H), 7.42 – 7.37 (m, 2H), 7.34 – 7.19 (m, 4H), 2.37 (s, 3H).

^{13}C NMR: (126 MHz, $CDCl_3$)

160.1, 149.3, 140.5, 136.1, 135.8, 130.7, 129.6, 128.3, 125.9, 124.1, 121.6, 20.3.

Data were in accordance with those previously reported[7].

Product characterisation – *meta*-silylmethylation product further reactivity

2-[3-(Pyridin-2-yl)benzyl]pyridine 23

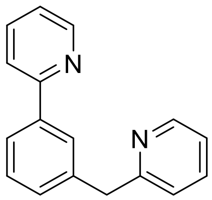

Tetrabutylammonium fluoride solution (1 M in THF, 50 µL, 50 µmol, 20 mol%) was added to a stirred mixture of 2-{3-[bis(trimethylsilyl)methyl]phenyl}pyridine (78.4 mg, 0.250 mmol) and pyridine *N*-oxide (23.8 mg, 0.250 mmol, 1.0 equiv.) in anhydrous dimethylformamide (600 µL) at 25 °C. The reaction mixture was stirred for 24 hours before the addition of brine (5 mL). The organic products were extracted using dichloromethane (3 x 5 mL), washed with brine (5 mL), dried (Na_2SO_4), the solvent removed *in vacuo* (600 to 300 mbar, 40 °C) and the product purified by flash column chromatography (SiO_2, 25 g, biotage isolera, dichloromethane/methanol – 100/0 to 92/8, 80 mL/min) to give 2-[3-(pyridin-2-yl)benzyl]pyridine (37.0 mg, 0.150 mmol, 60%) as a yellow oil.

TLC: R_f = 0.64 (95:5, dichloromethane/methanol) [UV]

^{1}H NMR: (500 MHz, $CDCl_3$)

8.68 (ddd, *J* = 4.8, 1.8, 1.0 Hz, 1H), 8.56 (ddd, *J* = 4.9, 1.9, 1.0 Hz, 1H), 7.95 – 7.91 (m, 1H), 7.84 (dt, *J* = 7.8, 1.5 Hz, 1H), 7.76 – 7.69 (m, 2H), 7.57 (td, *J* = 7.7, 1.9 Hz, 1H), 7.42 (t, *J* = 7.7 Hz, 1H), 7.35 – 7.30 (m, 1H), 7.22 (ddd, *J* = 6.7, 4.8, 1.7 Hz, 1H), 7.16 – 7.13 (m, 1H), 7.11 (ddd, *J* = 7.5, 4.9, 1.2 Hz, 1H), 4.26 (s, 2H).

^{13}C NMR: (126 MHz, $CDCl_3$)

160.9, 157.5, 149.7, 149.4, 140.0, 139.8, 136.7, 136.6, 129.8, 129.0, 127.8, 125.1, 123.2, 122.1, 121.3, 120.7, 44.8.

MS: (HRMS - ESI$^+$)

Found 269.1047 ($C_{17}H_{14}N_2Na$), requires 269.1049.

2-[3-(Pyridin-4-ylmethyl)phenyl]pyridine 24

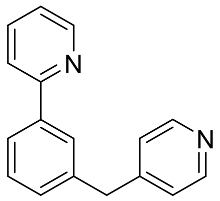

Caesium fluoride (38.0 mg, 0.250 mmol, 1.0 equiv.) was added to a stirred mixture of 2-{3-[bis(trimethylsilyl)methyl]phenyl}pyridine (78.4 mg, 0.250 mmol), 4-cyanopyridine (26.0 mg, 0.250 mmol, 1.0 equiv.) and 18-crown-6 (72.7 mg, 0.275 mmol, 1.1 equiv.) in dimethylsulfoxide (750 µL). The reaction mixture was stirred at 25 °C for 18 hours before addition of brine (15 mL). The organic phase was extracted with ethyl acetate (3 x 10 mL), dried (Na_2SO_4), the solvent removed *in vacuo* (500 to 25 mbar, 40 °C), and the product purified by flash column chromatography (SiO_2, 10 g, biotage isolera, hexane/ethyl acetate – 90/10 to 1/99, 40 mL/min) to give 2-[3-(pyridin-4-ylmethyl)phenyl]pyridine (44.0 mg, 0.179 mmol, 72%) as a yellow oil. ***Safety note**: the aqueous phase during work-up was monitored by pH indicator paper to ensure that this phase never became acidic.*

TLC: R_f = 0.24 (ethyl acetate) [UV]

^{1}H NMR: (500 MHz, $CDCl_3$)

8.69 (ddd, *J* = 4.9, 1.8, 1.0 Hz, 1H), 8.53 – 8.46 (m, 2H), 7.88 – 7.86 (m, 1H), 7.84 (dt, *J* = 7.9, 1.4 Hz, 1H), 7.75 (td, *J* = 7.6, 1.8 Hz, 1H), 7.70 (dt, *J* = 7.9, 1.2 Hz, 1H), 7.42 (t, *J* = 7.7 Hz, 1H), 7.25 – 7.20 (m, 2H), 7.17 – 7.11 (m, 2H), 4.06 (s, 2H).

^{13}C NMR: (126 MHz, $CDCl_3$)

157.2, 149.9, 149.9, 149.7, 139.9, 139.5, 136.8, 129.7, 129.2, 127.7, 125.3, 124.2, 122.3, 120.7, 41.3.

MS: (HRMS - ESI$^+$)

Found 247.1230 ($C_{17}H_{15}N_2$), requires 247.1230.

_N,N_-Dimethyl-4-[3-(pyridin-2-yl)phenyl]-4-(pyridin-4-yl)butanamide 26

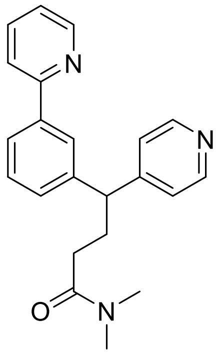

Caesium fluoride (45.6 mg, 0.300 mmol, 1.2 equiv.) was added to a stirred mixture of 2-{3-[bis(trimethylsilyl)methyl]phenyl}pyridine (78.4 mg, 0.250 mmol), 4-cyanopyridine (26.0 mg, 0.250 mmol, 1.0 equiv.), *N*,*N*-dimethylacrylamide (49.6 mg, 0.500 mmol, 2.0 equiv.) and 18-crown-6 (79.2 mg, 0.30 mmol, 1.2 equiv.) in dimethylsulfoxide (1.0 mL). The reaction mixture was stirred at 25 °C for 18 hours before addition of brine (5 mL). The organic phase was extracted with ethyl acetate (3 x 10 mL), dried (Na_2SO_4), the solvent removed *in vacuo* (500 to 25 mbar, 40 °C), and the product purified by flash column chromatography (SiO_2, 10 g, biotage isolera, dichloromethane/methanol – 100/0 to 92/8, 40 mL/min) to give *N,N*-dimethyl-4-[3-(pyridin-2-yl)phenyl]-4-(pyridin-4-yl)butanamide (40.0 mg, 0.116 mmol, 46%) as a yellow oil. ***Safety note**: the aqueous phase during work-up was monitored by pH indicator paper to ensure that this phase never became acidic.*

TLC: R_f = 0.23 (95:5, dichloromethane/methanol) [UV]

^{1}H NMR: (500 MHz, $CDCl_3$)

8.70 – 8.63 (m, 1H), 8.51 – 8.42 (m, 2H), 7.88 (app. s., 1H), 7.85 – 7.80 (m, 1H), 7.75 – 7.66 (m, 2H), 7.41 (td, *J* = 7.7, 2.3 Hz, 1H), 7.29 – 7.26 (m, 1H), 7.24 – 7.17 (m, 3H), 4.10 (t, *J* = 7.3 Hz, 1H), 2.94 – 2.87 (m, 3H), 2.85 – 2.78 (m, 3H), 2.52 – 2.38 (m, 2H), 2.36 – 2.16 (m, 2H).

^{13}C NMR: (126 MHz, $CDCl_3$)

172.0, 157.1, 153.4, 150.0, 149.7, 143.0, 139.9, 136.8, 129.2, 128.4, 126.8, 125.5, 123.2, 122.3, 120.7, 49.9, 37.1, 35.4, 31.0, 30.0.

MS: (HRMS - ESI$^+$)

Found 368.1730 ($C_{22}H_{23}ON_3Na$), requires 368.1733.

3-(Pyridin-2-yl)benzaldehyde 27

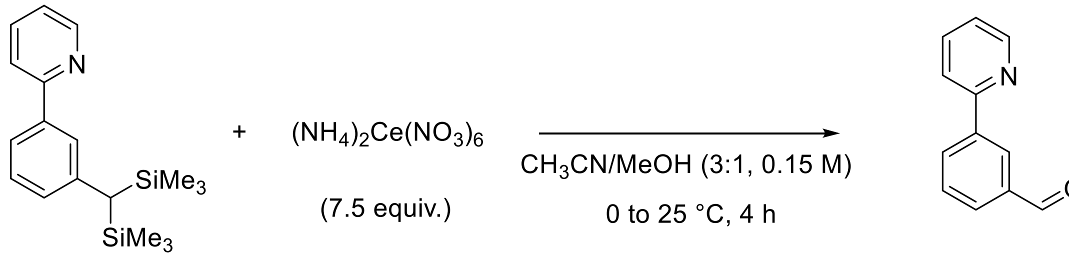

Ceric ammonium nitrate (2.06 g, 3.75 mmol, 7.50 equiv.) was added portion-wise (x 2) to a stirred solution of 2-{3-[bis(trimethylsilyl)methyl]phenyl}pyridine (157.0 mg, 0.500 mmol) in acetonitrile (2.5 mL) and methanol (800 µL) at 0 °C. The reaction mixture was warmed to 25 °C and stirred for 4 hours before the addition of saturated ammonium chloride (30 mL). The organics were extracted with dichloromethane (3 x 30 mL) and the solvent removed *in vacuo* (500 to 300 mbar, 40 °C). Methanol (30 mL) and sodium metabisulfite (1.05 g, 5.50 mmol) were added to the crude reaction mixture before the addition of water (30 mL). The mixture was stirred vigorously for 10 minutes and the organics extracted with ethyl acetate (2 x 20 mL). Aqueous sodium hydroxide (2 M) was then added dropwise until pH > 12. The organics were extracted with ethyl acetate (3 x 20 mL), dried ($MgSO_4$) and the solvent removed *in vacuo* (500 to 10 mbar, 40 °C) to give 3-(pyridine-2-yl)benzaldehyde (70.0 mg, 0.382 mmol, 60%) as a colourless oil.

TLC: R_f = 0.13 (4:1 hexane/diethylether) [UV]

IR: (*neat*, cm^{-1})

2923.5 (w), 1696.6 (s), 1600.1 (m), 1183.4 (m), 770.9 (s).

^{1}H NMR: (500 MHz, $CDCl_3$)

10.15 (s, 1H), 8.77 (dt, *J* = 4.9, 1.4 Hz, 1H), 8.54 (t, *J* = 1.8 Hz, 1H), 8.34 (dt, *J* = 7.8, 1.6 Hz, 1H), 7.98 (dt, *J* = 7.6, 1.4 Hz, 1H), 7.90 – 7.81 (m, 2H), 7.69 (t, *J* = 7.7 Hz, 1H), 7.38 – 7.31 (m, 1H).

^{13}C NMR: (126 MHz, $CDCl_3$)

192.2, 155.8, 149.7, 140.0, 137.4, 136.9, 132.8, 129.9, 129.6, 128.5, 122.9, 120.8.

Data were in accordance with those previously reported[8].

2-{4-[Bis(trimethylsilyl)methyl]-4'-methoxy-(1,1'-biphenyl)-2-yl}pyridine 28

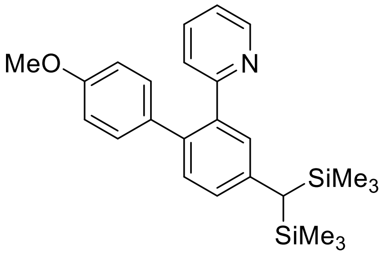

Following general procedure A, 2-phenylpyridine (38.8 mg, 0.250 mmol) was reacted with bis(trimethylsilyl)chloromethane (58.4 mg, 0.300 mmol, 1.2 equiv.) in the presence of $[(^{t}BuCN)_5Ru(H_2O)](BF_4)_2$ (9.0 mg, 13 μmol, 5 mol%), lithium carbonate (22.0 mg, 0.300 mmol, 1.2 equiv.), lithium cyclohexane carboxylate (3.4 mg, 25 μmol, 10 mol%), water (100 μL) and isopropanol (150 μL) at 100 °C for 4 hours. The reaction mixture was cooled to room temperature and 4-bromoanisole (46.8 mg, 0.250 mmol, 1.0 equiv.) added. The reaction was heated at 100 °C for 4 hours, cooled to room temperature, the solvent removed *in vacuo* (500 to 10 mbar, 40 °C) and the product purified by flash column chromatography (SiO_2, 10 g, biotage isolera, hexane/ethyl acetate – 100/0 to 92/8, 40 mL/min) to give 2-{4-[bis(trimethylsilyl)methyl]-4'-methoxy-(1,1'-biphenyl)-2-yl}pyridine (70.0 mg, 0.167 mmol, 67%) as a colourless amorphous solid.

TLC: R_f = 0.28 (9:1, hexane/ethyl acetate) [UV]

mpt: 124 – 126 °C (hexane)

^{1}H NMR: (400 MHz, C_6D_6)

8.63 (ddd, *J* = 4.8, 1.9, 1.0 Hz, 1H), 7.79 (d, *J* = 2.1 Hz, 1H), 7.37 (d, *J* = 7.9 Hz, 1H), 7.14 – 7.11 (m, 2H), 7.07 (dd, *J* = 7.9, 2.1 Hz, 1H), 6.97 – 6.93 (m, 1H), 6.80 (td, *J* = 7.7, 1.9 Hz, 1H), 6.67 – 6.58 (m, 2H), 6.54 (ddd, *J* = 7.4, 4.8, 1.2 Hz, 1H), 3.20 (s, 3H), 1.57 (s, 1H), 0.13 (s, 18H).

^{13}C NMR: (126 MHz, $CDCl_3$)

159.8, 157.8, 149.0, 141.8, 138.7, 135.0, 134.9, 133.7, 130.7, 130.4, 129.8, 128.3, 125.1, 120.7, 113.1, 54.9, 28.8, 0.0.

MS: (HRMS - ESI$^+$)

Found 442.2010 ($C_{25}H_{33}NONaSi_2$), requires 442.1993.

(*E*)-2-(3-Styrylphenyl)pyridine 29

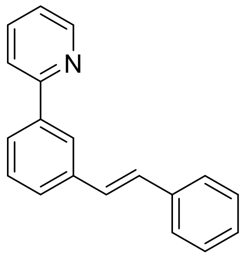

A solution of 2-{3-[bis(trimethylsilyl)methyl]phenyl}pyridine (78.4 mg, 0.250 mmol, 2.0 equiv.) and (*E*)-*N*,1-diphenylmethanimine (22.6 mg, 0.125 mmol) in anhydrous tetrahydrofuran (450 µL) was added to a mixture of tetrabutylammonium chloride (52.2 mg, 0.188 mmol, 1.50 equiv.) and potassium trimethylsilanolate (2M in THF, 94 µL, 0.188 mmol, 1.50 equiv.) in anhydrous tetrahydrofuran (400 µL) at –20 °C. The reaction mixture was stirred at –20 °C for 3 hours, quenched with sat. aqueous ammonium chloride solution (3 mL), warmed to room temperature and the organic products extracted using ethyl acetate (3 x 5 mL). The organic phase was dried (Na_2SO_4), the solvent removed *in vacuo* (500 to 25 mbar, 40 °C) and the product purified by flash column chromatography (SiO_2, 25 g, biotage isolera, hexane/ethyl acetate – 100/0 to 90/10, 80 mL/min) to give (*E*)-2-(3-styrylphenyl)pyridine (25.0 mg, 97.2 µmol, 78%, >99:1 E/Z) as a pale yellow oil.

TLC: R_f = 0.45 (4:1, hexane/ethyl acetate) [UV]

^{1}H NMR: (500 MHz, $CDCl_3$)

8.73 (dt, *J* = 4.8, 1.4 Hz, 1H), 8.20 – 8.16 (m, 1H), 7.86 (dt, *J* = 7.7, 1.5 Hz, 1H), 7.82 – 7.75 (m, 2H), 7.61 – 7.52 (m, 3H), 7.47 (t, *J* = 7.7 Hz, 1H), 7.38 (t, *J* = 7.6 Hz, 2H), 7.30 – 7.26 (m, 2H), 7.22 (dd, *J* = 16.7, 3.2 Hz, 2H).

^{13}C NMR: (126 MHz, $CDCl_3$)

157.3, 149.7, 139.8, 137.9, 137.3, 136.8, 129.2, 129.1, 128.7, 128.5, 127.7, 127.1, 126.6, 126.1, 125.2, 122.3, 120.7.

Data were in accordance with those previously reported[8].

2-(*m*-Tolyl)pyridine 30

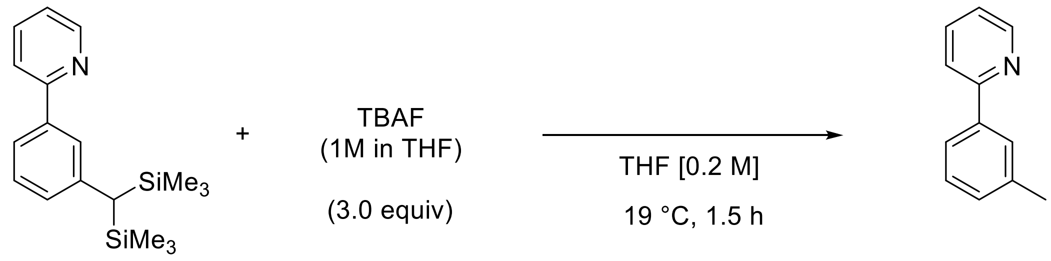

Tetra-n-butylammonium fluoride (1.50 mL, 1.50 mmol, 1 M in THF) was added dropwise to a stirred solution of 2-{3-[bis(trimethylsilyl)methyl]phenyl}pyridine (156.7 mg, 0.500 mmol) in tetrahydrofuran at 19 °C. The reaction mixture was stirred for 1.5 hours before the addition of dichloromethane (10 mL) and saturated aqueous potassium carbonate (15 mL). The organics were extracted with dichloromethane (10 mL x 3), dried ($MgSO_4$), the solvent removed *in vacuo* (600 mbar to 100 mbar, 40 °C) and the crude product purified by flash column chromatography (SiO_2, 10 g, biotage isolera, hexane/ethyl acetate – 100/0 to 80/20, 25 mL/min) to give 2-(*m*-tolyl)pyridine (76 mg, 0.45 mmol, 90%) as a colourless oil.

TLC: R_f = 0.35 (4:1 hexane/ethyl acetate) [UV]

^{1}H NMR: (500 MHz, $CDCl_3$)

8.70 (dt, *J* = 4.8, 1.4 Hz, 1H), 7.86 – 7.83 (m, 1H), 7.78 – 7.69 (m, 3H), 7.37 (t, *J* = 7.6 Hz, 1H), 7.26 – 7.19 (m, 2H), 2.45 (s, 3H).

^{13}C NMR: (126 MHz, $CDCl_3$)

157.6, 149.6, 139.3, 138.5, 136.8, 129.8, 128.7, 127.7, 124.0, 122.0, 120.7, 21.5.

Data were in accordance with those previously reported[9].

NMR Spectra

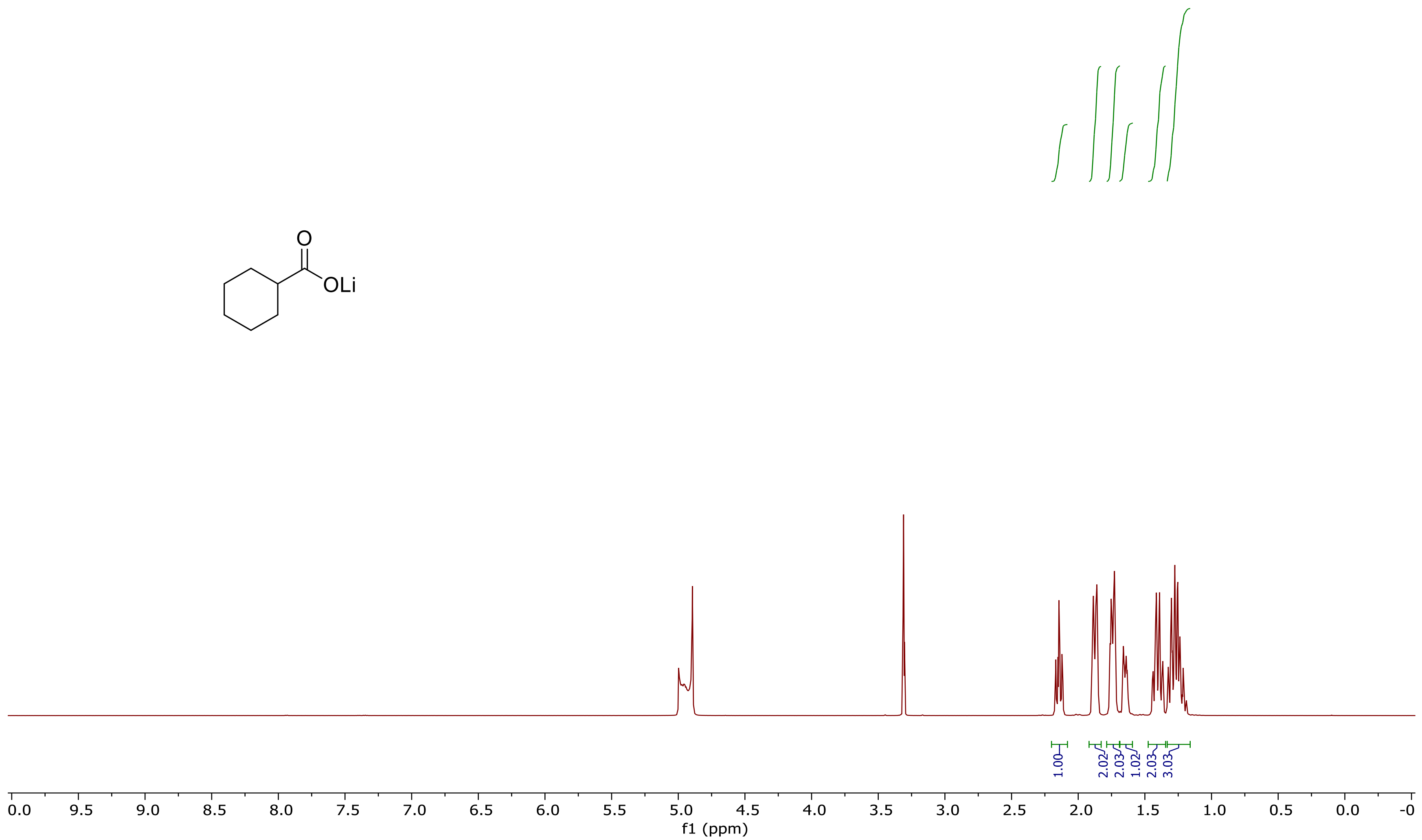

Supplementary Figure 48. ^{1}H NMR (500 MHz, d_4-MeOD) of lithium cyclohexane carboxylate.

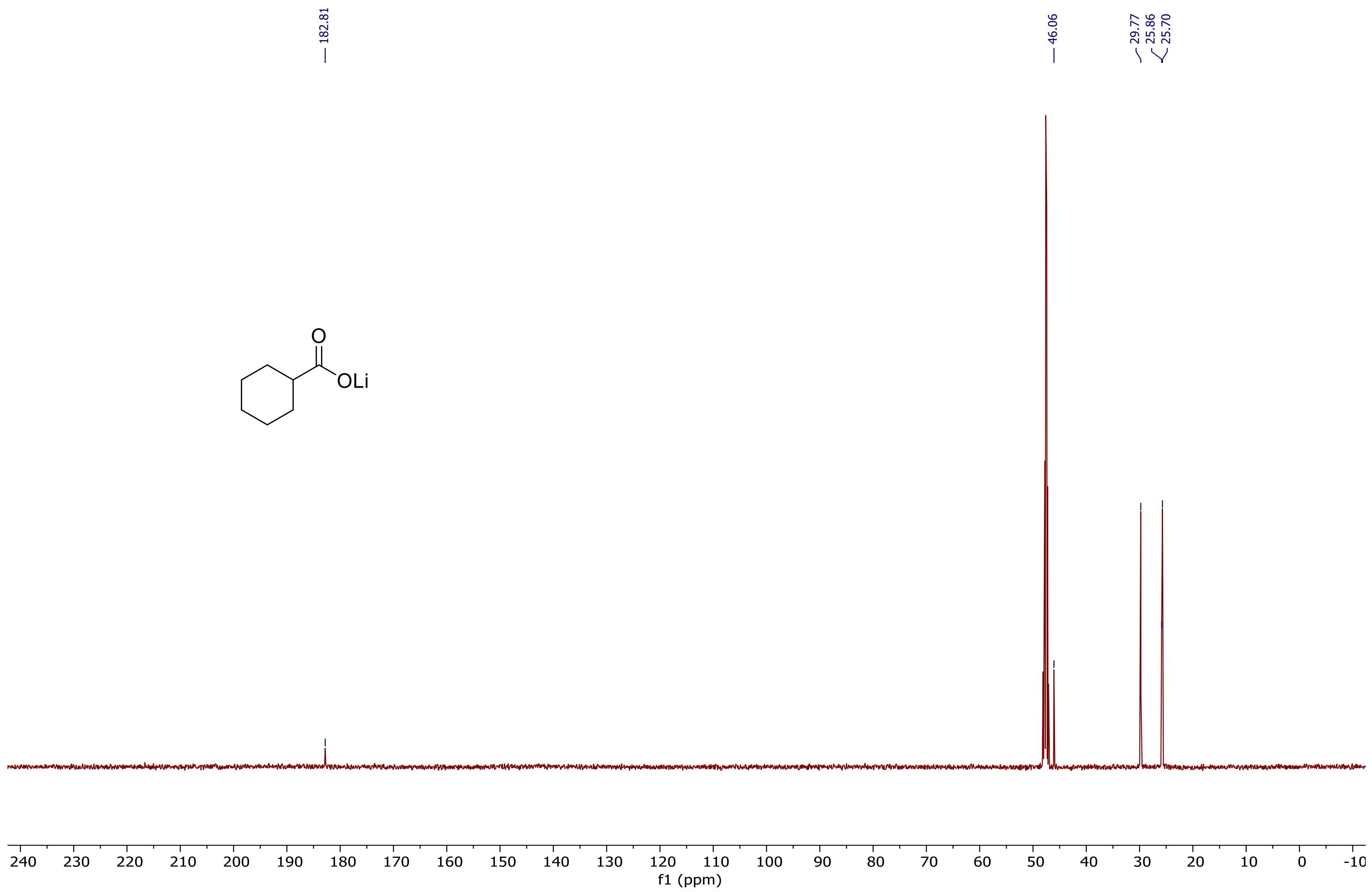

Supplementary Figure 49. ^{13}C NMR (126 MHz, d_4-MeOD) of lithium cyclohexane carboxylate.

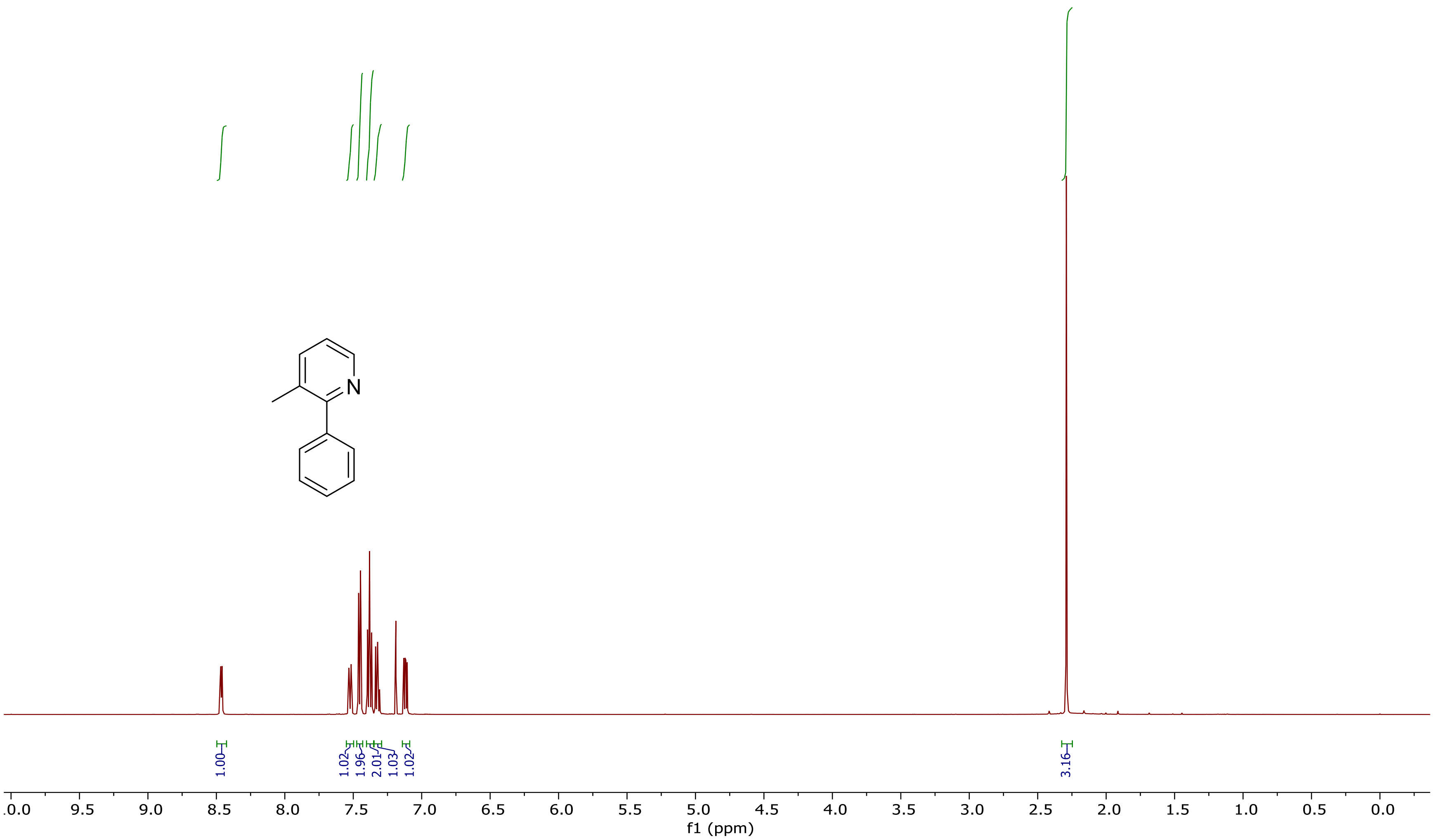

Supplementary Figure 50. ^{1}H NMR (500 MHz, $CDCl_3$) of 2-phenyl-3-methylpyridine.

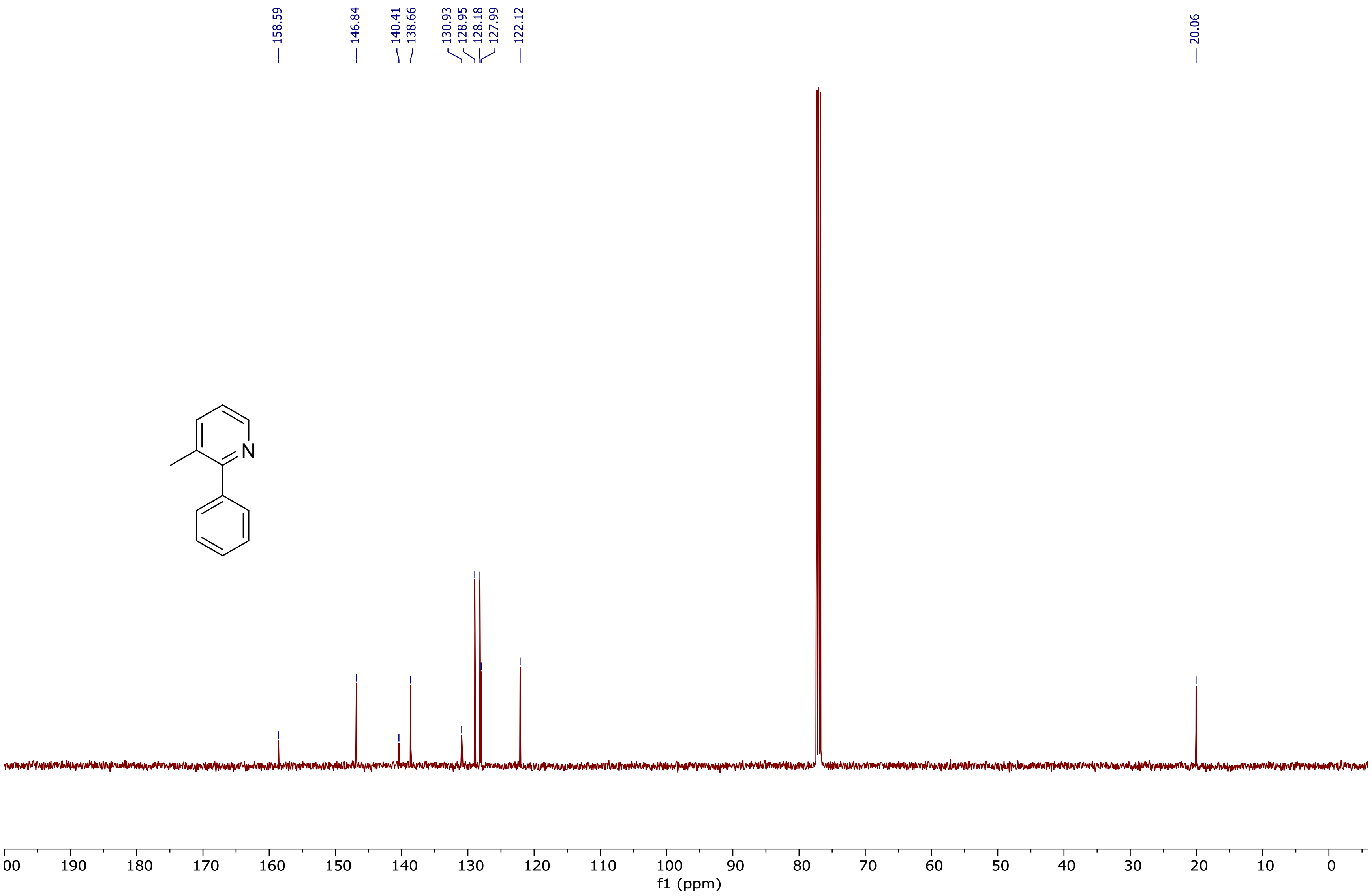

Supplementary Figure 51. ^{13}C NMR (126 MHz, $CDCl_3$) of 2-phenyl-3-methylpyridine.

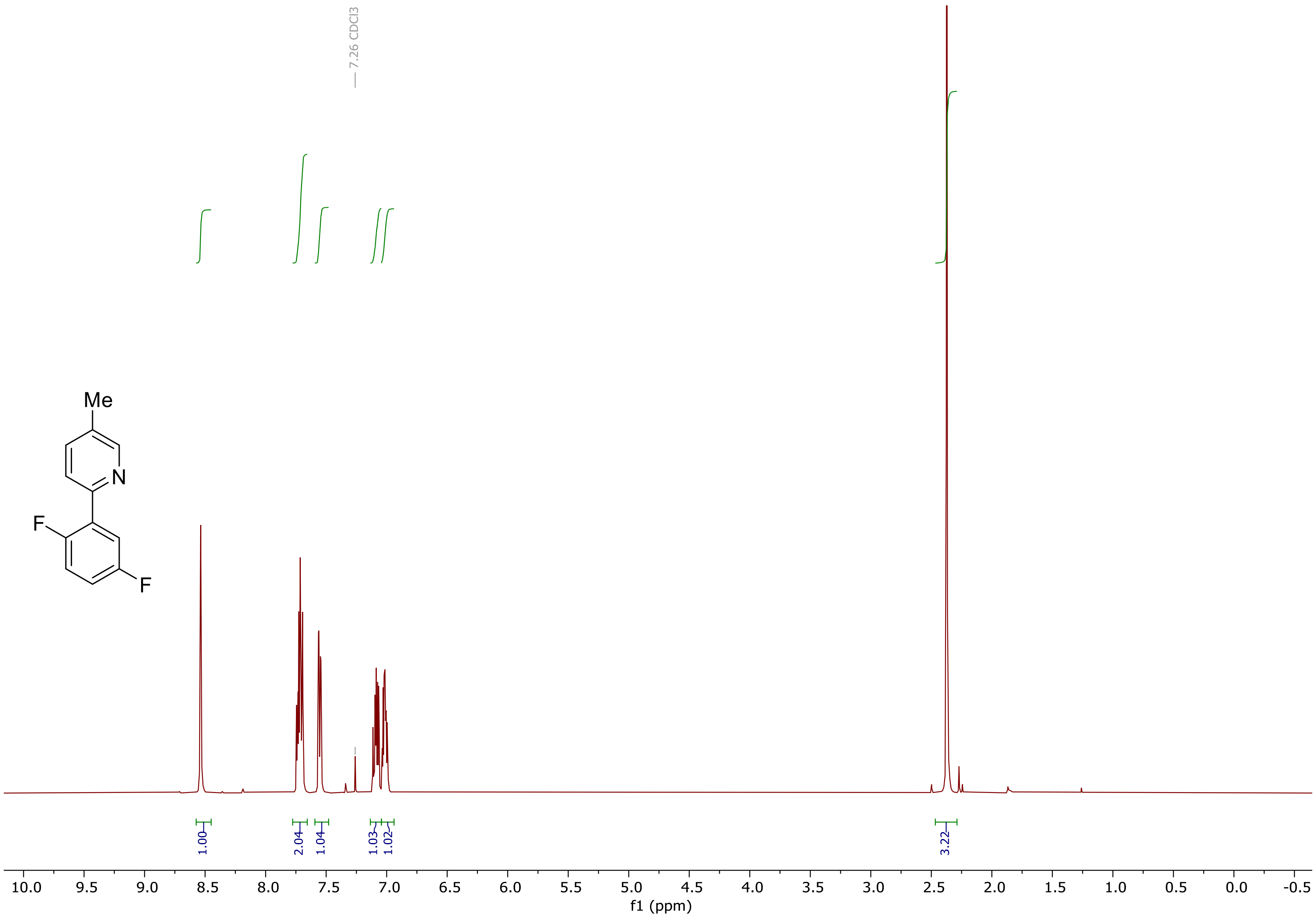

Supplementary Figure 52. ^1H NMR (500 MHz, $CDCl_3$) of 2-(2,5-difluorophenyl)-5-methylpyridine.

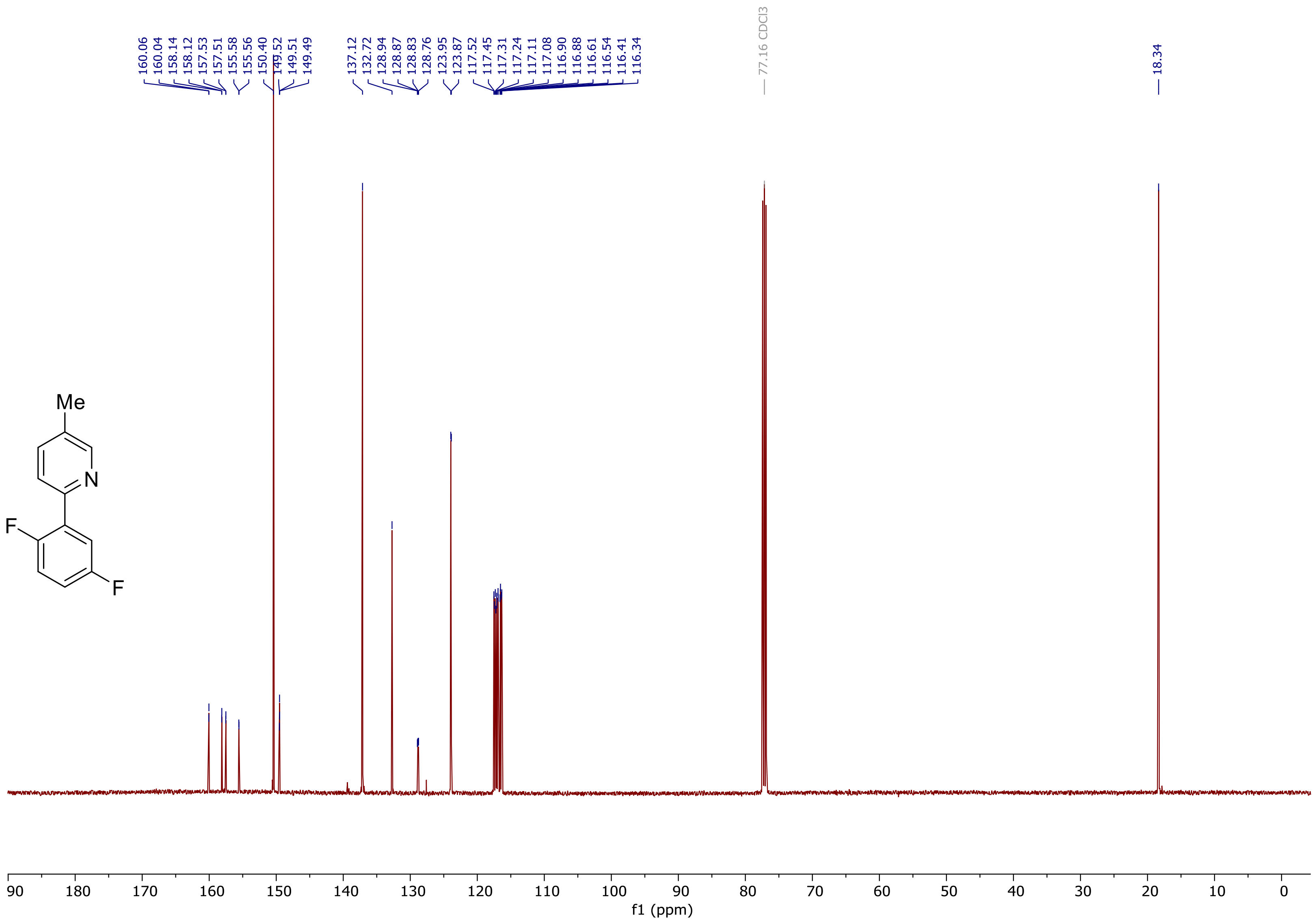

Supplementary Figure 53. ^{13}C NMR (126 MHz, $CDCl_3$) of 2-(2,5-difluorophenyl)-5-methylpyridine.

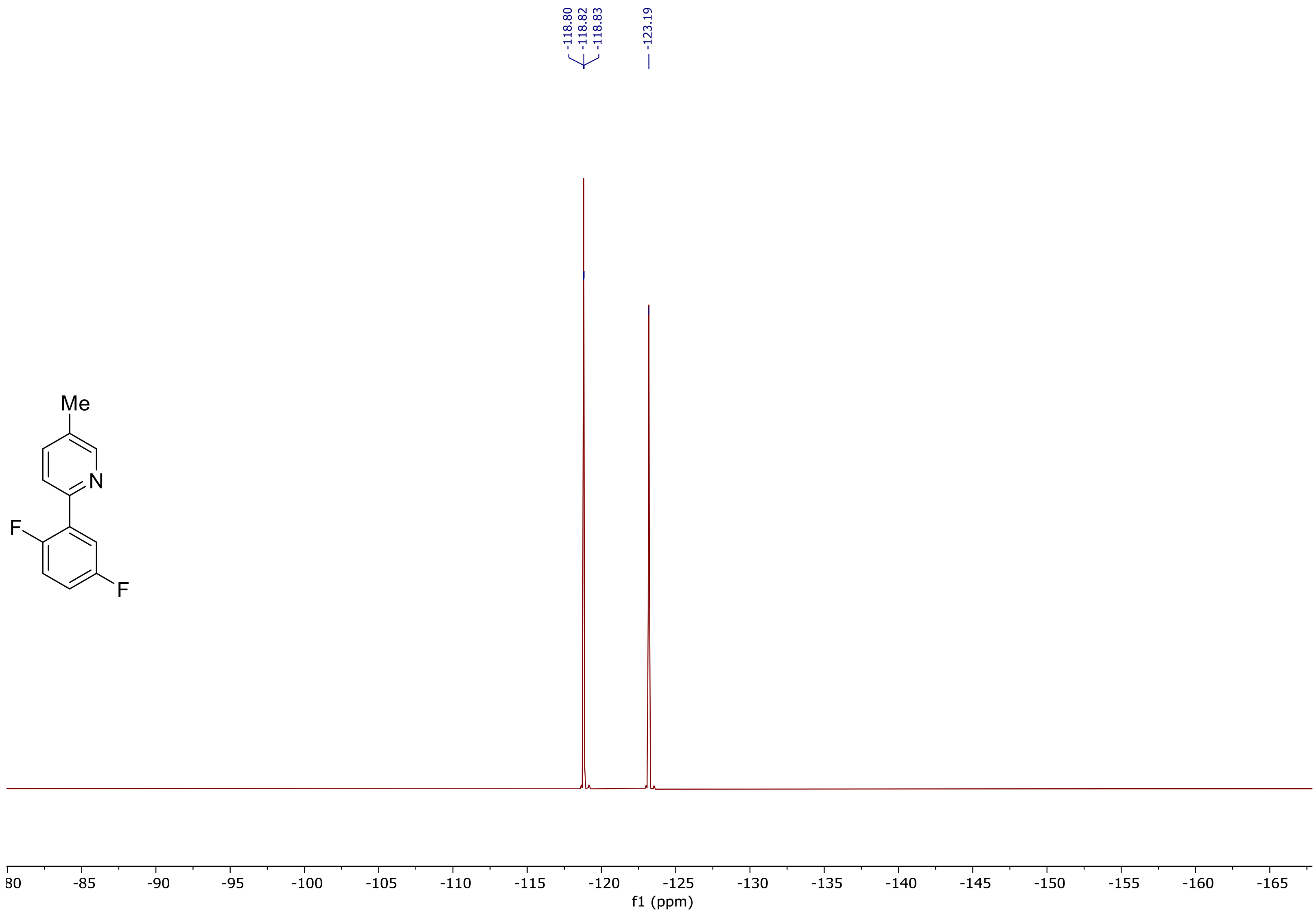

Supplementary Figure 54. ^{19}F NMR (471 MHz, $CDCl_3$) of 2-(2,5-difluorophenyl)-5-methylpyridine.

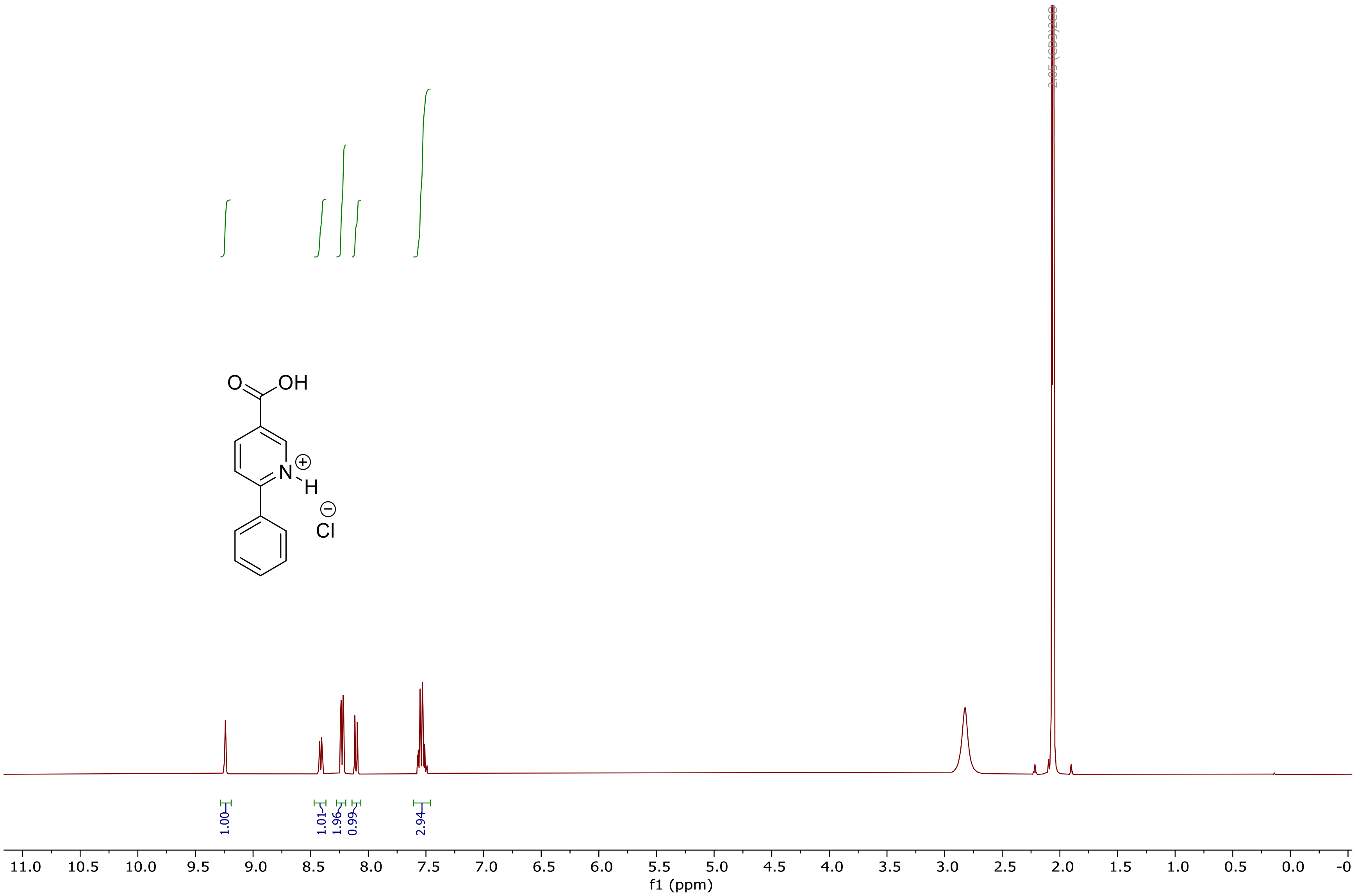

Supplementary Figure 55. ^{1}H NMR (400 MHz, d_6-acetone) of 6-phenylpyridine-3-carboxylic acid hydrochloride.

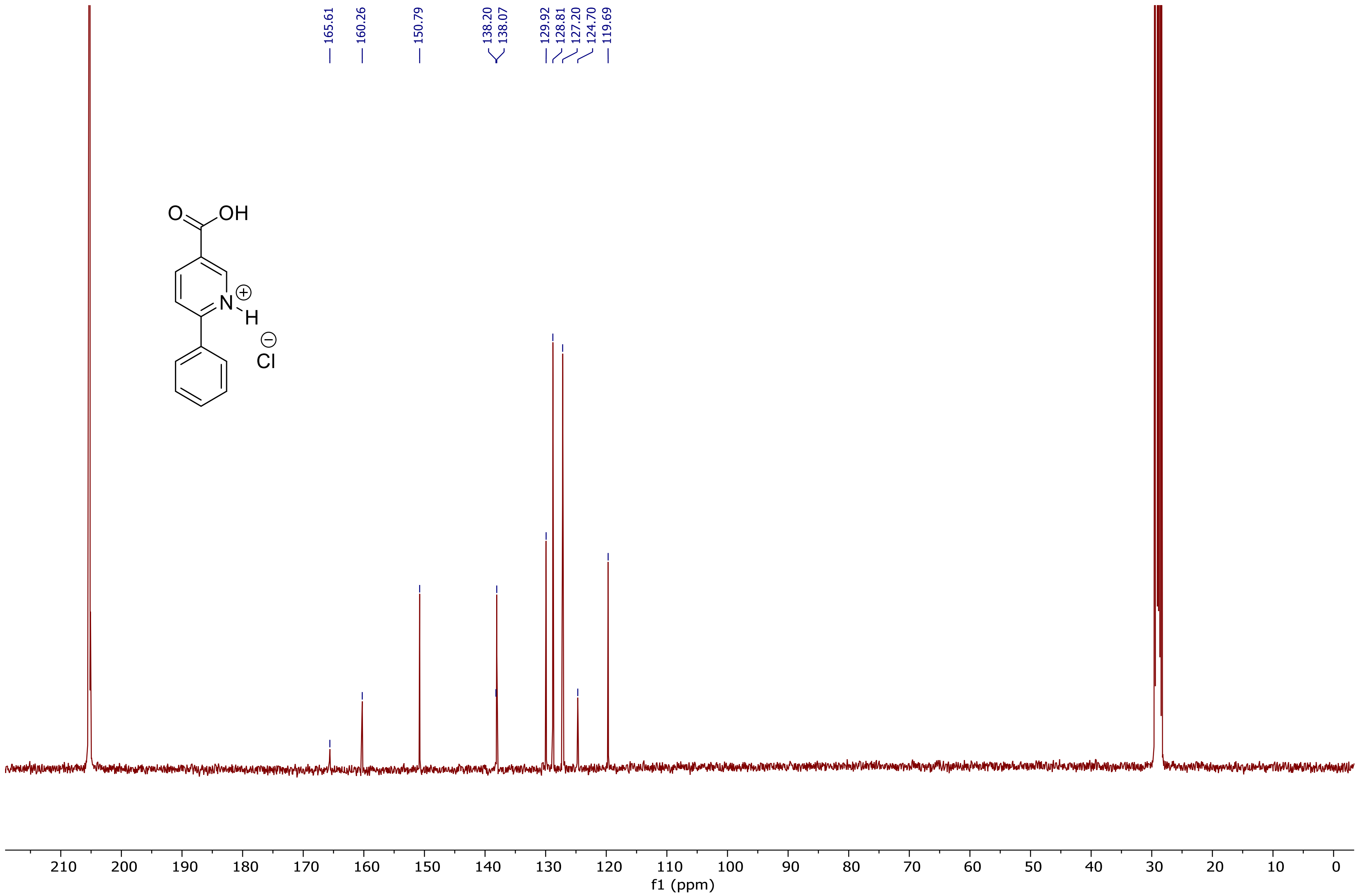

Supplementary Figure 56. ^{13}C NMR (101 MHz, d_6-acetone) of 6-phenylpyridine-3-carboxylic acid hydrochloride.

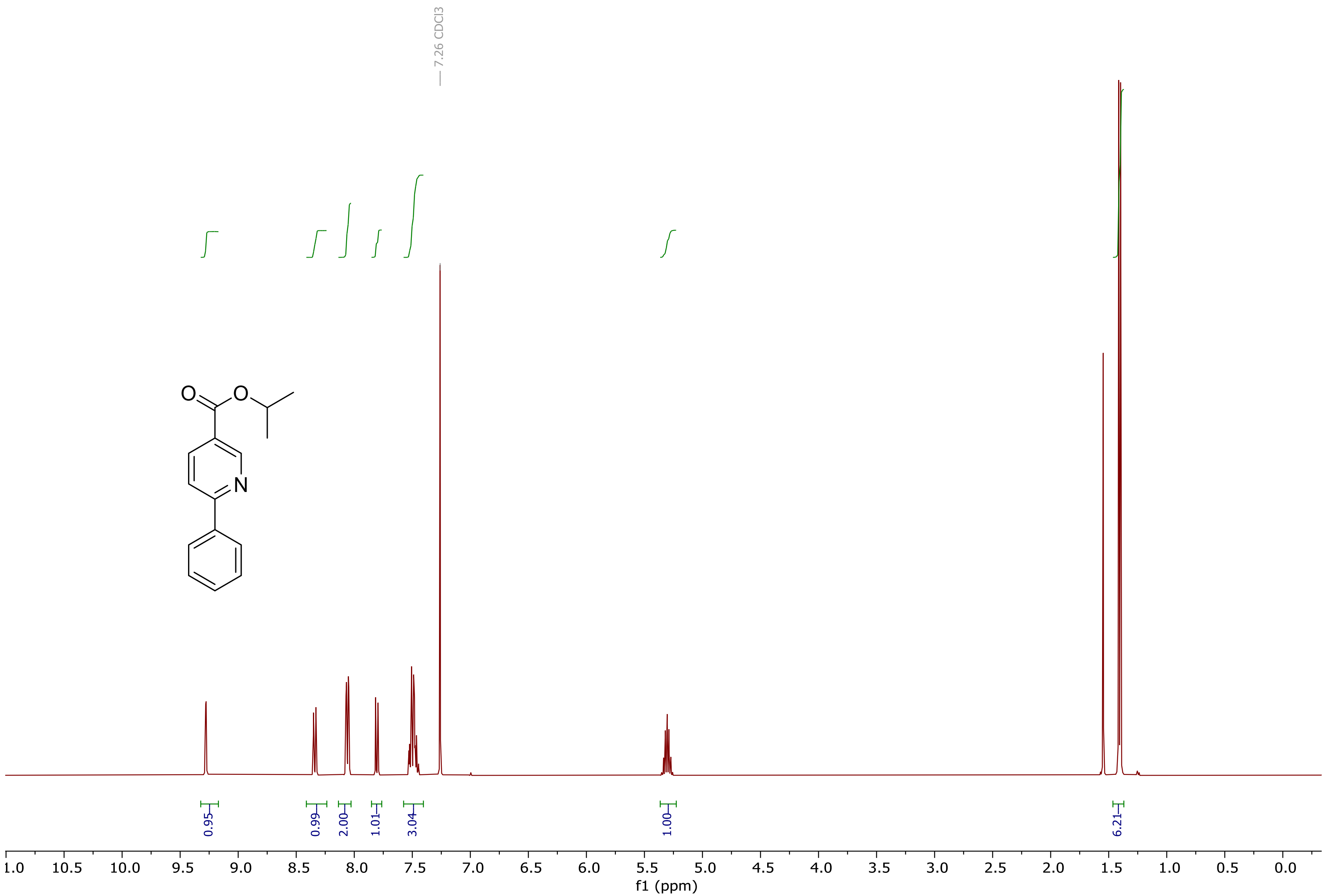

Supplementary Figure 57. ^{1}H NMR (400 MHz, $CDCl_3$) of isopropyl 6-phenylnicotinate.

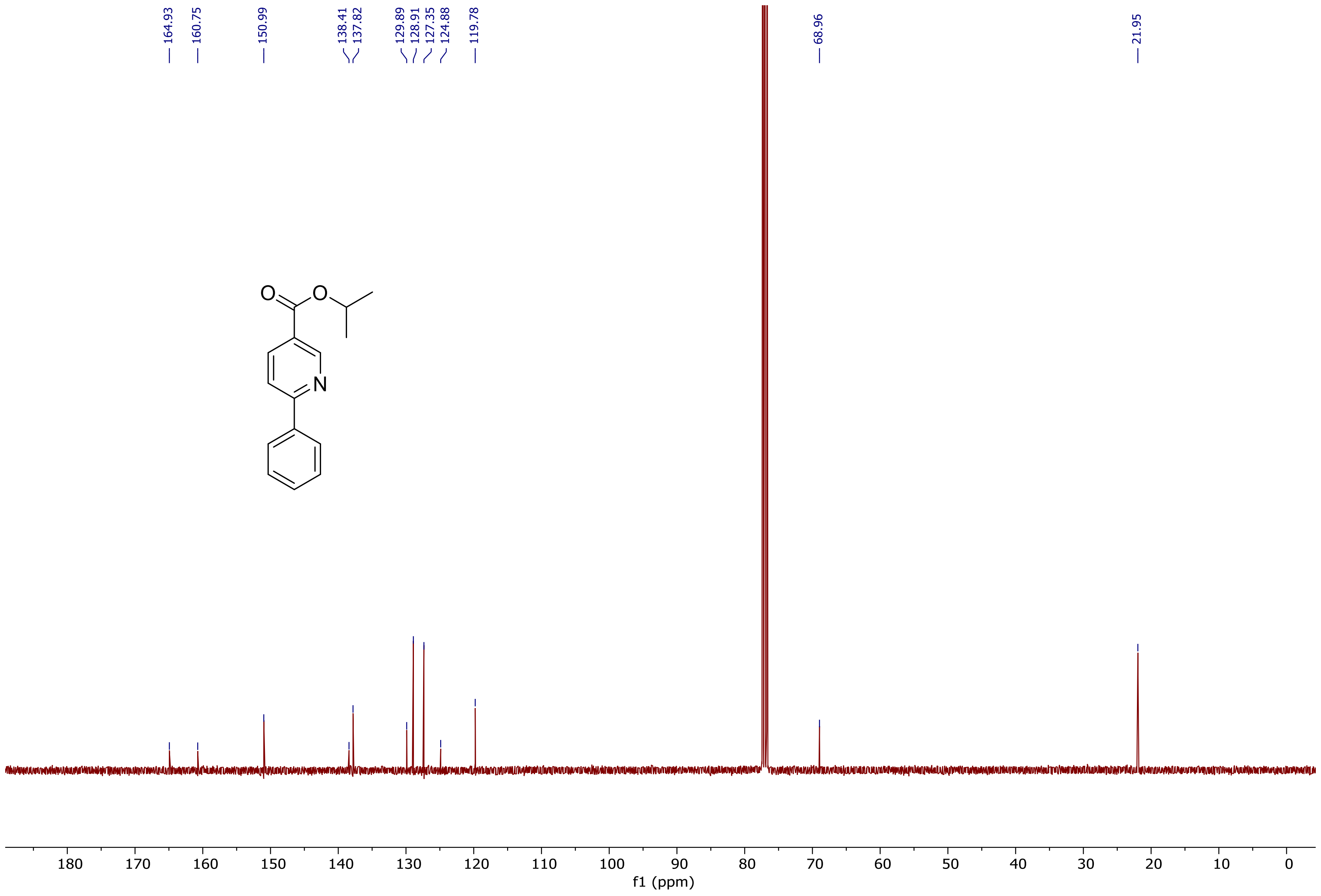

Supplementary Figure 58. ^{13}C NMR (101 MHz, $CDCl_3$) of isopropyl 6-phenylnicotinate.

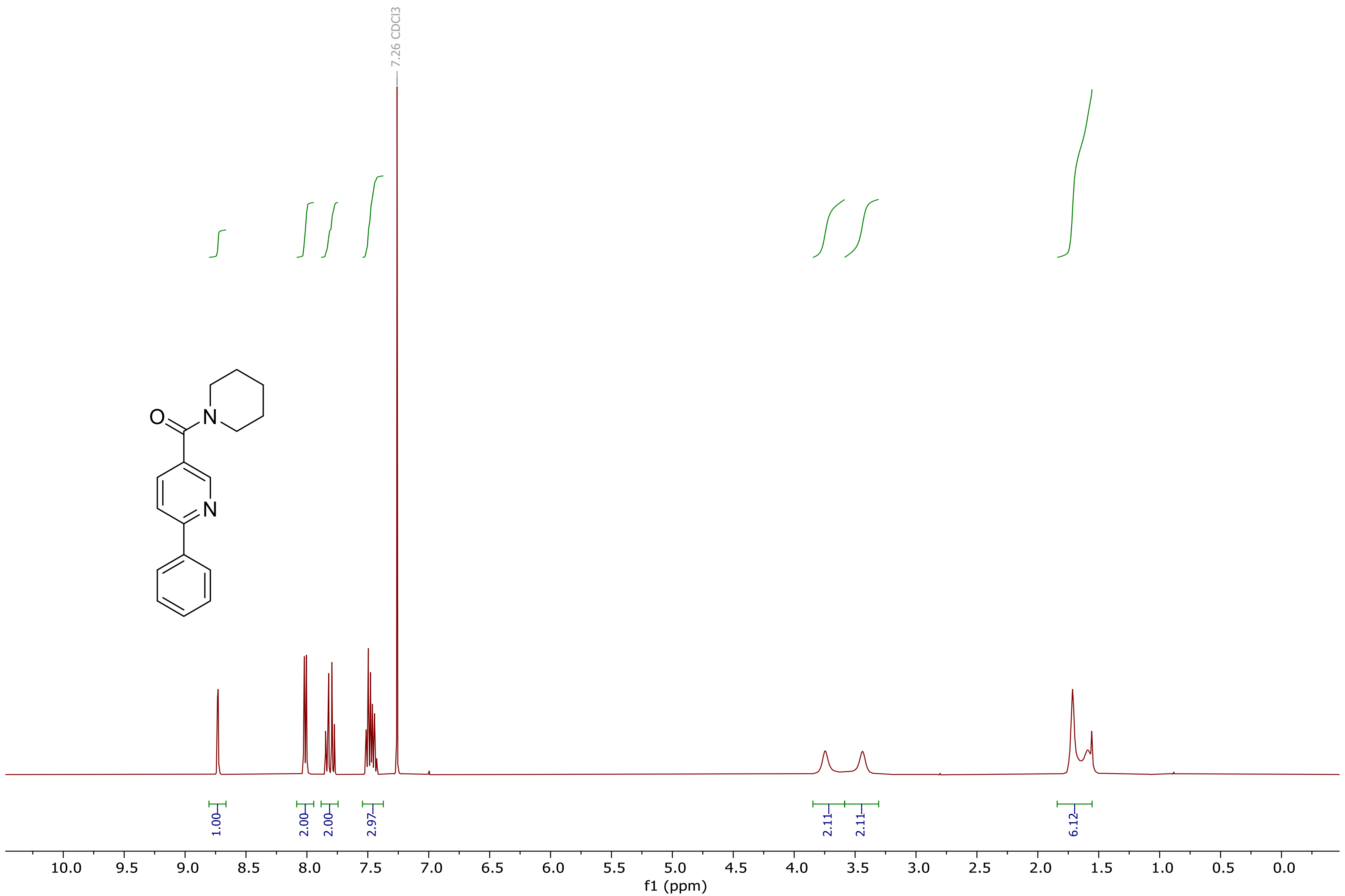

Supplementary Figure 59. ^{1}H NMR (400 MHz, $CDCl_3$) of (6-phenylpyridin-3-yl)-piperidin-1-ylmethanone.

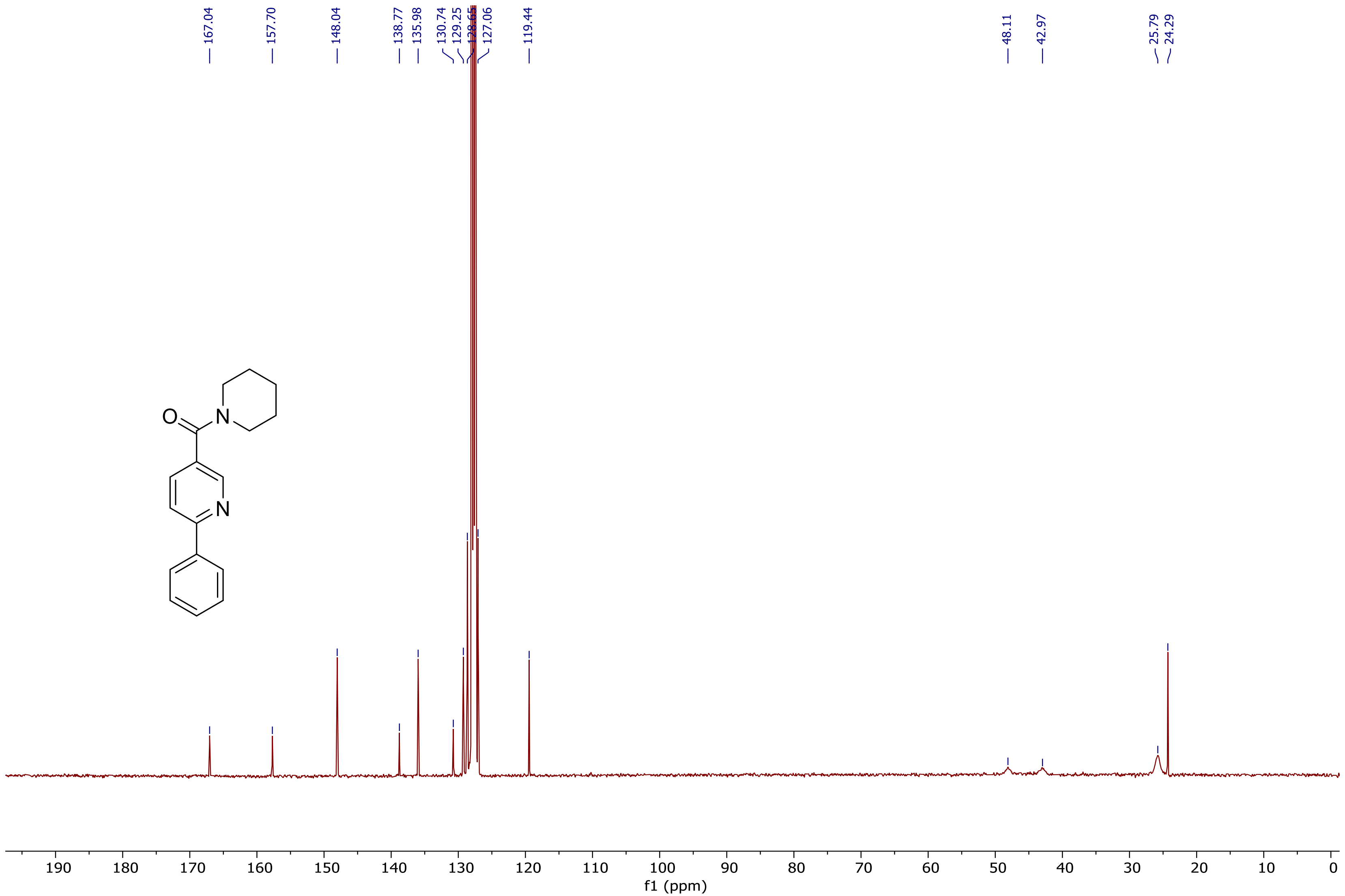

Supplementary Figure 60. ^{13}C NMR (101 MHz, C_6D_6) of (6-phenylpyridin-3-yl)-piperidin-1-ylmethanone

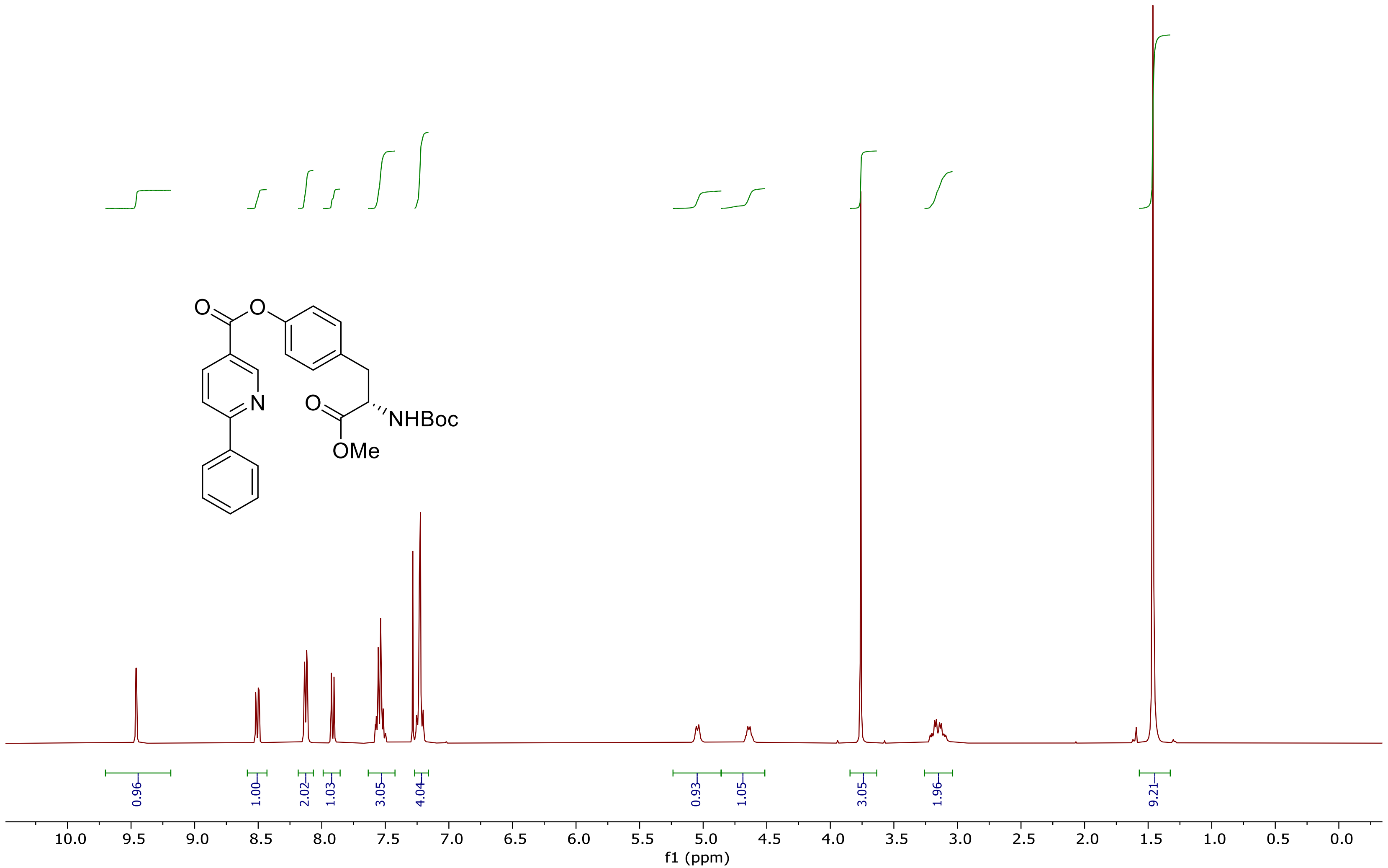

Supplementary Figure 61. ^{1}H NMR (400 MHz, $CDCl_3$) of (*S*)-4-{2-[(*tert*-butoxycarbonyl)amino]-3-methoxy-3-oxopropyl}phenyl 6-phenylnicotinate.

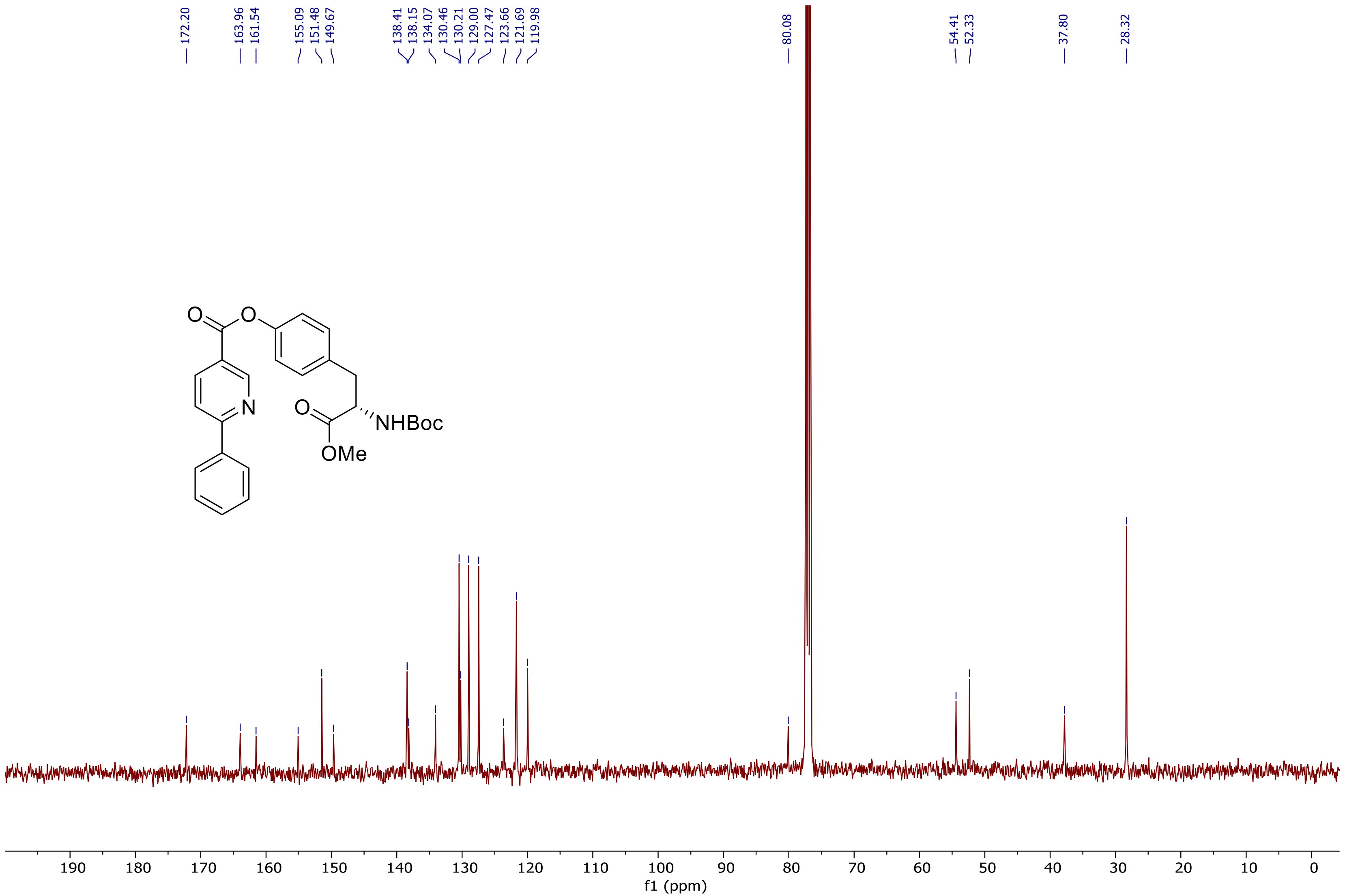

Supplementary Figure 62. ^{13}C NMR (101 MHz, $CDCl_3$) of (*S*)-4-{2-[(*tert*-butoxycarbonyl)amino]-3-methoxy-3-oxopropyl}phenyl 6-phenylnicotinate

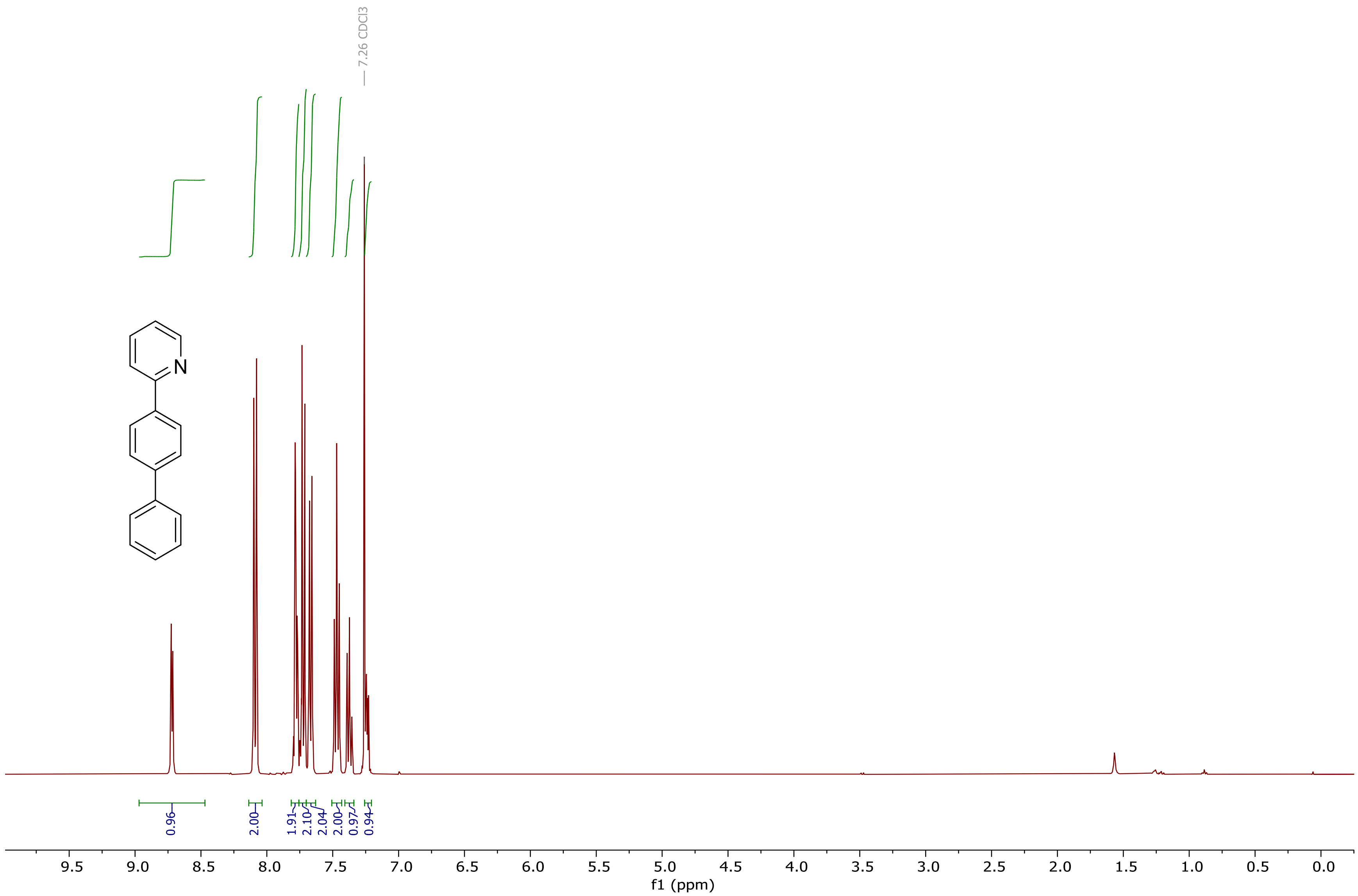

Supplementary Figure 63. ^{1}H NMR (400 MHz, $CDCl_3$) of 2-[(1,1'-biphenyl)-4-yl]-pyridine.

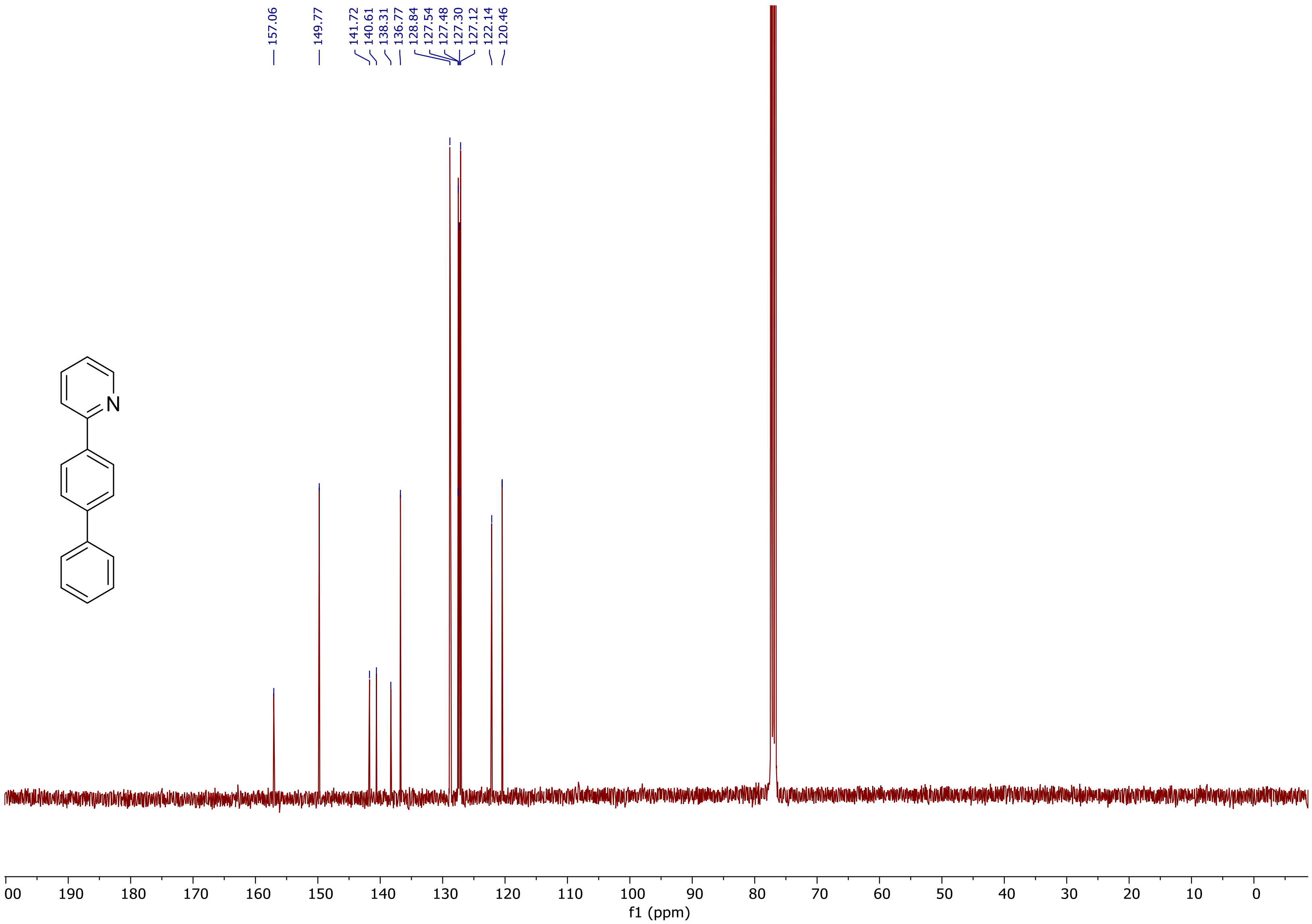

Supplementary Figure 64. ^{13}C NMR (101 MHz, $CDCl_3$) of 2-[(1,1'-biphenyl)-4-yl]-pyridine.

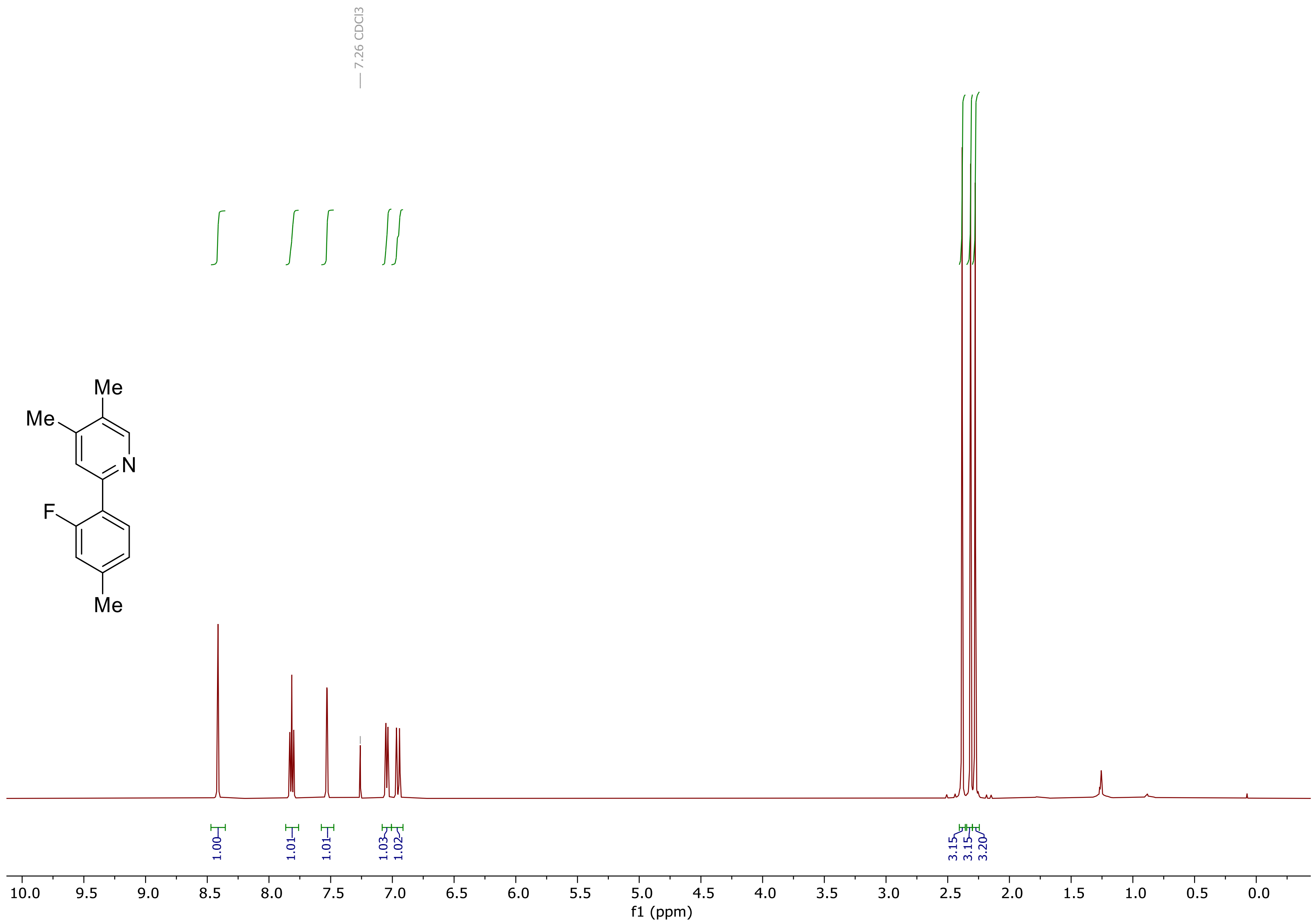

Supplementary Figure 65. ^1H NMR (500 MHz, $CDCl_3$) of 2-(2-fluoro-4-methylphenyl)-4,5-dimethylpyridine.

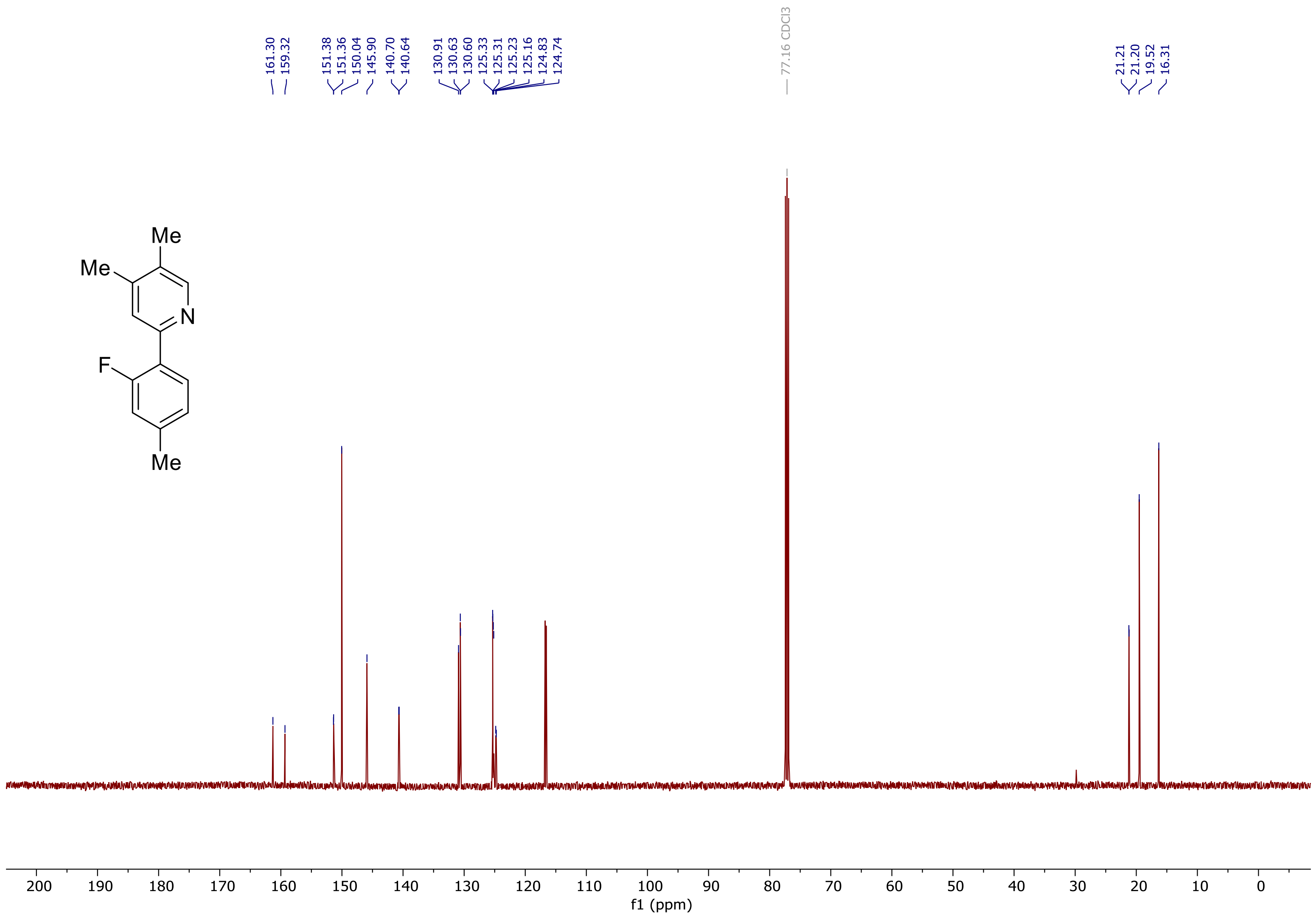

Supplementary Figure 66. ^{13}C NMR (126 MHz, $CDCl_3$) of 2-(2-fluoro-4-methylphenyl)-4,5-dimethylpyridine.

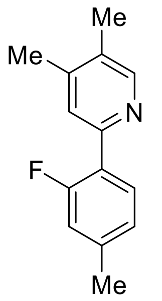

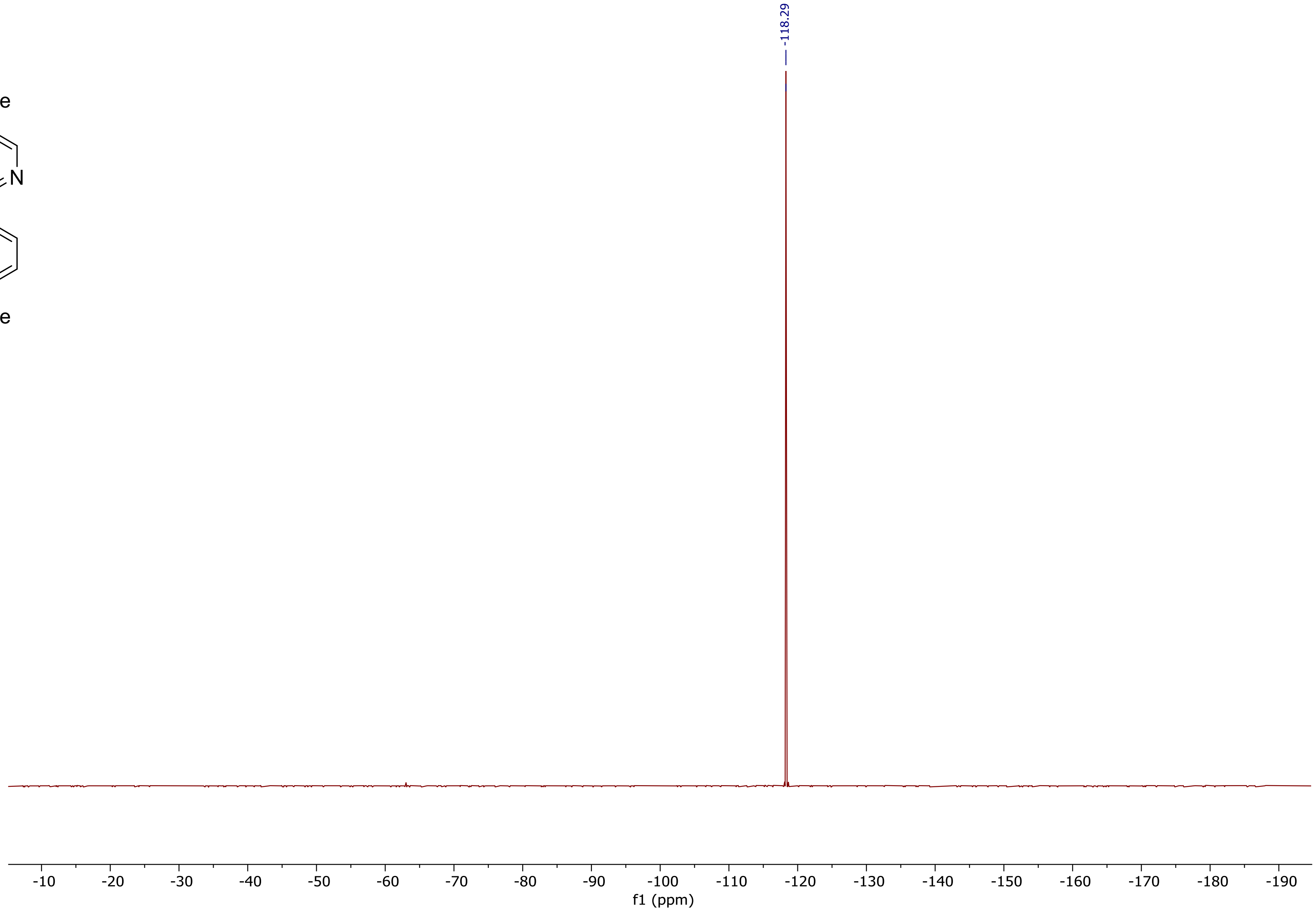

Supplementary Figure 67. ^{19}F NMR (471 MHz, $CDCl_3$) of 2-(2-fluoro-4-methylphenyl)-4,5-dimethylpyridine.

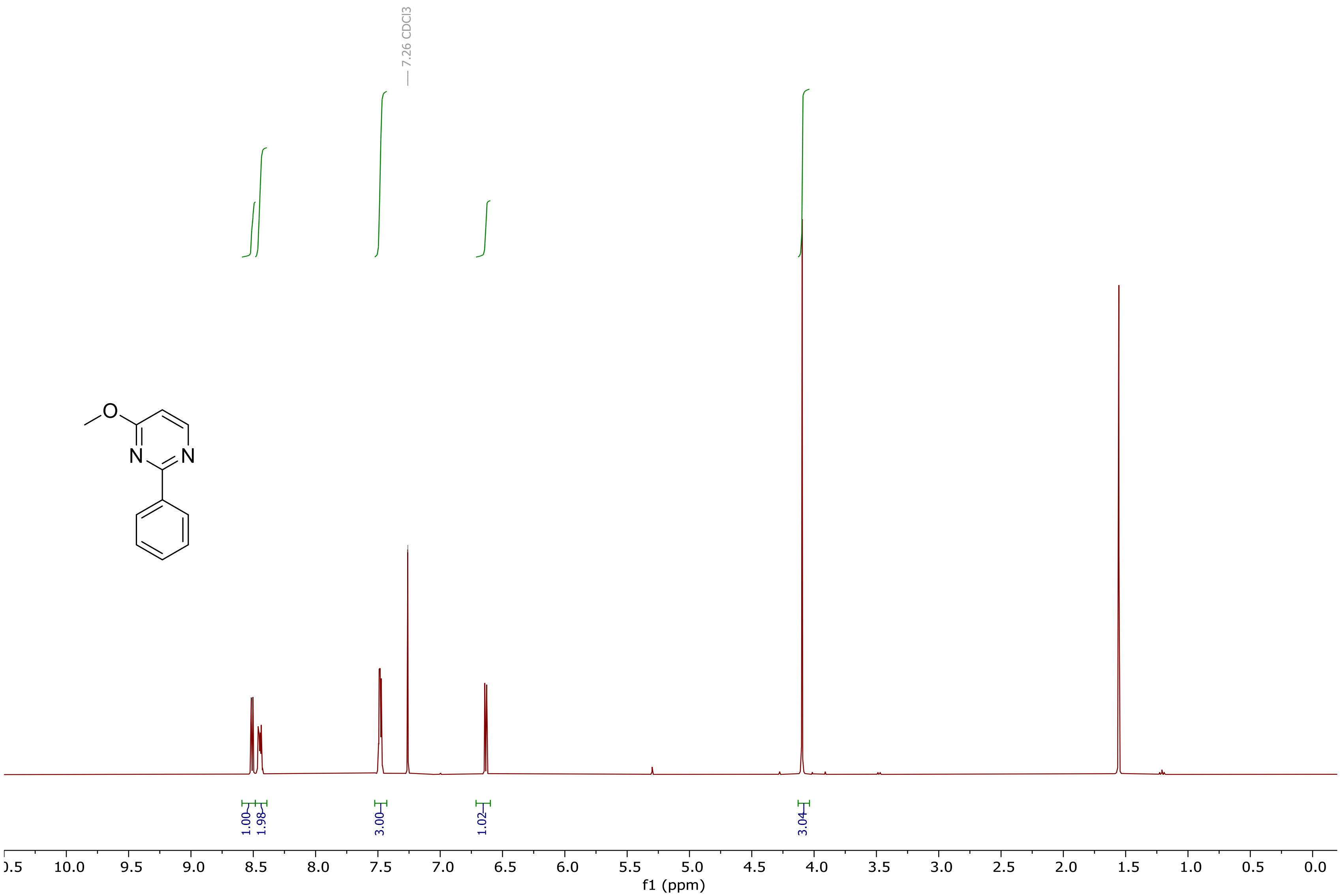

Supplementary Figure 68. ^{1}H NMR (400 MHz, $CDCl_3$) of 2-phenyl-4-methoxypyrimidine.

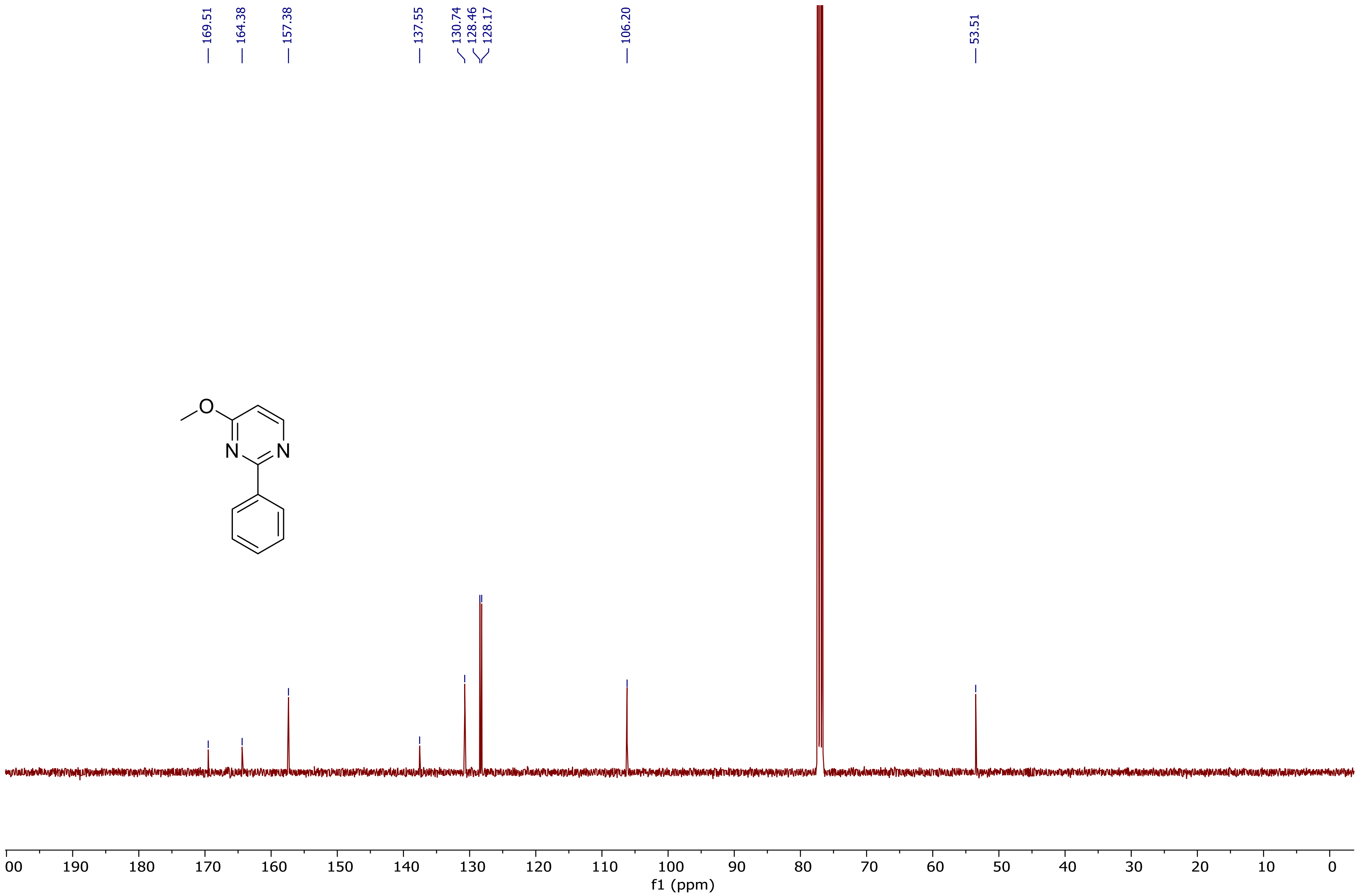

Supplementary Figure 69. ^{13}C NMR (101 MHz, $CDCl_3$) of 2-phenyl-4-methoxypyrimidine.

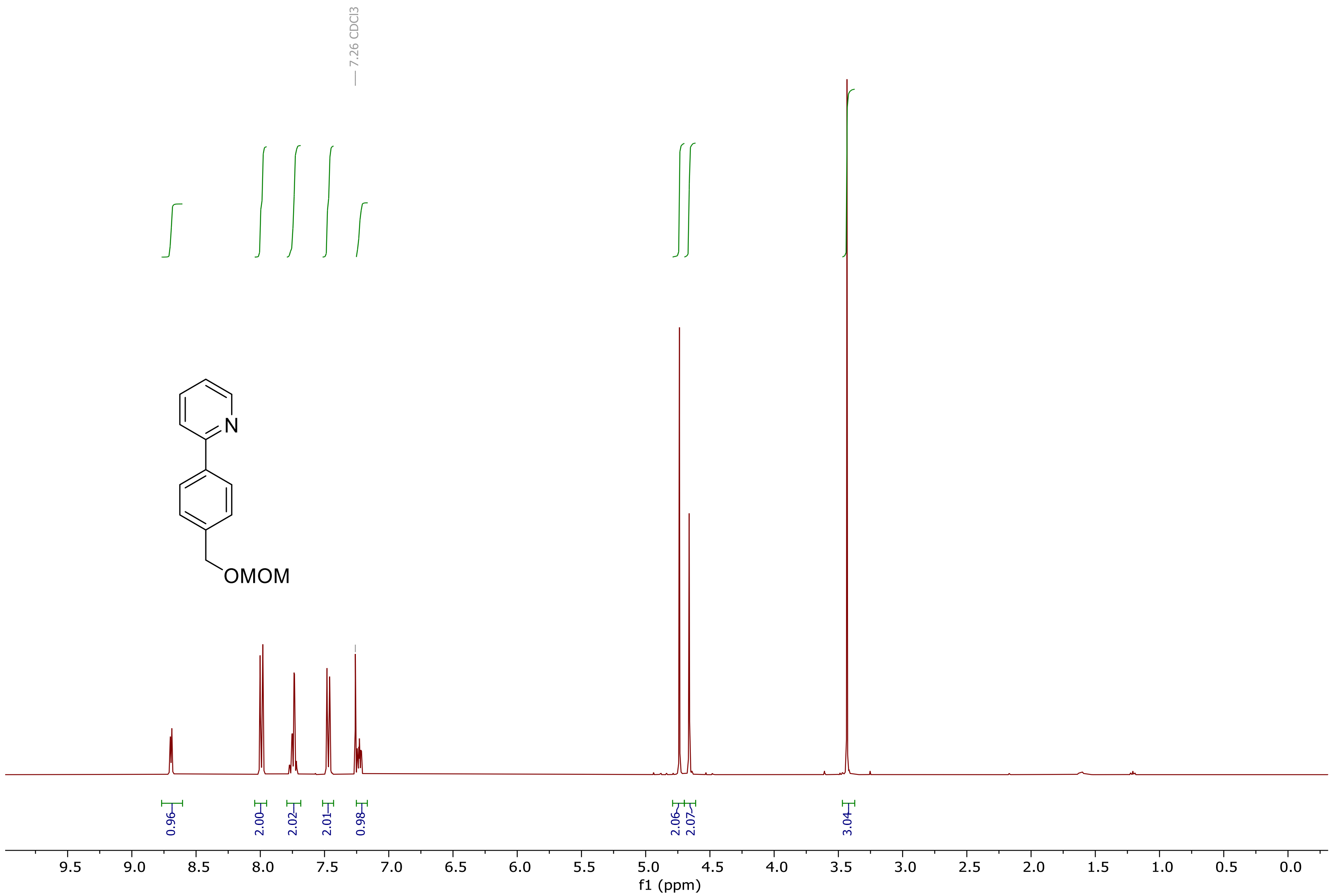

Supplementary Figure 70. ^{1}H NMR (400 MHz, $CDCl_3$) of 2-{4-[(methoxymethoxy)methyl]phenyl}pyridine.

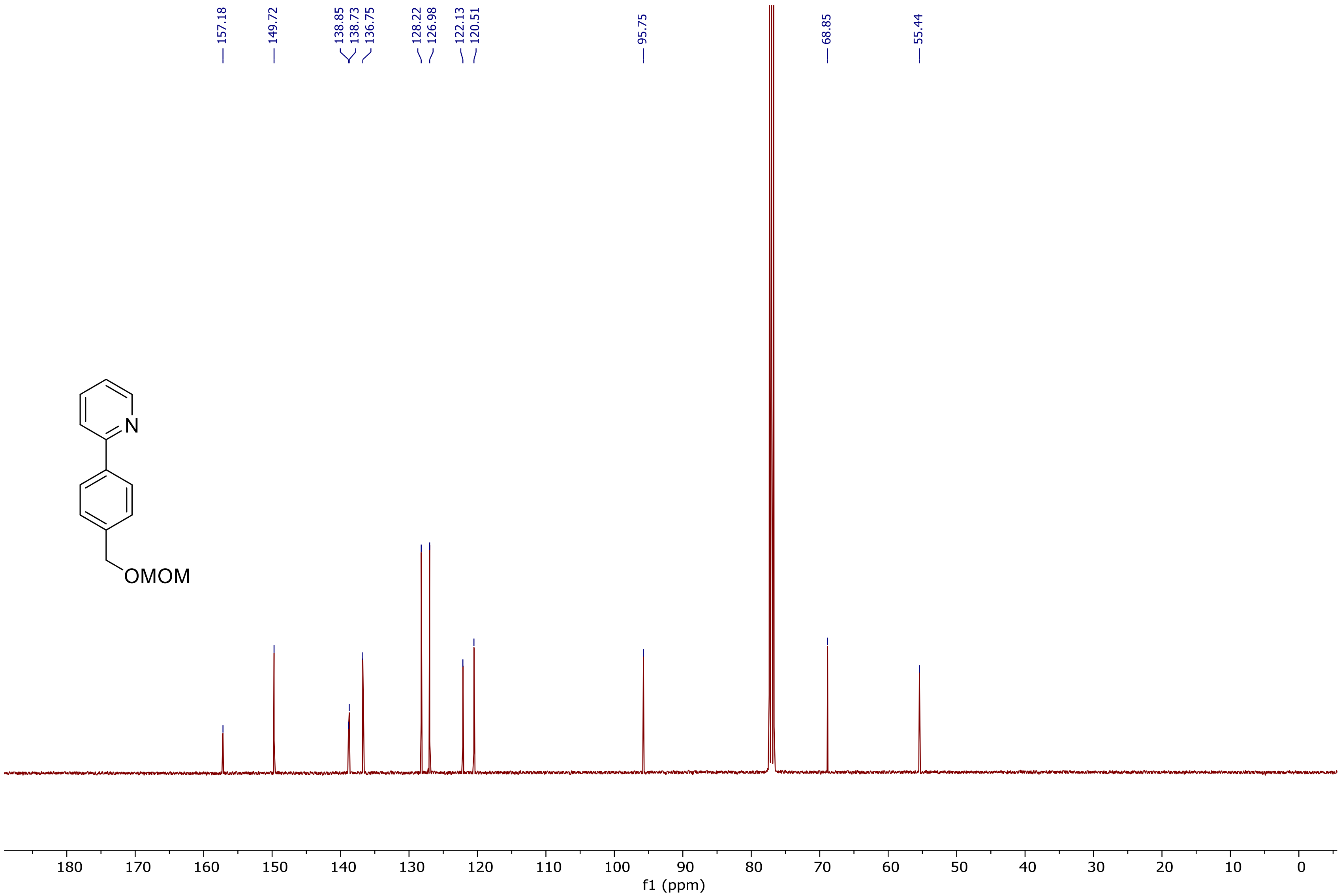

Supplementary Figure 71. ^{13}C NMR (101 MHz, $CDCl_3$) of 2-{4-[(methoxymethoxy)methyl]phenyl}pyridine.

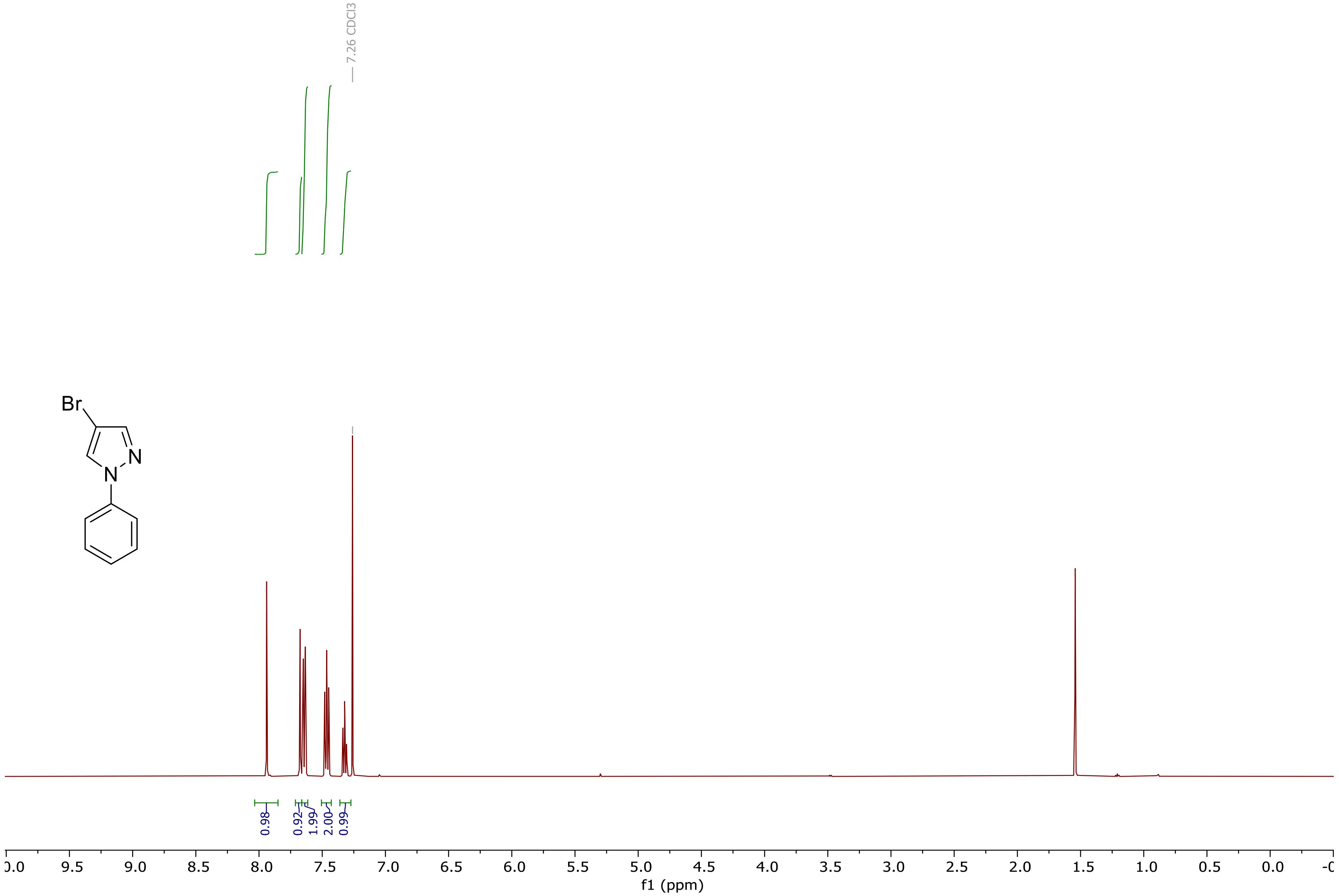

Supplementary Figure 72. ^{1}H NMR (500 MHz, $CDCl_3$) of 4-bromo-1-phenyl-1H-pyrazole.

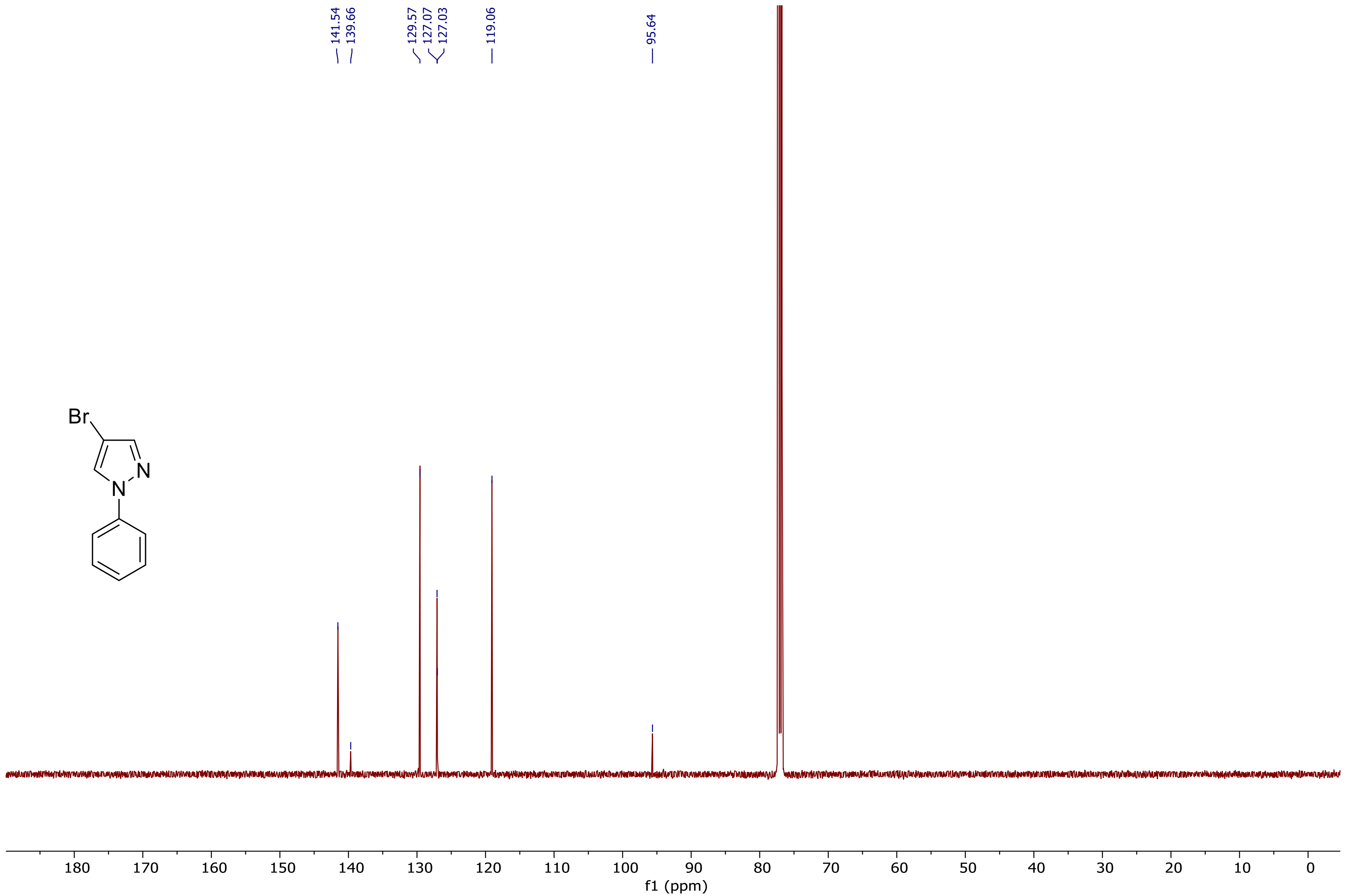

Supplementary Figure 73. ^{1}H NMR (126 MHz, $CDCl_3$) of 4-bromo-1-phenyl-1H-pyrazole.

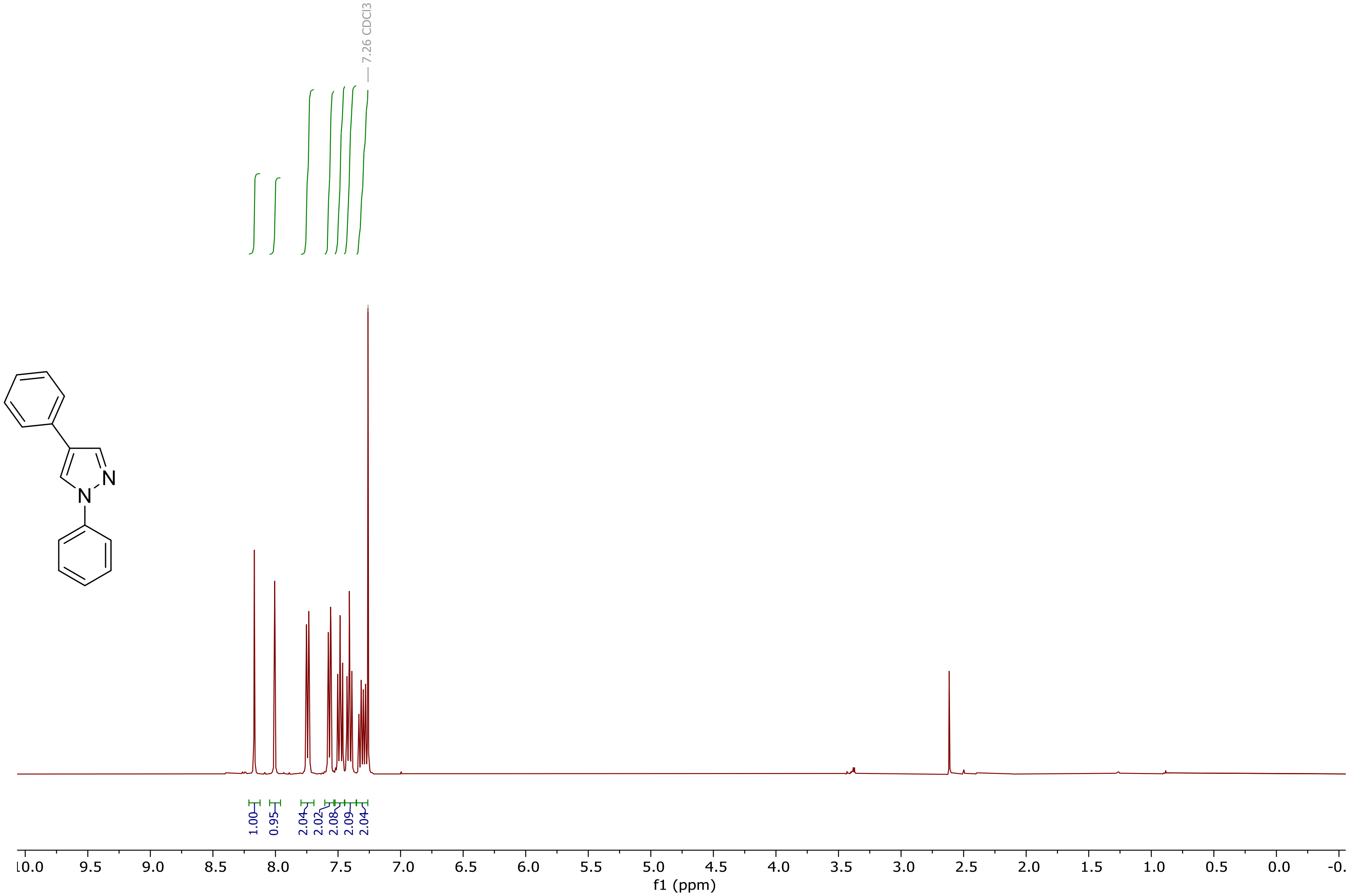

Supplementary Figure 74. ^{1}H NMR (400 MHz, $CDCl_3$) of 1,4-diphenyl-1H-pyrazole.

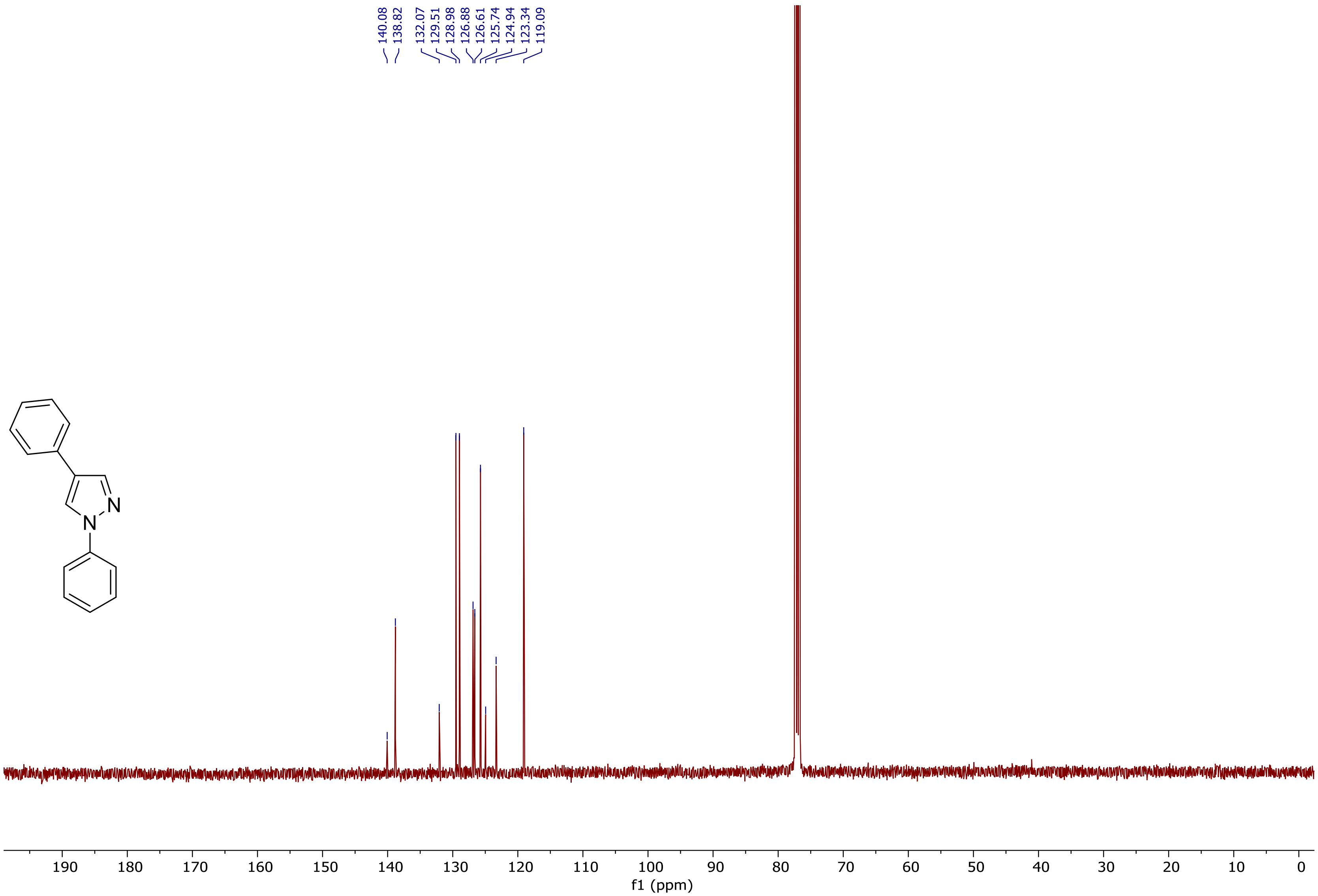

Supplementary Figure 75. ^{13}C NMR (101 MHz, $CDCl_3$) of 1,4-diphenyl-1H-pyrazole.

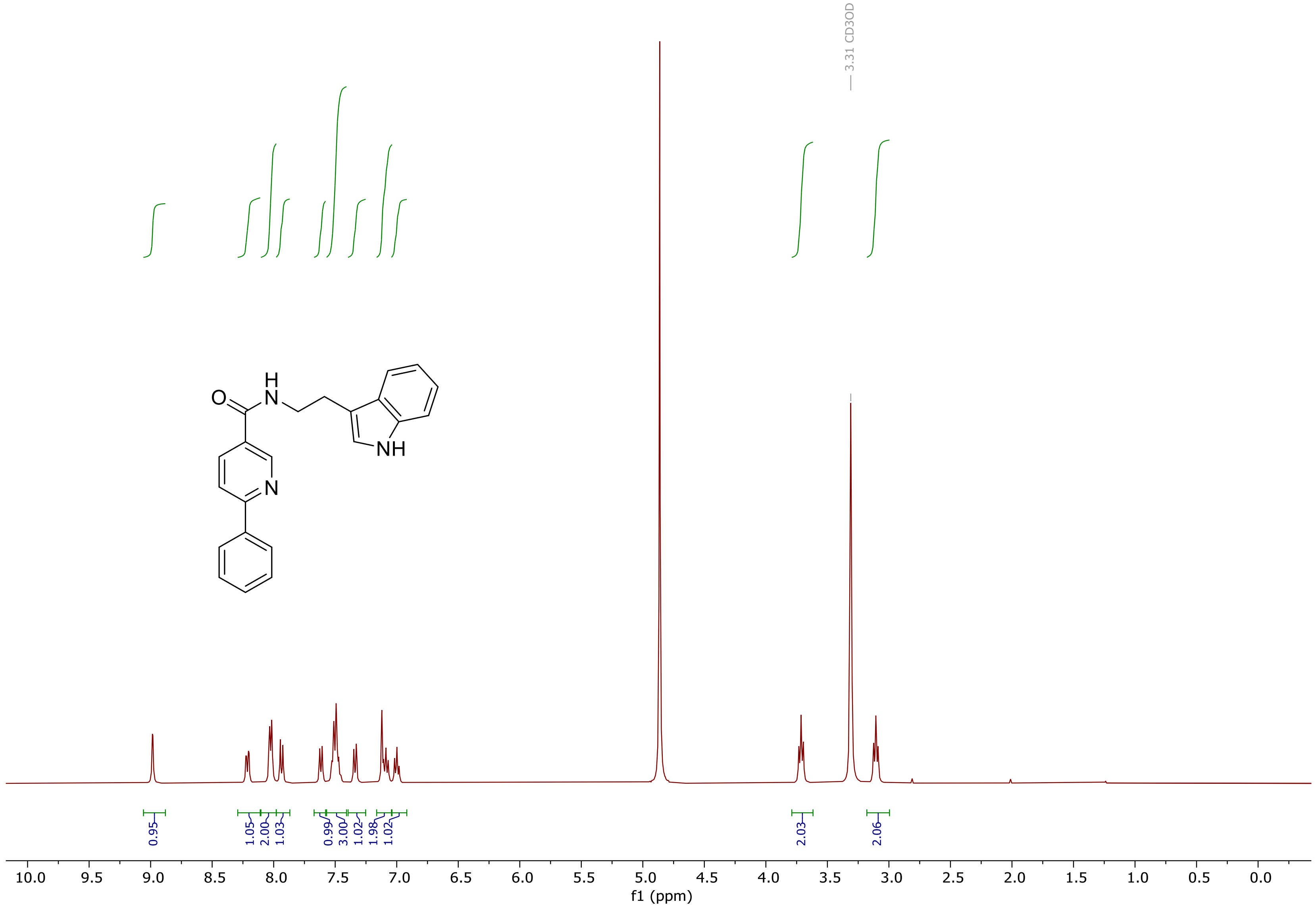

Supplementary Figure 76. ^{1}H NMR (400 MHz, d_4-MeOD) of *N*-[2-(1*H*-indol-3-yl)ethyl]-6-phenylnicotinamide.

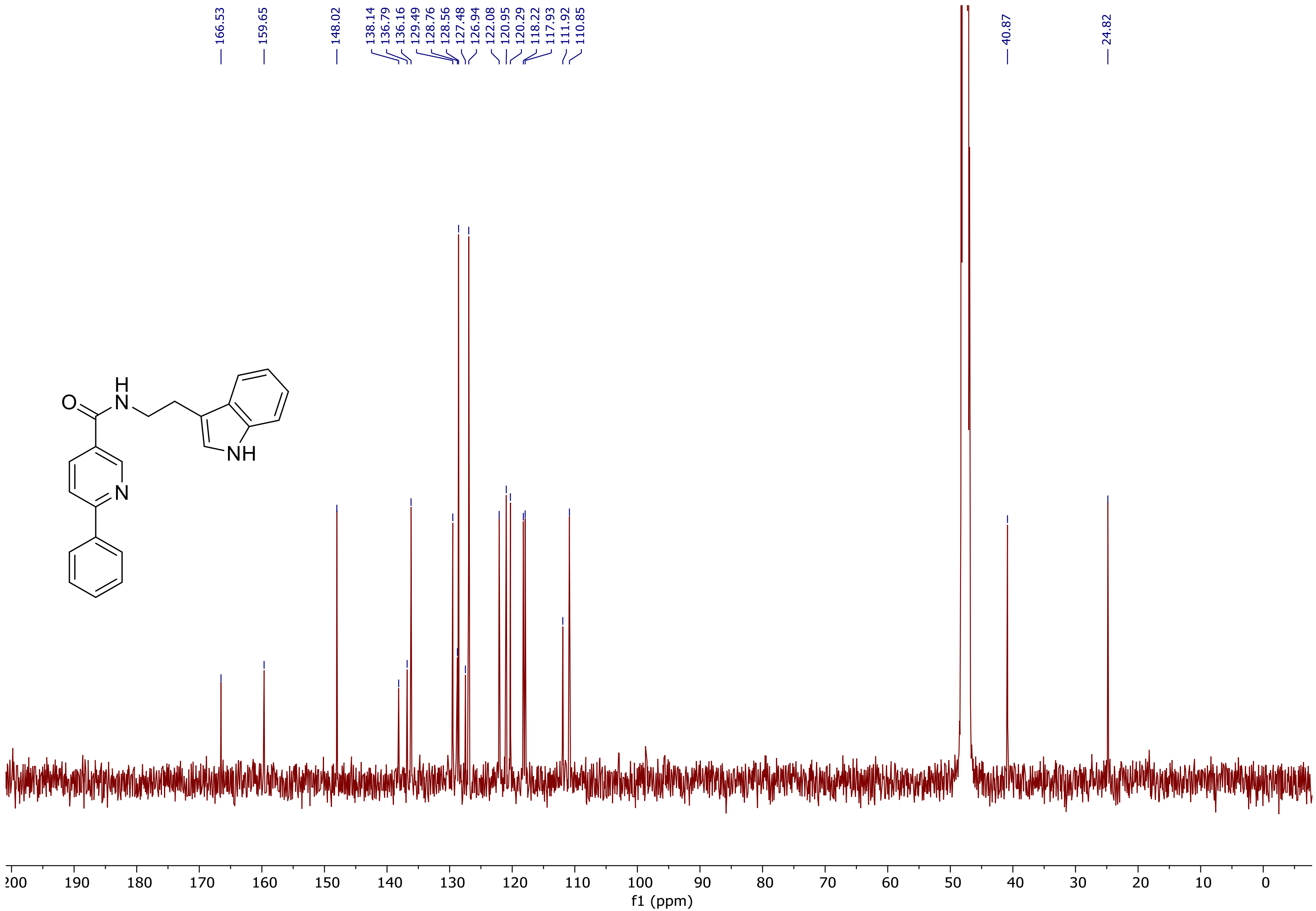

Supplementary Figure 77. ^{13}C NMR (101 MHz, d_4-MeOD) of *N*-[2-(1*H*-indol-3-yl)ethyl]-6-phenylnicotinamide.

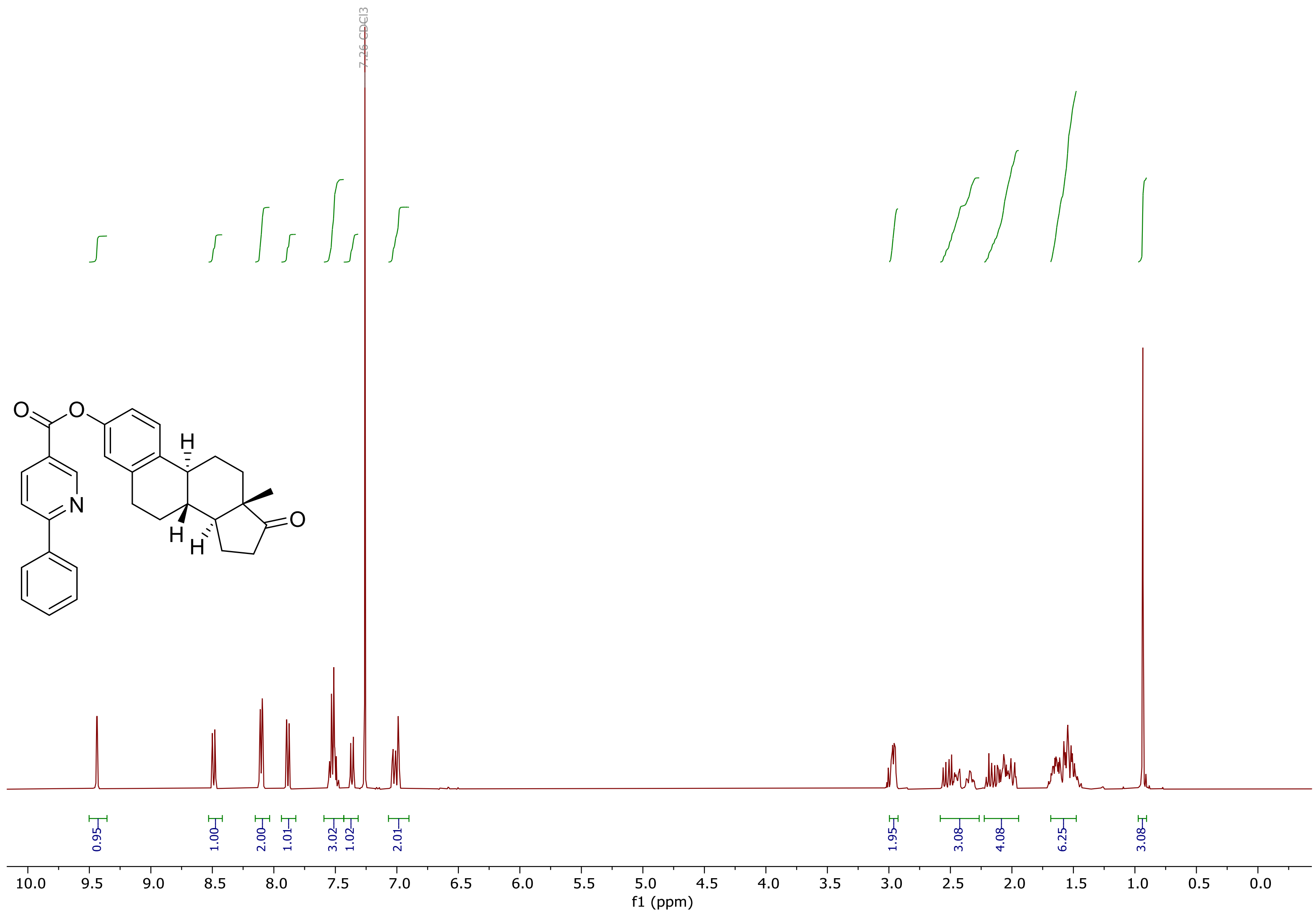

Supplementary Figure 78. ^{1}H NMR (400 MHz, $CDCl_3$) of (8R,9S,13S,14S)-13-methyl-17-oxo-7,8,9,11,12,13,14,15,16,17-decahydro-6H-cyclopenta[a]phenanthren-3-yl 6-phenylnicotinate.

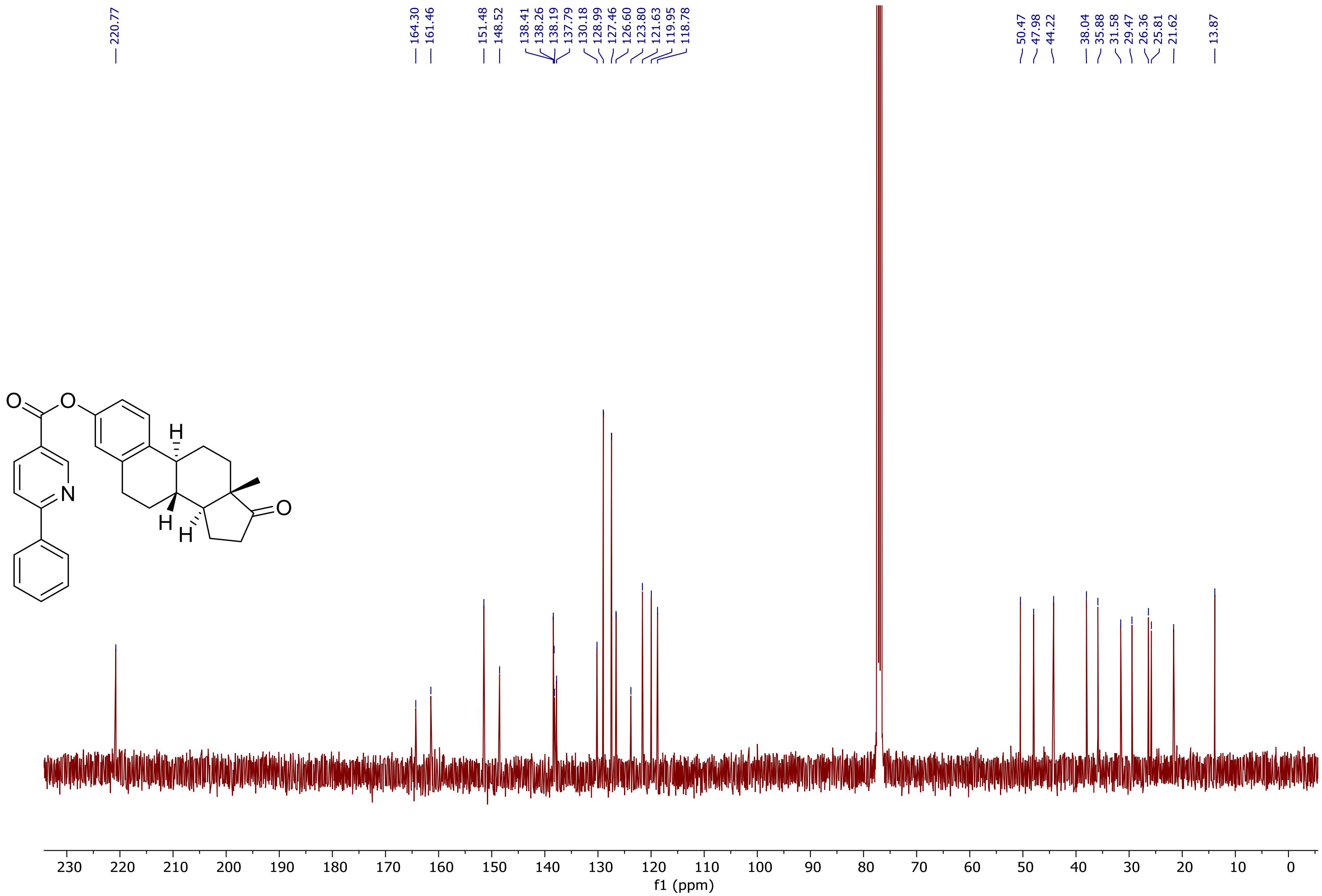

Supplementary Figure 79. ^{13}C NMR (101 MHz, $CDCl_3$) of (8R,9S,13S,14S)-13-methyl-17-oxo-7,8,9,11,12,13,14,15,16,17-decahydro-6H-cyclopenta[a]phenanthren-3-yl 6-phenylnicotinate.

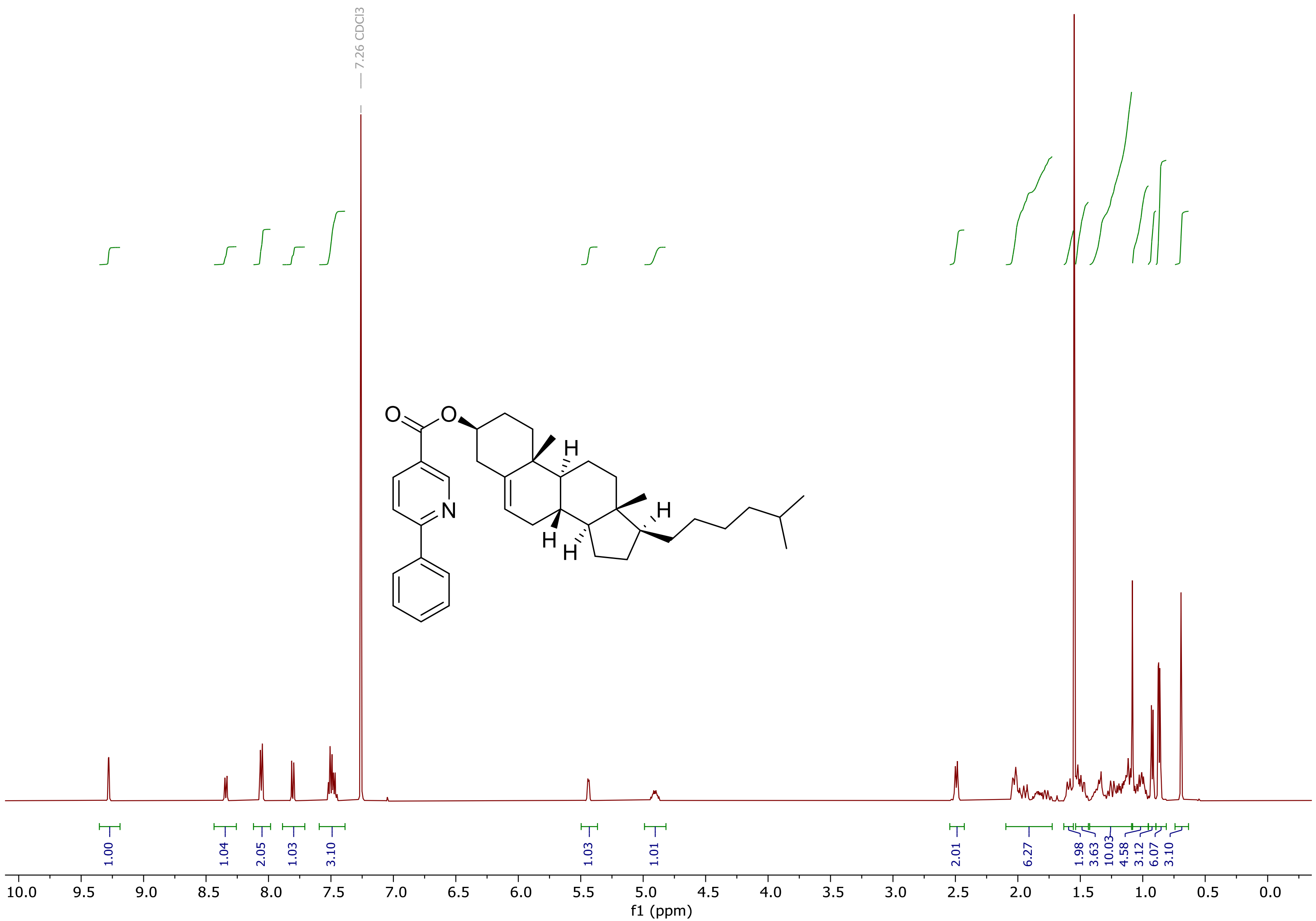

Supplementary Figure 80. ^{1}H NMR (500 MHz, $CDCl_3$) of (3S,8S,9S,10R,13R,14S,17S)-10,13-dimethyl-17-(5-methylhexyl)-2,3,4,7,8,9,10,11,12,13,14,15,16,17-tetradecahydro-*1H*-cyclopenta[a]phenanthren-3-yl 6-phenylnicotinate.

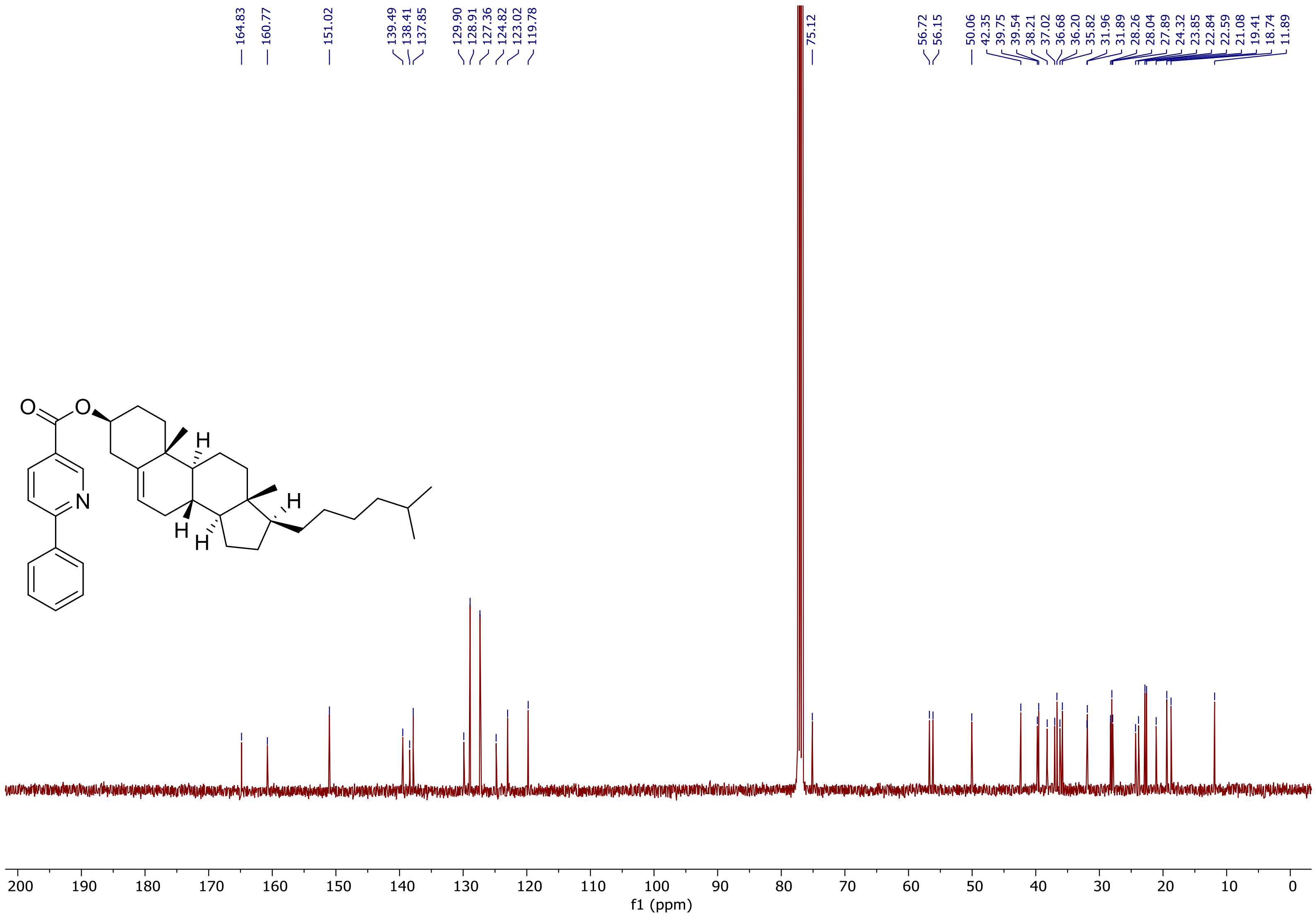

Supplementary Figure 81. ^{13}C NMR (101 MHz, $CDCl_3$) of (3S,8S,9S,10R,13R,14S,17S)-10,13-dimethyl-17-(5-methylhexyl)-2,3,4,7,8,9,10,11,12,13,14,15,16,17-tetradecahydro-*1H*-cyclopenta[a]phenanthren-3-yl 6-phenylnicotinate.

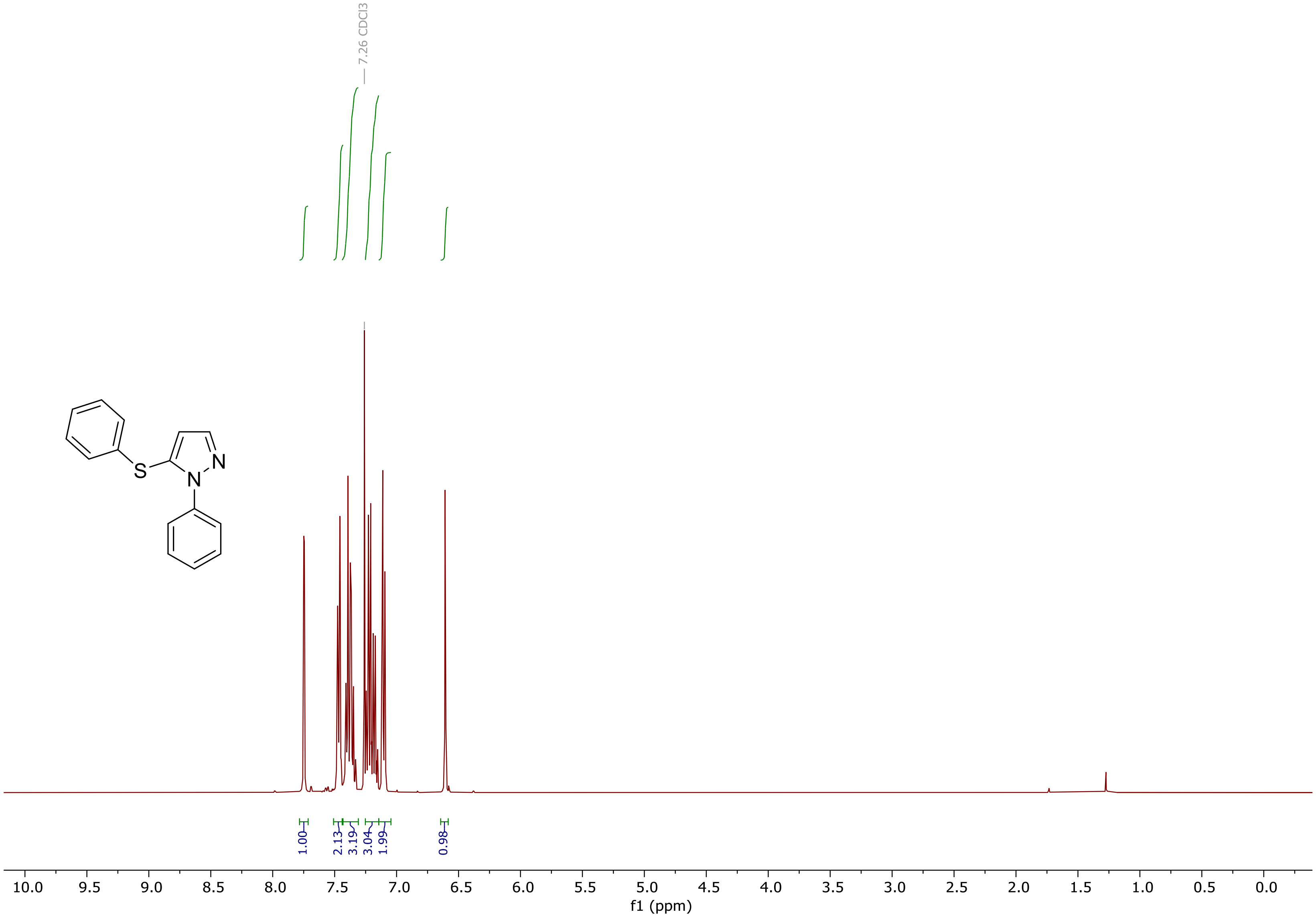

Supplementary Figure 82. ^{1}H NMR (400 MHz, $CDCl_3$) of 1-phenyl-5-(phenylthio)-*1H*-pyrazole.

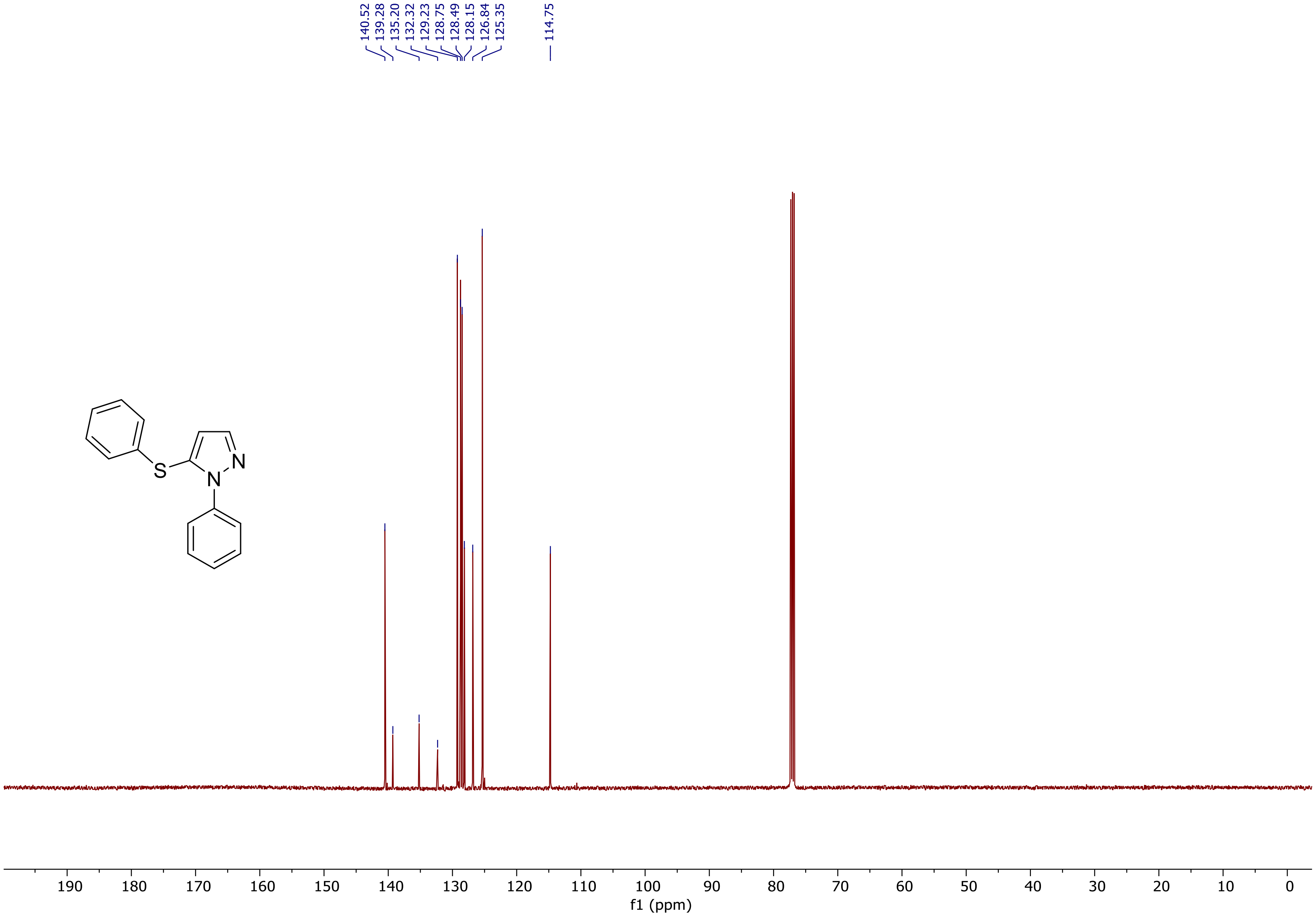

Supplementary Figure 83. ^{13}C NMR (126 MHz, $CDCl_3$) of 1-phenyl-5-(phenylthio)-*1H*-pyrazole.

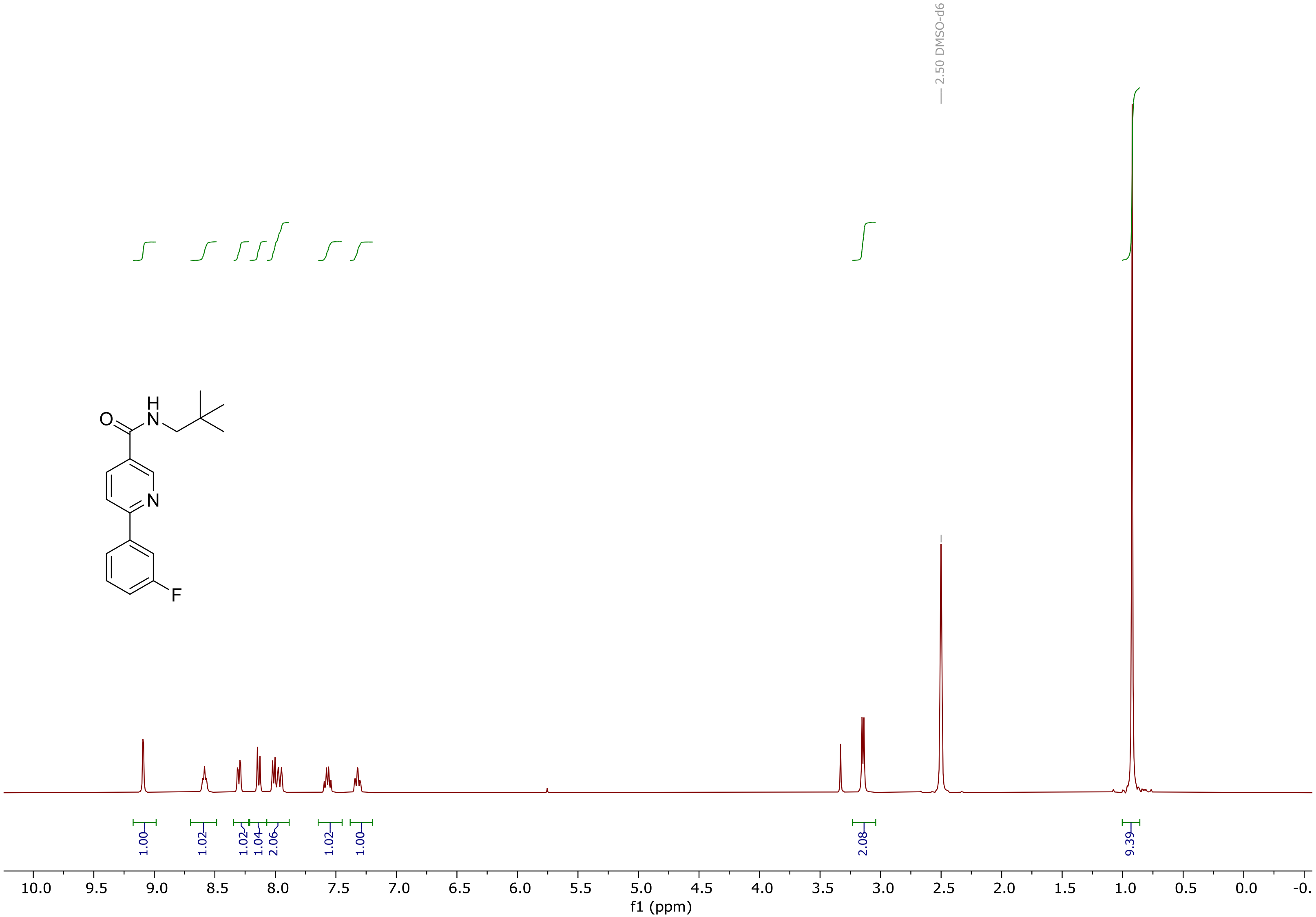

Supplementary Figure 84. ^{1}H NMR (400 MHz, d_6-Me_2SO) of 6-(3-fluorophenyl)-*N*-neopentylnicotinamide **2ac**.

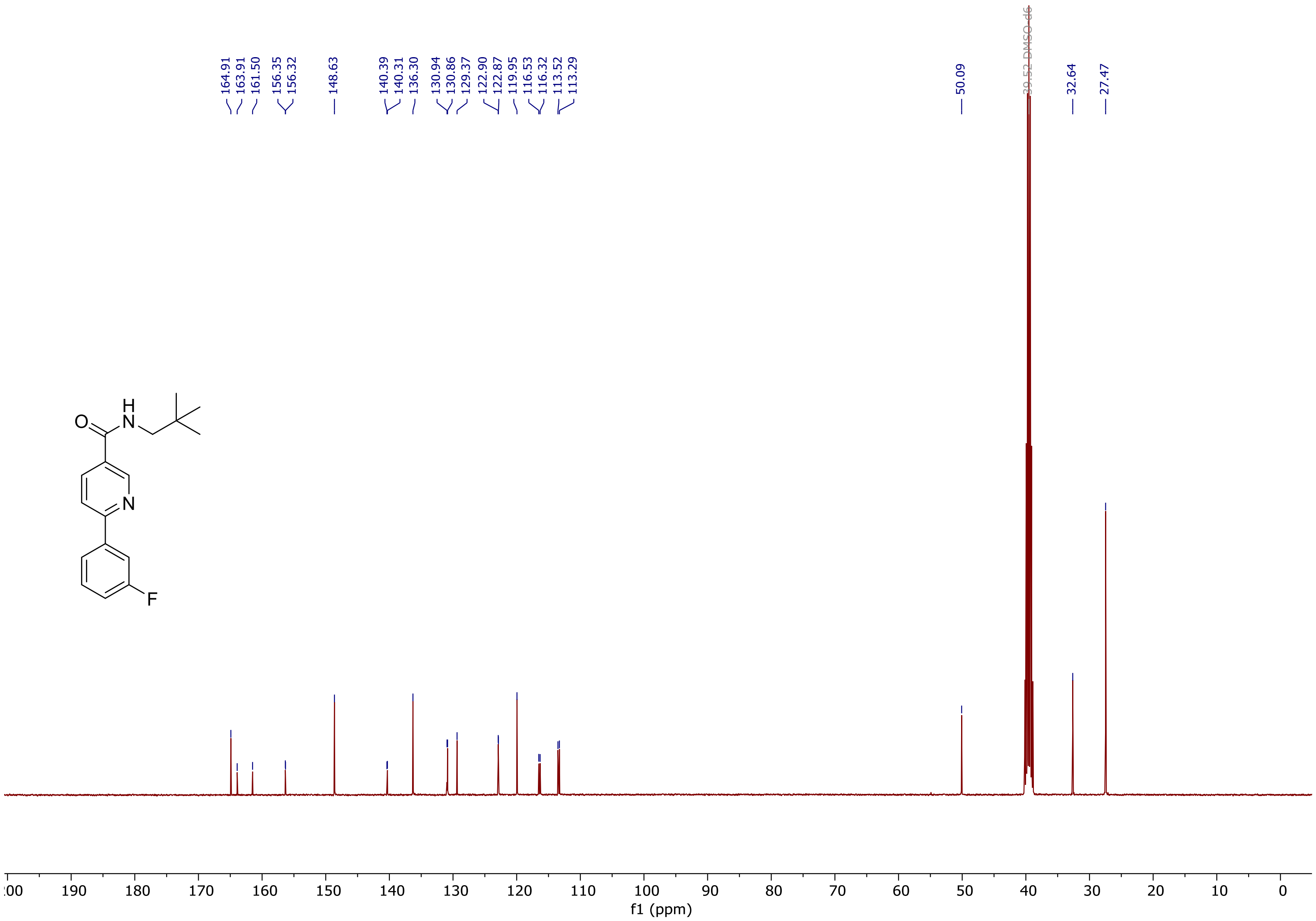

Supplementary Figure 85. ^{13}C NMR (101 MHz, d_6-Me_2SO) of 6-(3-fluorophenyl)-*N*-neopentylnicotinamide **2ac**.

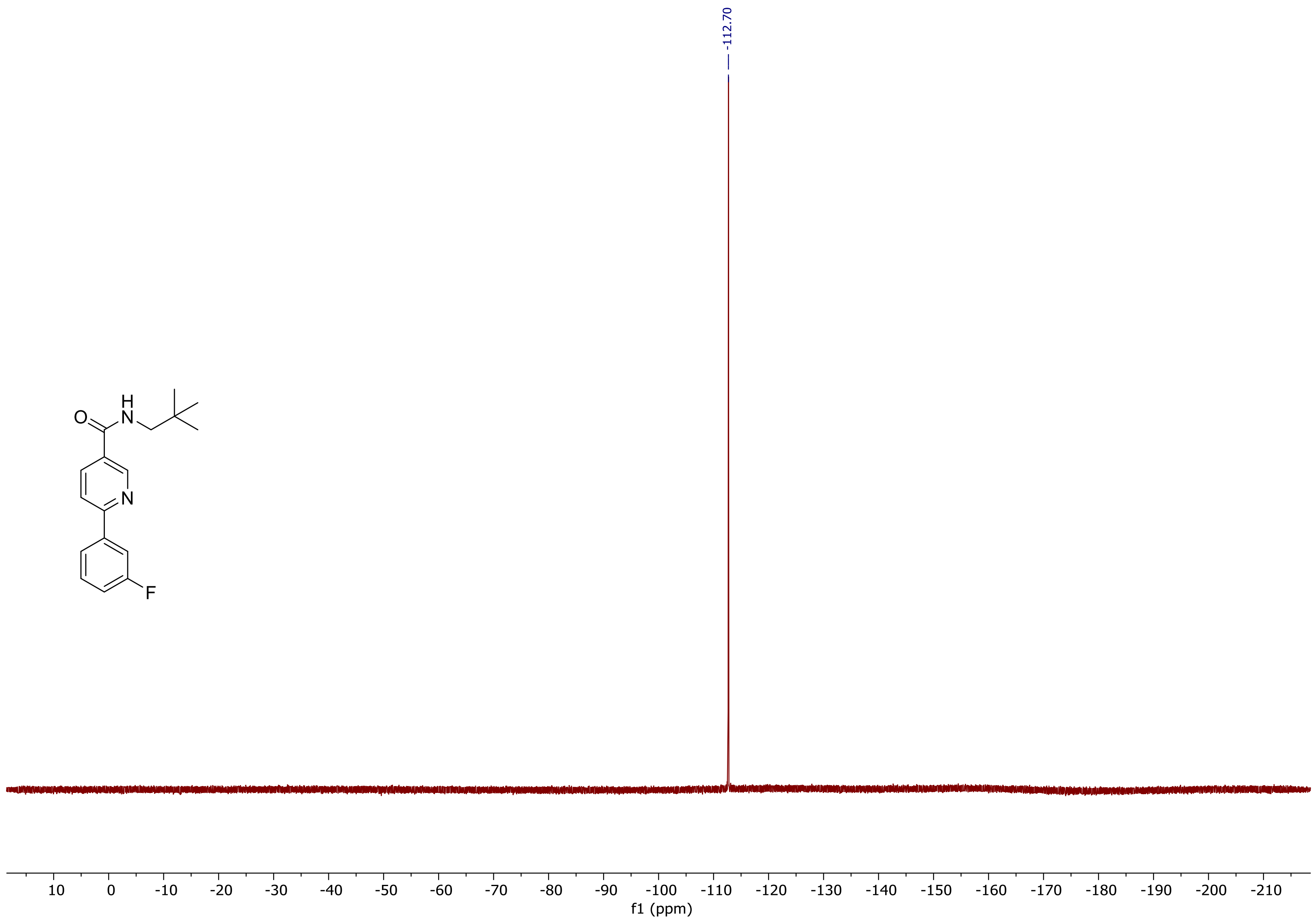

Supplementary Figure 86. ^{19}F NMR (376 MHz, d_6-Me_2SO) of 6-(3-fluorophenyl)-*N*-neopentylnicotinamide **2ac**.

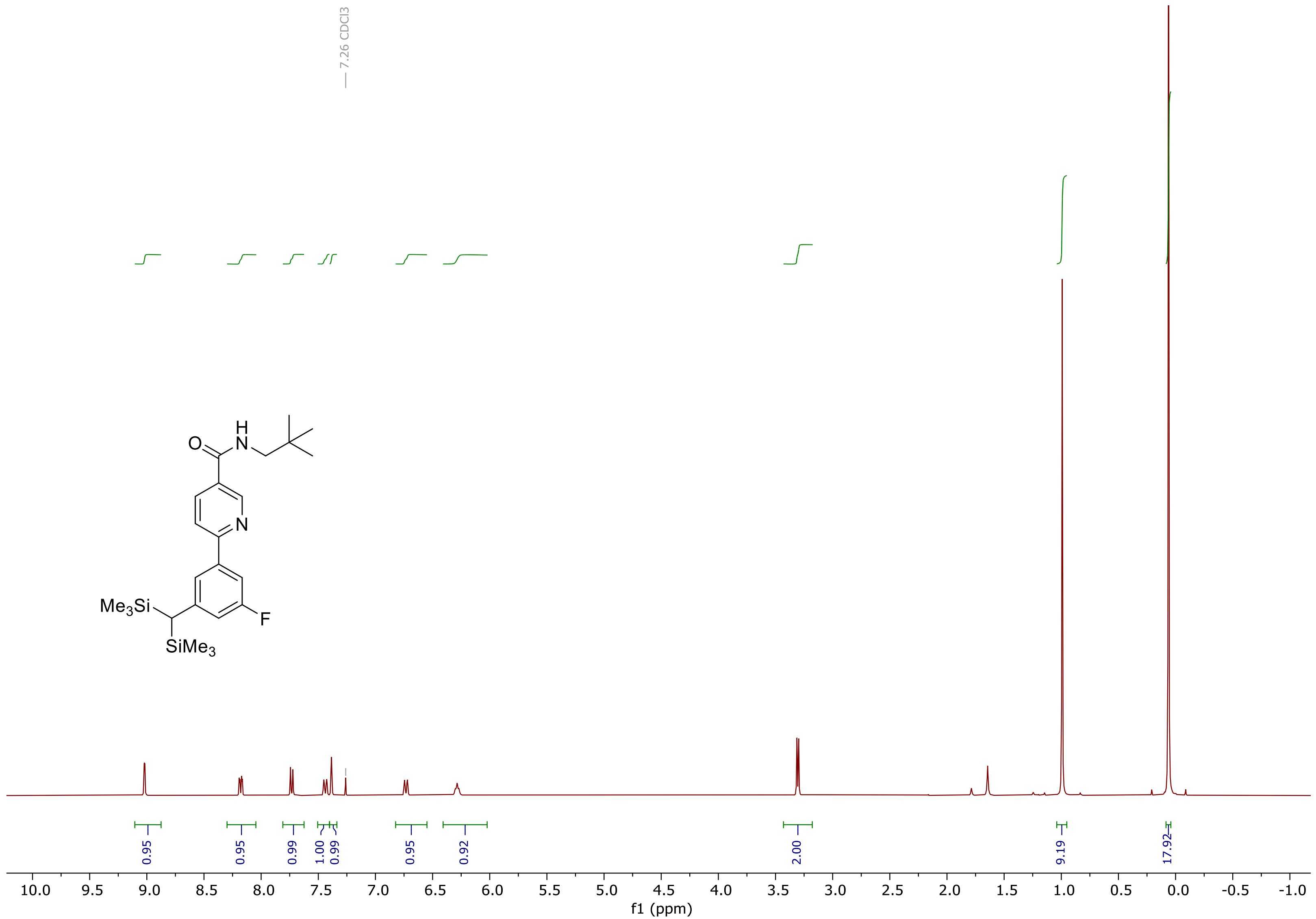

Supplementary Figure 87. ^{1}H NMR (400 MHz, $CDCl_3$) of 6-{3-[bis(trimethylsilyl)methyl]-5-fluorophenyl}-*N*-neopentylnicotinamide **6w**.

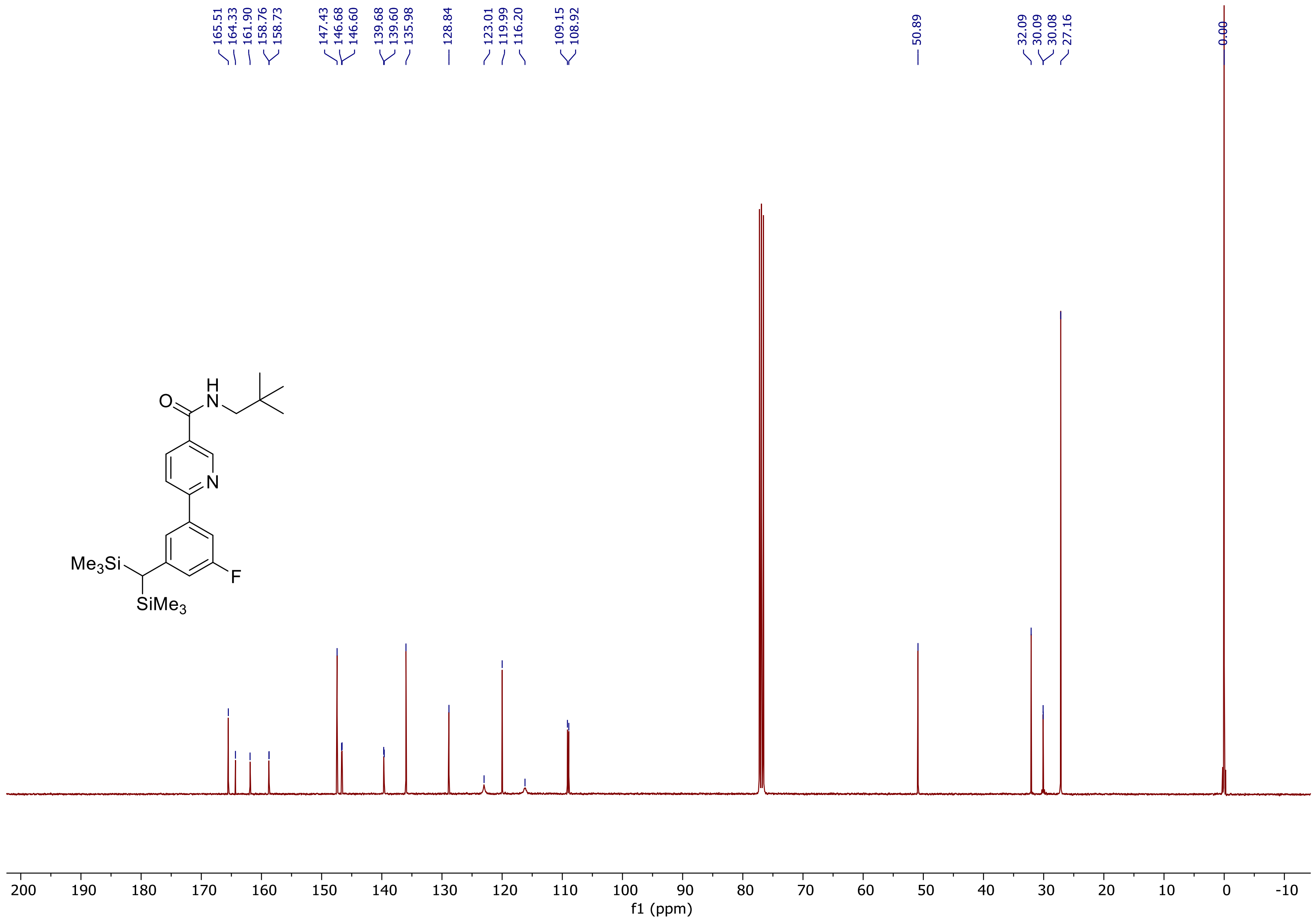

Supplementary Figure 88. ^{13}C NMR (101 MHz, $CDCl_3$) of 6-{3-[bis(trimethylsilyl)methyl]-5-fluorophenyl}-*N*-neopentylnicotinamide **6w**.

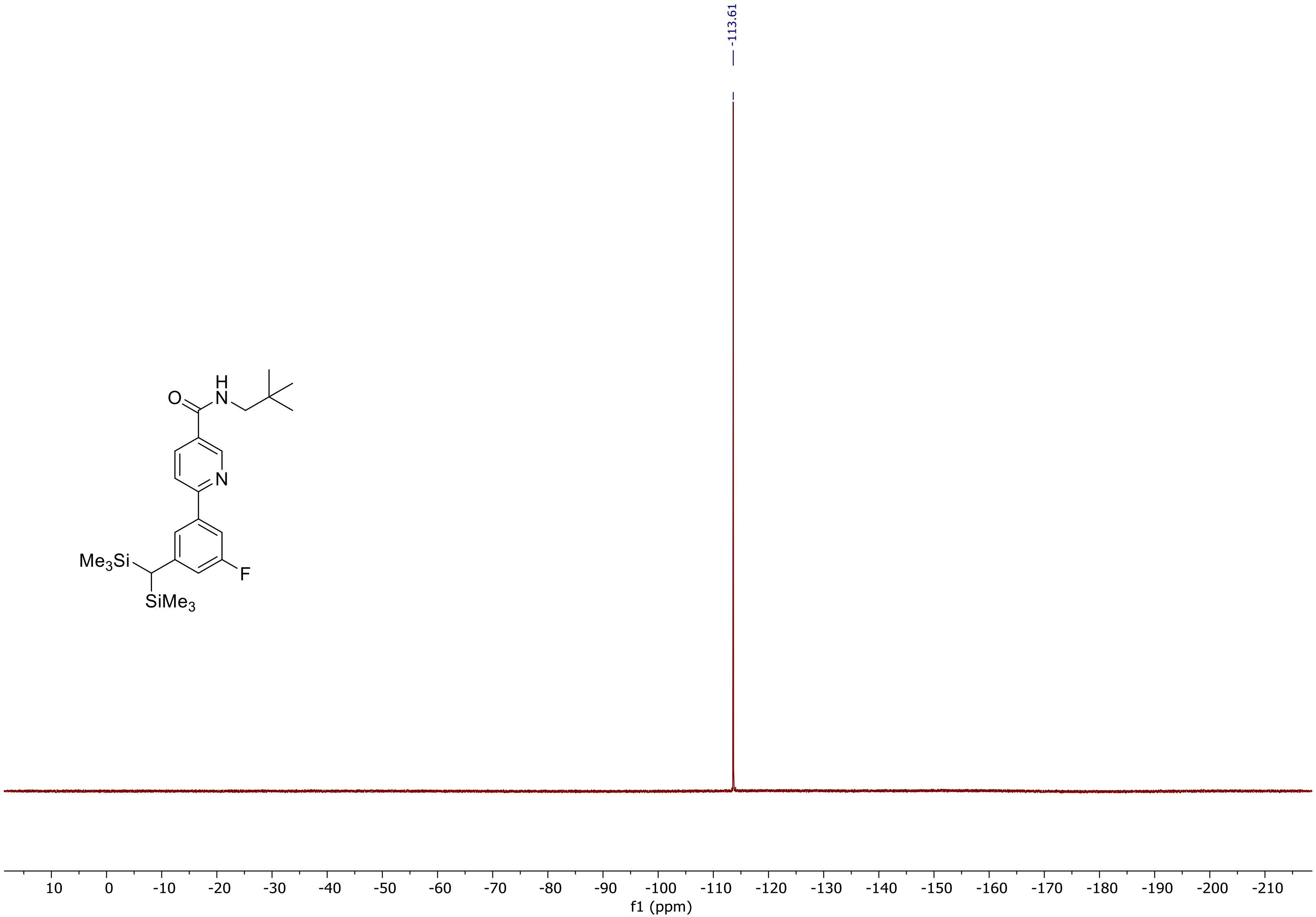

Supplementary Figure 89. ^{19}F NMR (376 MHz, $CDCl_3$) of 6-{3-[bis(trimethylsilyl)methyl]-5-fluorophenyl}-*N*-neopentylnicotinamide **6w**.

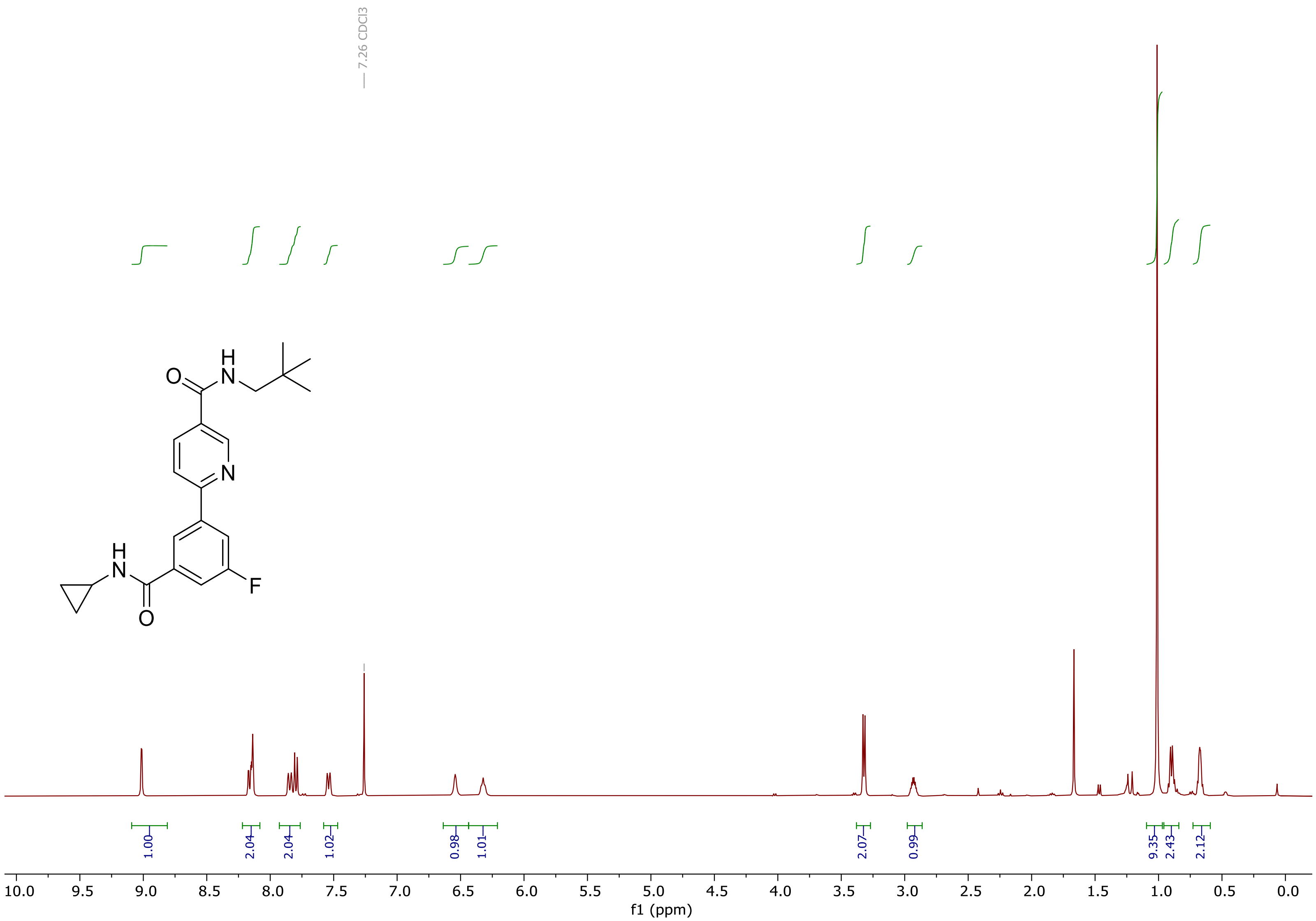

Supplementary Figure 90. ^{1}H NMR (400 MHz, $CDCl_3$) of 6-[3-(cyclopropylcarbamoyl)-5-fluorophenyl]-*N*-neopentylnicotinamide **7**.

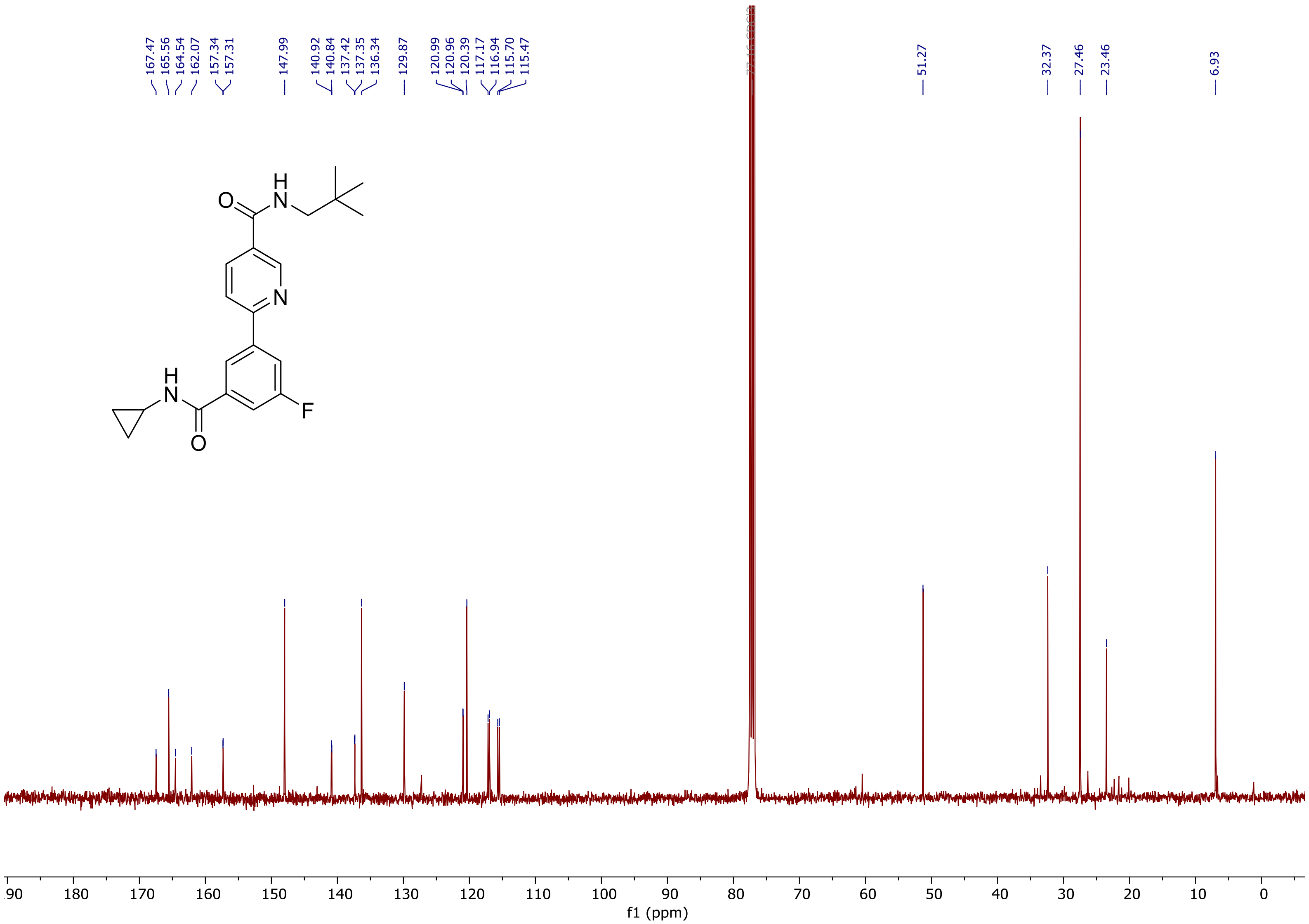

Supplementary Figure 91. ^{13}C NMR (101 MHz, $CDCl_3$) of 6-[3-(cyclopropylcarbamoyl)-5-fluorophenyl]-*N*-neopentylnicotinamide **7**.

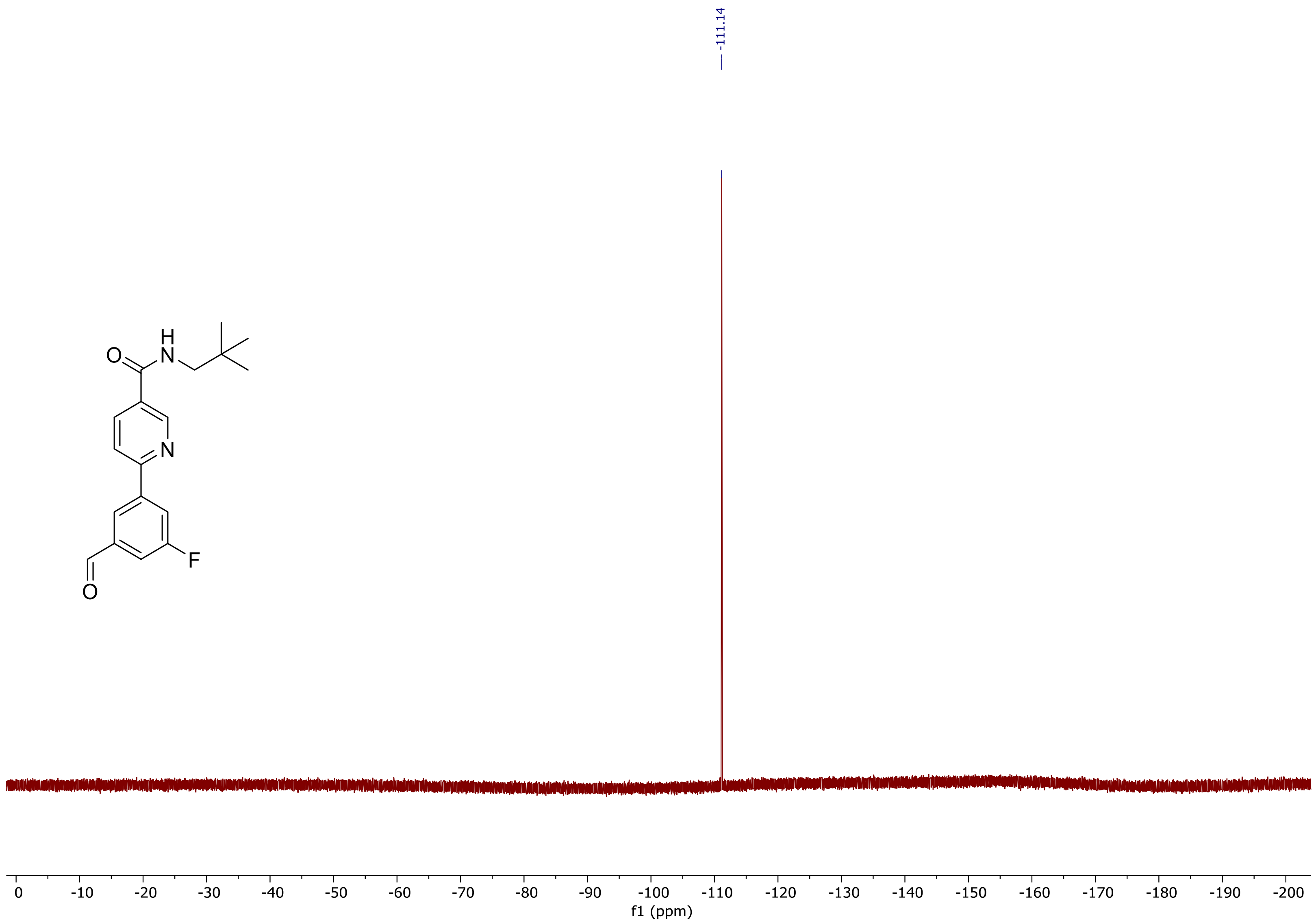

Supplementary Figure 92. ^{19}F NMR (376 MHz, $CDCl_3$) of 6-[3-(cyclopropylcarbamoyl)-5-fluorophenyl]-*N*-neopentylnicotinamide **7**.

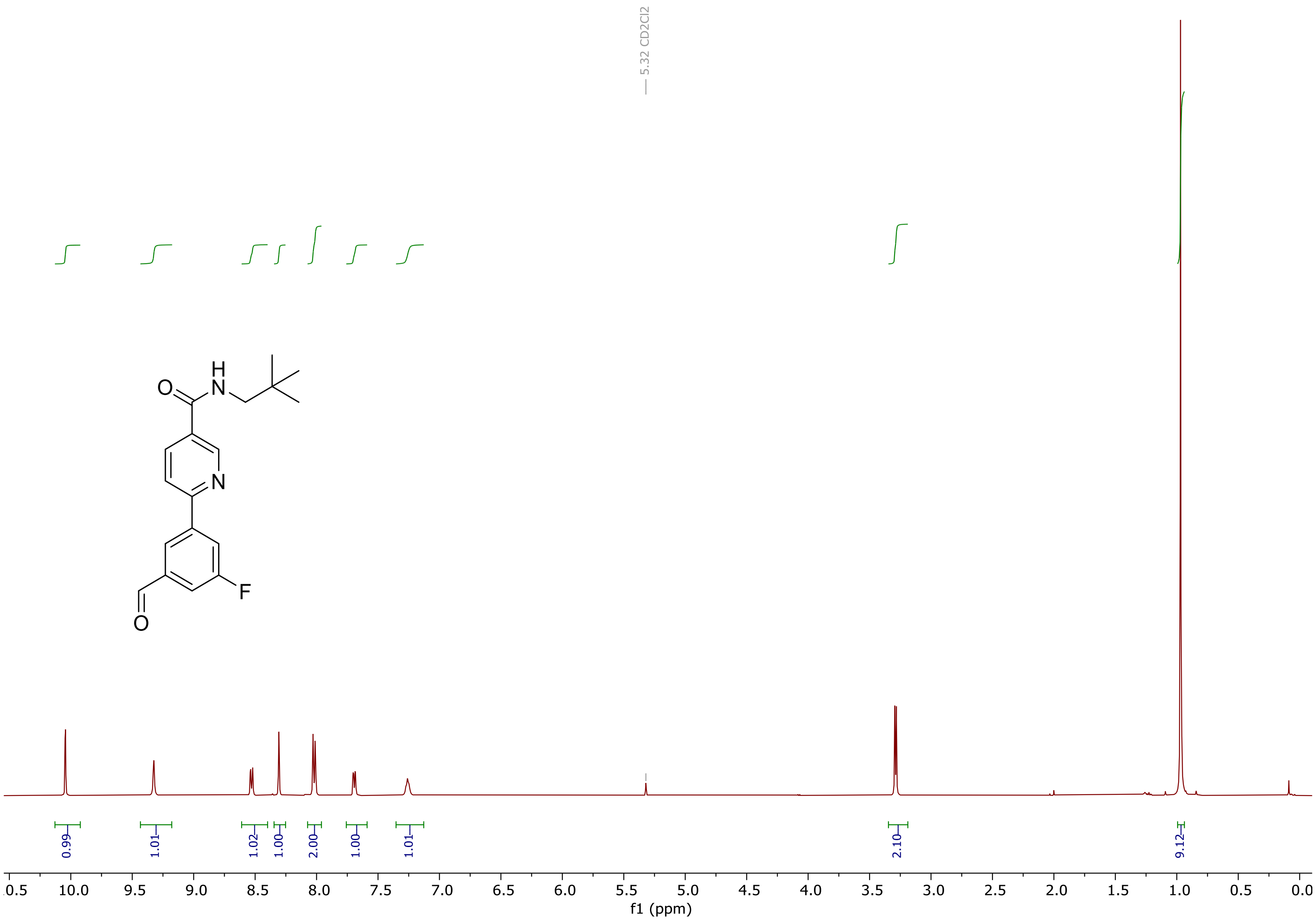

Supplementary Figure 93. ^{1}H NMR (500 MHz, CD_2Cl_2) of 6-(3-fluoro-5-formylphenyl)-*N*-neopentylnicotinamide.

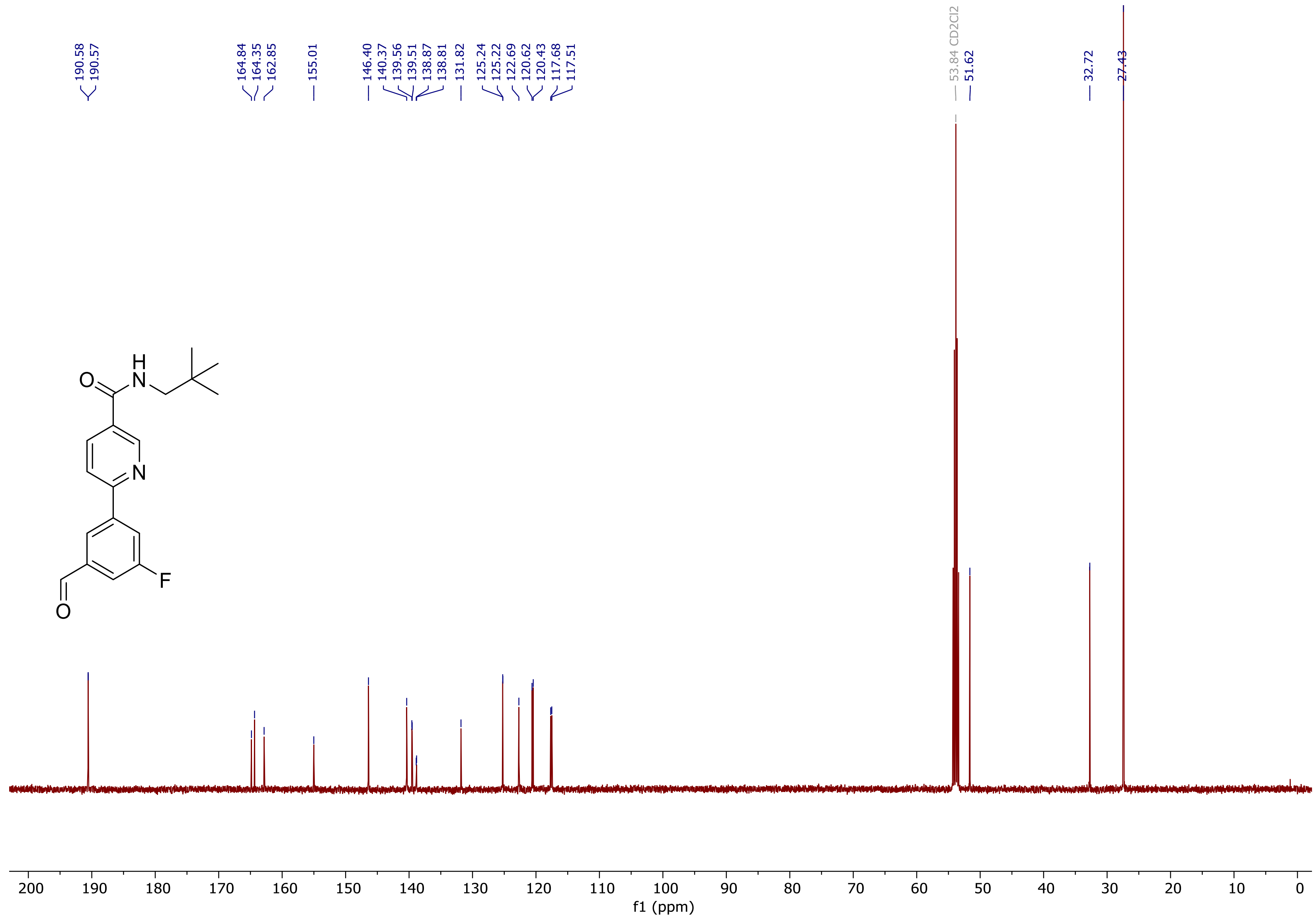

Supplementary Figure 94. ^{13}C NMR (126 MHz, CD_2Cl_2) of 6-(3-fluoro-5-formylphenyl)-*N*-neopentylnicotinamide.

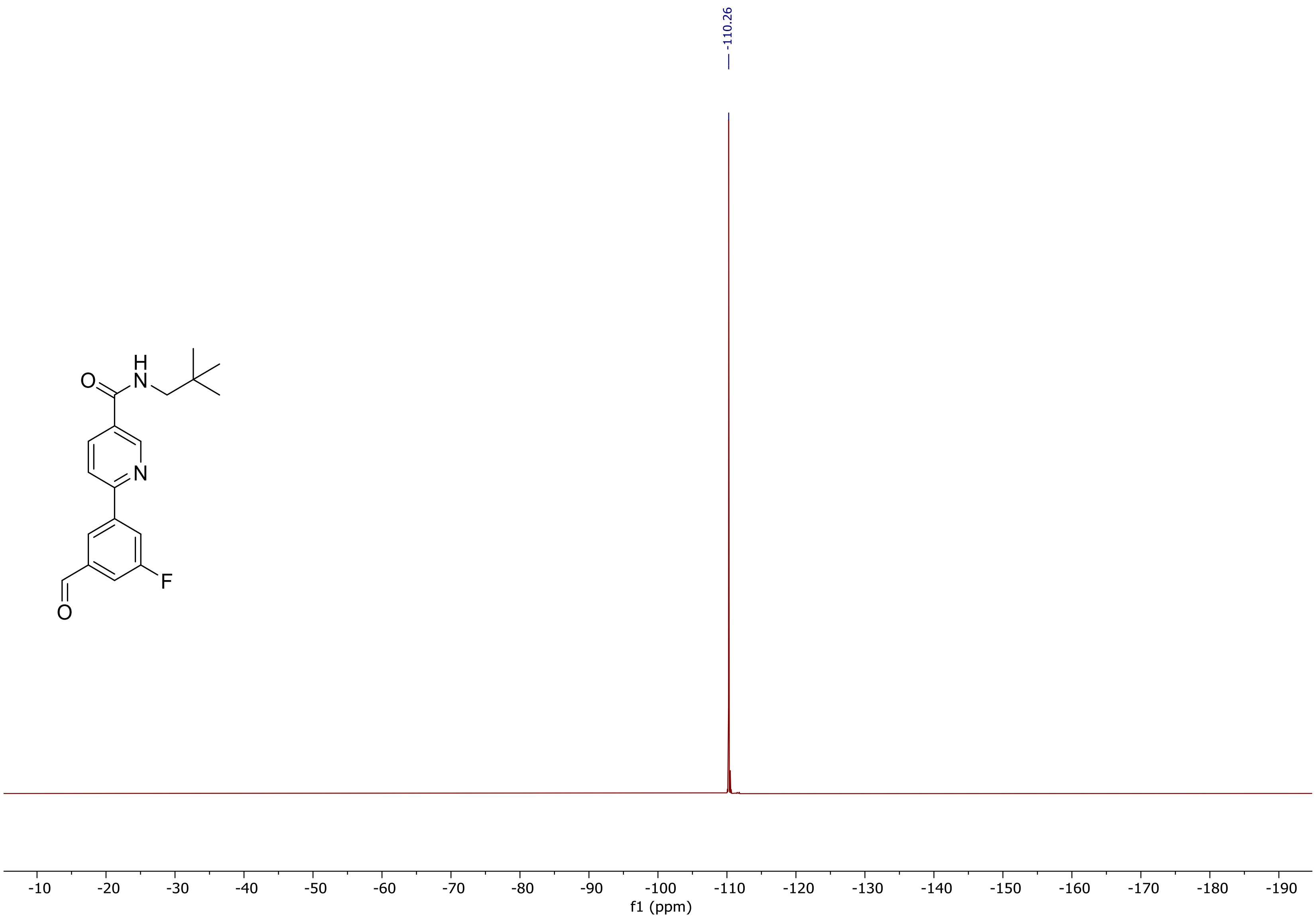

Supplementary Figure 95. ^{19}F NMR (471 MHz, CD_2Cl_2) of 6-(3-fluoro-5-formylphenyl)-*N*-neopentylnicotinamide.

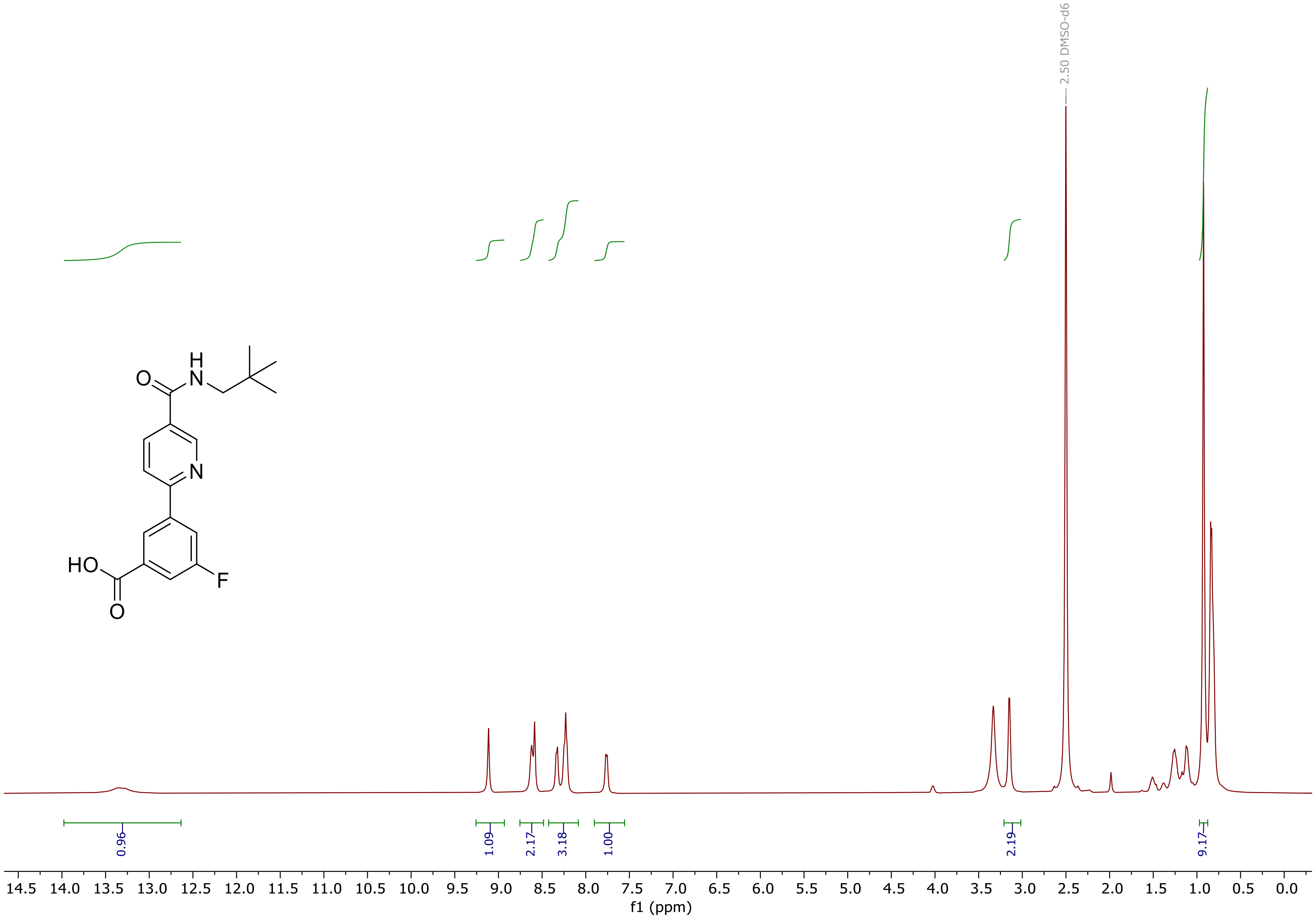

Supplementary Figure 96. ^{1}H NMR (500 MHz, $(CD_3)_2SO$) of 3-fluoro-5-[5-(neopentylcarbamoyl)pyridin-2-yl]benzoic acid.

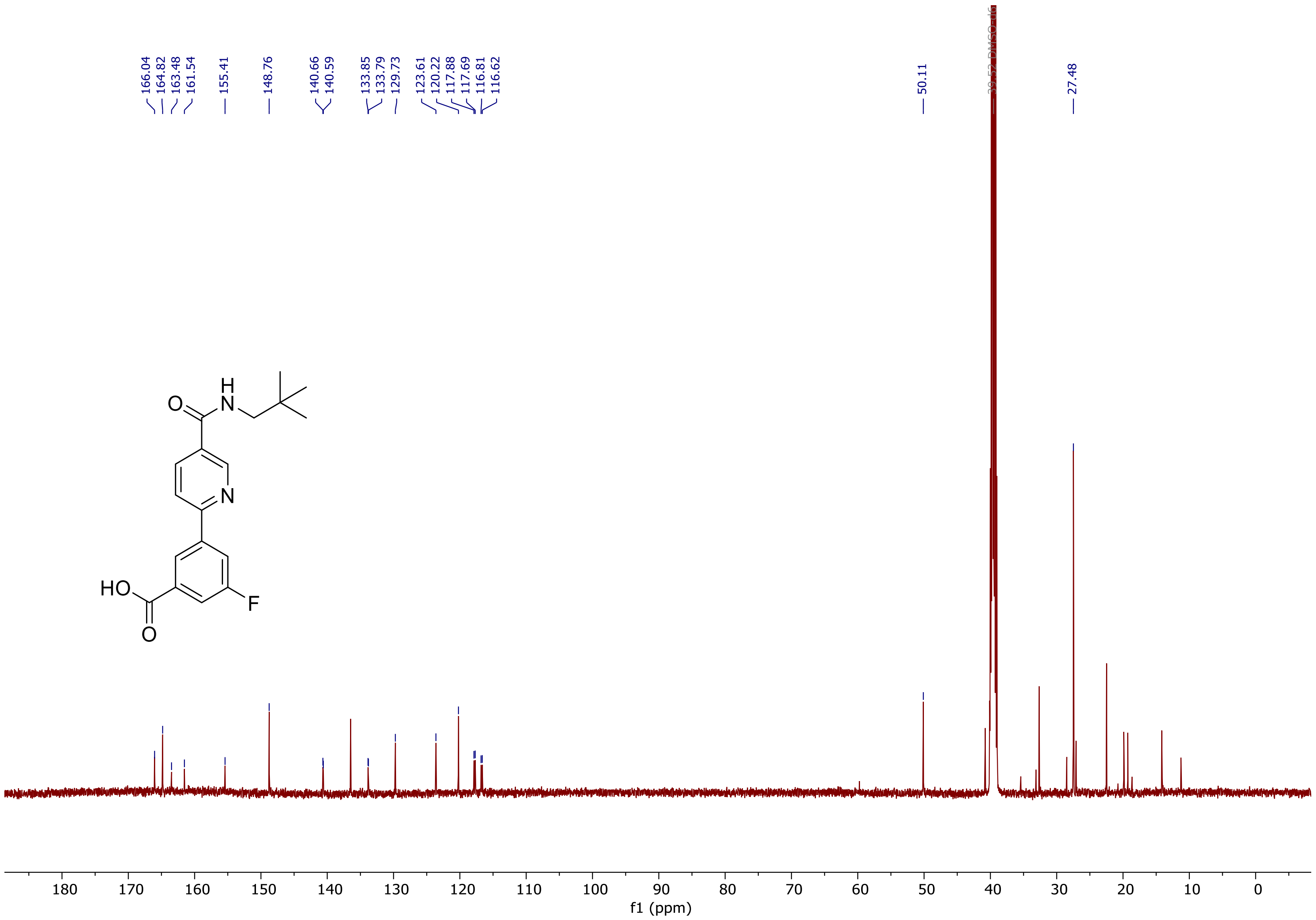

Supplementary Figure 97. ^{13}C NMR (126 MHz, $(CD_3)_2SO$) of 3-fluoro-5-[5-(neopentylcarbamoyl)pyridin-2-yl]benzoic acid.

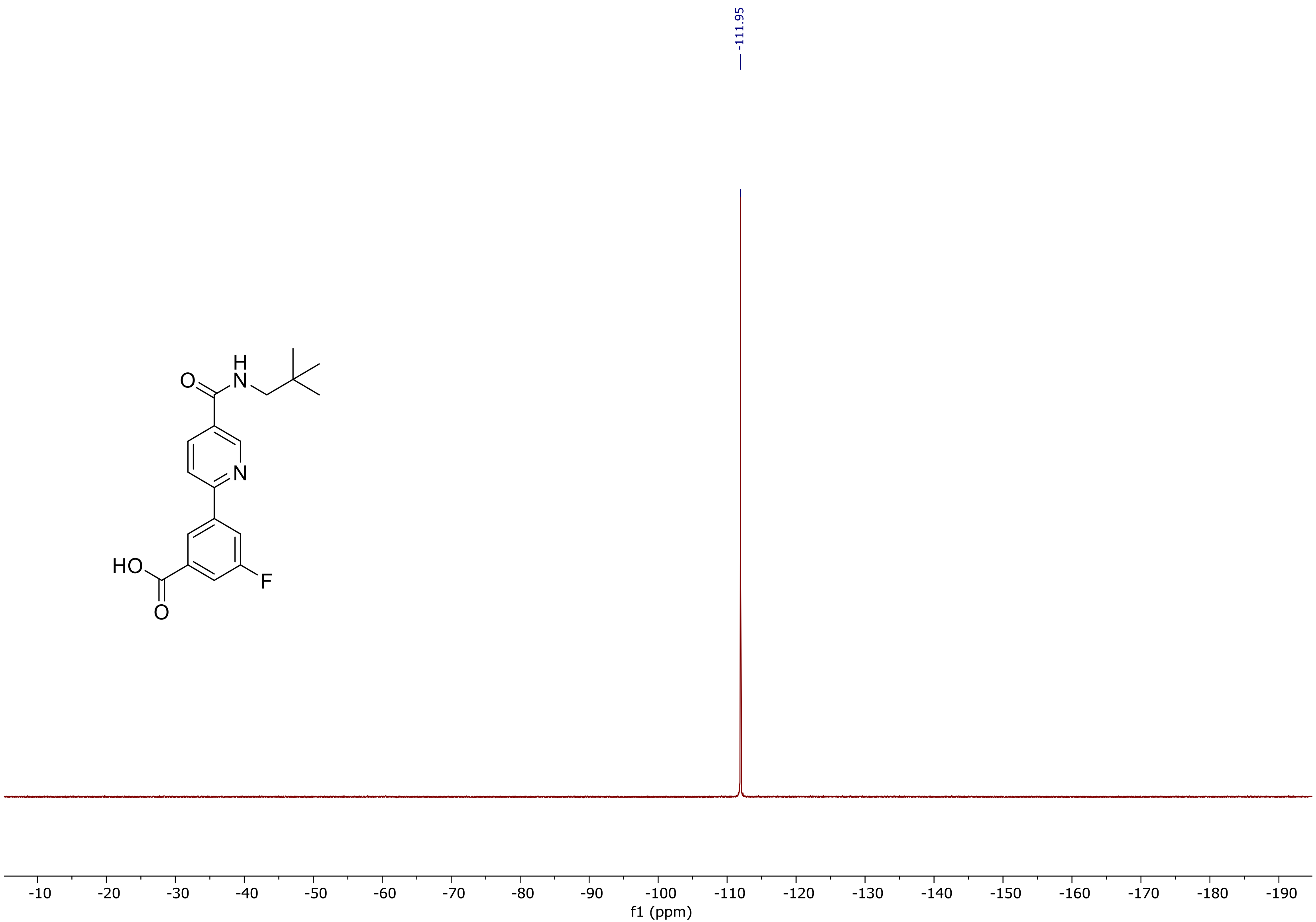

Supplementary Figure 98. ^{19}F NMR (126 MHz, $(CD_3)_2SO$) of 3-fluoro-5-[5-(neopentylcarbamoyl)pyridin-2-yl]benzoic acid.

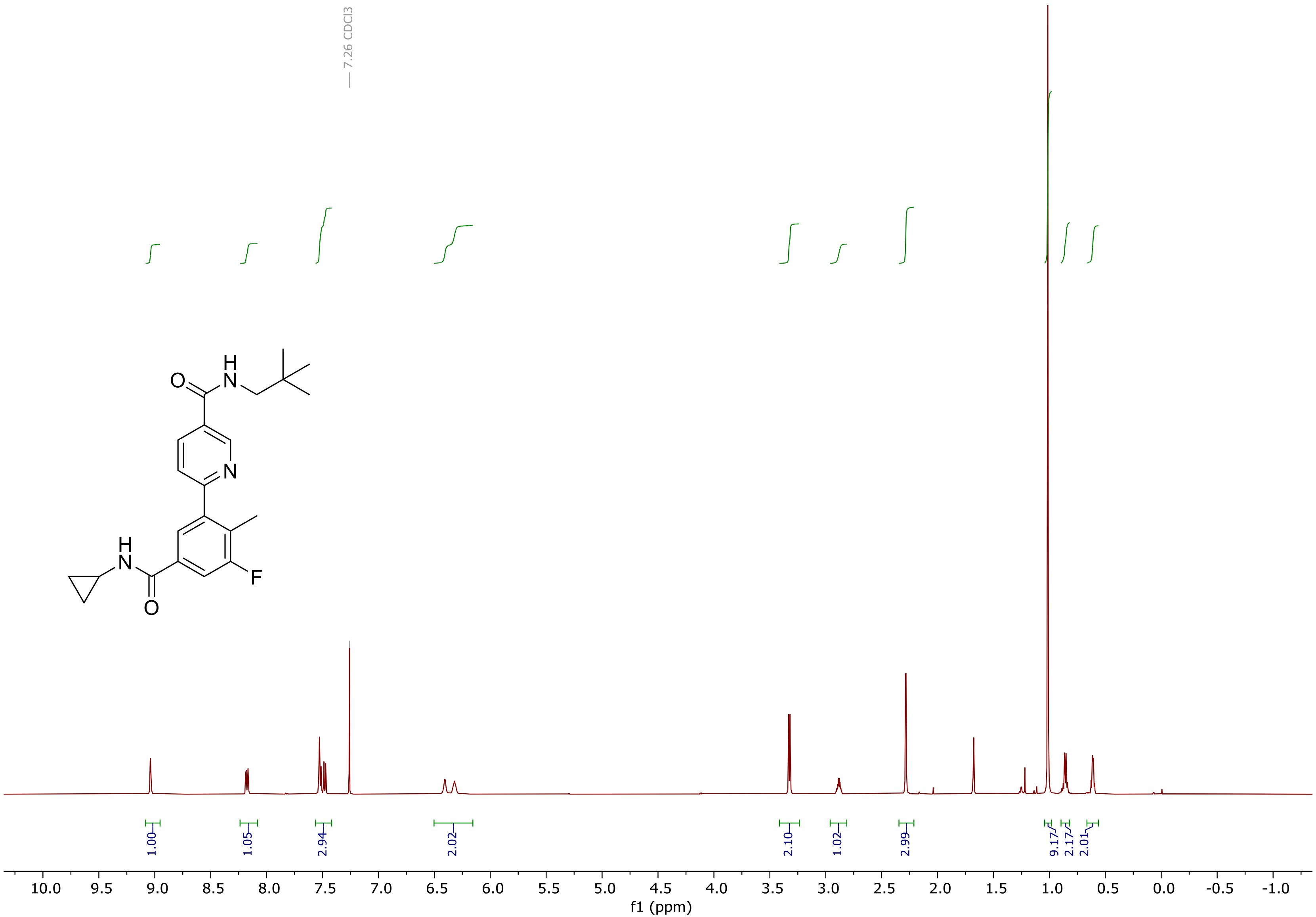

Supplementary Figure 99. ^{1}H NMR (500 MHz, $CDCl_3$) of Losmapimod **8**.

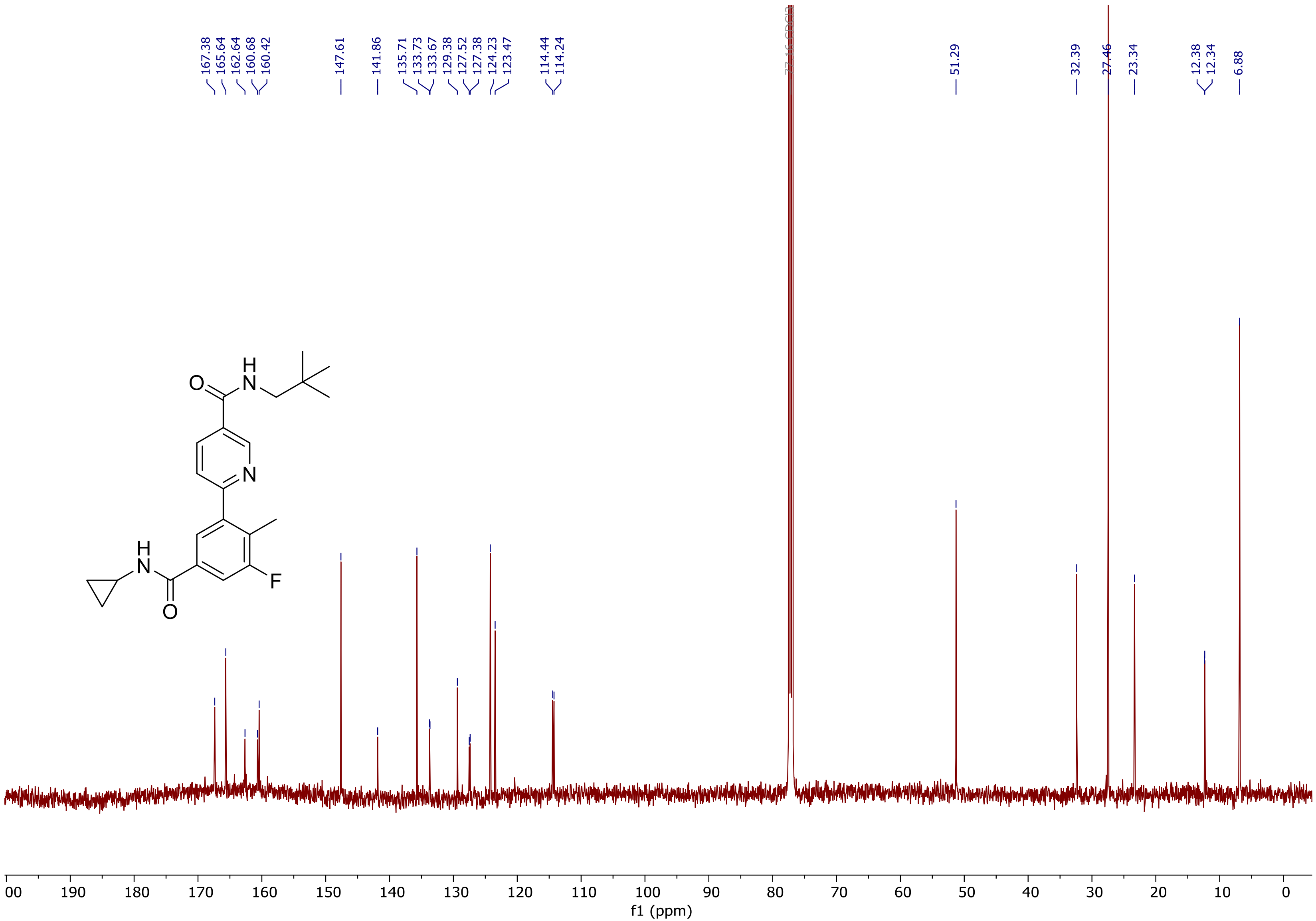

Supplementary Figure 100. ^{13}C NMR (126 MHz, $CDCl_3$) of Losmapimod **8**.

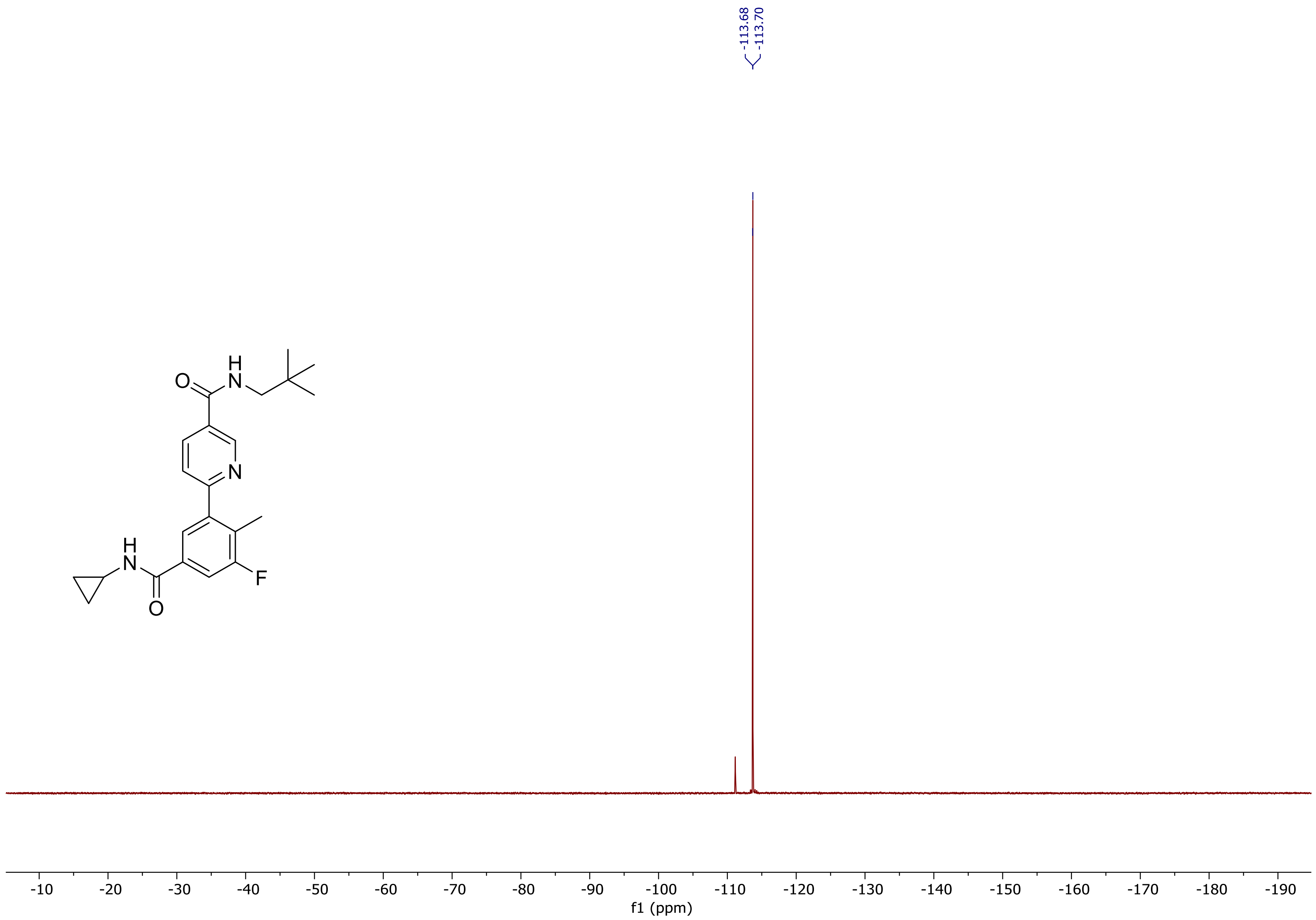

Supplementary Figure 101. ^{19}F NMR (471 MHz, $CDCl_3$) of Losmapimod **8**.

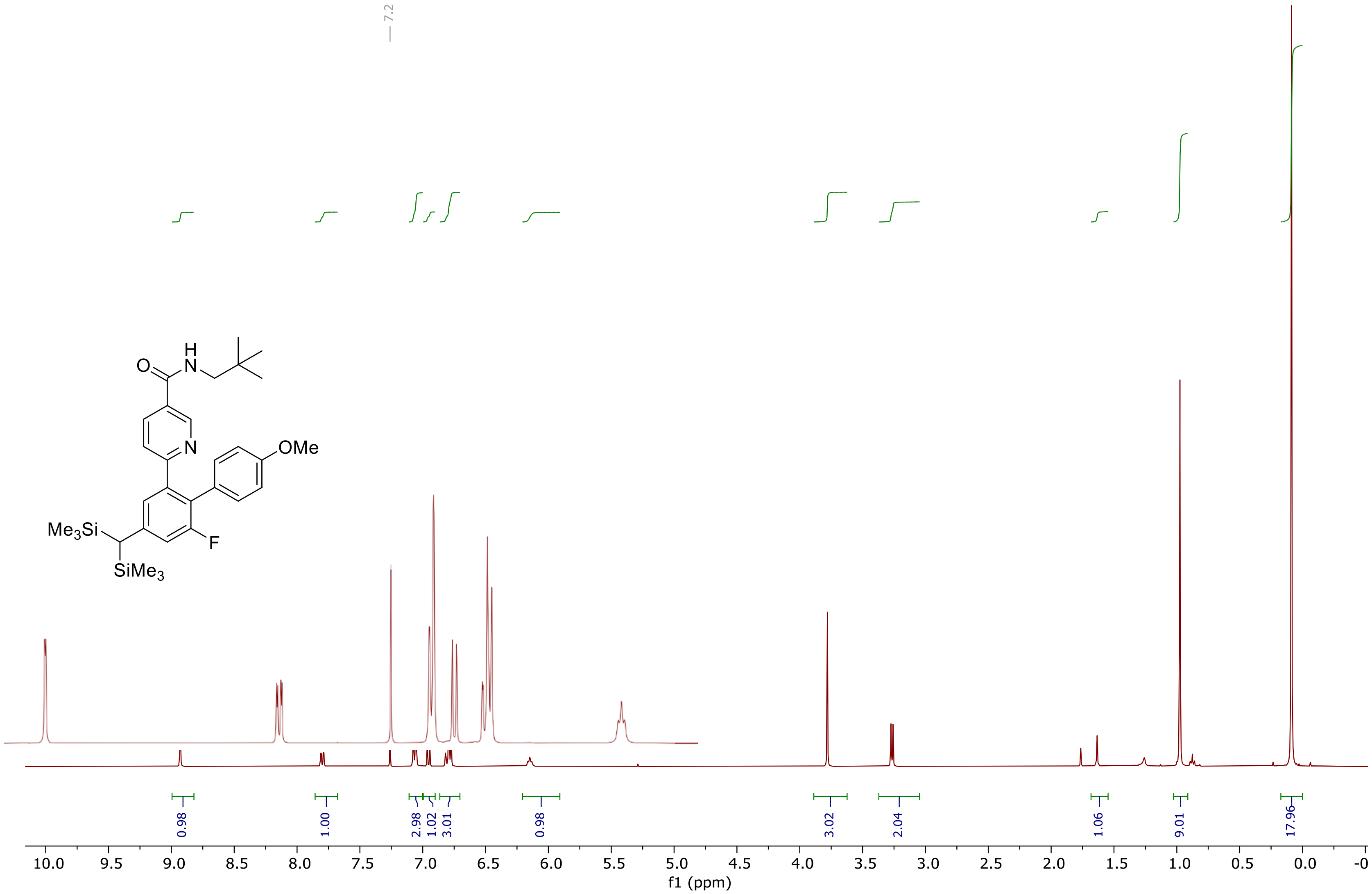

Supplementary Figure 102. ^{1}H NMR (400 MHz, $CDCl_3$) of 6-{4-[bis(trimethylsilyl)methyl]-6-fluoro-4'-methoxy-[1,1'-biphenyl]-2-yl}-*N*-neopentylnicotinamide.

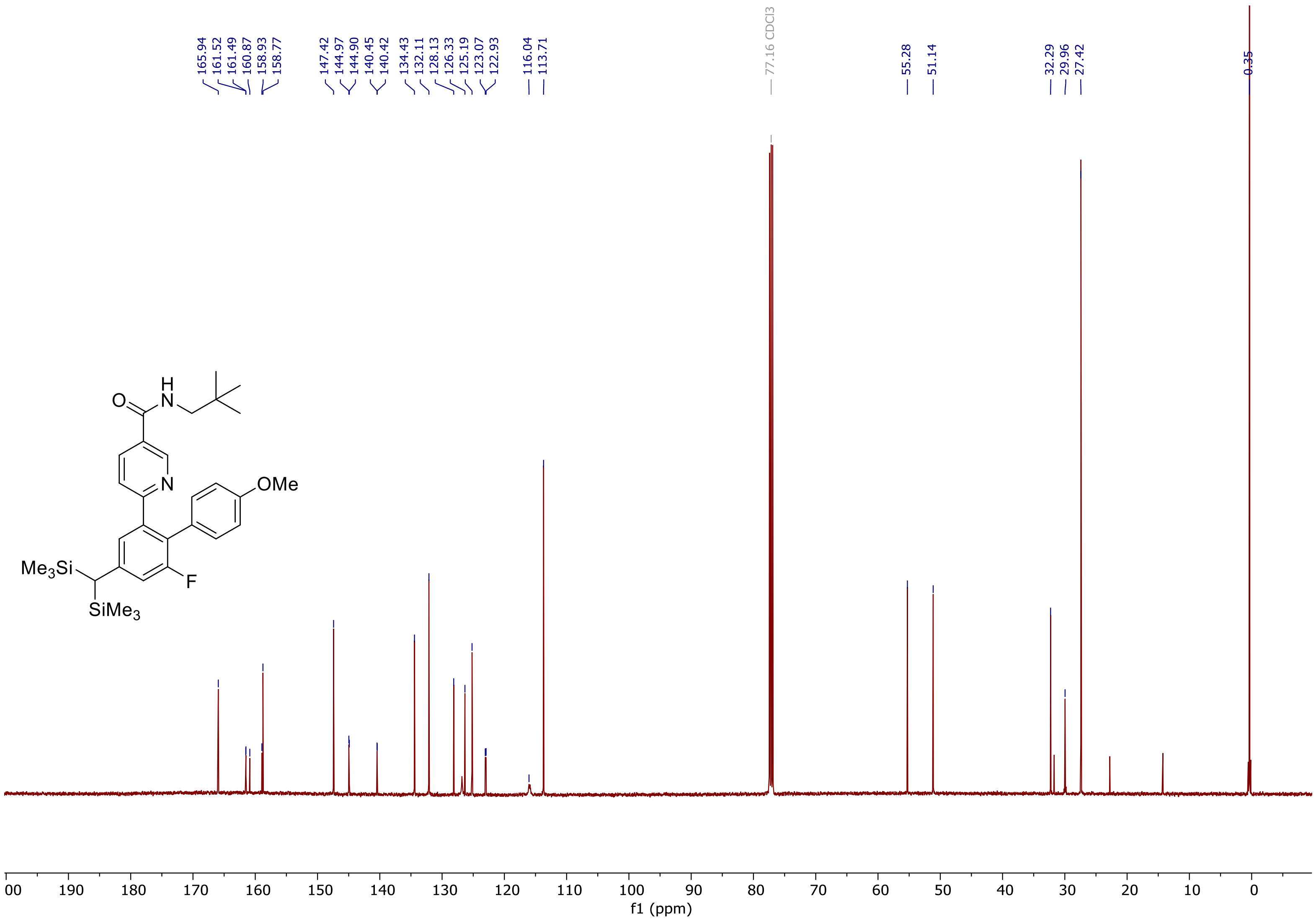

Supplementary Figure 103. ^{13}C NMR (101 MHz, $CDCl_3$) of 6-{4-[bis(trimethylsilyl)methyl]-6-fluoro-4'-methoxy-[1,1'-biphenyl]-2-yl}-*N*-neopentylnicotinamide.

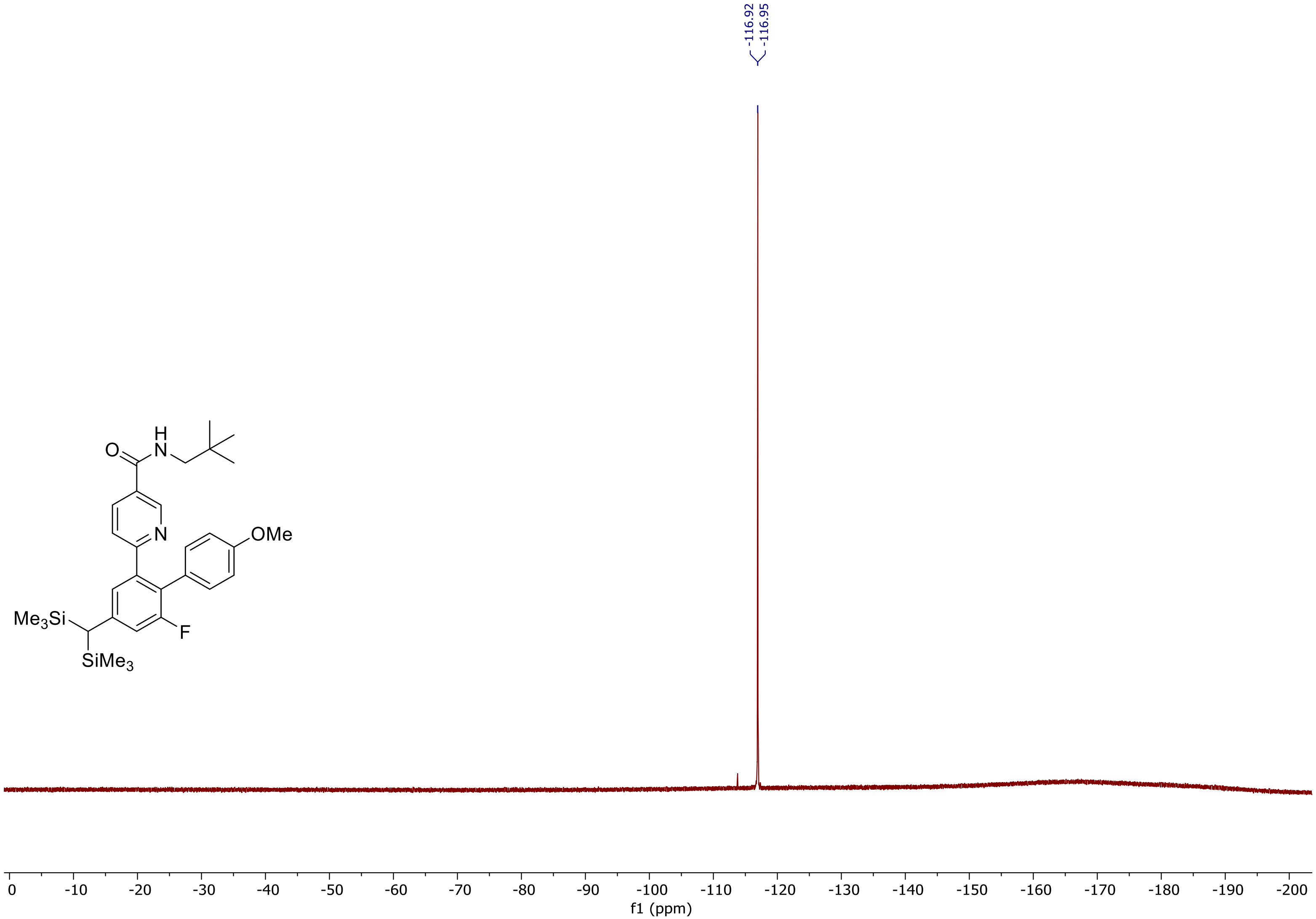

Supplementary Figure 104. ^{19}F NMR (376 MHz, $CDCl_3$) of 6-{4-[bis(trimethylsilyl)methyl]-6-fluoro-4'-methoxy-[1,1'-biphenyl]-2-yl}-*N*-neopentylnicotinamide.

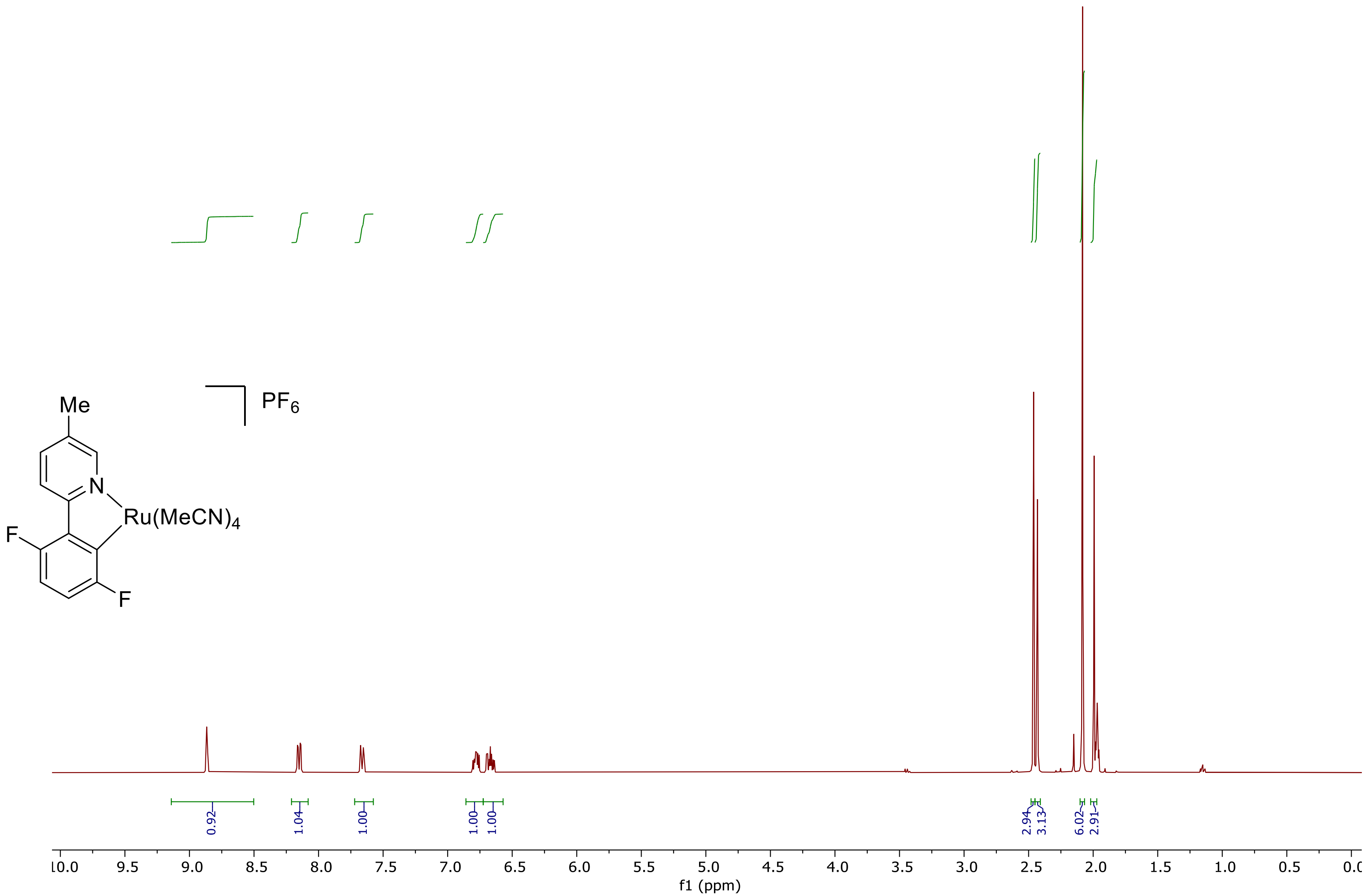

Supplementary Figure 105. ^{1}H NMR (400 MHz, d_3-CD_3CN) of 2-(2,5-difluorophenyl)-5-methylpyridine ruthenium(II) tetraacetonitrile hexafluorophosphate **31**.

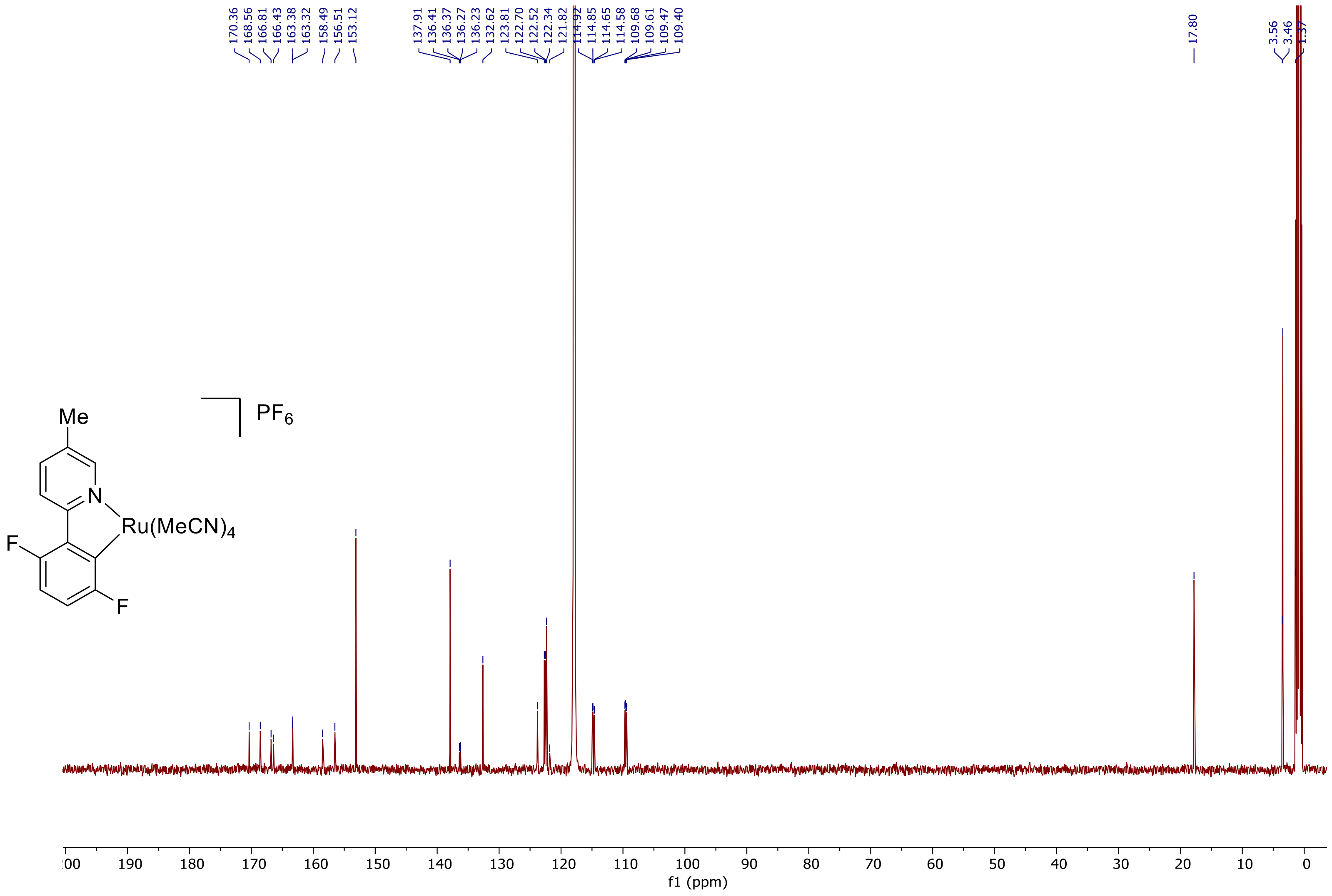

Supplementary Figure 106. ^{13}C NMR (126 MHz, d_3-CD_3CN) of 2-(2,5-difluorophenyl)-5-methylpyridine ruthenium(II) tetraacetonitrile hexafluorophosphate **31**.

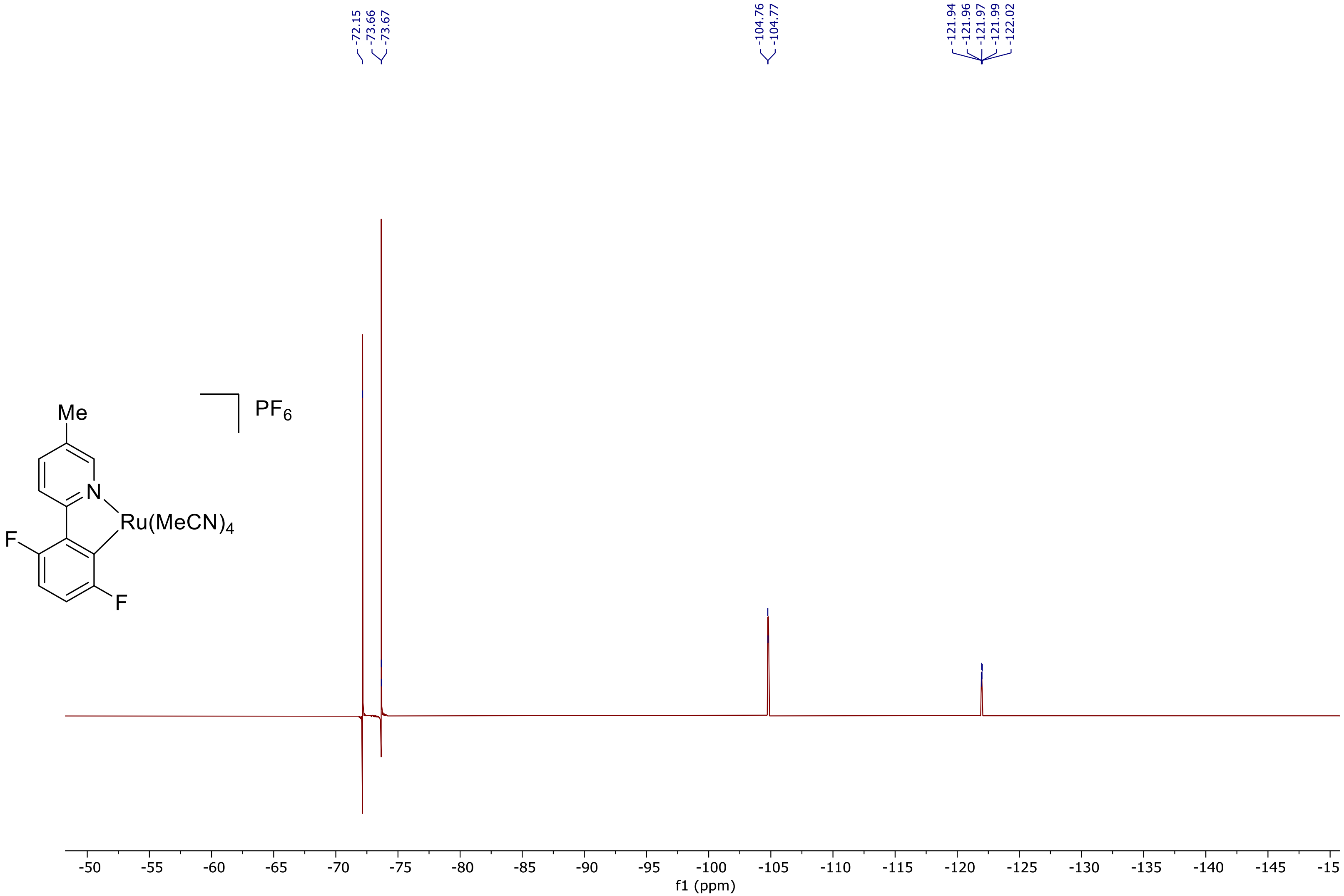

Supplementary Figure 107. ^{19}F NMR (471 MHz, d_3-CD_3CN) of 2-(2,5-difluorophenyl)-5-methylpyridine ruthenium(II) tetraacetonitrile hexafluorophosphate **31**.

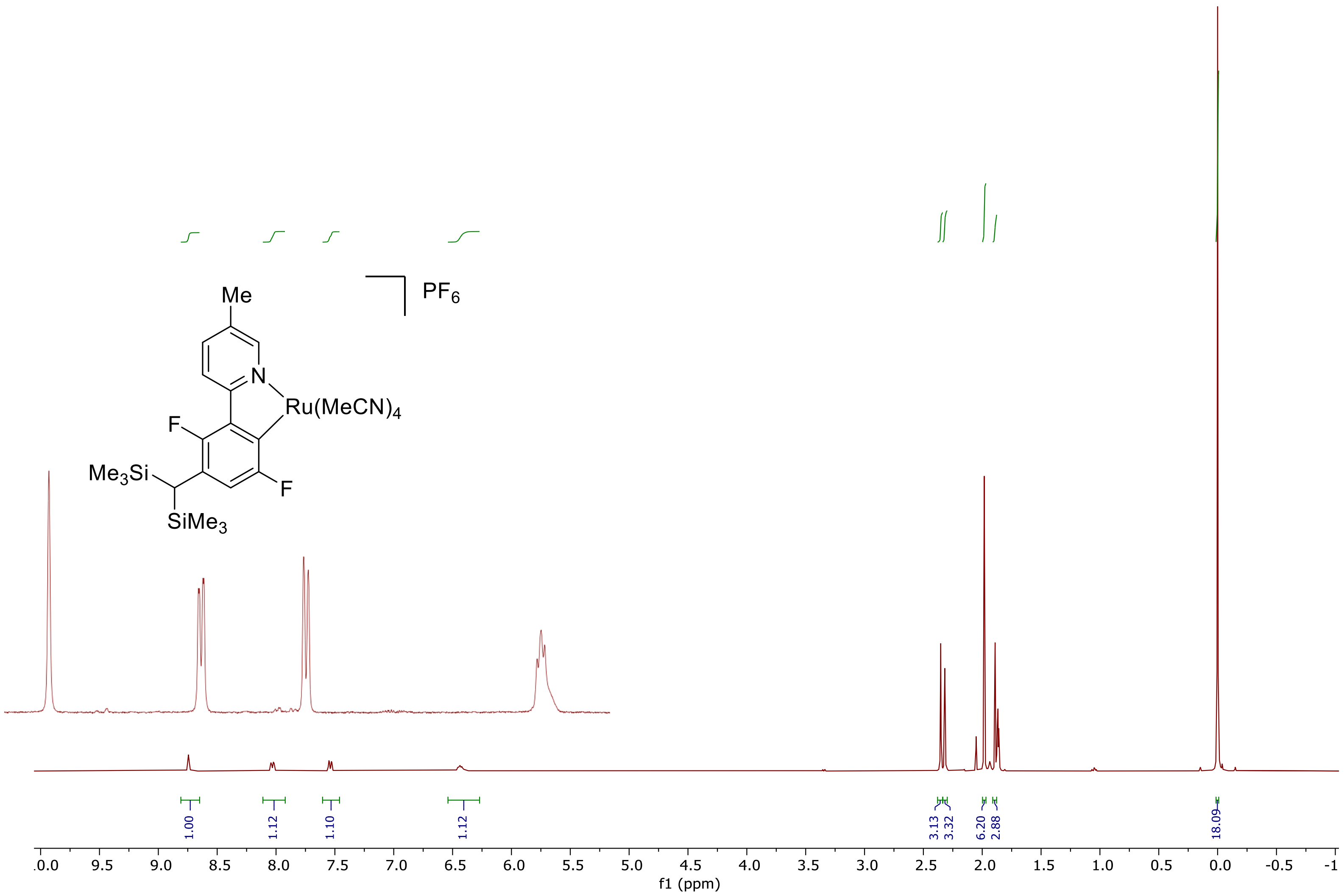

Supplementary Figure 108. ^{1}H NMR (400 MHz, d_3-CD_3CN) of 2-{3-[bis(trimethylsilyl)methyl]-2,5-difluorophenyl}-5-methylpyridine ruthenium(II) tetraacetonitrile hexafluorophosphate **36**.

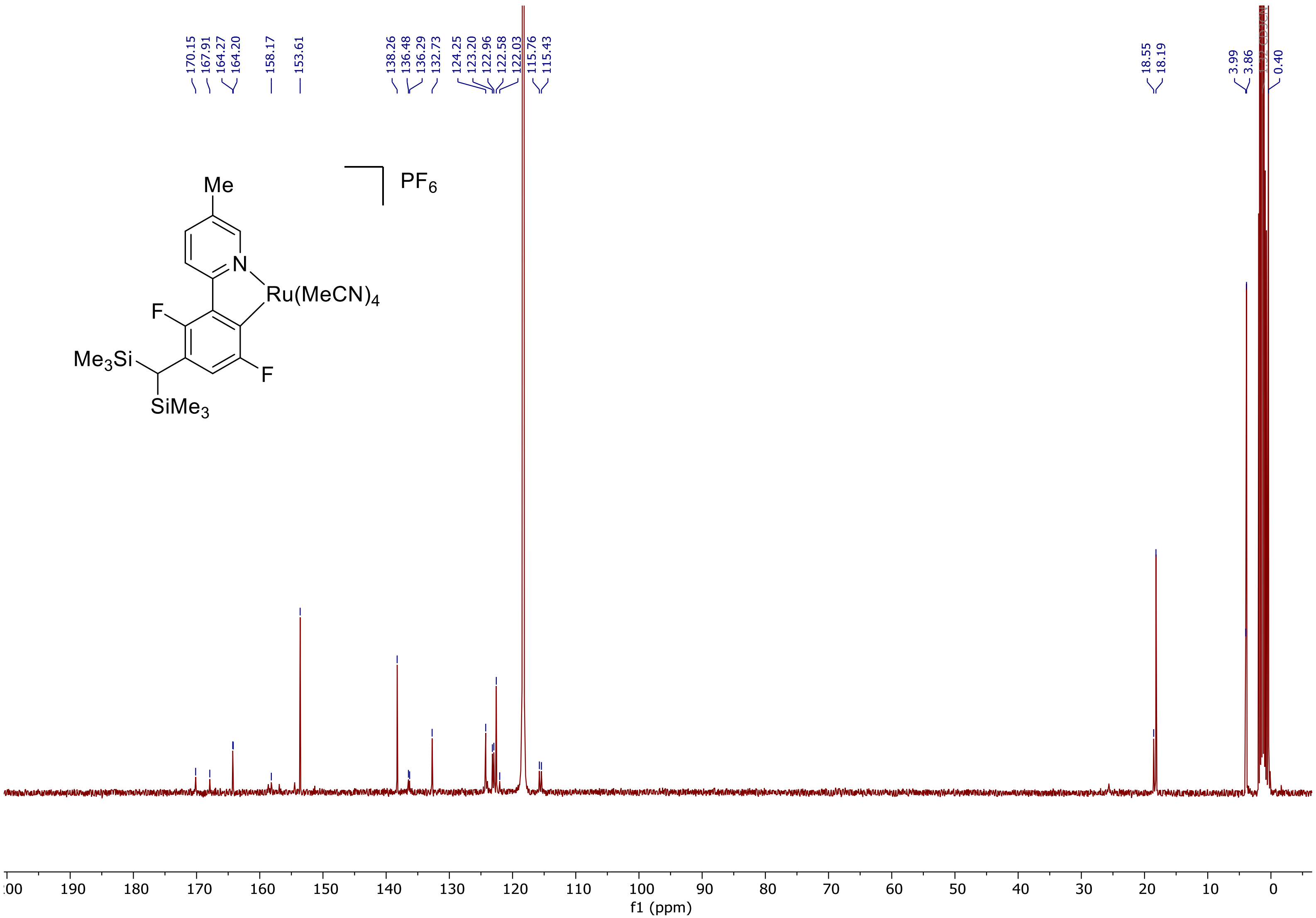

Supplementary Figure 109. ^{13}C NMR (101 MHz, d_3-CD_3CN) of 2-{3-[bis(trimethylsilyl)methyl]-2,5-difluorophenyl}-5-methylpyridine ruthenium(II) tetraacetonitrile hexafluorophosphate **36**.

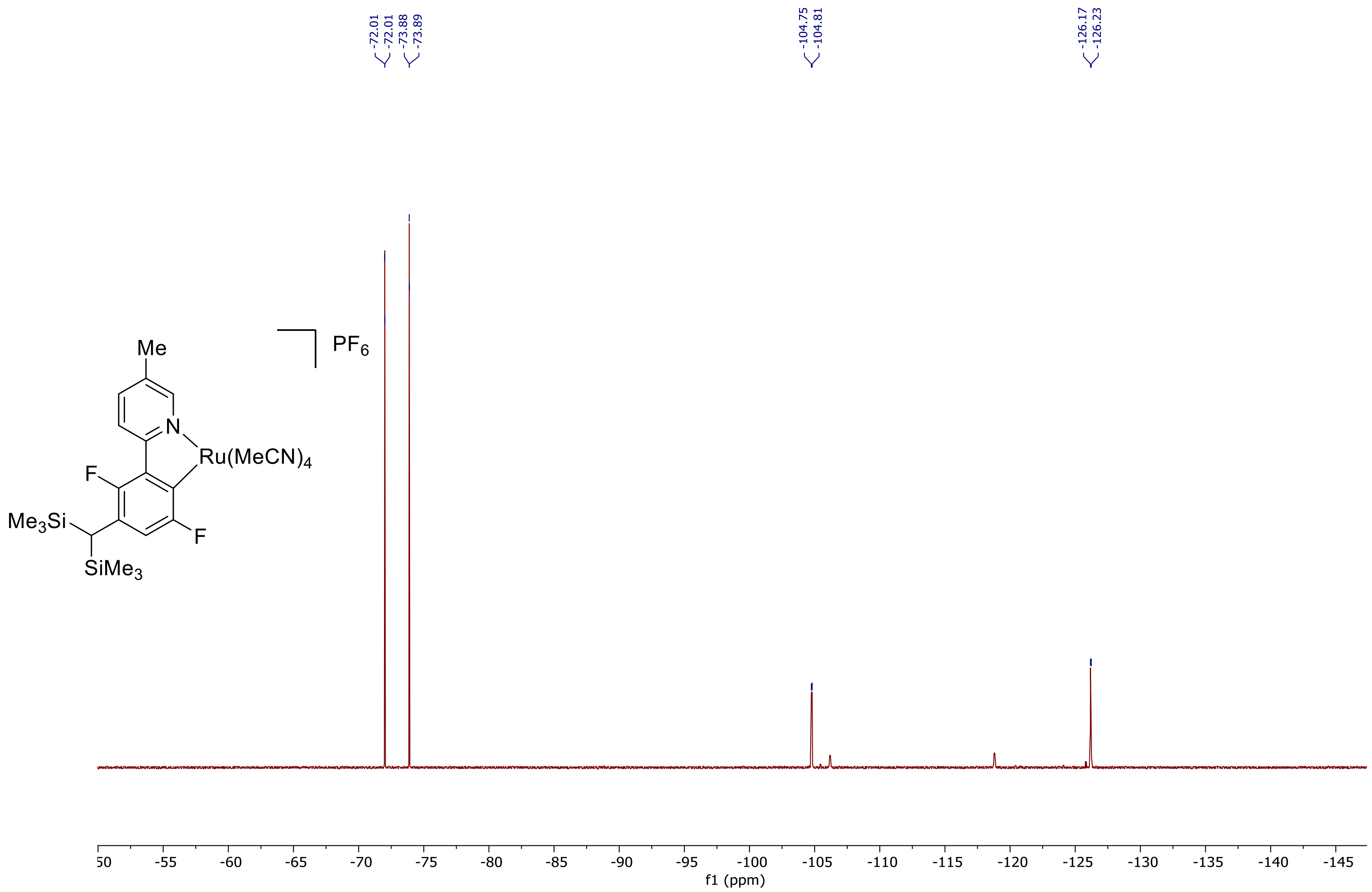

Supplementary Figure 110. ^{19}F NMR (471 MHz, d_3-CD_3CN) of 2-{3-[bis(trimethylsilyl)methyl]-2,5-difluorophenyl}-5-methylpyridine ruthenium(II) tetraacetonitrile hexafluorophosphate **36**.

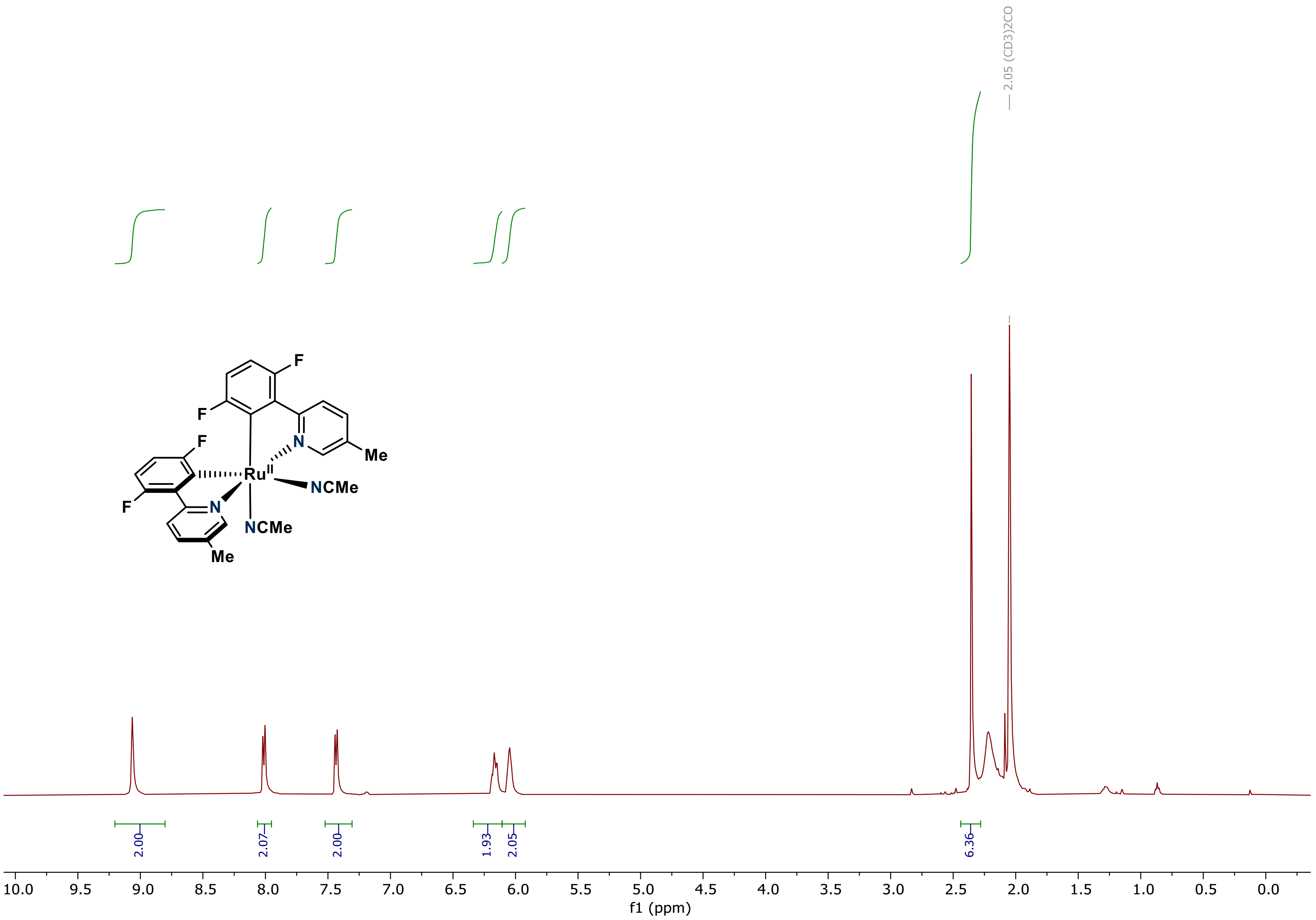

Supplementary Figure 111. ^1H NMR (500 MHz, d_6-$(CD_3)_2CO$) of bis[2-(2,5-difluorophenyl)-5-methylpyridine] ruthenium(II) bisacetonitrile **40**.

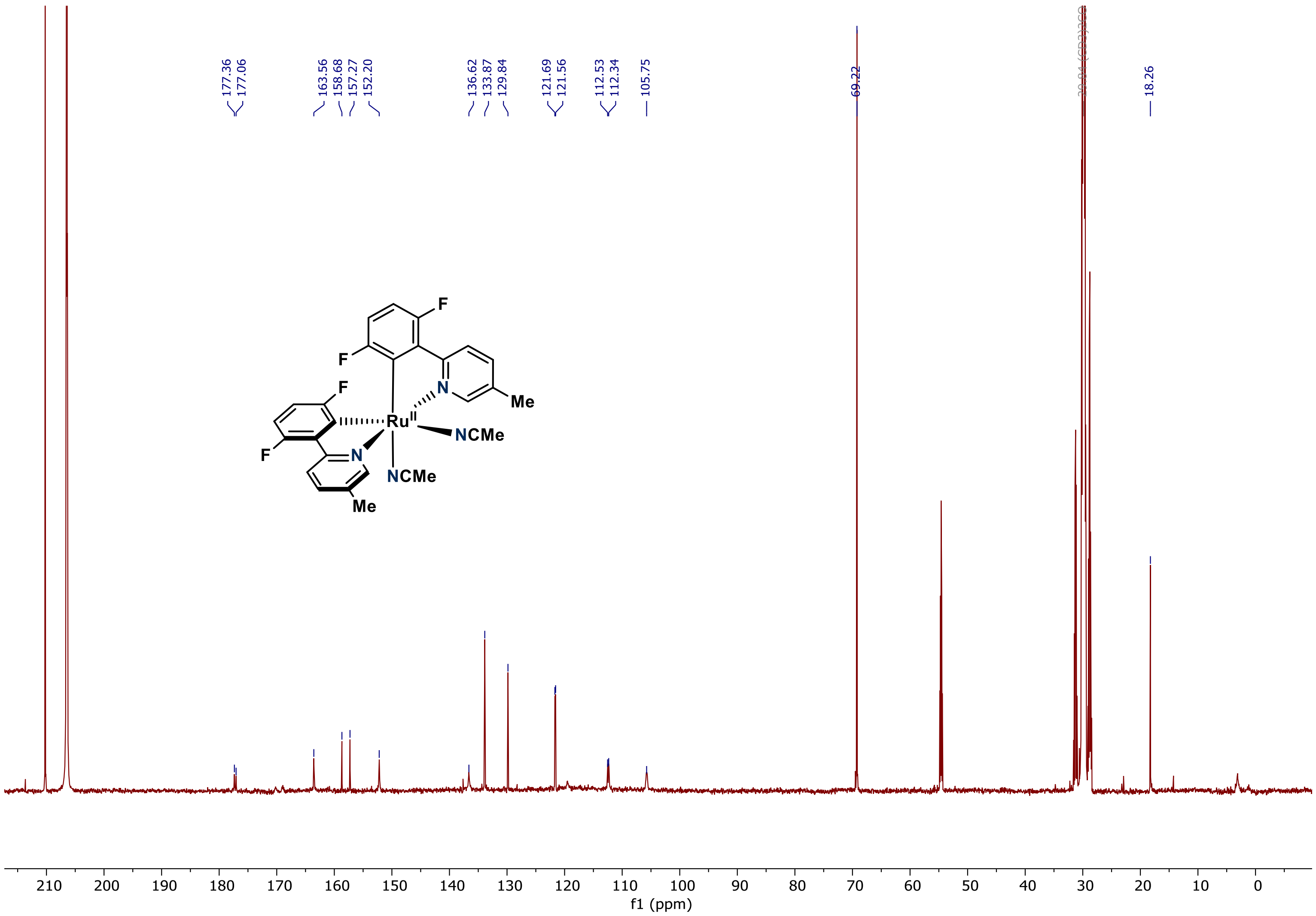

Supplementary Figure 112. ^{13}C NMR (171 MHz, d_6-$(CD_3)_2CO$) of bis[2-(2,5-difluorophenyl)-5-methylpyridine] ruthenium(II) bisacetonitrile **40**. (acetone/MeCN exchange observed)

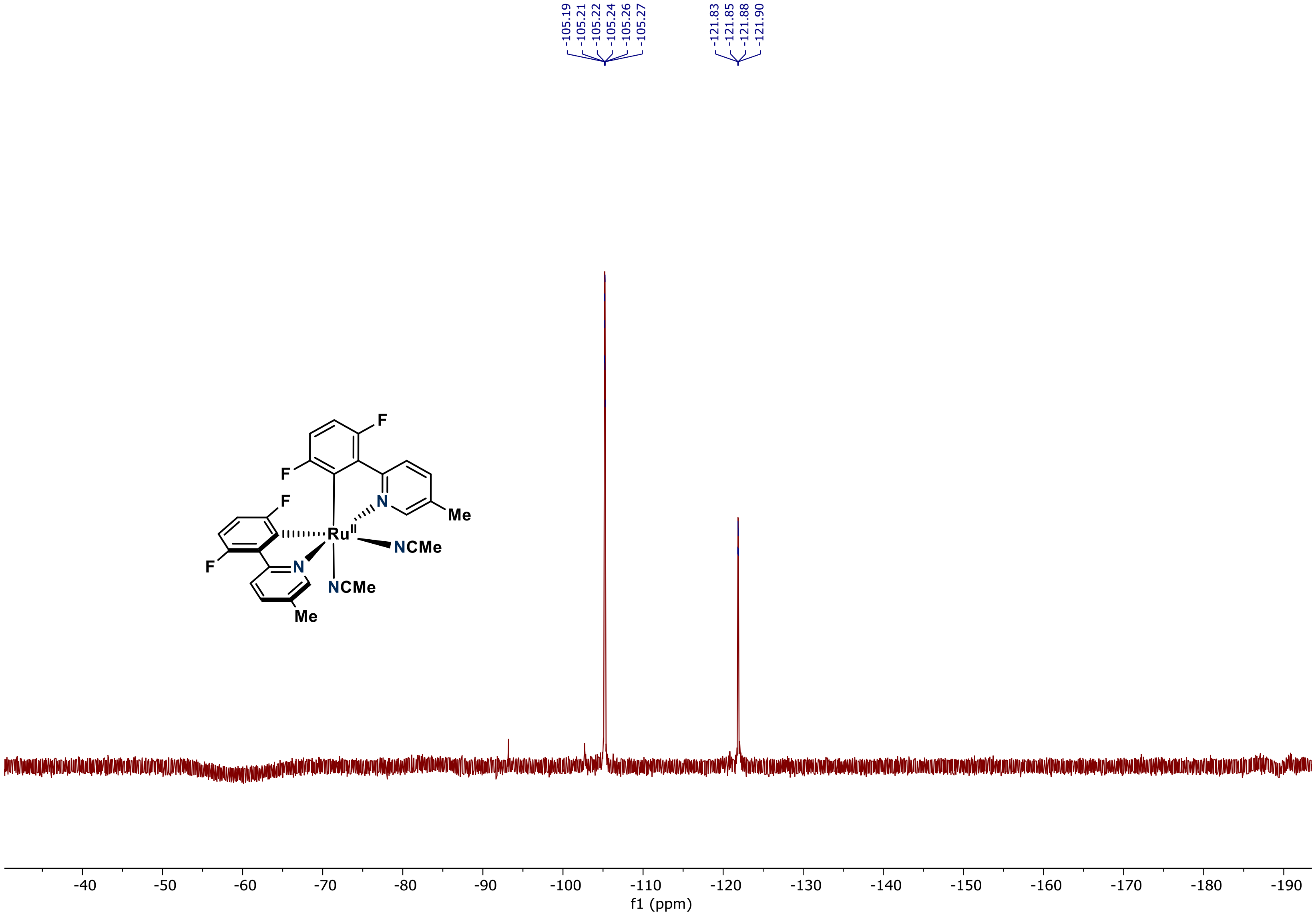

Supplementary Figure 113. ^{19}F NMR (471 MHz, d_6-$(CD_3)_2CO$) of bis[2-(2,5-difluorophenyl)-5-methylpyridine] ruthenium(II) bisacetonitrile **40**.

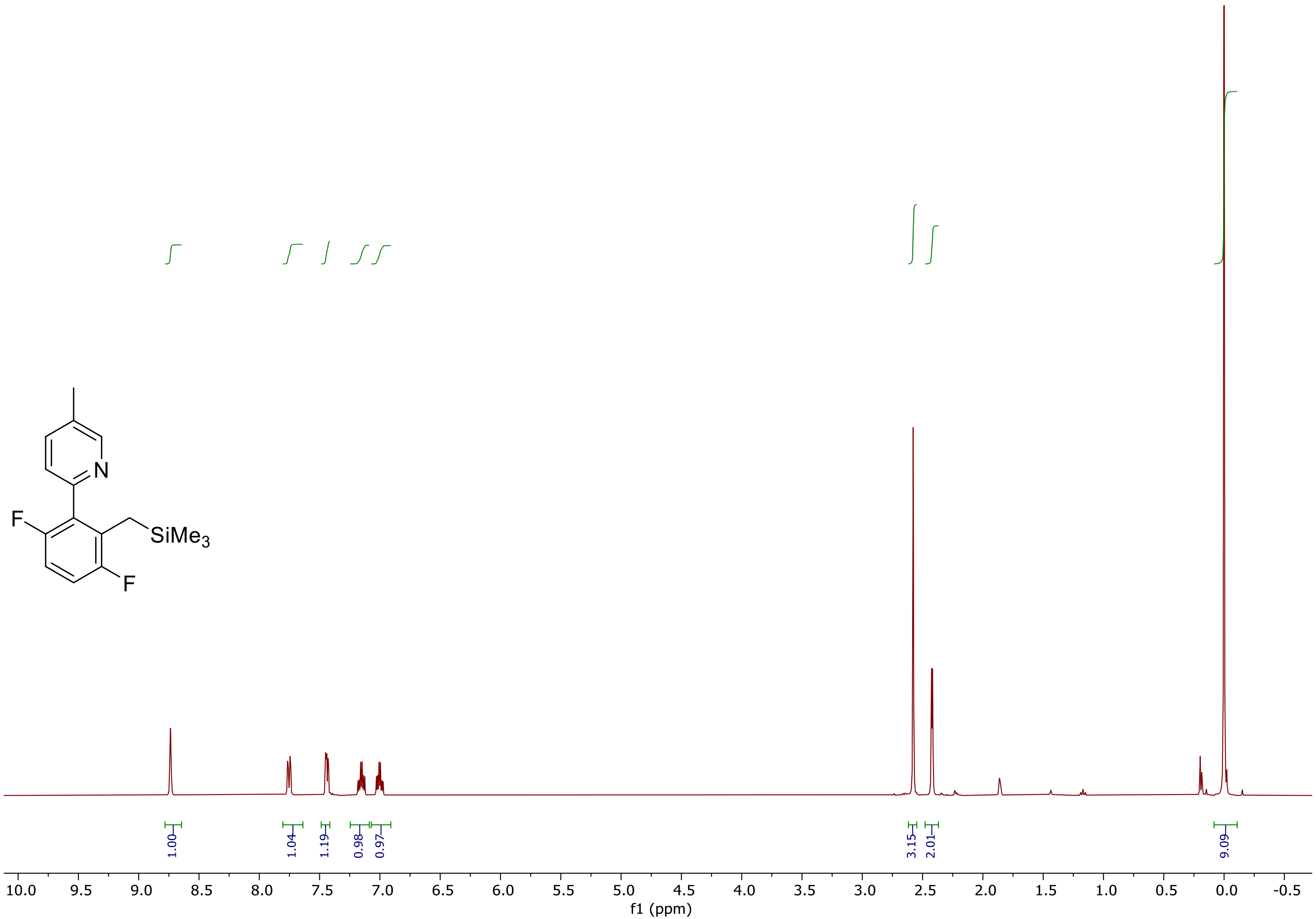

Supplementary Figure 114. ^{1}H NMR (400 MHz, $CDCl_3$) of 2-(3,6-difluoro-2-((trimethylsilyl)methyl)phenyl)-5-methylpyridine **4j**.

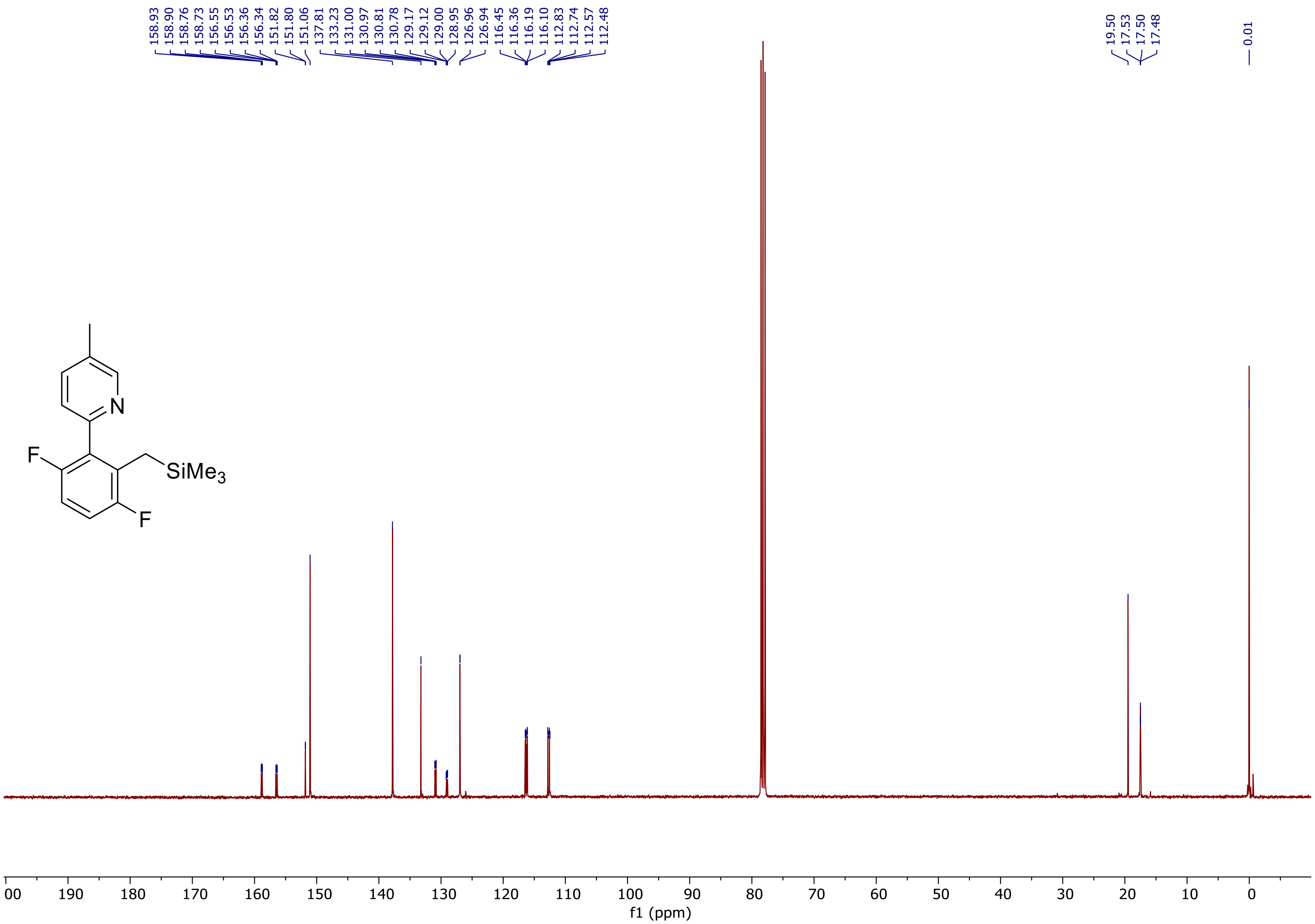

Supplementary Figure 115. ^{13}C NMR (101 MHz, $CDCl_3$) of 2-(3,6-difluoro-2-((trimethylsilyl)methyl)phenyl)-5-methylpyridine **4j**.

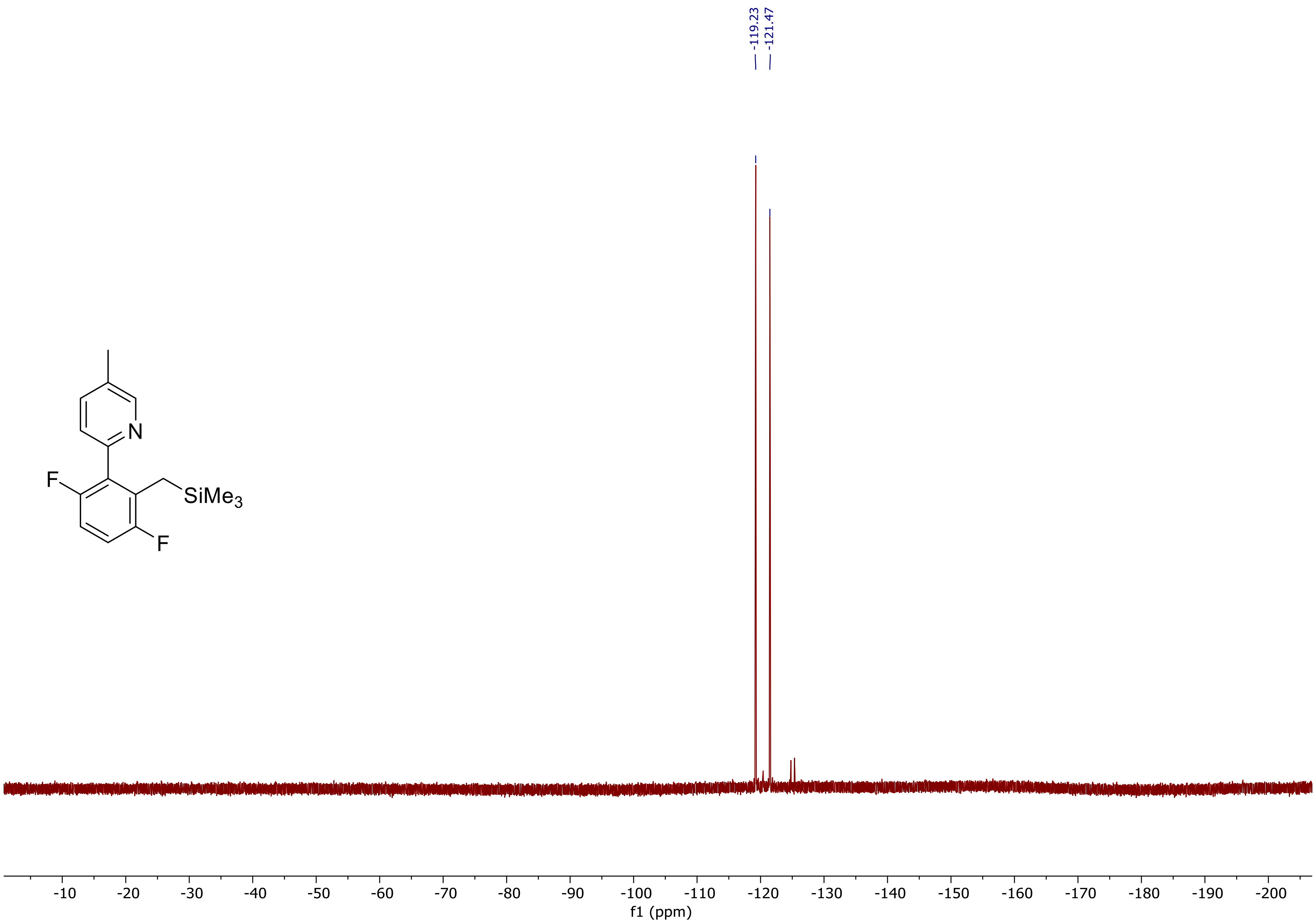

Supplementary Figure 116. ^{19}F NMR (376 MHz, $CDCl_3$) of 2-(3,6-difluoro-2-((trimethylsilyl)methyl)phenyl)-5-methylpyridine **4**.

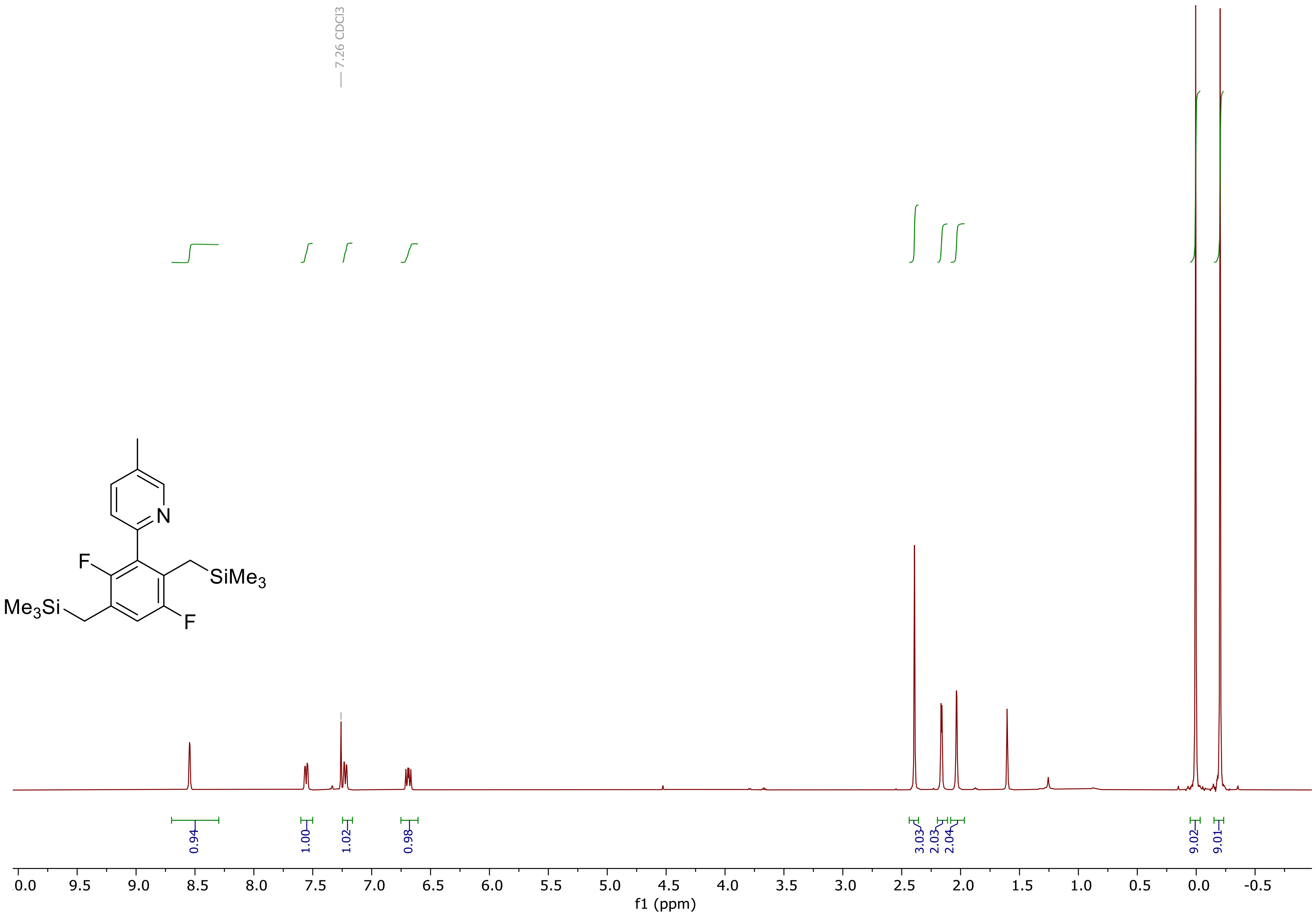

Supplementary Figure 117. ^1H NMR (400 MHz, $CDCl_3$) of 2-(2,5-difluoro-3,6-bis((trimethylsilyl)methyl)phenyl)-5-methylpyridine.

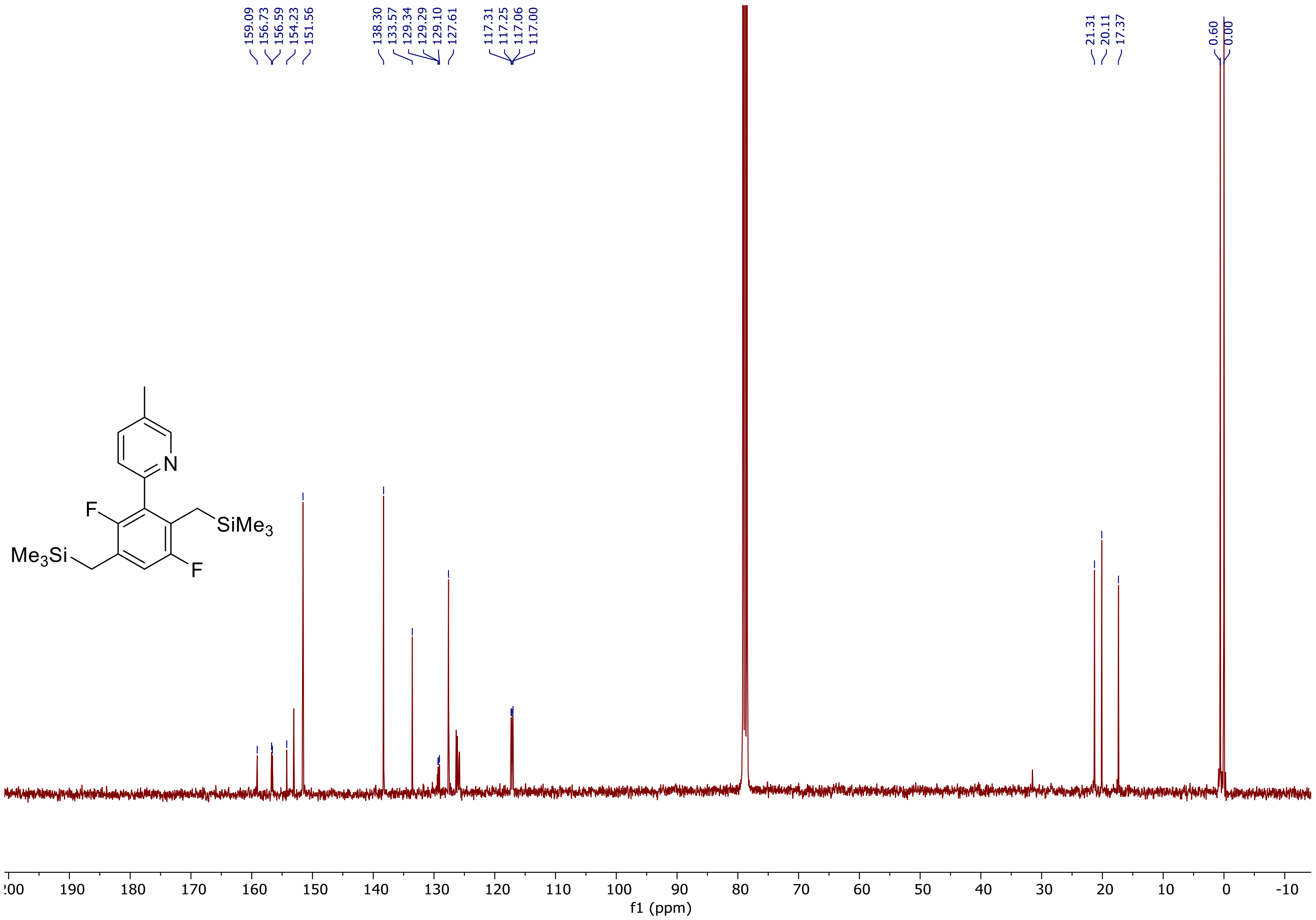

Supplementary Figure 118. ^{13}C NMR (101 MHz, $CDCl_3$) of 2-(2,5-difluoro-3,6-bis((trimethylsilyl)methyl)phenyl)-5-methylpyridine.

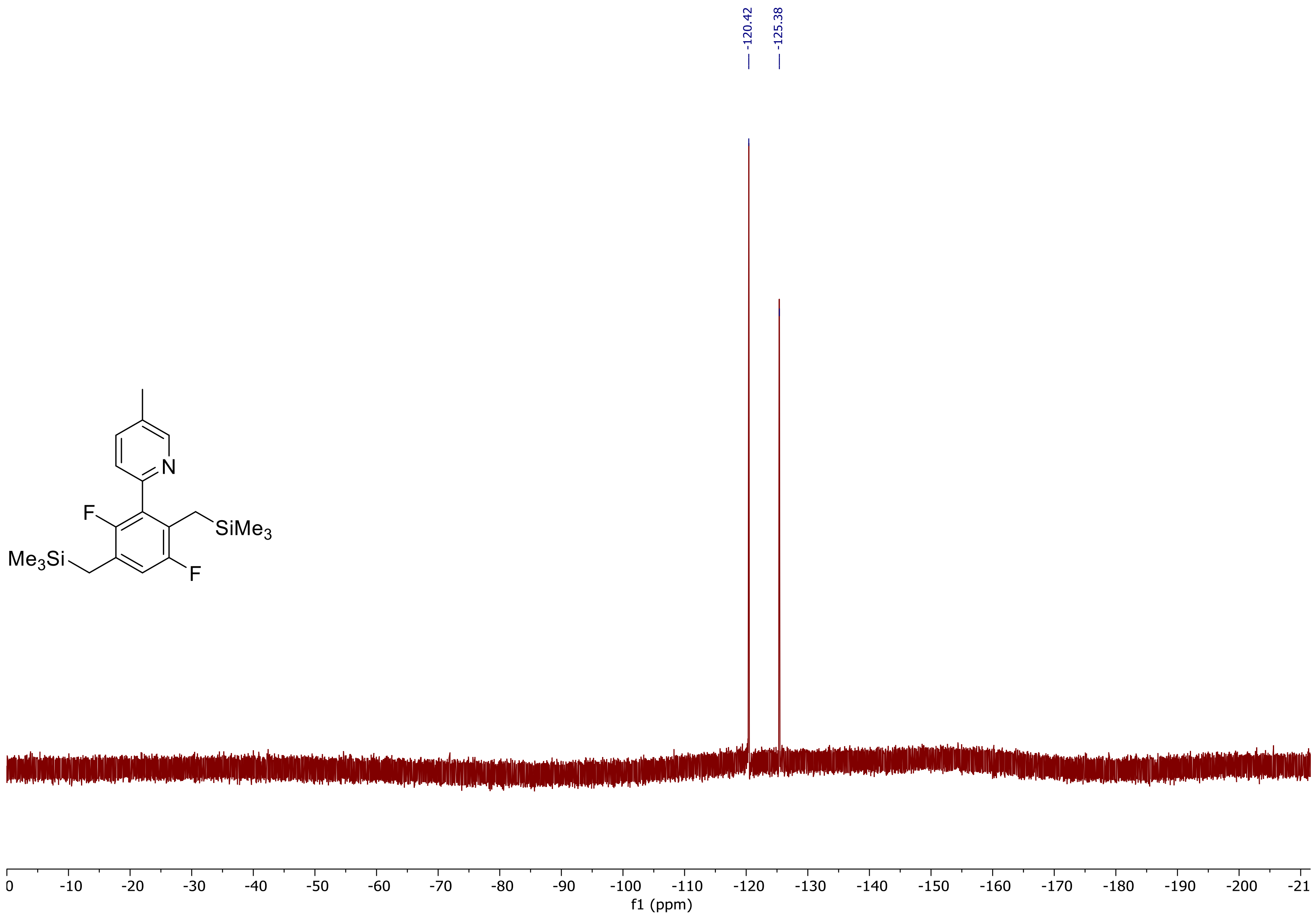

Supplementary Figure 119. ^{19}F NMR (376 MHz, $CDCl_3$) of 2-(2,5-difluoro-3,6-bis((trimethylsilyl)methyl)phenyl)-5-methylpyridine.

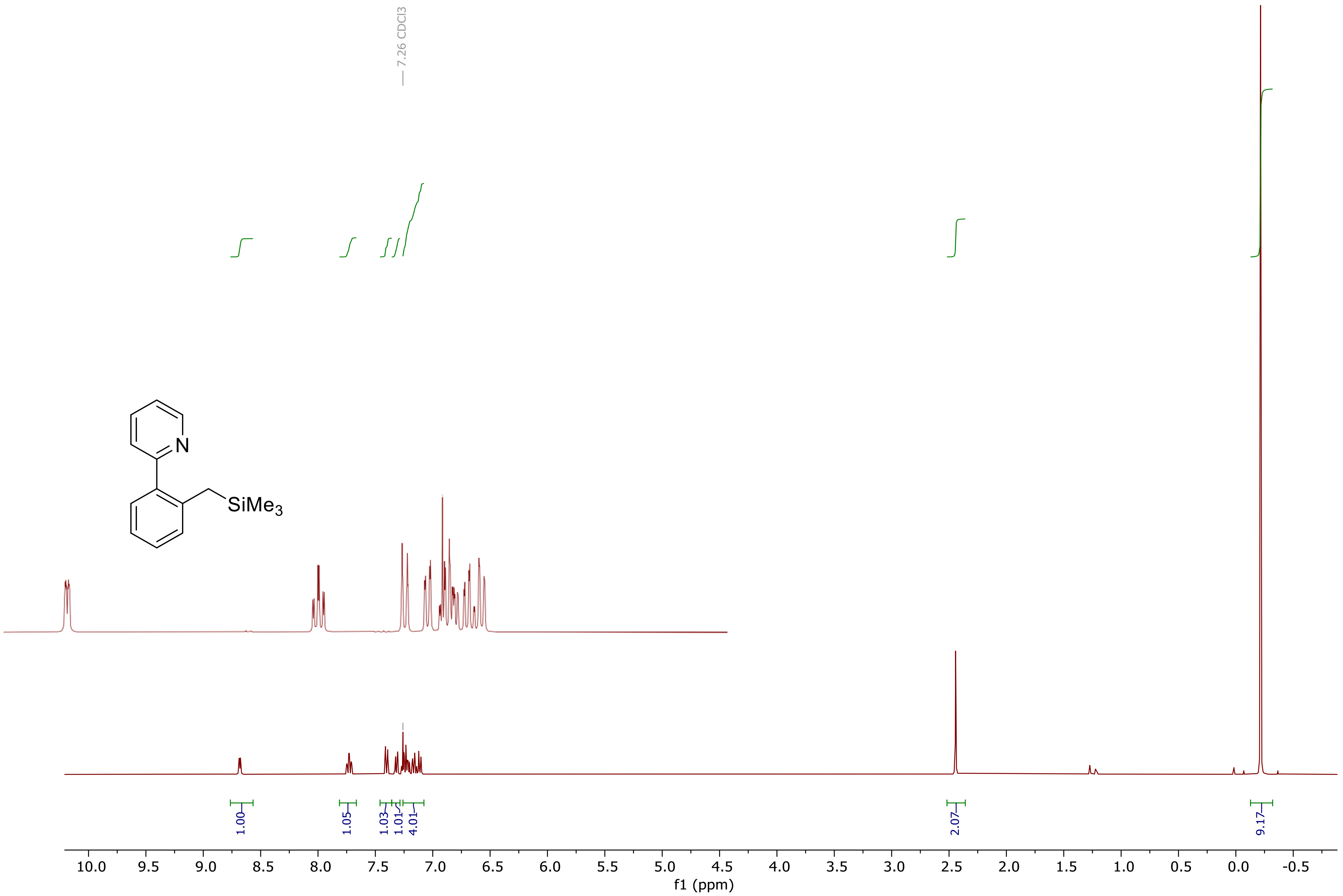

Supplementary Figure 120. ^{1}H NMR (400 MHz, $CDCl_3$) of 2-{2-[(trimethylsilyl)methyl]phenyl}pyridine **4a**.

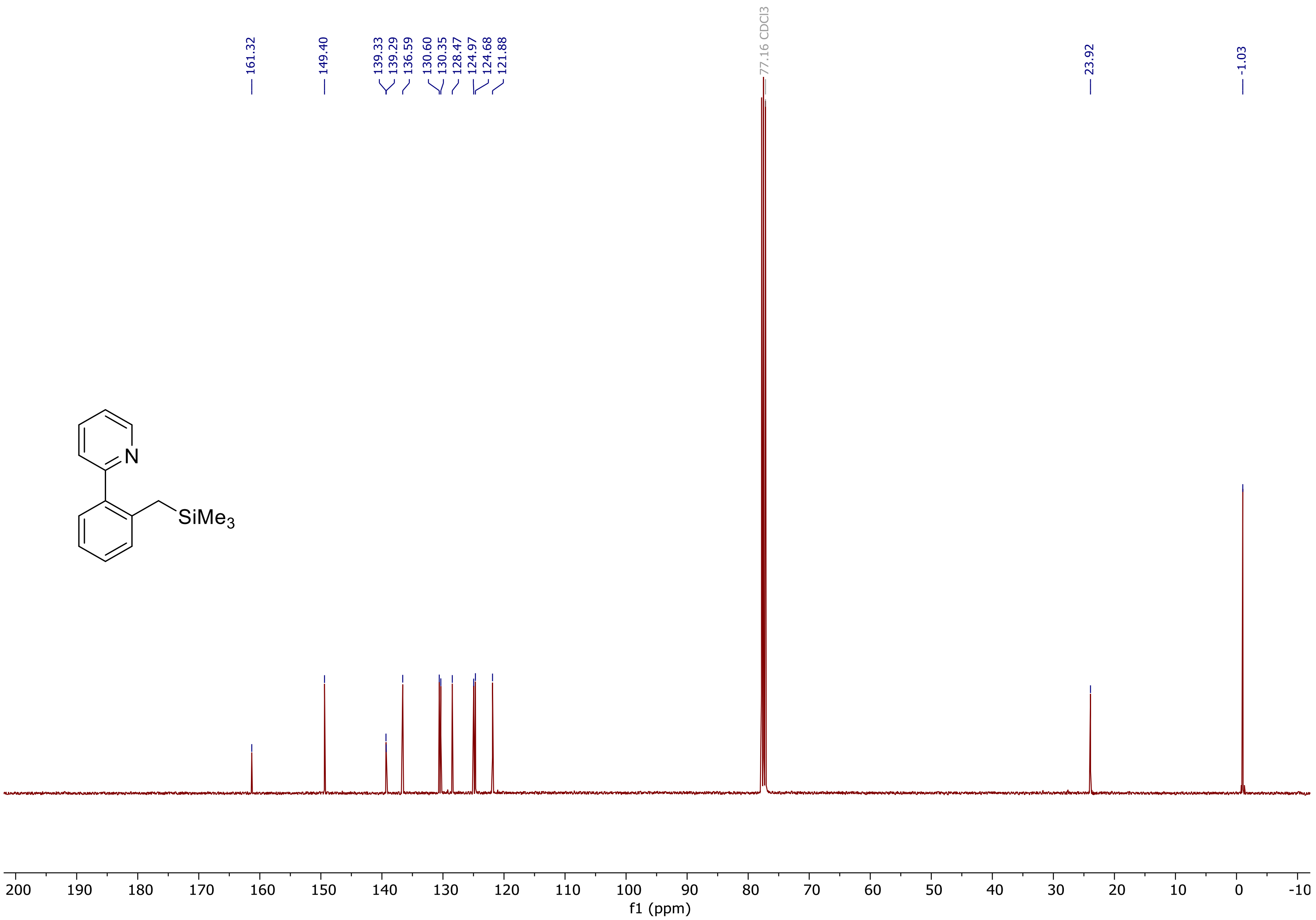

Supplementary Figure 121. ^{13}C NMR (101 MHz, $CDCl_3$) of 2-{2-[(trimethylsilyl)methyl]phenyl}pyridine **4a**.

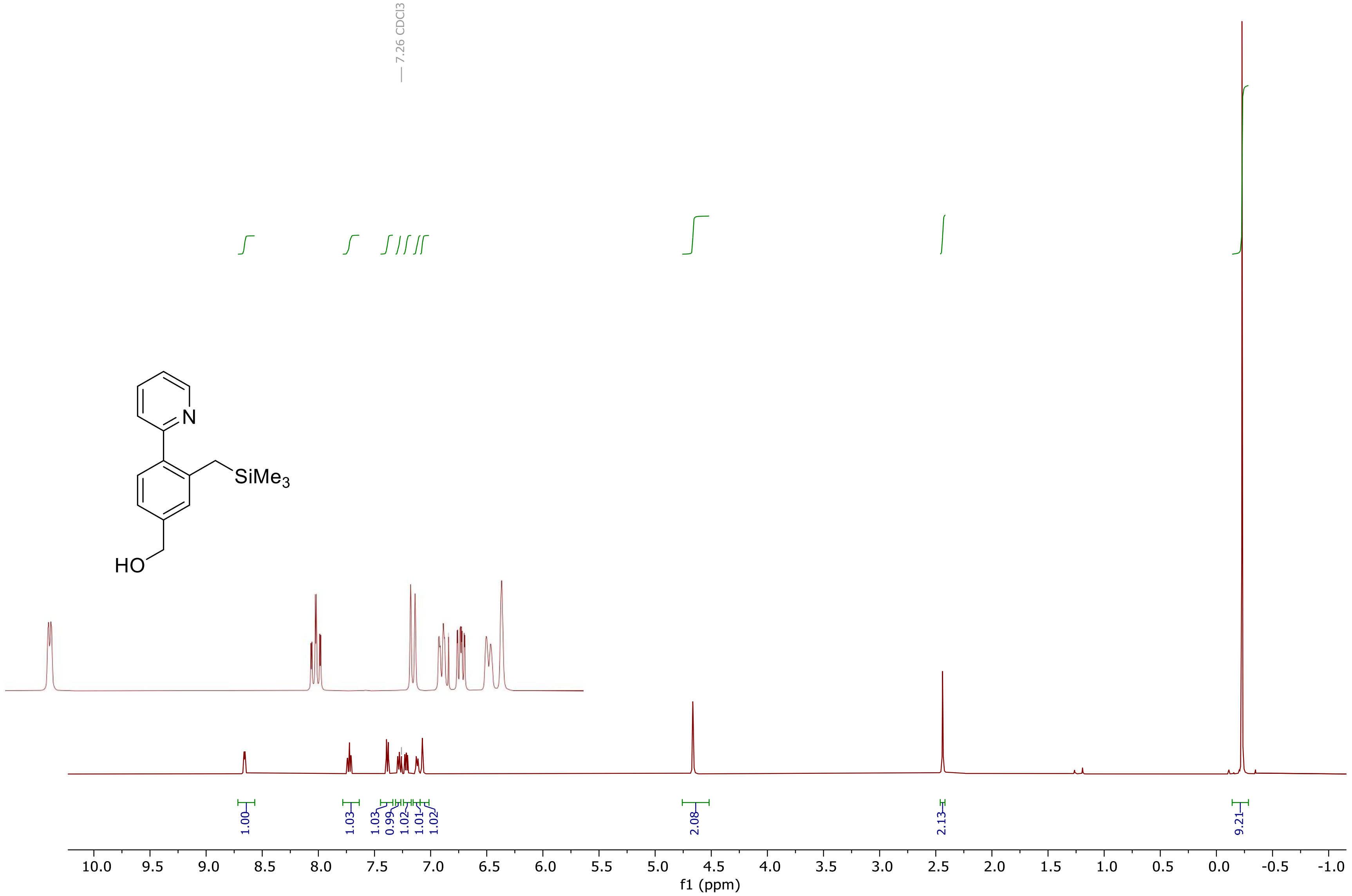

Supplementary Figure 122. ^1H NMR (500 MHz, $CDCl_3$) of {4-(pyridin-2-yl)-3-[(trimethylsilyl)methyl]phenyl}methanol **4b**.

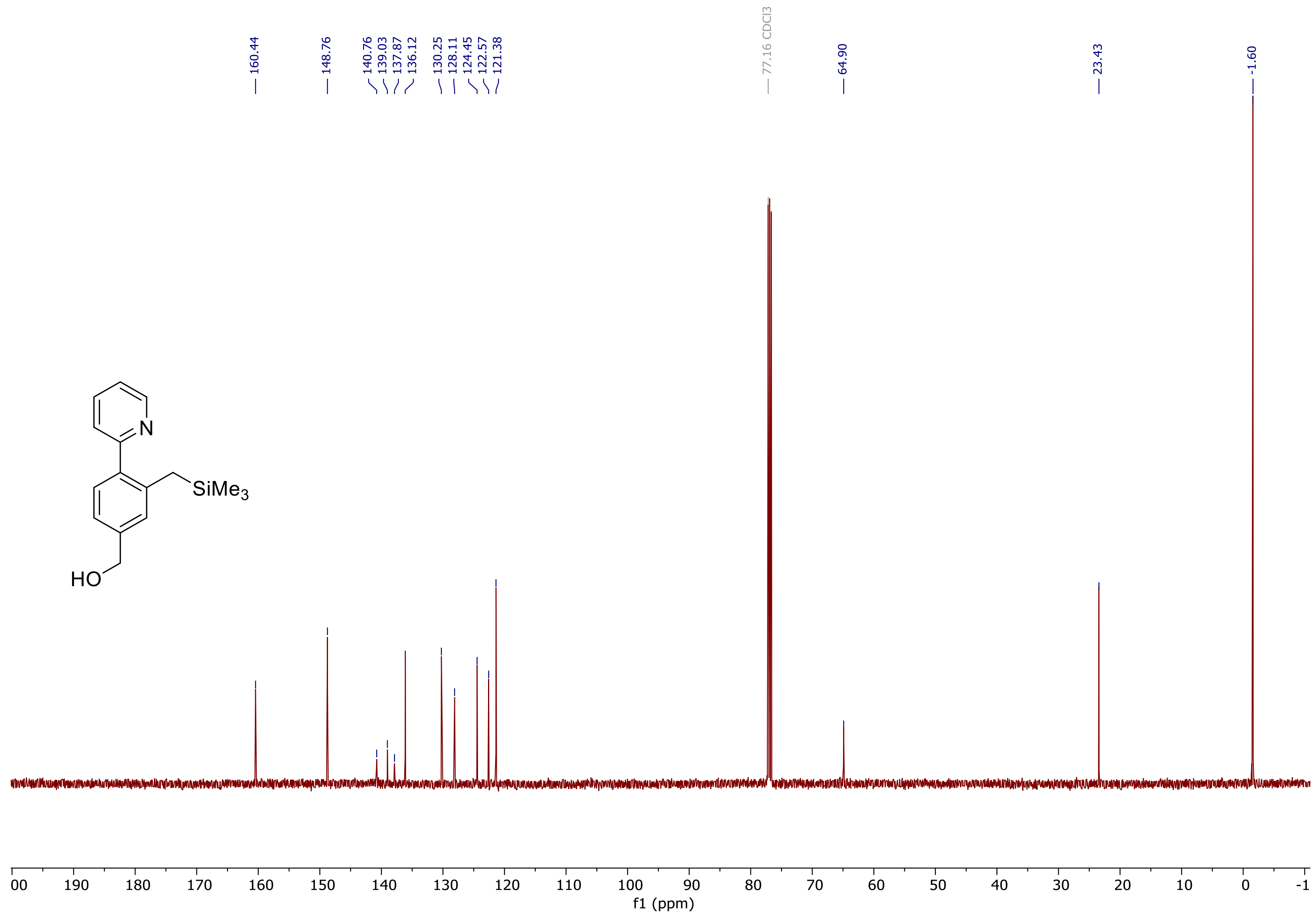

Supplementary Figure 123. ^{13}C NMR (126 MHz, $CDCl_3$) of {4-(pyridin-2-yl)-3-[(trimethylsilyl)methyl]phenyl}methanol **4b**.

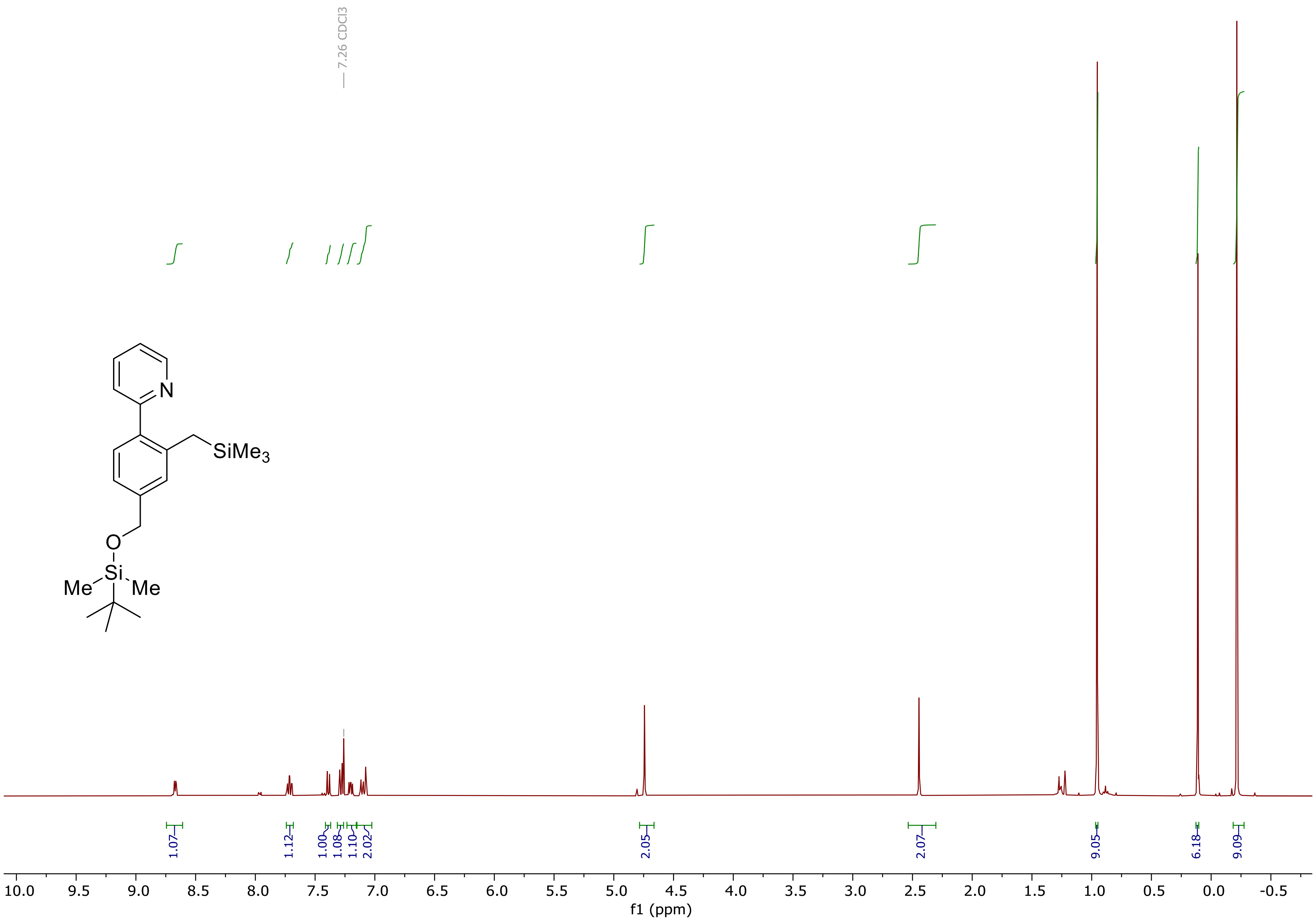

Supplementary Figure 124. ^{1}H NMR (500 MHz, $CDCl_3$) of 2-{4-[((*tert*-butyldimethylsilyl)oxy)methyl]-2-[(trimethylsilyl)methyl]phenyl}pyridine **4c**.

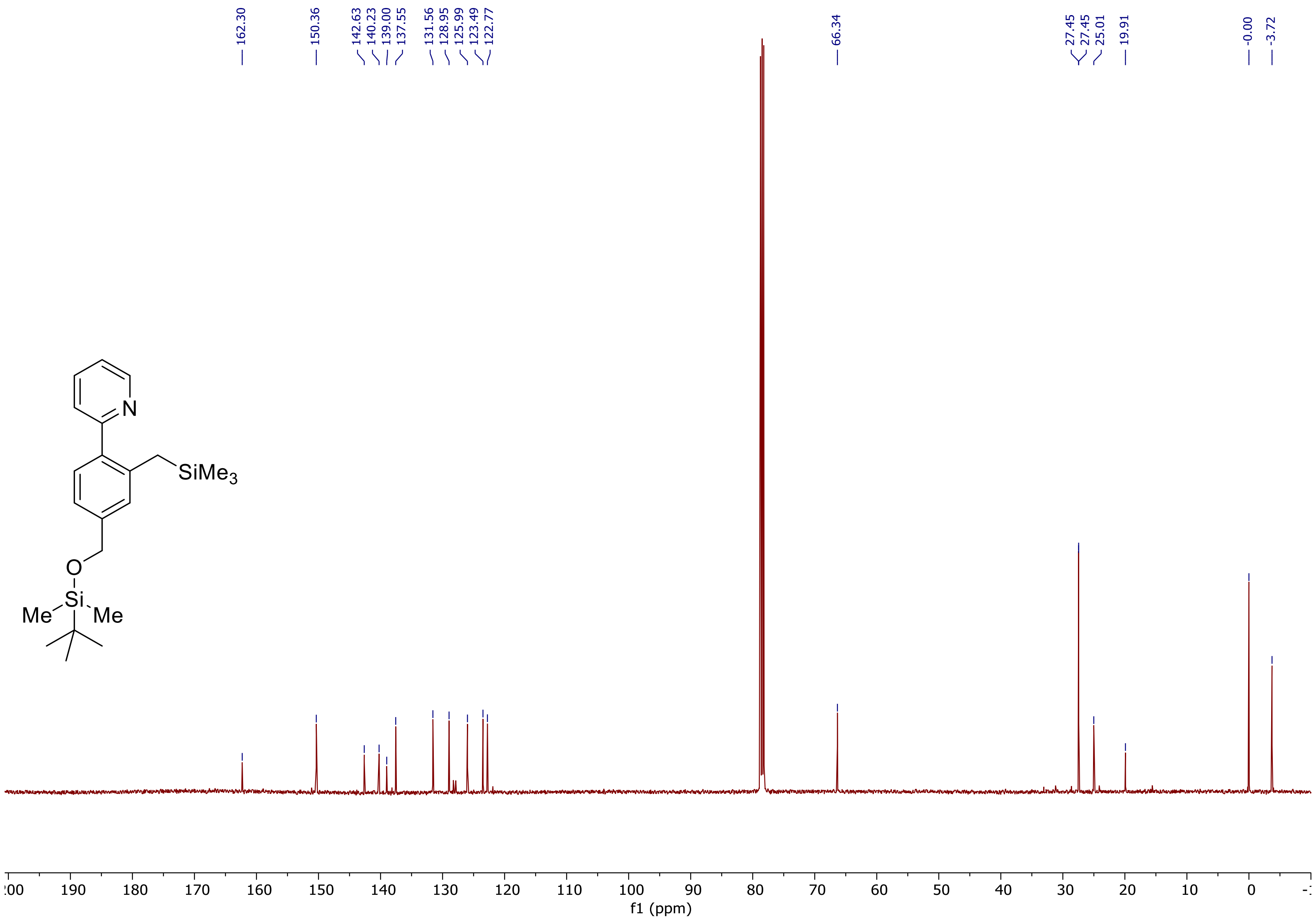

Supplementary Figure 125. ^{13}C NMR (101 MHz, $CDCl_3$) of 2-{4-[((*tert*-butyldimethylsilyl)oxy)methyl]-2-[(trimethylsilyl)methyl]phenyl}pyridine **4c**.

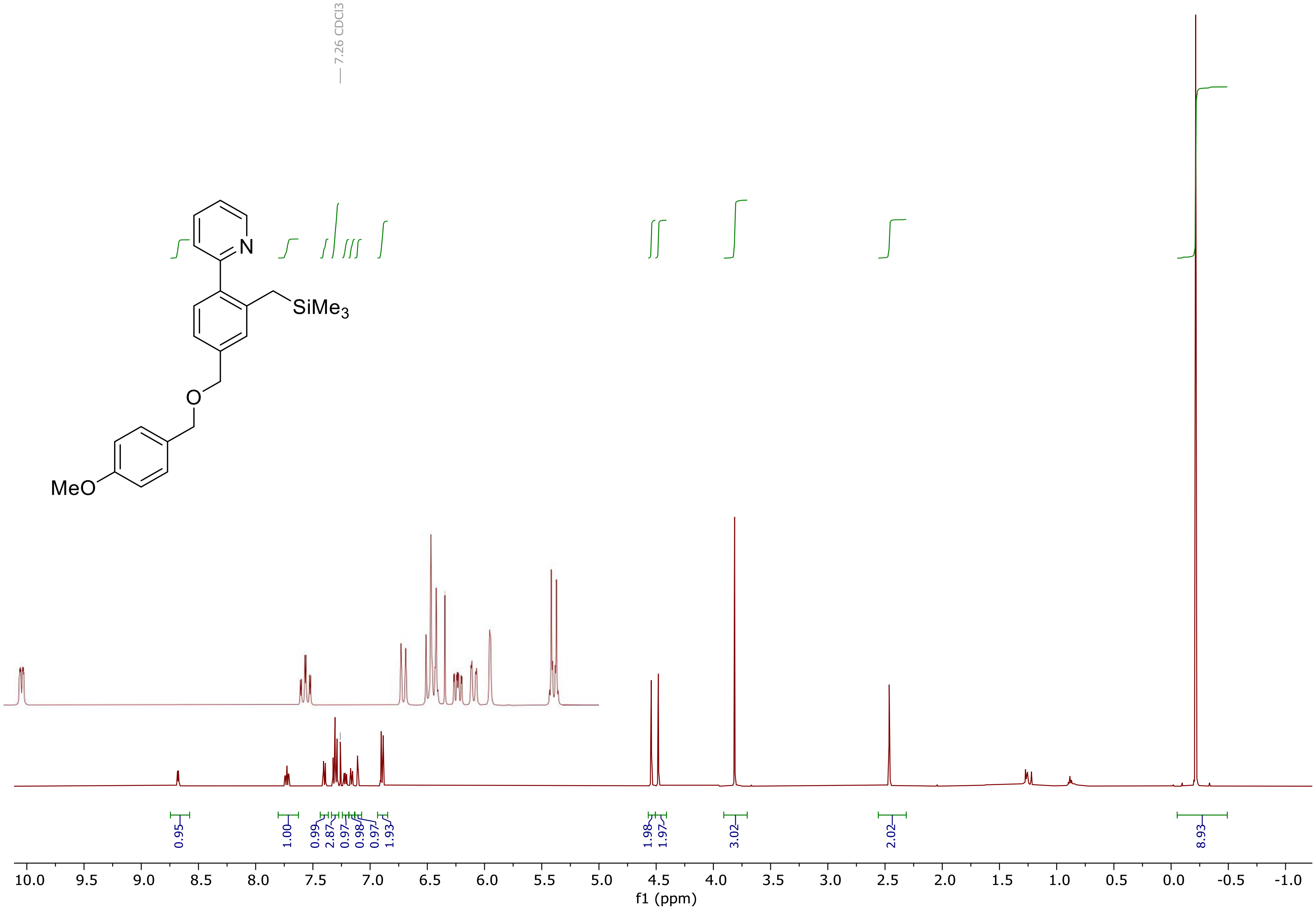

Supplementary Figure 126. ^{1}H NMR (500 MHz, $CDCl_3$) of 2-{4-[((4-methoxybenzyl)oxy)methyl]-2-[(trimethylsilyl)methyl]phenyl}pyridine **4d**.

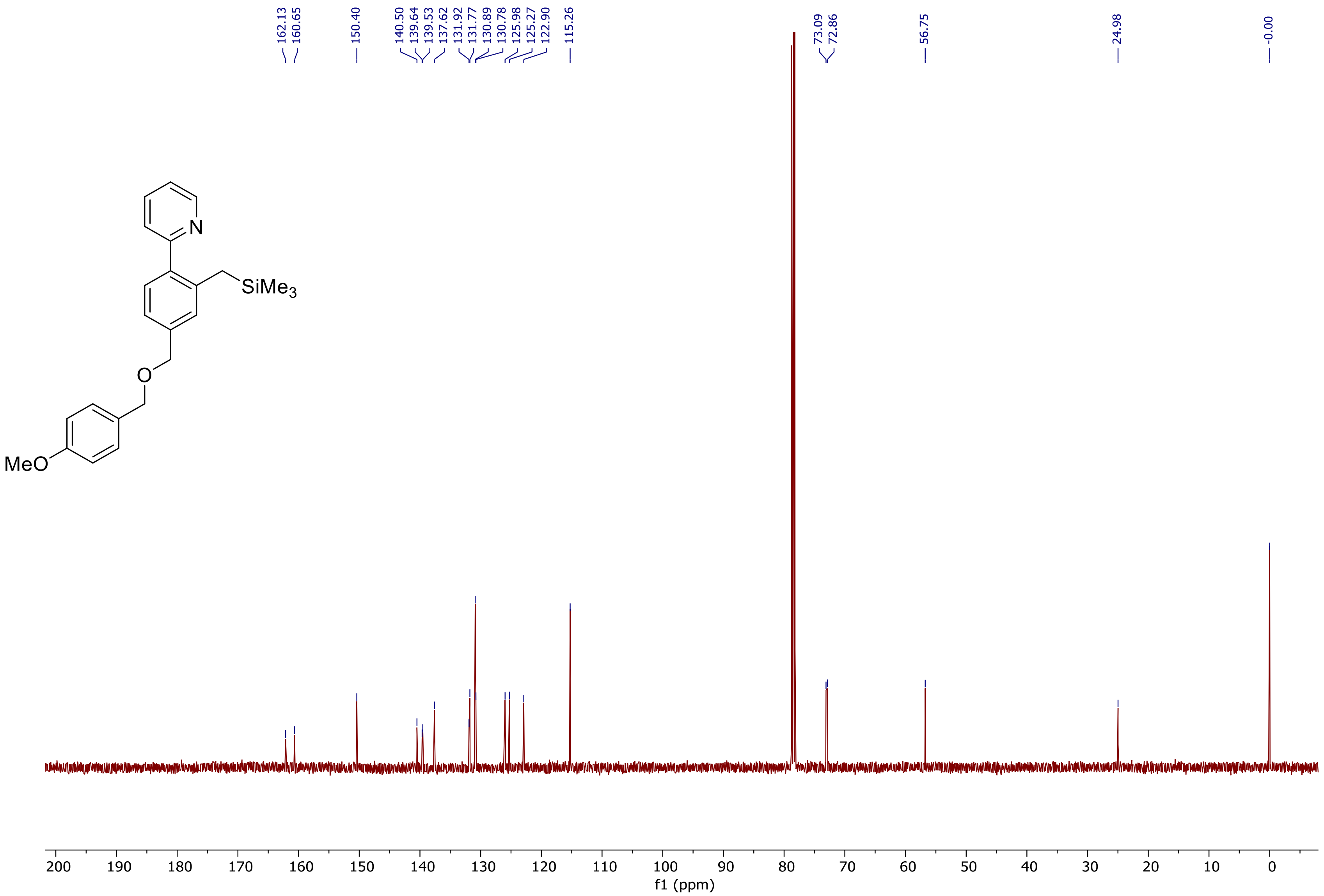

Supplementary Figure 127. ^{13}C NMR (126 MHz, $CDCl_3$) of 2-{4-[((4-methoxybenzyl)oxy)methyl]-2-[(trimethylsilyl)methyl]phenyl}pyridine **4d**.

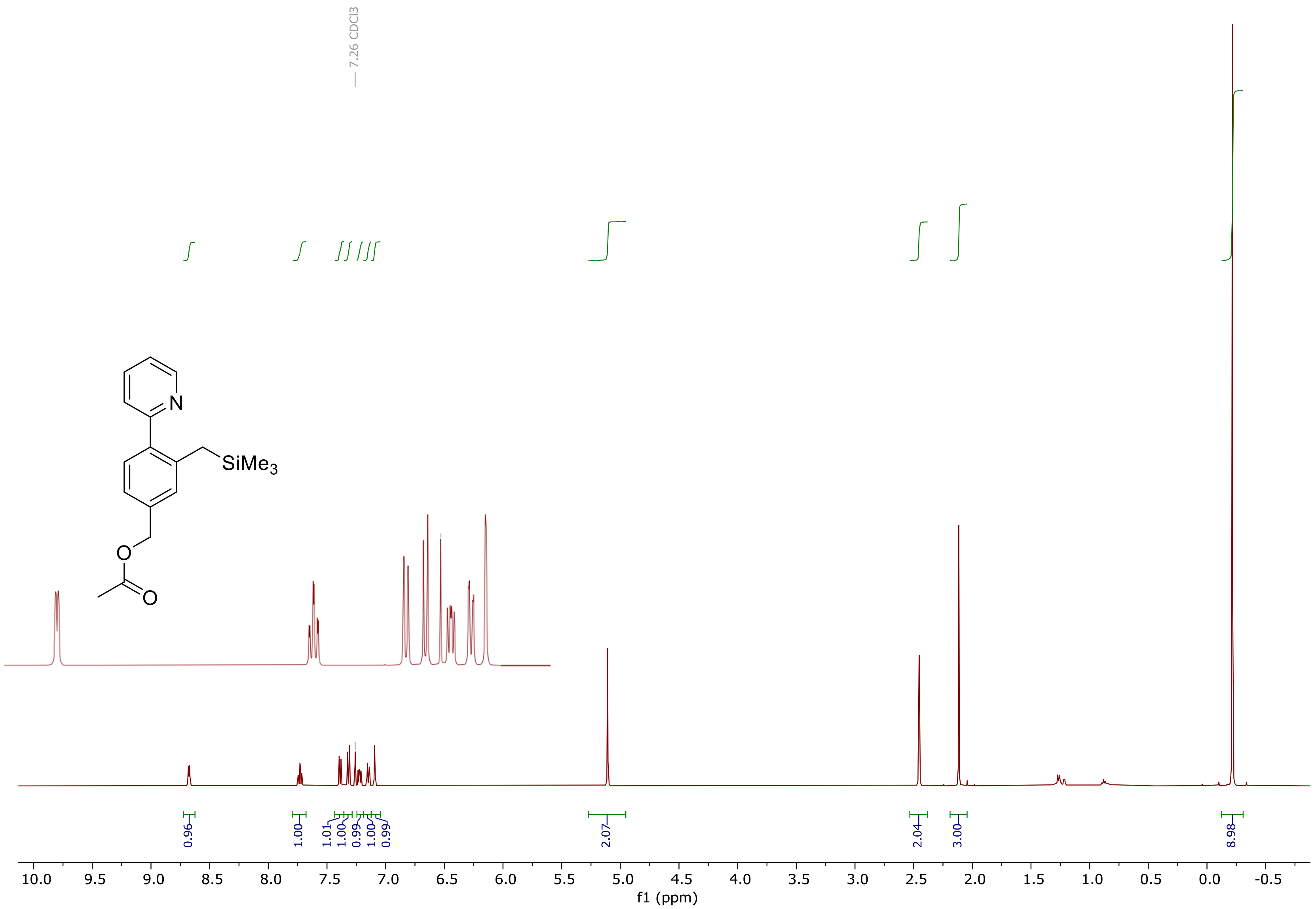

Supplementary Figure 128. ^{1}H NMR (500 MHz, $CDCl_3$) of 4-(pyridin-2-yl)-3-[(trimethylsilyl)methyl]benzyl acetate **4e**

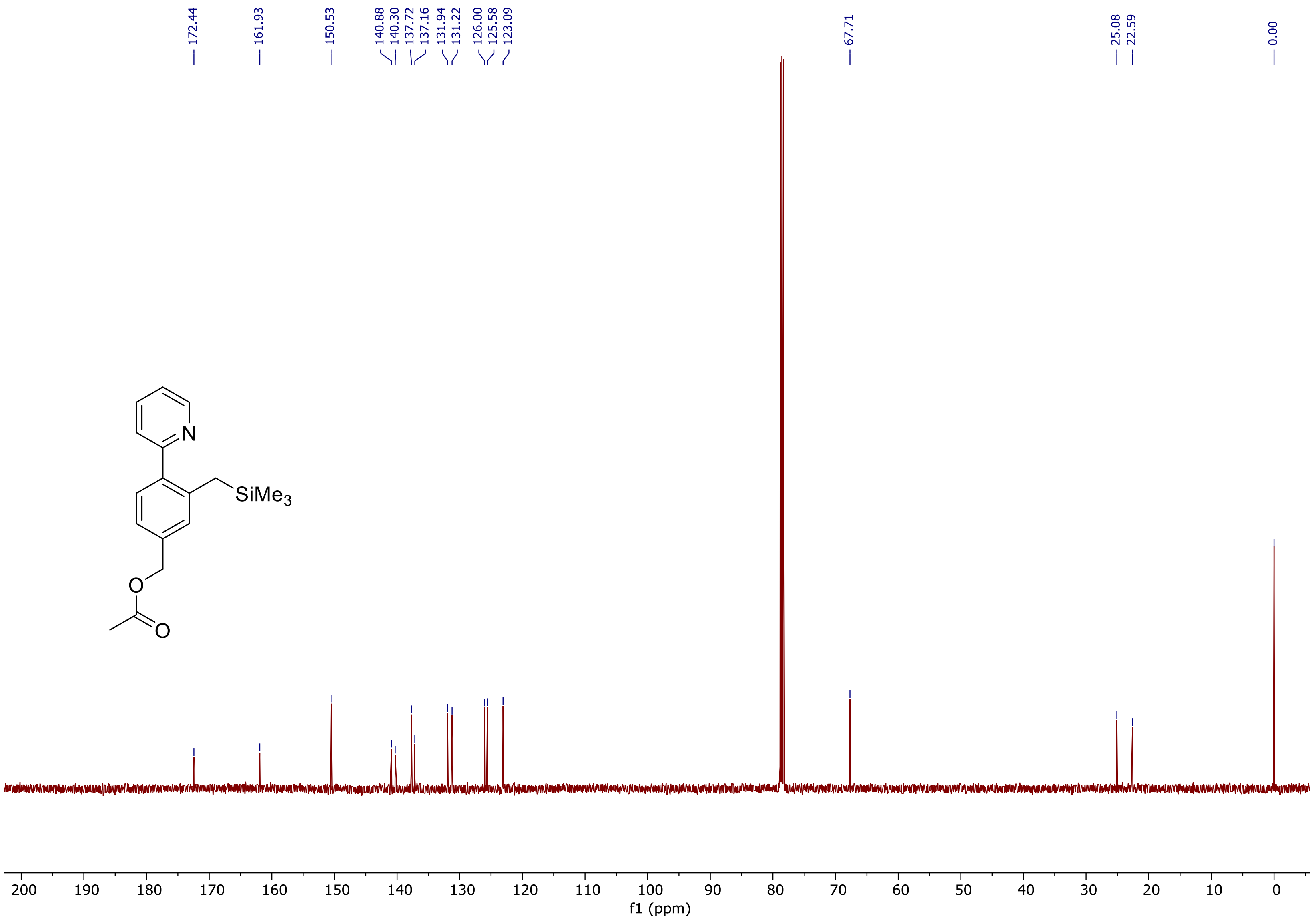

Supplementary Figure 129. ^{13}C NMR (126 MHz, $CDCl_3$) of 4-(pyridin-2-yl)-3-[(trimethylsilyl)methyl]benzyl acetate **4e**.

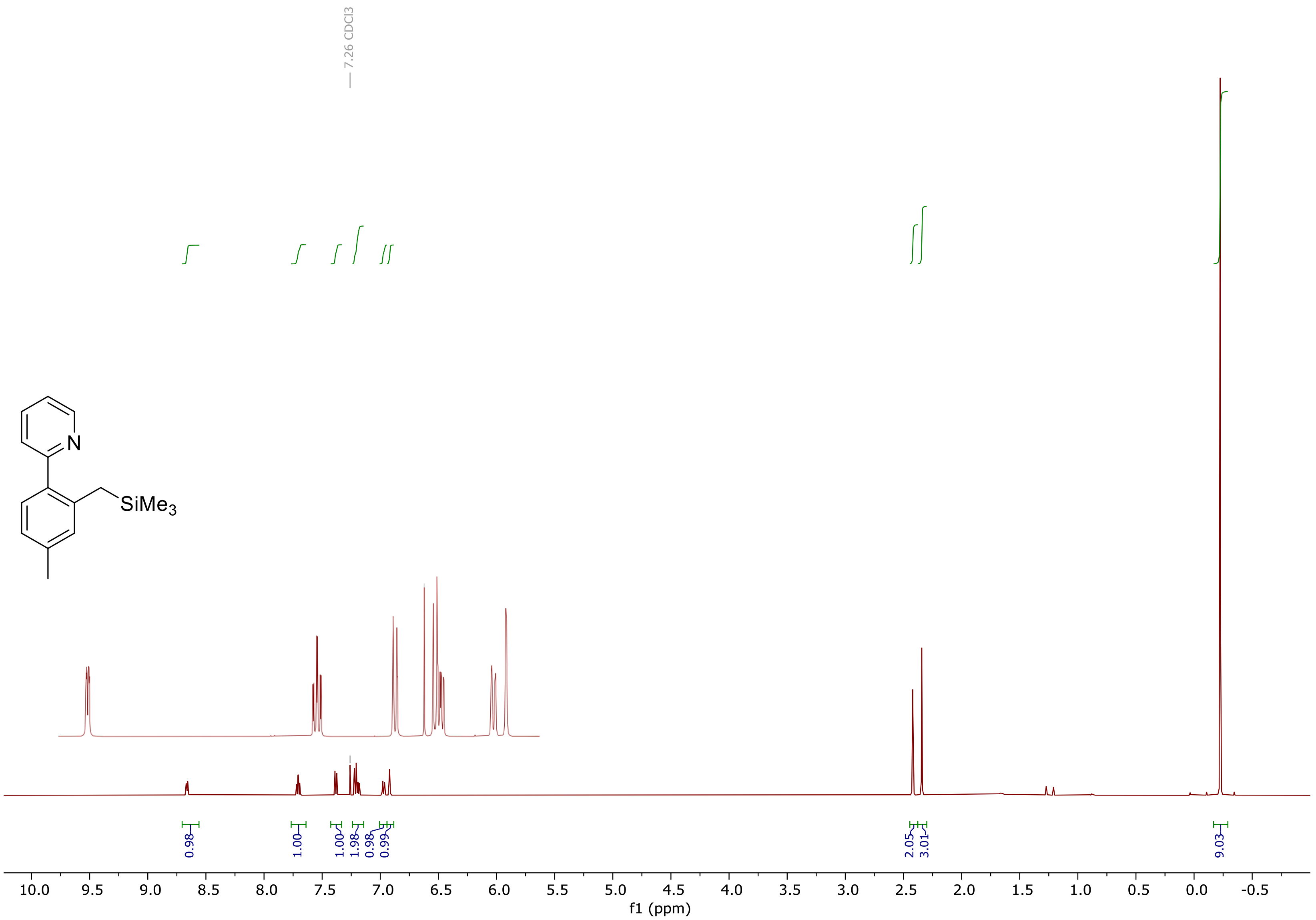

Supplementary Figure 130. ^1H NMR (500 MHz, $CDCl_3$) of 2-{4-methyl-2-[(trimethylsilyl)methyl]phenyl}pyridine **4f**.

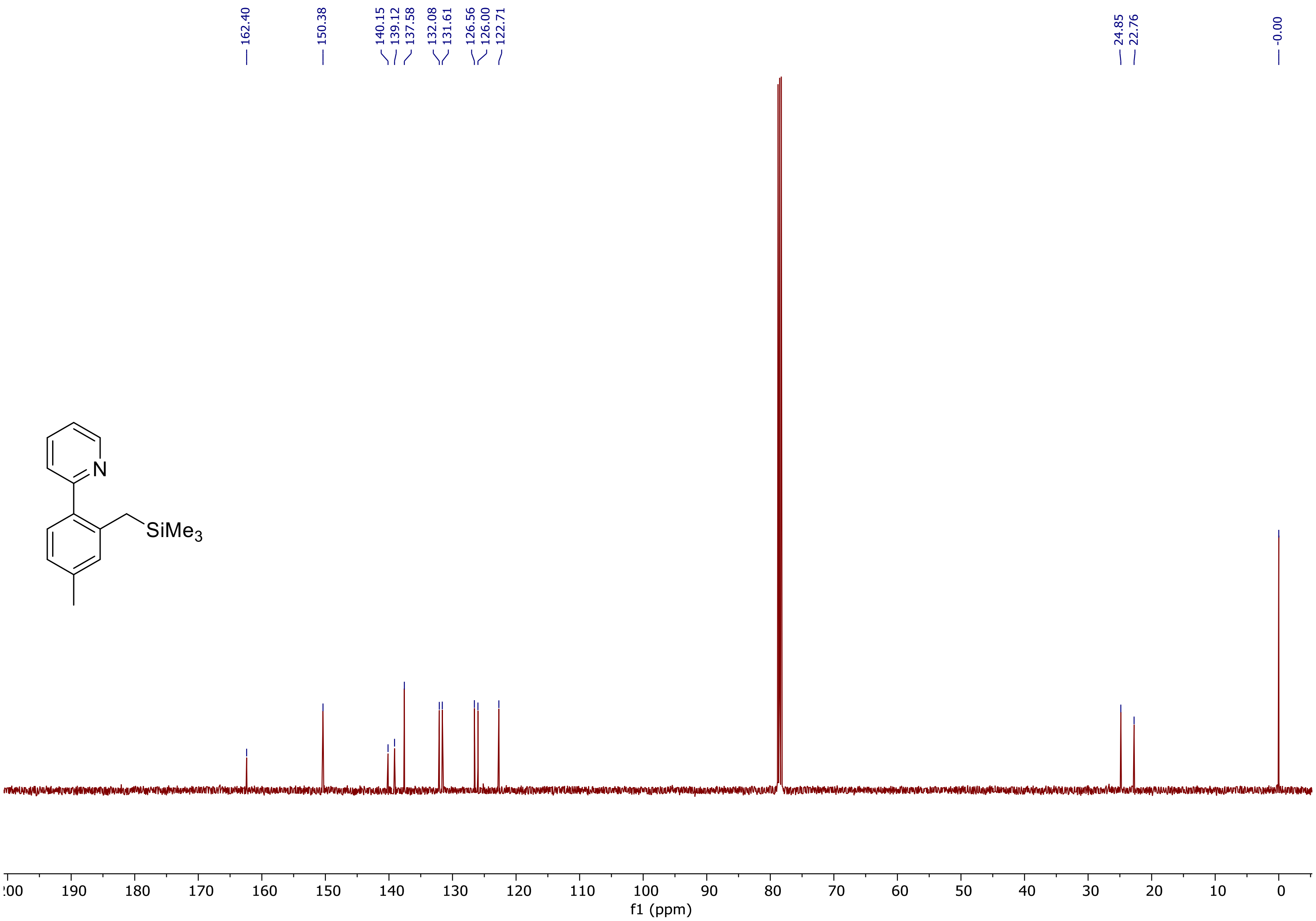

Supplementary Figure 131. ^{13}C NMR (126 MHz, $CDCl_3$) of 2-{4-methyl-2-[(trimethylsilyl)methyl]phenyl}pyridine **4f**.

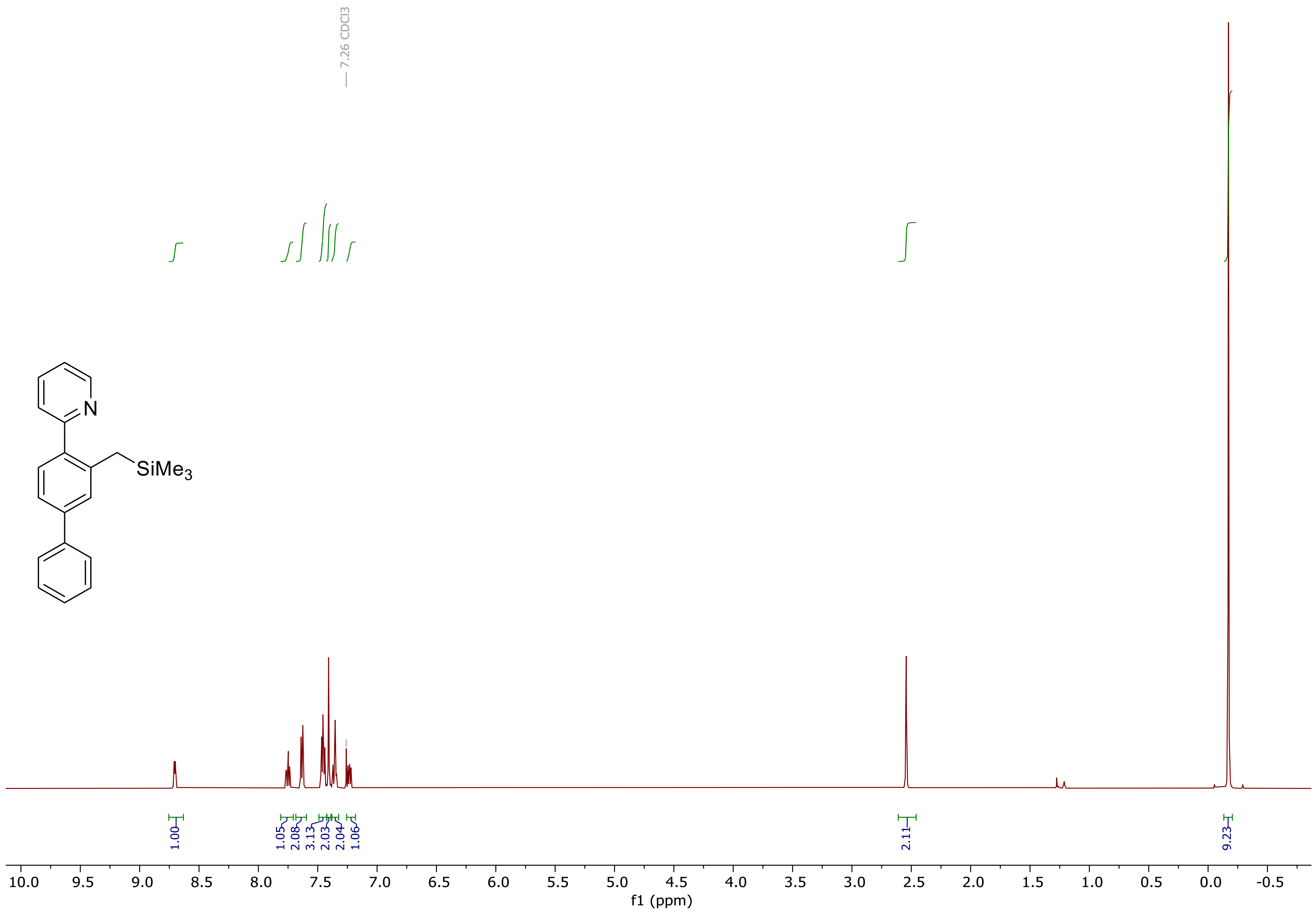

Supplementary Figure 132. ^1H NMR (500 MHz, $CDCl_3$) of 2-{3-[(trimethylsilyl)methyl]-[1,1'-biphenyl]-4-yl}pyridine **4g**.

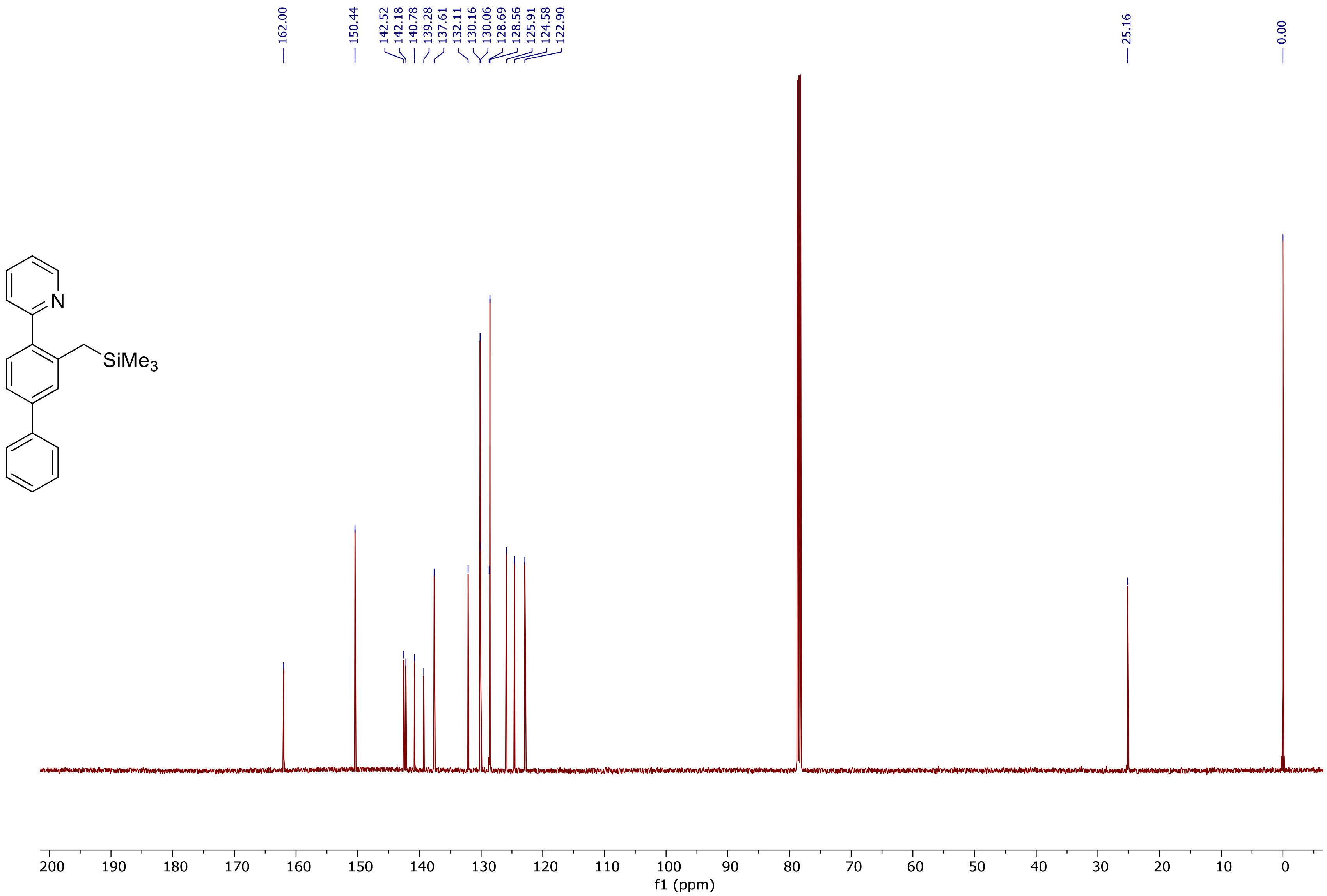

Supplementary Figure 133. ^{13}C NMR (126 MHz, $CDCl_3$) of 2-{3-[(trimethylsilyl)methyl]-[1,1'-biphenyl]-4-yl}pyridine **4g**.

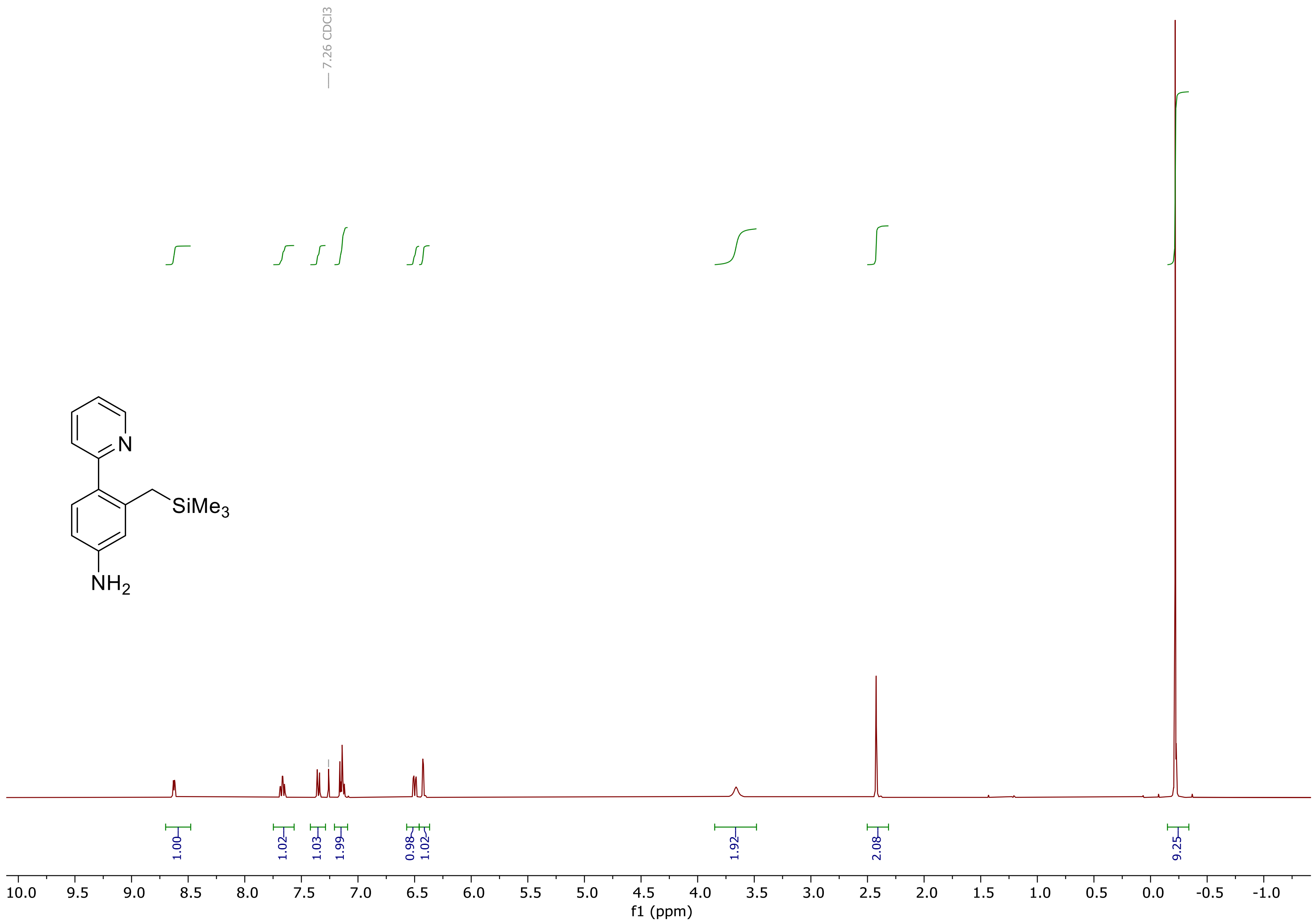

Supplementary Figure 134. ^{1}H NMR (400 MHz, $CDCl_3$) of 4-(pyridin-2-yl)-3-[(trimethylsilyl)methyl]aniline **4h**.

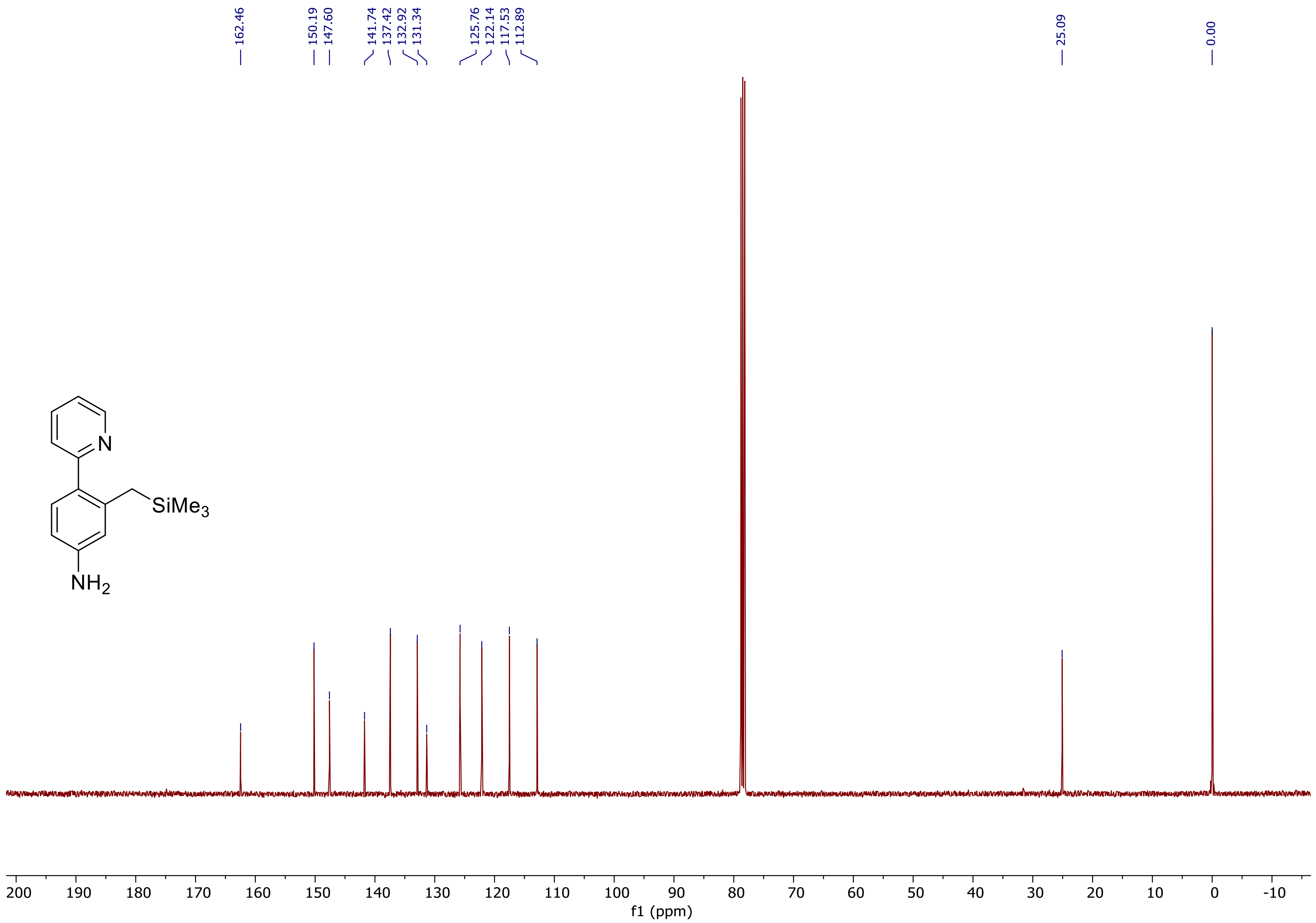

Supplementary Figure 135. ^{13}C NMR (101 MHz, $CDCl_3$) of 4-(pyridin-2-yl)-3-[(trimethylsilyl)methyl]aniline **4h**.

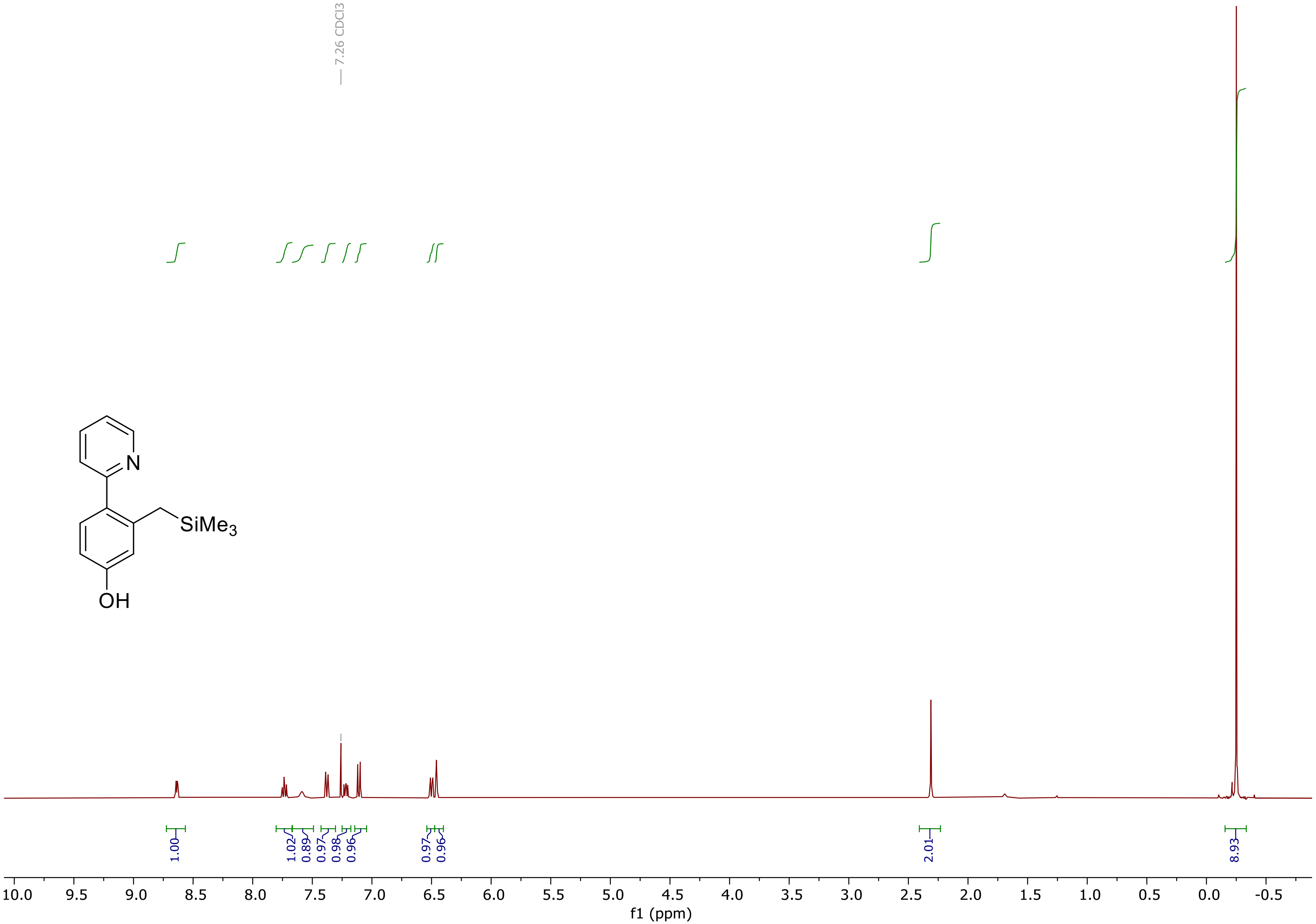

Supplementary Figure 136. ^{1}H NMR (400 MHz, $CDCl_3$) of 4-(pyridin-2-yl)-3-[(trimethylsilyl)methyl]phenol **4i**.

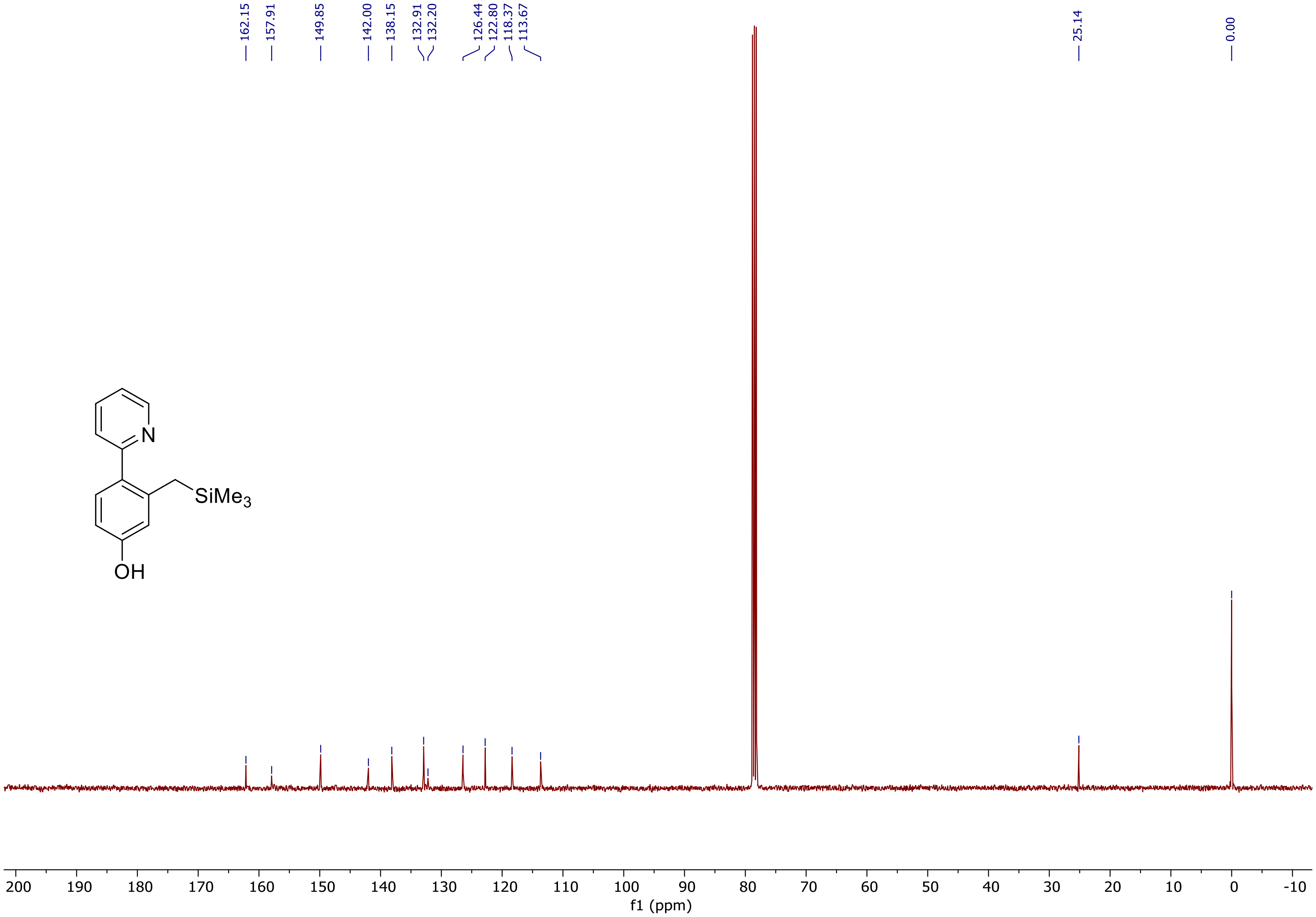

Supplementary Figure 137. ^{13}C NMR (101 MHz, $CDCl_3$) of 4-(pyridin-2-yl)-3-[(trimethylsilyl)methyl]phenol **4i**.

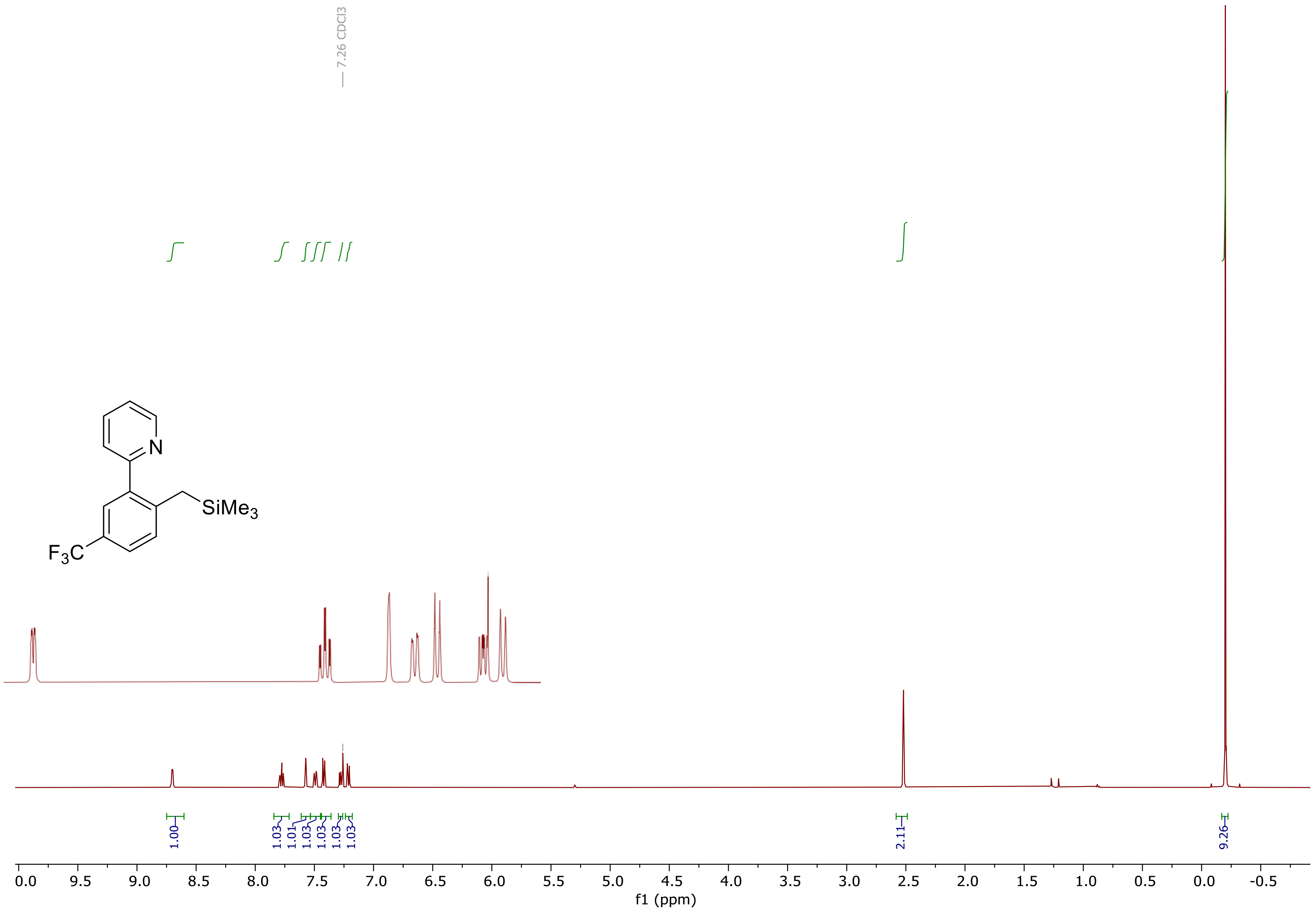

Supplementary Figure 138. ^{1}H NMR (500 MHz, $CDCl_3$) of 2-{5-(trifluoromethyl)-2-[(trimethylsilyl)methyl]phenyl}pyridine **4k**.

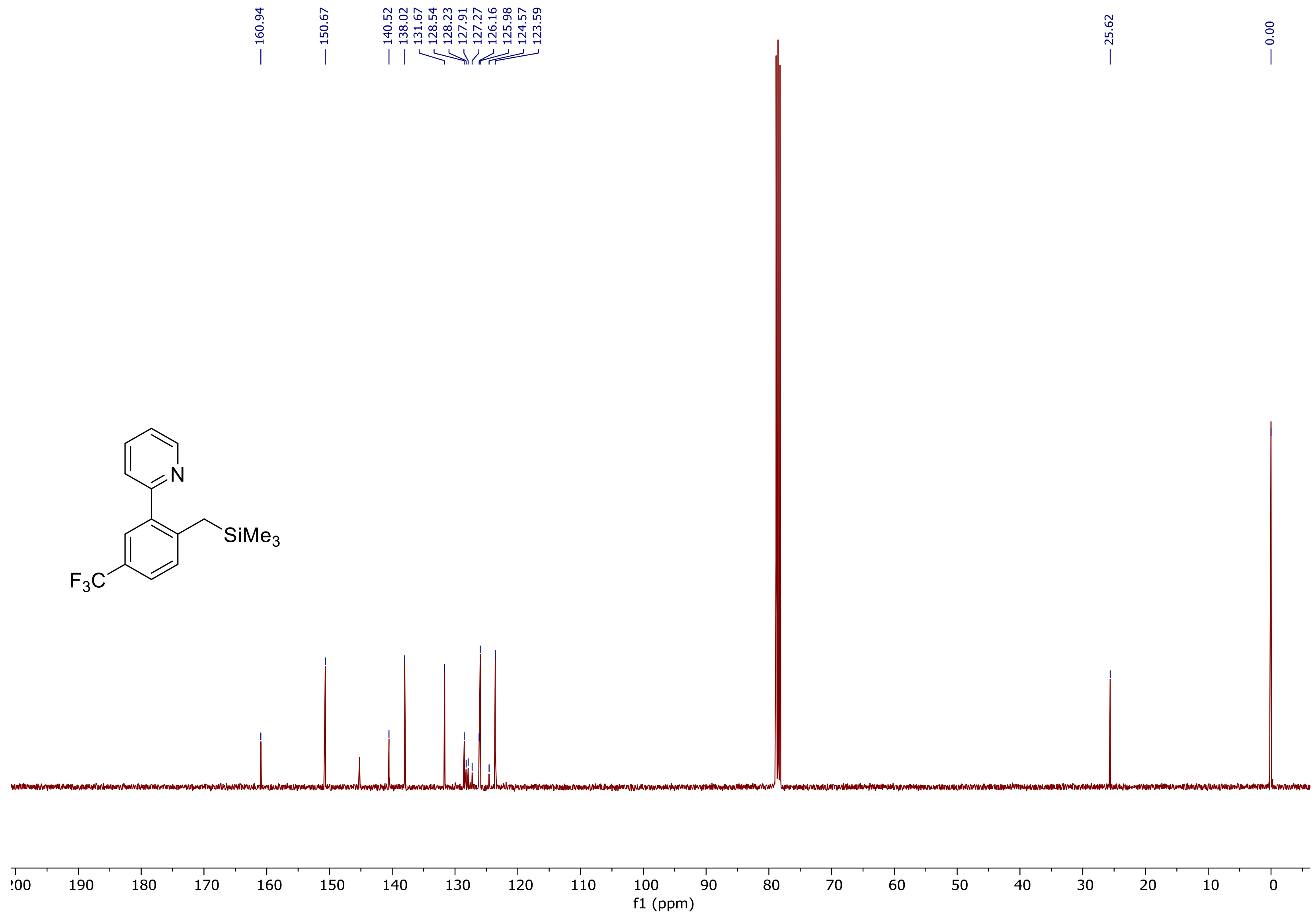

Supplementary Figure 139. ^{13}C NMR (101 MHz, $CDCl_3$) of 2-{5-(trifluoromethyl)-2-[(trimethylsilyl)methyl]phenyl}pyridine **4k**.

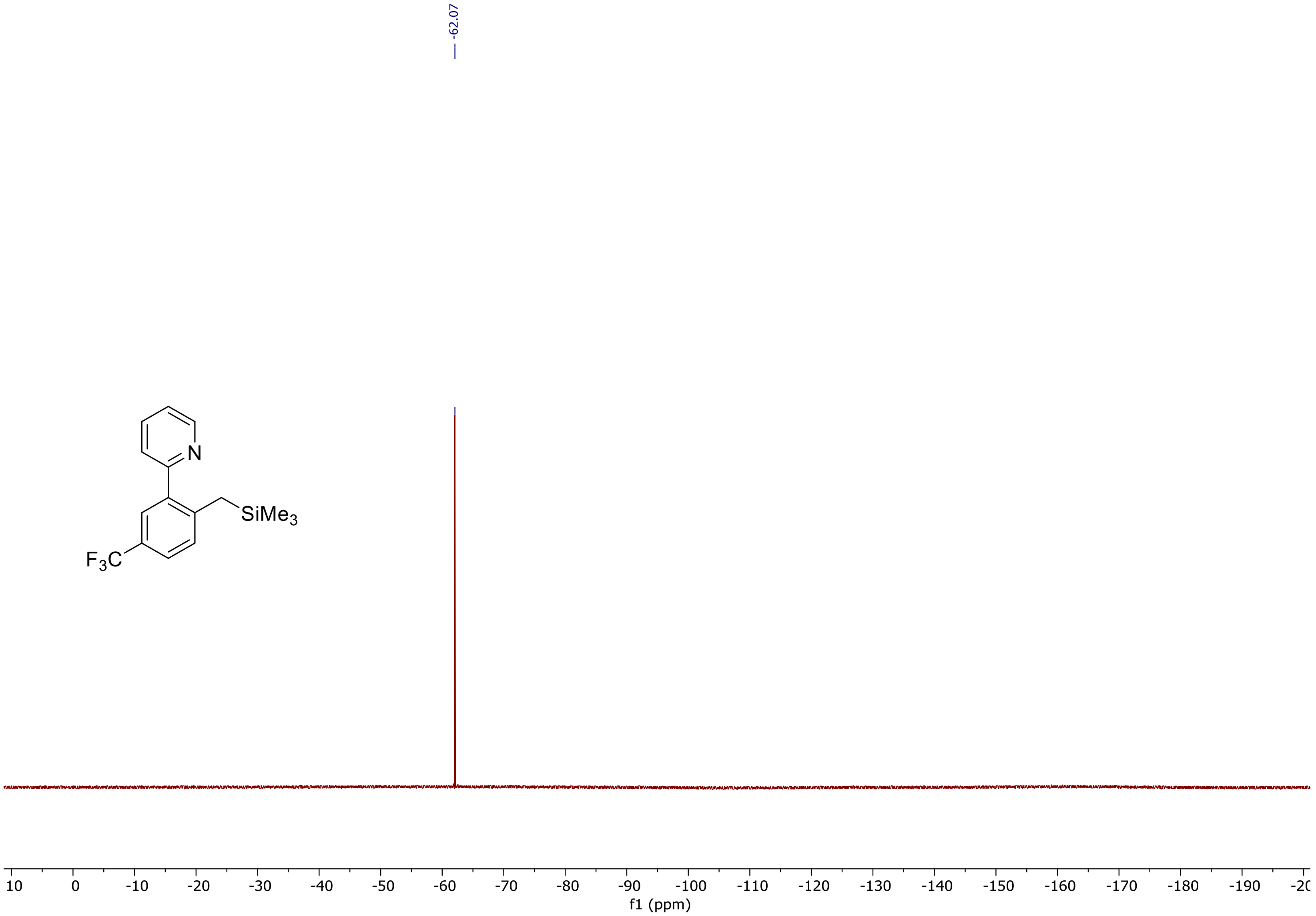

Supplementary Figure 140. ^{19}F NMR (376 MHz, $CDCl_3$) of 2-{5-(trifluoromethyl)-2-[(trimethylsilyl)methyl]phenyl}pyridine **4k**.

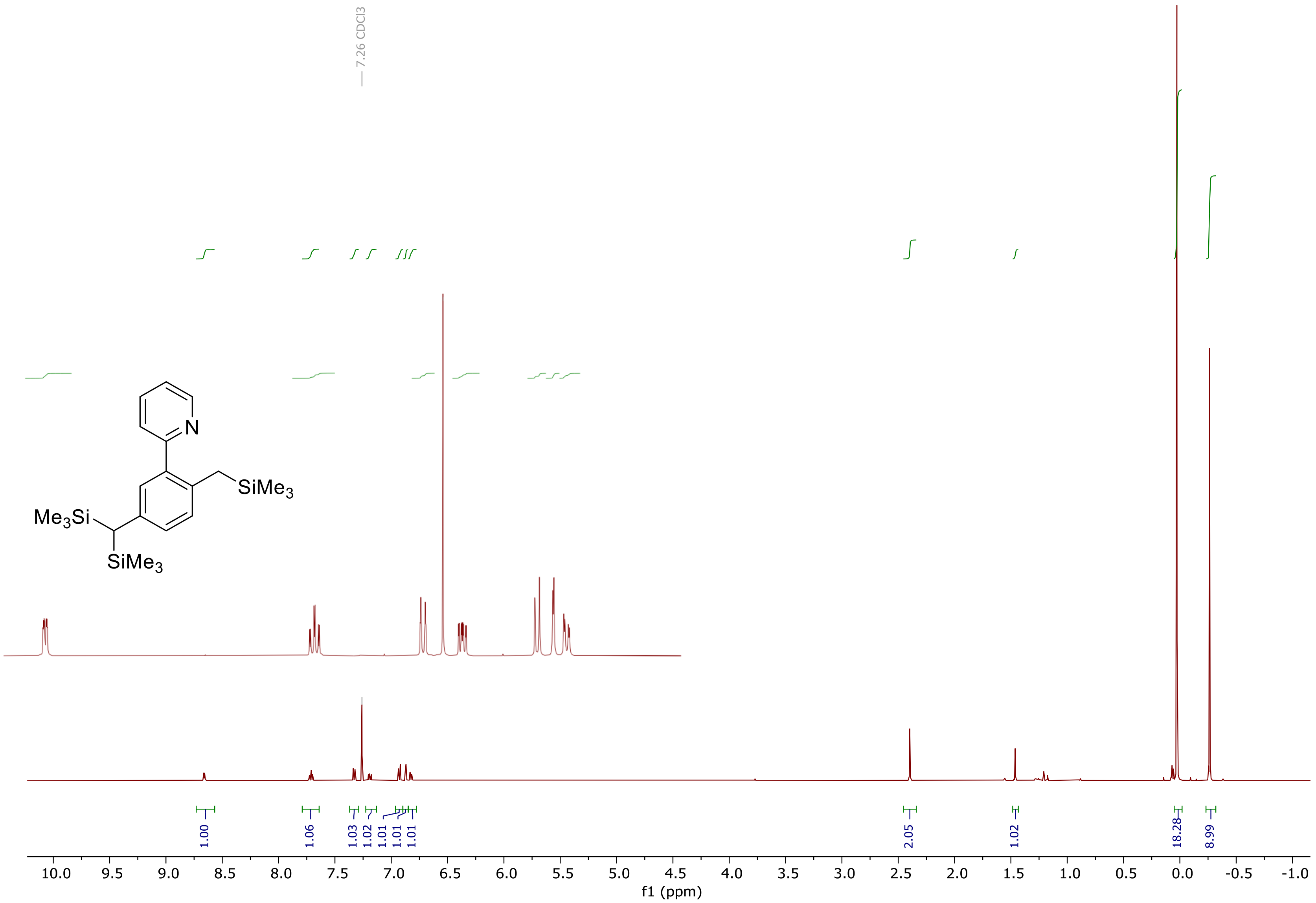

Supplementary Figure 141. ^{1}H NMR (500 MHz, $CDCl_3$) of 2-{5-[bis(trimethylsilyl)methyl]-2-[(trimethylsilyl)methyl]phenyl}pyridine **4l**.

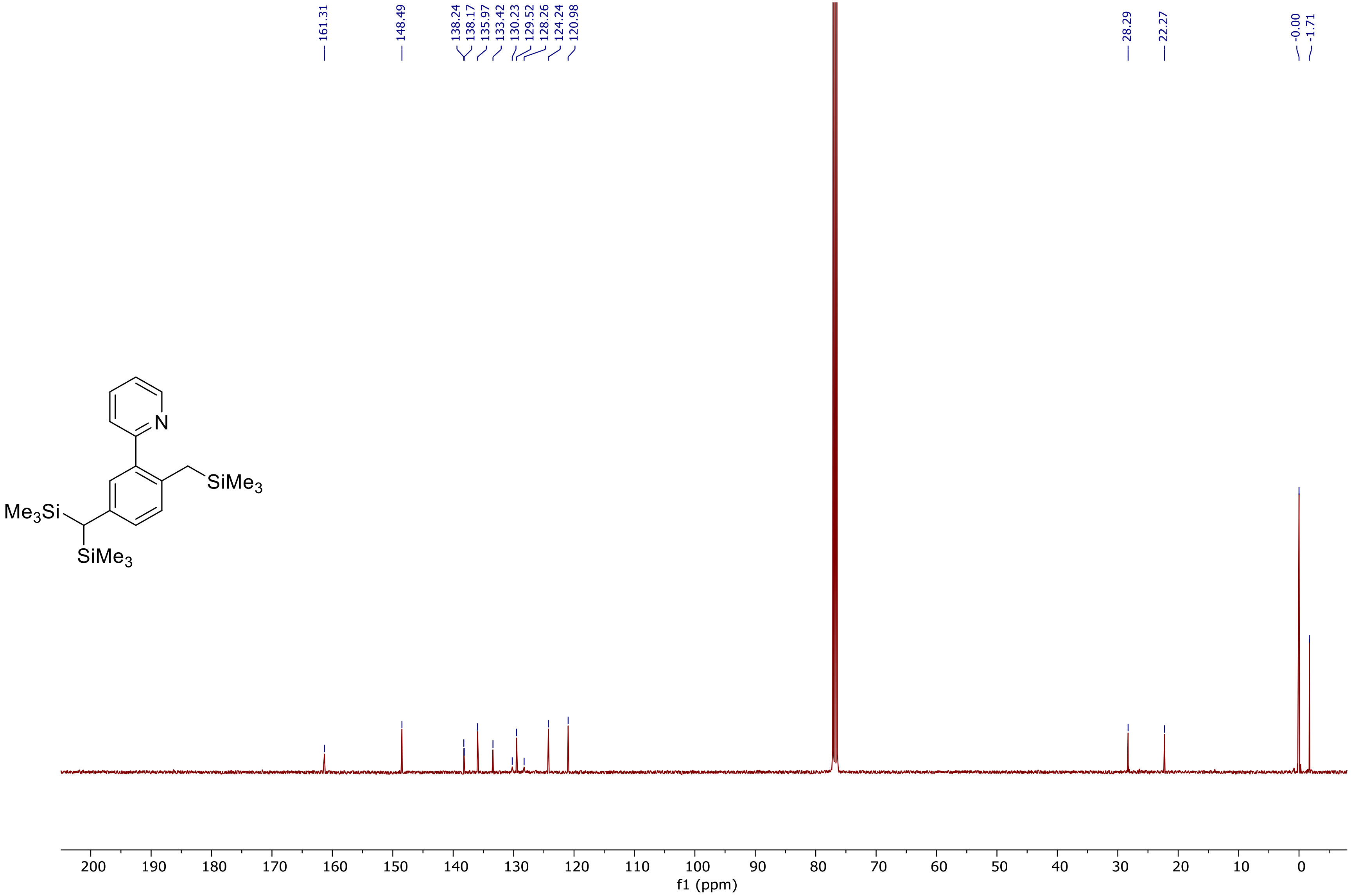

Supplementary Figure 142. ^{13}C NMR (126 MHz, $CDCl_3$) of 2-{5-[bis(trimethylsilyl)methyl]-2-[(trimethylsilyl)methyl]phenyl}pyridine **4l**.

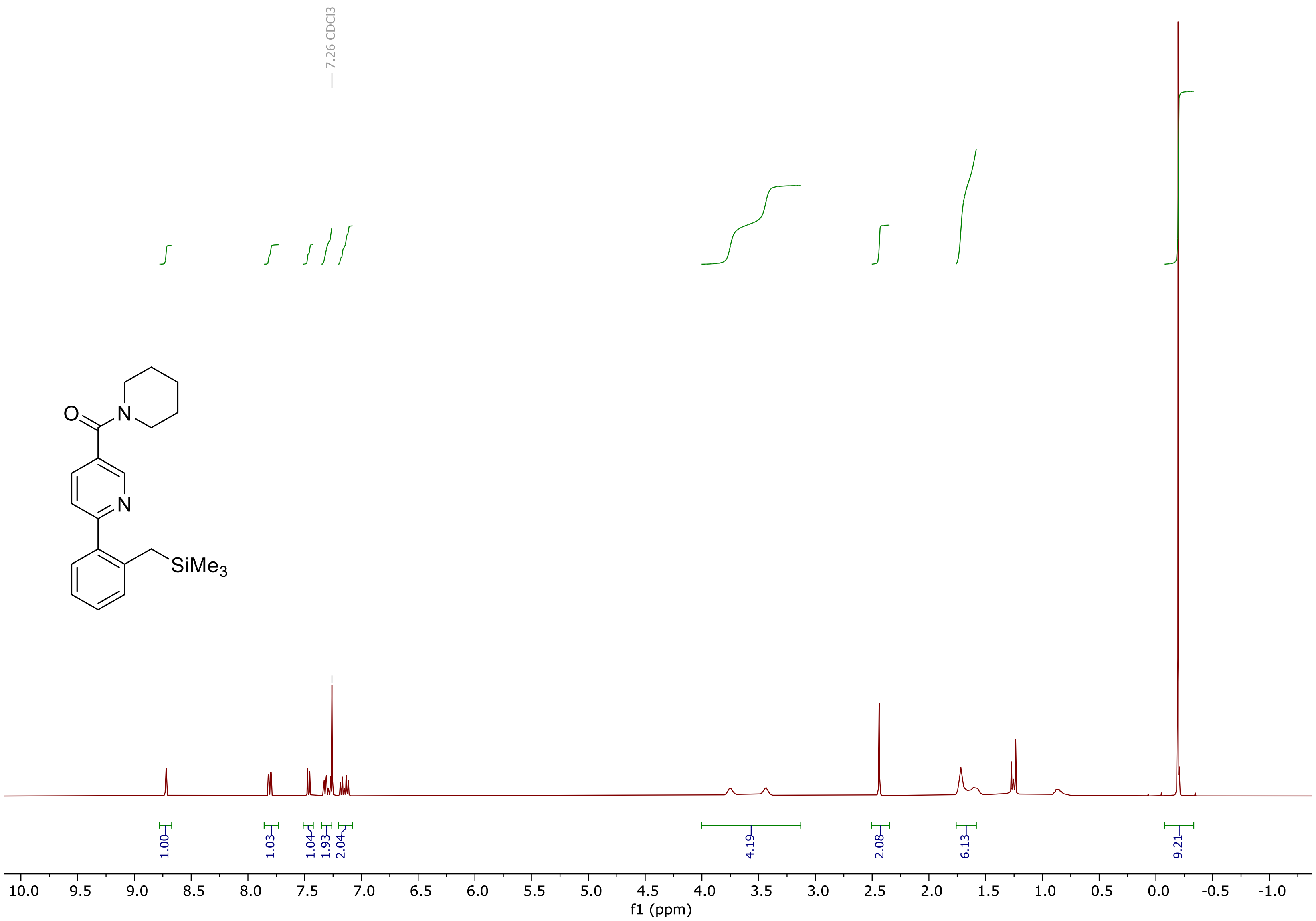

Supplementary Figure 143. ^1H NMR (400 MHz, $CDCl_3$) of piperidin-1-yl{6-[2-((trimethylsilyl)methyl)phenyl]pyridin-3-yl}methanone **4m**.

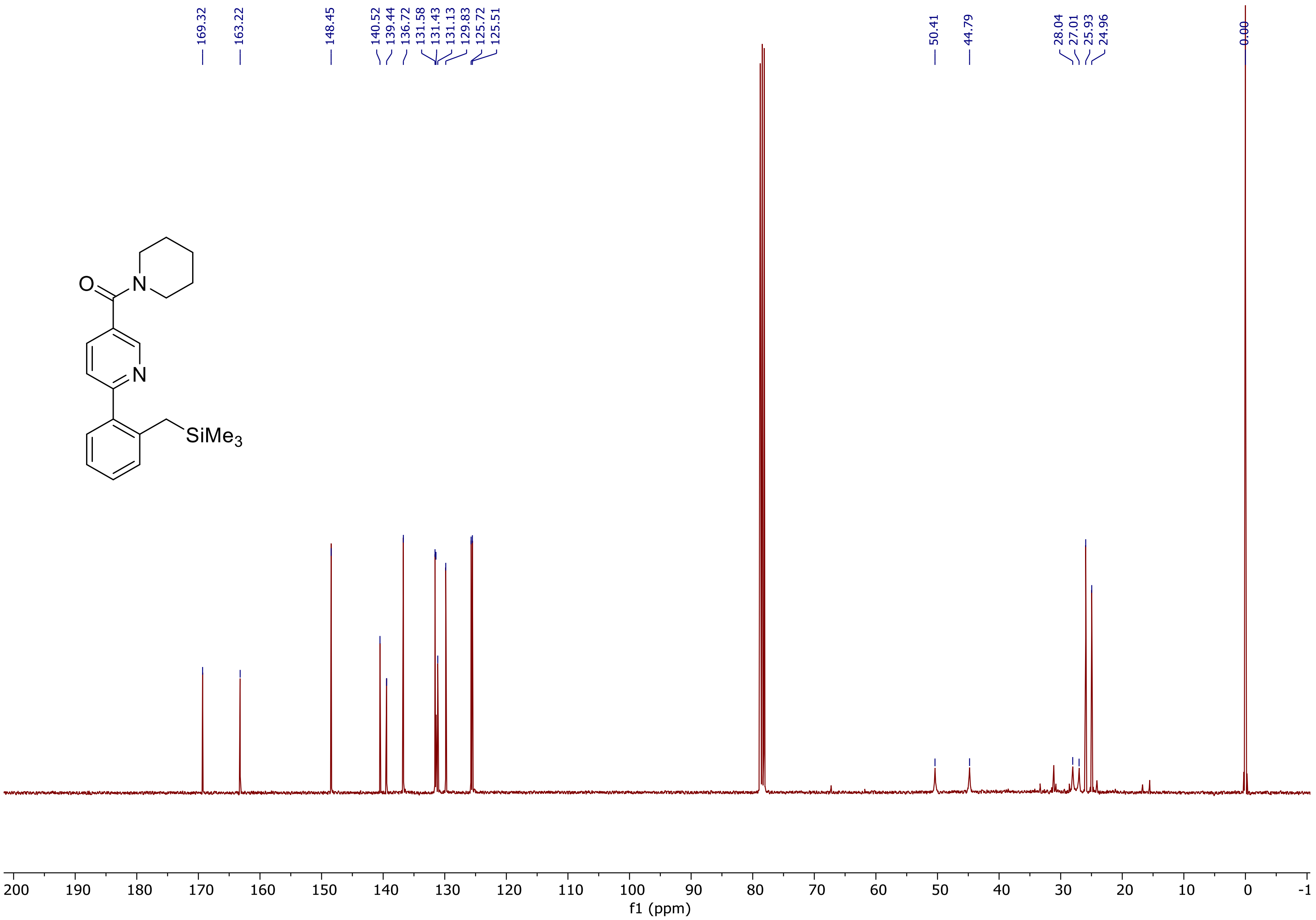

Supplementary Figure 144. ^{13}C NMR (101 MHz, $CDCl_3$) of piperidin-1-yl{6-[2-((trimethylsilyl)methyl)phenyl]pyridin-3-yl}methanone **4m**.

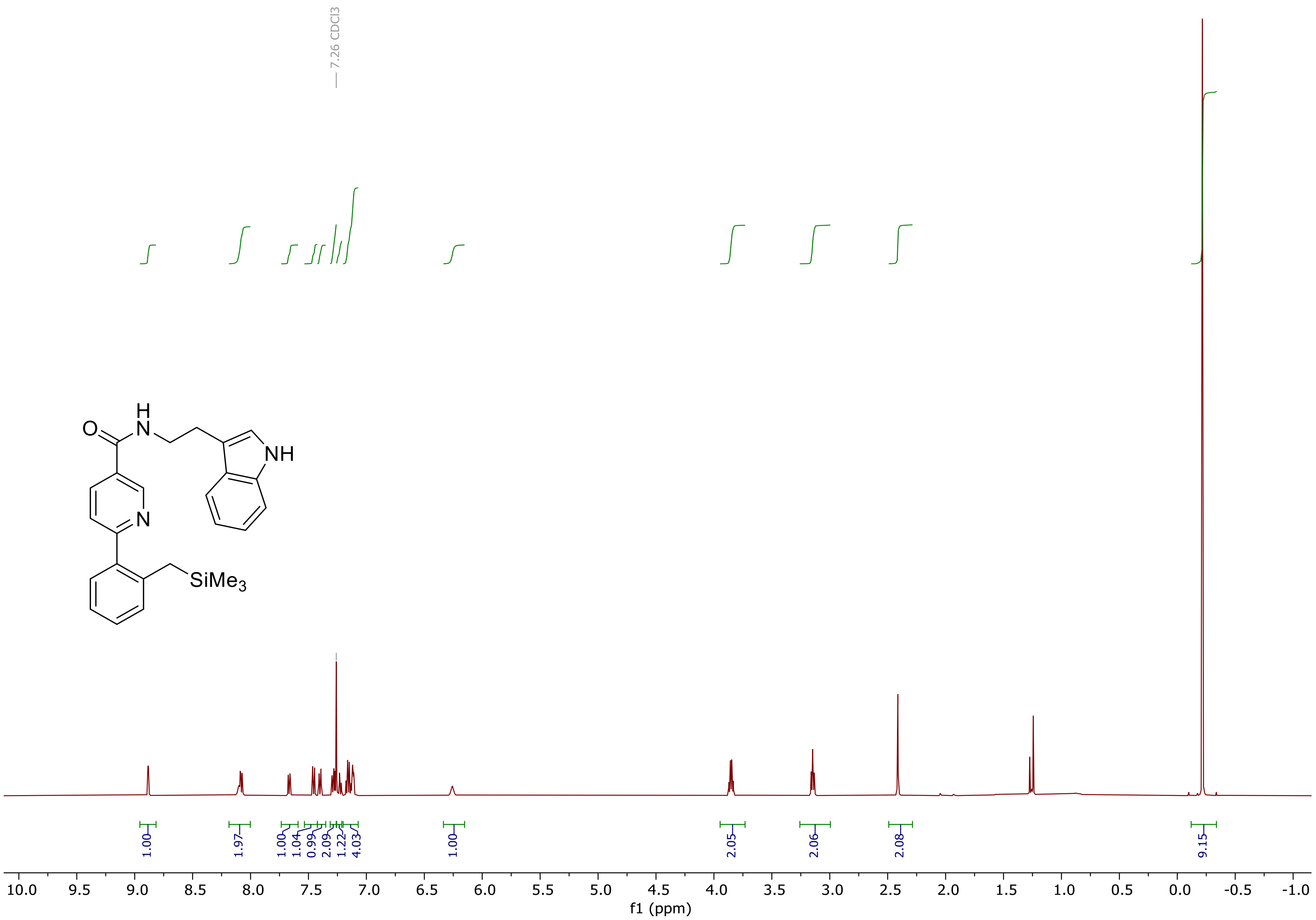

Supplementary Figure 145. ^{1}H NMR (400 MHz, $CDCl_3$) of *N*-[2-(*1H*-indol-3-yl)ethyl]-6-{2-[(trimethylsilyl)methyl]phenyl}nicotinamide **4n**.

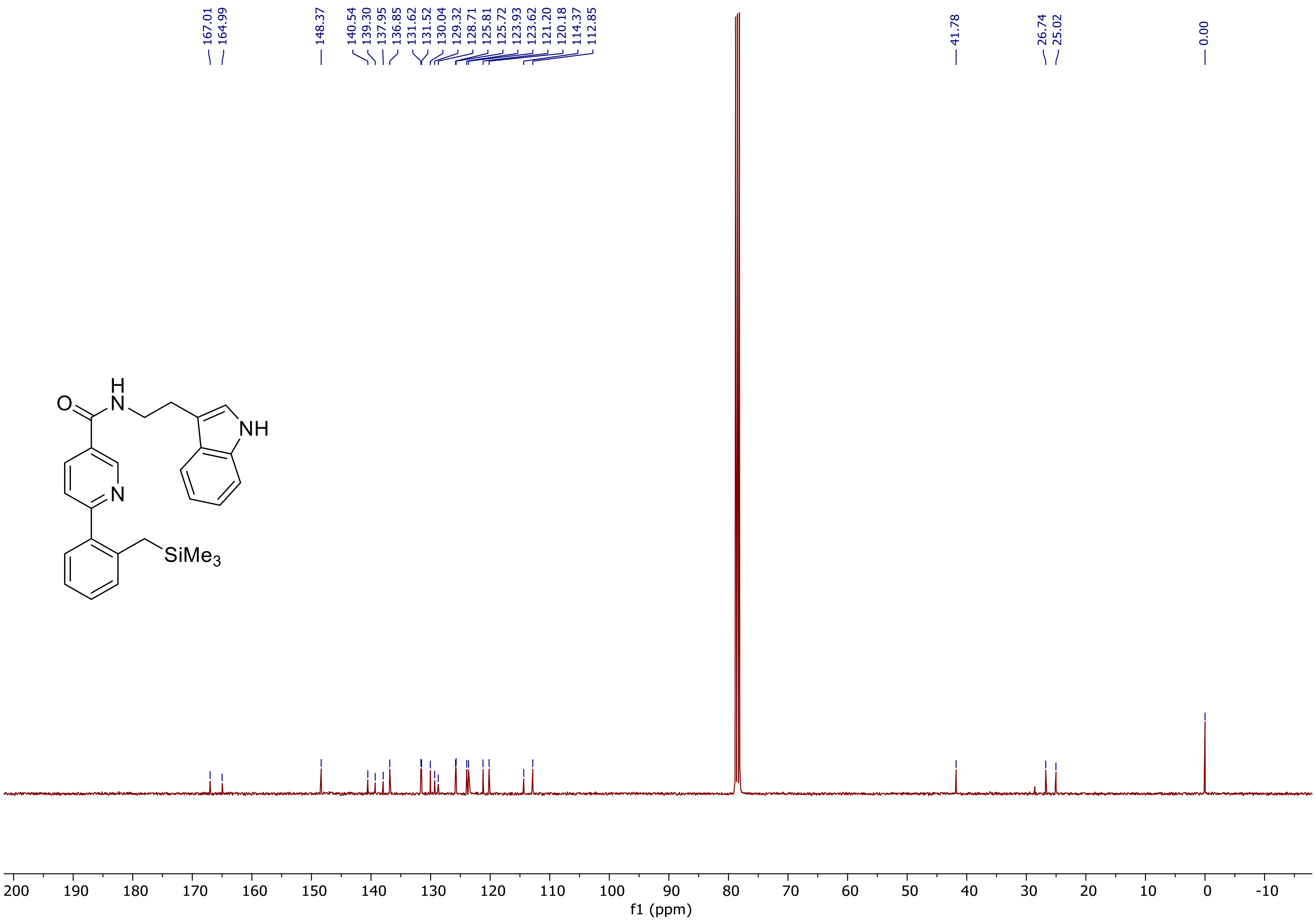

Supplementary Figure 146. ^{13}C NMR (101 MHz, $CDCl_3$) of *N*-[2-(*1H*-indol-3-yl)ethyl]-6-{2-[(trimethylsilyl)methyl]phenyl}nicotinamide **4n**.

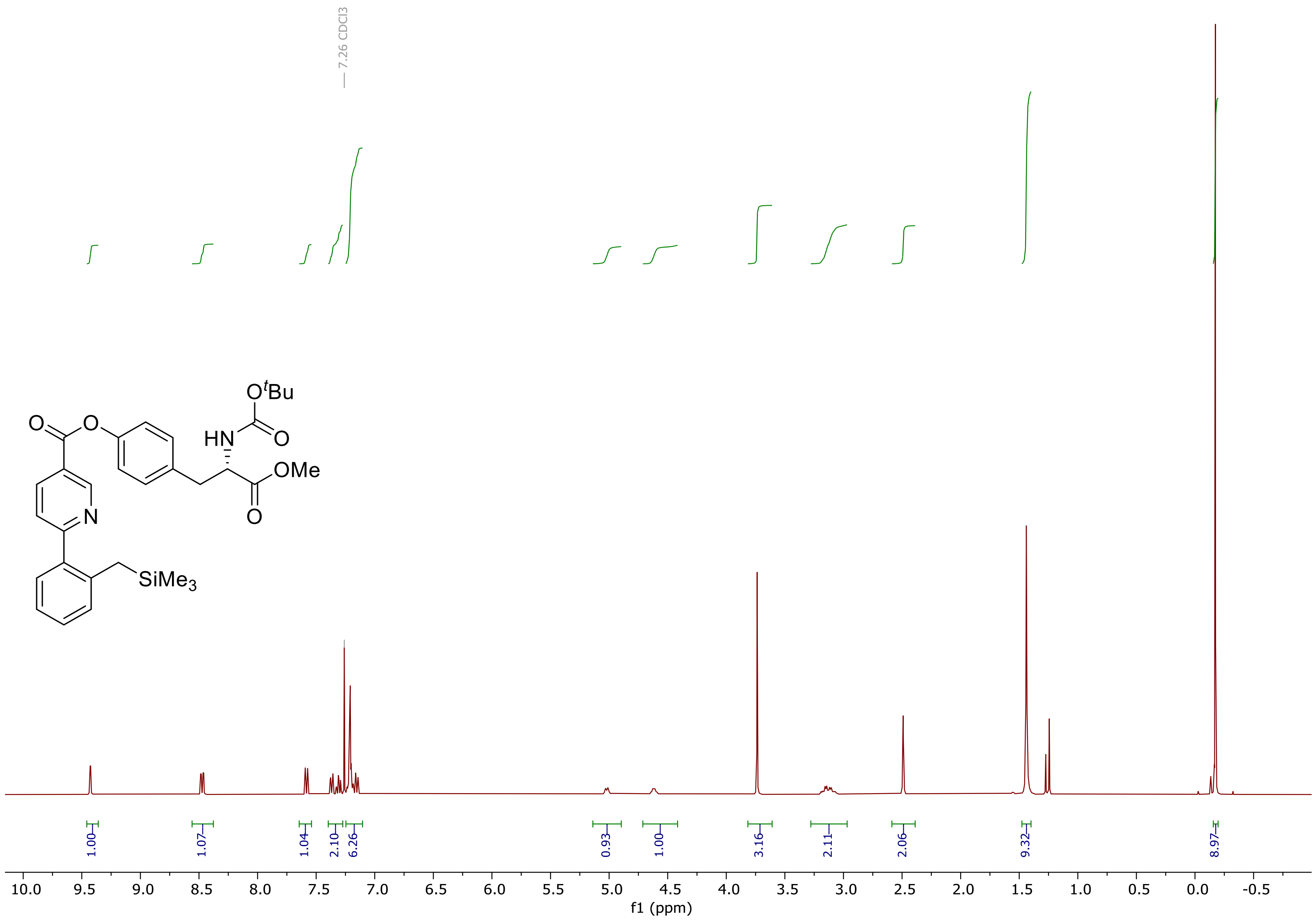

Supplementary Figure 147. ^{1}H NMR (400 MHz, $CDCl_3$) of (*S*)-4-{2-[(*tert*-butoxycarbonyl)amino]-3-methoxy-3-oxopropyl}phenyl 6-{2-[(trimethylsilyl)methyl]phenyl}nicotinate **4o**.

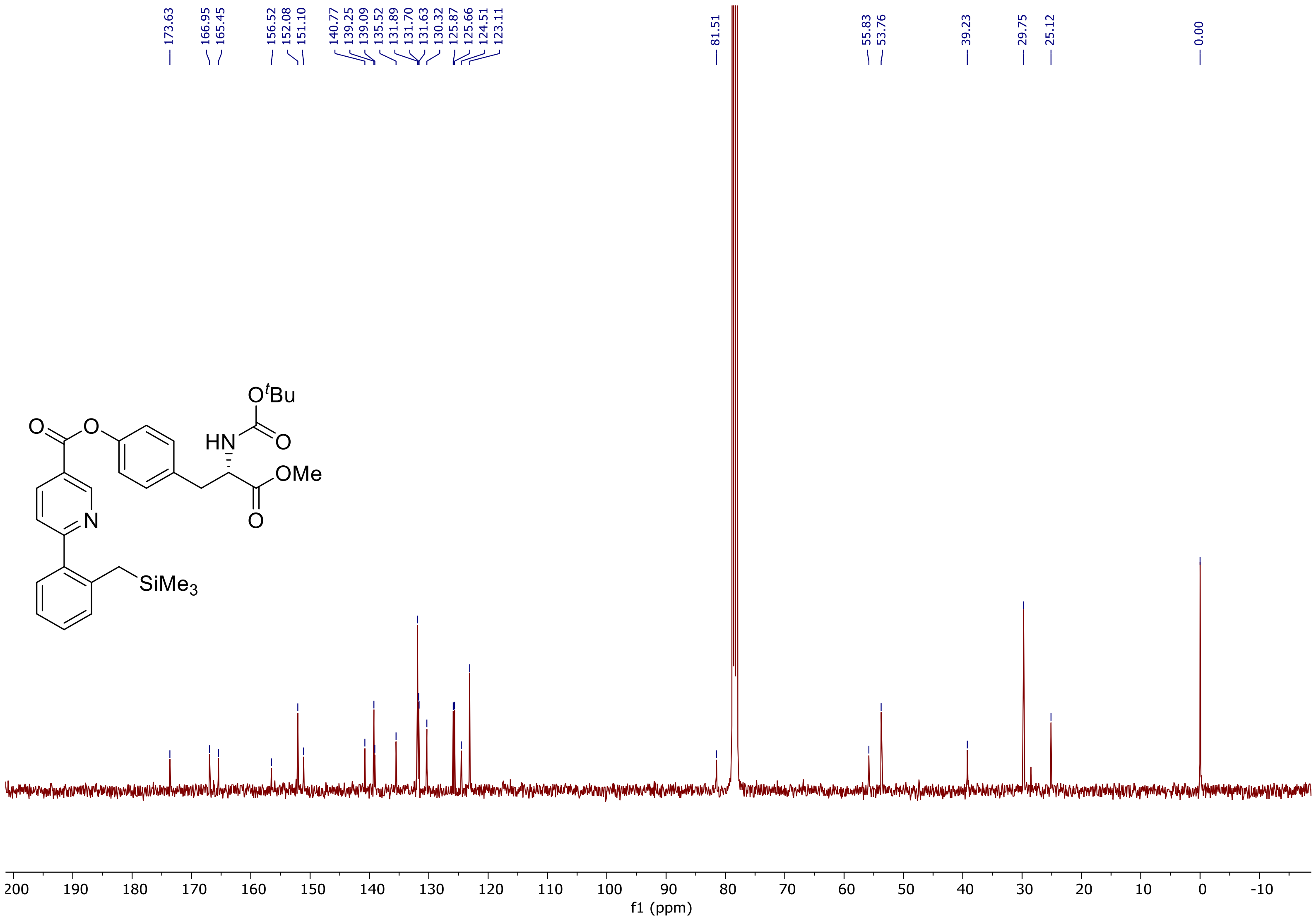

Supplementary Figure 148. ^{13}C NMR (101 MHz, $CDCl_3$) of (*S*)-4-{2-[(*t*ert-butoxycarbonyl)amino]-3-methoxy-3-oxopropyl}phenyl 6-{2-[(trimethylsilyl)methyl]phenyl}nicotinate.

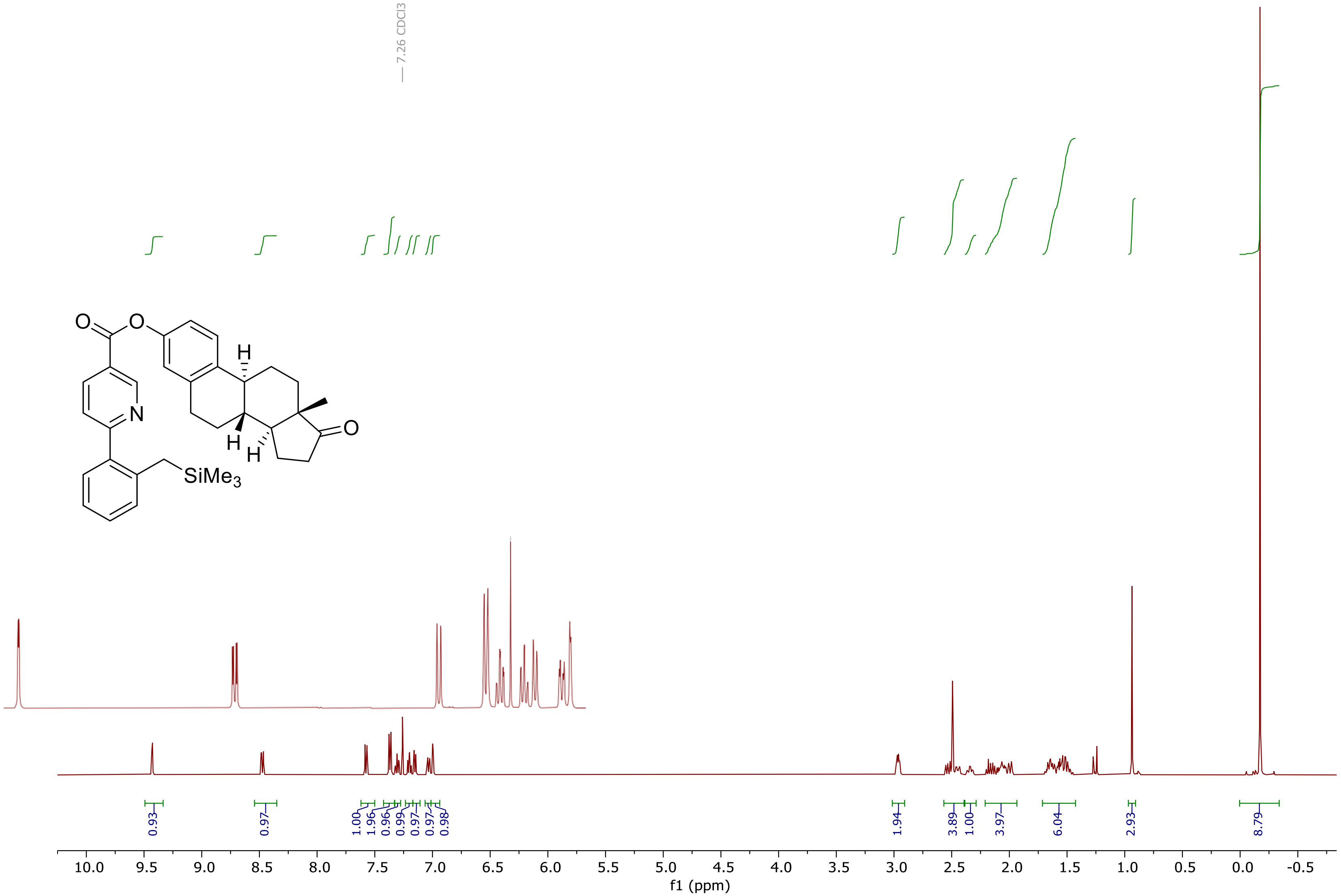

Supplementary Figure 149. ^{1}H NMR (500 MHz, $CDCl_3$) of (8R,9S,13S,14S)-13-methyl-17-oxo-7,8,9,11,12,13,14,15,16,17-decahydro-*6H*-cyclopenta[a]phenanthren-3-yl 6-{2-[(trimethylsilyl)methyl]phenyl}nicotinate **4p**.

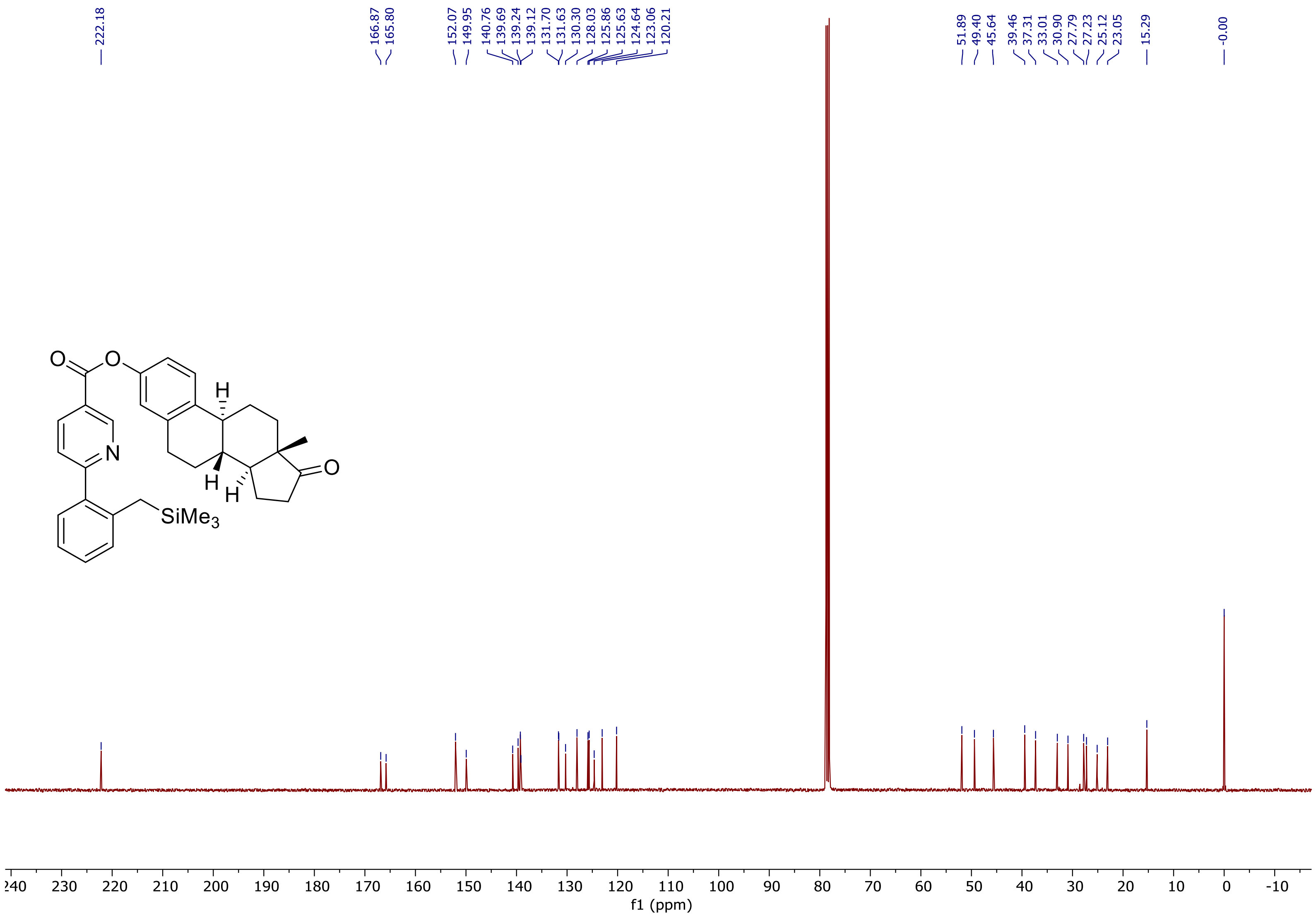

Supplementary Figure 150. ^{13}C NMR (101 MHz, $CDCl_3$) of (8R,9S,13S,14S)-13-methyl-17-oxo-7,8,9,11,12,13,14,15,16,17-decahydro-*6H*-cyclopenta[a]phenanthren-3-yl 6-{2-[(trimethylsilyl)methyl]phenyl}nicotinate **4p**.

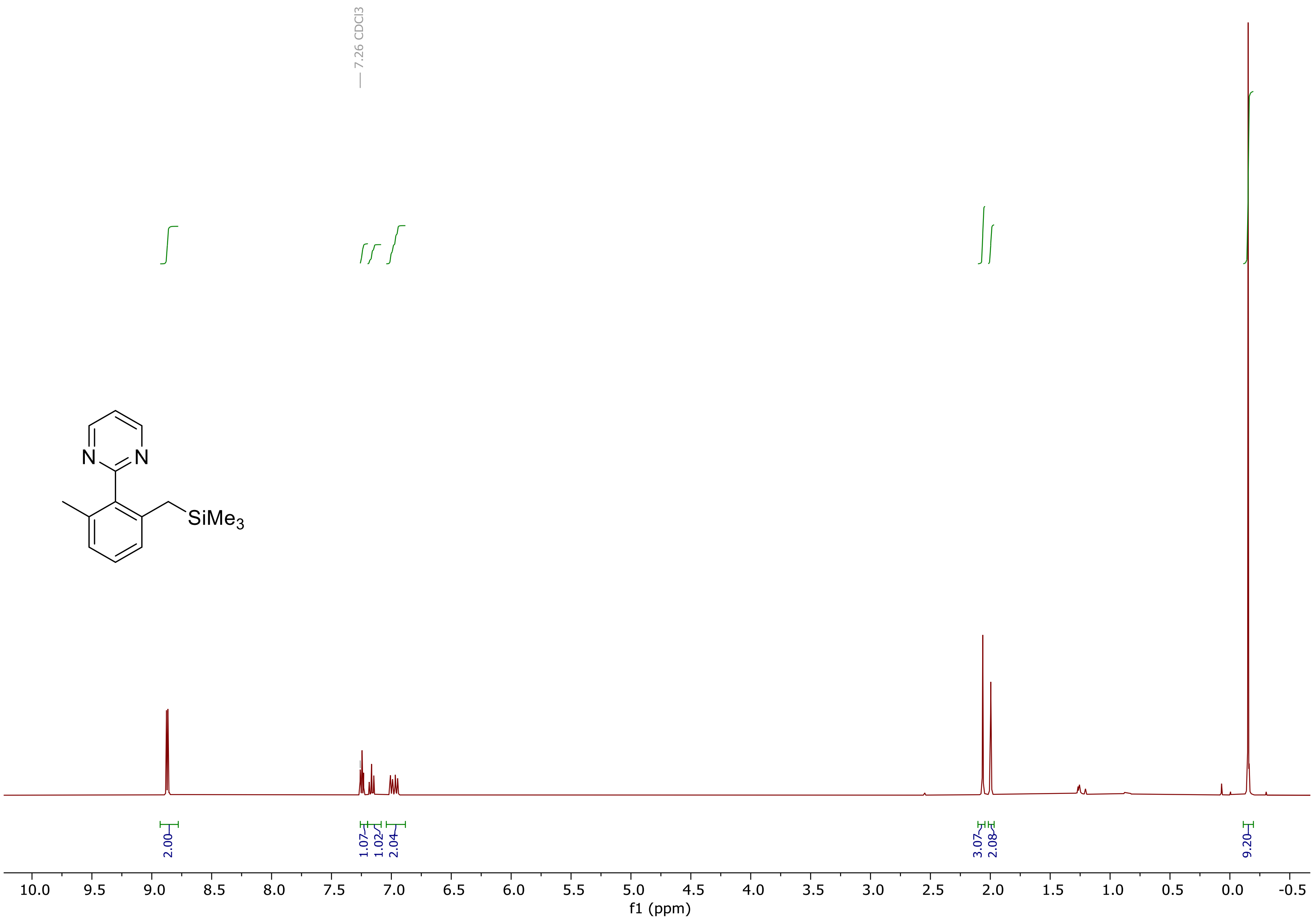

Supplementary Figure 151. ^{1}H NMR (400 MHz, $CDCl_3$) of 2-{2-methyl-6-[(trimethylsilyl)methyl]phenyl}pyrimidine **4q**.

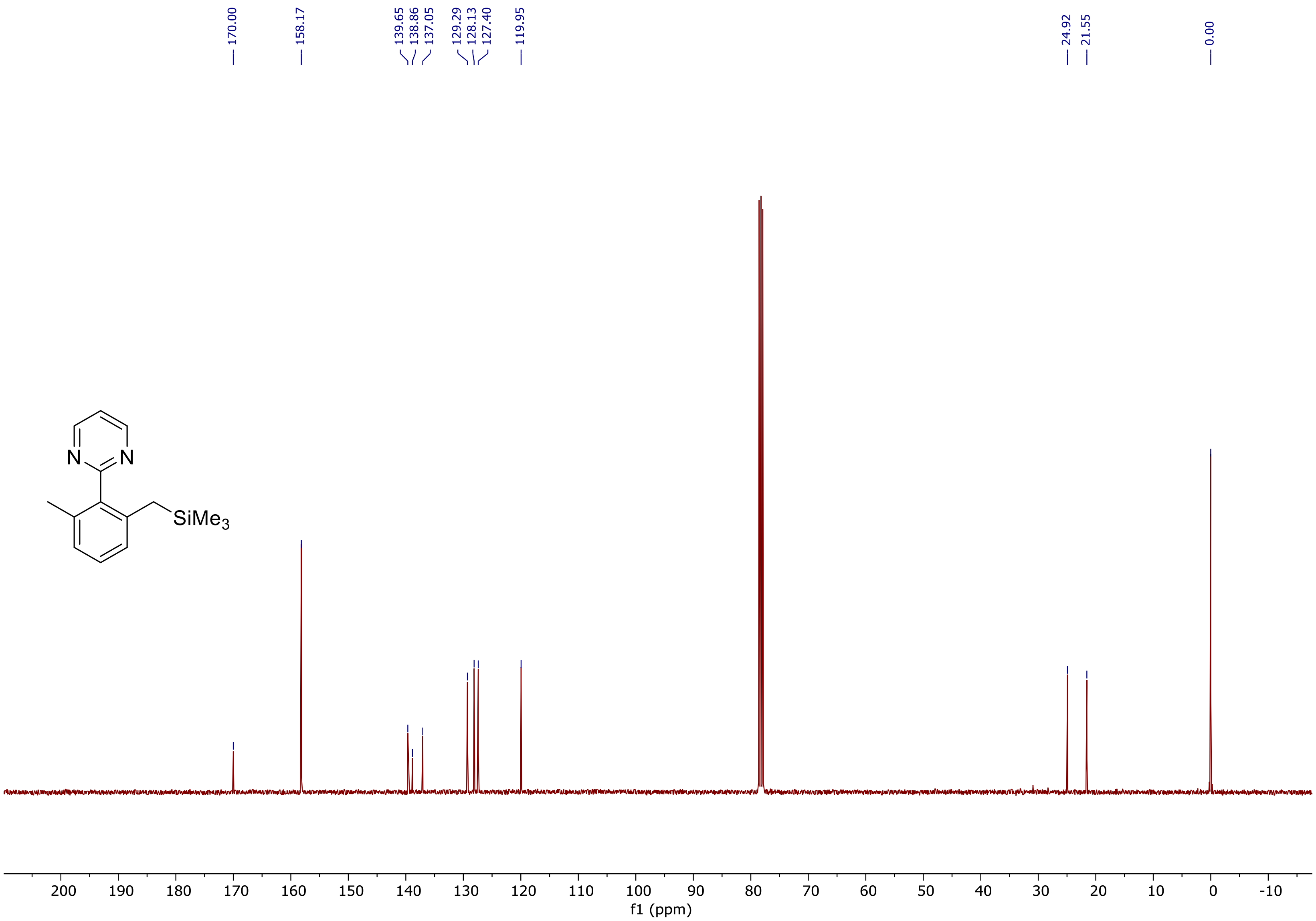

Supplementary Figure 152. ^{13}C NMR (101 MHz, $CDCl_3$) of 2-{2-methyl-6-[(trimethylsilyl)methyl]phenyl}pyrimidine **4q**.

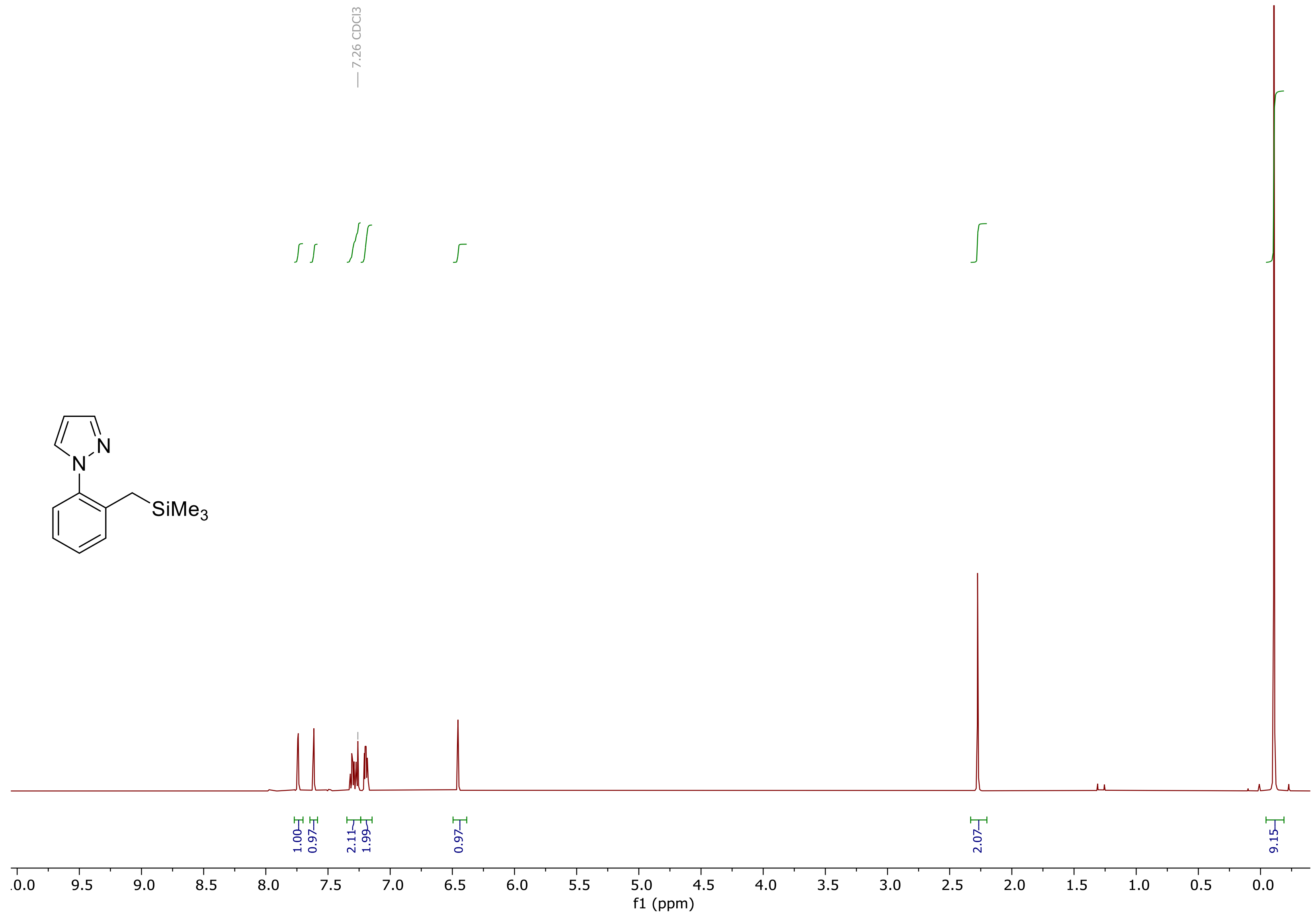

Supplementary Figure 153. ^{1}H NMR (500 MHz, $CDCl_3$) of 1-{2-[trimethylsilyl)methyl]phenyl}-*1H*-pyrazole **4r**.

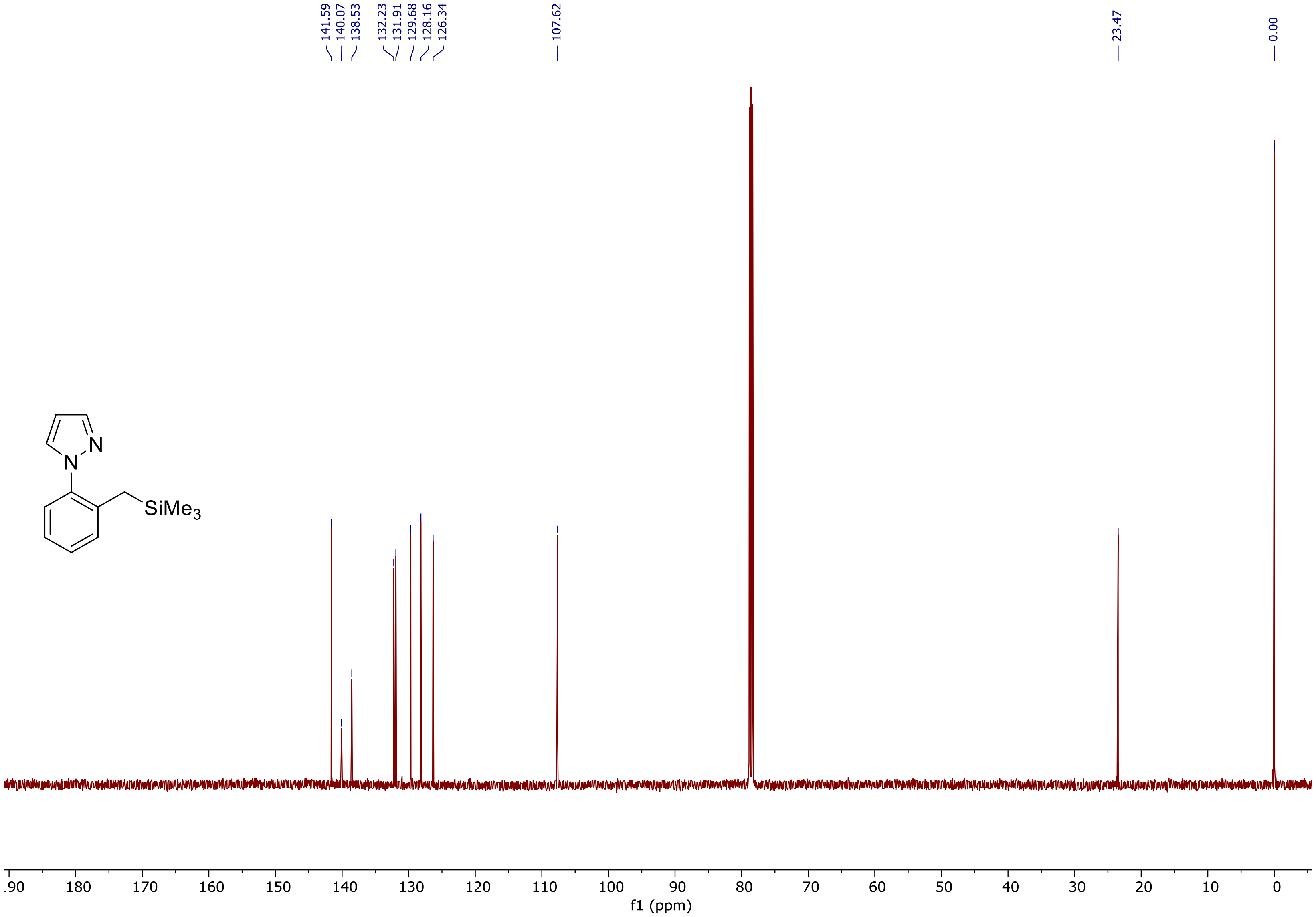

Supplementary Figure 154. ^{13}C NMR (126 MHz, $CDCl_3$) of 1-{2-[trimethylsilyl)methyl]phenyl}-*1H*-pyrazole **4r**.

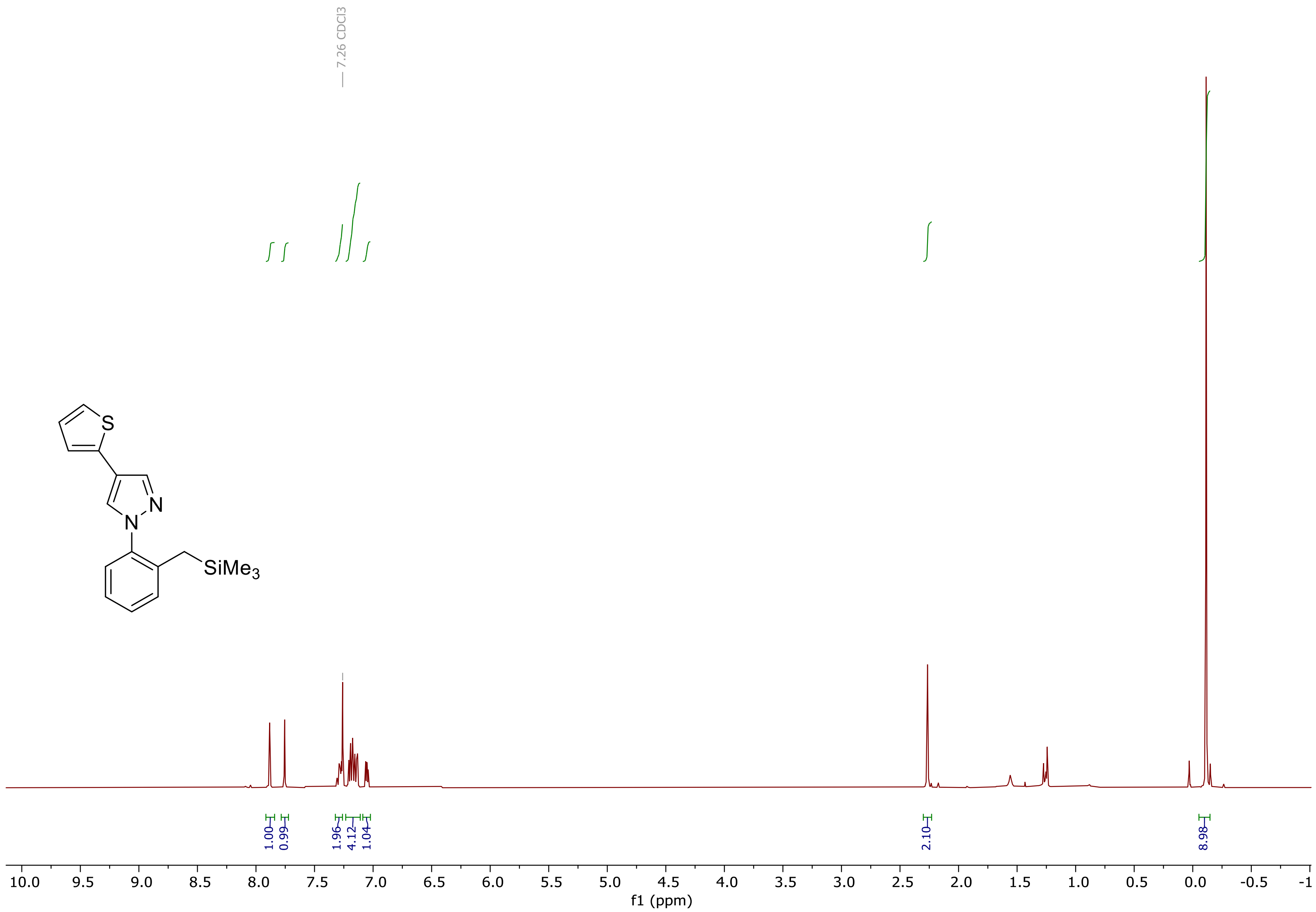

Supplementary Figure 155. ^{1}H NMR (400 MHz, $CDCl_3$) of 4-(thiophen-2-yl)-1-{2-[(trimethylsilyl)methyl]phenyl}-*1H*-pyrazole **4s**.

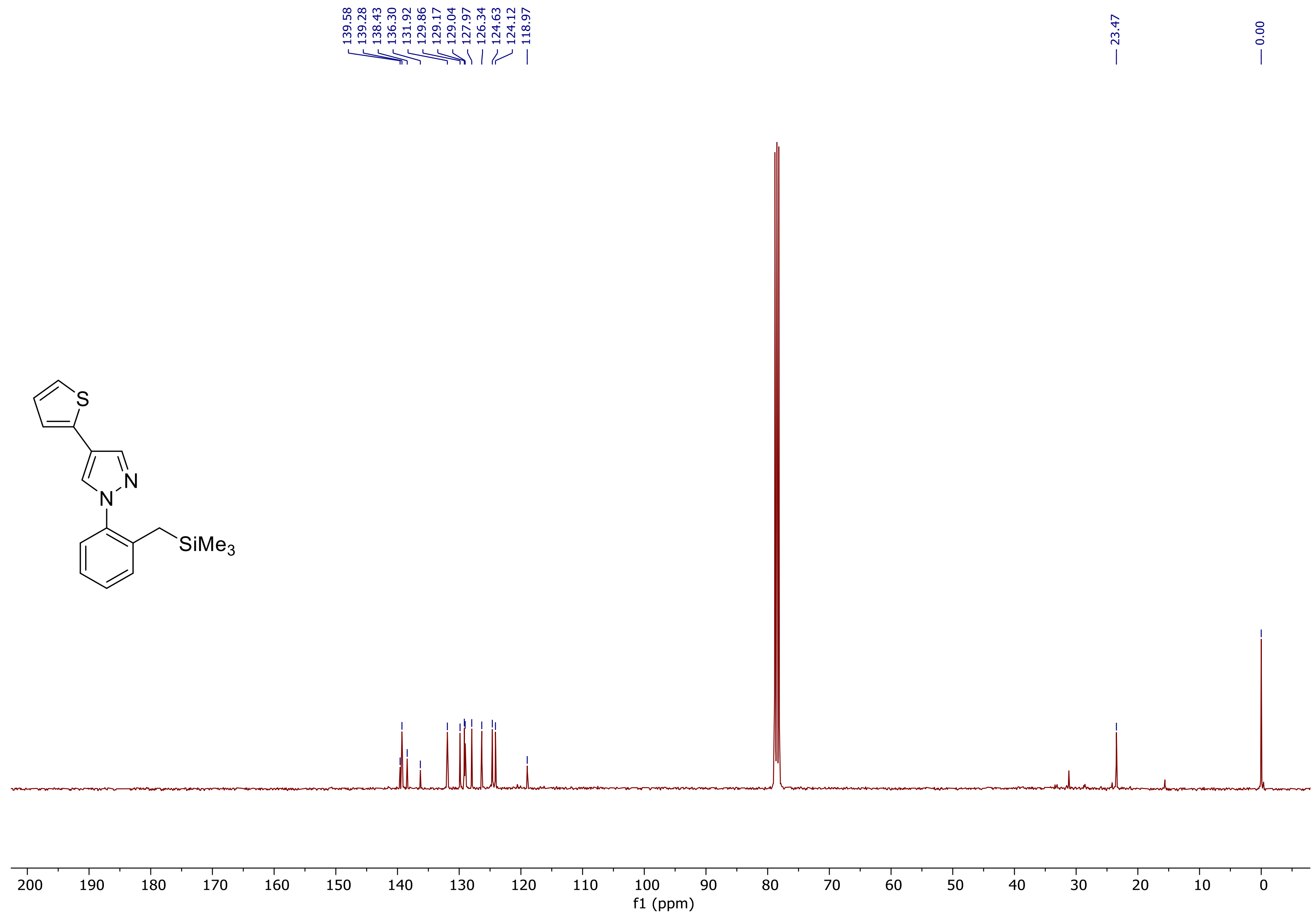

Supplementary Figure 156. ^{13}C NMR (101 MHz, $CDCl_3$) of 4-(thiophen-2-yl)-1-{2-[(trimethylsilyl)methyl]phenyl}-*1H*-pyrazole **4s**.

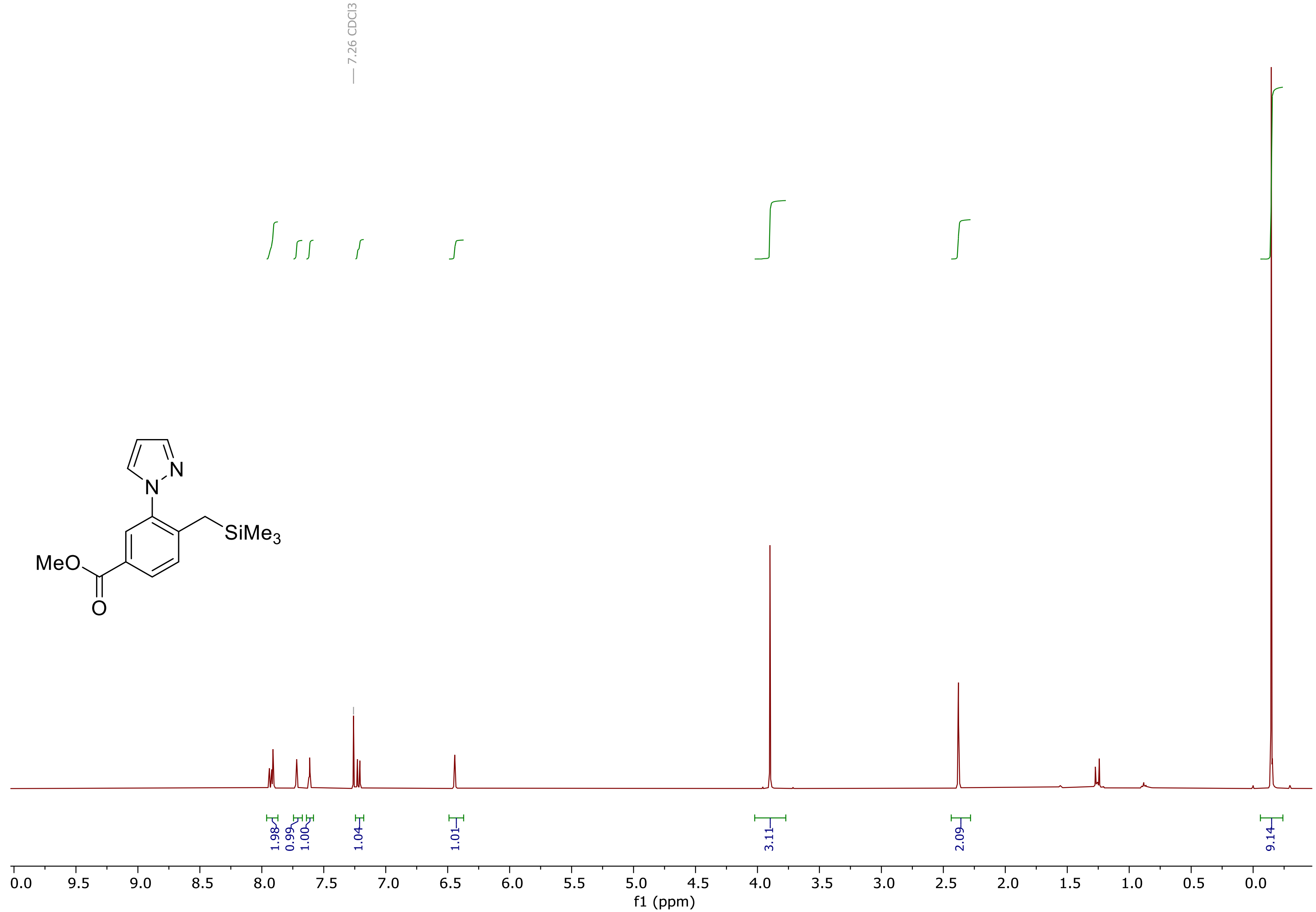

Supplementary Figure 157. ^{1}H NMR (400 MHz, $CDCl_3$) of methyl 3-(*1H*-pyrazol-1-yl)-4-[(trimethylsilyl)methyl]benzoate **4t**.

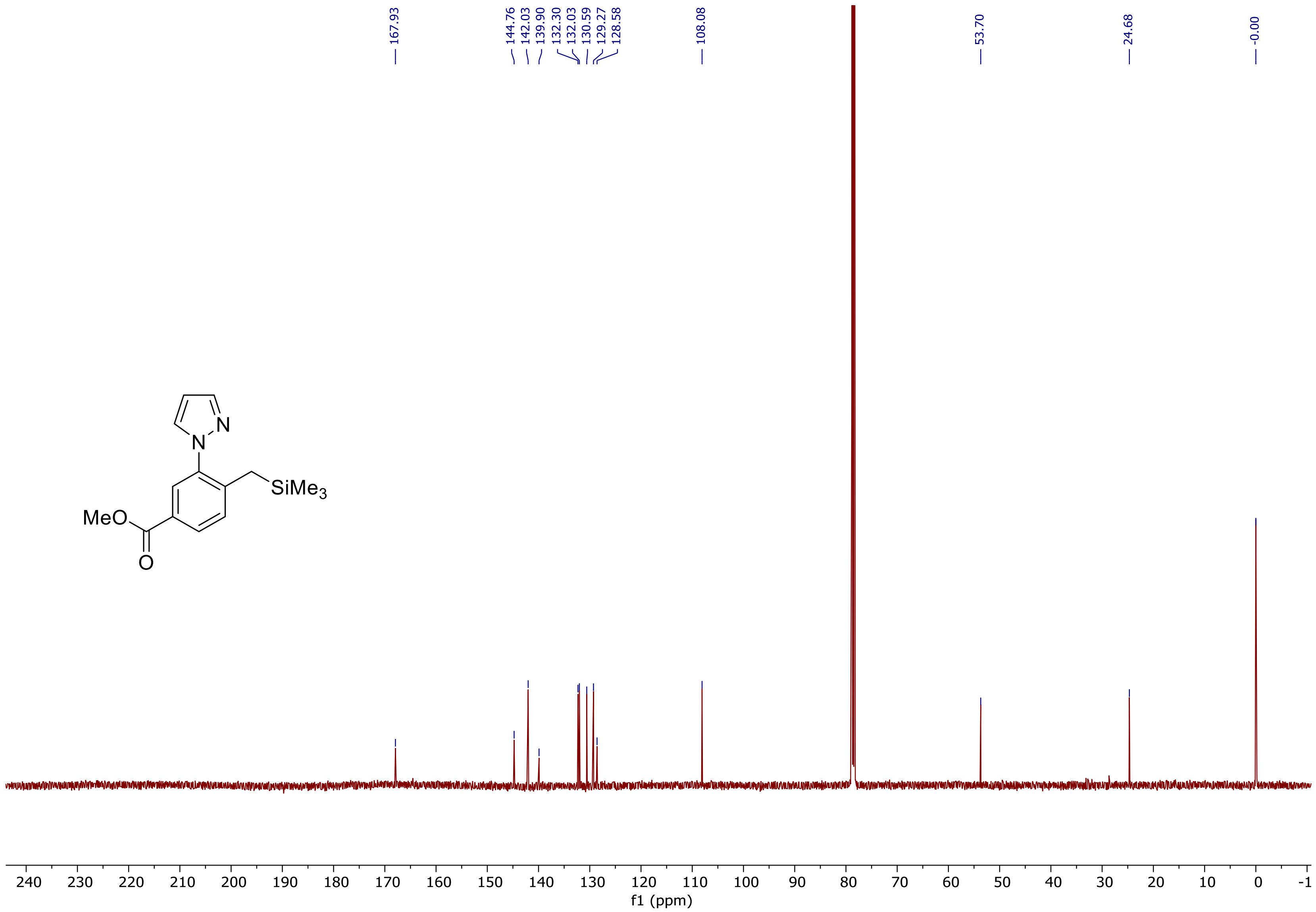

Supplementary Figure 158. ^{13}C NMR (126 MHz, $CDCl_3$) of methyl 3-(*1H*-pyrazol-1-yl)-4-[(trimethylsilyl)methyl]benzoate **4t**.

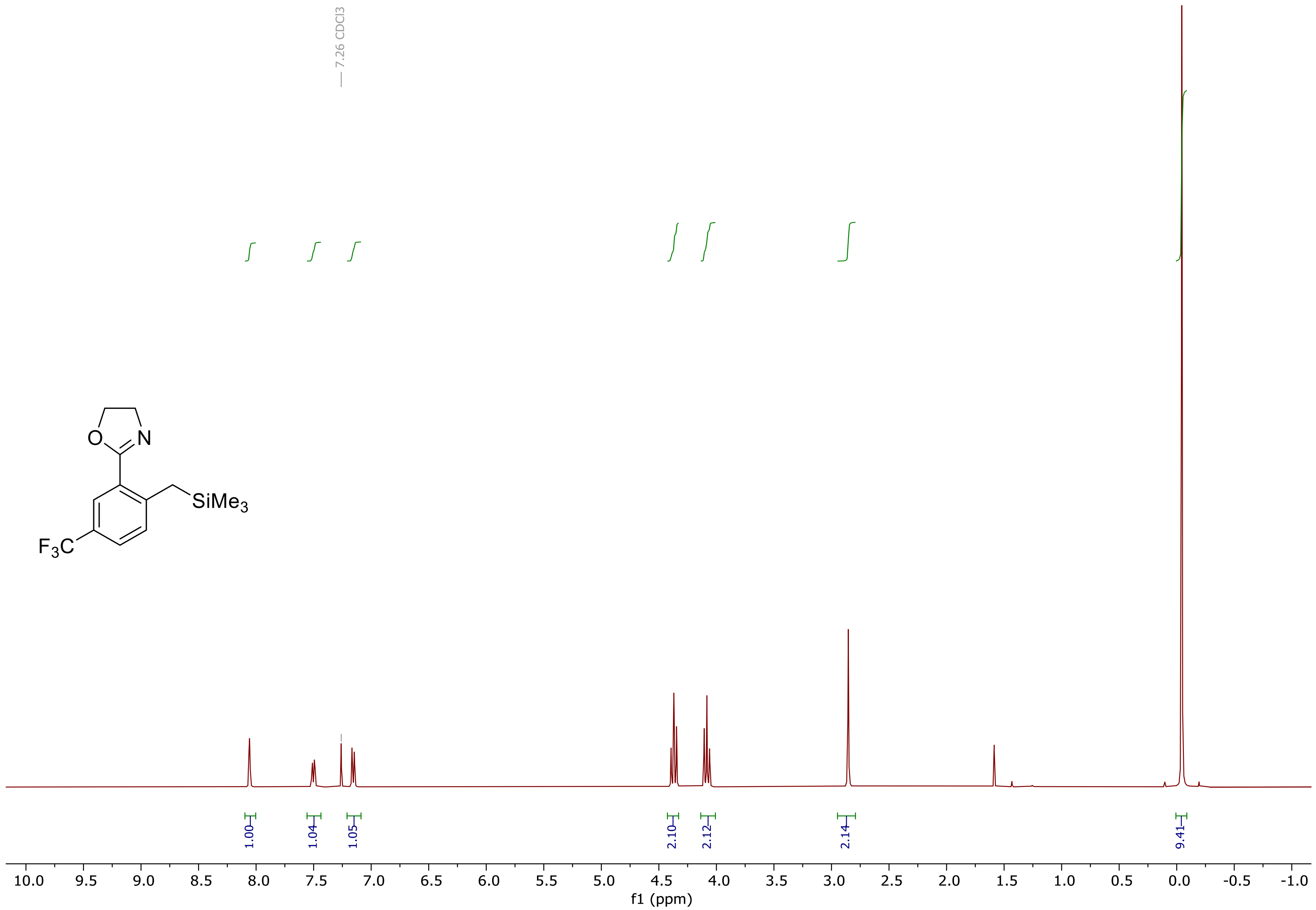

Supplementary Figure 159. ^{1}H NMR (400 MHz, $CDCl_3$) of 2-{5-(trifluoromethyl)-2-[(trimethylsilyl)methyl]phenyl}-4,5-dihydrooxazole **4u**.

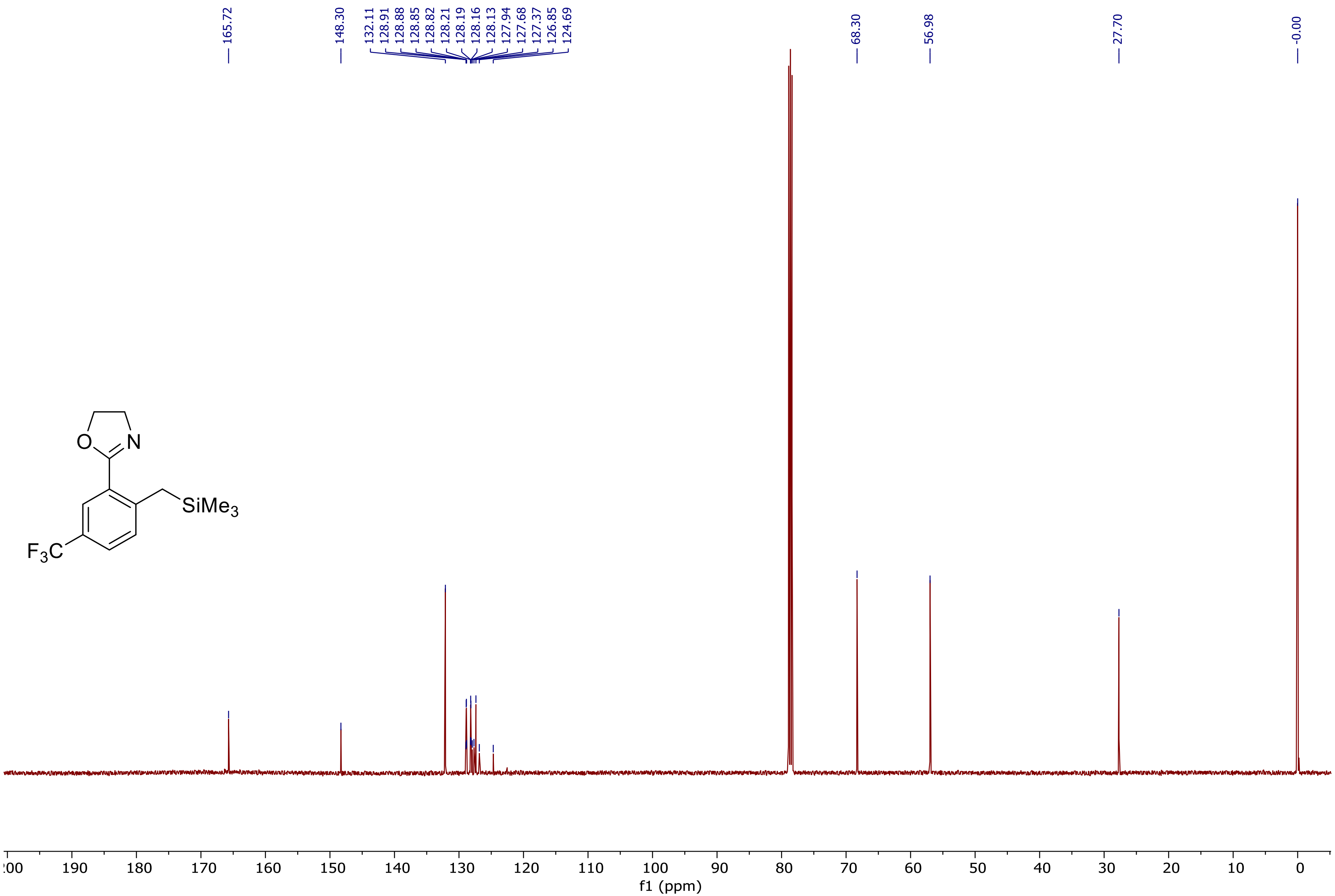

Supplementary Figure 160. ^{13}C NMR (126 MHz, $CDCl_3$) of 2-{5-(trifluoromethyl)-2-[(trimethylsilyl)methyl]phenyl}-4,5-dihydrooxazole **4u**.

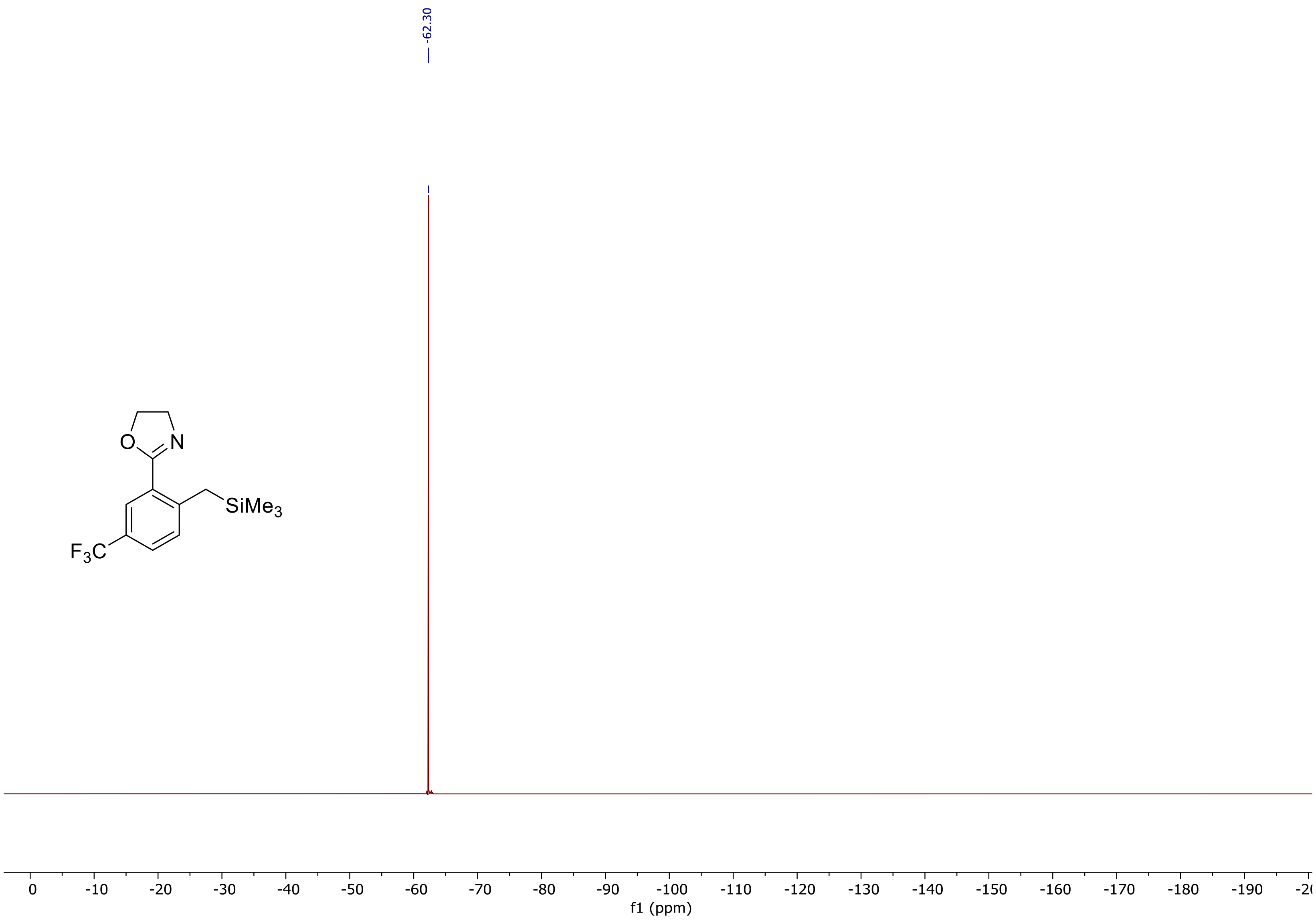

Supplementary Figure 161. ^{19}F NMR (376 MHz, $CDCl_3$) of 2-{5-(trifluoromethyl)-2-[(trimethylsilyl)methyl]phenyl}-4,5-dihydrooxazole **4u**.

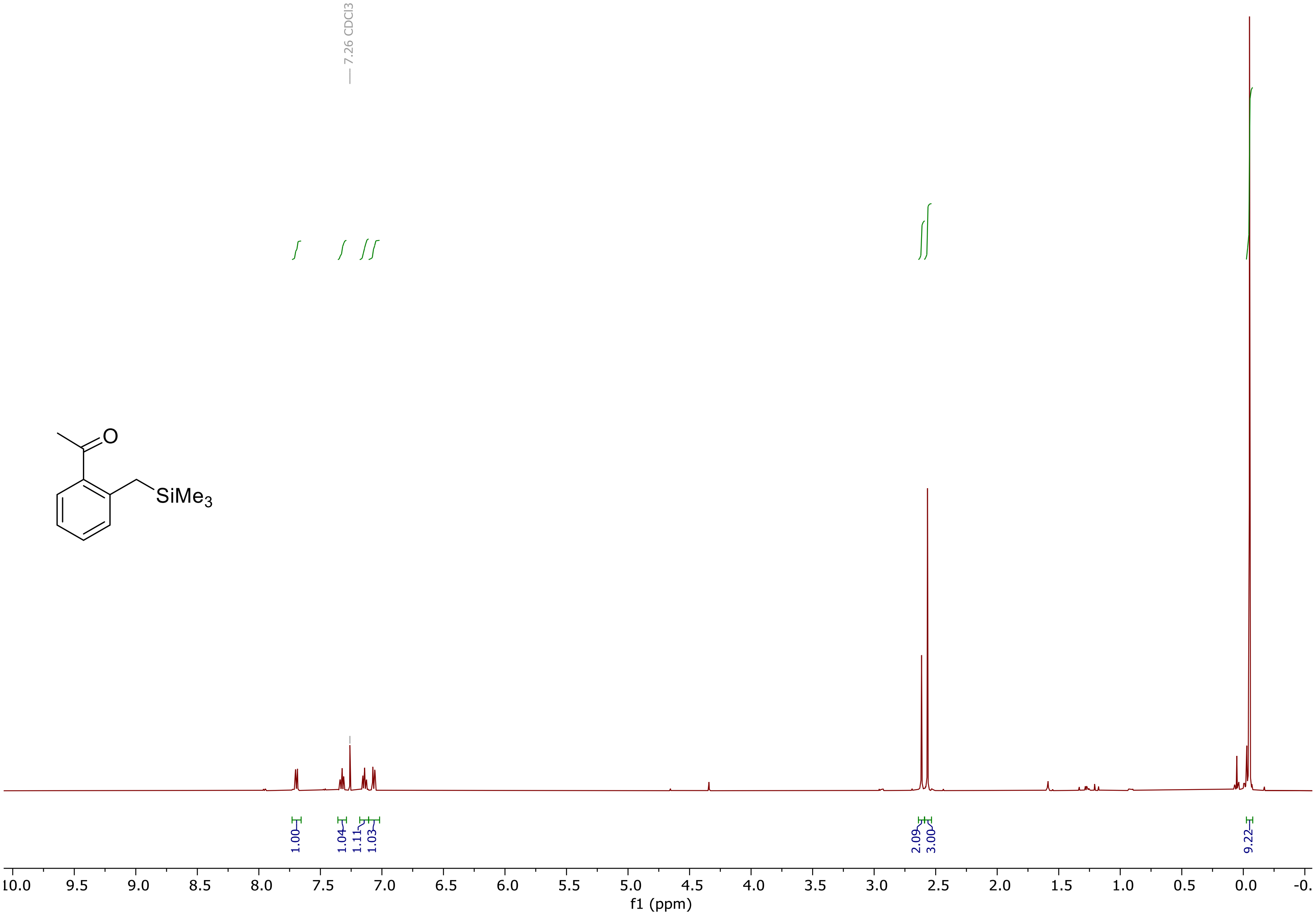

Supplementary Figure 162. ^{1}H NMR (500 MHz, $CDCl_3$) of 1-{2-[(trimethylsilyl)methyl]phenyl}ethan-1-one **4v**.

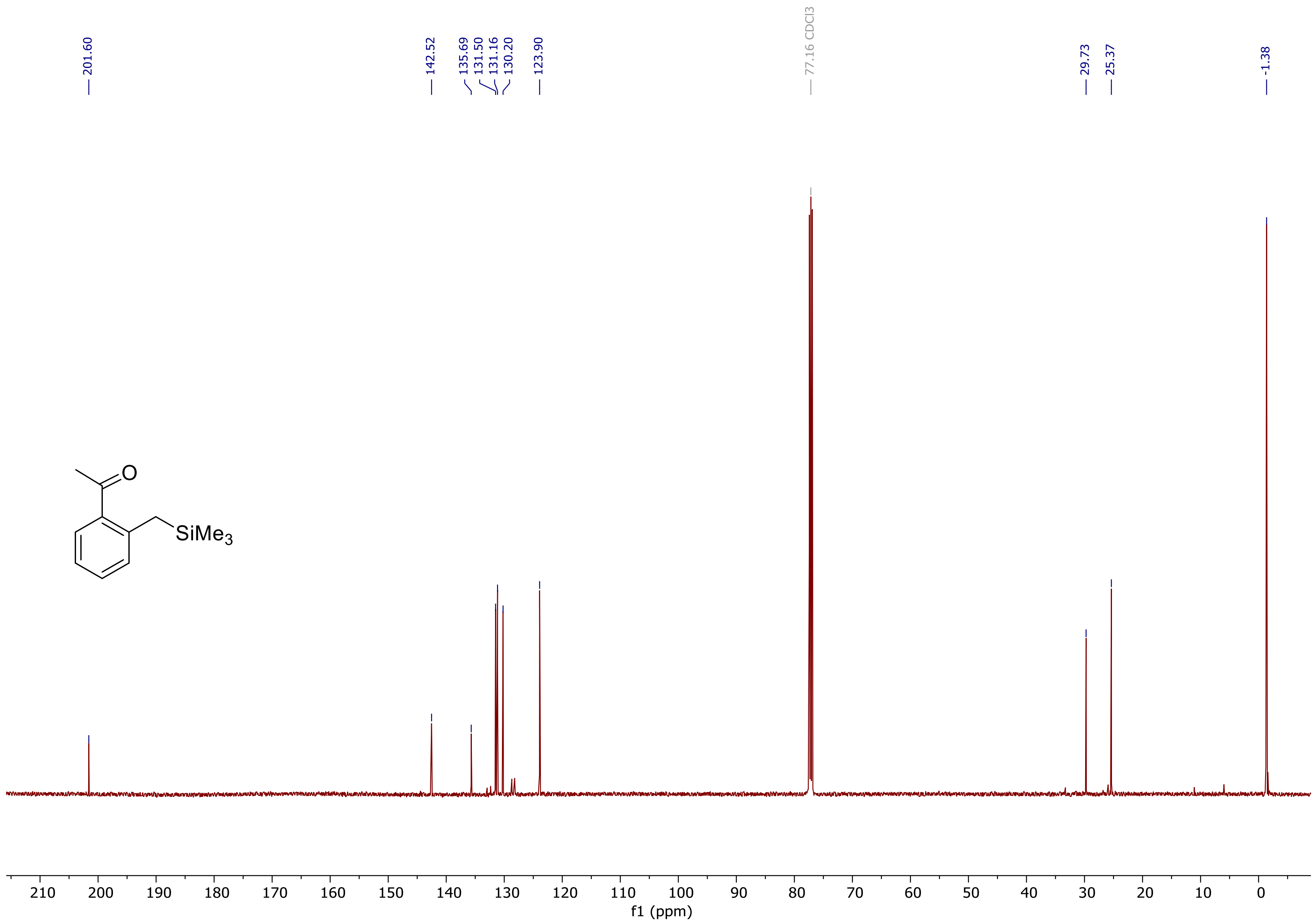

Supplementary Figure 163. ^{13}C NMR (101 MHz, $CDCl_3$) of 1-{2-[(trimethylsilyl)methyl]phenyl}ethan-1-one **4v**.

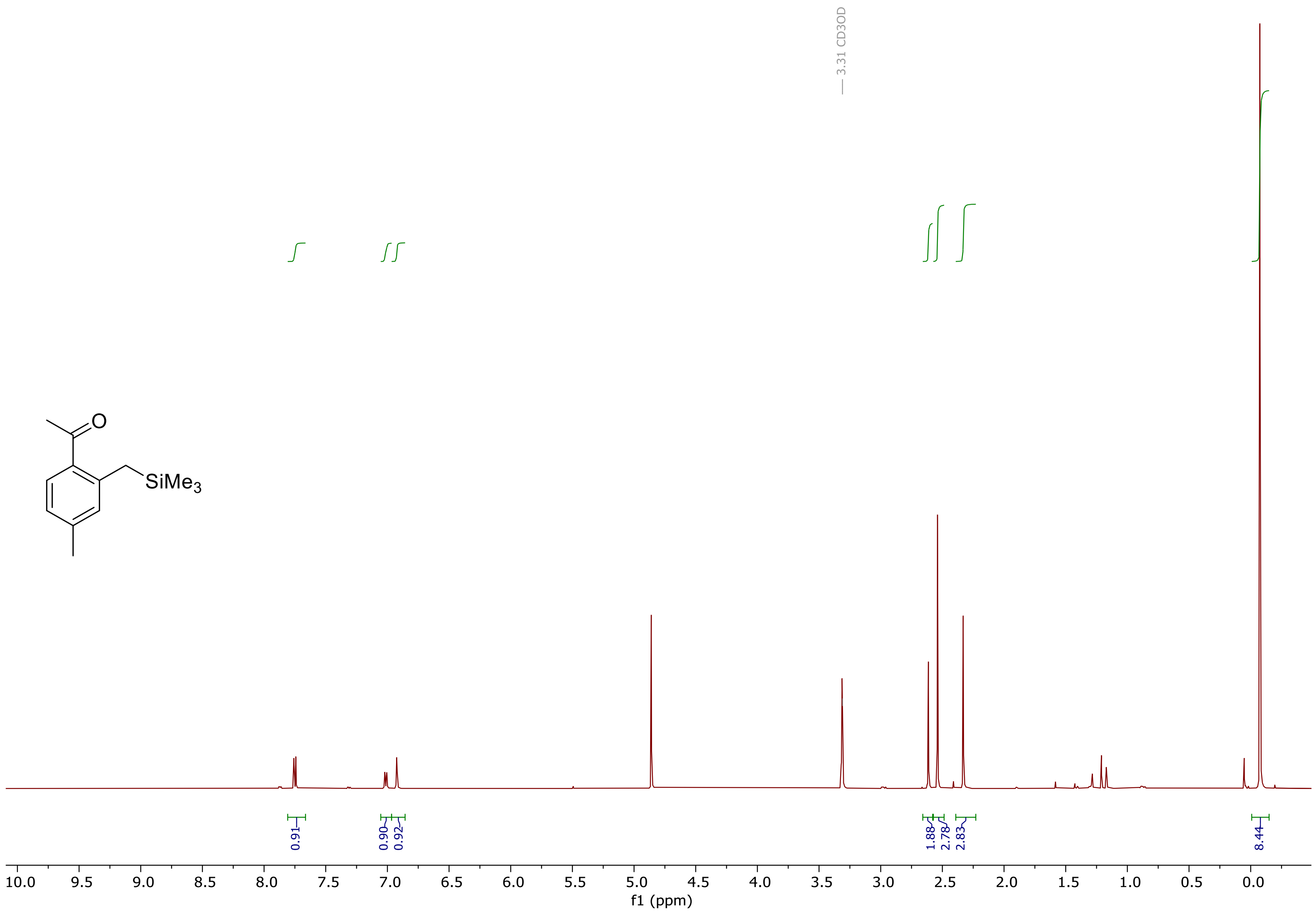

Supplementary Figure 164. ^{1}H NMR (500 MHz, d_4-CD_3OD) of 1-{4-methyl-2-[(trimethylsilyl)methyl]phenyl}ethan-1-one **4w**.

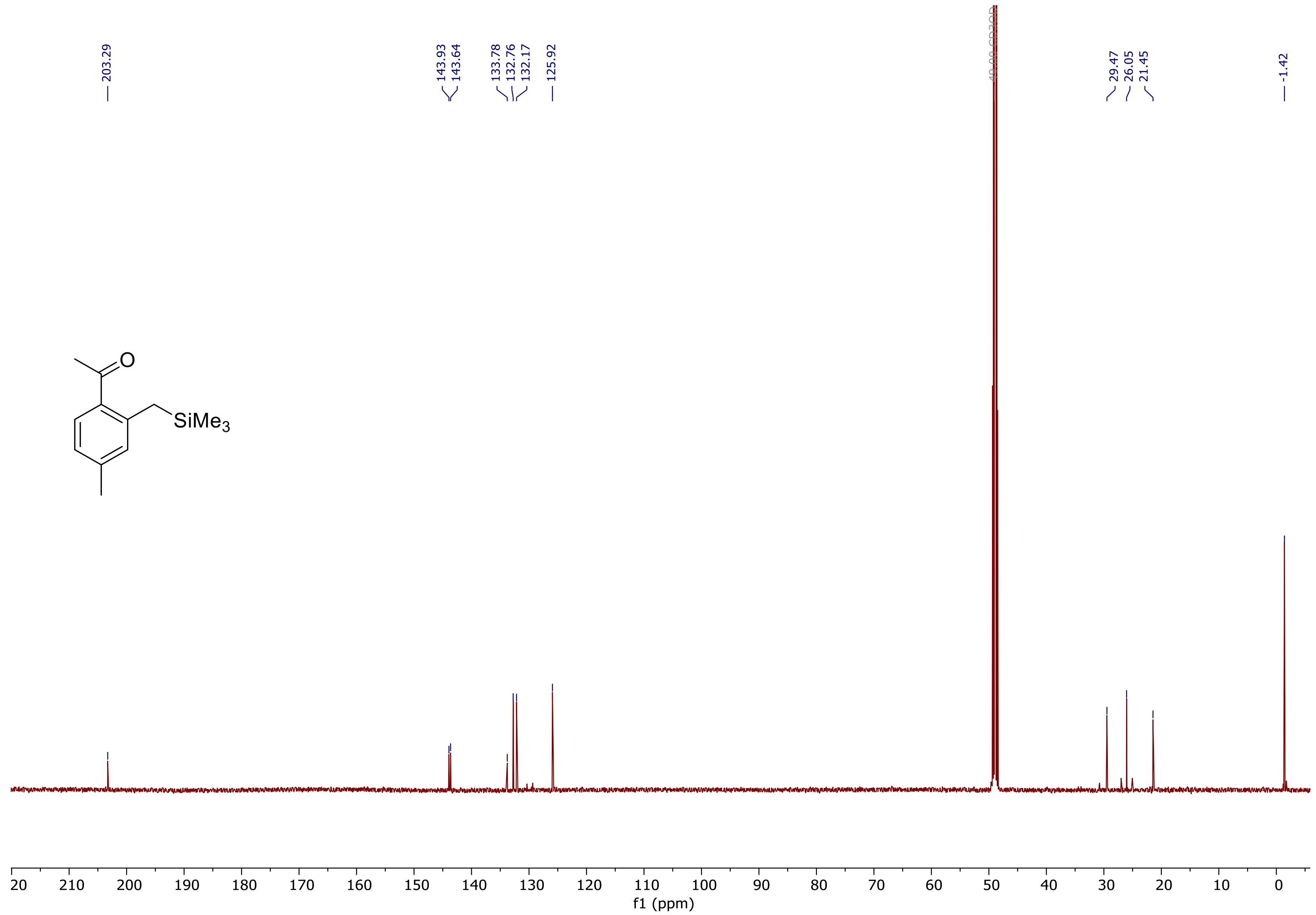

Supplementary Figure 165. ^{13}C NMR (126 MHz, d_4-CD_3OD) of 1-{4-methyl-2-[(trimethylsilyl)methyl]phenyl}ethan-1-one **4w**.

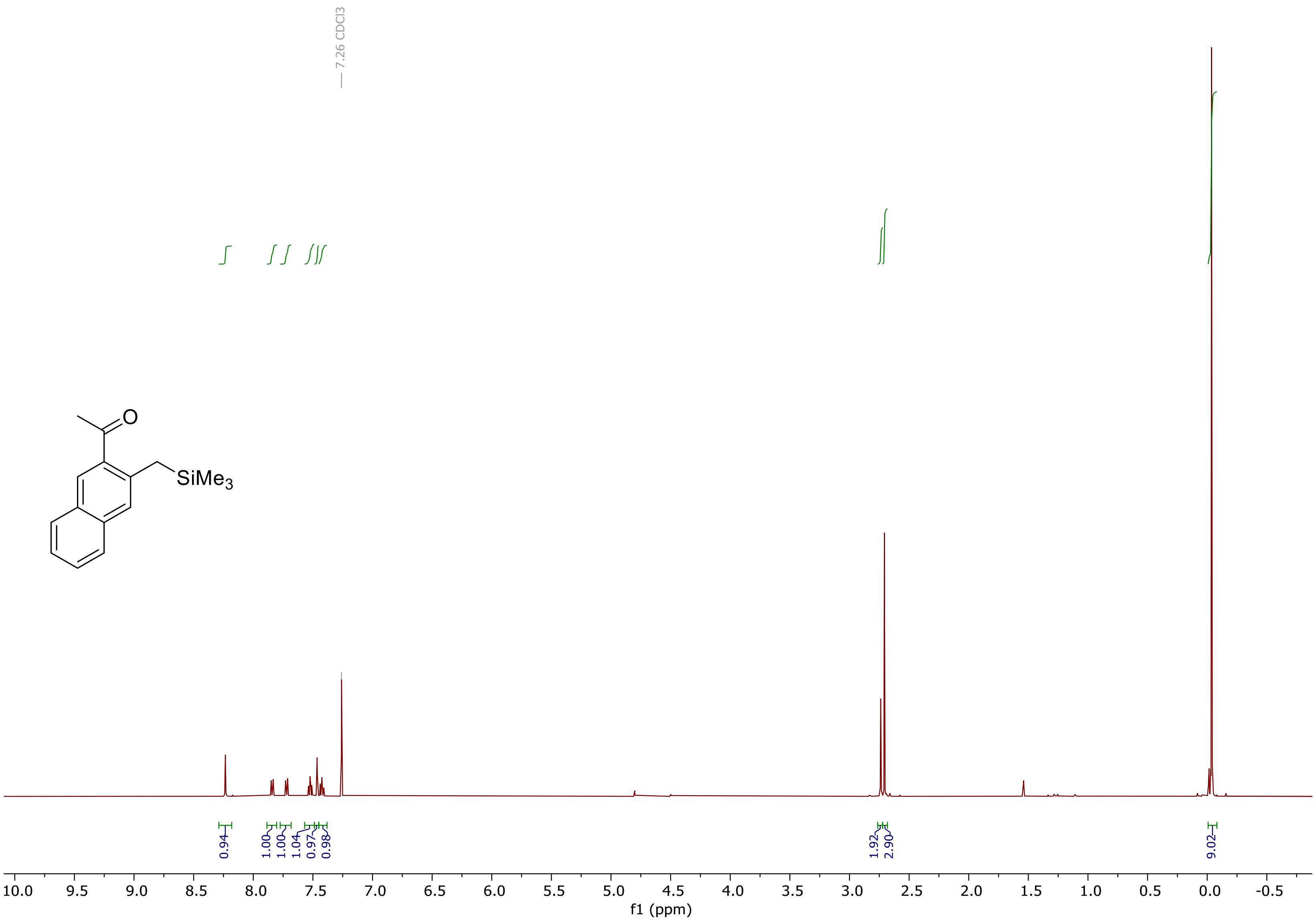

Supplementary Figure 166. ^{1}H NMR (500 MHz, $CDCl_3$) of 1-{3-[(trimethylsilyl)methyl]naphthalen-2-yl}ethan-1-one **4x**.

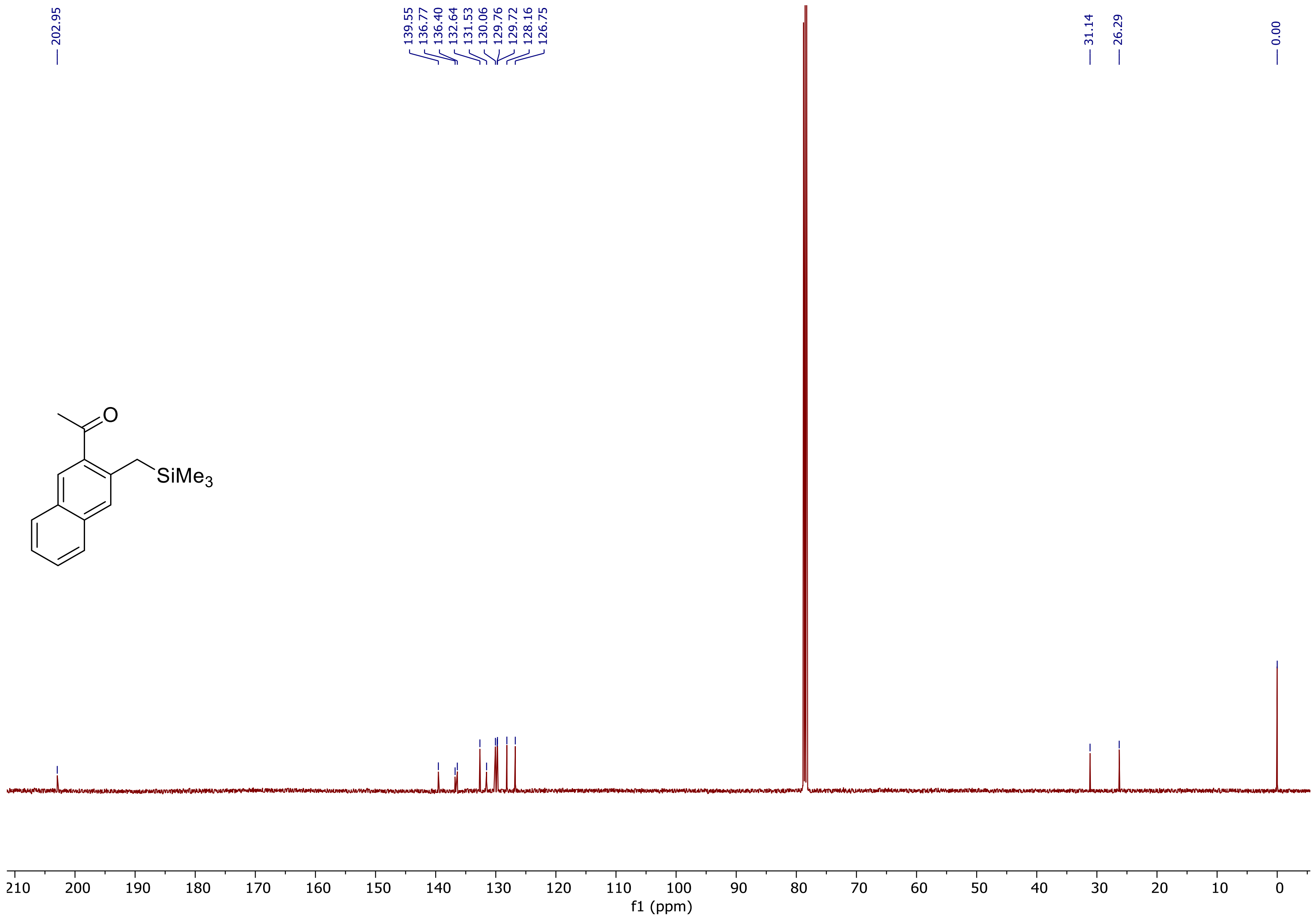

Supplementary Figure 167. ^{13}C NMR (126 MHz, $CDCl_3$) of 1-{3-[(trimethylsilyl)methyl]naphthalen-2-yl}ethan-1-one **4x**.

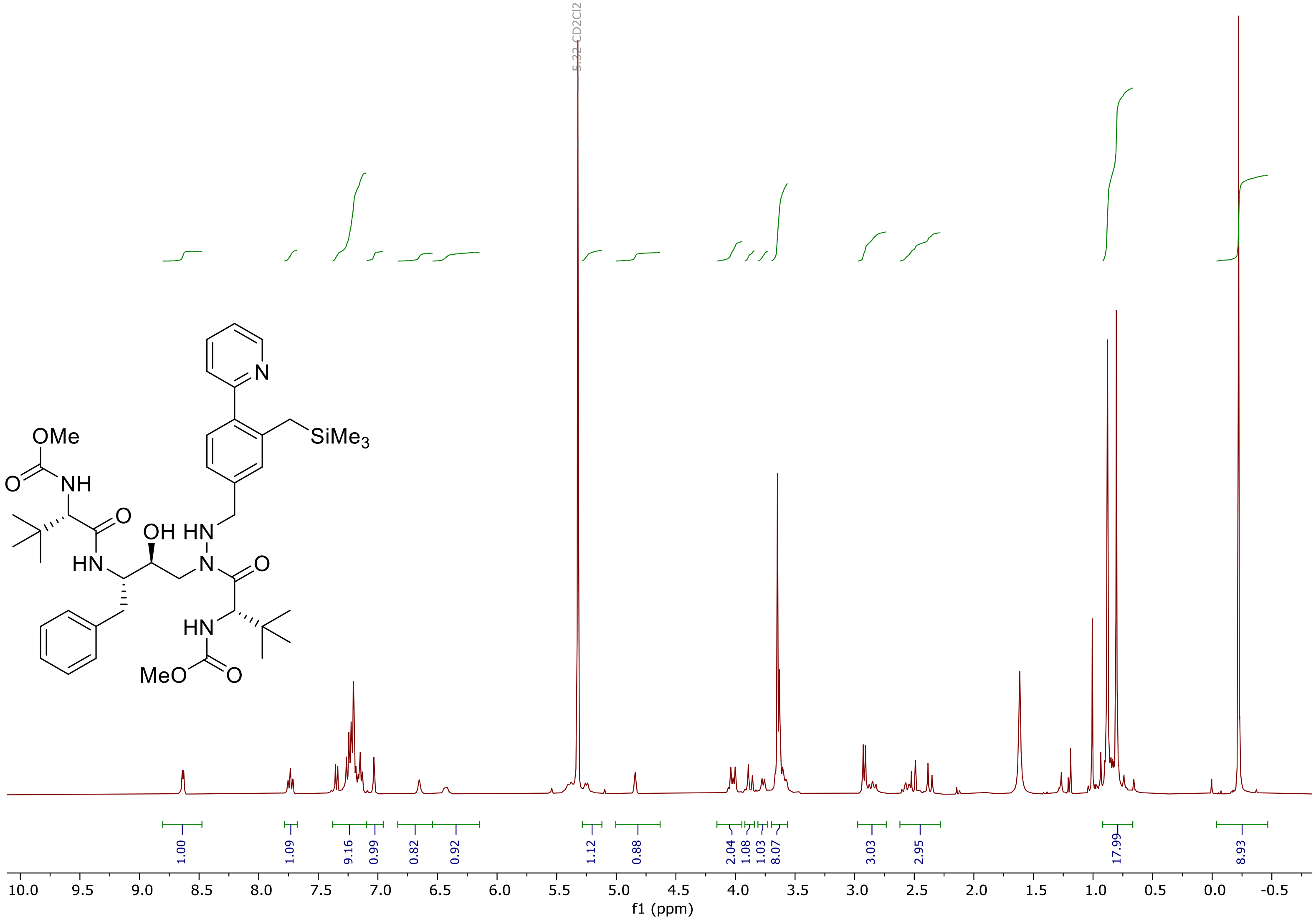

Supplementary Figure 168. ^{1}H NMR (400 MHz, CD_2Cl_2) of methyl {(5S,8S,9S,13S)-8-benzyl-5-(*tert*-butyl)-9-hydroxy-14,14-dimethyl-3,6,12-trioxo-11-[(4-(pyridin-2-yl)-3-((trimethylsilyl)methyl)benzyl)amino]-2-oxa-4,7,11-triazapentadecan-13-yl}carbamate **4y**.

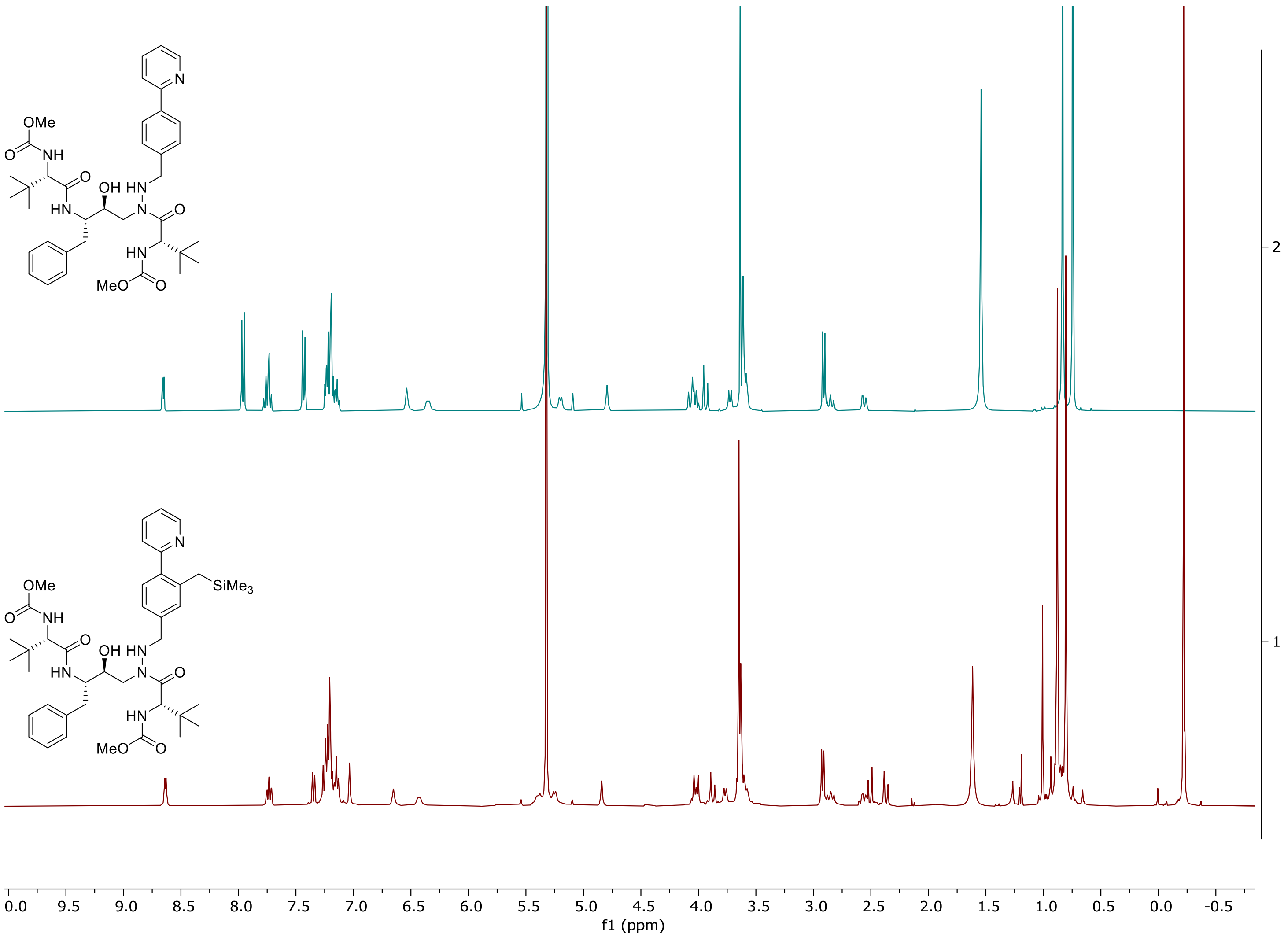

Supplementary Figure 169. ^{1}H NMR (400 MHz, CD_2Cl_2) of (upper – cyan) Atazanavir and (lower – maroon) methyl {(5S,8S,9S,13S)-8-benzyl-5-(*tert*-butyl)-9-hydroxy-14,14-dimethyl-3,6,12-trioxo-11-[(4-(pyridin-2-yl)-3-((trimethylsilyl)methyl)benzyl)amino]-2-oxa-4,7,11-triazapentadecan-13-yl}carbamate **4y**.

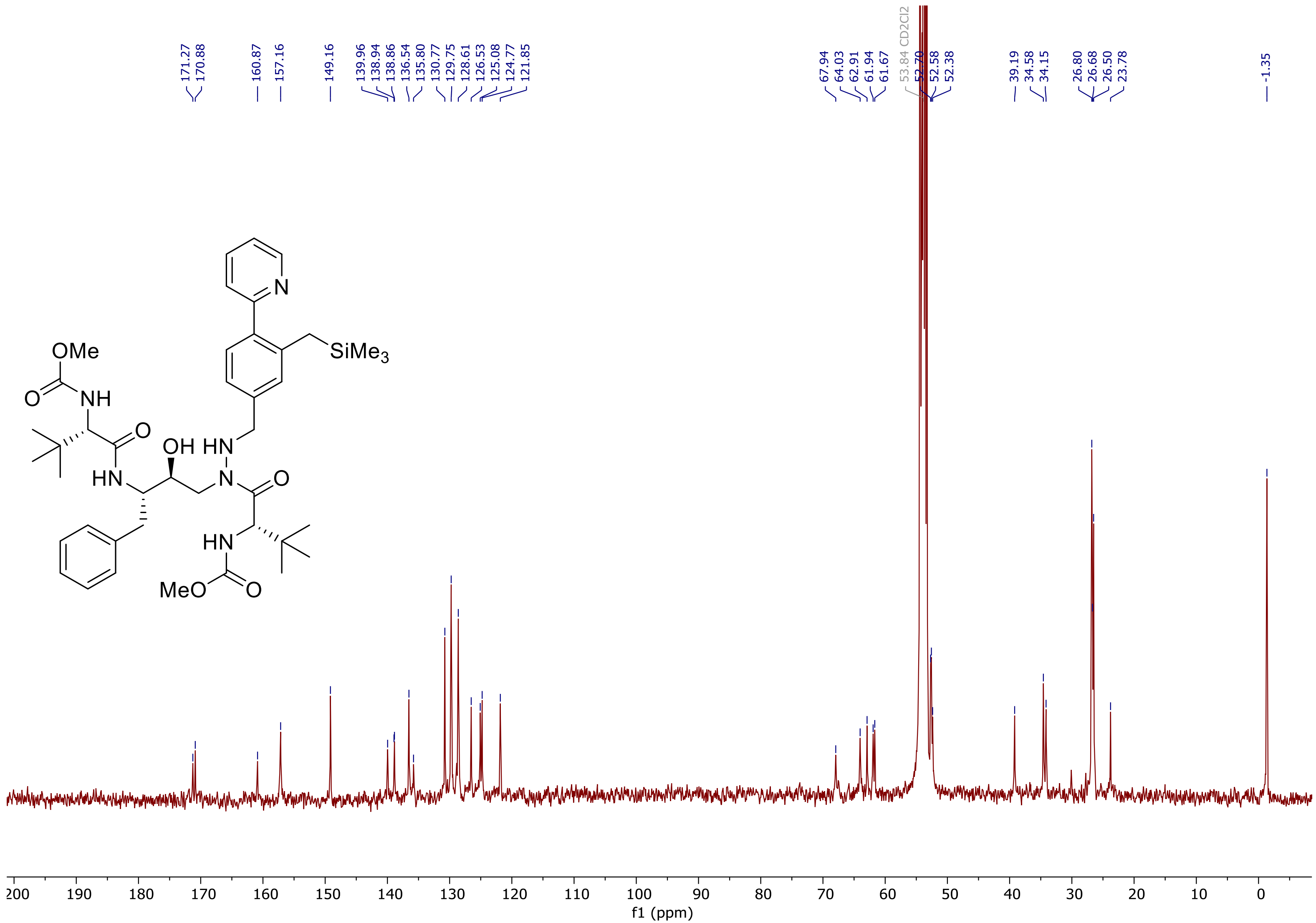

Supplementary Figure 170. ^{13}C NMR (101 MHz, CD_2Cl_2) of methyl {(5S,8S,9S,13S)-8-benzyl-5-(*tert*-butyl)-9-hydroxy-14,14-dimethyl-3,6,12-trioxo-11-[(4-(pyridin-2-yl)-3-((trimethylsilyl)methyl)benzyl)amino]-2-oxa-4,7,11-triazapentadecan-13-yl}carbamate **4y**.

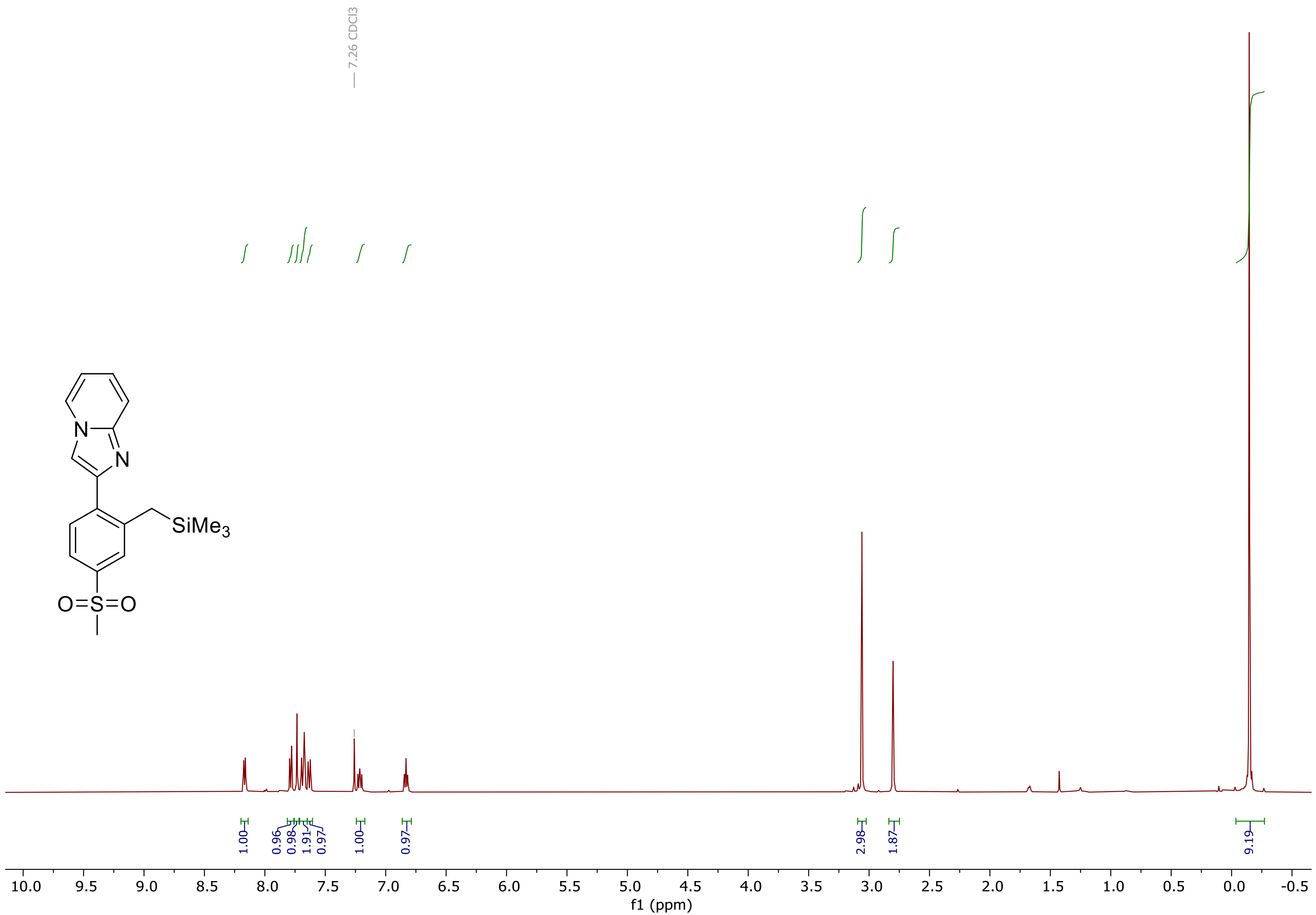

Supplementary Figure 171. ^{1}H NMR (500 MHz, $CDCl_3$) of 2-{4-(methylsulfonyl)-2-[(trimethylsilyl)methyl]phenyl}imidazo[1,2-a]pyridine **4z**.

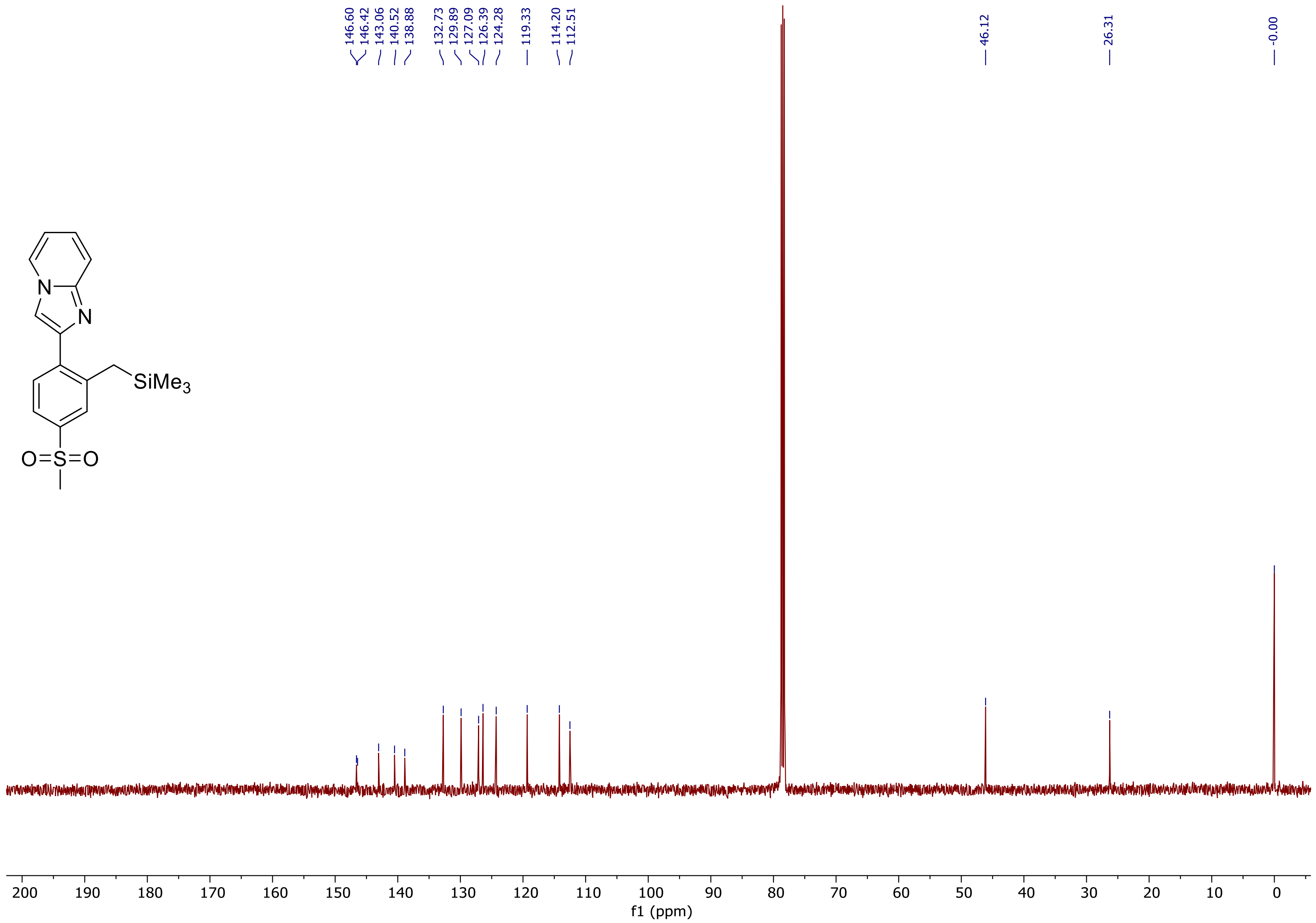

Supplementary Figure 172. ^{13}C NMR (126 MHz, $CDCl_3$) of 2-{4-(methylsulfonyl)-2-[(trimethylsilyl)methyl]phenyl}imidazo[1,2-a]pyridine **4z**.

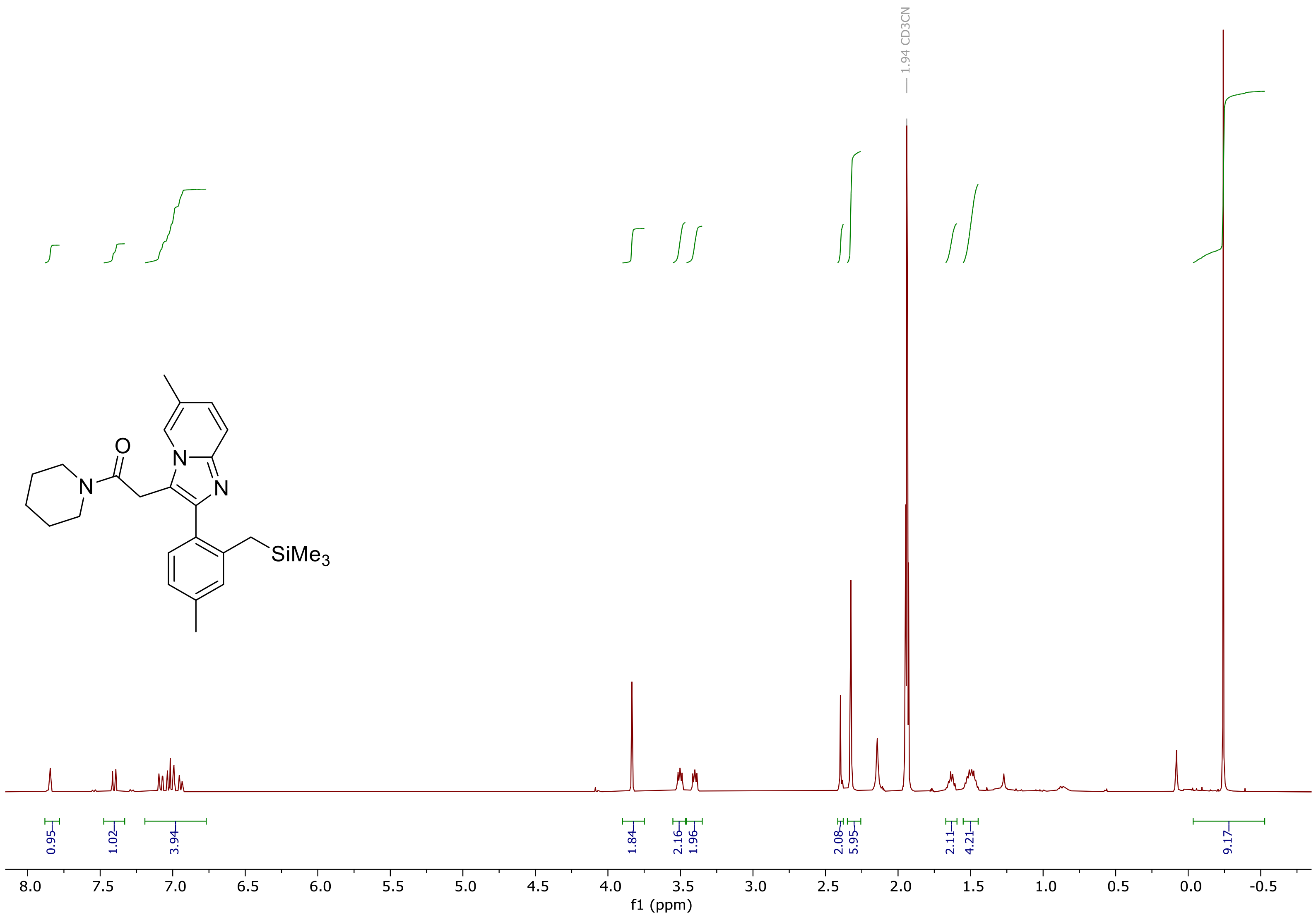

Supplementary Figure 173. ^{1}H NMR (400 MHz, d_3-CD_3CN) of 2-{6-bethyl-2-[4-methyl-2-((trimethylsilyl)methyl)phenyl]imidazo[1,2-a]pyridin-3-yl}-1-(piperidin-1-yl)ethan-1-one **4aa**.

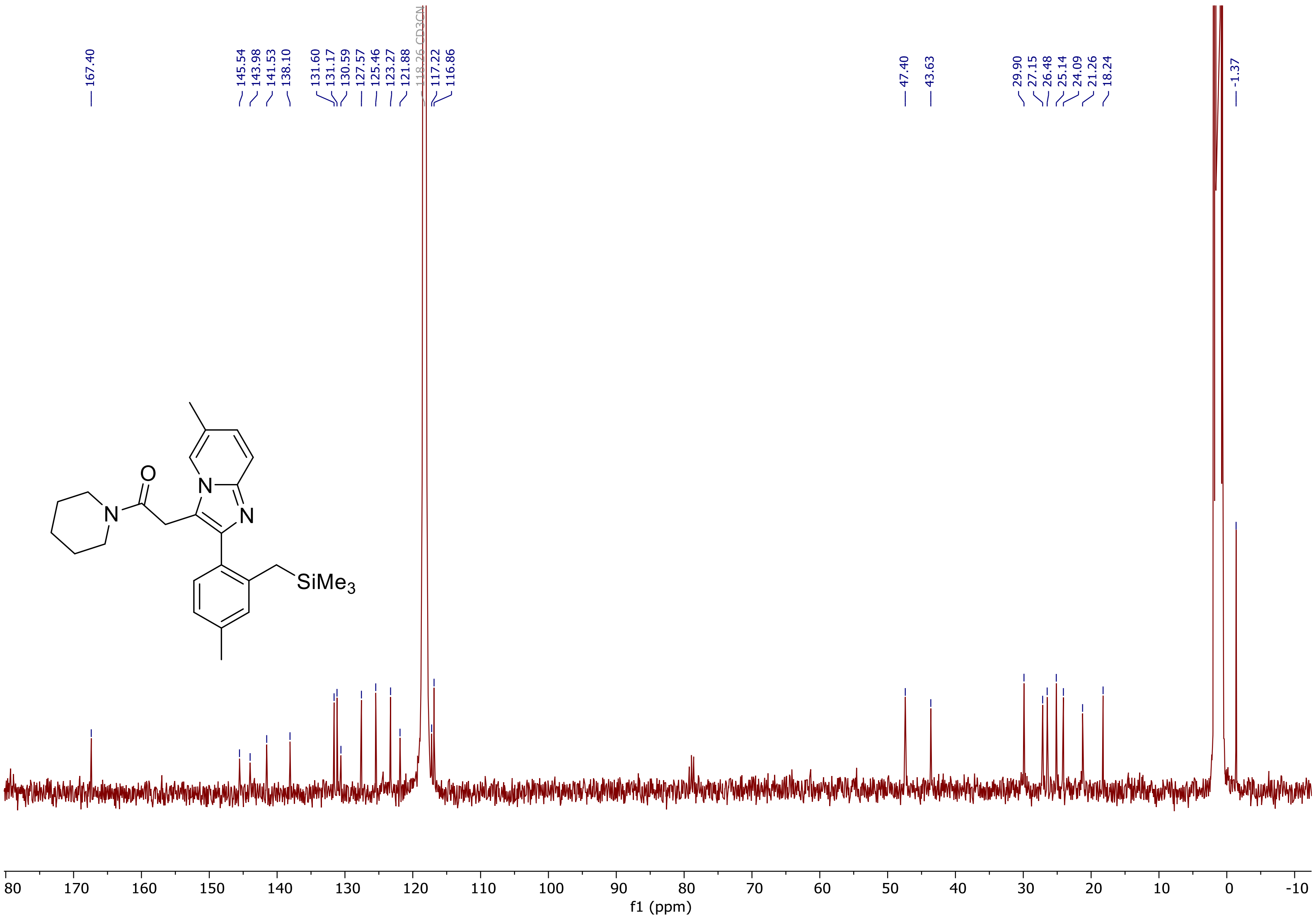

Supplementary Figure 174. ^{13}C NMR (126 MHz, d_3-CD_3CN) of 2-{6-bethyl-2-[4-methyl-2-((trimethylsilyl)methyl)phenyl]imidazo[1,2-a]pyridin-3-yl}-1-(piperidin-1-yl)ethan-1-one **4aa**.

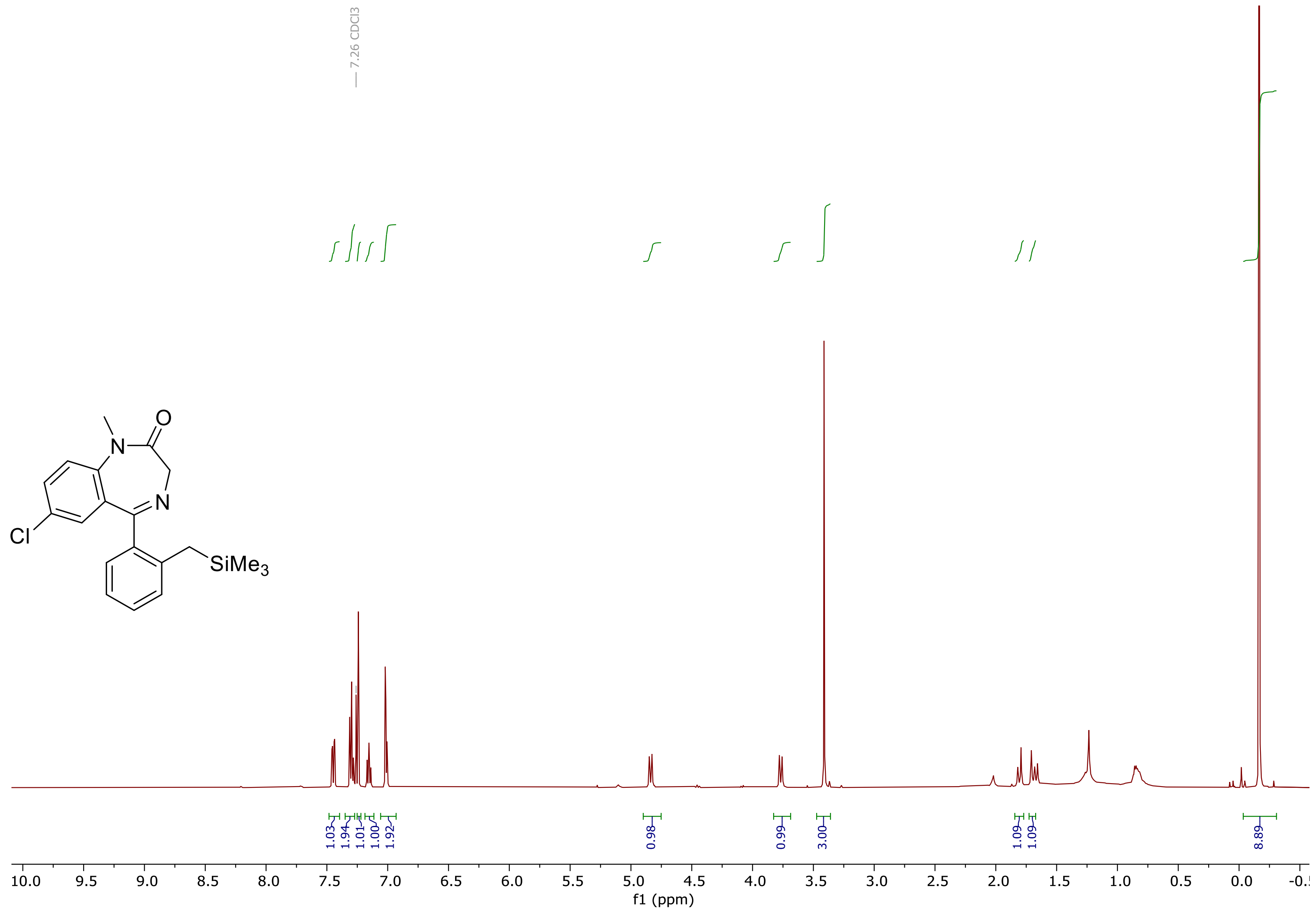

Supplementary Figure 175. ^{1}H NMR (500 MHz, $CDCl_3$) of 7-chloro-1-methyl-5-{2-[(trimethylsilyl)methyl]phenyl}-1,3-dihydro-2H-benzo[e][1,4]diazepin-2-one **4ab**.

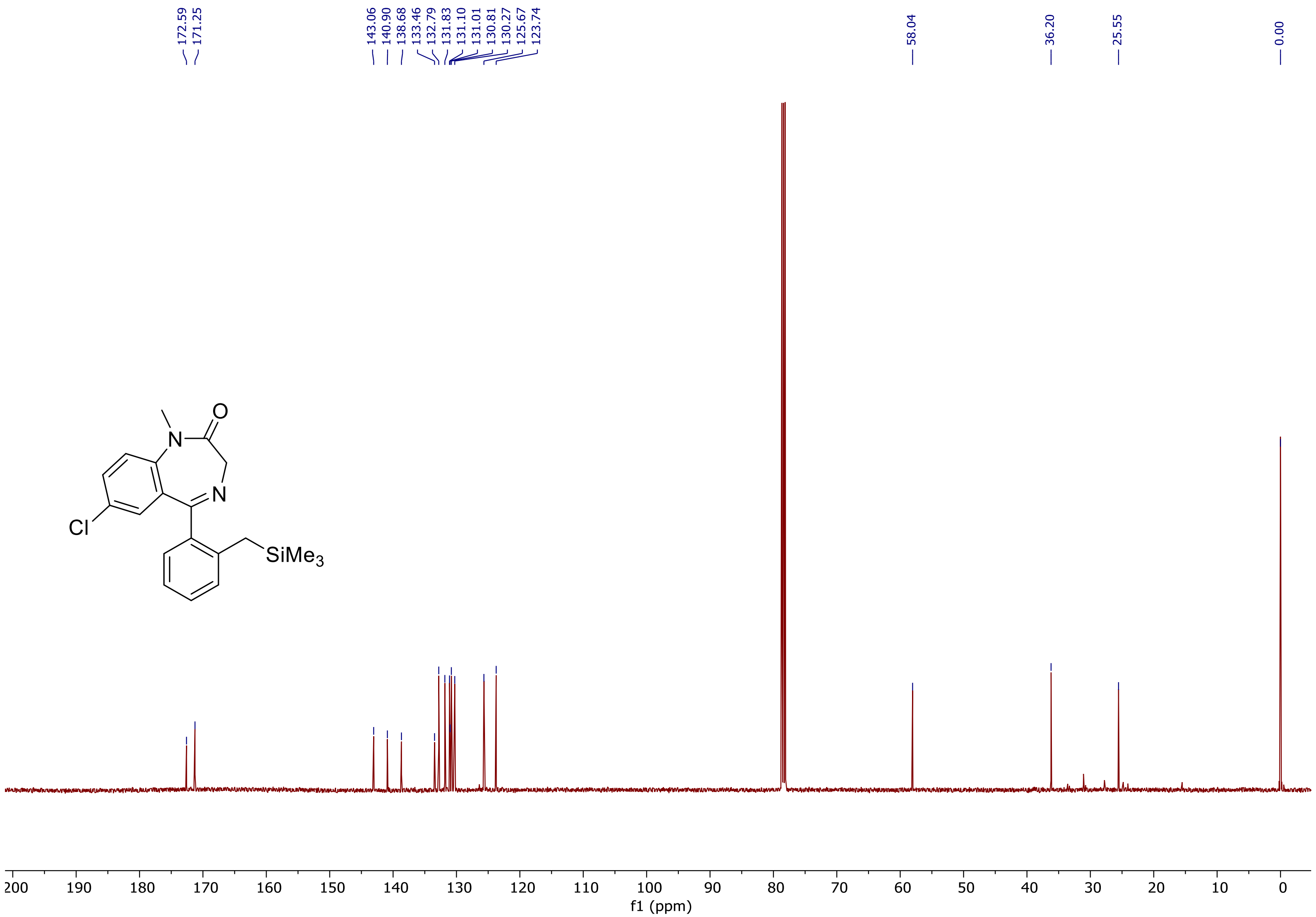

Supplementary Figure 176. ^{13}C NMR (126 MHz, $CDCl_3$) of 7-chloro-1-methyl-5-{2-[(trimethylsilyl)methyl]phenyl}-1,3-dihydro-2H-benzo[e][1,4]diazepin-2-one **4ab**.

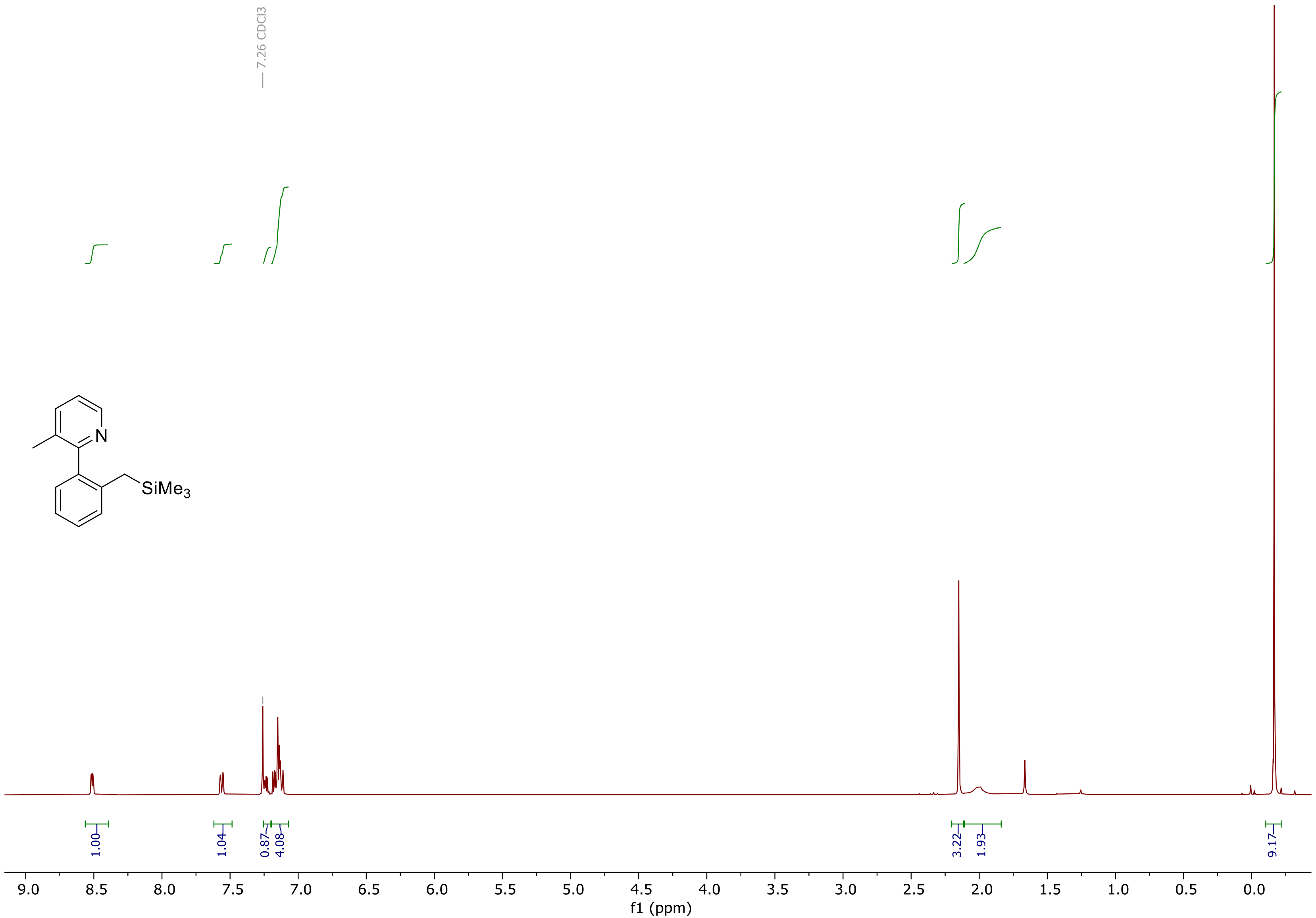

Supplementary Figure 177. ^{1}H NMR (400 MHz, $CDCl_3$) of 3-methyl-2-{2-[(trimethylsilyl)methyl]phenyl}pyridine.

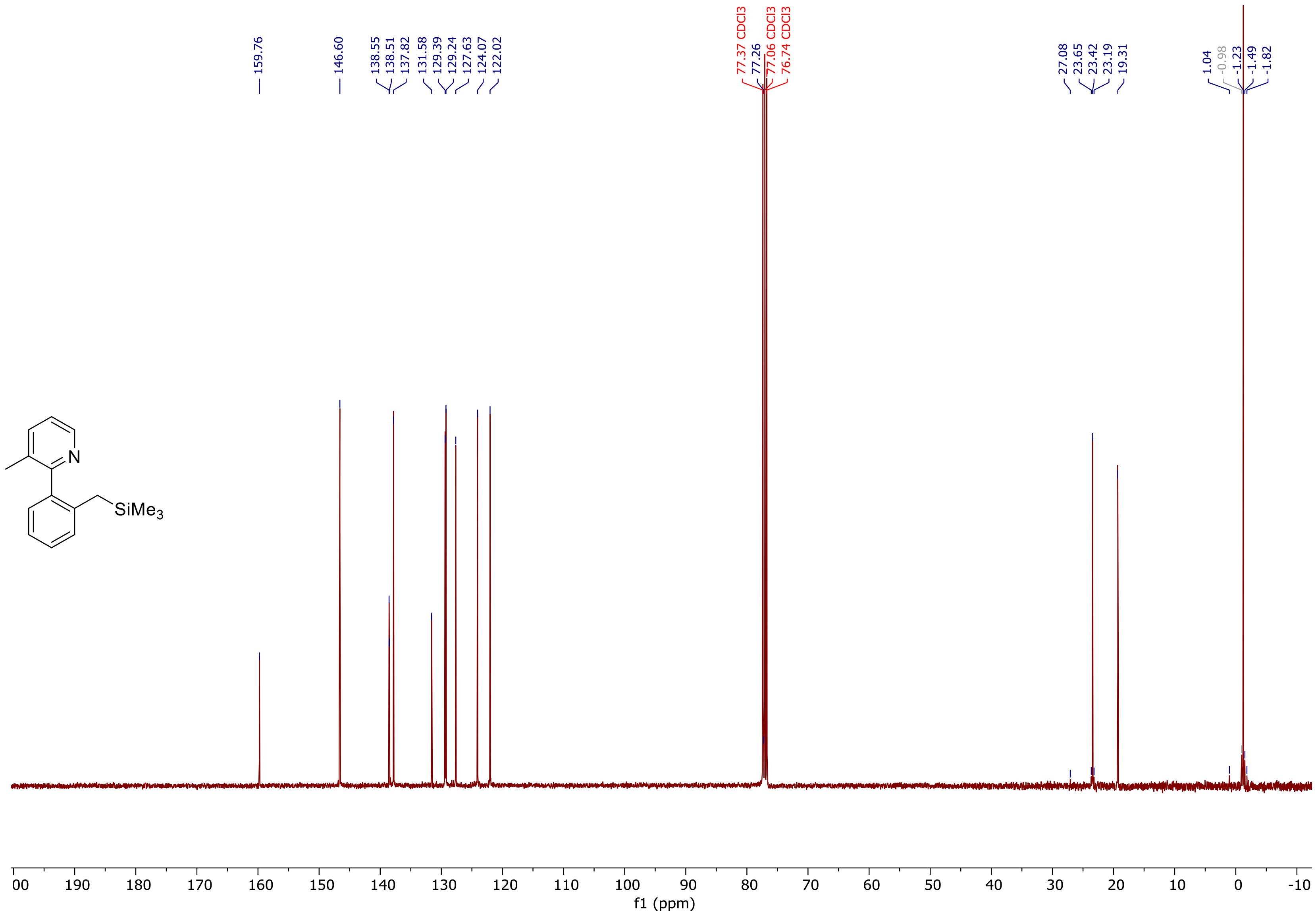

Supplementary Figure 178. ^{13}C NMR (101 MHz, $CDCl_3$) of 3-methyl-2-{2-[(trimethylsilyl)methyl]phenyl}pyridine.

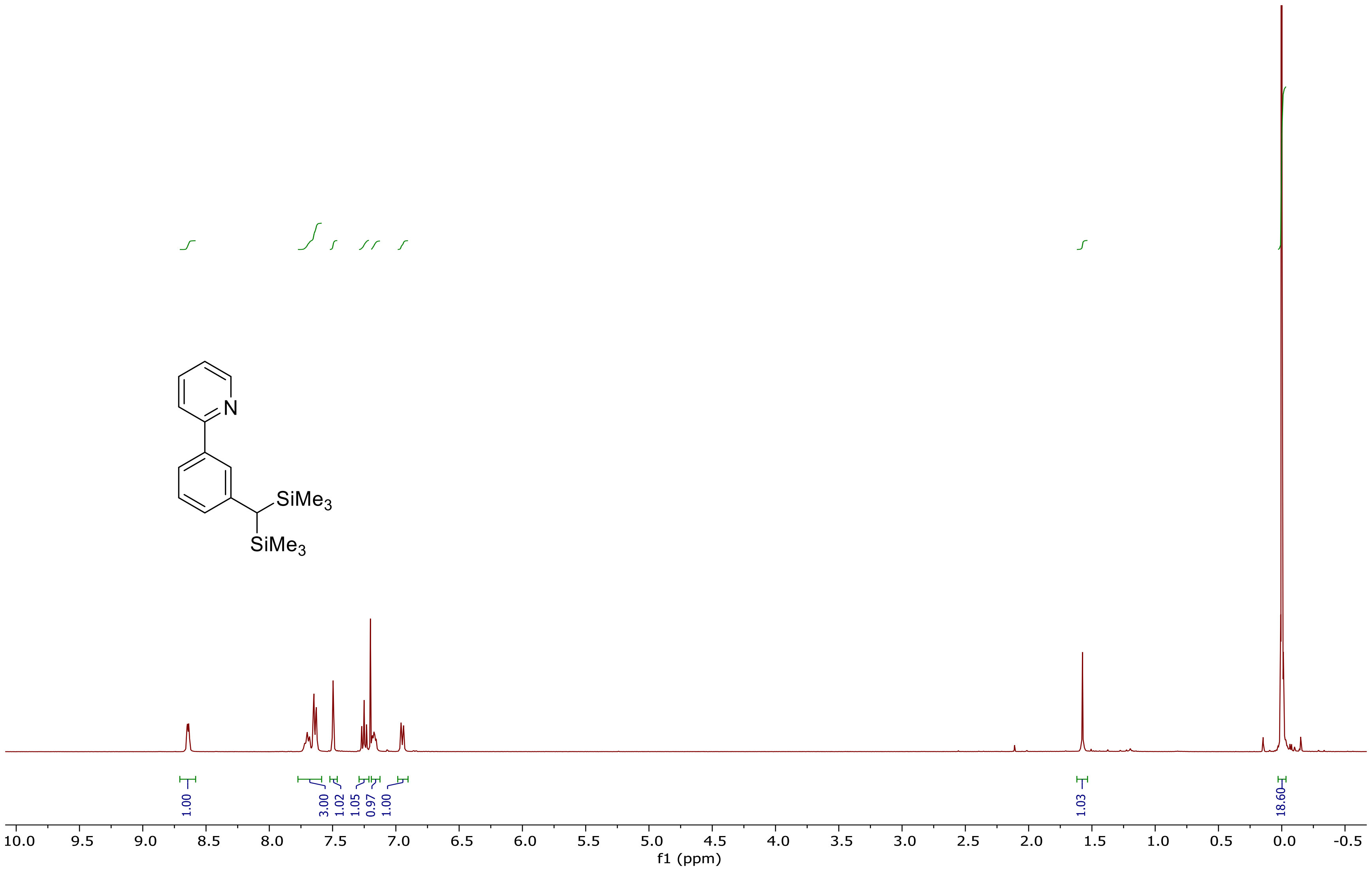

Supplementary Figure 179. ^{1}H NMR (400 MHz, $CDCl_3$) of 2-{3-[bis(trimethylsilyl)methyl]phenyl}pyridine **6a**.

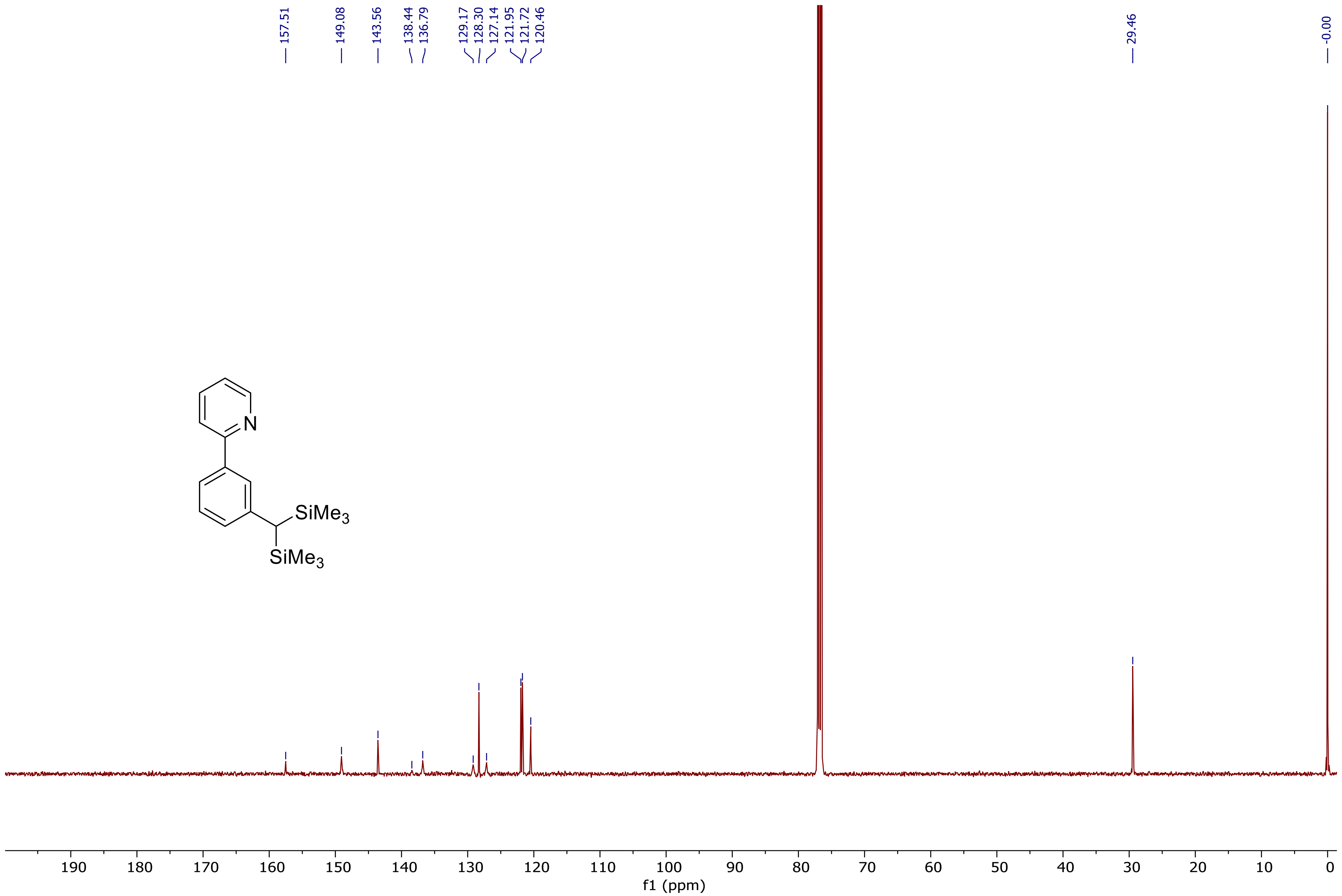

Supplementary Figure 180. ^{13}C NMR (126 MHz, $CDCl_3$) of 2-{3-[bis(trimethylsilyl)methyl]phenyl}pyridine **6a**

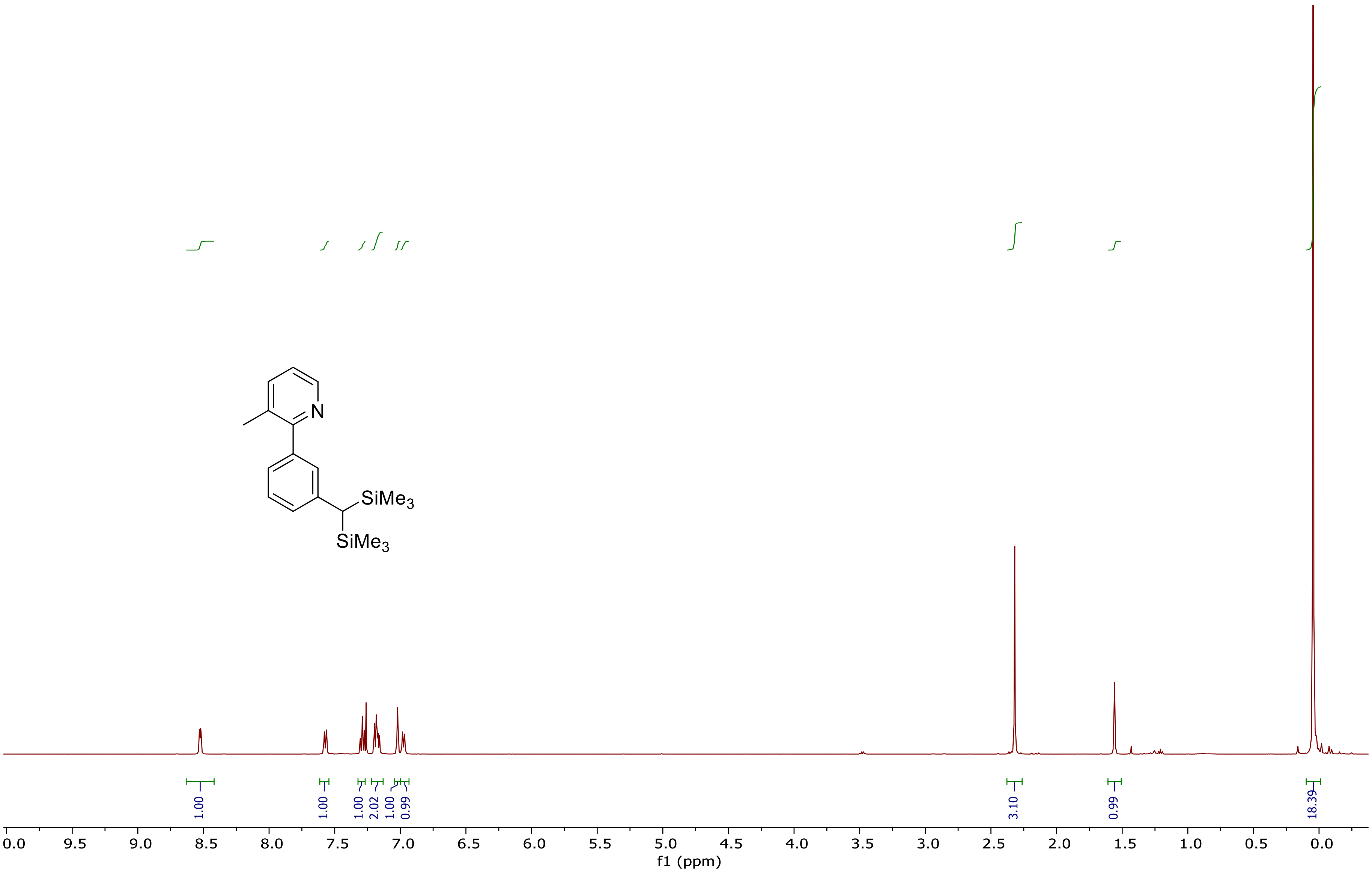

Supplementary Figure 181. ^{1}H NMR (500 MHz, $CDCl_3$) of 2-{3-[bis(trimethylsilyl)methyl]phenyl}pyridine **6b**.

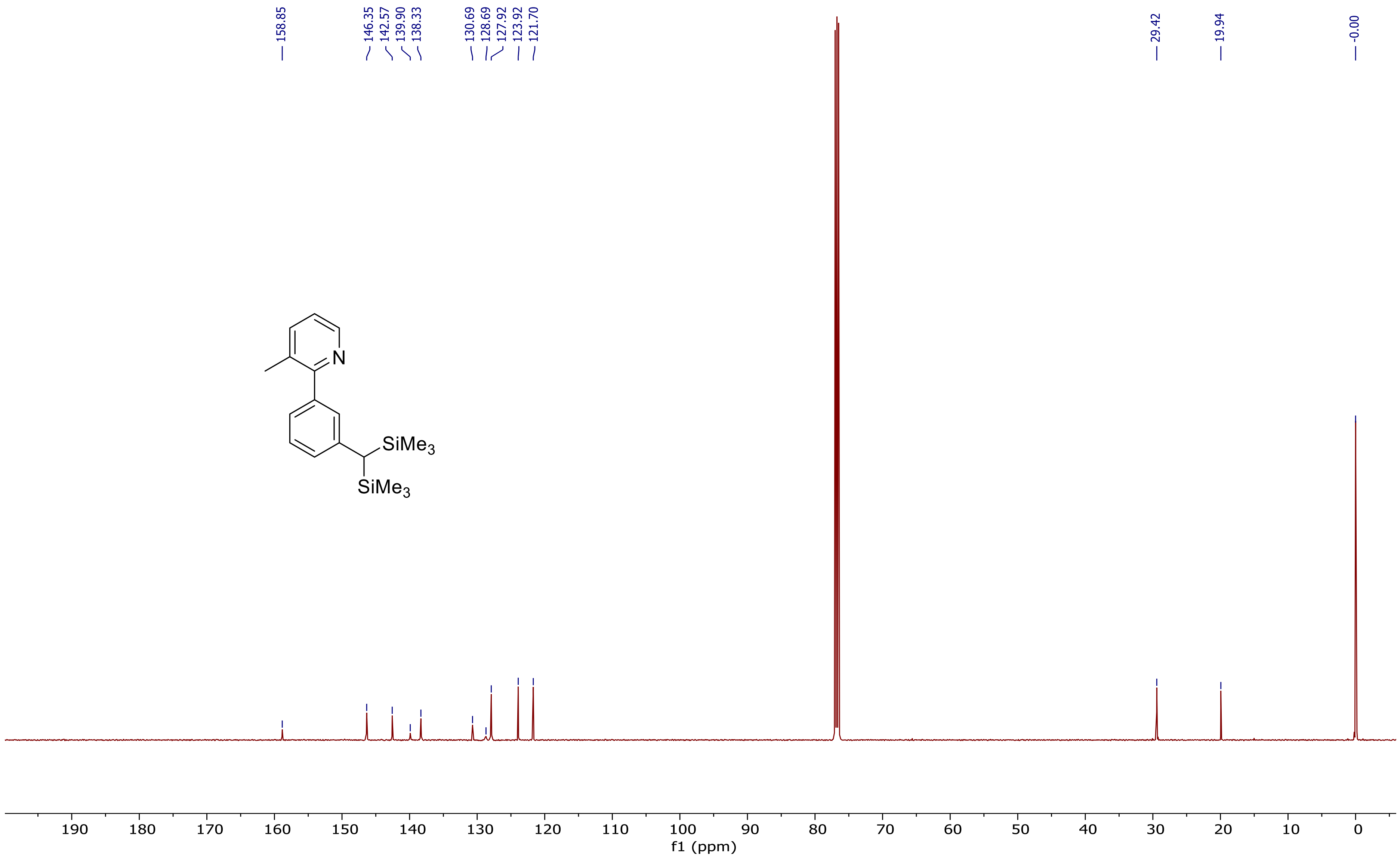

Supplementary Figure 182. ^{13}C NMR (126 MHz, $CDCl_3$) of 2-(3-(bis(trimethylsilyl)methyl)phenyl)pyridine **6b**.

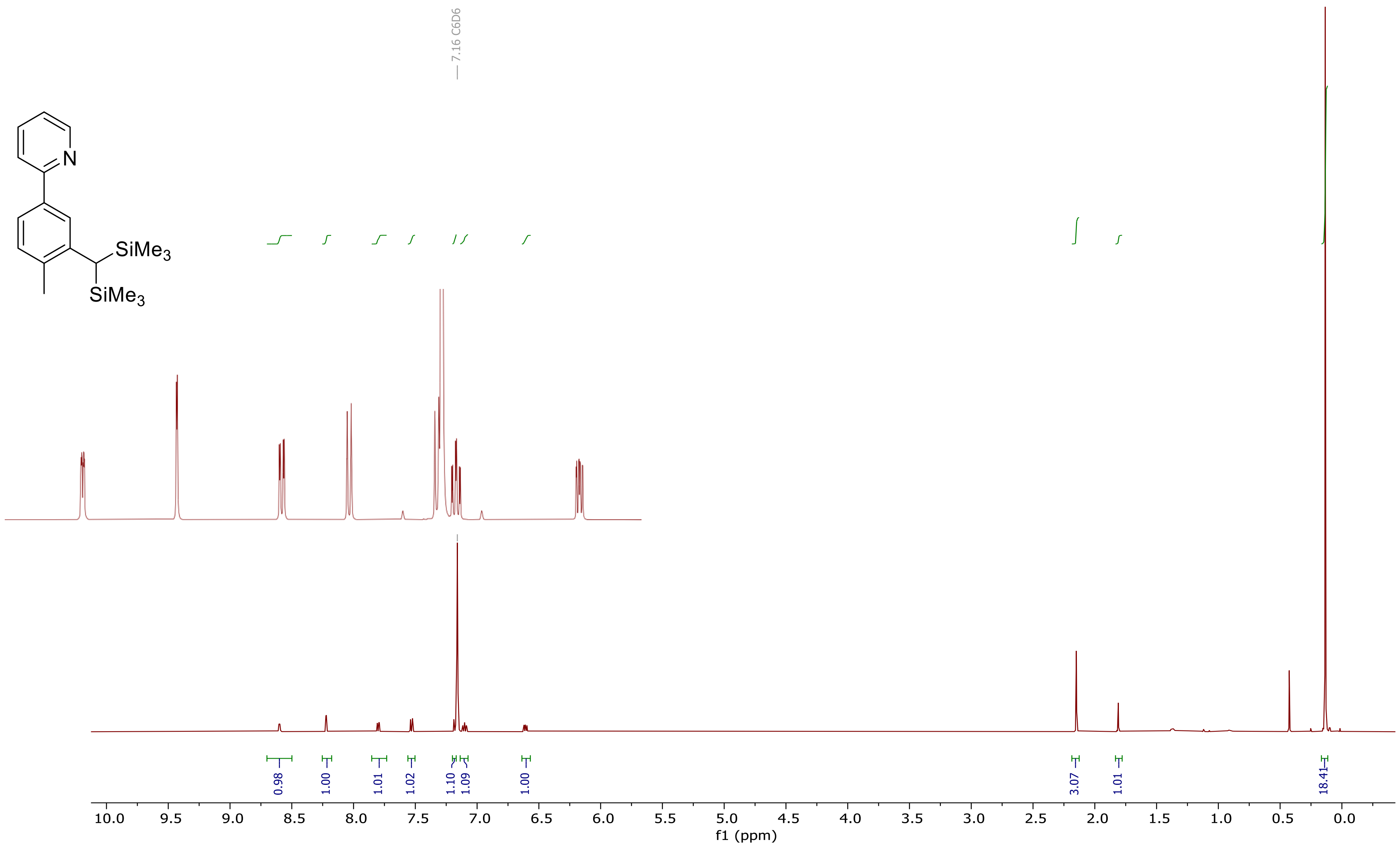

Supplementary Figure 183. ^{1}H NMR (500 MHz, C_6D_6) of 2-{3-[bis(trimethylsilyl)methyl]-4-methylphenyl}pyridine **6c**.

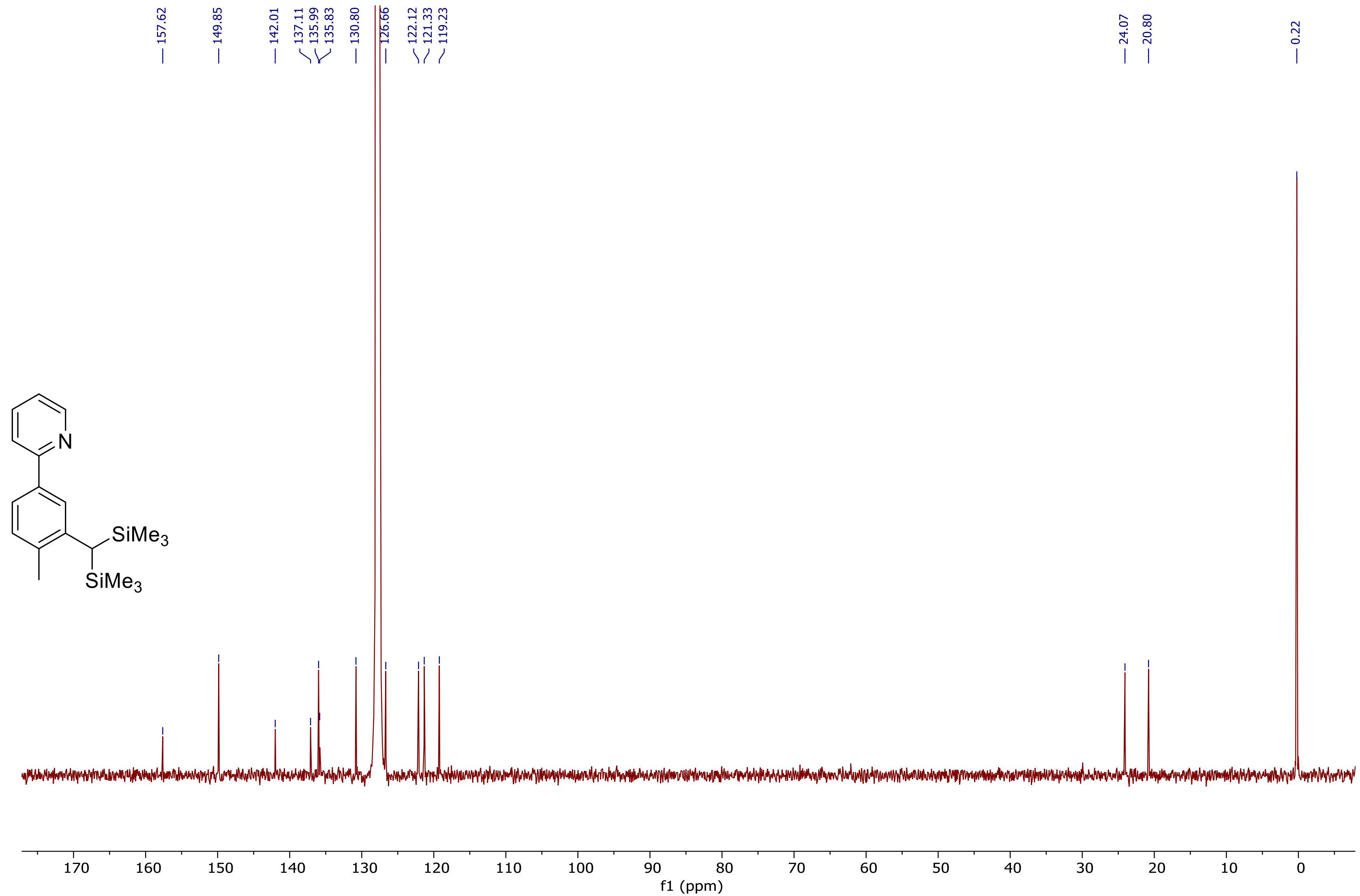

Supplementary Figure 184. ^{13}C NMR (126 MHz, C_6D_6) of 2-{3-[bis(trimethylsilyl)methyl]-4-methylphenyl}pyridine **6c**.

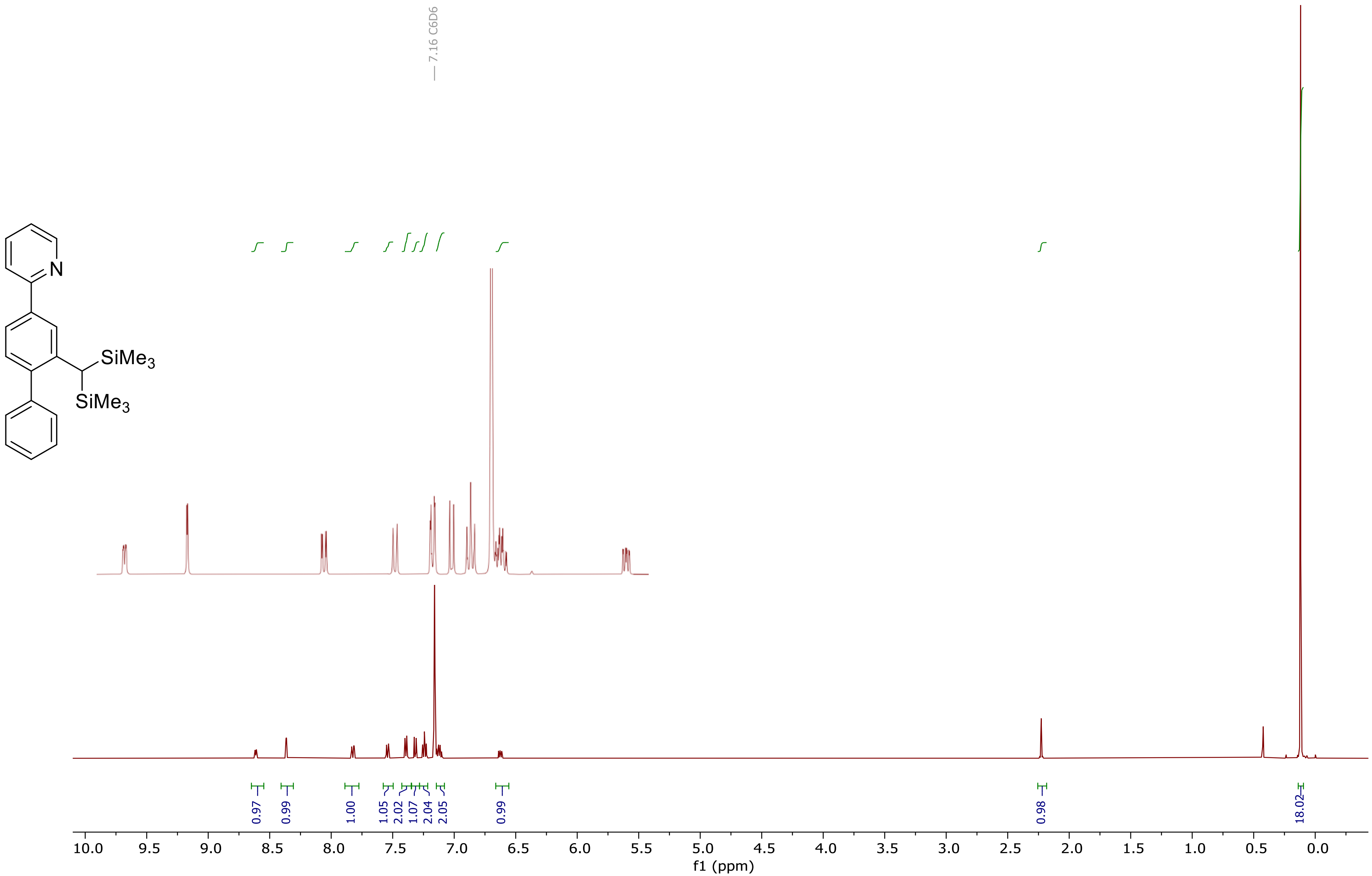

Supplementary Figure 185. ^{1}H NMR (500 MHz, C_6D_6) of 2-{2-[bis(trimethylsilyl)methyl]-[1,1'-biphenyl]-4-yl}pyridine **6d**.

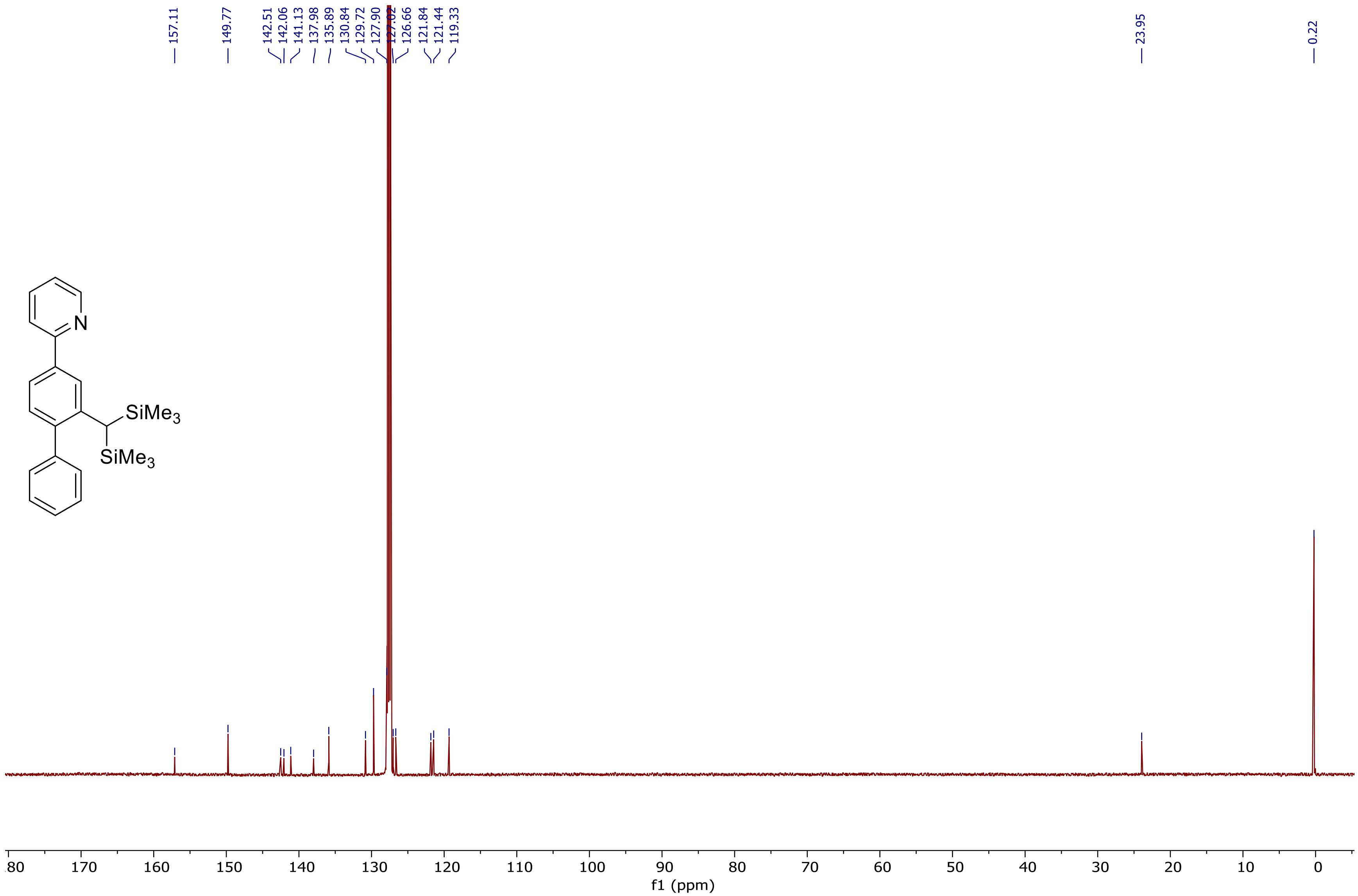

Supplementary Figure 186. ^{13}C NMR (126 MHz, C_6D_6) of 2-{2-[bis(trimethylsilyl)methyl]-[1,1'-biphenyl]-4-yl}pyridine **6d**.

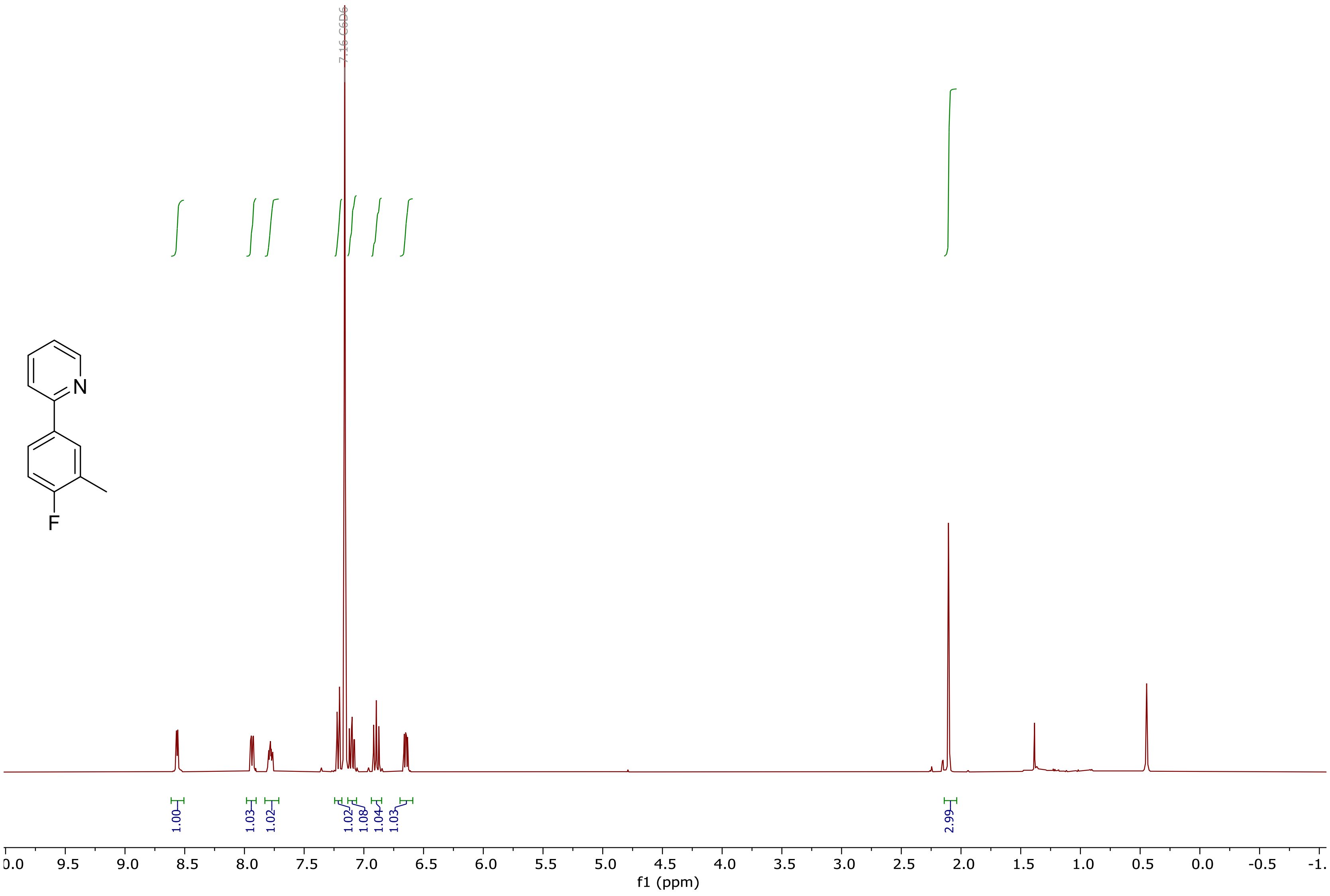

Supplementary Figure 187. ^{1}H NMR (400 MHz, C_6D_6) of 2-(4-fluoro-3-methylphenyl)pyridine **6e**.

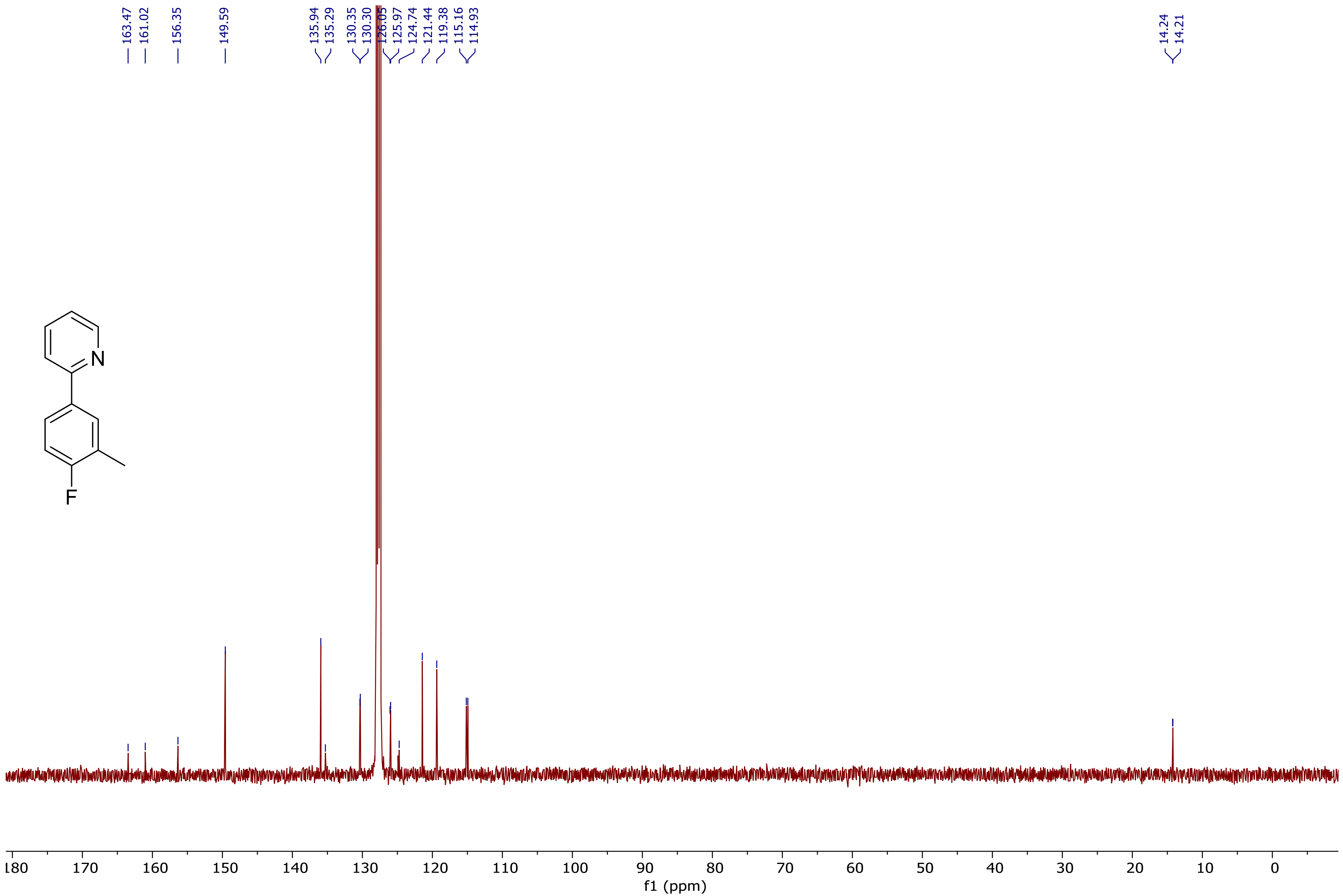

Supplementary Figure 188. ^{13}C NMR (101 MHz, C_6D_6) of 2-(4-fluoro-3-methylphenyl)pyridine **6e**.

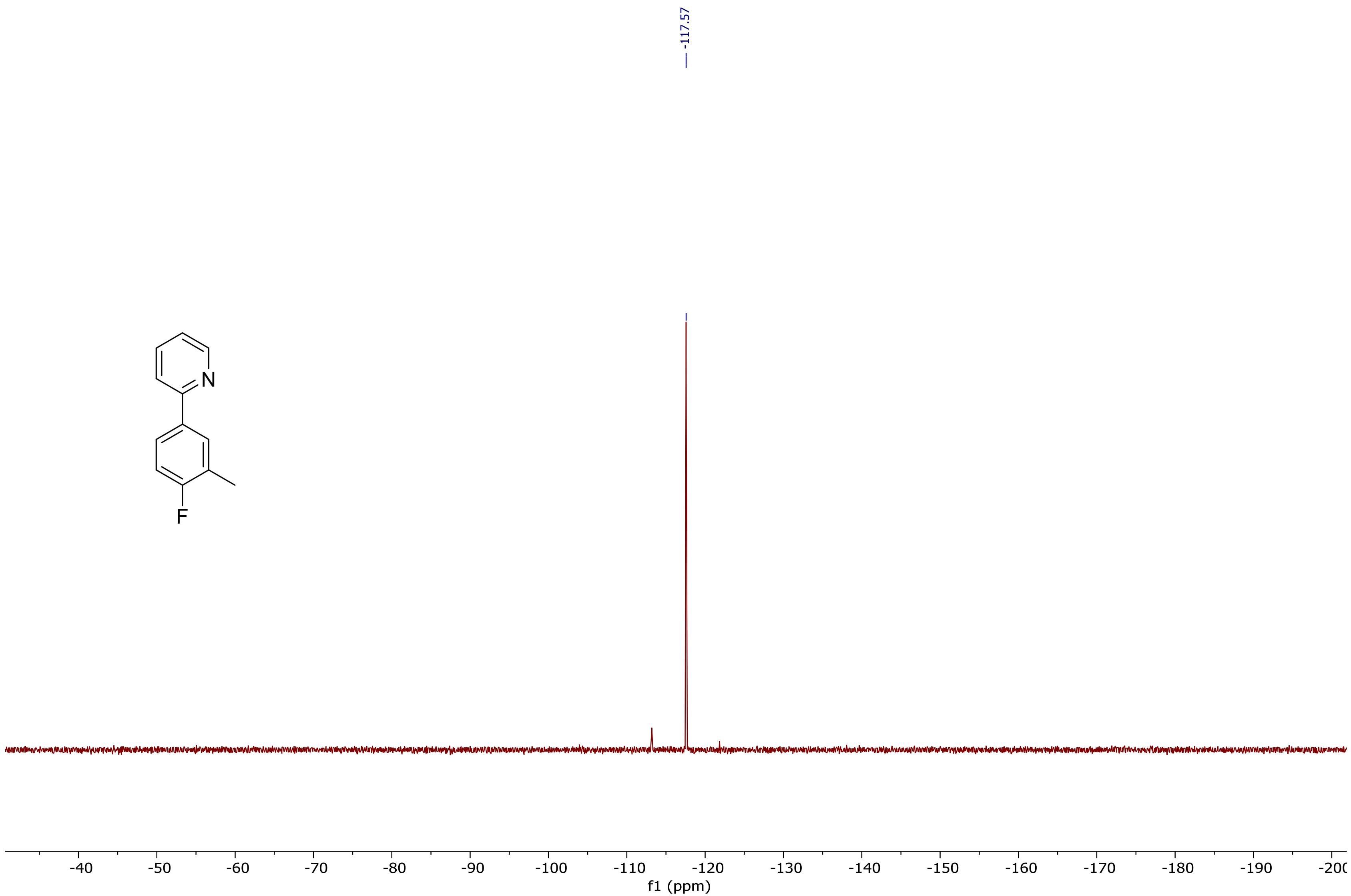

Supplementary Figure 189. ^{19}F NMR (376 MHz, C_6D_6) of 2-(4-fluoro-3-methylphenyl)pyridine **6e**.

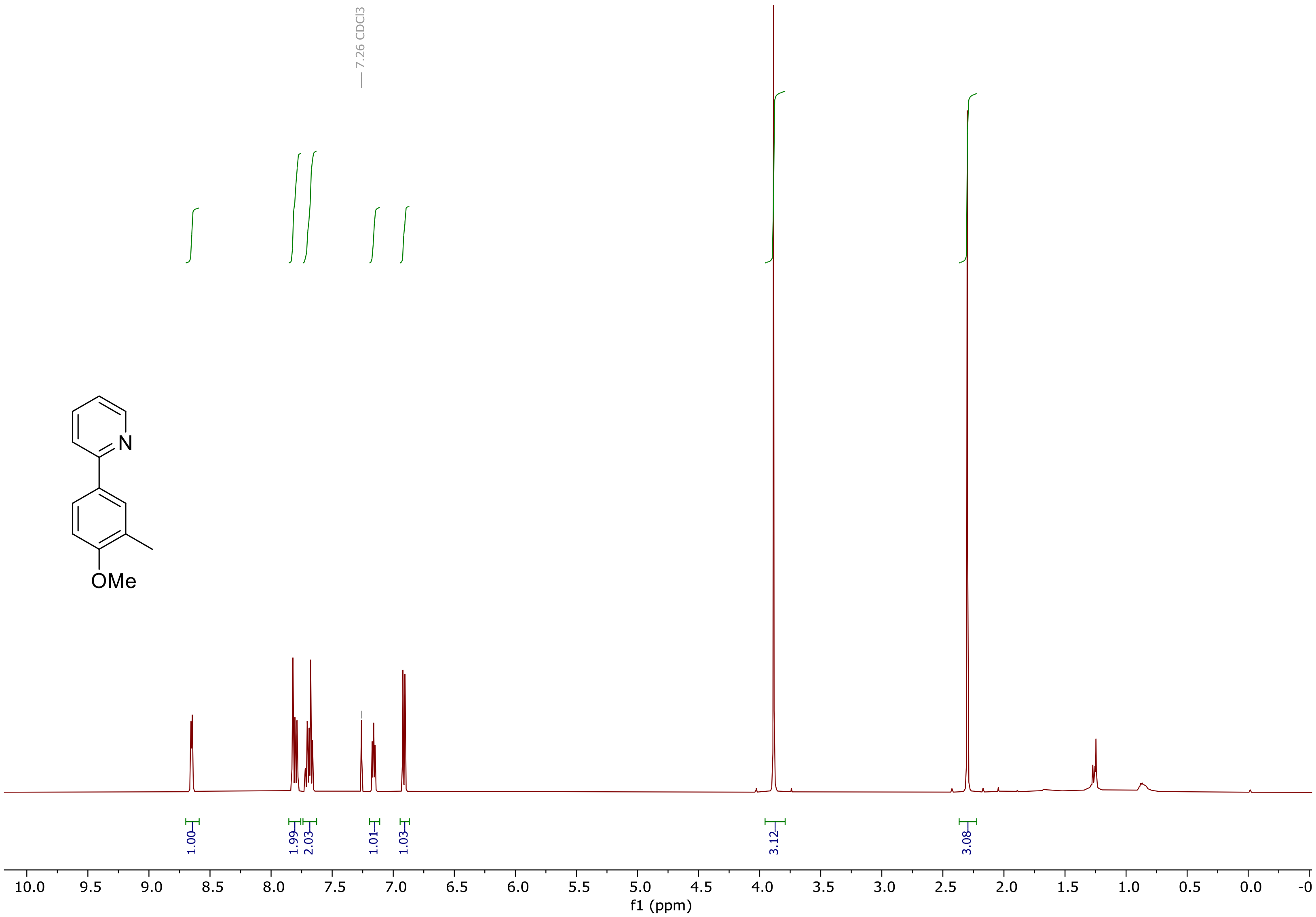

Supplementary Figure 190. ^{1}H NMR (500 MHz, $CDCl_3$) of 2-(4-methoxy-3-methylphenyl)pyridine **6f**.

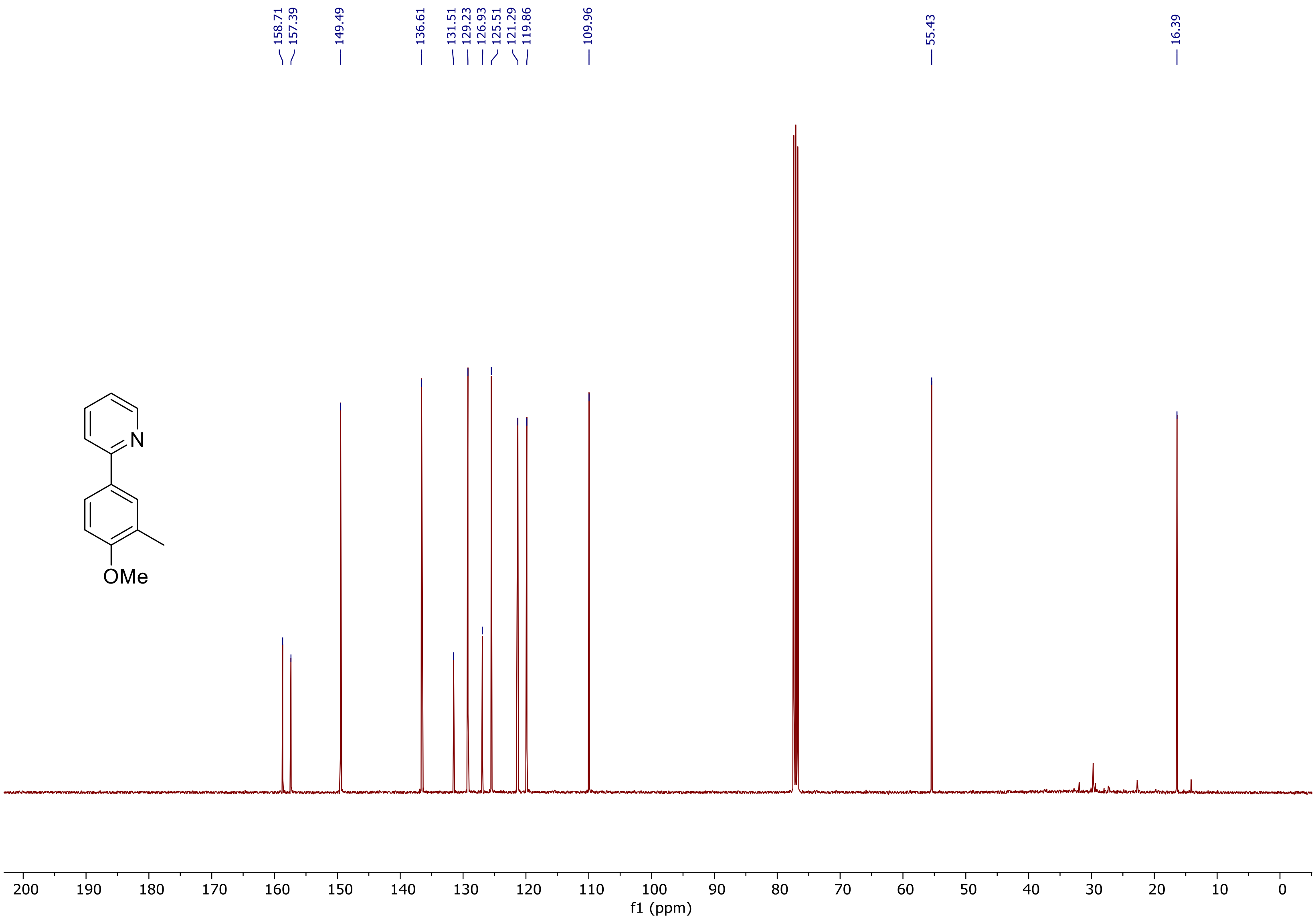

Supplementary Figure 191. ^{13}C NMR (101 MHz, $CDCl_3$) of 2-(4-methoxy-3-methylphenyl)pyridine **6f**.

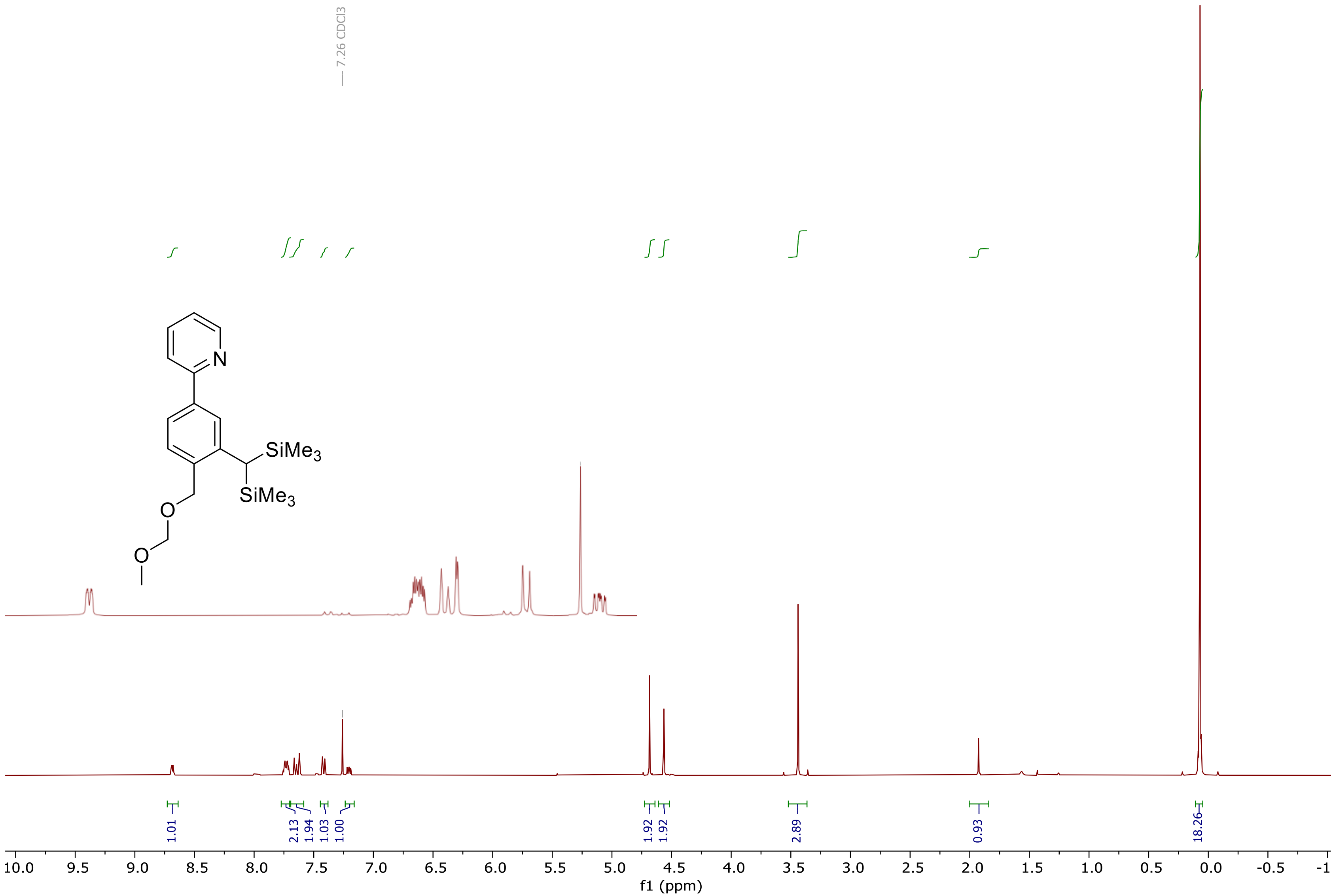

Supplementary Figure 192. ^{1}H NMR (400 MHz, $CDCl_3$) of 2-{3-[bis(trimethylsilyl)methyl)-4-((methoxymethoxy)methyl]phenyl}pyridine **6g**.

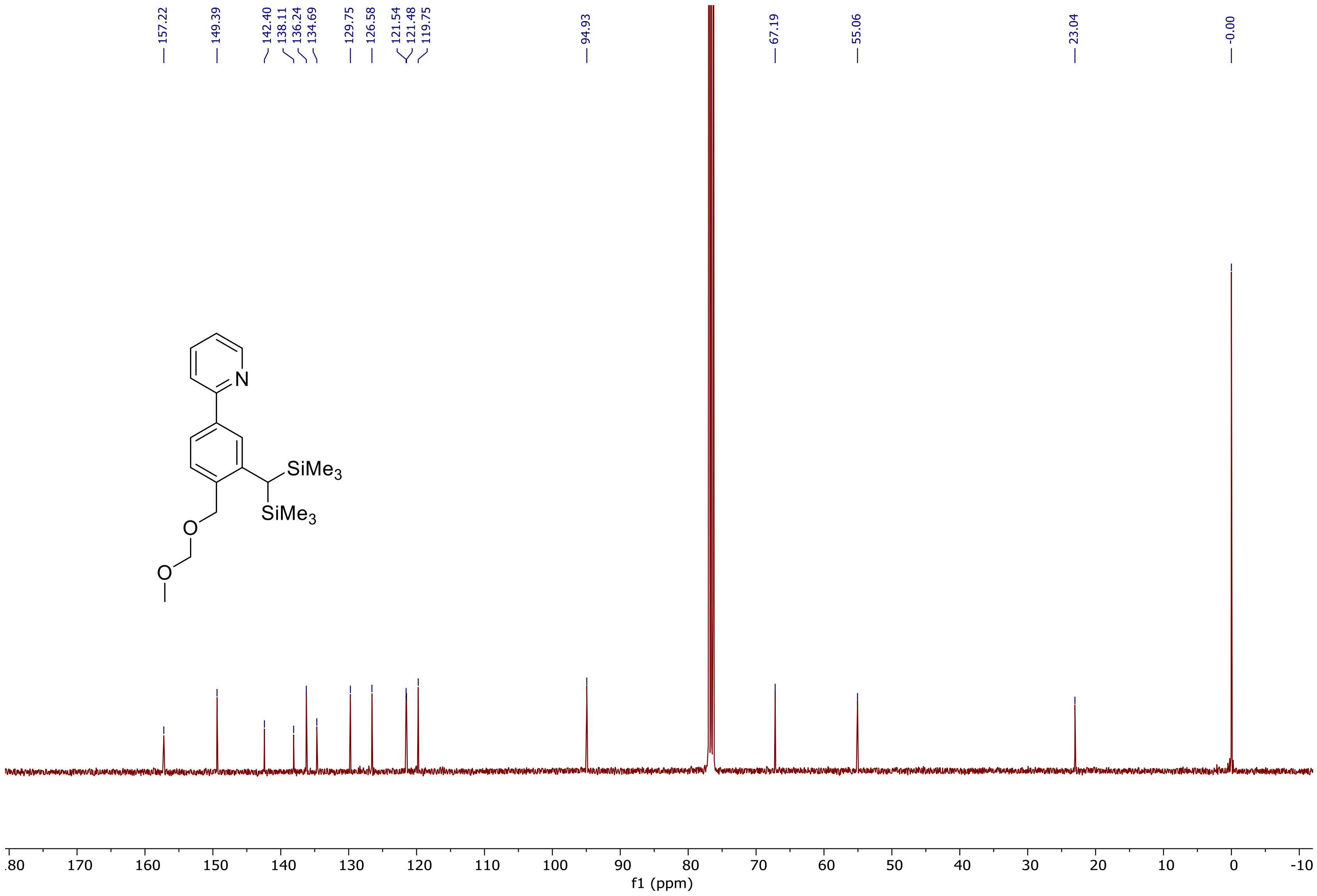

Supplementary Figure 193. ^{13}C NMR (101 MHz, $CDCl_3$) of 2-{3-[bis(trimethylsilyl)methyl)-4-((methoxymethoxy)methyl]phenyl}pyridine **6g**.

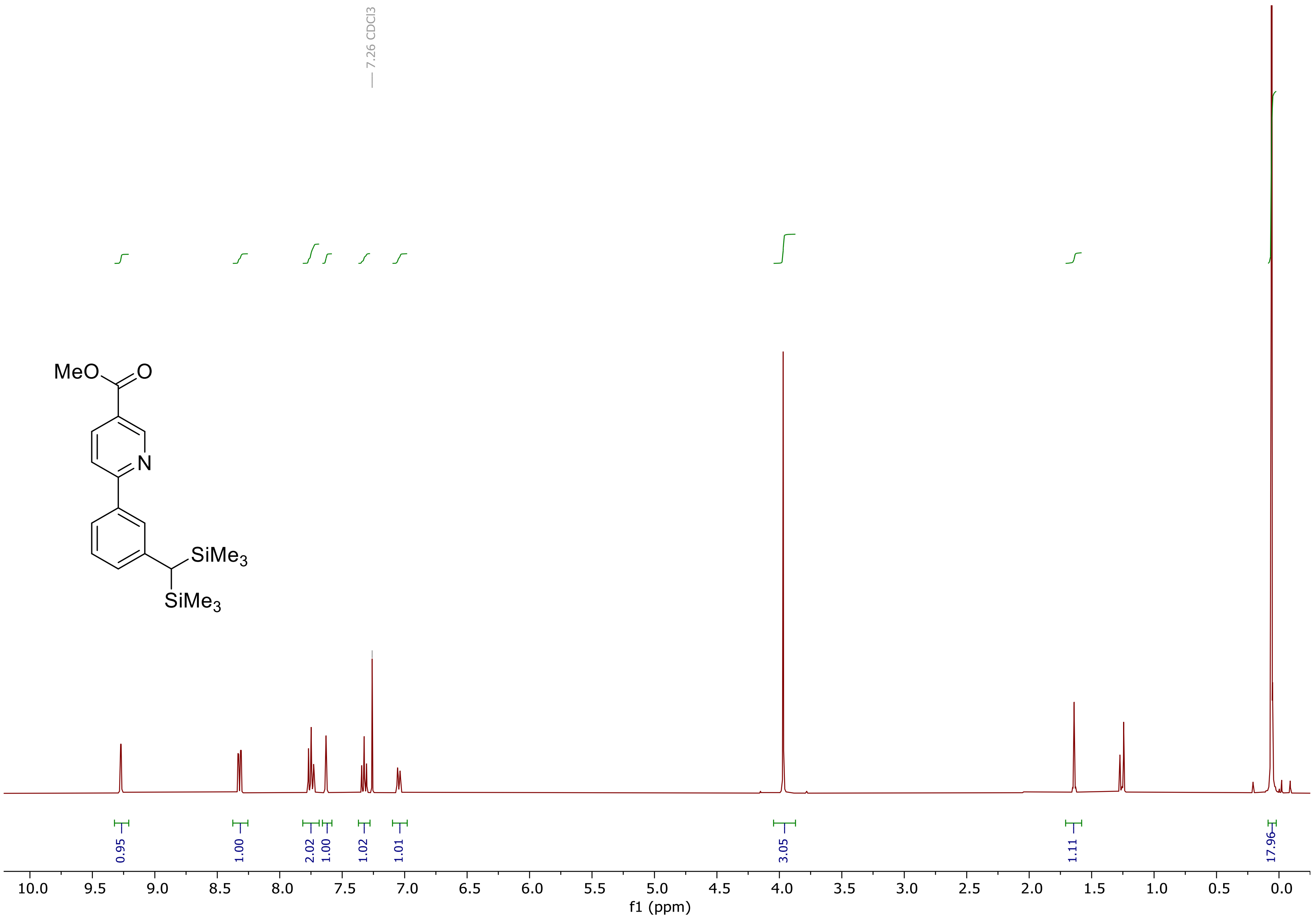

Supplementary Figure 194. ^{1}H NMR (400 MHz, $CDCl_3$) of methyl 6-{3-[bis(trimethylsilyl)methyl]phenyl}nicotinate **6h**.

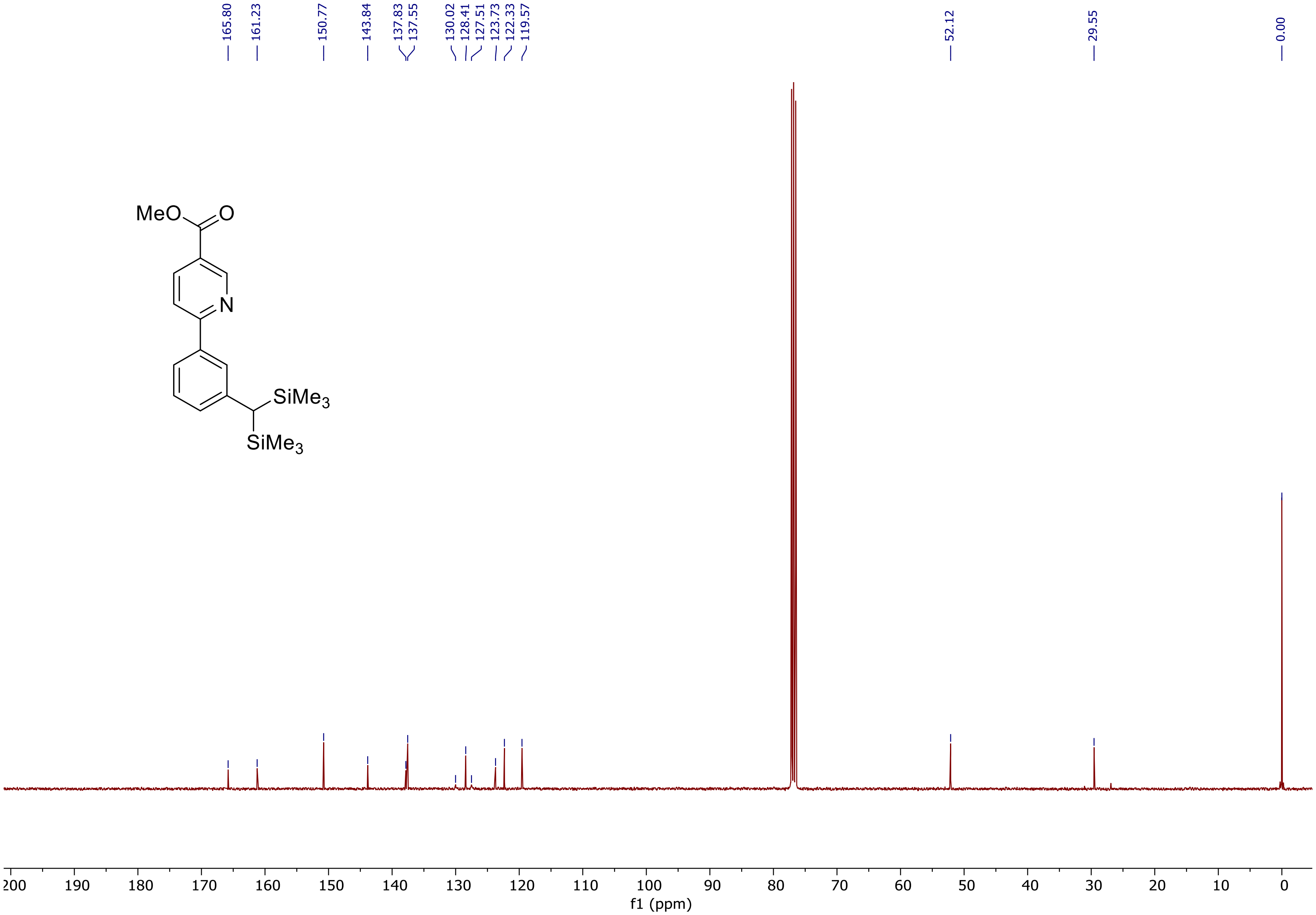

Supplementary Figure 195. ^{13}C NMR (101 MHz, $CDCl_3$) of methyl 6-{3-[bis(trimethylsilyl)methyl]phenyl}nicotinate **6h**.

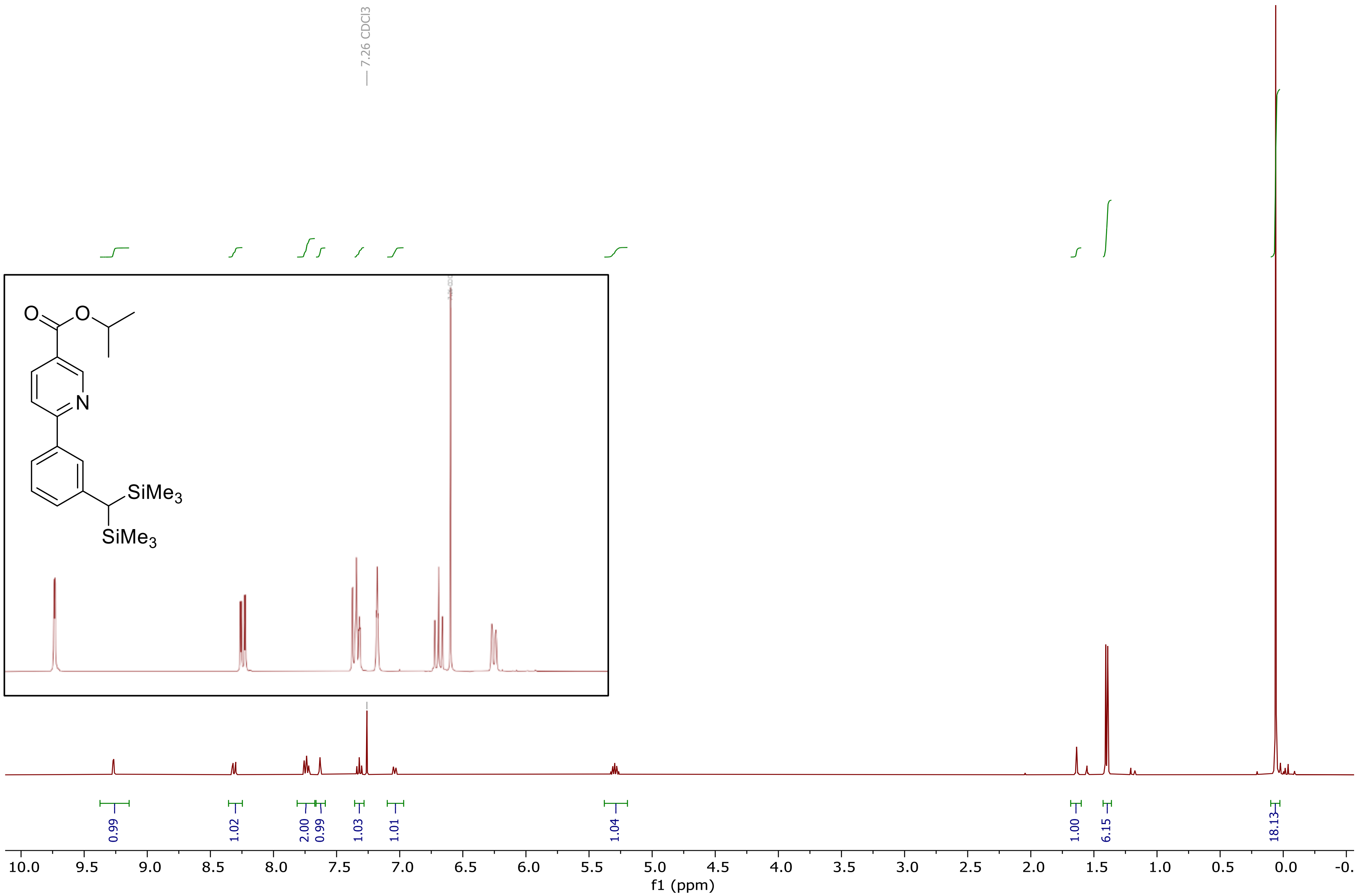

Supplementary Figure 196. ^{1}H NMR (400 MHz, $CDCl_3$) of isopropyl 6-{3-[bis(trimethylsilyl)methyl]phenyl}nicotinate **6i**.

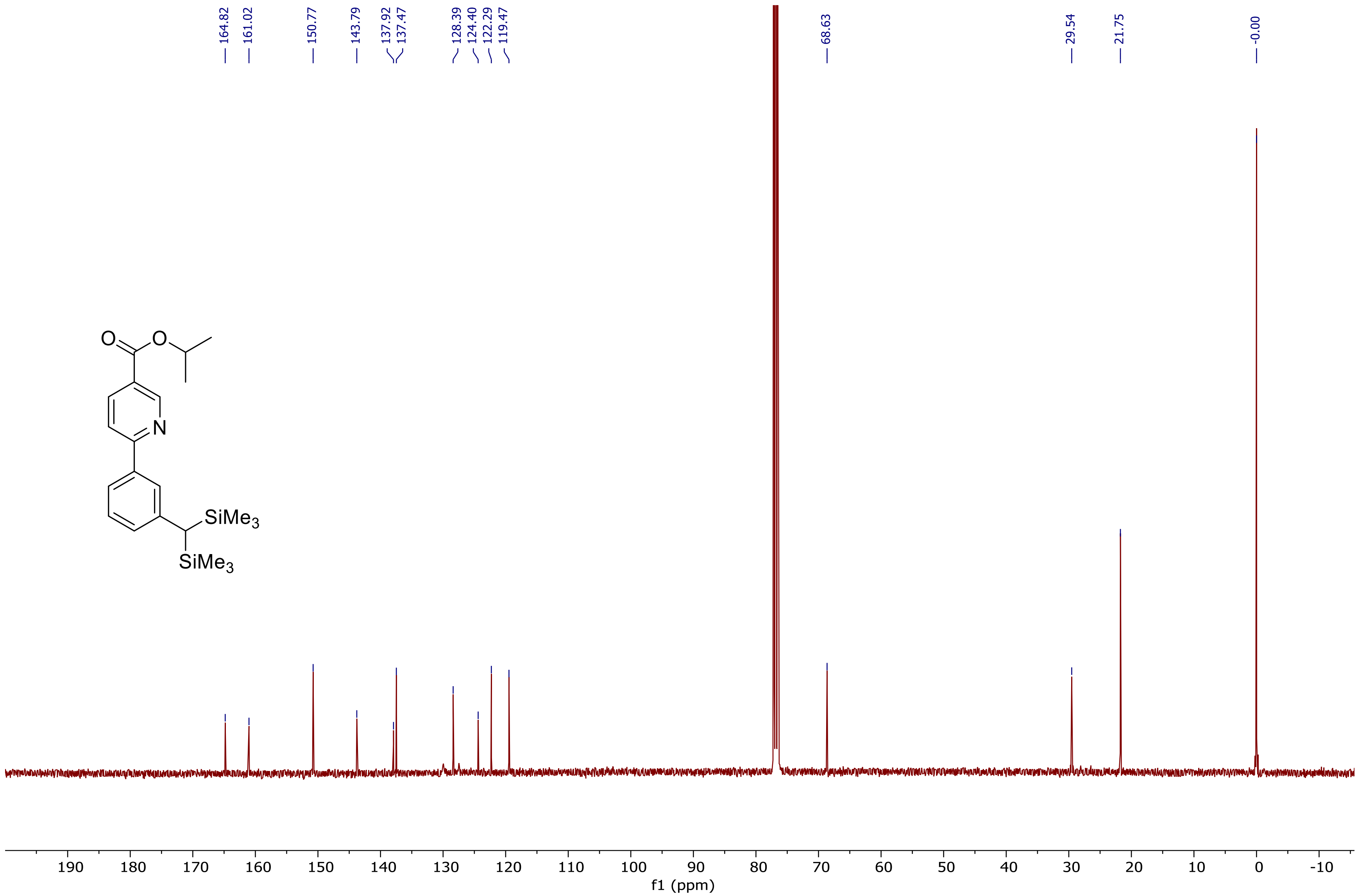

Supplementary Figure 197. ^{13}C NMR (101 MHz, $CDCl_3$) of isopropyl 6-{3-[bis(trimethylsilyl)methyl]phenyl}nicotinate **6i**.

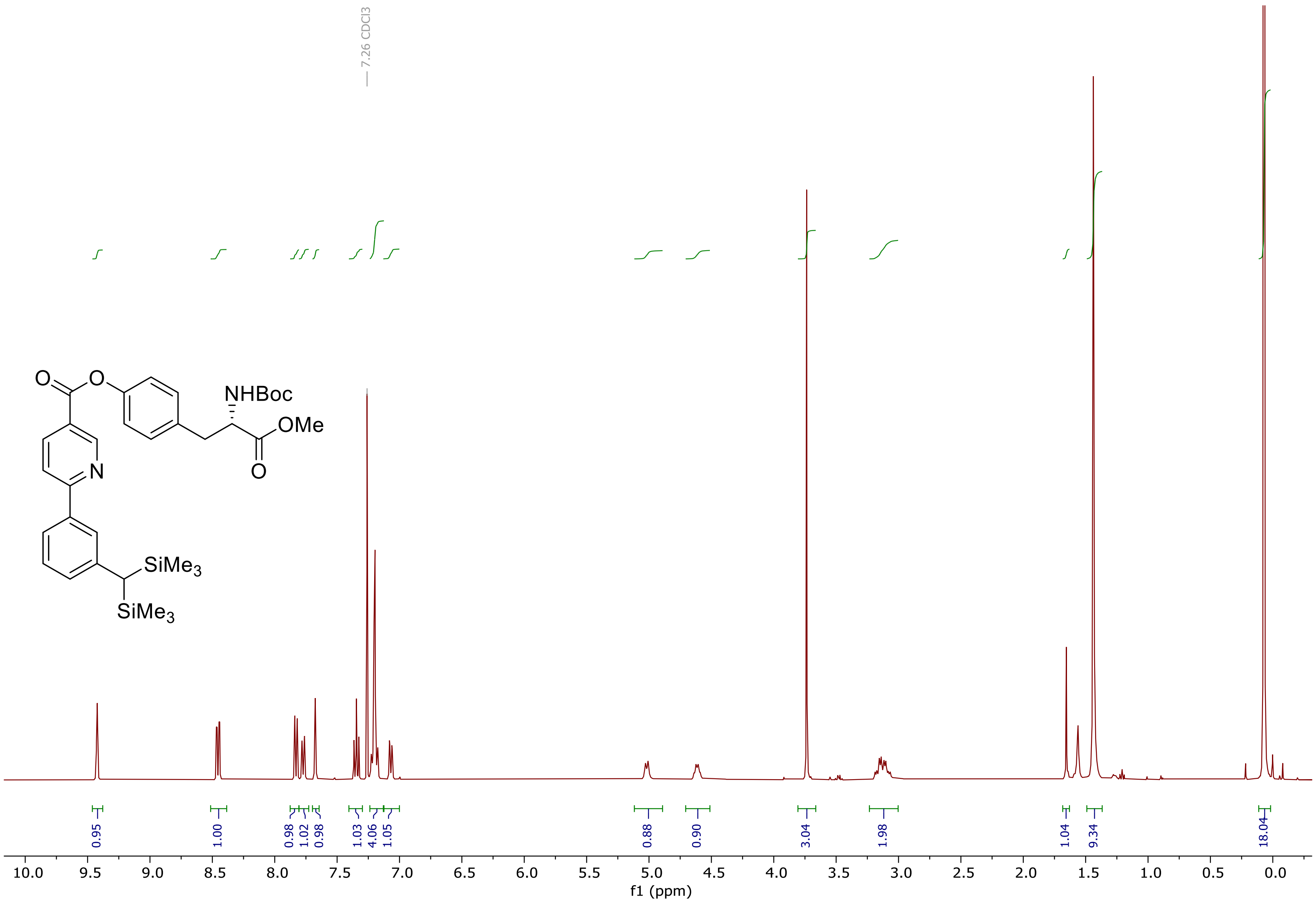

Supplementary Figure 198. ^{1}H NMR (400 MHz, $CDCl_3$) of (*S*)-4-{2-[(*tert*-butoxycarbonyl)amino]-3-methoxy-3-oxopropyl}phenyl 6-{3-[bis(trimethylsilyl)methyl]phenyl}nicotinate **6j**.

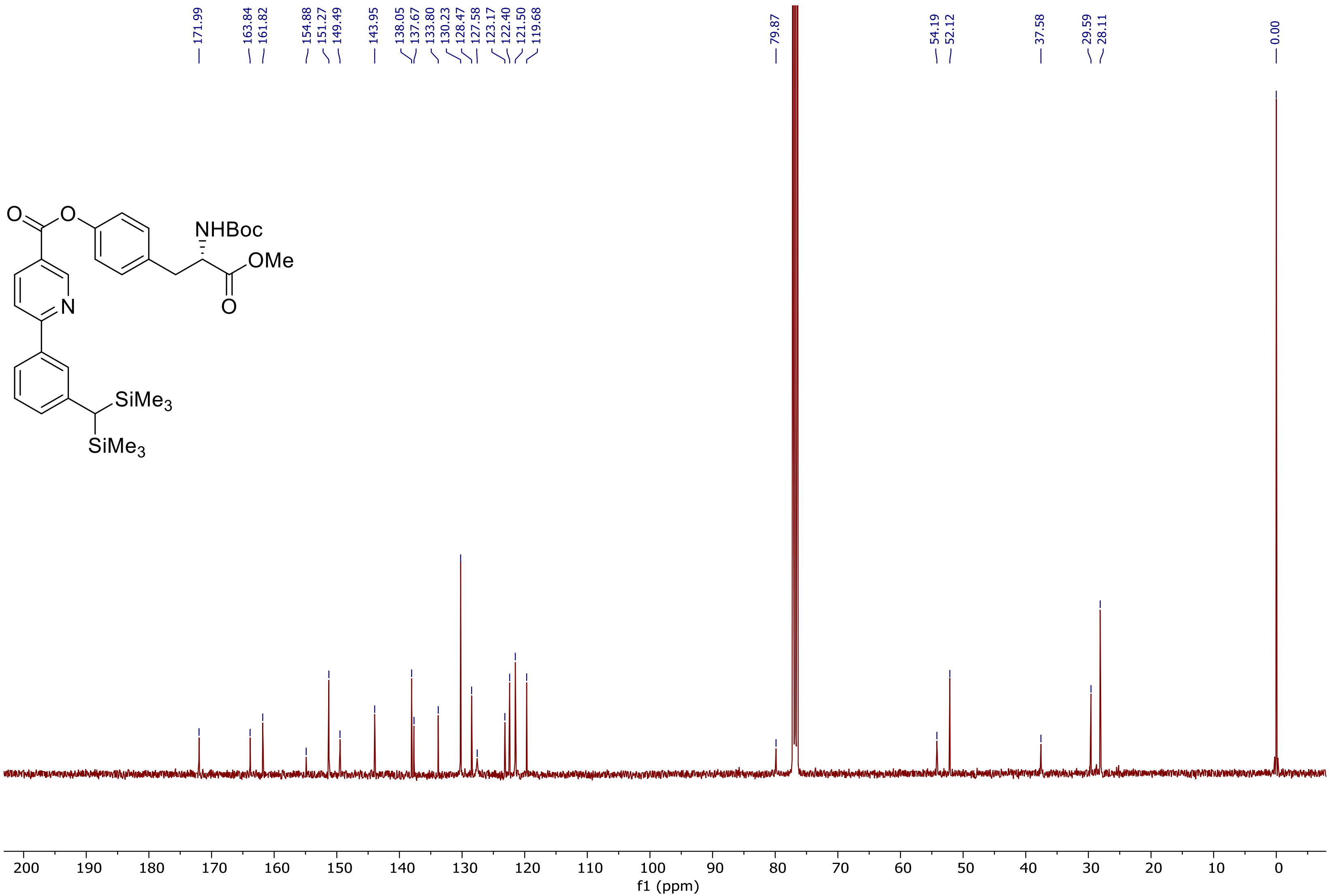

Supplementary Figure 199. ^{13}C NMR (101 MHz, $CDCl_3$) of (*S*)-4-{2-[(*tert*-butoxycarbonyl)amino]-3-methoxy-3-oxopropyl}phenyl 6-{3-[bis(trimethylsilyl)methyl]phenyl}nicotinate **6j**.

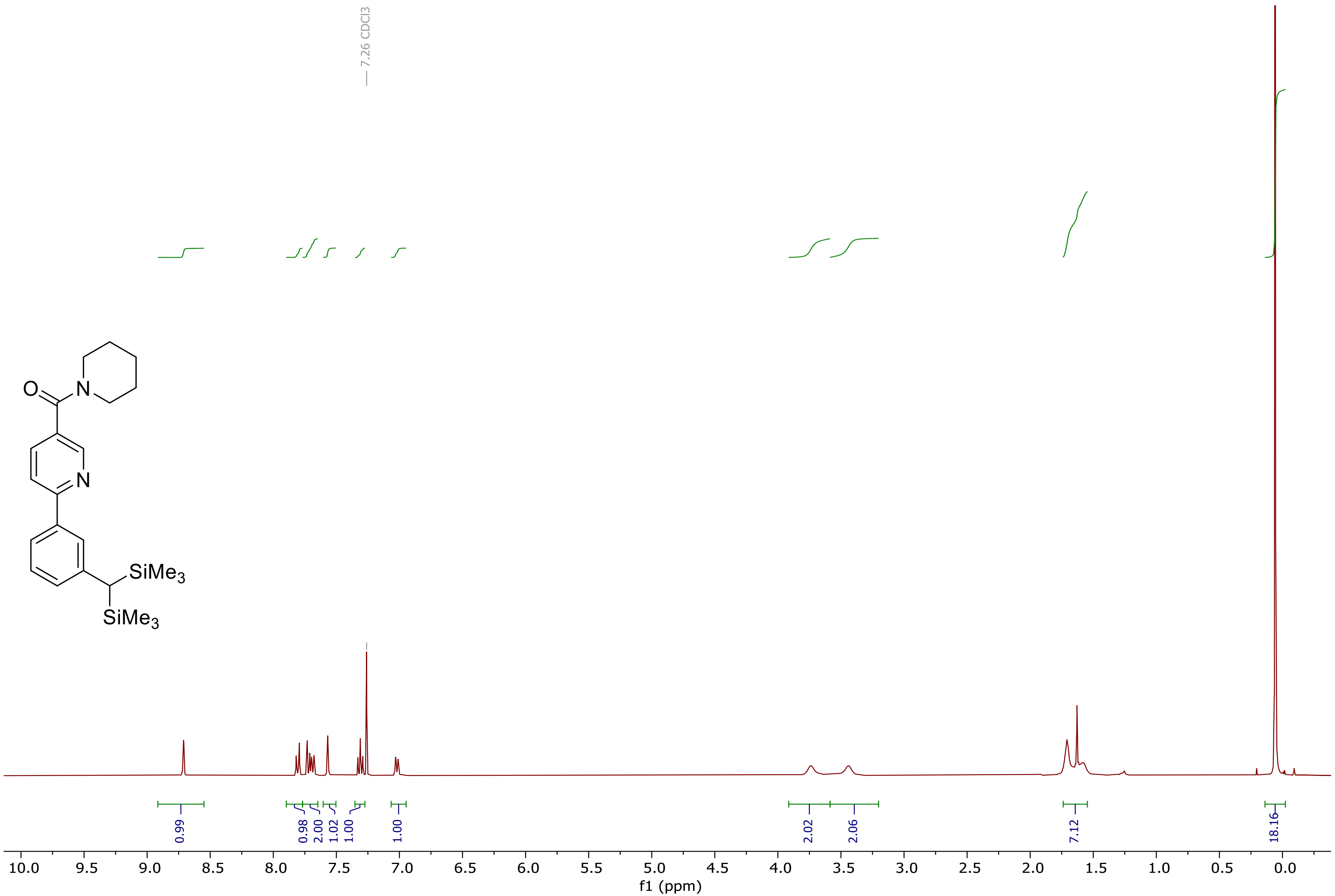

Supplementary Figure 200. ^{1}H NMR (400 MHz, $CDCl_3$) of {6-[3-(bis(trimethylsilyl)methyl)phenyl]pyridin-3-yl}(piperidin-1-yl)methanone **6k**.

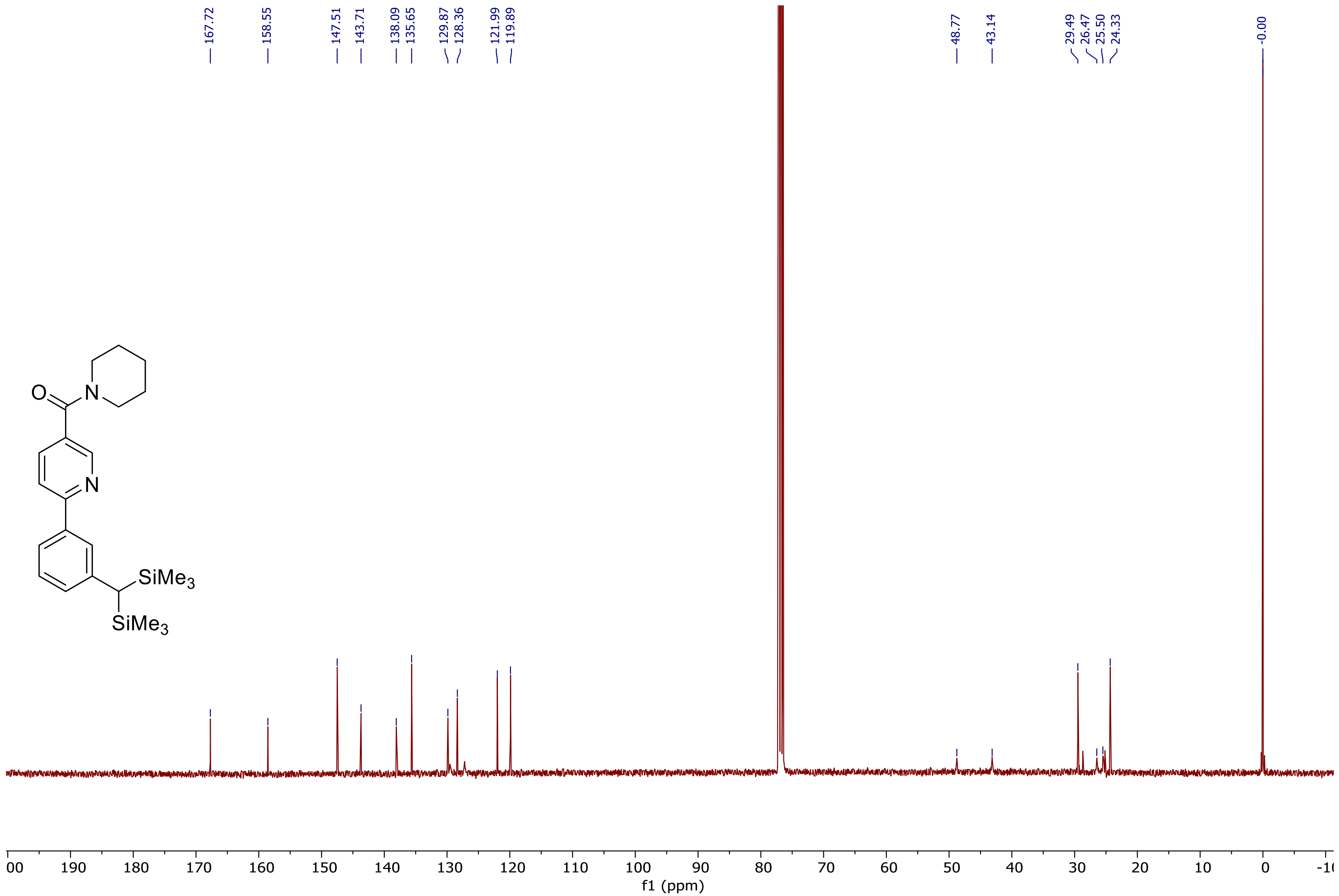

Supplementary Figure 201. ^{13}C NMR (101 MHz, $CDCl_3$) of {6-[3-(bis(trimethylsilyl)methyl)phenyl]pyridin-3-yl}(piperidin-1-yl)methanone **6k.**

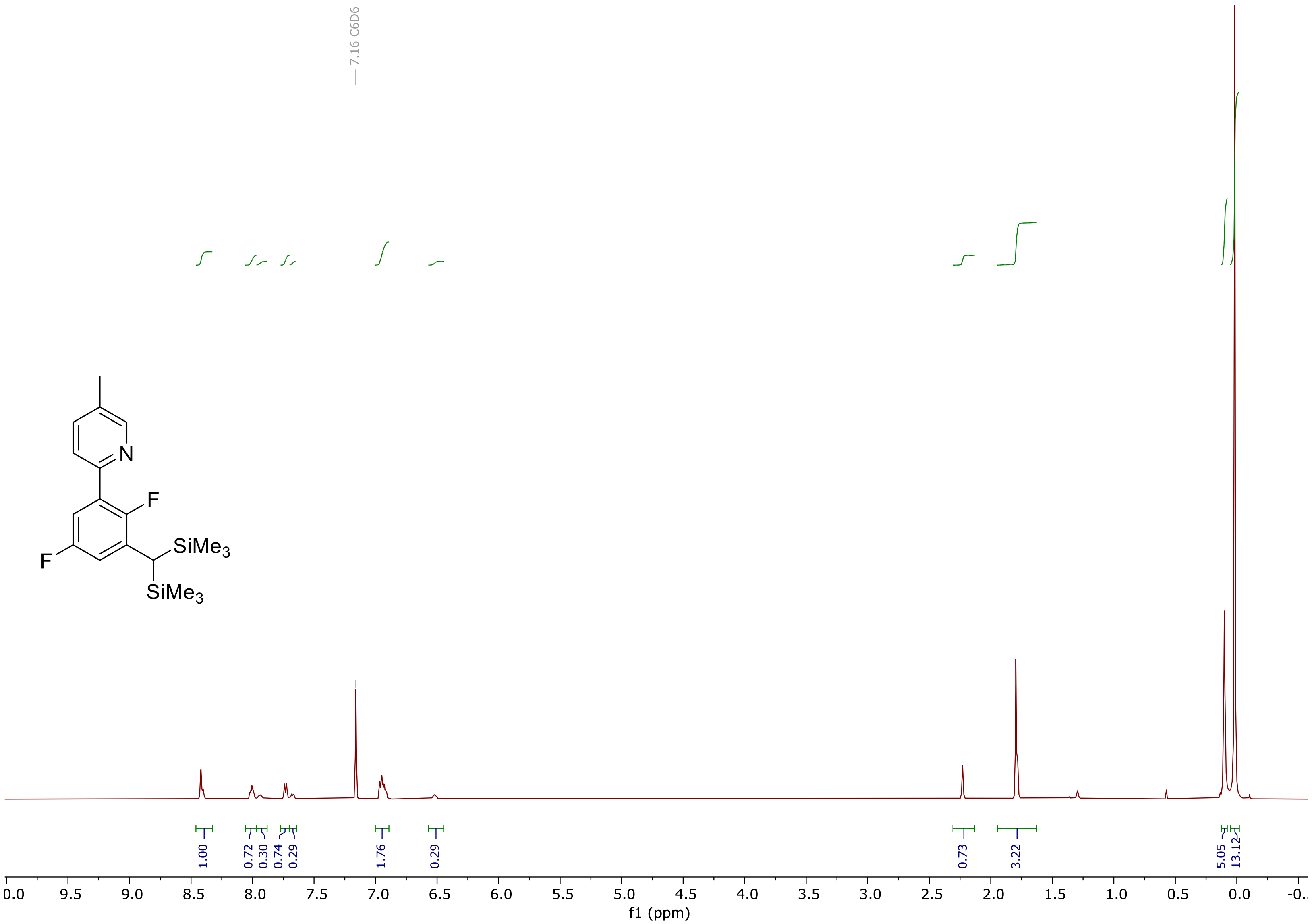

Supplementary Figure 202. ^{1}H NMR (500 MHz, C_6D_6) of 2-{3-[bis(trimethylsilyl)methyl]-2,5-difluorophenyl}-5-methylpyridine **6l** (rotameric).

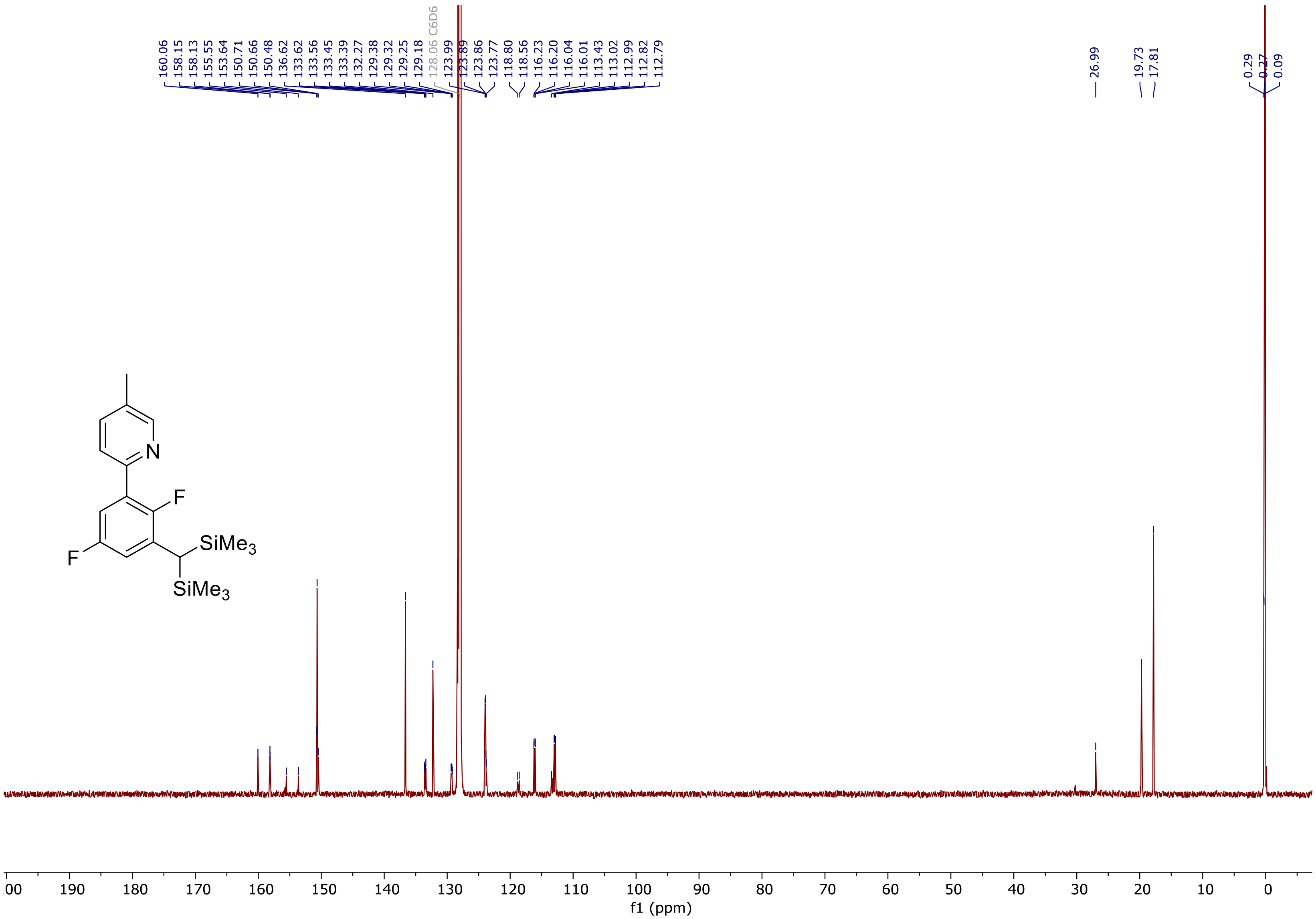

Supplementary Figure 203. ^{13}C NMR (126 MHz, C_6D_6) of 2-{3-[bis(trimethylsilyl)methyl]-2,5-difluorophenyl}-5-methylpyridine **6l** (rotameric).

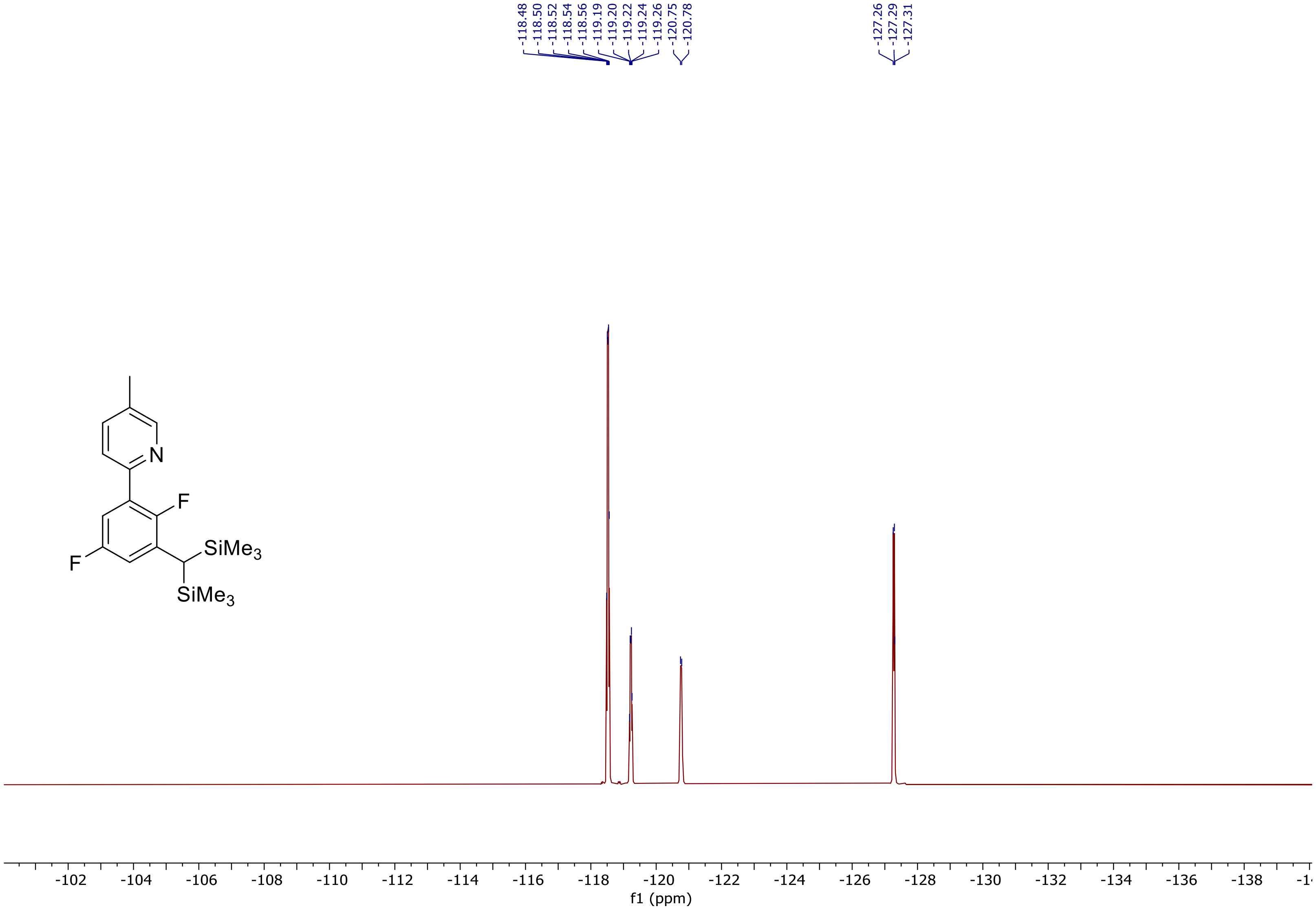

Supplementary Figure 204. ^{19}F NMR (471 MHz, C_6D_6) of 2-{3-[bis(trimethylsilyl)methyl]-2,5-difluorophenyl}-5-methylpyridine **6l** (rotameric).

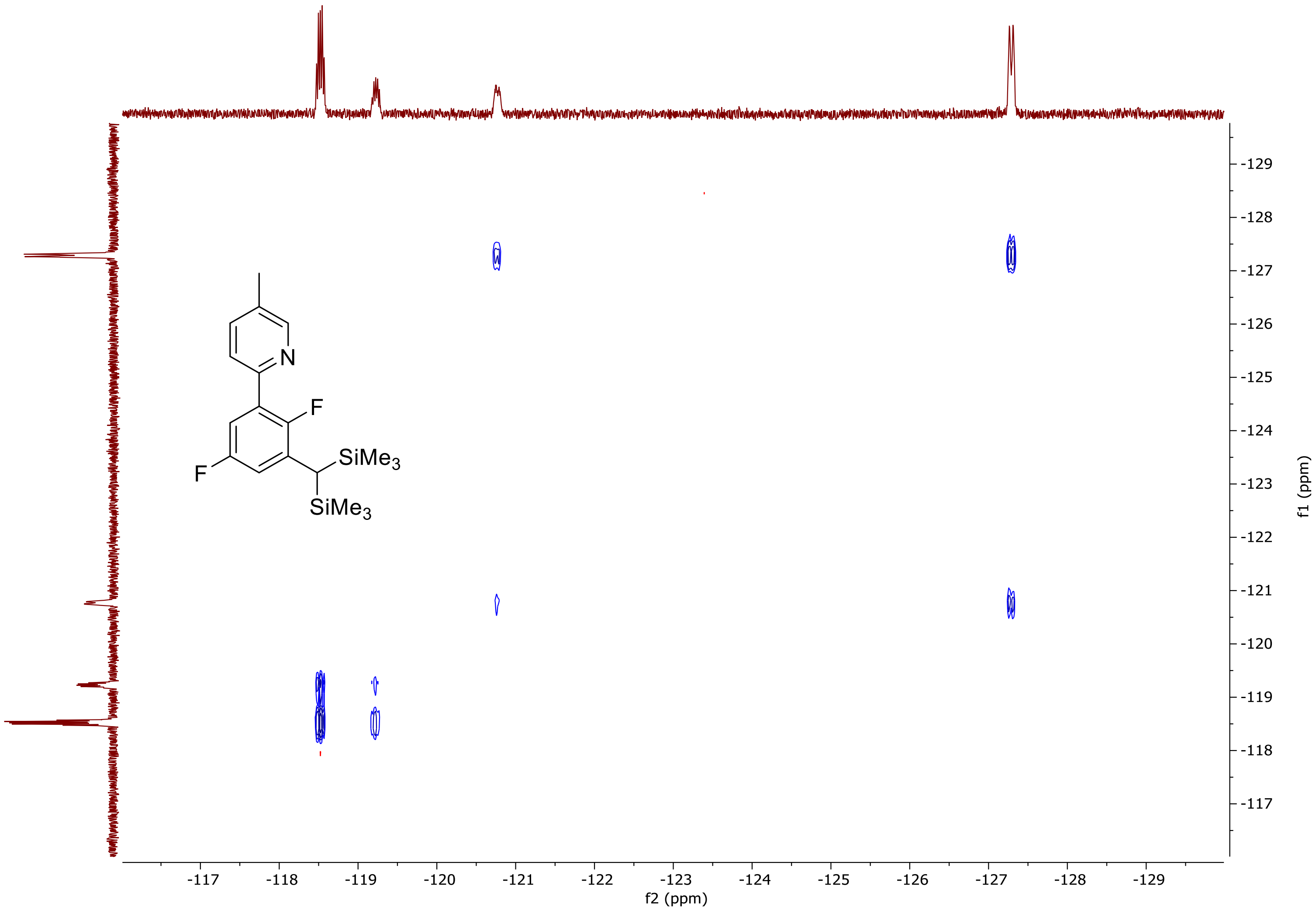

Supplementary Figure 205. ^{19}F – ^{19}F NOESY NMR (376 MHz, C_6D_6) of 2-{3-[bis(trimethylsilyl)methyl]-2,5-difluorophenyl}-5-methylpyridine **6l** (rotameric).

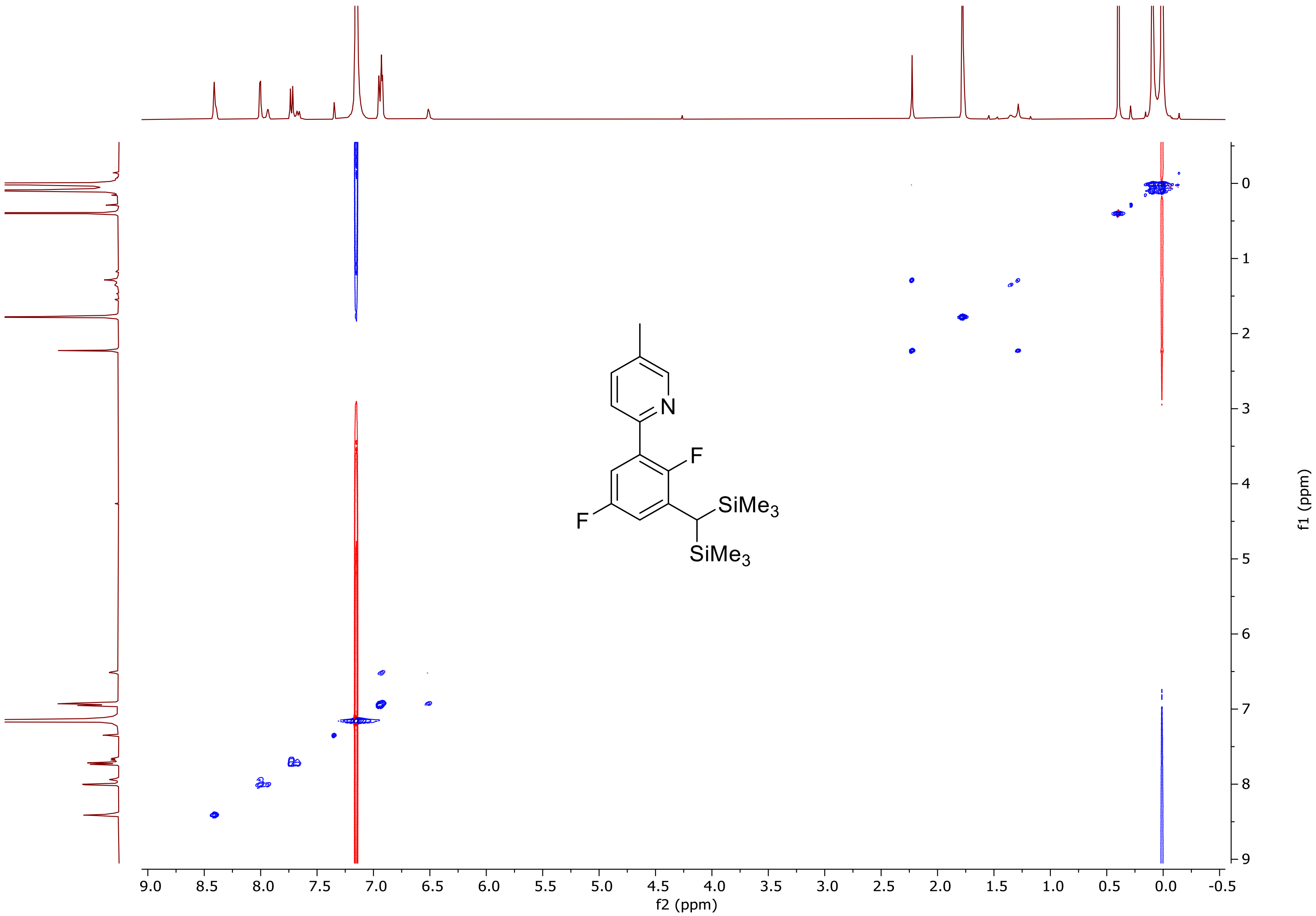

Supplementary Figure 206. ^{1}H – ^{1}H NOESY NMR (400 MHz, C_6D_6) of 2-{3-[bis(trimethylsilyl)methyl]-2,5-difluorophenyl}-5-methylpyridine 6l (rotameric).

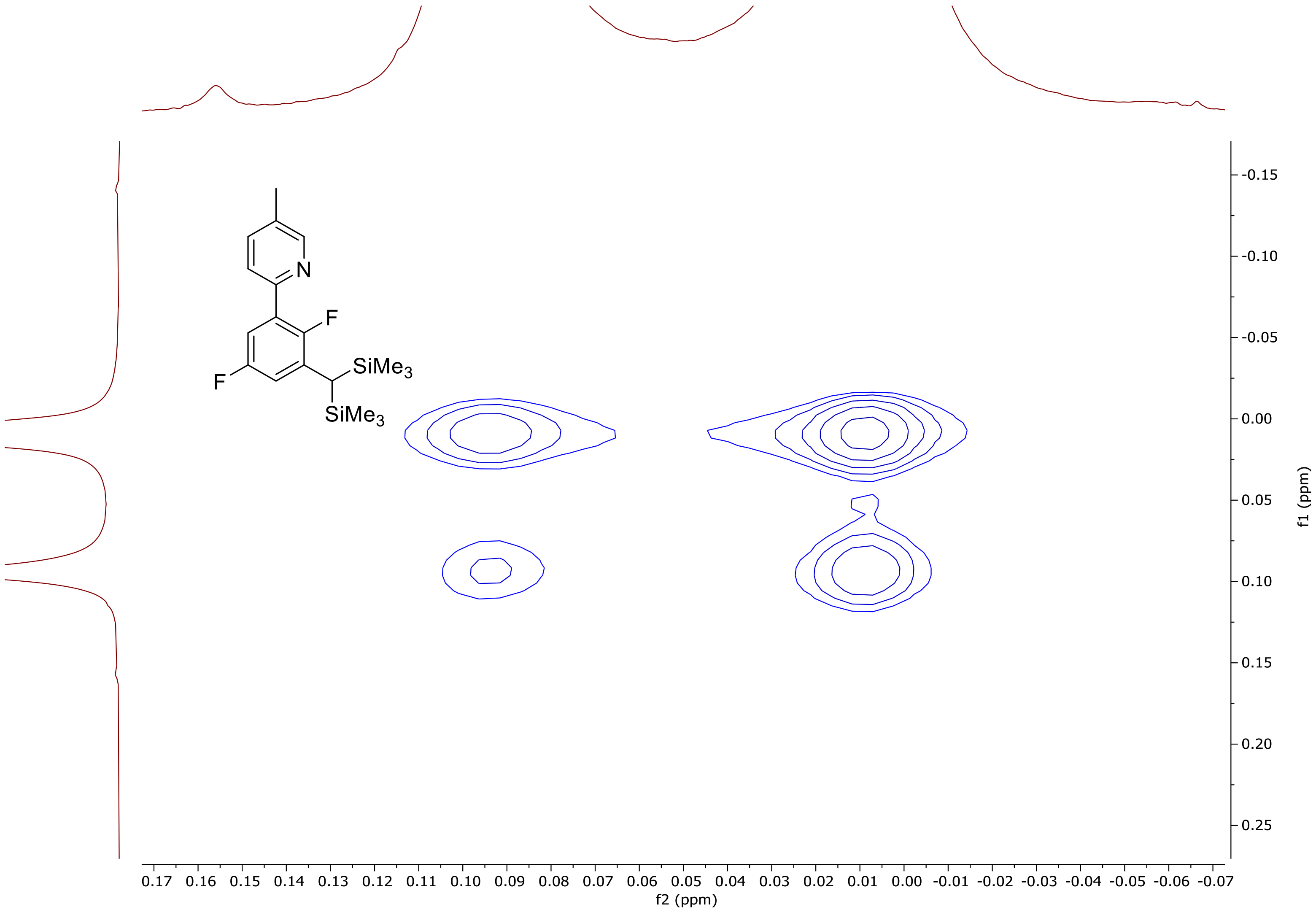

Supplementary Figure 207. ^{1}H – ^{1}H NOESY NMR (400 MHz, C_6D_6) of 2-{3-[bis(trimethylsilyl)methyl]-2,5-difluorophenyl}-5-methylpyridine **6l** (rotameric) – magnified TMS exchange signals.

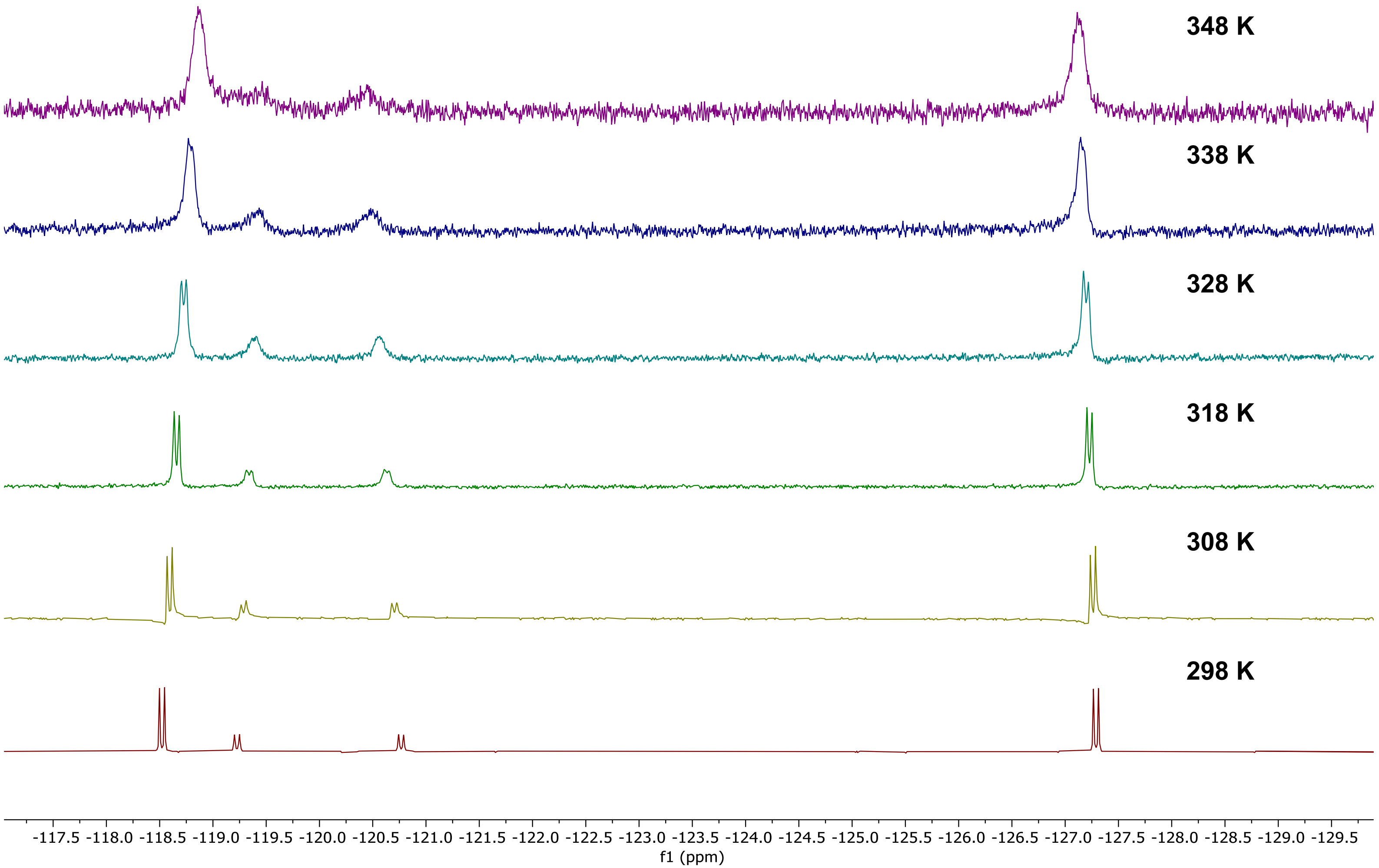

Supplementary Figure 208. ^{19}F Variable Temperature NMR (376 MHz, C_6D_6) of 2-{3-[bis(trimethylsilyl)methyl]-2,5-difluorophenyl}-5-methylpyridine **6l** (rotameric) – 298 K to 348 K.

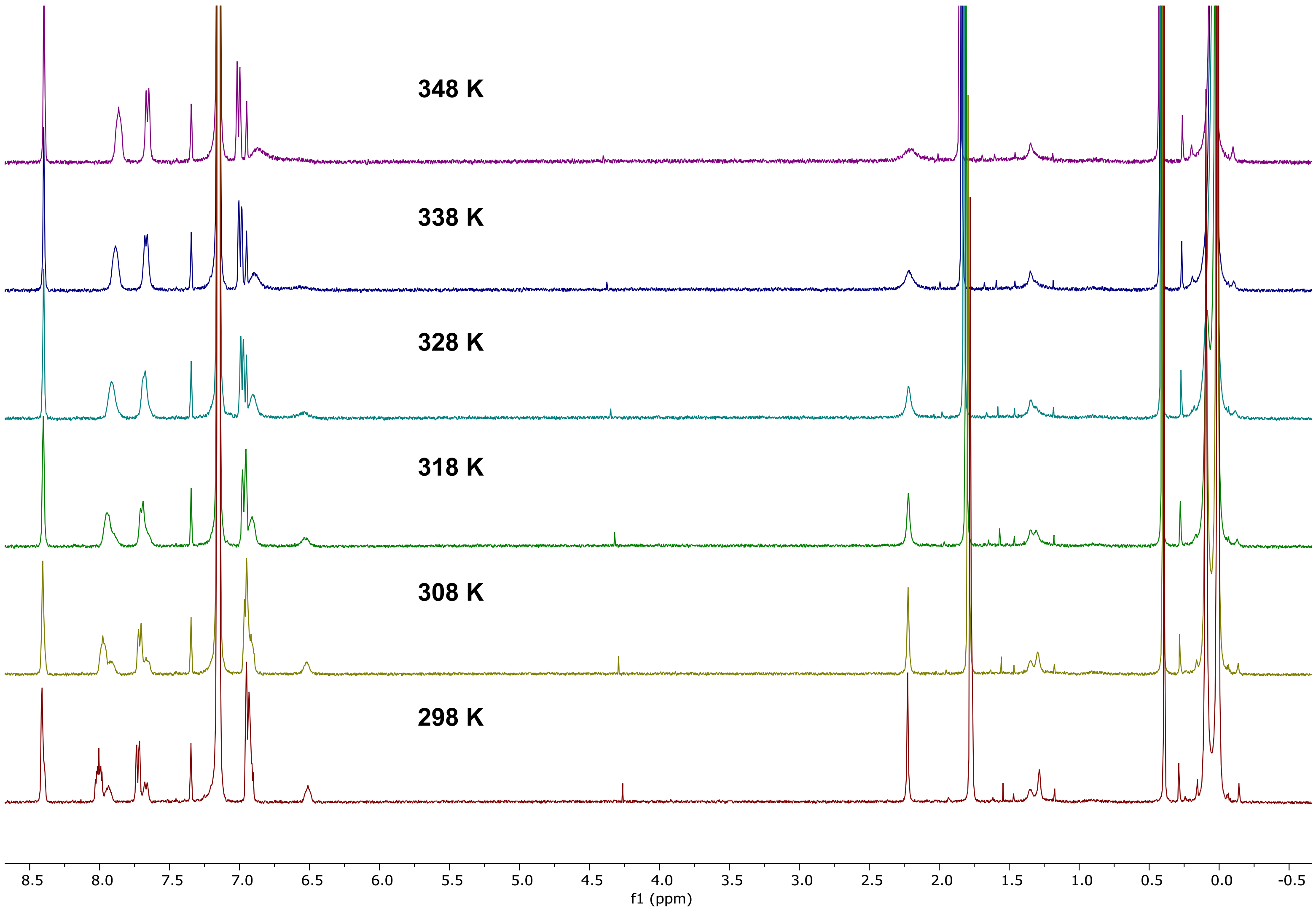

Supplementary Figure 209. ^{1}H Variable Temperature NMR (400 MHz, C_6D_6) of 2-{3-[bis(trimethylsilyl)methyl]-2,5-difluorophenyl}-5-methylpyridine **6l** (rotameric) – 298 K to 348 K.

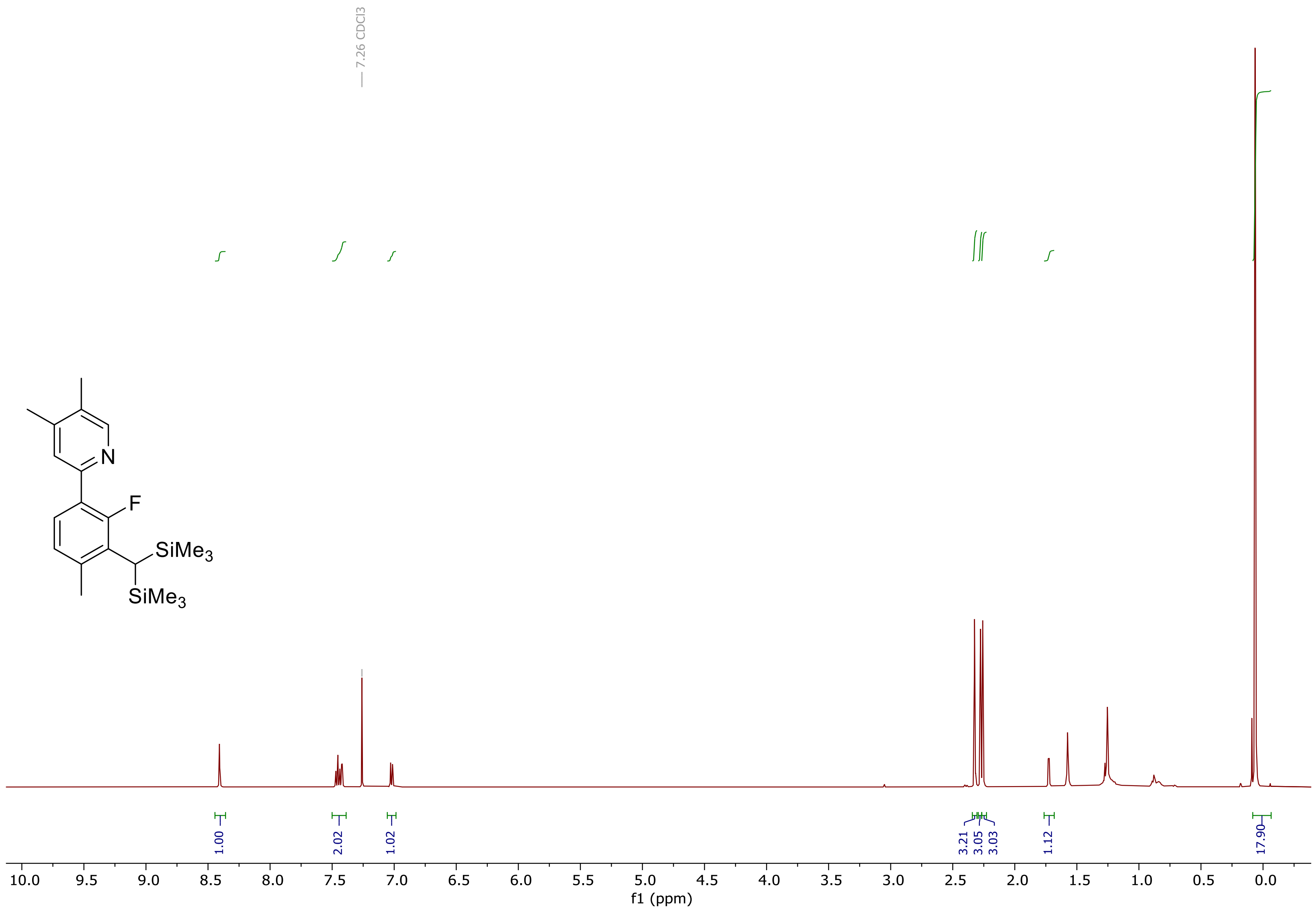

Supplementary Figure 210. ^{1}H NMR (500 MHz, $CDCl_3$) of 2-{3-[bis(trimethylsilyl)methyl]-2-fluoro-4-methylphenyl}-4,5-dimethylpyridine **6m.**

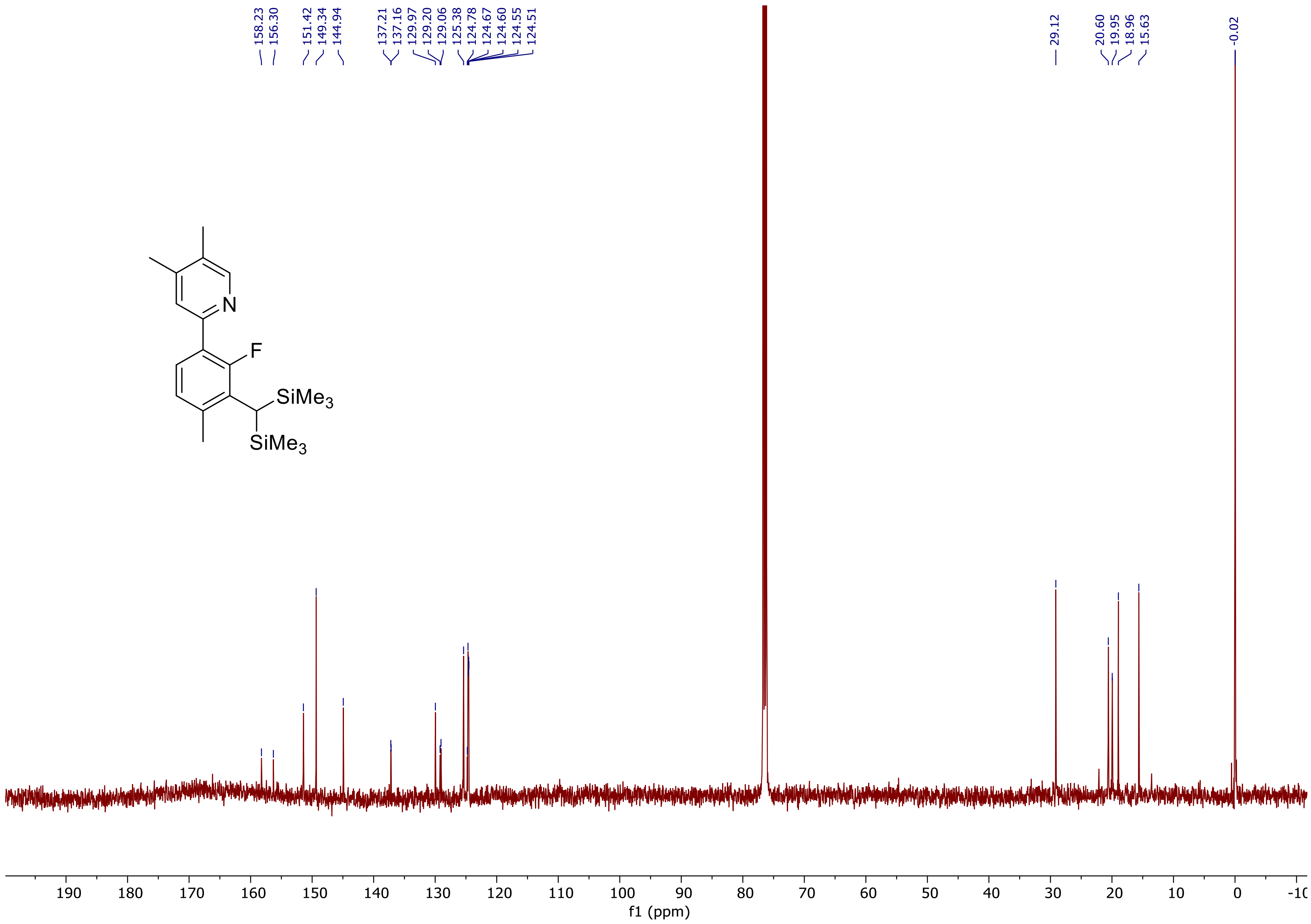

Supplementary Figure 211. ^{13}C NMR (126 MHz, $CDCl_3$) of 2-{3-[bis(trimethylsilyl)methyl]-2-fluoro-4-methylphenyl}-4,5-dimethylpyridine **6m**.

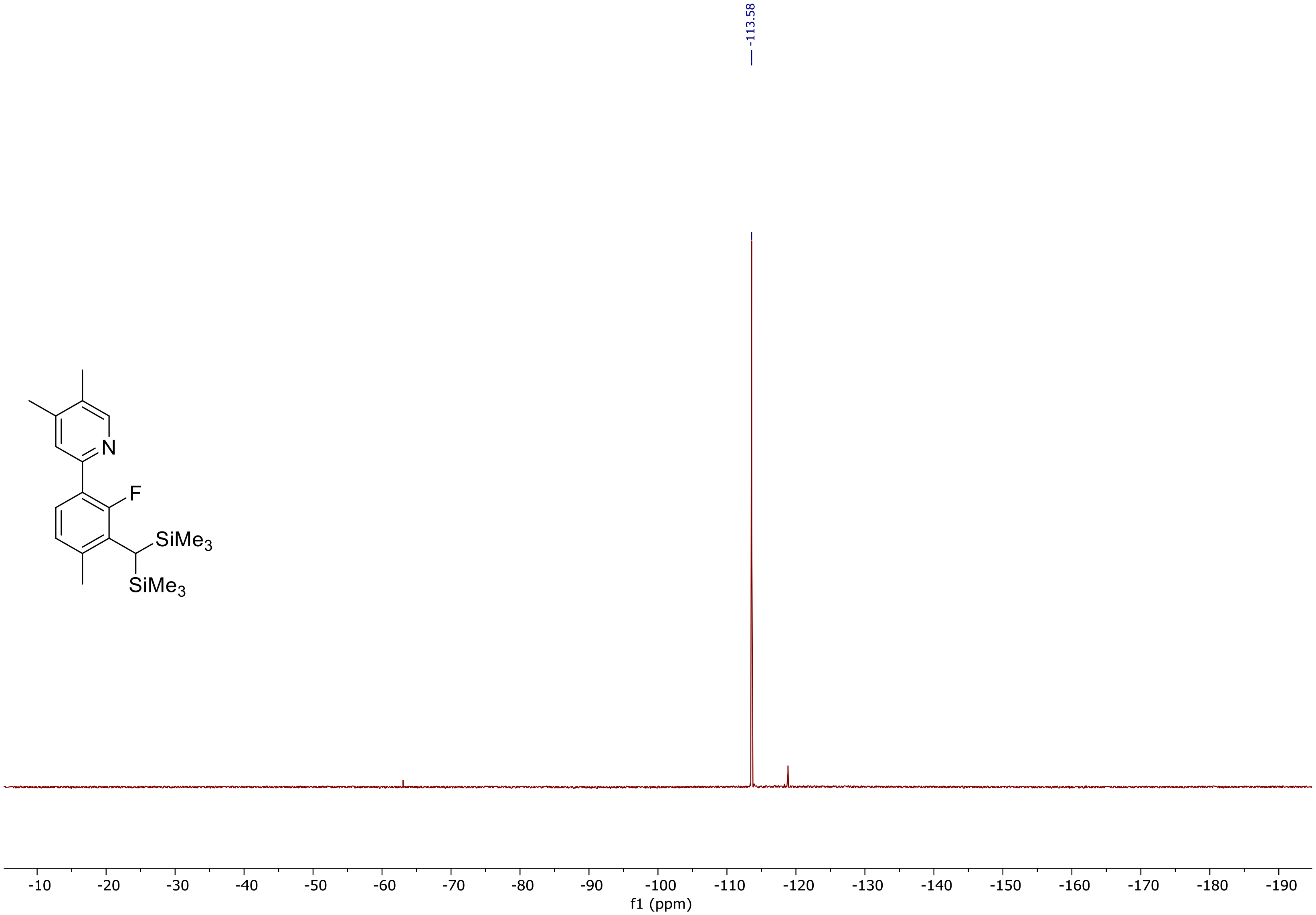

Supplementary Figure 212. ^{19}F NMR (471 MHz, $CDCl_3$) of 2-{3-[bis(trimethylsilyl)methyl]-2-fluoro-4-methylphenyl}-4,5-dimethylpyridine **6m**.

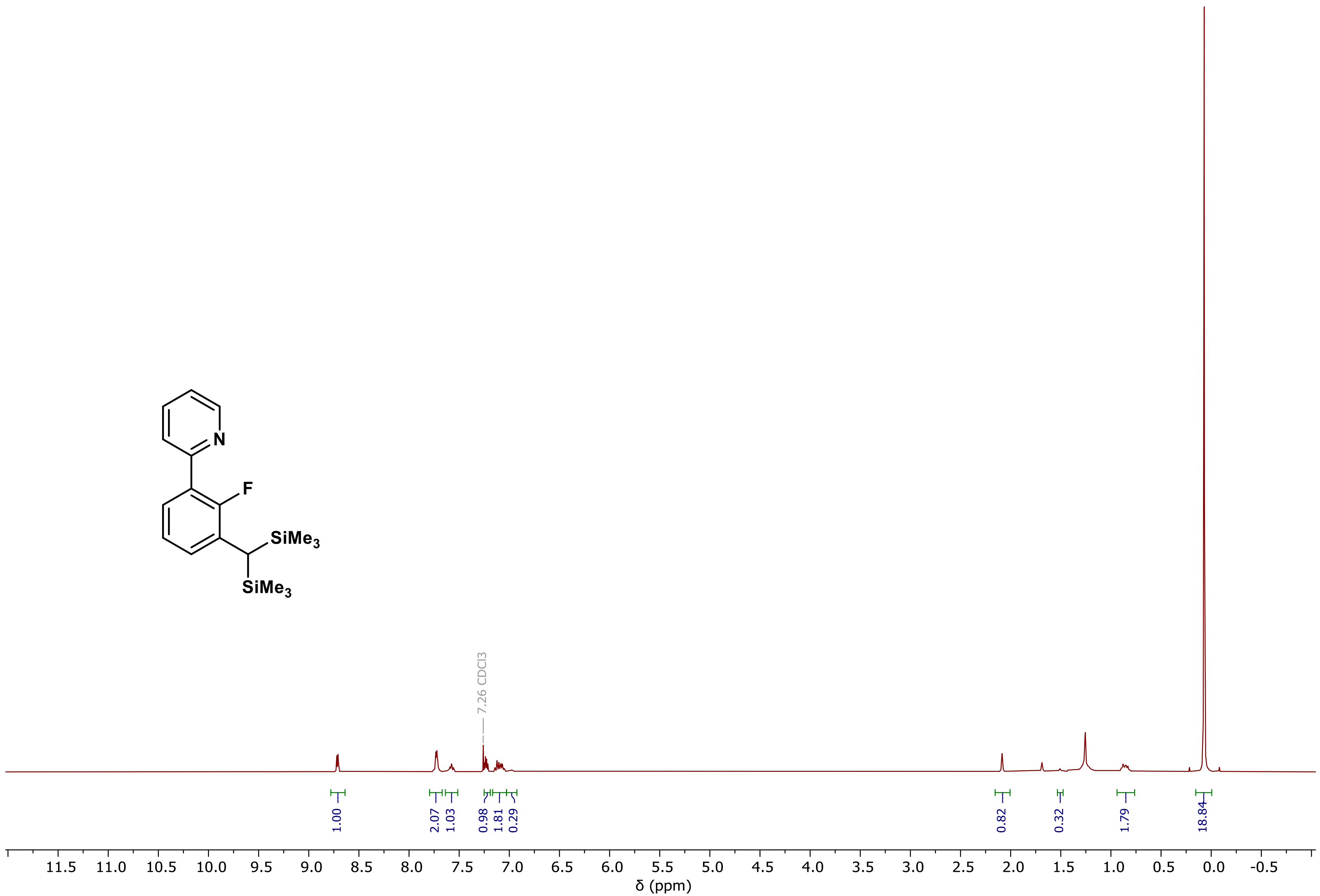

Supplementary Figure 213. ^1H NMR (400 MHz, $CDCl_3$) of 2-{3-[bis(trimethylsilyl)methyl)]2-fluorophenyl}pyridine **6n** (rotameric).

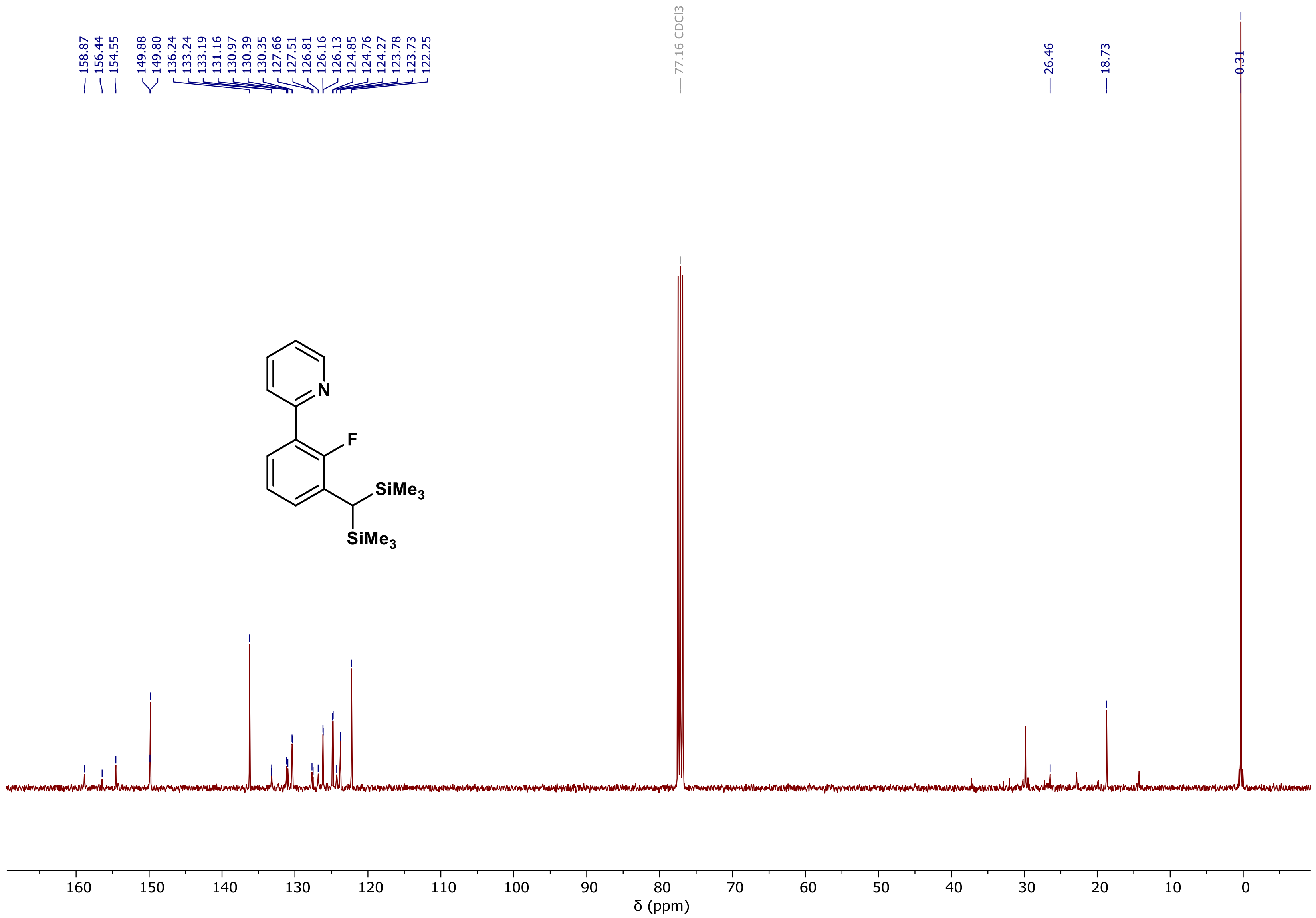

Supplementary Figure 214. ^{13}C NMR (101 MHz, $CDCl_3$) of 2-{3-[bis(trimethylsilyl)methyl]-2-fluorophenyl}pyridine **6n** (rotameric).

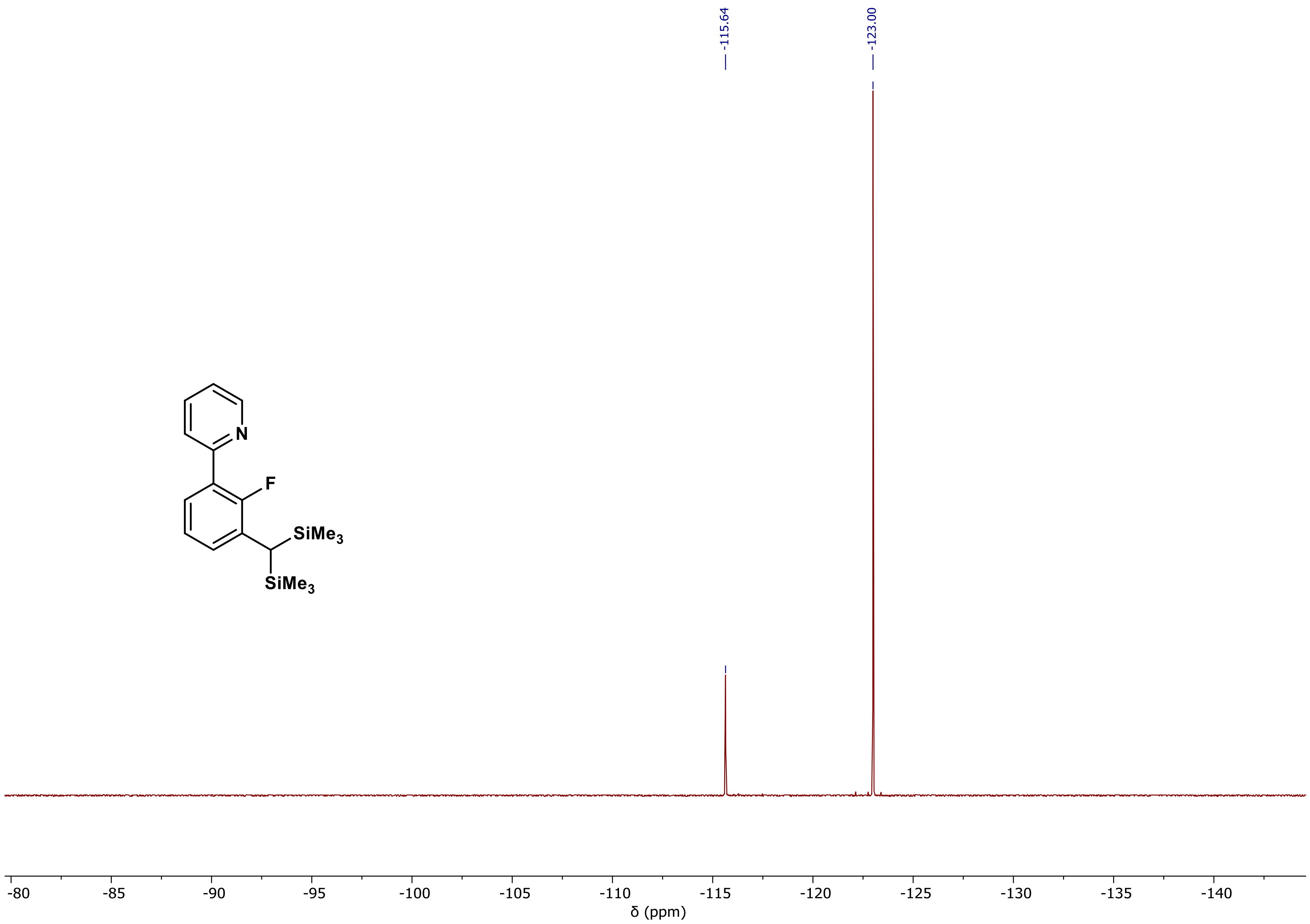

Supplementary Figure 215. ^{19}F NMR (376 MHz, $CDCl_3$) of 2-{3-[bis(trimethylsilyl)methyl]-2-fluorophenyl}pyridine **6n** (rotameric).

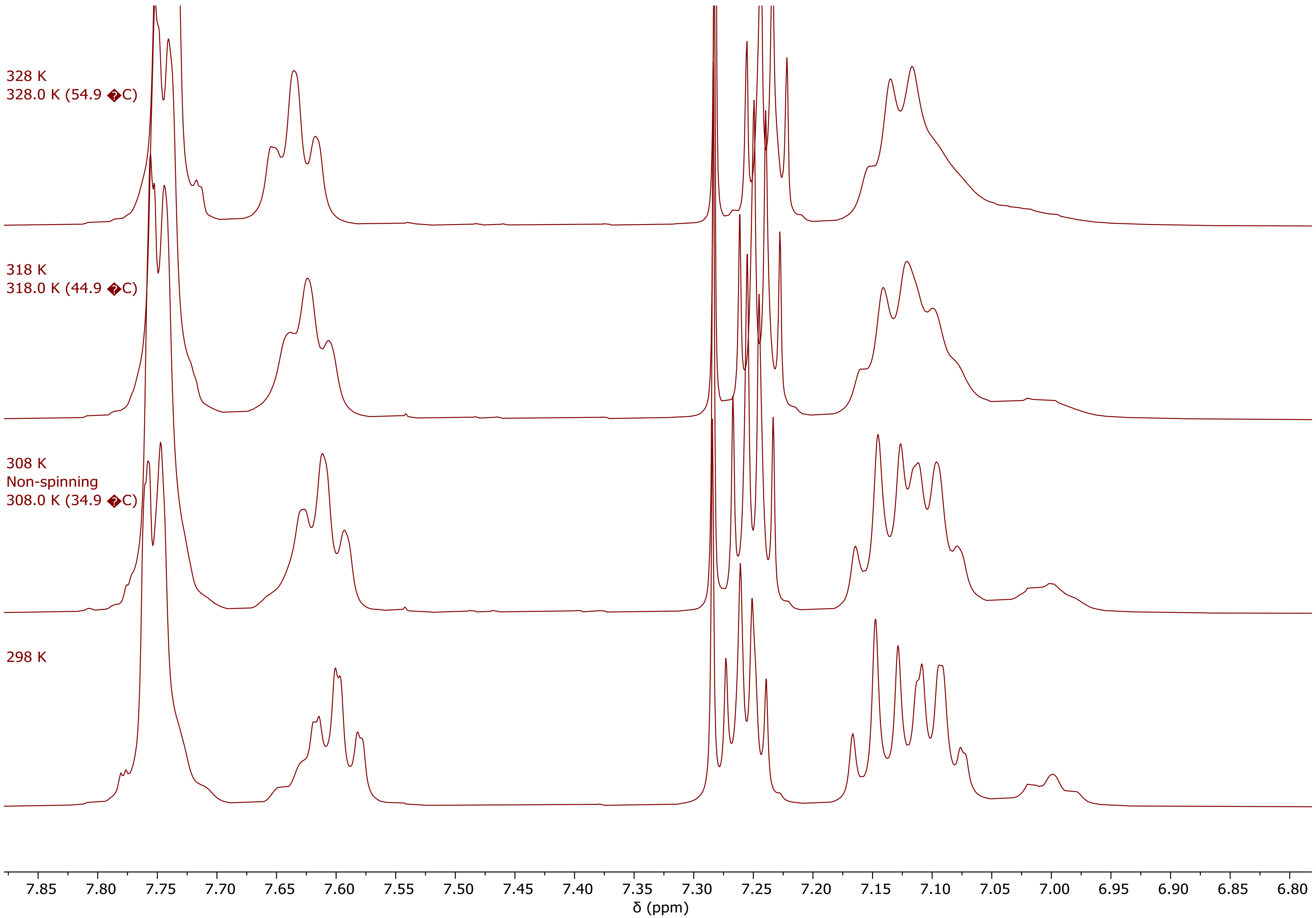

Supplementary Figure 216. ^{1}H Variable Temperature NMR (400 MHz, $CDCl_3$, aromatic region) of 2-{3-[bis(trimethylsilyl)methyl]-2-fluorophenyl}pyridine **6n** (rotameric) – 298 K to 328 K.

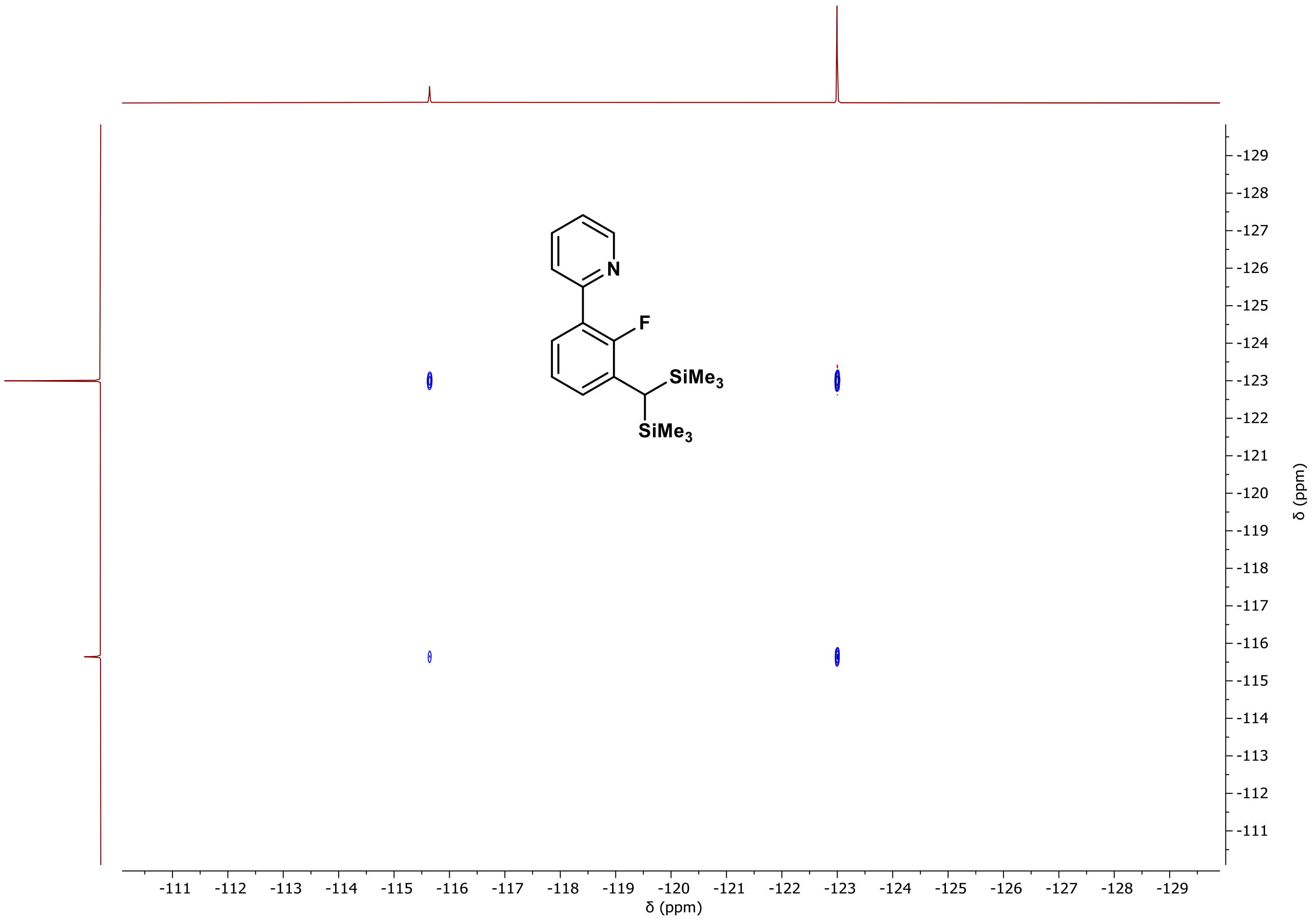

Supplementary Figure 217. $^{19}F - ^{19}F$ NOESY NMR (376 MHz, $CDCl_3$) of 2-{3-[bis(trimethylsilyl)methyl]-2,5-difluorophenyl}-5-methylpyridine **6n** (rotameric)

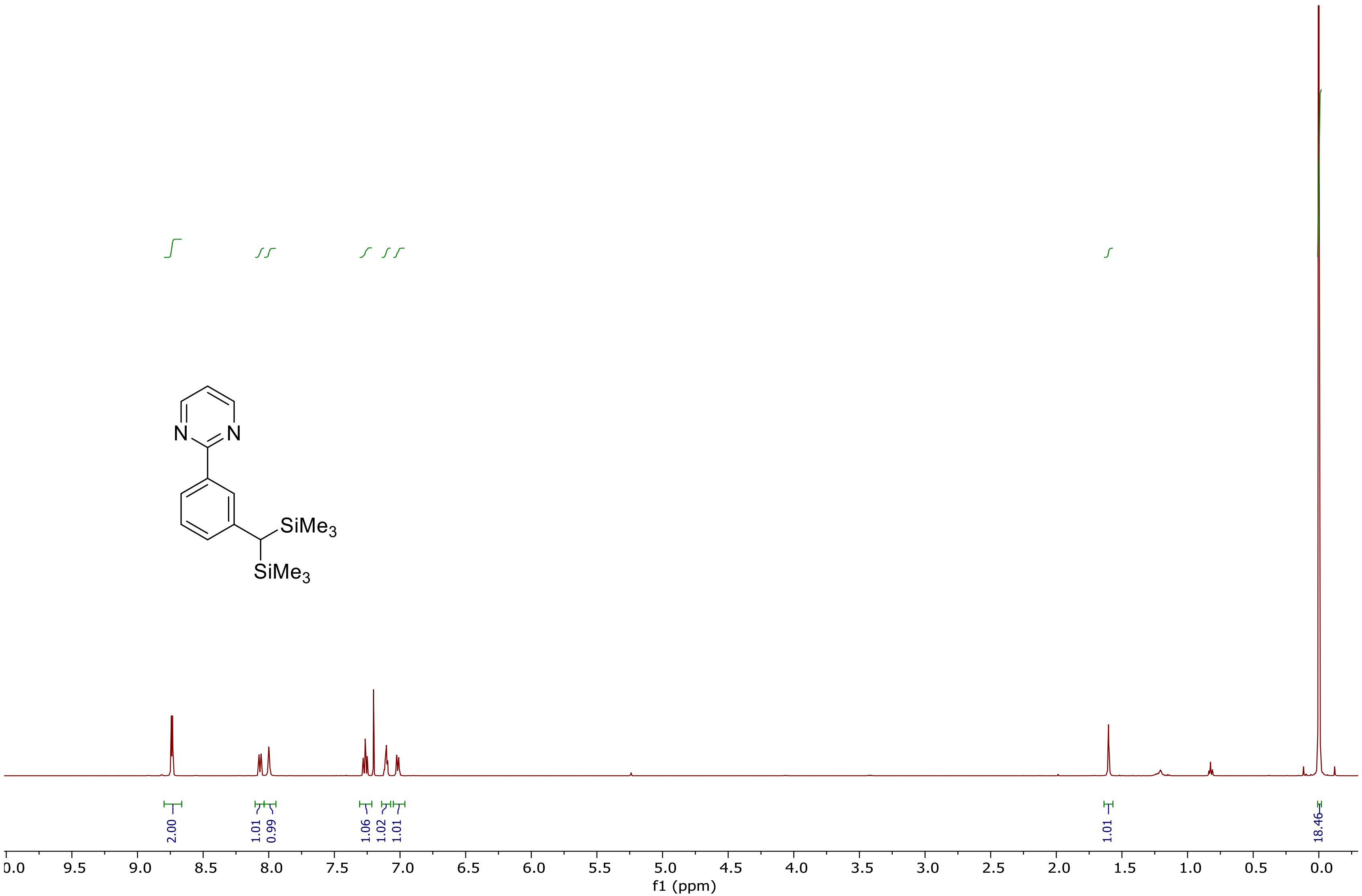

Supplementary Figure 218. ^{1}H NMR (500 MHz, $CDCl_3$) of 2-(3-(bis(trimethylsilyl)methyl)phenyl)pyrimidine **6o**.

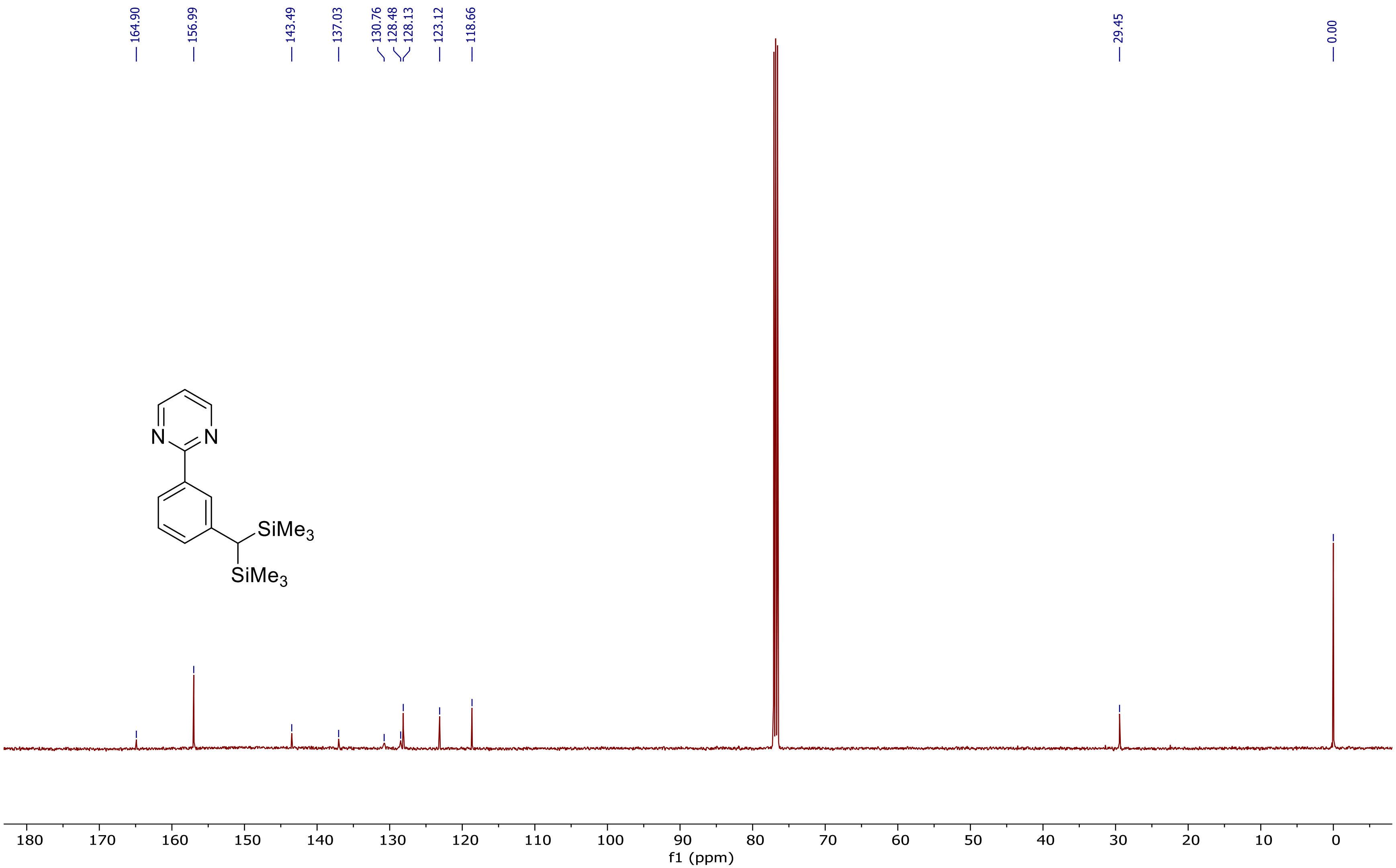

Supplementary Figure 219. ^{13}C NMR (126 MHz, $CDCl_3$) of 2-(3-(bis(trimethylsilyl)methyl)phenyl)pyrimidine **6o**.

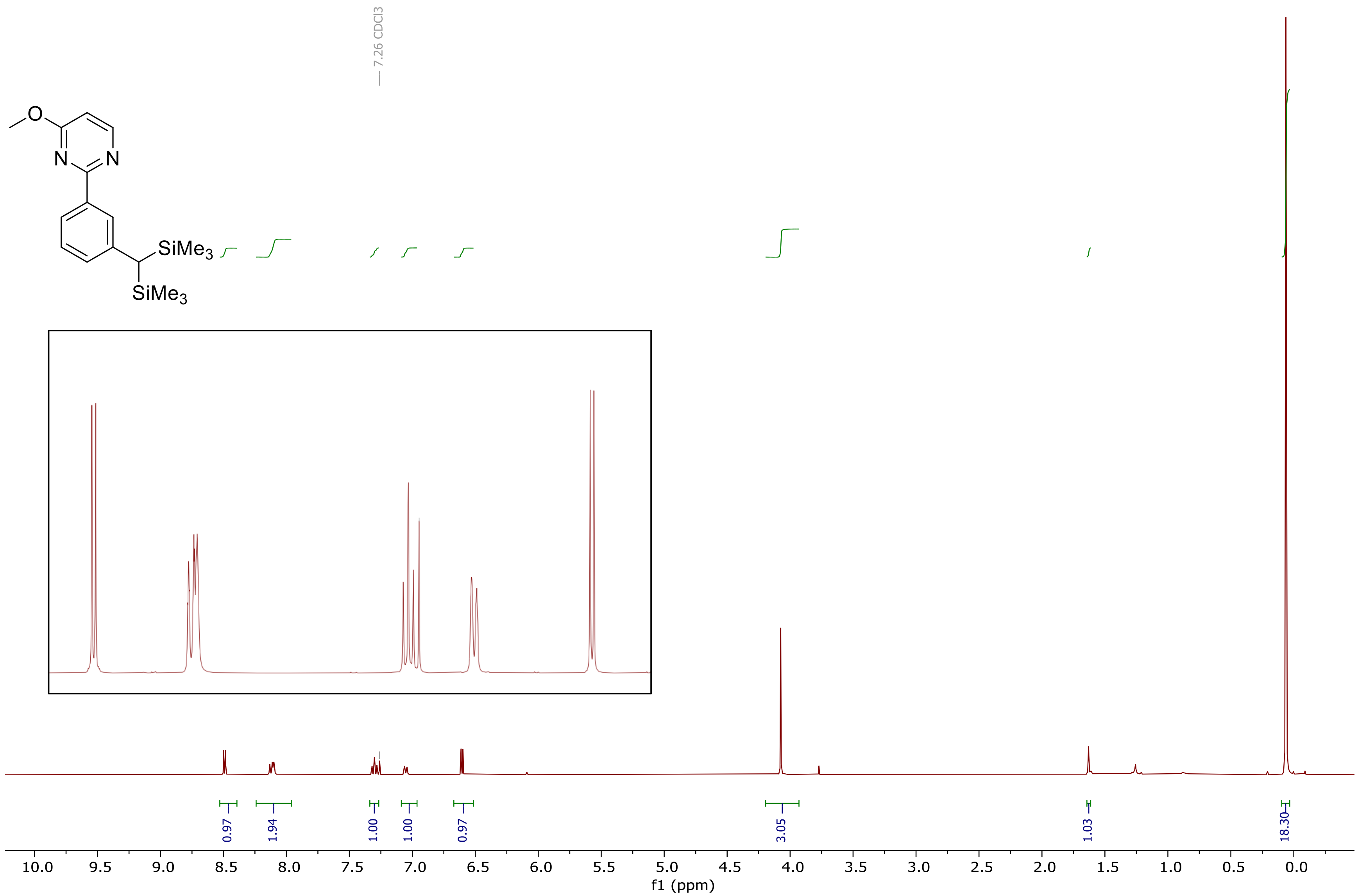

Supplementary Figure 220. ^{1}H NMR (400 MHz, $CDCl_3$) of 2-(3-(bis(trimethylsilyl)methyl)phenyl)-4-methoxypyrimidine **6p**.

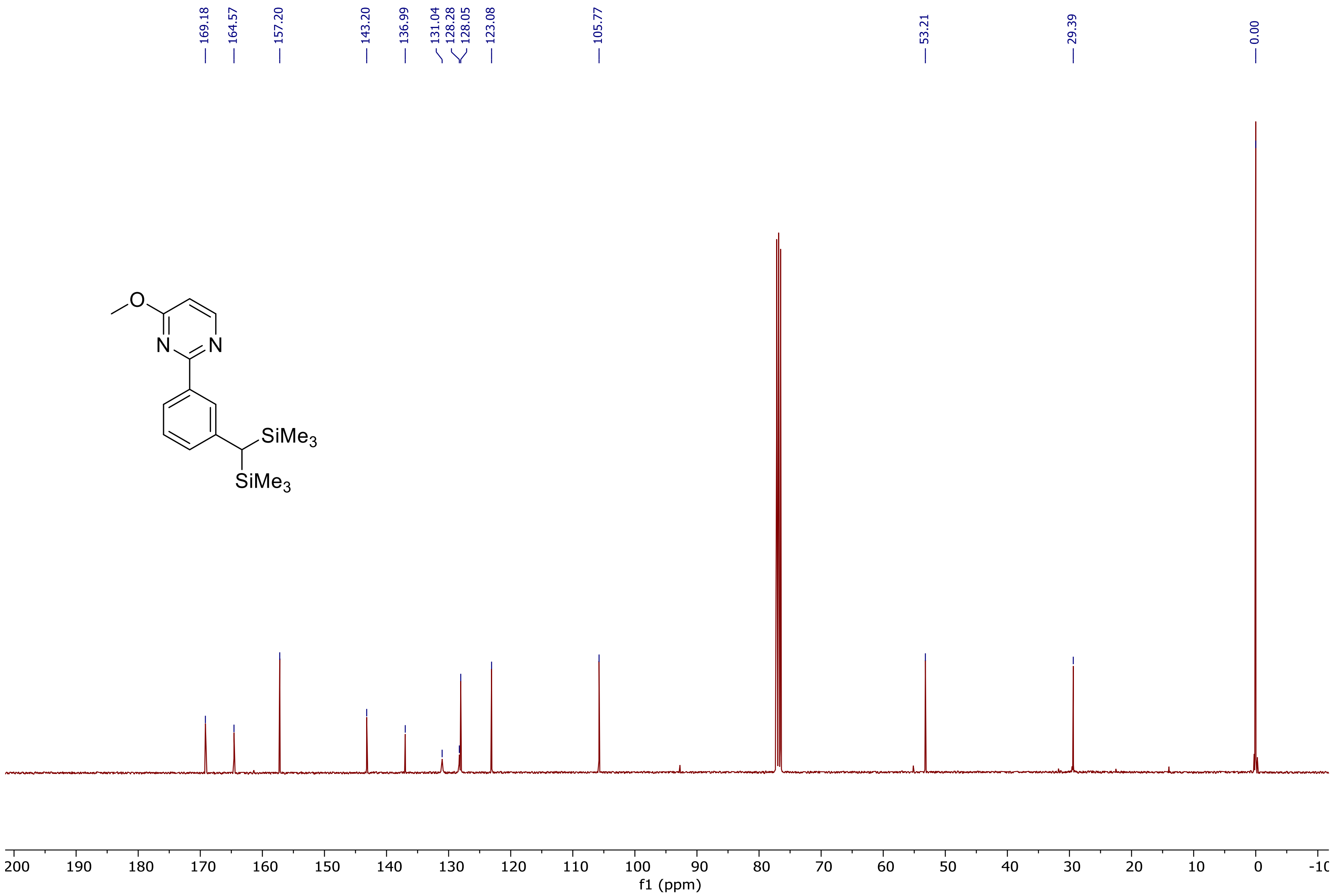

Supplementary Figure 221. ^{13}C NMR (101 MHz, $CDCl_3$) of 2-{3-[bis(trimethylsilyl)methyl]phenyl}-4-methoxypyrimidine **6p**.

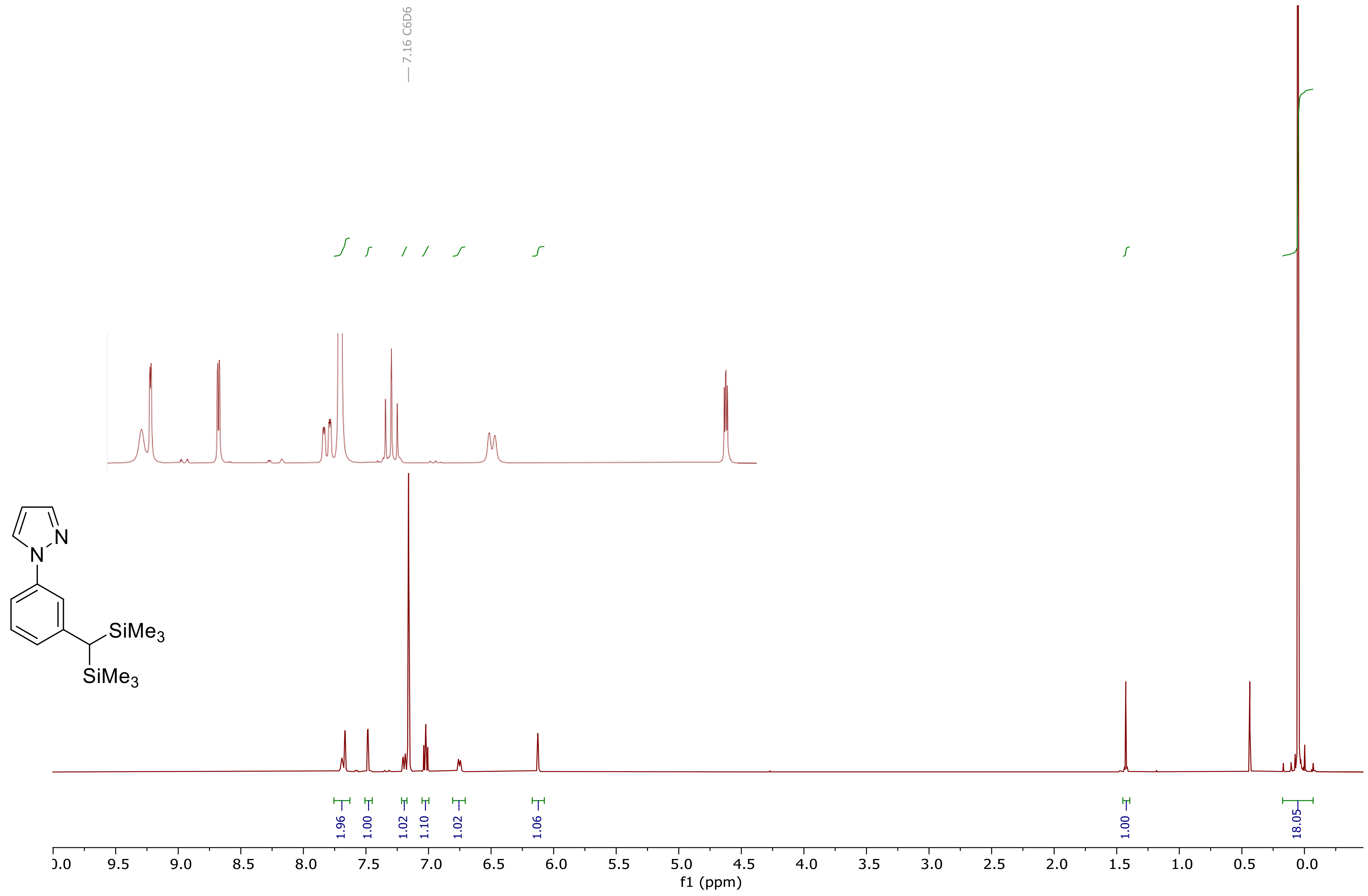

Supplementary Figure 222. ^{1}H NMR (500 MHz, C_6D_6) of 1-{3-[bis(trimethylsilyl)methyl]phenyl}-1H-pyrazole **6q**.

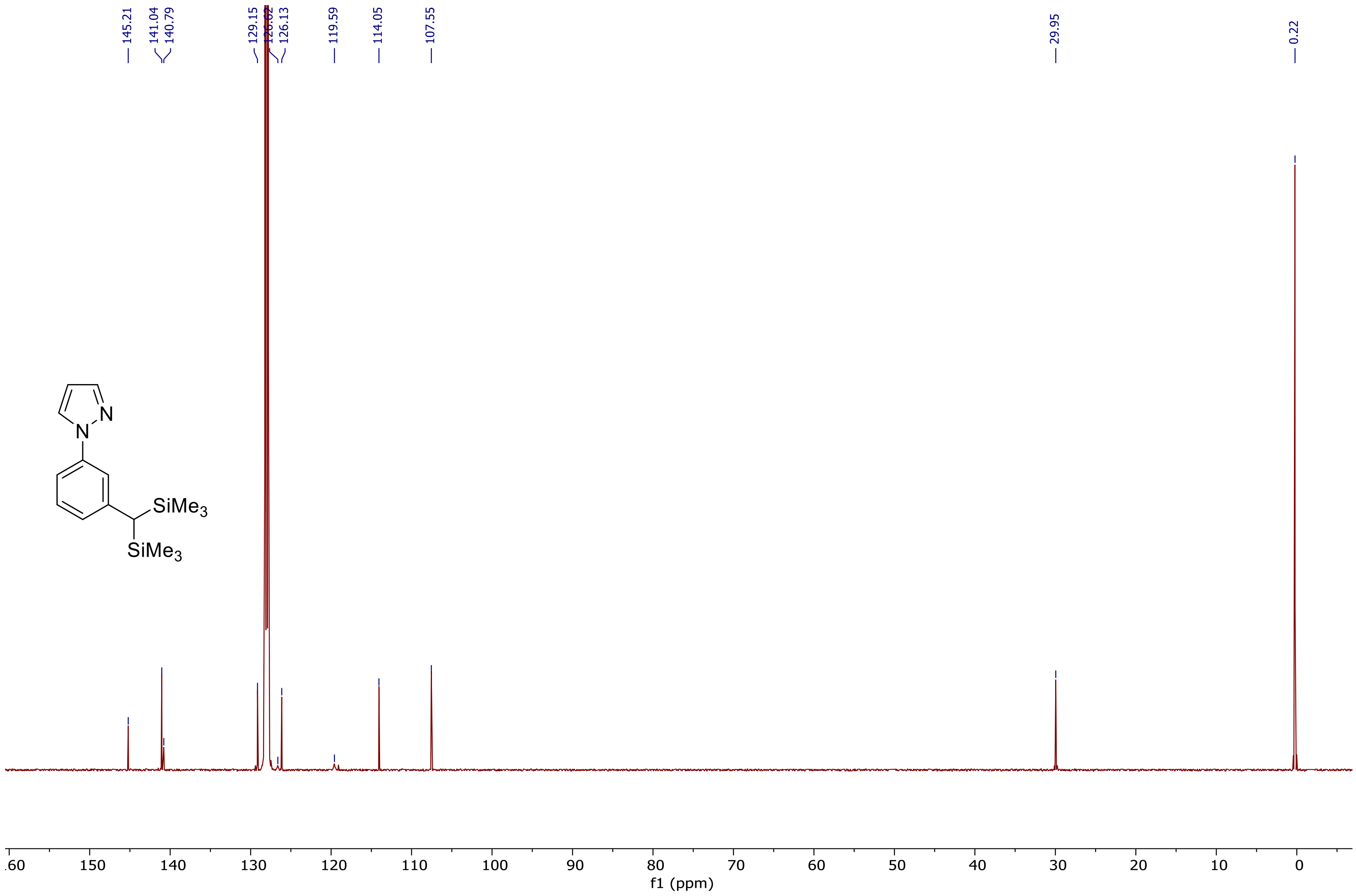

Supplementary Figure 223. ^{13}C NMR (126 MHz, C_6D_6) of 1-{3-[bis(trimethylsilyl)methyl]phenyl}-1H-pyrazole **6q**.

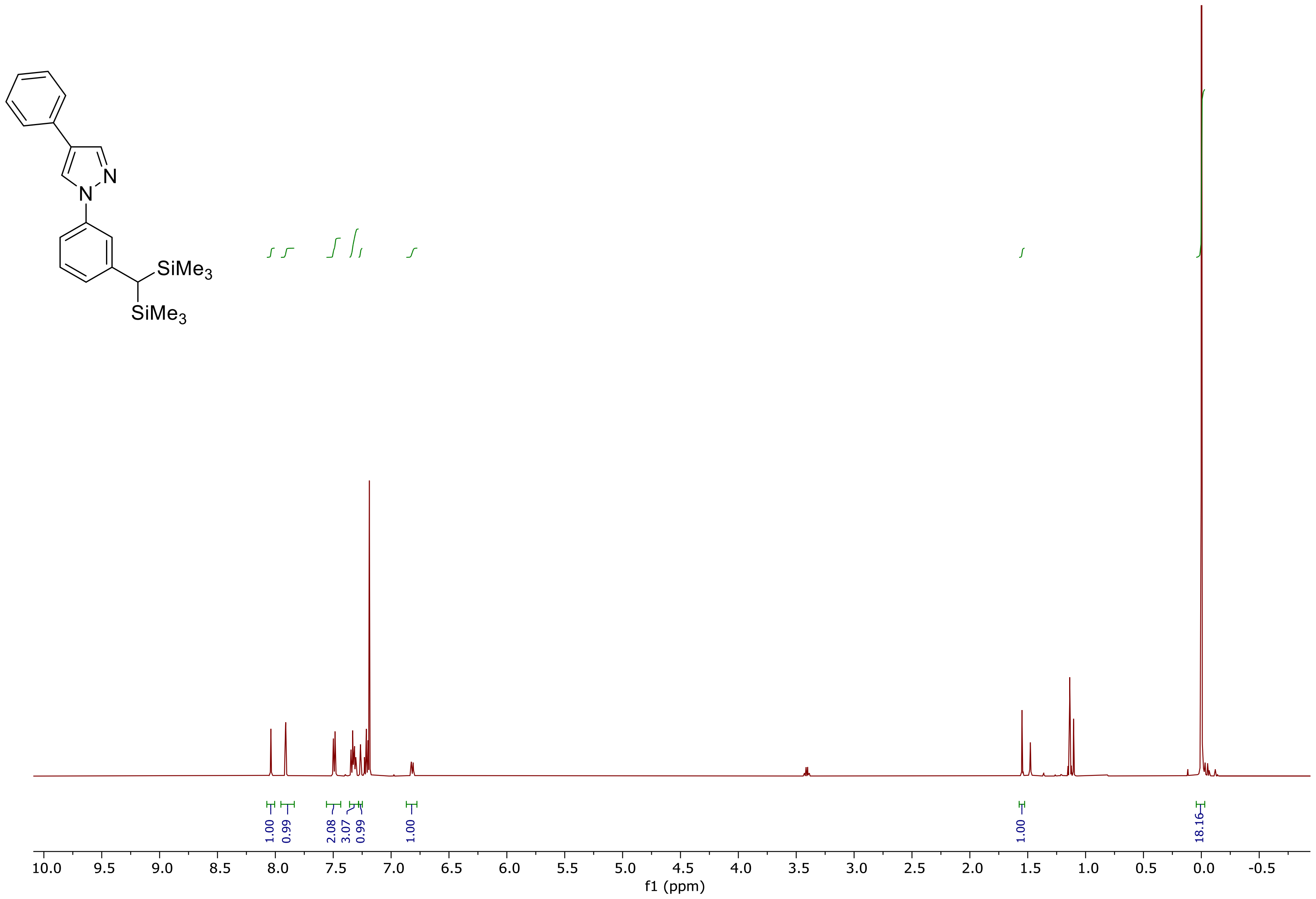

Supplementary Figure 224. ^{1}H NMR (500 MHz, $CDCl_3$) of 1-{3-[bis(trimethylsilyl)methyl]]phenyl}-4-phenyl-1H-pyrazole **6r**.

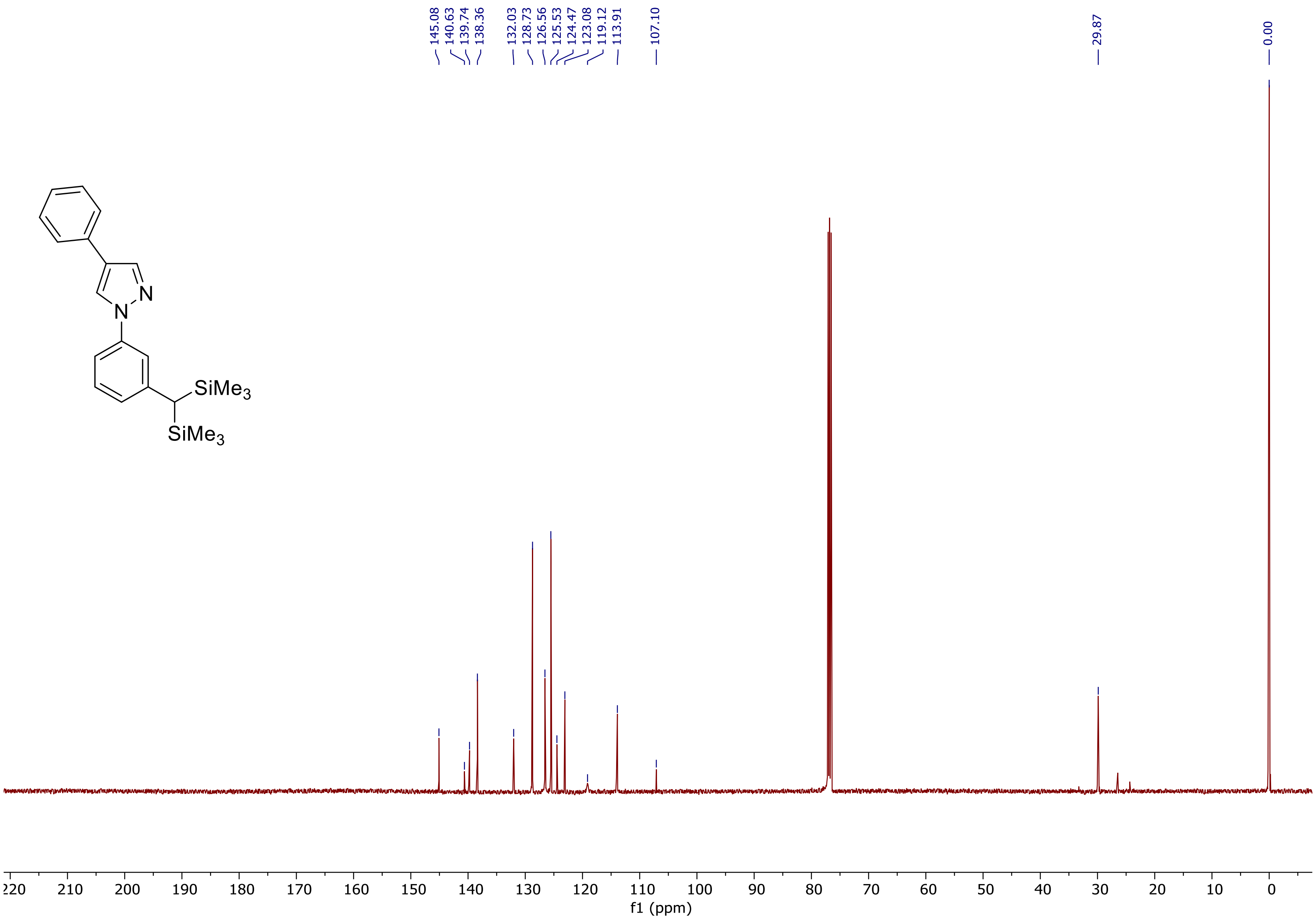

Supplementary Figure 225. ^{13}C NMR (126 MHz, $CDCl_3$) of 1-{3-[bis(trimethylsilyl)methyl]]phenyl}-4-phenyl-1H-pyrazole **6r**.

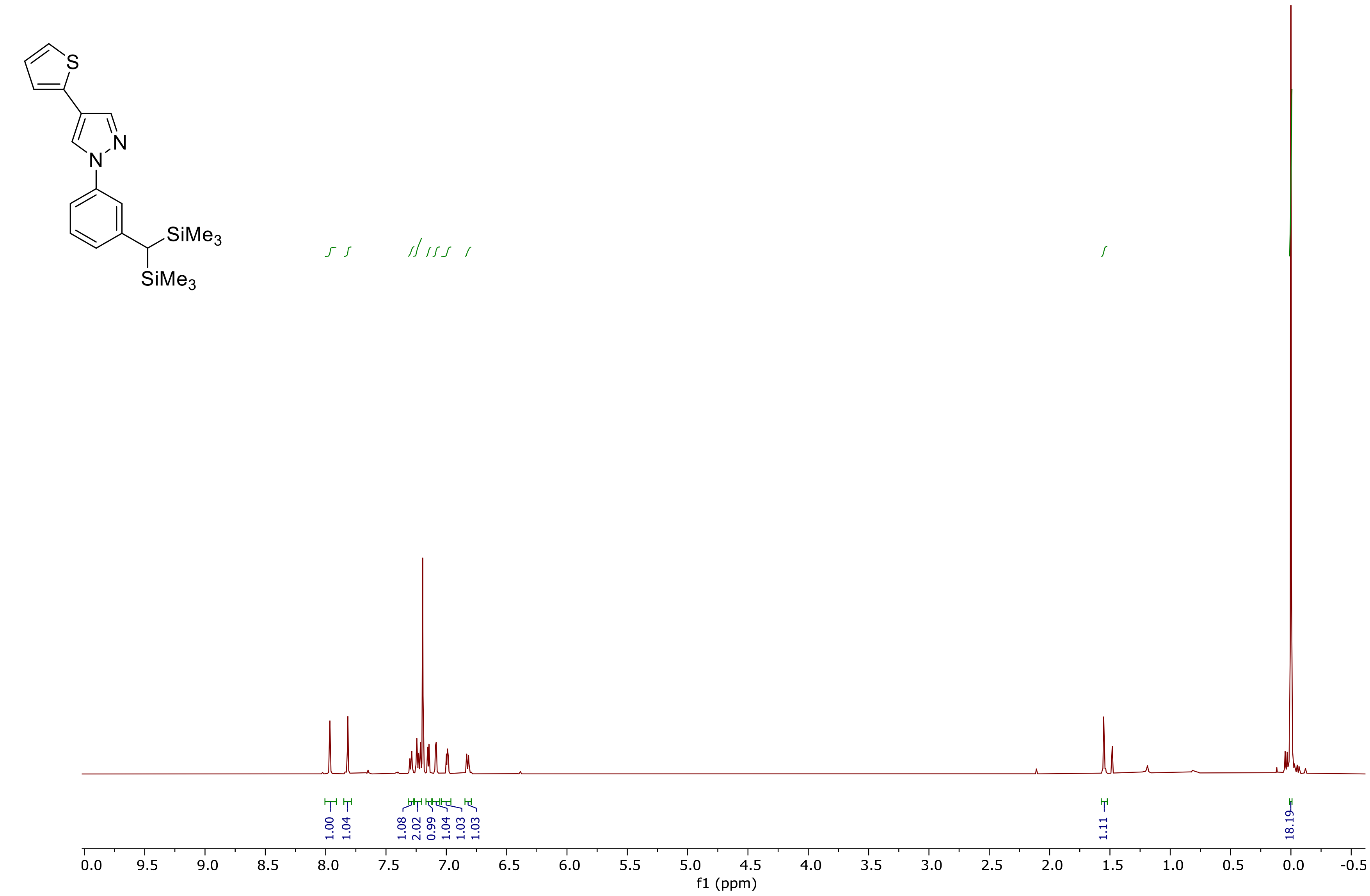

Supplementary Figure 226. ^1H NMR (500 MHz, $CDCl_3$) of 1-{3-[bis(trimethylsilyl)methyl]phenyl}-4-(thiophen-2-yl)-1H-pyrazole **6s**.

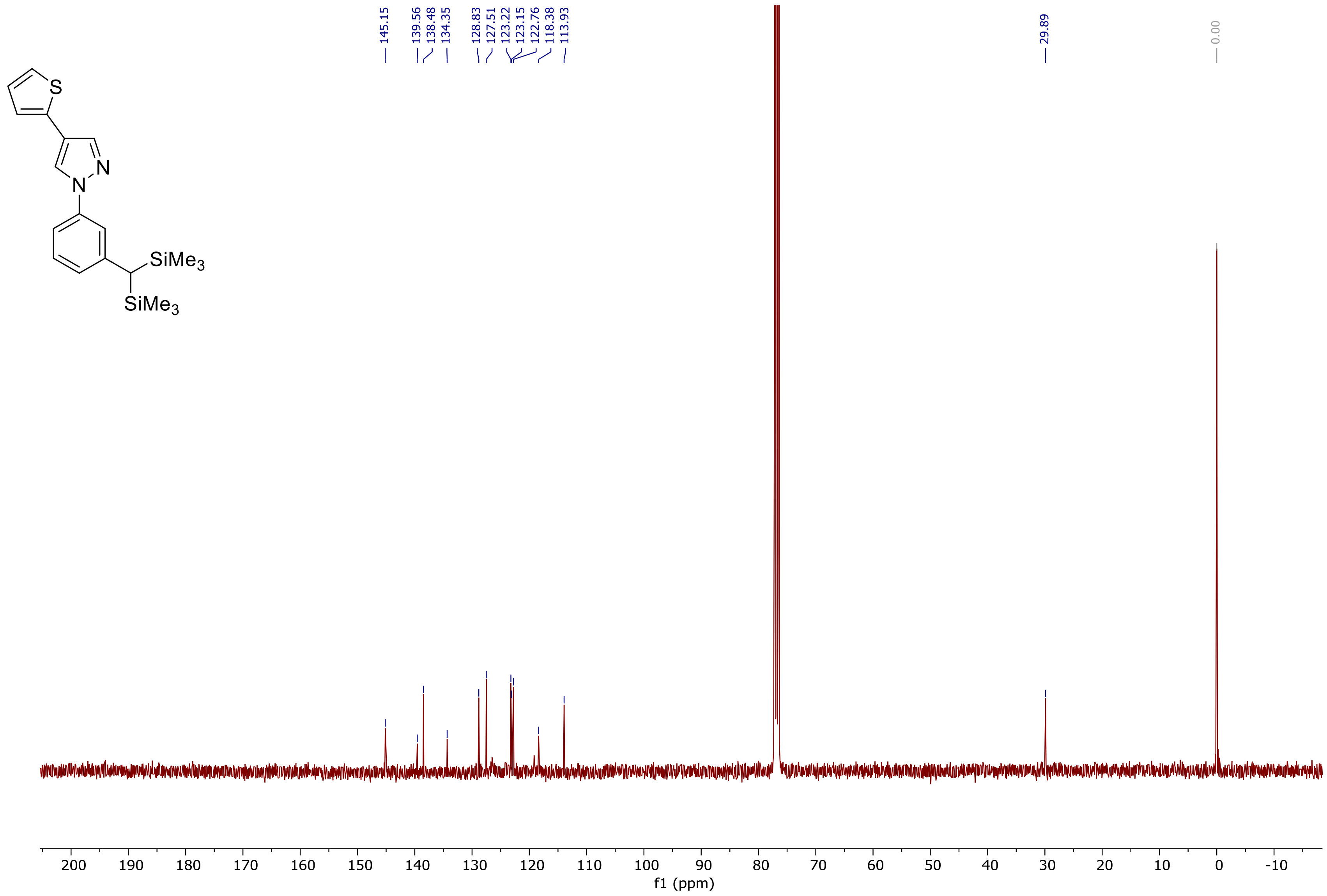

Supplementary Figure 227. ^{13}C NMR (101 MHz, $CDCl_3$) of 1-{3-[bis(trimethylsilyl)methyl]phenyl}-4-(thiophen-2-yl)-1H-pyrazole **6s**.

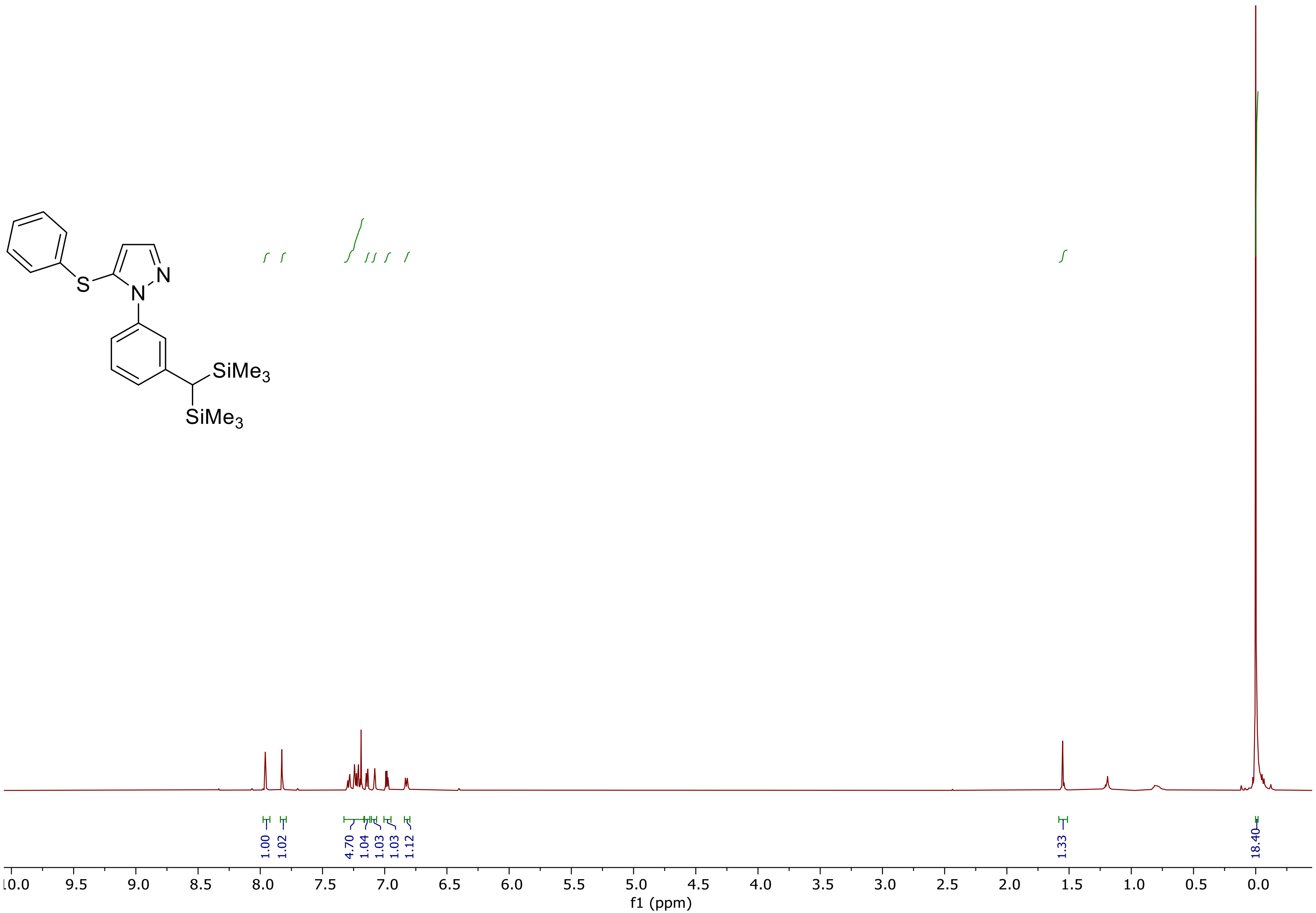

Supplementary Figure 228. ^{1}H NMR (500 MHz, $CDCl_3$) of 1-{3-[bis(trimethylsilyl)methyl]phenyl}-5-(phenylthio)-1H-pyrazole **6t**.

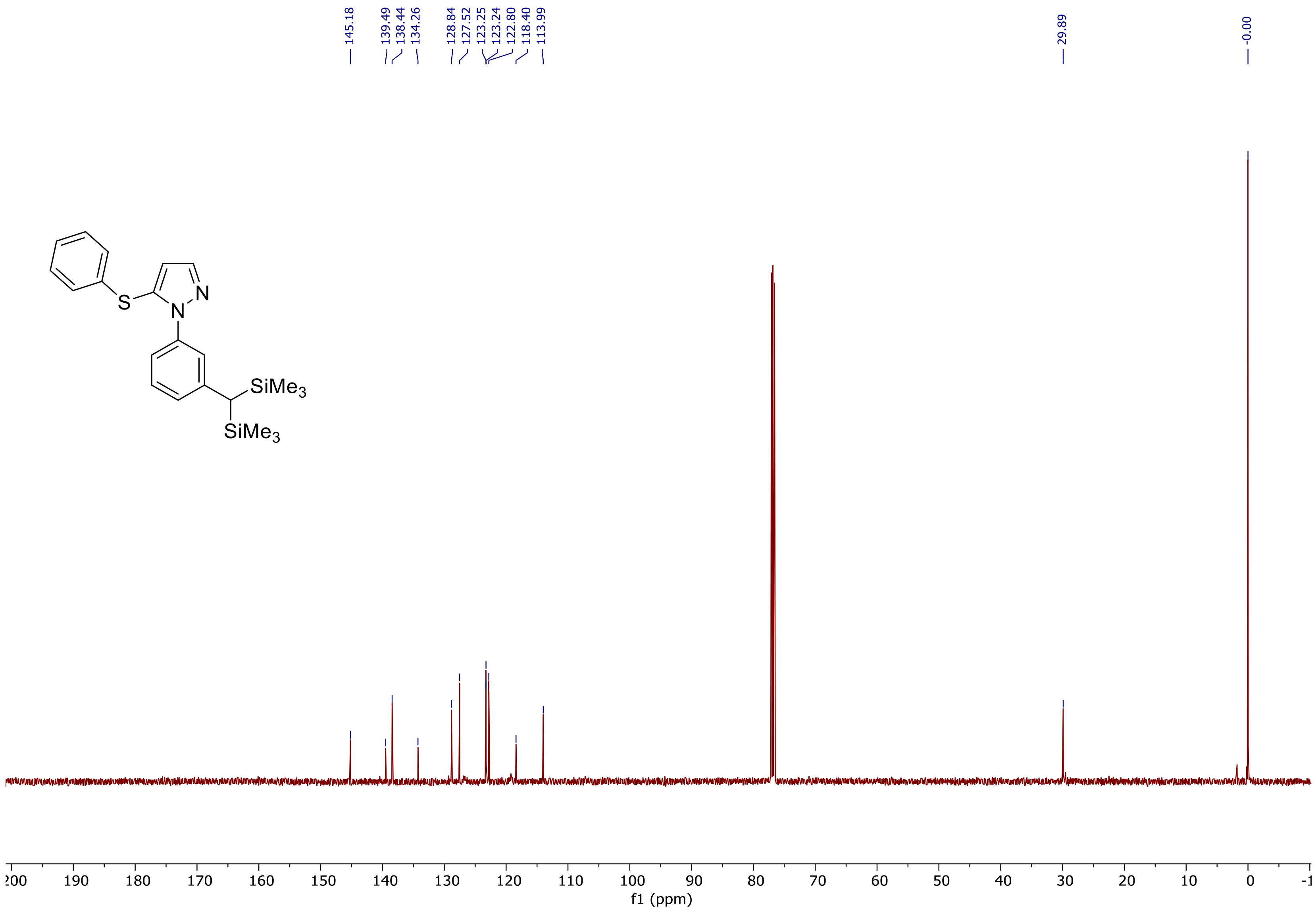

Supplementary Figure 229. ^{13}C NMR (126 MHz, $CDCl_3$) of 1-{3-[bis(trimethylsilyl)methyl]phenyl}-5-(phenylthio)-1H-pyrazole **6t**.

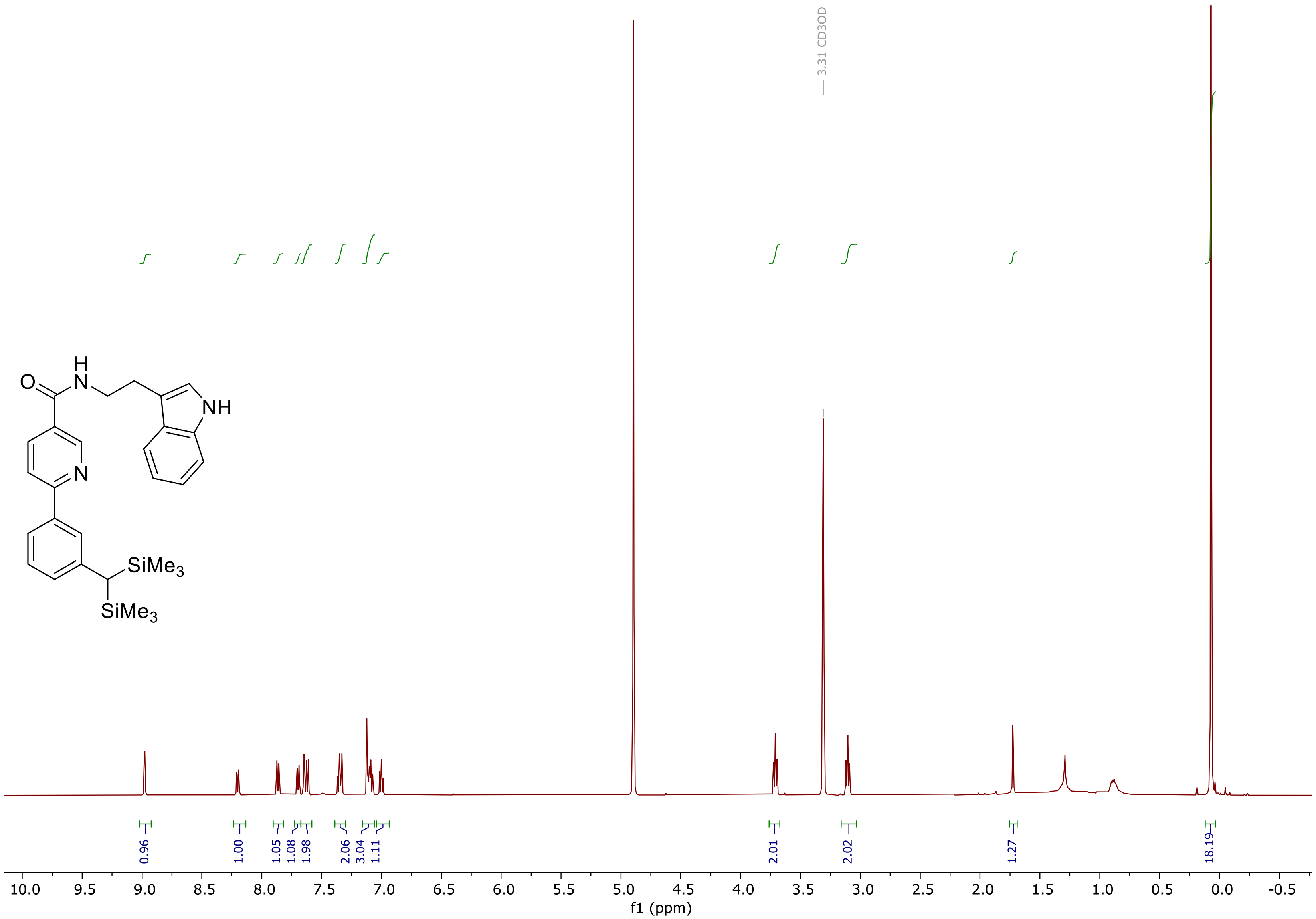

Supplementary Figure 230. ^{1}H NMR (500 MHz, $CDCl_3$) of *N*-[2-(*1H*-indol-3-yl)ethyl]-6{[3-(bis(trimethylsilyl)methyl]phenyl}nicotinamide **6u**.

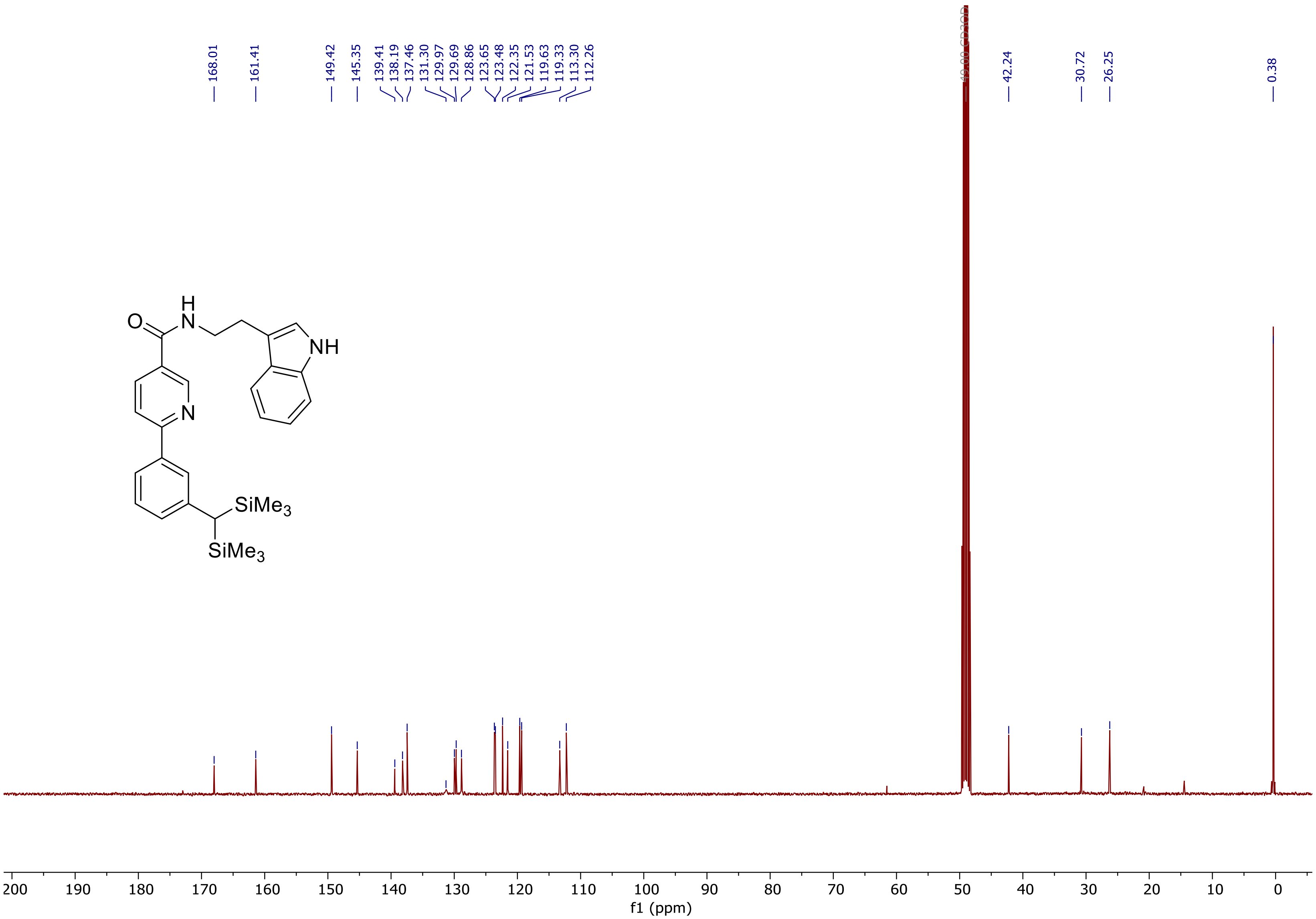

Supplementary Figure 231. ^{13}C NMR (101 MHz, $CDCl_3$) of *N*-[2-(*1H*-indol-3-yl)ethyl]-6{[3-(bis(trimethylsilyl)methyl]phenyl}nicotinamide **6u**.

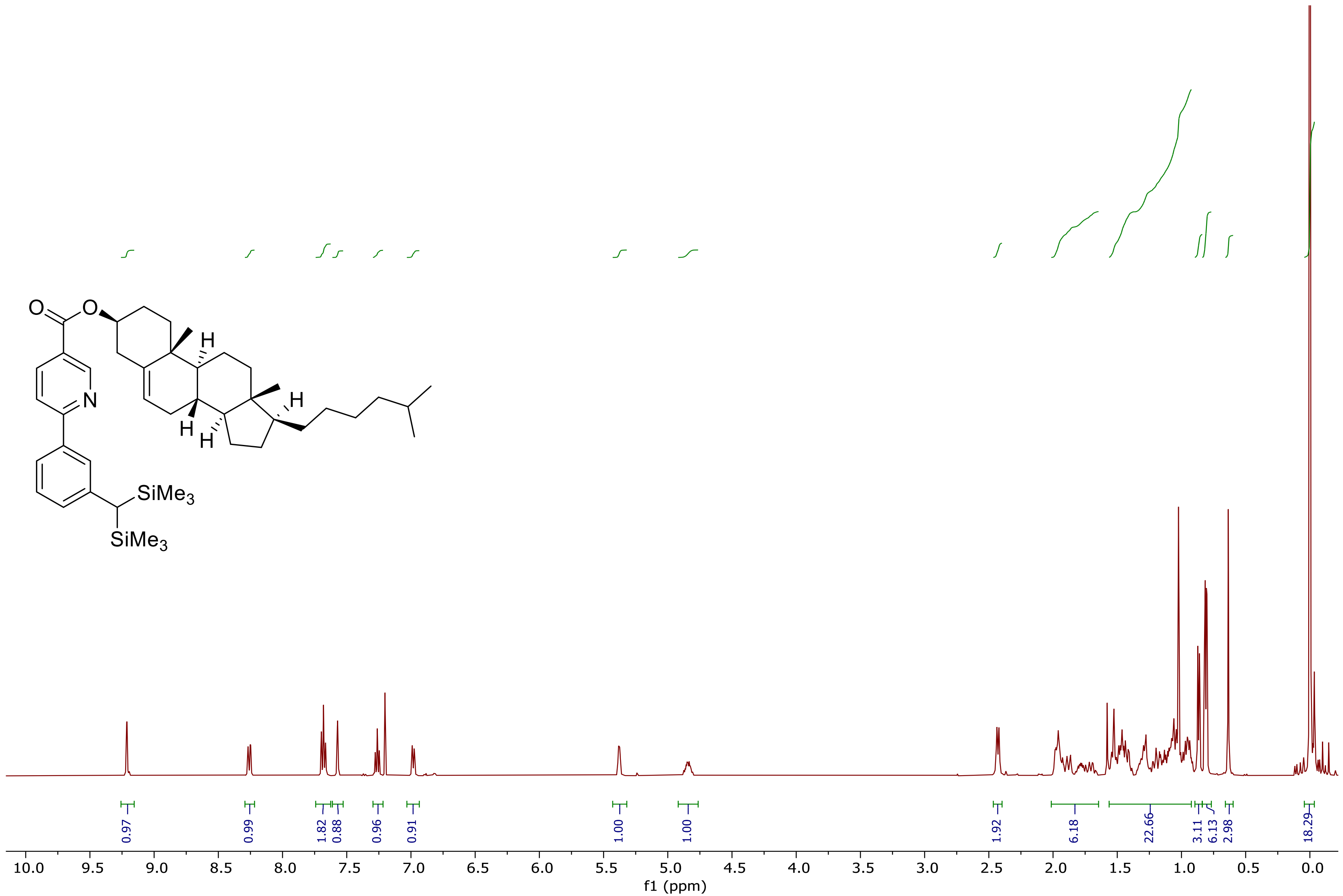

Supplementary Figure 232. ^{1}H NMR (500 MHz, $CDCl_3$) of (3S,8S,9S,10R,13R,14S,17S)-10,13-dimethyl-17-(5-methylhexyl)-2,3,4,7,8,9,10,11,12,13,14,15,16,17-tetradecahydro-1H-cyclopenta[a]phenanthren-3-yl 6-(3-(bis(trimethylsilyl)methyl)phenyl)nicotinate **6v**.

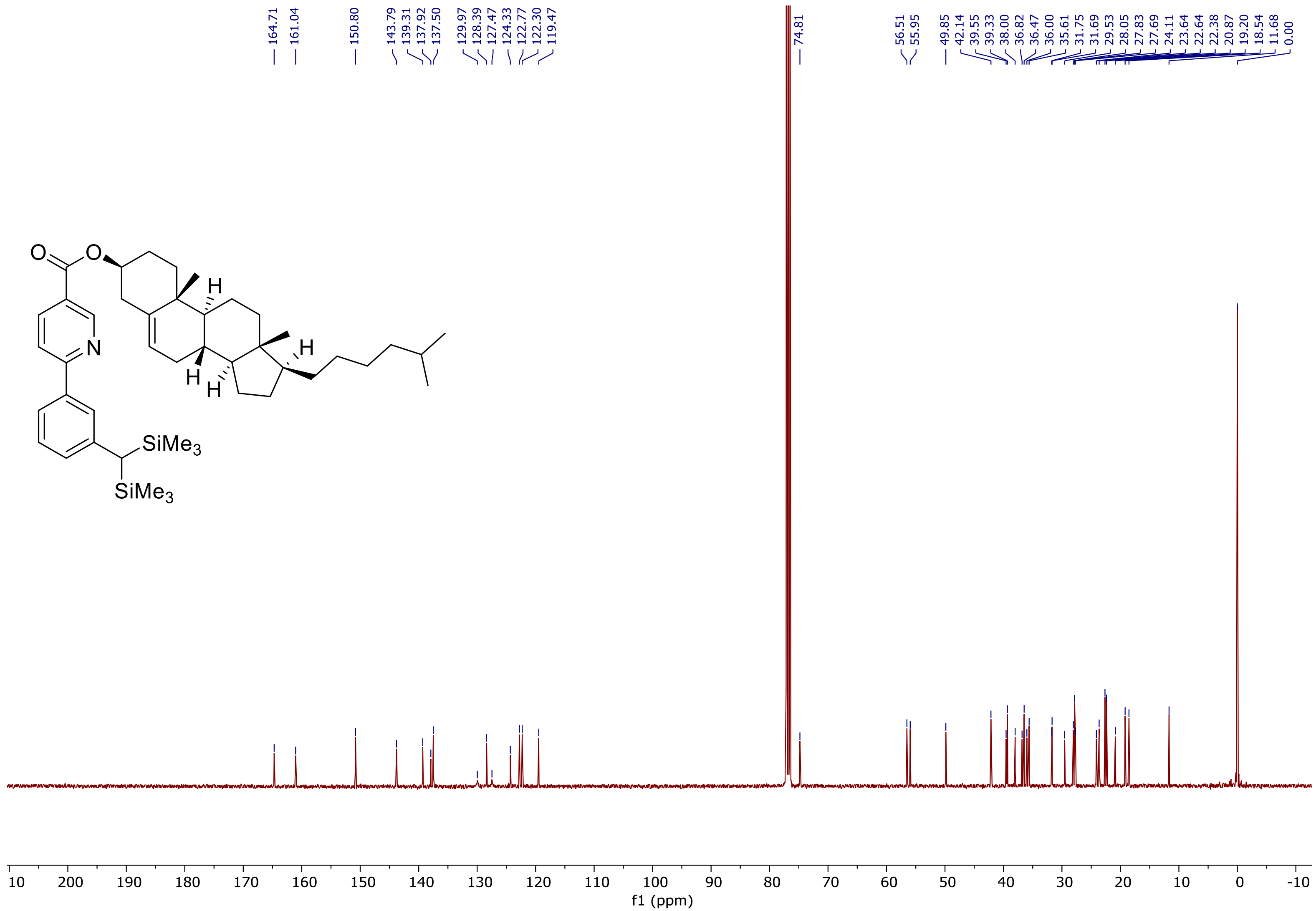

Supplementary Figure 233. ^{13}C NMR (101 MHz, $CDCl_3$) of (3S,8S,9S,10R,13R,14S,17S)-10,13-dimethyl-17-(5-methylhexyl)-2,3,4,7,8,9,10,11,12,13,14,15,16,17-tetradecahydro-1H-cyclopenta[a]phenanthren-3-yl 6-(3-(bis(trimethylsilyl)methyl)phenyl)nicotinate **6v**.

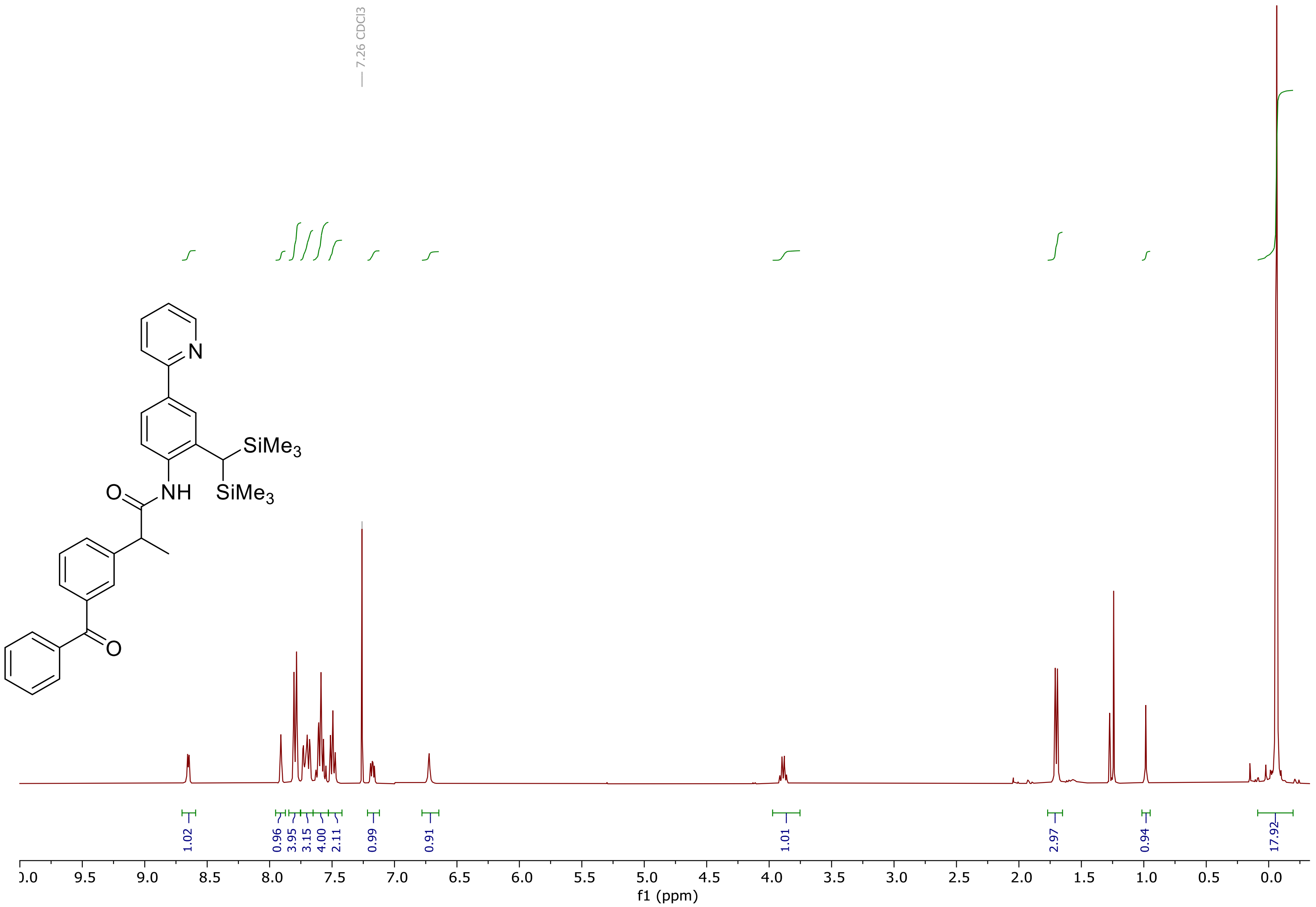

Supplementary Figure 234. ^{1}H NMR (400 MHz, $CDCl_3$) of 2-(3-benzoylphenyl)-*N*-{2-[bis(trimethylsilyl)methyl]-4-[pyridin-2-yl]phenyl}propenamide **6w**.

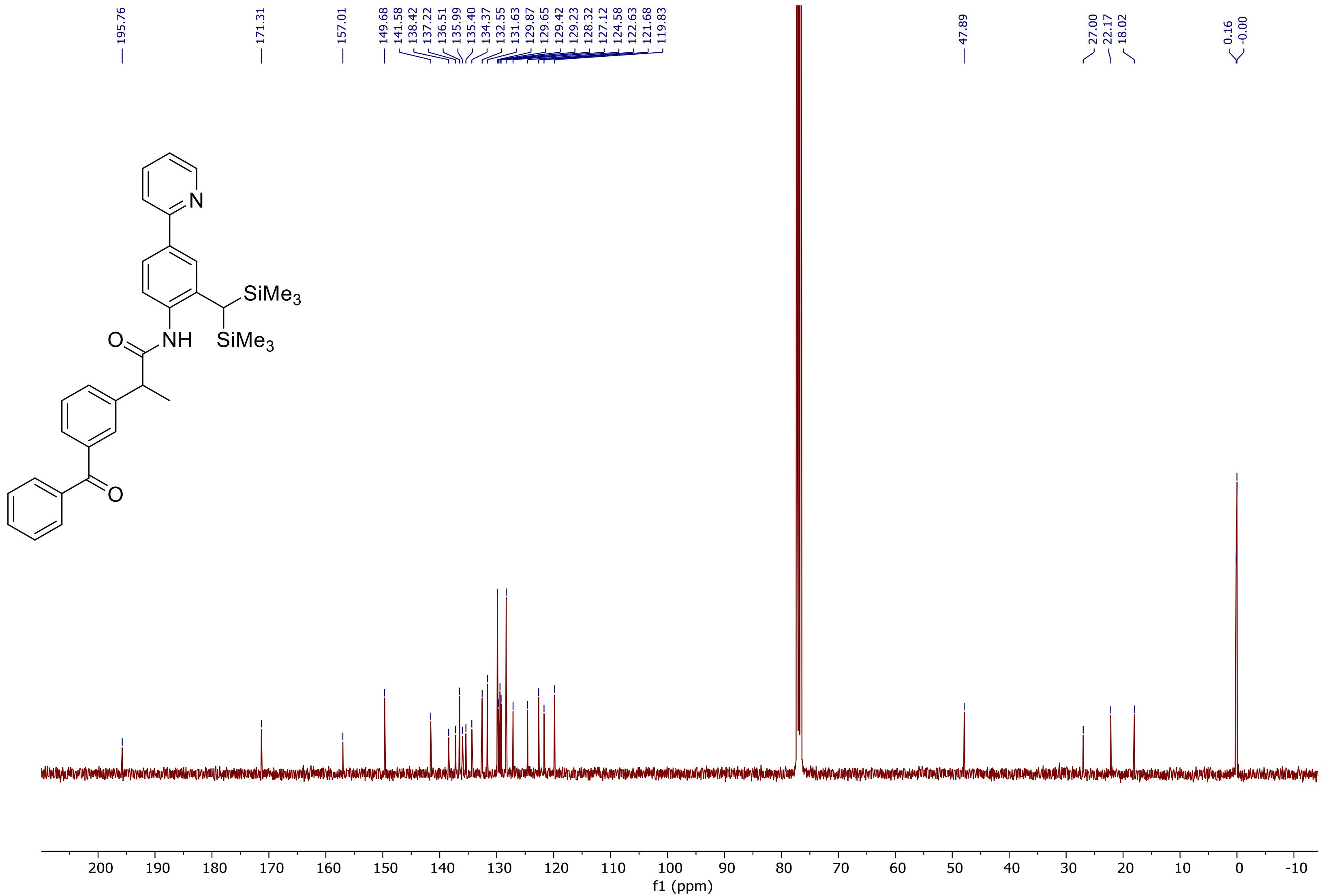

Supplementary Figure 235. ^{13}C NMR (101 MHz, $CDCl_3$) of 2-(3-benzoylphenyl)-*N*-{2-[bis(trimethylsilyl)methyl]-4-[pyridin-2-yl]phenyl}propenamide **6w**.

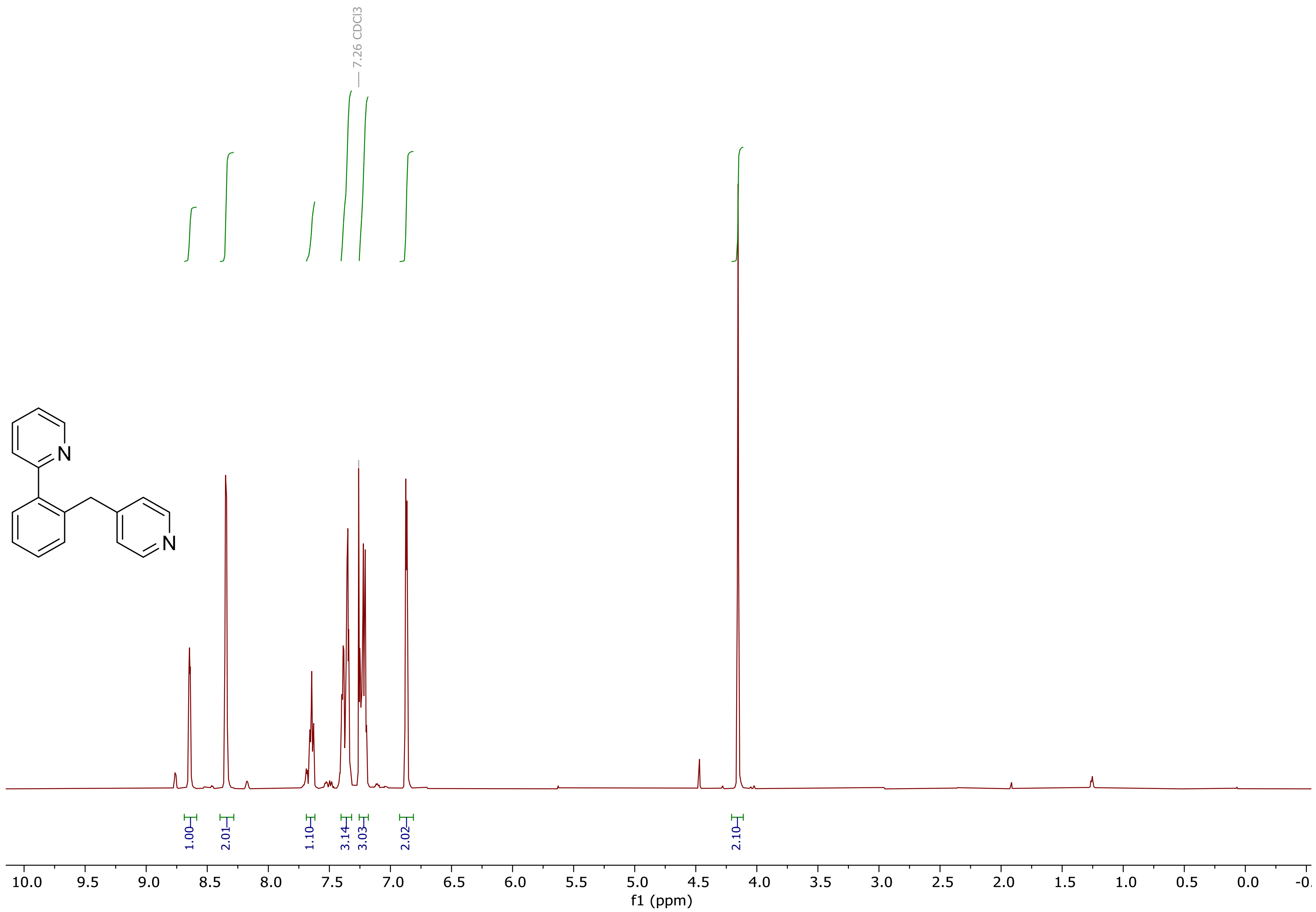

Supplementary Figure 236. ^{1}H NMR (500 MHz, $CDCl_3$) of 2-[2-(pyridin-4-ylmethyl)phenyl]pyridine **10**.

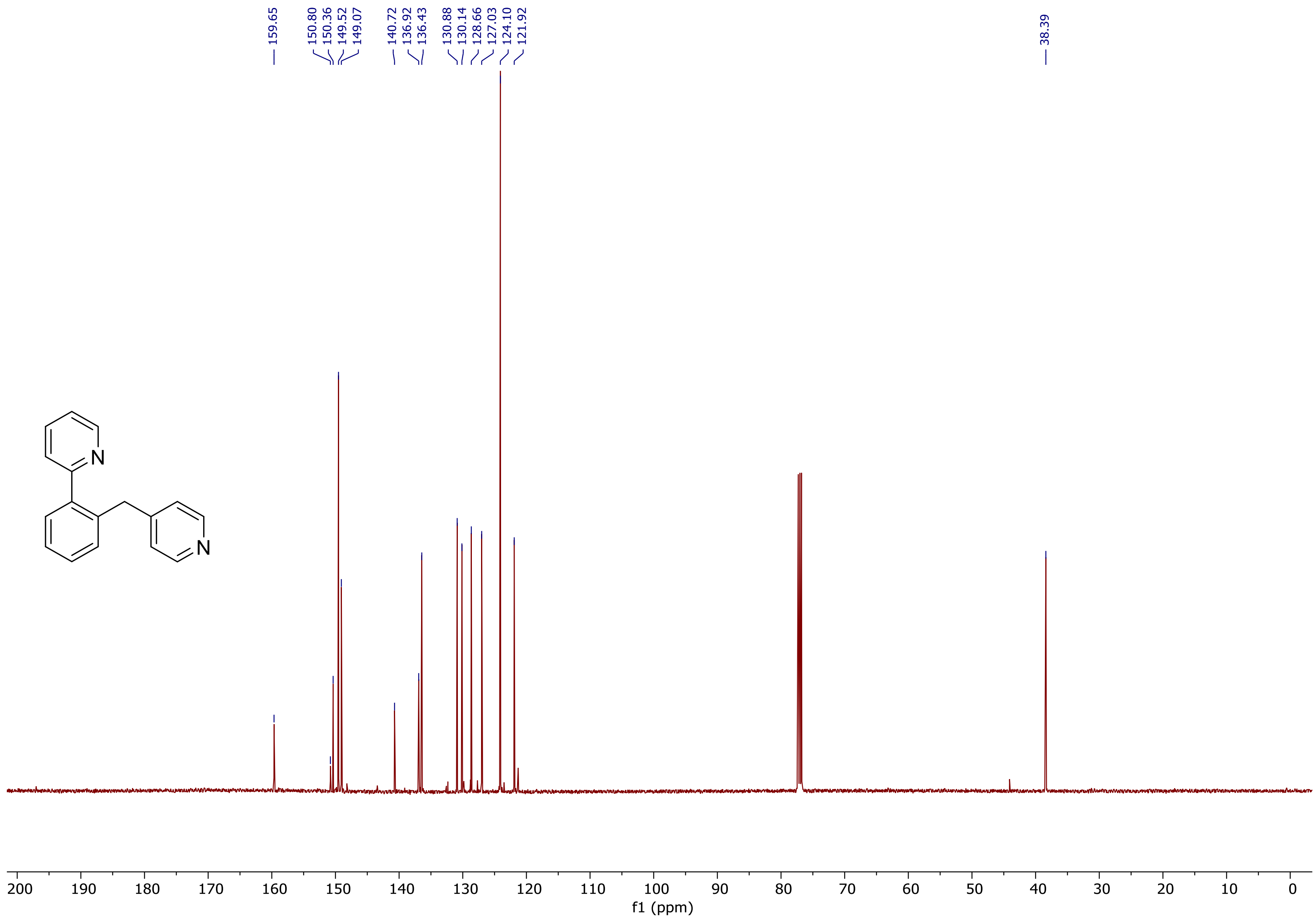

Supplementary Figure 237. ^{13}C NMR (126 MHz, $CDCl_3$) of 2-[2-(pyridin-4-ylmethyl)phenyl]pyridine **10**.

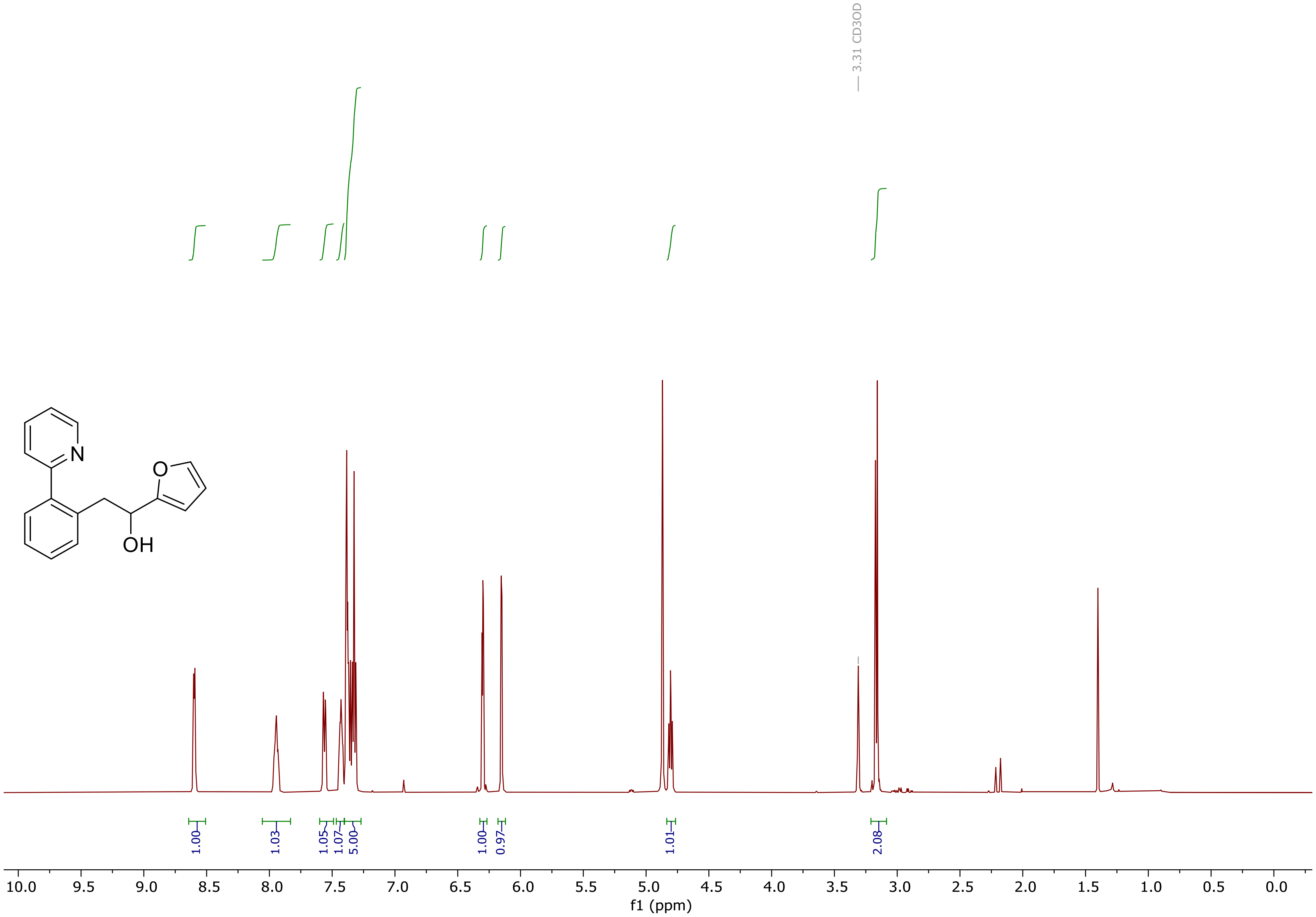

Supplementary Figure 238. ^{1}H NMR (500 MHz, d_4-CD_3OD) of 1-(furan-2-yl)-2-[2-(pyridin-2-yl)phenyl]ethan-1-ol **12**.

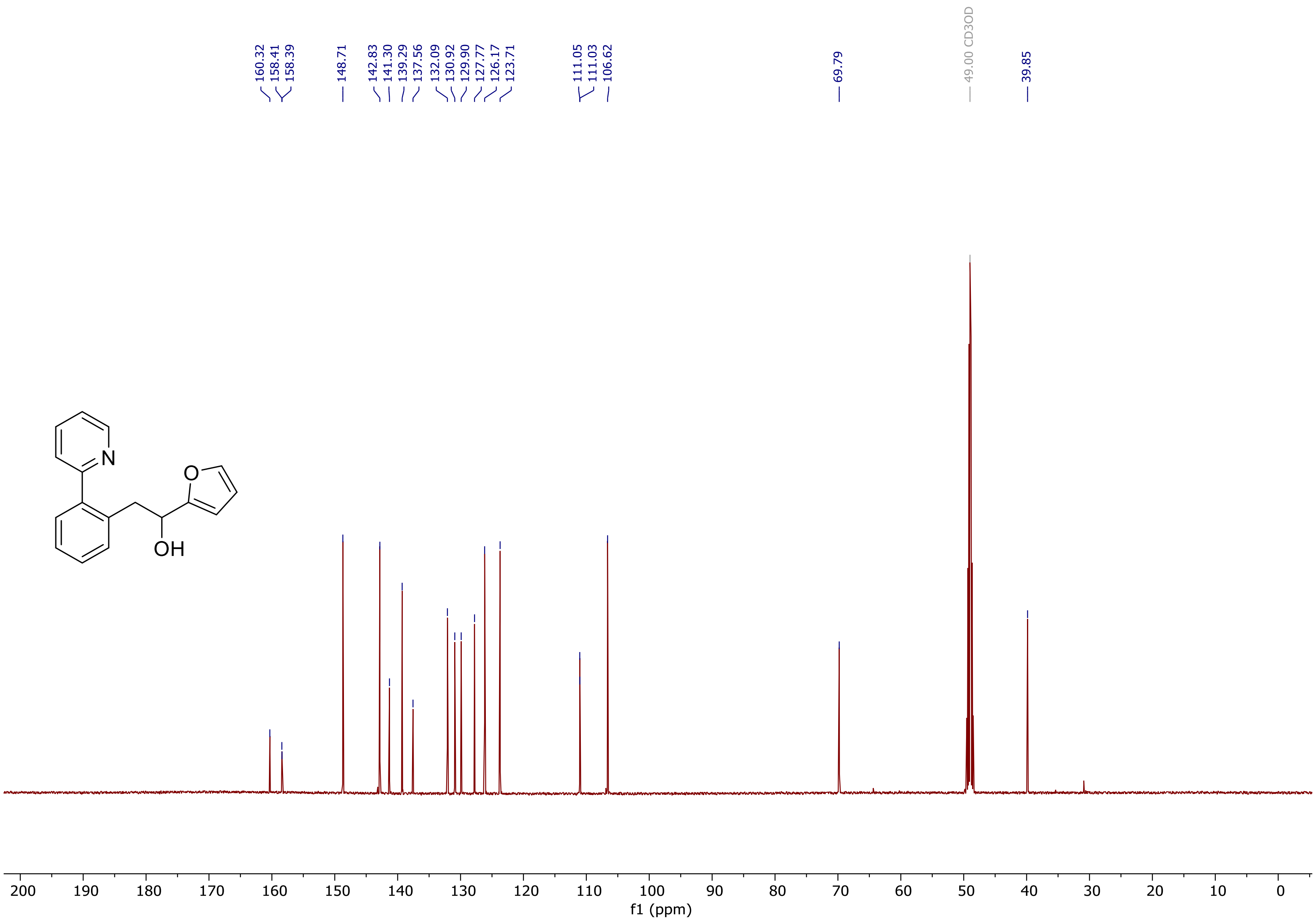

Supplementary Figure 239. ^{13}C NMR (126 MHz, d_4-CD_3OD) of 1-(furan-2-yl)-2-[2-(pyridin-2-yl)phenyl]ethan-1-ol **12**.

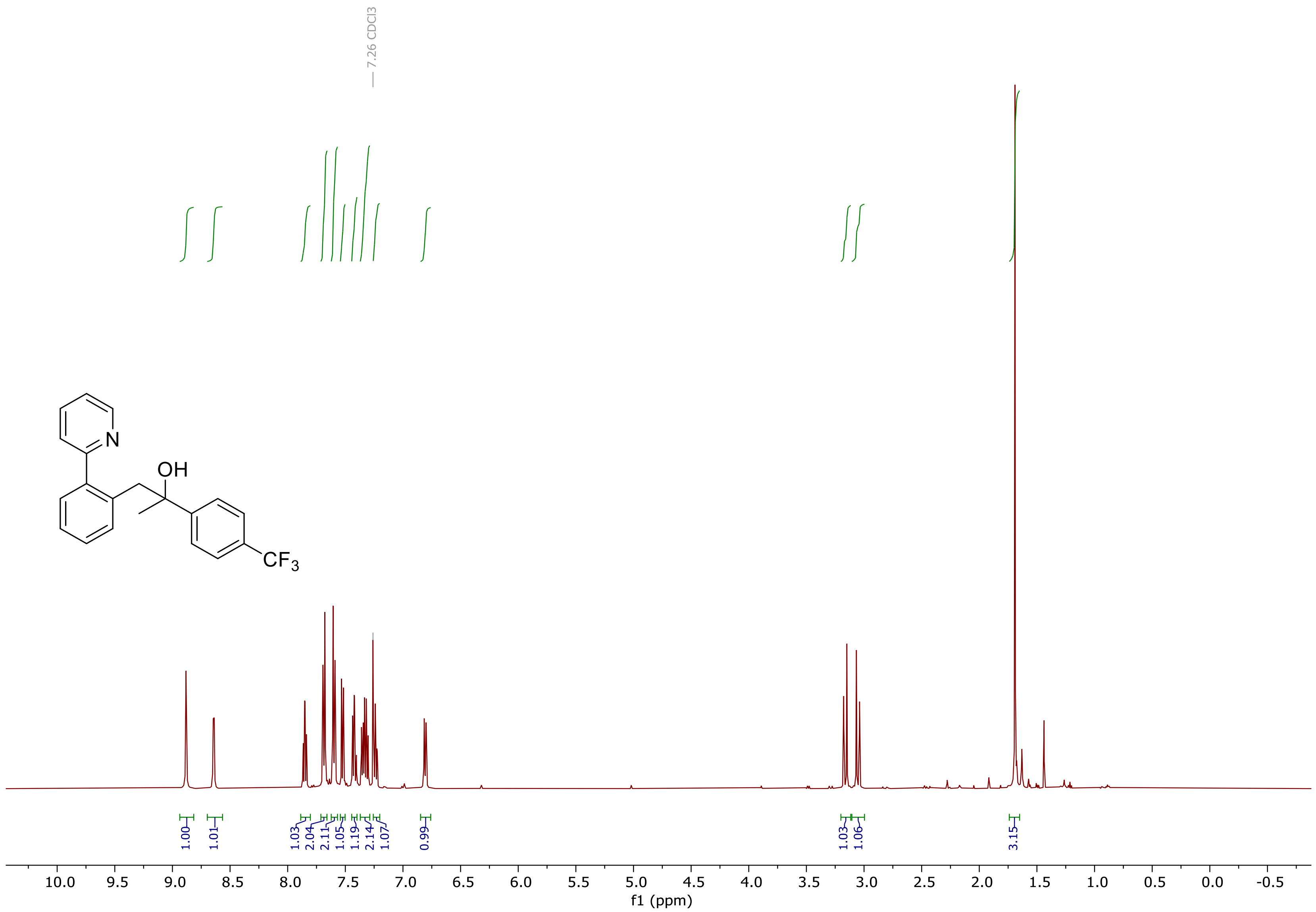

Supplementary Figure 240. ^{1}H NMR (500 MHz, $CDCl_3$) of 1-[2-(pyridin-2-yl)phenyl]-2-[4-(trifluoromethyl)phenyl]propan-2-ol **14**.

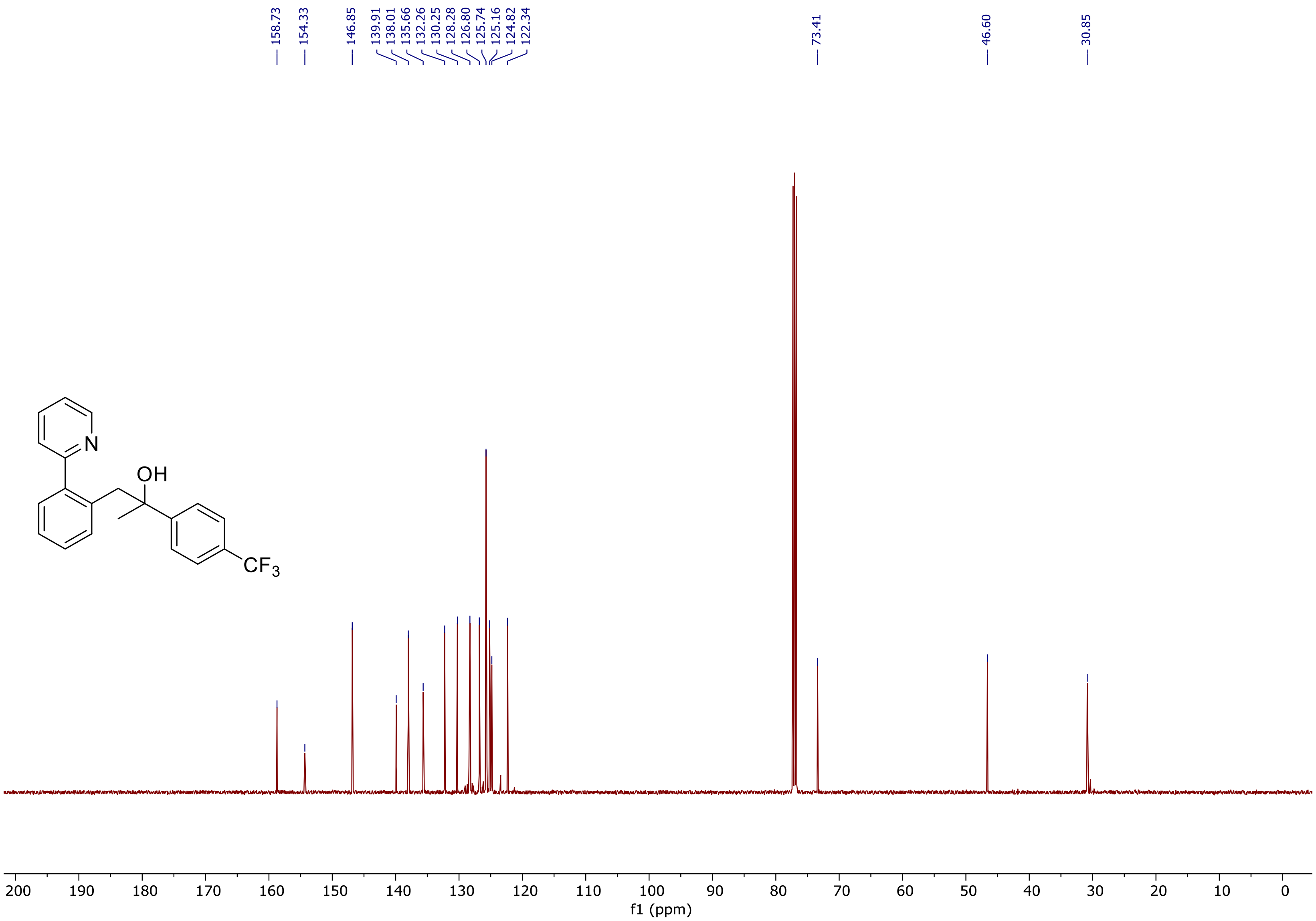

Supplementary Figure 241. ^{13}C NMR (126 MHz, $CDCl_3$) of 1-[2-(pyridin-2-yl)phenyl]-2-[4-(trifluoromethyl)phenyl]propan-2-ol **14**.

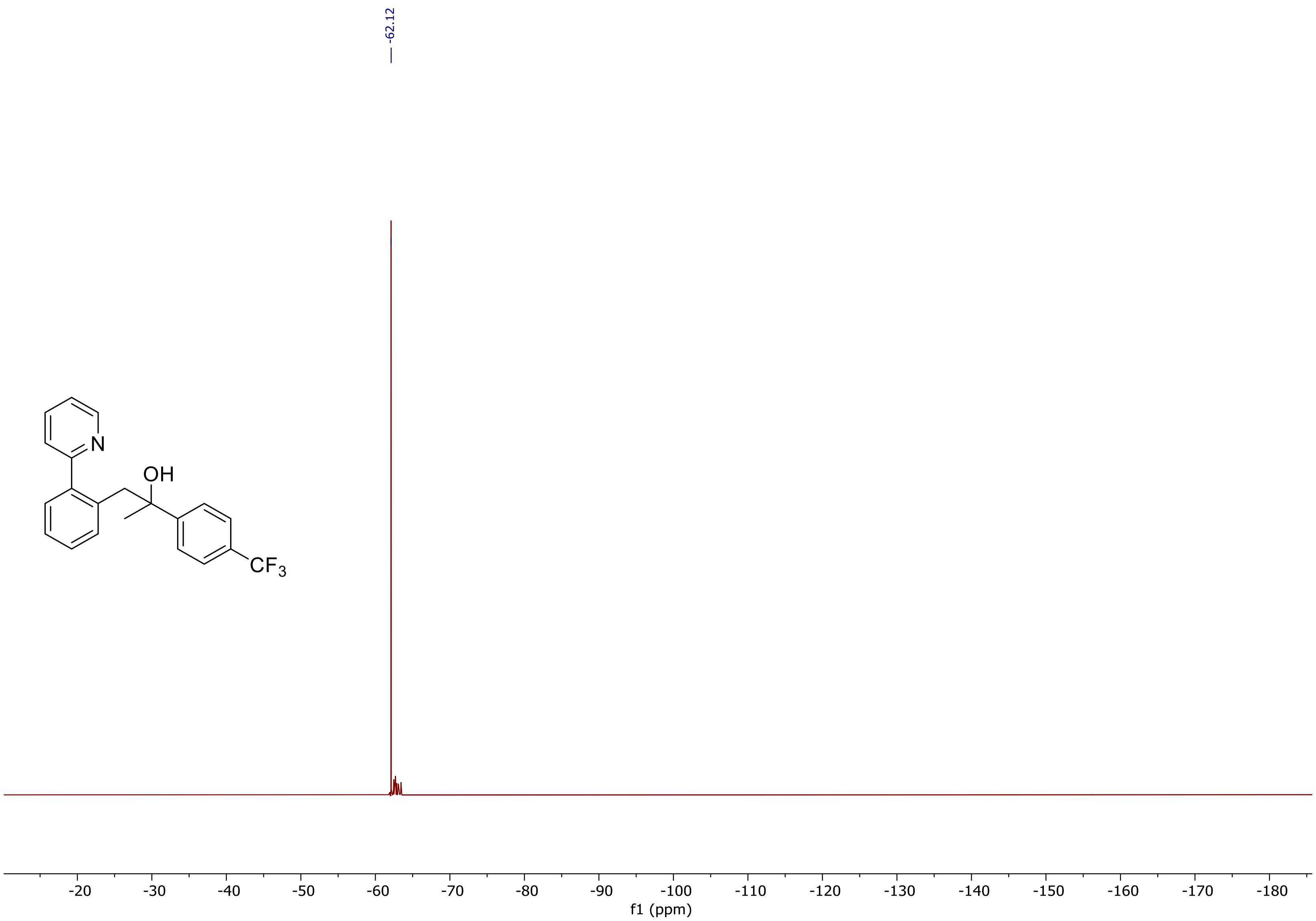

Supplementary Figure 242. ^{19}F NMR (471 MHz, $CDCl_3$) of 1-[2-(pyridin-2-yl)phenyl]-2-[4-(trifluoromethyl)phenyl]propan-2-ol **14**.

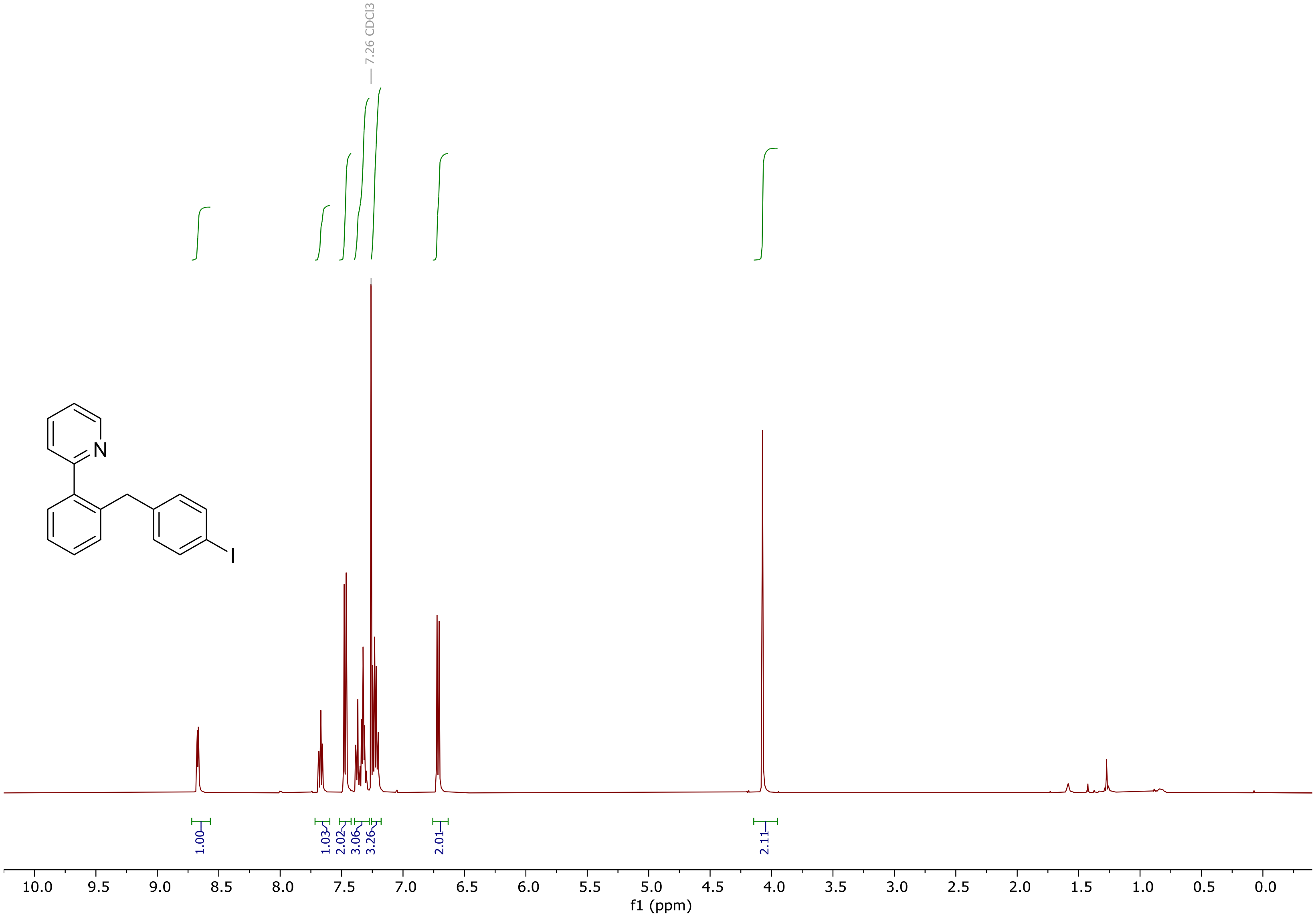

Supplementary Figure 243. ^{1}H NMR (500 MHz, $CDCl_3$) of 2-[2-(4-iodobenzyl)phenyl]pyridine **16**.

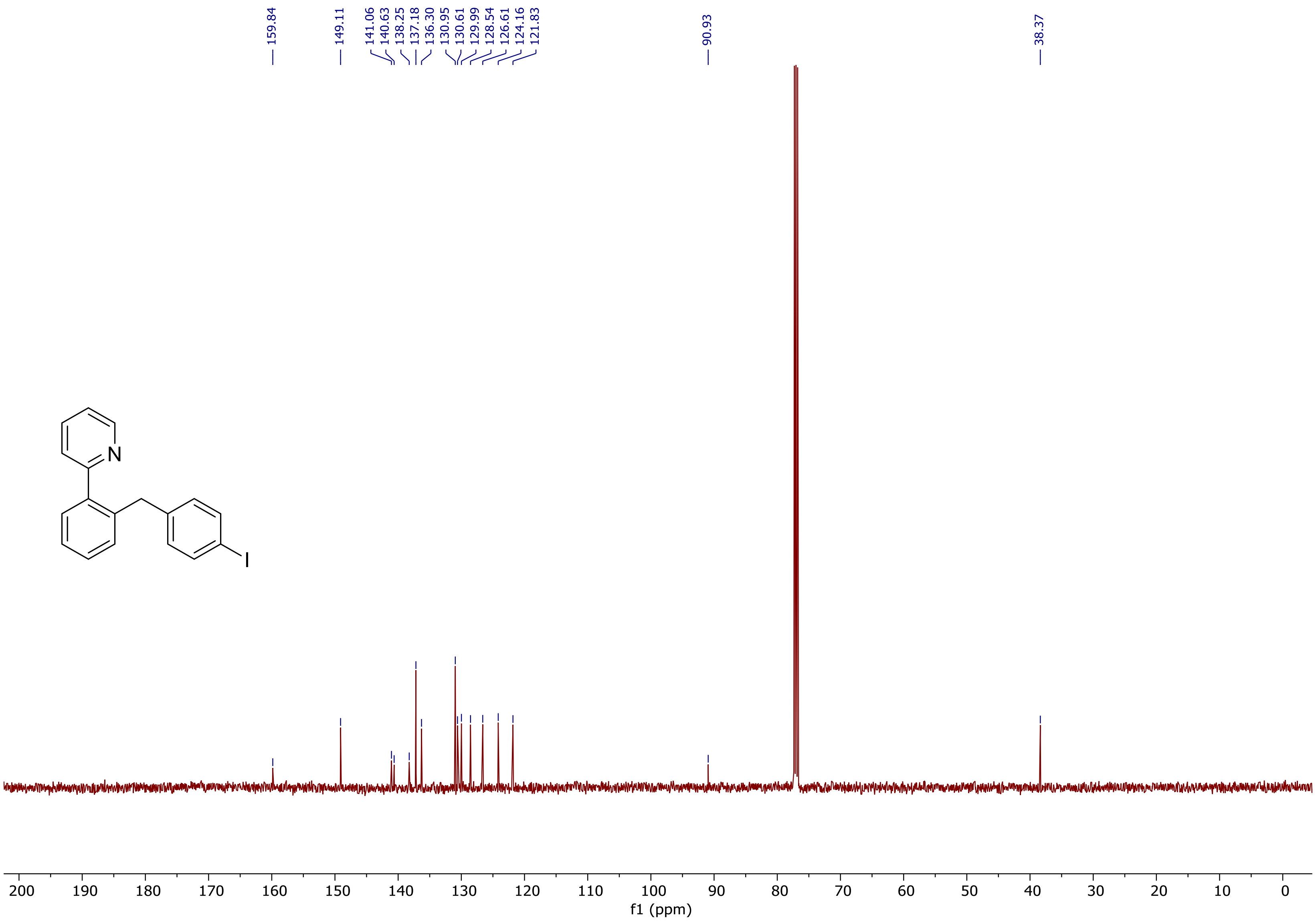

Supplementary Figure 244. ^{13}C NMR (101 MHz, $CDCl_3$) of 2-[2-(4-iodobenzyl)phenyl]pyridine **16**.

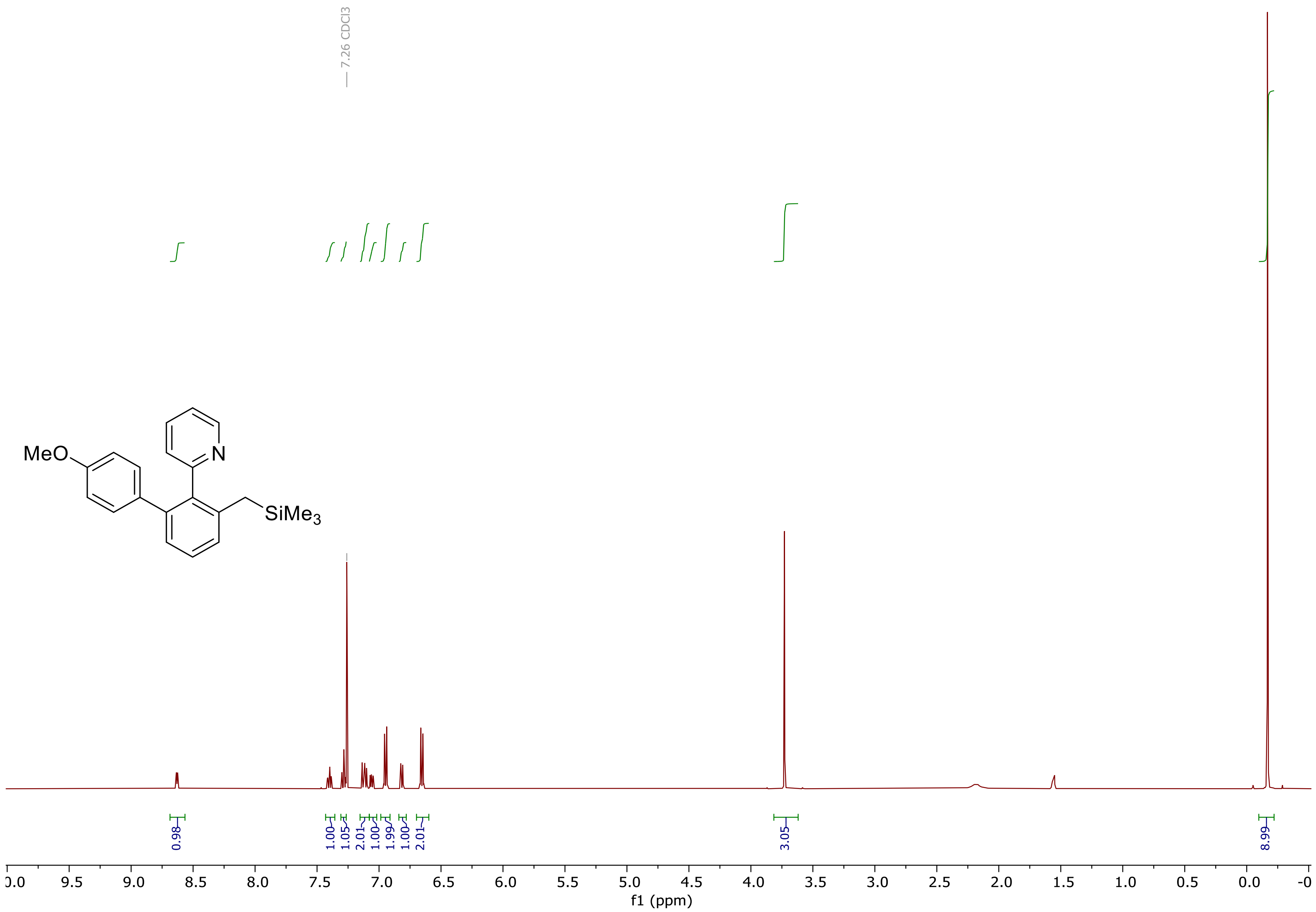

Supplementary Figure 245. ^{1}H NMR (500 MHz, $CDCl_3$) of 2-{4'-methoxy-3-[(trimethylsilyl)methyl]-(1,1'-biphenyl)-2-yl}pyridine **18**.

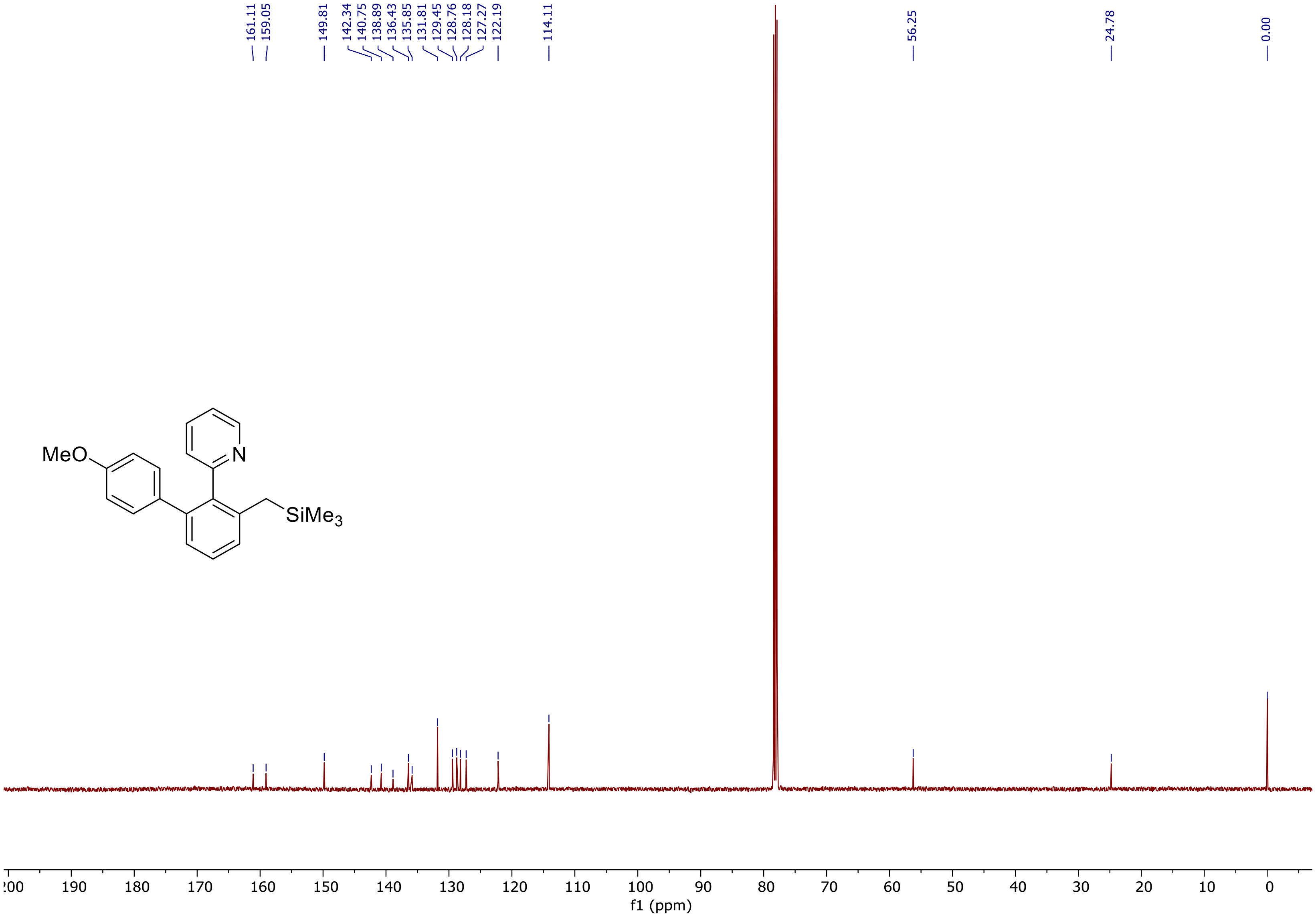

Supplementary Figure 246. ^{13}C NMR (126 MHz, $CDCl_3$) of 2-{4'-methoxy-3-[(trimethylsilyl)methyl]-(1,1'-biphenyl)-2-yl}pyridine **18**.

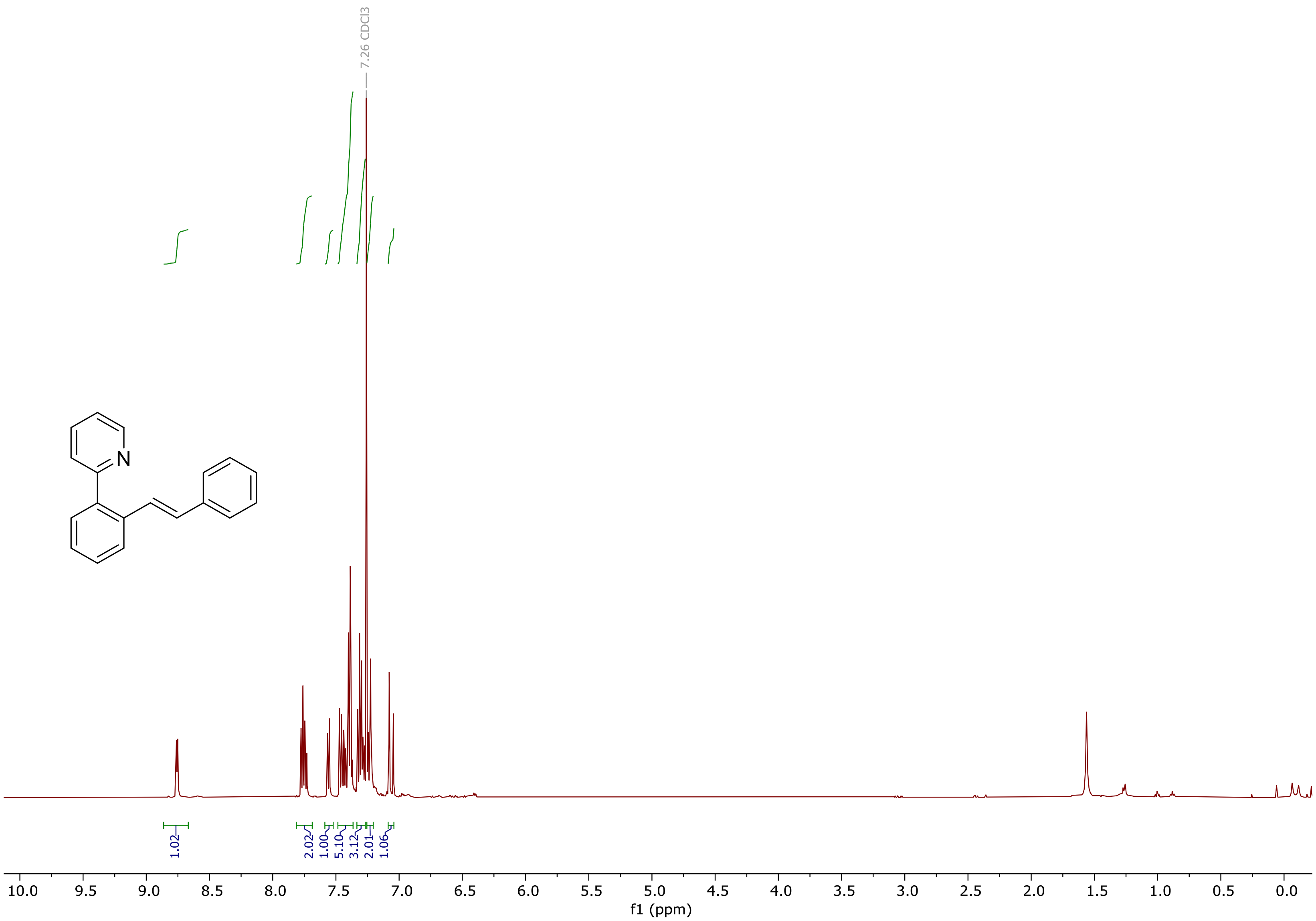

Supplementary Figure 247. ^{1}H NMR (500 MHz, $CDCl_3$) of (*E*)-2-(2-styrylphenyl)pyridine **20**.

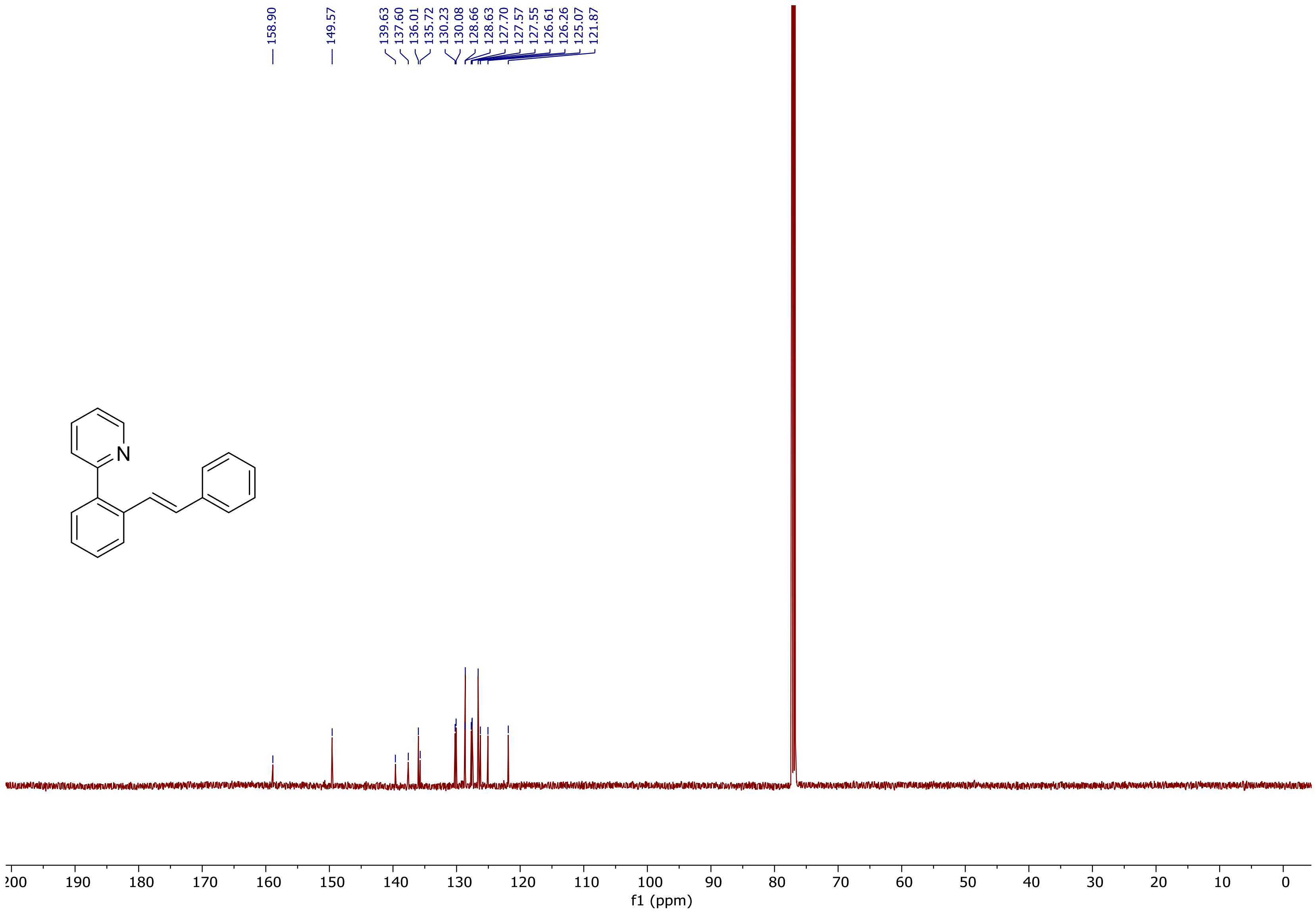

Supplementary Figure 248. ^{13}C NMR (126 MHz, $CDCl_3$) of (*E*)-2-(2-styrylphenyl)pyridine **20**.

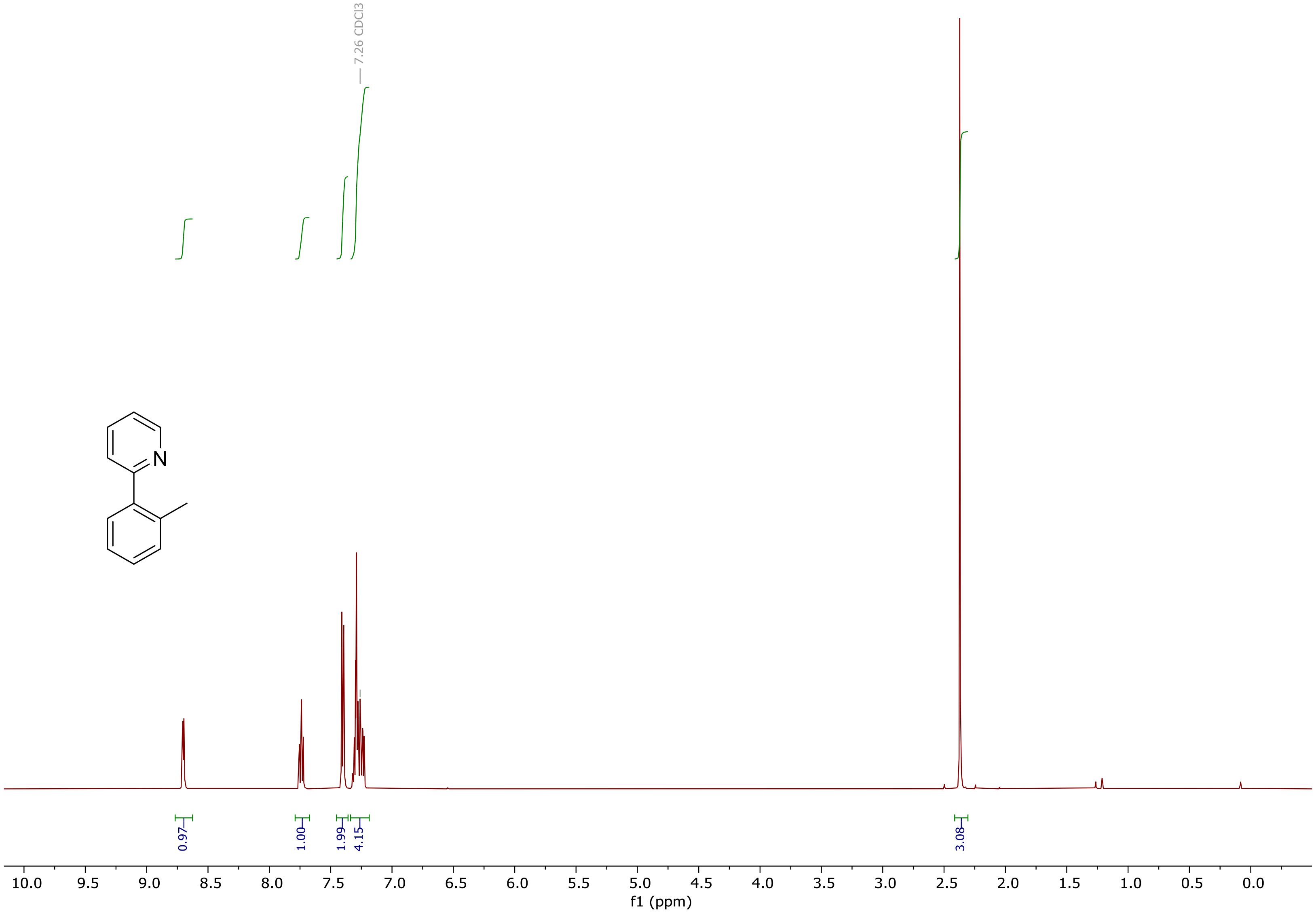

Supplementary Figure 249. ^{1}H NMR (500 MHz, $CDCl_3$) of 2-(*o*-tolyl)pyridine **21**.

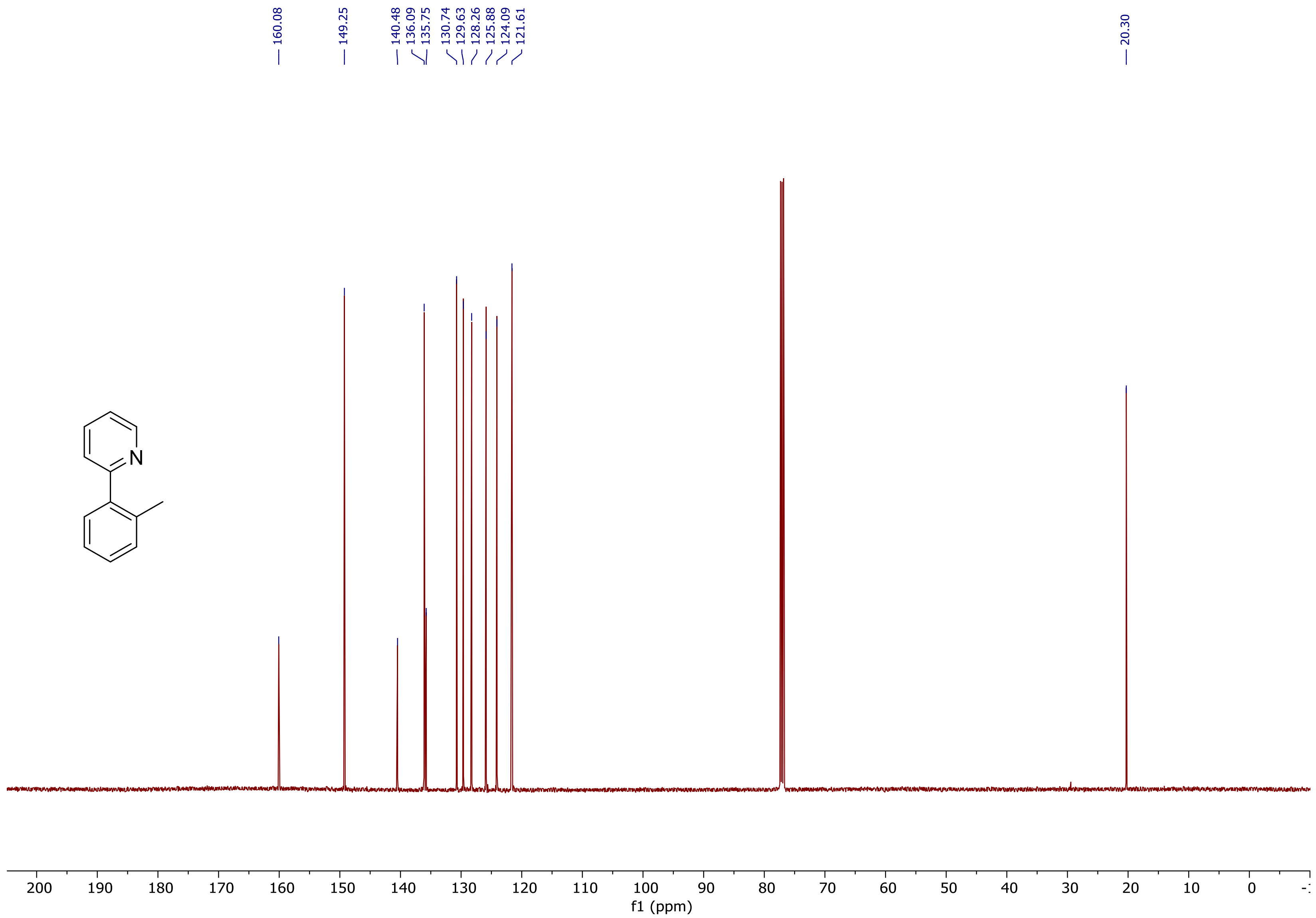

Supplementary Figure 250. ^{13}C NMR (126 MHz, $CDCl_3$) of 2-(*o*-tolyl)pyridine **21**.

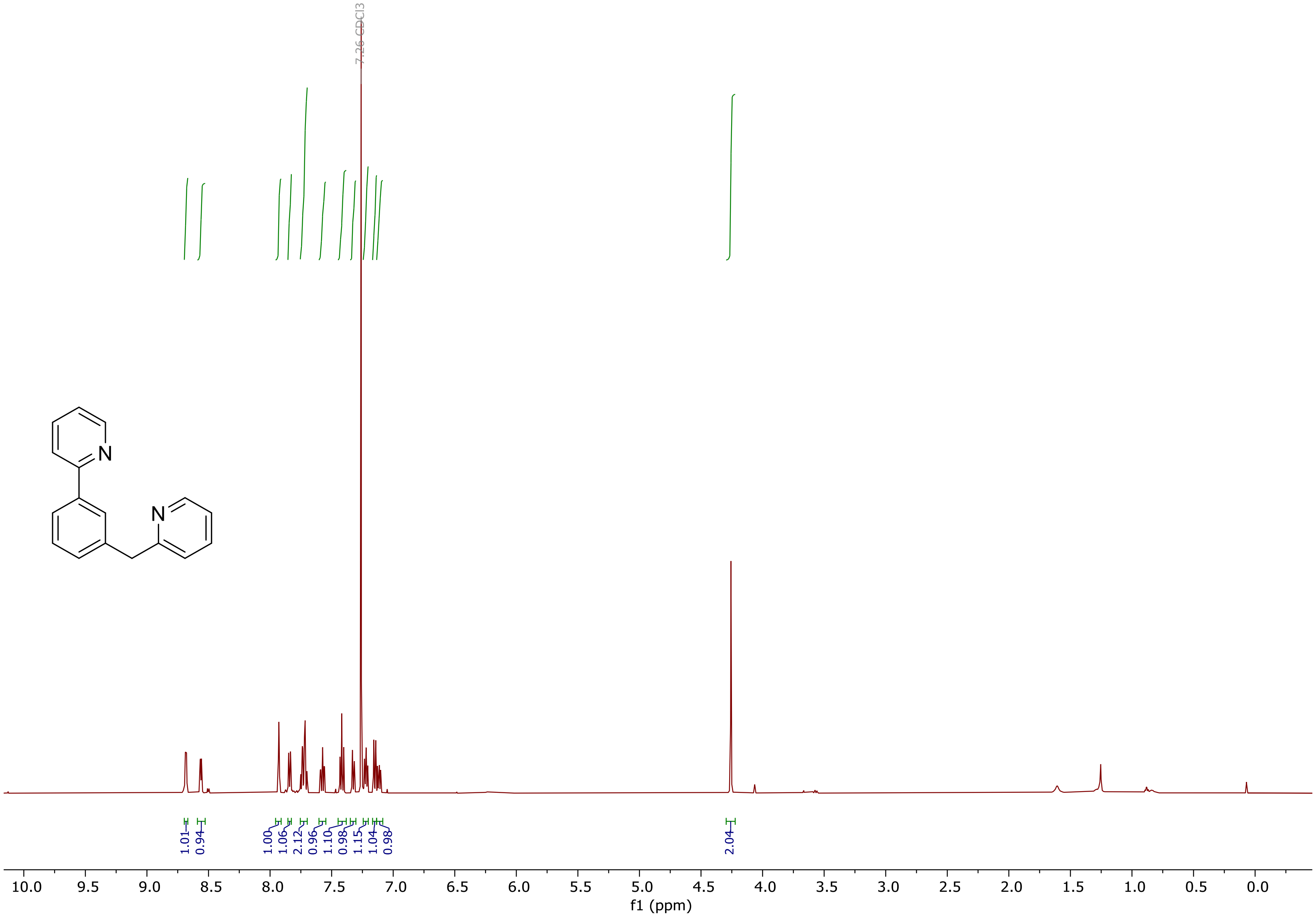

Supplementary Figure 251. ^{1}H NMR (500 MHz, $CDCl_3$) of 2-[3-(pyridin-2-yl)benzyl]pyridine **23**.

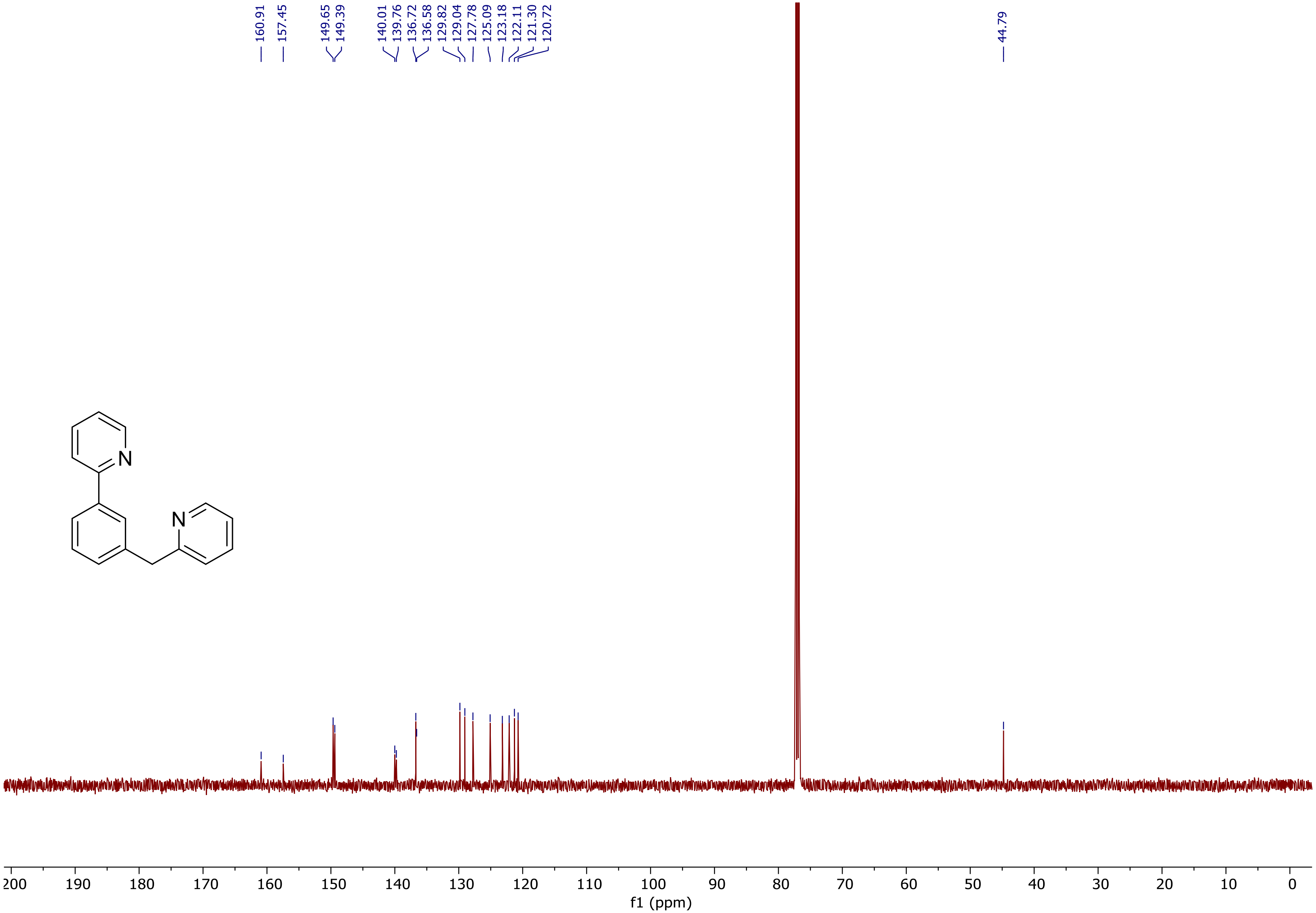

Supplementary Figure 252. ^{13}C NMR (126 MHz, $CDCl_3$) of 2-[3-(pyridin-2-yl)benzyl]pyridine **23**.

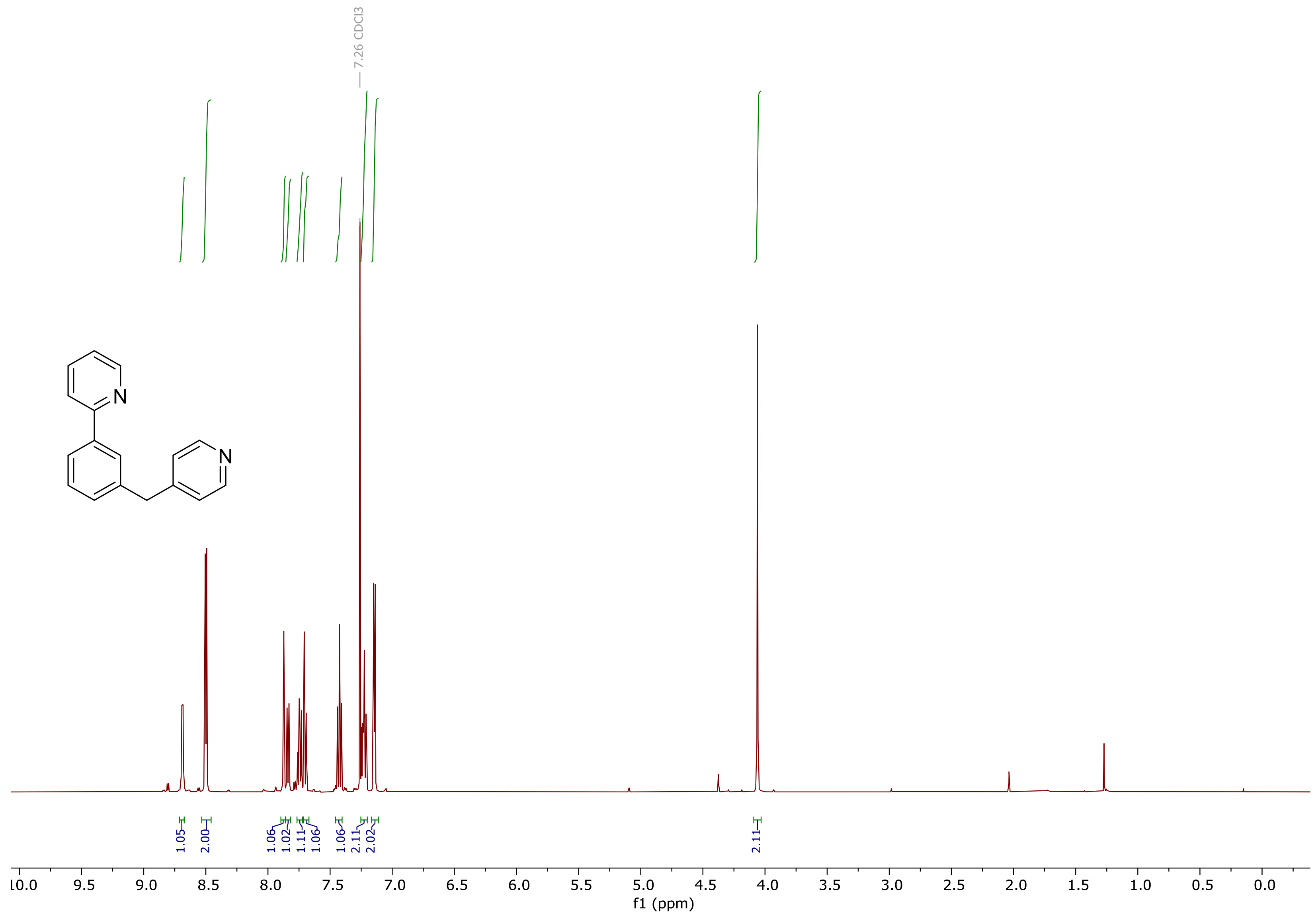

Supplementary Figure 253. ^{1}H NMR (500 MHz, $CDCl_3$) of 2-[3-(pyridin-4-ylmethyl)phenyl]pyridine **24**.

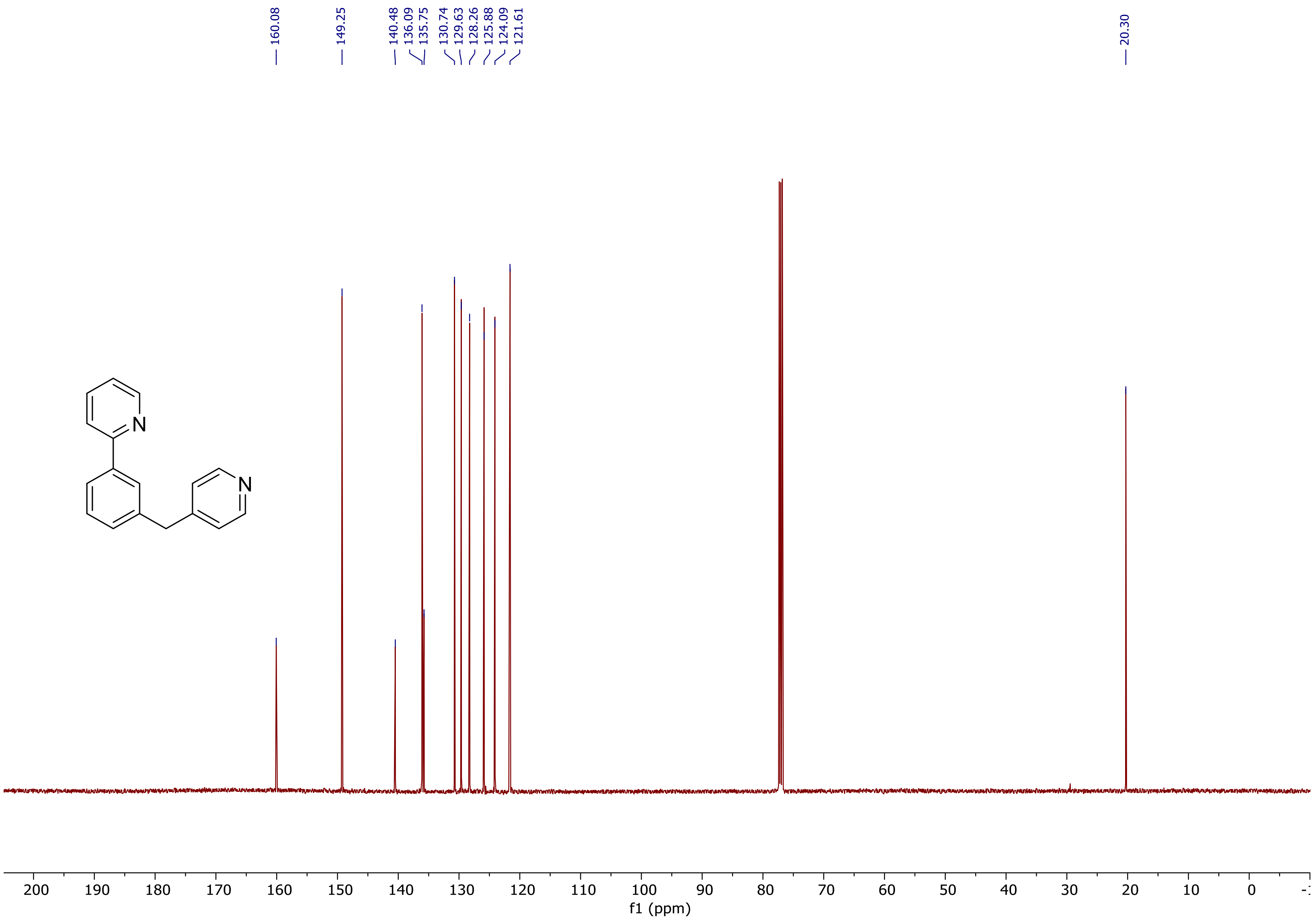

Supplementary Figure 254. ^{13}C NMR (126 MHz, $CDCl_3$) of 2-[3-(pyridin-4-ylmethyl)phenyl]pyridine **24**.

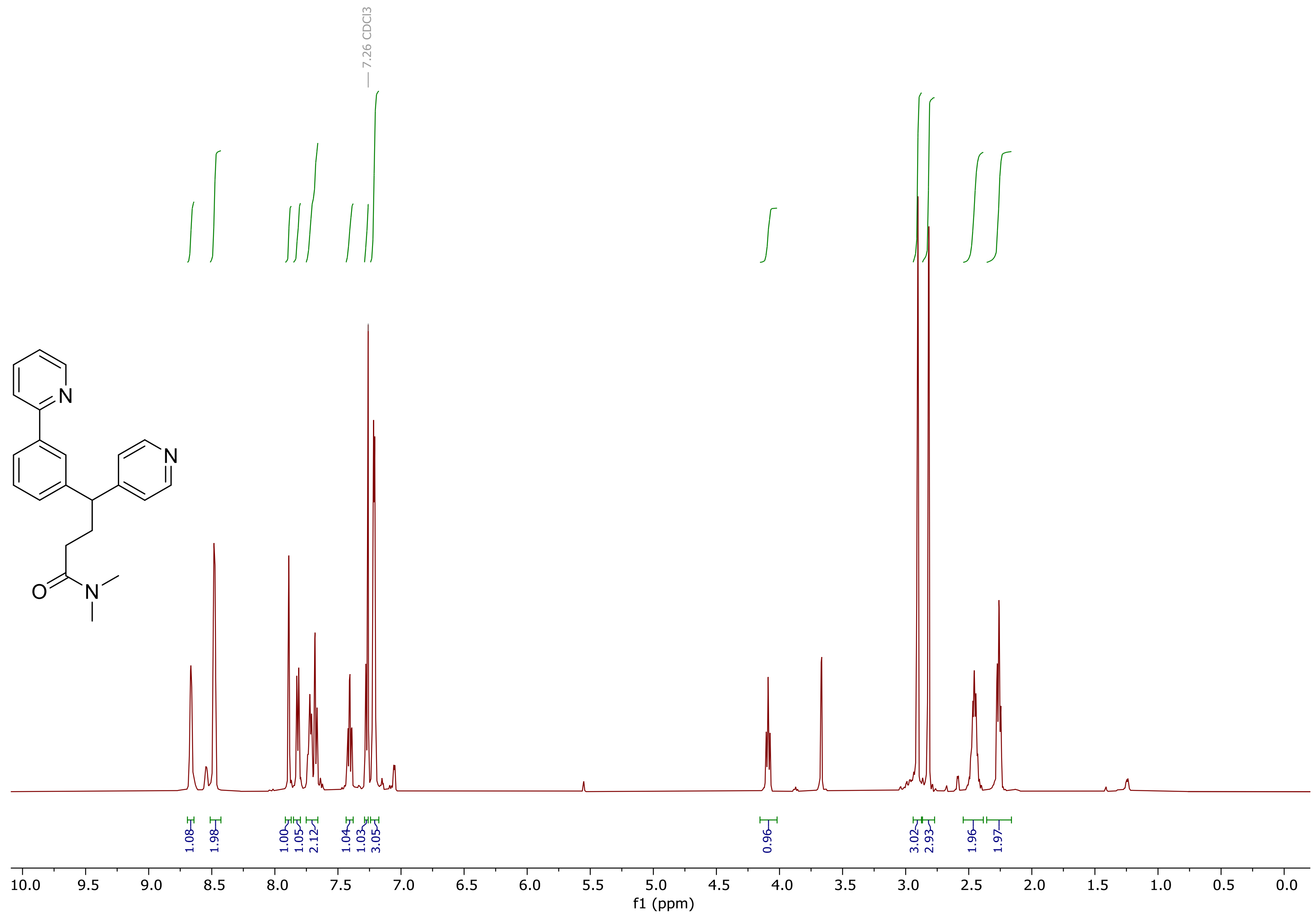

Supplementary Figure 255. ^{1}H NMR (500 MHz, $CDCl_3$) of *N,N*-dimethyl-4-[3-(pyridin-2-yl)phenyl]-4-(pyridin-4-yl)butanamide **26**.

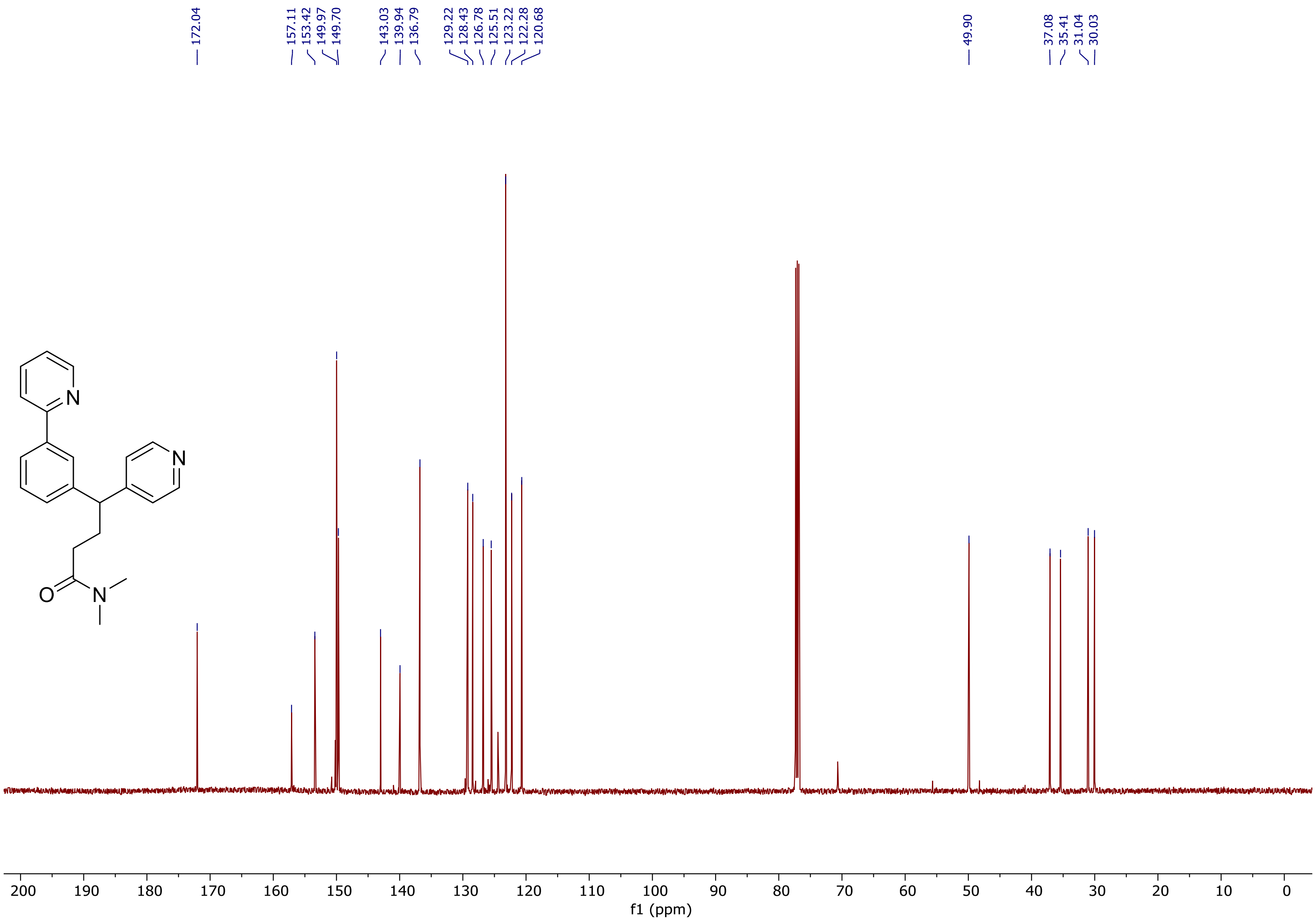

Supplementary Figure 256. ^{13}C NMR (126 MHz, $CDCl_3$) of *N,N*-dimethyl-4-[3-(pyridin-2-yl)phenyl]-4-(pyridin-4-yl)butanamide **26**.

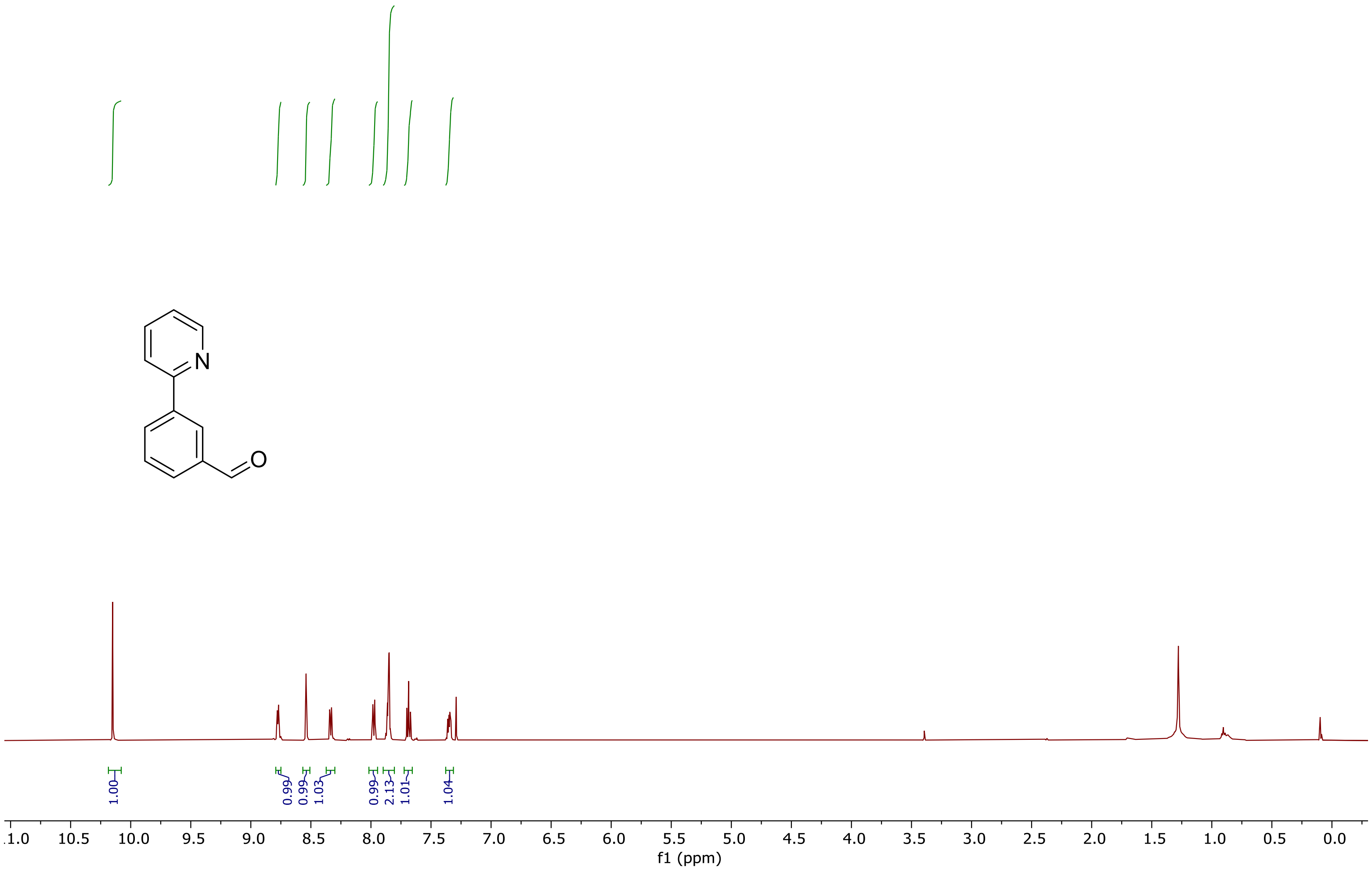

Supplementary Figure 257. ^{1}H NMR (500 MHz, $CDCl_3$) of 3-(pyridine-2-yl)benzaldehyde **27**.

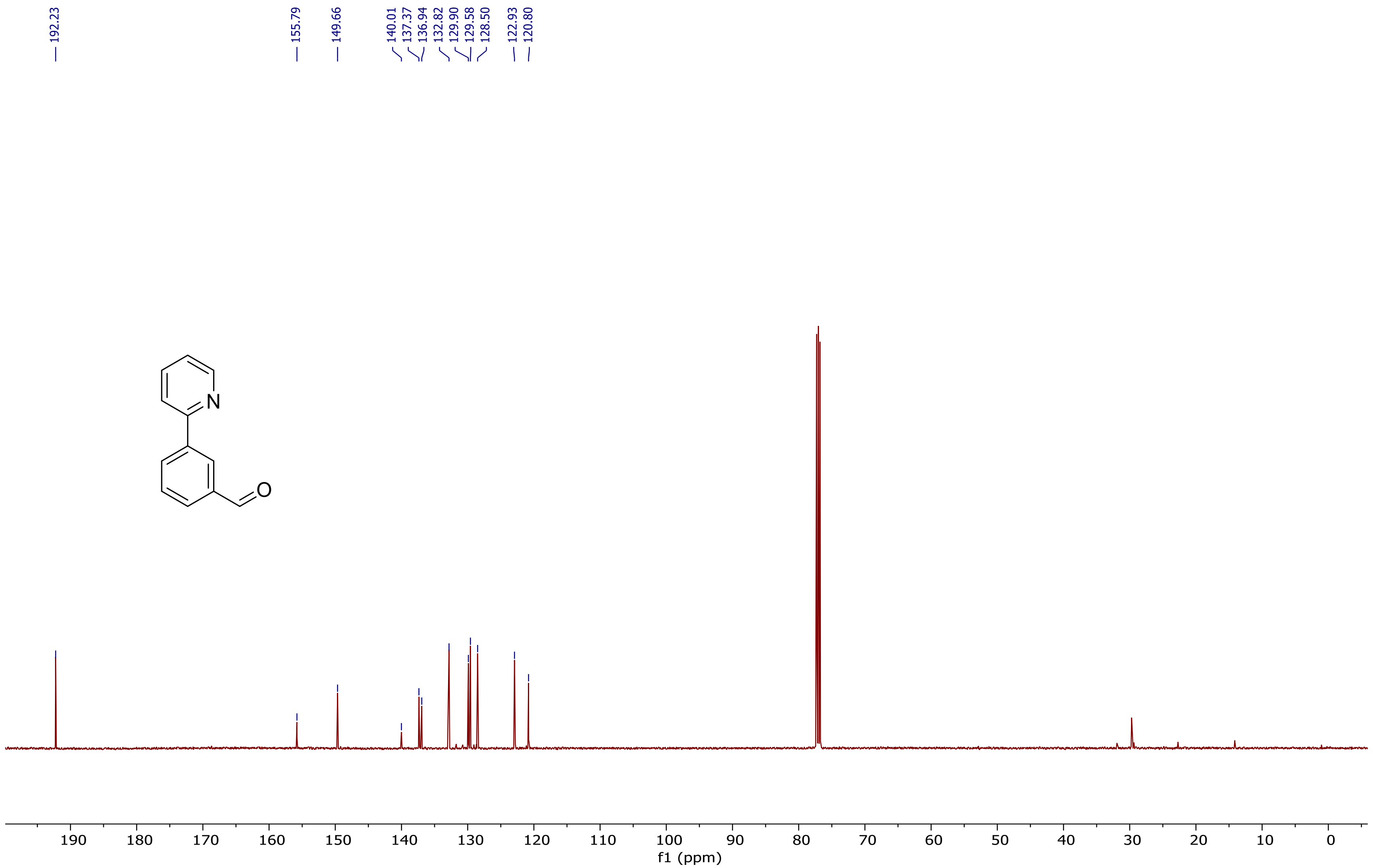

Supplementary Figure 258. ^{13}C NMR (126 MHz, $CDCl_3$) of 3-(pyridine-2-yl)benzaldehyde **27**.

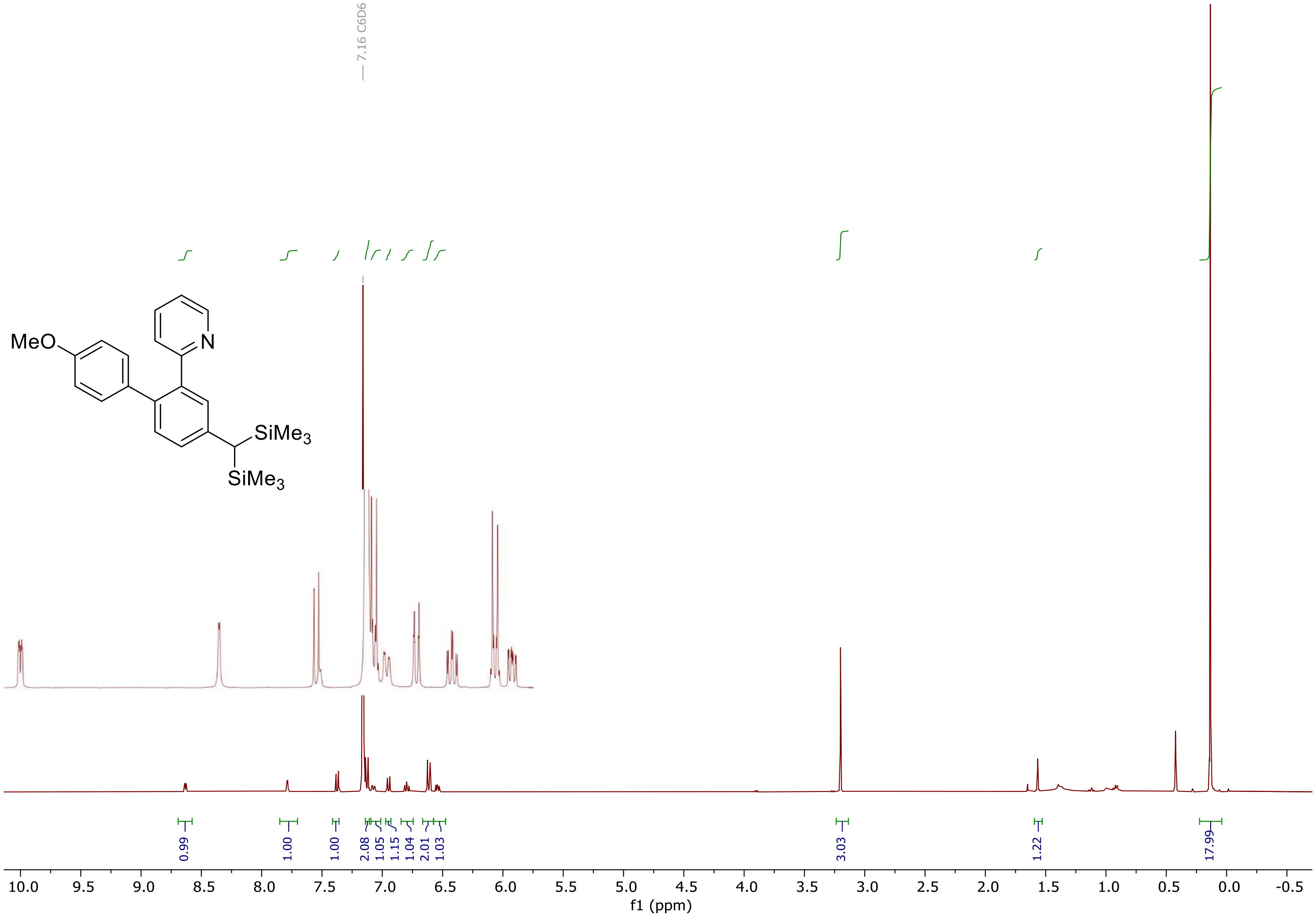

Supplementary Figure 259. ^{1}H NMR (400 MHz, C_6D_6) of 2-{4-[bis(trimethylsilyl)methyl]-4'-methoxy-(1,1'-biphenyl)-2-yl}pyridine **28**.

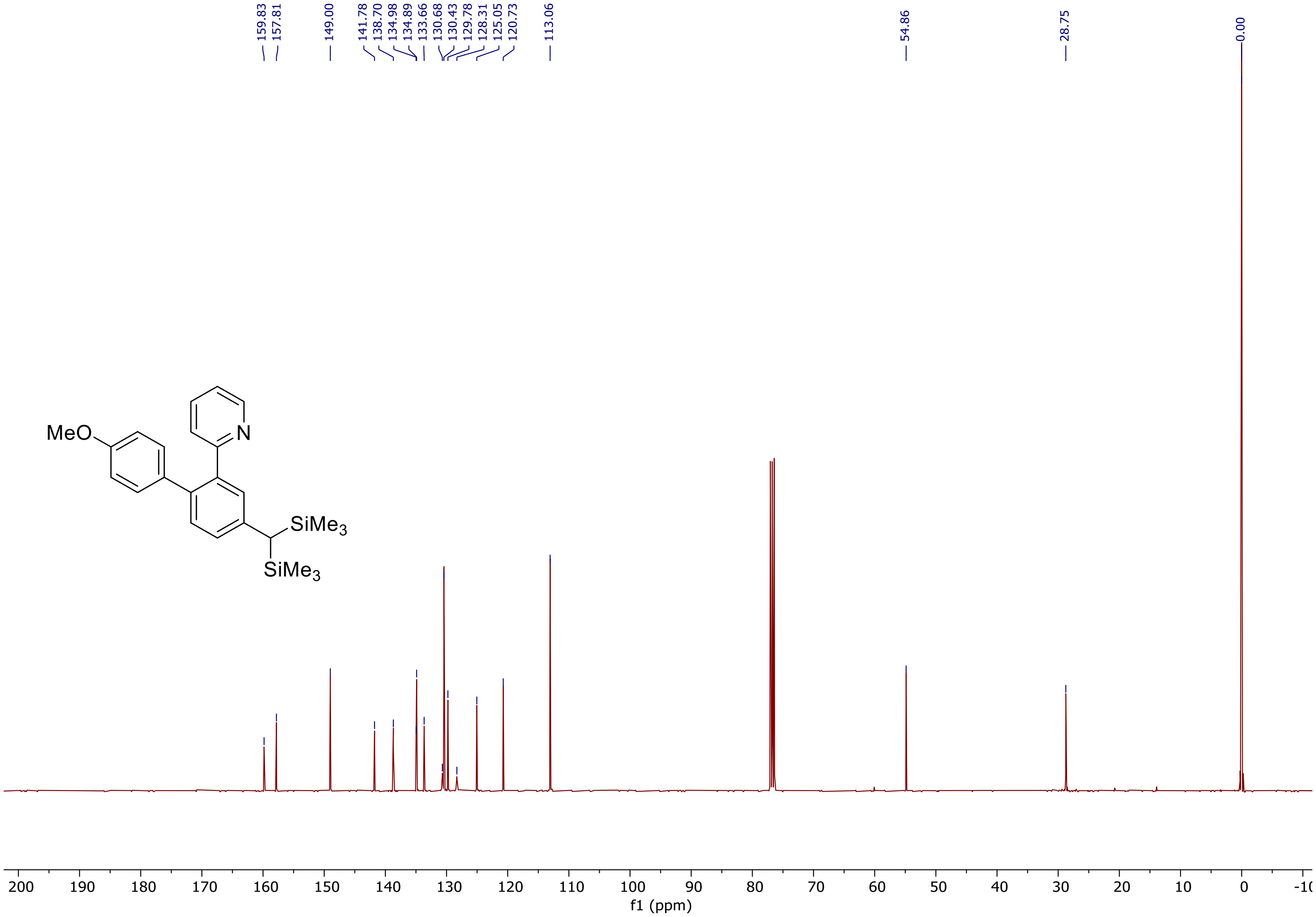

Supplementary Figure 260. ^{13}C NMR (101 MHz, $CDCl_3$) of 2-{4-[bis(trimethylsilyl)methyl]-4'-methoxy-(1,1'-biphenyl)-2-yl}pyridine **28**.

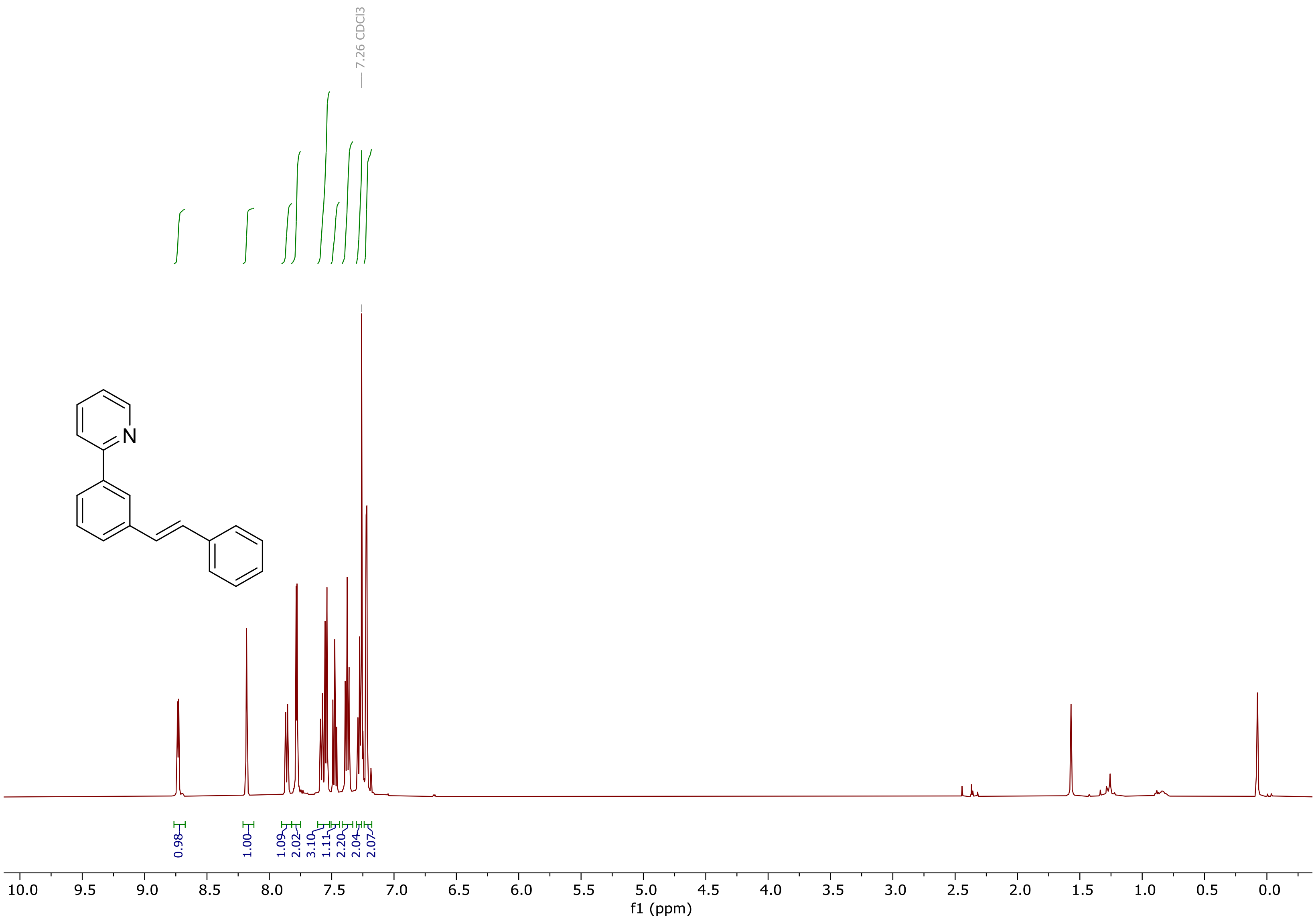

Supplementary Figure 261. ^{1}H NMR (500 MHz, $CDCl_3$) of (*E*)-2-(3-styrylphenyl)pyridine **29**.

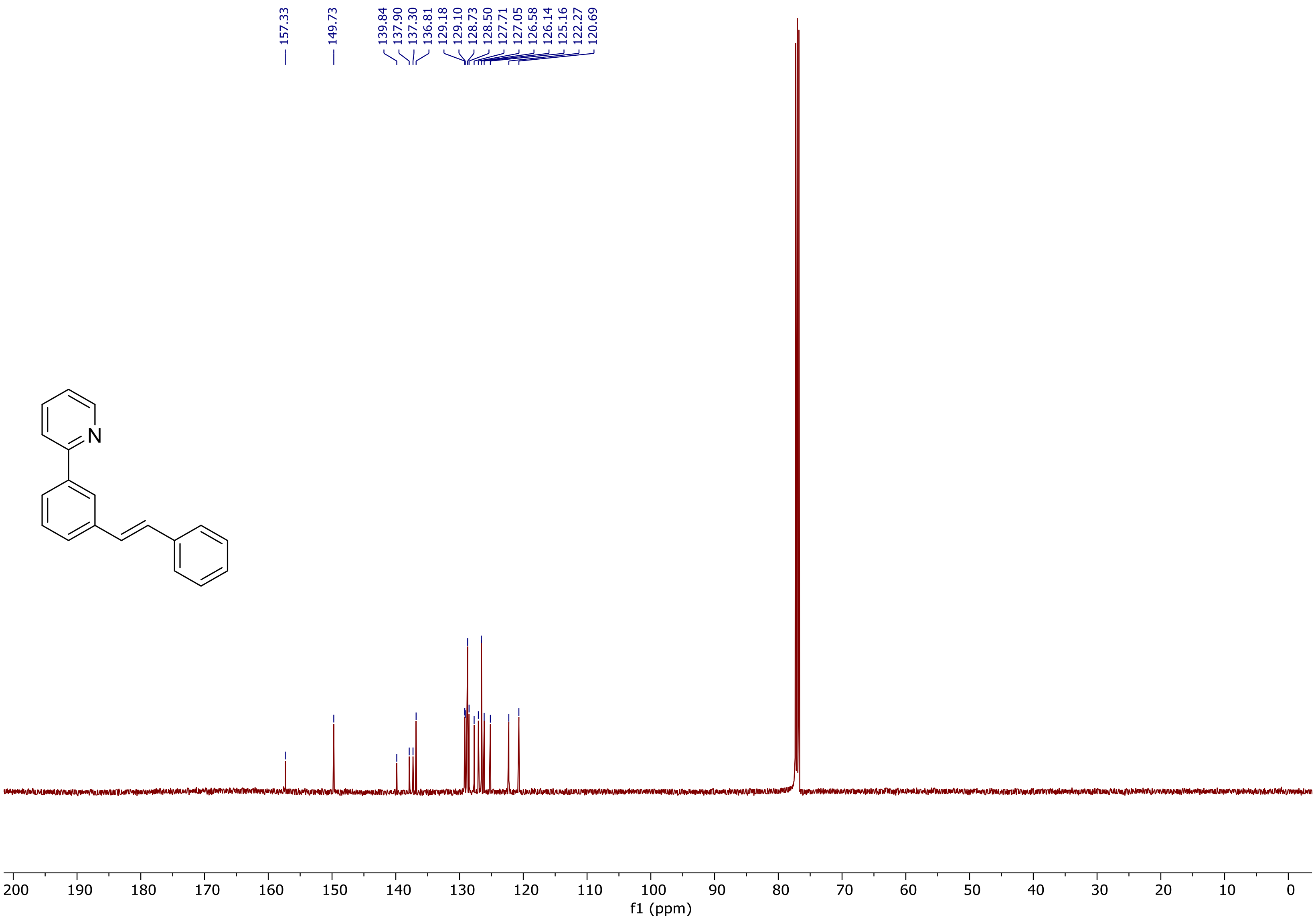

Supplementary Figure 262. ^{13}C NMR (126 MHz, $CDCl_3$) of (*E*)-2-(3-styrylphenyl)pyridine **29**.

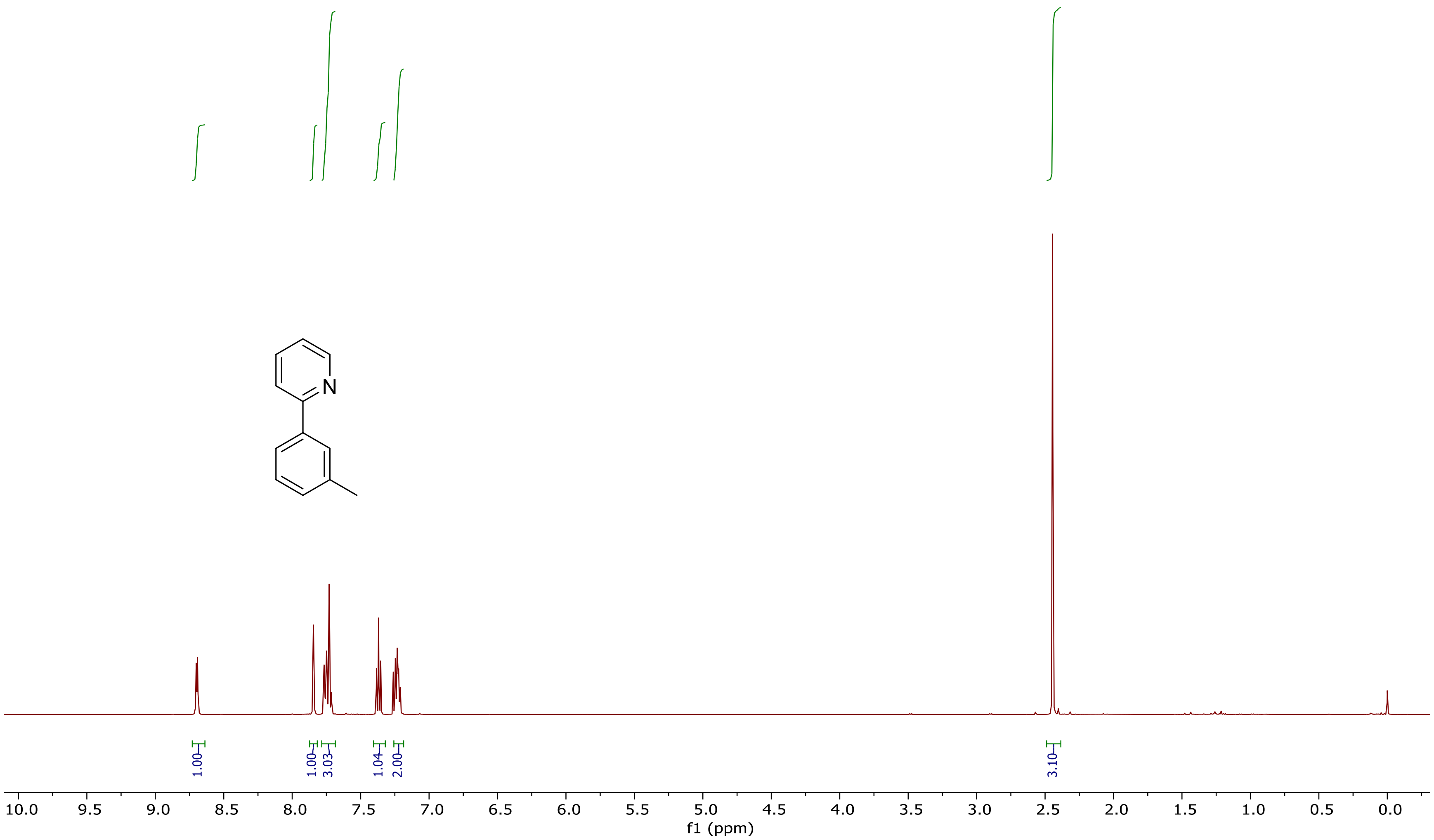

Supplementary Figure 263. ^{1}H NMR (500 MHz, $CDCl_3$) of 2-(m-tolyl)pyridine **30**.

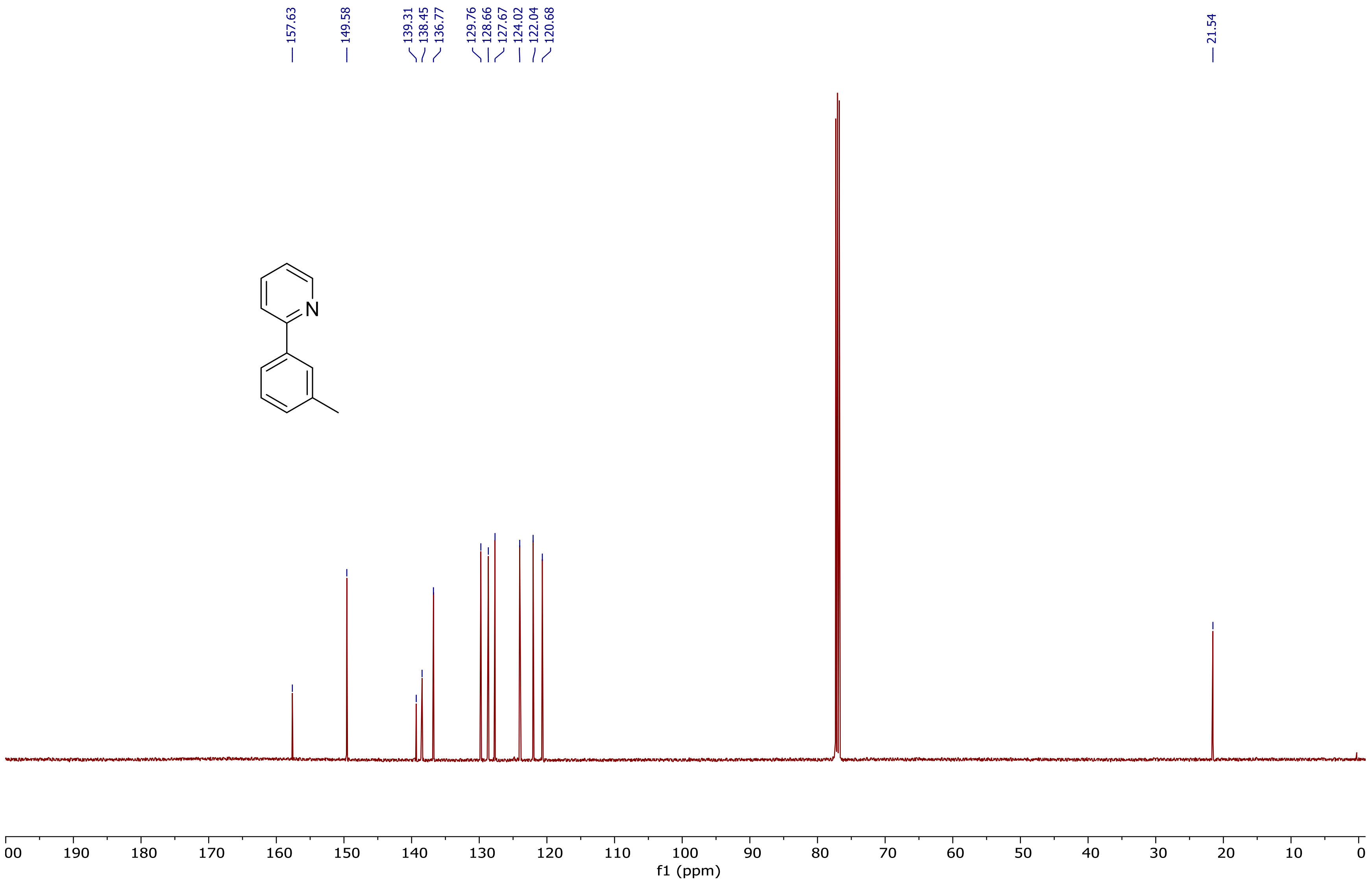

Supplementary Figure 264. ^{13}C NMR (126 MHz, $CDCl_3$) of 2-(m-tolyl)pyridine **30**.

X-ray crystallography data

Single crystal X-ray diffraction data compounds **31** and **40** were collected using a Rigaku FR-X diffractometer equipped with a HE6000Hypix and a graphite-monochromated Cu-Kα (λ = 1.54178 Å) radiation at 100 K. Single crystal X-ray diffraction data compounds **36** were collected using a Rigaku Supernova diffractometer equipped with a Eos CCDC detector and a graphite-monochromated Mo-Kα (λ = 0.71073 Å) radiation at 100 K. Data were collected and processed using CrysAlisPro suite of programmes. Intensities were integrated from data recorded on 1° scan width frames by ω rotation. Cell parameters were refined from the observed peak positions of all strong reflections in each data set. A Gaussian grid face-indexed were used to correct for X-ray absorption using Abstrac3 software in CrysAlisPro. The phase problem were solved using SHELXT[10]. The crystallographic models were refined by full-matrix least-squares on all unique F^2 values using SHELXL[11] implemented in OLEX2 software.[12] Atomic displacement parameters were refined anisotropically for all the atoms; Hydrogen atom positions were constrained using riding hydrogen geometries; and atomic displacement parameters were set at 1.2 (1.5 for methyl groups) times Ueq of the parent atom. The largest features in final difference syntheses were close to heavy atoms and were of no chemical significance. Disordered PF_6 anions were modelled over two positions and occupancies were refined. The P-F distances were restrained to be the same using SHELX SADI command and the atomic displacement parameters were also restrained to be similar using SHELX SIMU commands. CCDC 2383351 (**36**) and 2384328-2384329 (**31** and **40**) contain the supplementary crystal data for this article. These data can be obtained free of charge from the Cambridge Crystallographic Data Centre *via* www.ccdc.cam.ac.uk/data_request/cif

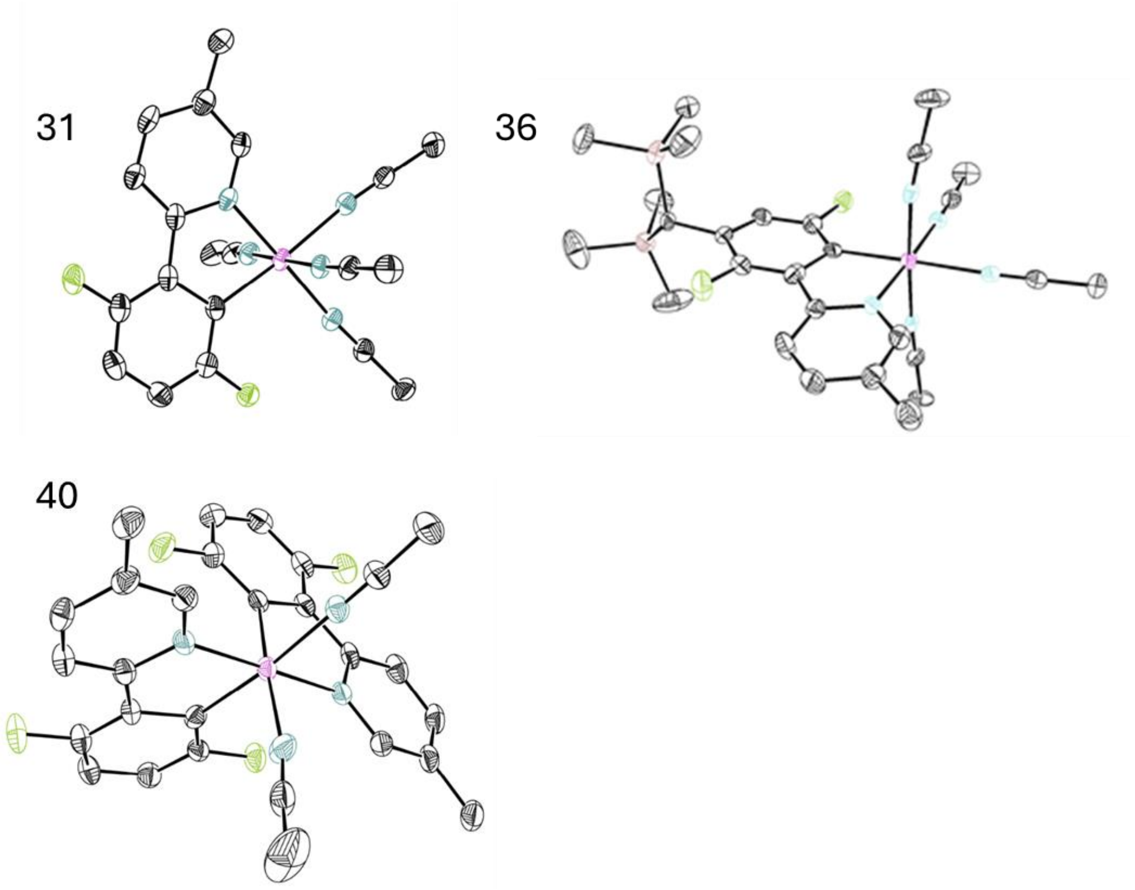

Supplementary Figure 265. XRD structures of compounds **31, 36** and **40** ellipsoids shown at 50% probability and counterions removed.

Supplementary Table 17. Crystal data and structure refinement for compounds **31**, **36** and **40**

Identification code	**31**	**36**	**40**
CCDC reference	2384328	2383351	2384329
Empirical formula	$C_{24}H_{26}F_{8}N_{7}PRu$	$C_{29}H_{41}F_{8}N_{6}PRuSi_{2}$	$C_{28}H_{22}F_{4}N_{4}Ru$
Formula weight	696.56	813.90	591.56
Temperature/K	100.01(14)	99.99(10)	100.0(3)
Crystal system	monoclinic	monoclinic	monoclinic
Space group	$P2_1/c$	$P2_1/n$	$P2_1/c$
a/Å	11.4756(2)	12.3190(3)	17.5882(4)
b/Å	32.5925(6)	8.3837(2)	8.9137(3)
c/Å	7.97290(10)	36.4192(8)	17.0925(5)
α/°	90	90	90
β/°	101.552(2)	96.318(2)	110.695(3)

γ/°	90	90	90
Volume/Å^3	2921.61(9)	3738.49(15)	2506.79(13)
Z	4	4	4
ρ_{calc}g/cm^3	1.584	1.446	1.567
μ/mm^{-1}	5.579	0.595	5.539
F(000)	1400.0	1664.0	1192.0
Crystal size/mm^3	0.12 × 0.11 × 0.1	0.44 × 0.14 × 0.12	0.05 × 0.04 × 0.02
Radiation	Cu Kα (λ = 1.54184)	Mo Kα (λ = 0.71073)	Cu Kα (λ = 1.54184)
2Θ range for data collection/°	5.422 to 151.846	6.754 to 58.432	5.372 to 152.212
Index ranges	-14 ≤ h ≤ 12, -40 ≤ k ≤ 40, -10 ≤ l ≤ 10	-16 ≤ h ≤ 15, -8 ≤ k ≤ 11, -47 ≤ l ≤ 48	-21 ≤ h ≤ 21, -11 ≤ k ≤ 8, -21 ≤ l ≤ 14
Reflections collected	37872	29808	12986
Independent reflections	6010 [R_{int} = 0.0932, R_{sigma} = 0.0550]	8879 [R_{int} = 0.0693, R_{sigma} = 0.0884]	5066 [R_{int} = 0.0379, R_{sigma} = 0.0507]
Data/restraints/parameters	6010/147/441	8879/0/436	5066/0/339
Goodness-of-fit on F^2	1.076	1.129	1.092
Final R indexes [I>=2σ (I)]	R_1 = 0.0476, wR_2 = 0.1285	R_1 = 0.0772, wR_2 = 0.1373	R_1 = 0.0473, wR_2 = 0.1283
Final R indexes [all data]	R_1 = 0.0569, wR_2 = 0.1356	R_1 = 0.1012, wR_2 = 0.1472	R_1 = 0.0548, wR_2 = 0.1329
Largest diff. peak/hole / e Å^{-3}	1.98/-0.70	2.50/-1.64	1.78/-1.58

Supplementary References

1. Xi, L-Y., Zhang, R-Y., Liang, S., Chen, S-Y. & Yu, X-Q. Copper-Catalyzed Aerobic Synthesis of 2-Arylpyridines from Acetophenones and 1,3-Diaminopropane. *Org. Lett.* **16**, 5269–5271 (2014).

2. Darabantu, M., Boully, L., Turck, A. & Plé, N. Synthesis of new polyaza heterocycles. Part 42: Diazines. *Tetrahedron* **61**, 2897–2905 (2005).

3. Pawar, A. B. & Chang, S. Cobalt-Catalyzed C–H Cyanation of (Hetero)arenes and 6-Arylpurines with N-Cyanosuccinimide as a New Cyanating Agent. *Org. Lett.* **17**, 660–663 (2015).

4. Song, S., Sun, X., Li, X., Yuan, Y. & Jiao, N. Efficient and Practical Oxidative Bromination and Iodination of Arenes and Heteroarenes with DMSO and Hydrogen Halide: A Mild Protocol for Late-Stage Functionalization. *Org. Lett.* **17**, 2886–2889 (2015).

5. Tian, P. & Tong, R. In-water oxidative Suzuki coupling of arenes and arylboronic acids using H_2O_2 as a terminal oxidant. *Green Chem.* 2**5**, 1345–1350 (2023).

6. Popov, I., Do, H.Q. & Daugulis, O. In Situ Generation and Trapping of Aryllithium and Arylpotassium Species by Halogen, Sulfur, and Carbon Electrophiles. *J. Org. Chem.* **74**, 8309–8313 (2009).

7. Hogg, A., Wheatley, M., Domingo-Legarda, P., Carral-Menoyo, A., Cottam, N. & Larrosa, I. Ruthenium-Catalyzed Monoselective C–H Methylation and d3-Methylation of Arenes. *JACS Au* **2**, 2529–2538 (2022).

8. Jia, C., Wu, N., Cai, X., Li, G., Zhong, L., Zou, L. & Cui, X. Ruthenium-Catalyzed meta-Selective CAr—H Bond Formylation of Arenes. *J. Org. Chem.* **85**, 4536–4542 (2020).

9. Hu, Y., Gao, Y., Ye, J., Ma, Z., Feng, J., Liu, X., Lei, P. & Szostak, M. Suzuki–Miyaura Cross-Coupling of 2-Pyridyl Trimethylammonium Salts by N–C Activation Catalyzed by Air- and Moisture-Stable Pd–NHC Precatalysts: Application to the Discovery of Agrochemicals. *Org. Lett.* **25**, 2975–2980 (2023).

10. Sheldrick, G. M. SHELXT – Integrated space-group and crystal-structure determination. *Acta Cryst.* **A71**, 3–8 (2015).

11. Sheldrick, G. M. Crystal structure refinement with SHELXL. *Acta Cryst.* **C71**, 3–8 (2015).

12. Dolomanov, O. V., Bourhis, L. J., Gildea, R. J., Howard, J. A. K. & Puschmann, H. OLEX2: a complete structure solution, refinement and analysis program. *J. Appl. Cryst.* **42**, 339–341

(2009).
